# Regioselective Markovnikov hydrodifluoroalkylation of alkenes using difluoroenoxysilanes

**DOI:** 10.1038/s41467-020-19387-4

**Published:** 2020-10-30

**Authors:** Xiao-Si Hu, Jun-Xiong He, Su-Zhen Dong, Qiu-Hua Zhao, Jin-Sheng Yu, Jian Zhou

**Affiliations:** 1 Shanghai Engineering Research Center of Molecular Therapeutics and New Drug Development, Shanghai Key Laboratory of Green Chemistry and Chemical Processes, East China Normal University, Shanghai, 200062 China; 2 Key Laboratory of Tropical Medicinal Resource Chemistry of Ministry of Education, Hainan Normal University, Haikou, 571158 China; 3 State Key Laboratory of Organometallic Chemistry, Shanghai Institute of Organic Chemistry, CAS, Shanghai, 200032 China

**Keywords:** Catalytic mechanisms, Homogeneous catalysis, Synthetic chemistry methodology

## Abstract

Alkene hydrodifluoroalkylation is a fruitful strategy for synthesizing difluoromethylated compounds that are interesting for developing new medicinal agents, agrochemicals, and advanced materials. Whereas the anti-Markovnikov hydrodifluoroalkylation to linear-type products is developed, employing radical-based processes, the Markovnikov synthesis of branched adducts remains unexplored. Herein, we describe acid-catalyzed processes involving carbocation intermediates as a promising strategy to secure the Markovnikov regioselectivity. Accordingly, the Markovnikov hydrodifluoroalkylation of mono-, di-, tri-, and tetrasubstituted alkenes using difluoroenoxysilanes, catalyzed by Mg(ClO_4_)_2_·6H_2_O, is achieved. This allows the diversity-oriented synthesis of α,α-difluoroketones with a quaternary or tertiary carbon at the β-position that are otherwise difficult to access. The method is applied to the modification of natural products and drug derivatives. The resulting α,α-difluorinated ketones could be converted to the corresponding α,α-difluorinated esters or alcohols, or organofluorine compounds featuring a CF_2_H or CF_2_CF_2_Ph moiety. Mechanistic studies support that Mg(ClO_4_)_2_·6H_2_O functions as a hidden Brønsted acid catalyst.

## Abstract (web summary)

Alkene hydrodifluoroalkylation is a fruitful strategy for synthesizing medicinally relevant difluoromethylated molecules. Here, the authors report a Markovnikov hydrodifluoroalkylation of aryl and aliphatic alkenes using inexpensive Mg(ClO_4_)_2_·6H_2_O as catalyst and difluoroenoxysilanes as the nucleophiles.

## Introduction

Organofluorine compounds featuring a fluoroalkyl group have found widespread application in pharmaceutical, agrochemical, and material science^1–3^ because a fluorine moiety often brings about beneficial effects on the physical, chemical, and pharmaceutical properties of organic compounds^4–7^. While a trifluoromethyl group is typically used to modulate the properties of molecules^8–12^, increasing attention is being paid to the selective incorporation of a difluoromethyl group (CF_2_H) or a difluoromethylene fragment^13,14^ because of the capacity of CF_2_H as a lipophilic H-bond donor^15^ and the isosteric relationship between the CF_2_ moiety and ethereal oxygen^16^. In particular, with the advent of difluoromethylated drugs such as pantoprazole^17^, eflornithine^18^, and gemcitabine^19^, efficient methods for selective difluoroalkylation are very much in demand in the field of drug discovery and development. Therefore, it is of current interest to develop the diversity-oriented synthesis of difluoroalkyl-containing molecules from readily accessible starting materials in an operationally simple manner.

In this context, hydrodifluoroalkylation of alkenes is an important strategy due to the abundance and diversity of alkenes as carbon feedstocks in organic synthesis. In parallel to the advances made in hydrotrifluoromethylation^20–24^ and hydroperfluoroalkylation^25–27^ of simple alkenes, much progress has also been made in hydrodifluoroalkylation^28–38^. A number of elegant protocols with anti-Markovnikov regioselectivity have been developed using different sources of CF_2_, thereby allowing the facile synthesis of linear-type difluoroalkylated adducts. Notable examples hereof include the following. Qing et al. used a bromodifluoromethylphosphonium salt to realize a visible-light-induced hydrodifluoromethylation of terminal alkenes^34^. Jui described the hydrodifluoroalkylation of alkenes using difluorobenzylic radicals produced in situ via the C–F cleavage of ArCF_3_^36,37^. Most recently, Gouverneur et al. accomplished an economic protocol using inexpensive difluoroacetic acid as the difluoromethyl reagent, under the action of 3 equiv. PhI(OAc)_2_ and visible light^38^.

**Fig. 1:**
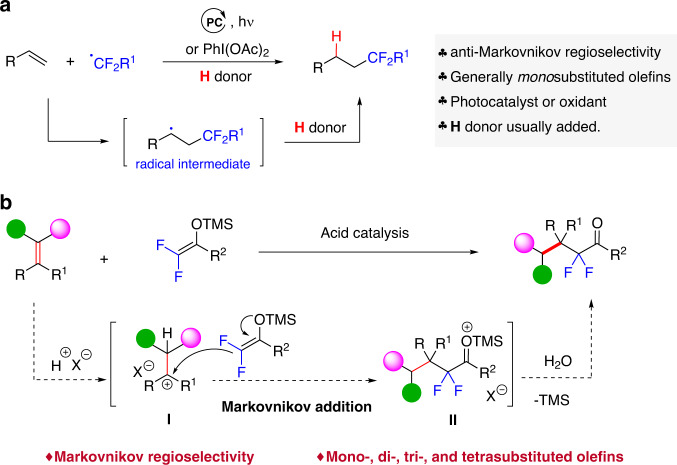
State of the art and our proposed hydrodifluoroalkylation of simple alkenes. **a** Anti-Markovnikov alkene hydrodifluoroalkylation based on radical processes (well established). **b** Markovnikov alkene hydrodifluoroalkylation (this work).

Despite significant advances, Markovnikov hydrodifluoroalkylation of alkenes remains a largely unsolved challenge. Currently, all the known protocols afford linear adducts with anti-Markovnikov regioselectivity. This is because these protocols rely on radical processes that involve the formation of more stable radicals after the addition of in situ-generated difluoroalkyl radicals to alkenes (Fig. 1a). However, Markovnikov hydrodifluoroalkylation would yield branched adducts with a chemical space shape distinct from linear products.

In view of the intimate relationship between the properties of organic molecules and their shape^39,40^, branched adducts are therefore of significant interest to the scientific community. If 1,1-di-, tri-, and tetrasubstituted alkenes are used, Markovnikov regioselectivity will allow the construction of difluoroalkylated quaternary carbons. Because the selective incorporation of quaternary carbons to enhance conformational restriction has been recognized as an effective strategy to improve the properties of bioactive compounds^40–43^, it seems only logical that molecules bearing a difluoromethylated quaternary carbon are interesting targets for medicinal research. With these considerations in mind, it was postulated highly desirable to develop the Markovnikov hydrodifluoroalkylation of alkenes. However, such research requires several challenges to be overcome, including control of the regioselectivity—as yet, unattainable by any known radical strategy—and the increased steric hindrance in the C–C bond-forming step between a more substituted alkene carbon and the fluorinated reagents.

To address these challenges, we speculated that the acid-catalyzed hydrodifluoroalkylation of alkenes involving carbocation intermediates offers a promising solution (Fig. 1b). In principle, this approach is characterized by the initial protonation of the double bond of alkenes to form the more stable carbocation according to the Markovnikov rule, followed by a C–C bond-forming reaction with difluoroalkylated nucleophiles. Such a mechanism provides the regioselectivity complementary to known anti-Markovnikov radical reactions and, beneficially, substantially expands the scope of alkenes. Current hydrodifluoroalkylation reactions are largely based on monosubstituted aliphatic alkenes, with very limited examples of aryl alkenes^31,33,34^, disubstituted alkenes^30–34,36–38^, and trisubstituted alkenes^32,36,37^ being incorporated. Furthermore, to our knowledge, tetrasubstituted alkenes have never been used. In particular, aryl alkenes still present a challenge as a substrate class for radical olefin hydrofluoroalkylation, due to unproductive side reactions such as overoxidation and dimerization^21,23^. In contrast, processes involving carbocations may be useful for aryl alkenes and polysubstituted alkenes because either aryl or aliphatic substituents could stabilize the carbocation intermediate, and the steric hindrance expected for the initial protonation of alkenes is much less than that for the addition of fluoroalkyl radical (Fig. 1b vs. a). This could offer a promising approach to access structurally diverse difluoroalkylated adducts from a broad scope of alkenes, including those with a difluoroalkylated quaternary carbon.

Surprisingly, despite these attractive features, acid-catalyzed olefin hydrodifluoroalkylation remains unexplored, possibly because alkyl carbocation intermediates (strong acids) are usually unstable—they are apt to undergo deprotonation, substitution, and various side reactions with olefins, such as alkylation, dimerization, and oligomerization^44–47^.

Therefore, we speculate that the key to reaction development is balancing the acid strength and the activity of the difluoro reagent, allowing the rate of carbocation generation to match the C–C bond-forming reaction. To minimize side reactions, the acid catalyst should be able to produce the carbocation from alkenes under mild conditions, and the difluoroalkylated nucleophile should react with the in situ-generated carbocation, once produced, at least at a substantially faster rate than the occurrence of side reactions.

Here, we report the use of Mg(ClO_4_)_2_·6H_2_O as an effective hidden Brønsted acid to achieve the Markovnikov hydrodifluoroalkylation of mono-, di-, tri-, and tetrasubstituted alkenes using difluoroenoxysilanes, affording valuable α-difluoromethylated ketones that were rarely prepared via olefin hydrodifluoroalkylation^35^ (Fig. 1b).

## Results

### Optimization of the reaction conditions

Easily available difluoroenoxysilane **2**^48^ has been established as a versatile difluoro reagent^49–57^, to introduce an α,α-difluorinated ketone moiety that can undergo various diversifying reactions. However, its application in alkene hydrodifluoroalkylation remains unexplored. With our efforts in selective difluoroalkylation^50^, we have developed a variety of functionalization reactions of **2**, including catalyst-free-on-water aldol reaction^52^, olefination using diazo reagents^53^, and Michael addition to tetrasubstituted electron-deficient alkenes^54^. These studies brought the following to our attention: difluoroenoxysilanes **2** are often more reactive than their nonfluorinated analogs^50^, which are with high nucleophilicity comparable to allylstannanes^58^; furthermore, to some extent, they are compatible with water^52^ and the reaction conditions that include superacids such as HOTf and HClO_4_^50^. This suggested to us that **2** could be a promising reagent in developing acid-catalyzed Markovnikov hydrodifluoroalkylation, affording structurally diverse α-branched α,α-difluorinated ketones as fluorinated synthons, as well as interesting targets for medicinal researches^59^.

**Fig. 2:**
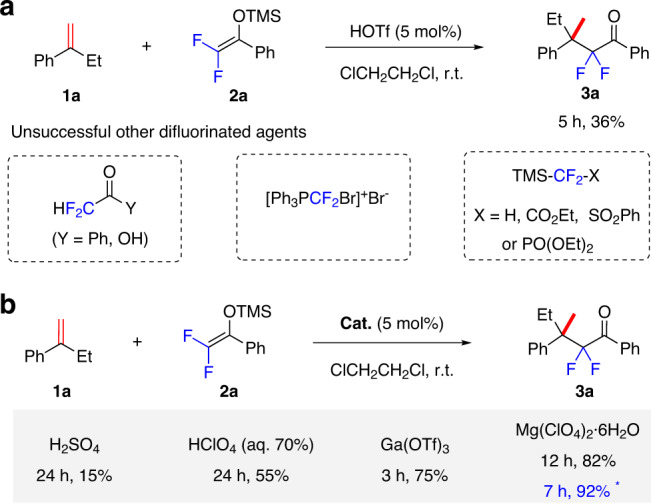
The development of Markovnikov hydrodifluoroaklylation of alkenes. **a** Performance of different difluoro reagents. **b** Comparing different acid catalysts. *With 10 mol% Mg(ClO_4_)_2_·6H_2_O.

Accordingly, we tried the reaction of 1,1-disubstituted α-ethylstyrene **1a** with difluoroenoxysilane **2a** at room temperature (r.t.) in the presence of 5 mol% HOTf—a superacid widely used to generate carbocations^44–47^ (Fig. 2a). To our delight, the reaction finished within 5 h to afford branched adduct **3a** in 36% yield. We further examined several other typical difluoroagents previously used for alkene hydrodifluoroalkylation, including difluoroacetic acid, bromodifluoromethylphosphonium bromide, and trimethylsilyl (TMS)-based difluorinated esters or sulfones. In these cases, no target products were detected, under the same conditions mentioned above, besides an olefin dimerization product (determined by gas chromatography–mass spectrometry analysis).

Because extensive olefin dimerization occurred, we next tried optimizing the acid catalysts in an effort to improve the yield—this emerged as being crucial (Fig. 2b). Ordinary strong Brønsted acids such as *p*-TsOH and CF_3_CO_2_H failed to give the desired product **3a**; only H_2_SO_4_ afforded **3a** in 15% yield. Another common superacid, HClO_4_, used as a 70% aqueous solution, improved the yield of **3a** to 55%, but the reaction time was longer. This indicated that a small amount of water could be tolerated. Because the reaction was performed in air, a trace amount of water should be present, which might bind to a Lewis acid to give a conjugate acid to then generate a carbocation from the alkene^44–47,60^. We therefore next examined the performance of Lewis acids.

Of the metal triflates we screened, Fe(OTf)_3_, Sc(OTf)_3_, Ph_3_PAuOTf, and Ga(OTf)_3_ could mediate this reaction. Ga(OTf)_3_ proved to be the best; **3a** was obtained in 75% yield after 3 h. However, the highly hygroscopic nature of triflates made it difficult to reproduce the result. We therefore turned our attention to using the easier-to-handle metal perchlorate hydrates. After intensive optimization, Mg(ClO_4_)_2_·6H_2_O was identified as the catalyst of choice; it afforded **3a** in a reproducible 82% yield, which was further improved to 92% if using 10 mol% catalyst. For details of optimization of conditions, see the Supplementary Information.

### Scope with respect to different alkenes

**Fig. 3:**
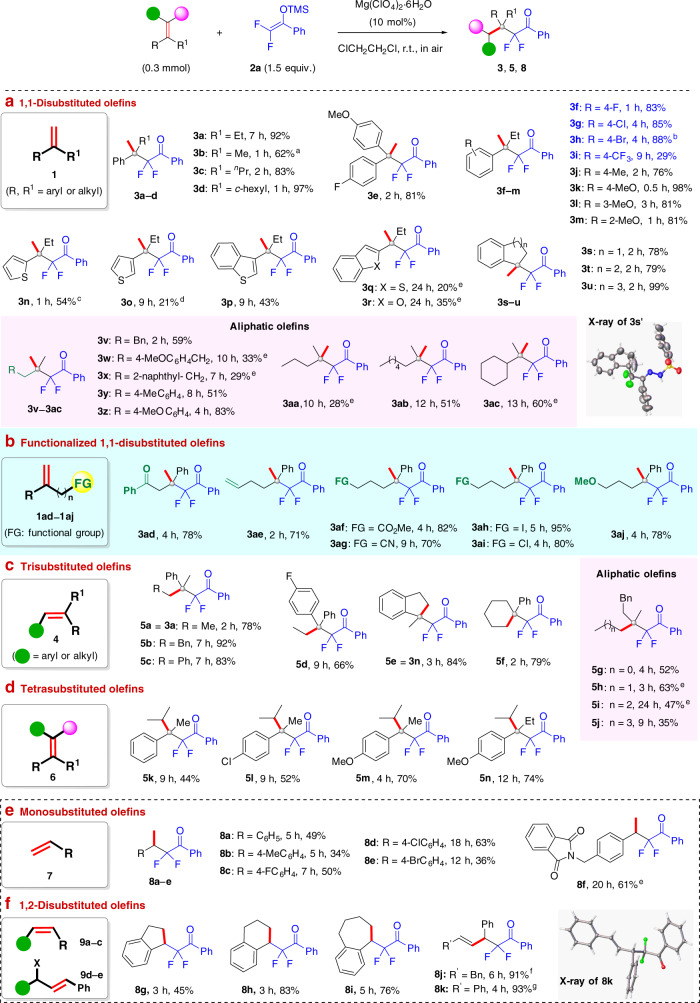
Scope of the Markovnikov hydrodifluoroalkylation with respect to different alkenes. The structure of each numbered alkene is shown in the Supplementary Information. Reaction conditions: alkenes (0.3 mmol), **2a** (0.45 mmol), Mg(ClO_4_)_2_·6H_2_O (10 mol%), and ClCH_2_CH_2_Cl (3 mL), in air at room temperature. ^a^In all, 3 mol% Mg(ClO_4_)_2_‧6H_2_O, 3 equiv. **2a**. ^b^At 50 °C. ^c^In all, 5 mol% Mg(ClO_4_)_2_‧6H_2_O, 3 equiv. **2a**. ^d^In all, 1 mol% Mg(ClO_4_)_2_‧6H_2_O, 3 equiv. **2a**. ^e^In all, 20 mol% Mg(ClO_4_)_2_‧6H_2_O, 3 equiv. **2a**. ^f^(*E*)-*N*-(1,4-diphenylbut-3-en-2-yl)-4-methyl benzenesulfonamide **9d** was used. ^g^(*E*)-((1,3-diphenylallyl)oxy) trimethylsilane **9e** was used.

With the optimized condition in hand, the scope of this Markovnikov hydrodifluoroalkylation was determined (Fig. 3). First, the synthesis of α,α-*gem*-difluoro-β-quaternary ketones **3a–3aj** from 1,1-disubstituted alkene **1** were examined. Styrenes with either an α-alkyl or α-aryl group all worked well to afford products **3a–e** in 62–97% yields. 2-Aryl-1-butene with different α-aryl and α-heteroaryl substituents afforded the products **3f–r** in 20–98% yields. Exocyclic 1,1-disubstituted alkenes afforded the corresponding ketones **3s–u** in 78–99% yields. The structure of **3s** was confirmed by the X-ray diffraction analysis of its hydrazine derivative **3s′** (see the Supplementary Information for details). Alkenes with various α-positioned aliphatic groups were also viable substrates; they afforded products **3v–3ac** in 28–83% yields. Notably, functionalized 1,1-disubstituted alkenes bearing a ketone, alkenyl, ester, cyano, halogen atom, or ether group all afforded the target adducts **3ad–3aj** in 70–95% yields.

Remarkably, the scope of this hydrodifluoroalkylation could be extended to polysubstituted alkenes. Trisubstituted styrenes, with aryl or alkyl substituents, reacted with difluoroenoxysilane **2a** to afford the desired adducts **5a–d** in 66–92% yields. Trisubstituted endocyclic olefins also afforded products **5e** and **5f** in 84% and 79% yield, respectively. Besides aryl-substituted olefins, trialkyl-substituted alkenes were also viable substrates, affording the desired products **5g–j** in reasonable yields. Notably, tetrasubstituted olefin **6**, never before used for hydrofluoroalkylation, worked well with **2a** to afford the target adducts **5k–n** in 44–74% yields.

Aside from the diversity-oriented construction of difluoroalkylated quaternary carbons, this method is workable for the synthesis of α,α-difluoroalkyl ketone **8** with a tertiary carbon from mono- and 1,2-disubstituted alkenes **7** and **9**. Monosubstituted alkene **7**, *cis*-1,2-disubstituted olefins such as indene, dihydronaphthalene, and 6,7-dihydro-5*H*-benzocycloheptene all readily afforded the corresponding adducts **8a–i** in 34–83% yields. Interestingly, *N*-Ts allyl amine **9d** or *O*-TMS-protected allyl alcohol **9e**, with a *trans*-1,2-disubstituted alkene, afforded difluoroalkylated ketones **8j** and **8k** in 91% and 93% yield, respectively, possibly via an S_N_1′ reaction pathway. The structure of **8k** was further confirmed by X-ray crystallographic analysis.

These results strongly support our hypothesis that can secure Markovnikov regioselectivity complementary to radical processes. Furthermore, it promisingly incorporates the use of a broad range of alkenes, including mono-, di-, tri-, and tetrasubstituted ones, with good functional group compatibility. Noticeably, various aryl alkenes, which are problematic substrates for radical processes, afforded the desired branched adducts in generally good-to-excellent yields.

### Scope of fluorinated silyl enol ethers

**Fig. 4:**
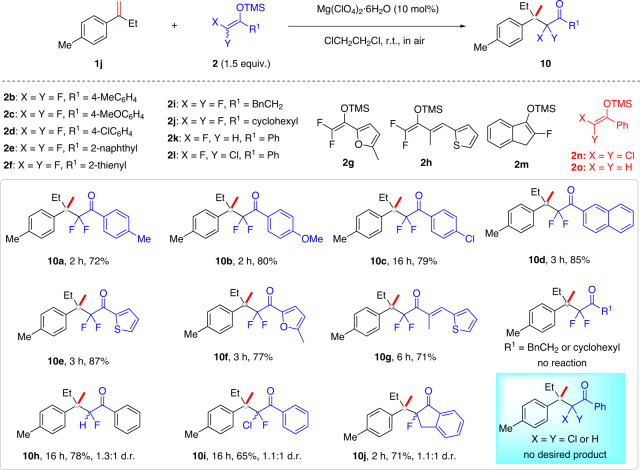
Scope of silyl enol ether 2 and the fluorine effect. Reaction conditions: alkene **1j** (0.3 mmol), **2** (0.45 mmol), Mg(ClO_4_)_2_‧6H_2_O (10 mol%), and ClCH_2_CH_2_Cl (3 mL) in air at room temperature.

Next, various difluoroenoxysilanes **2** were examined. Those that have methyl, methoxy, or chloro groups as substituents on the phenyl ring reacted with alkene **1j** to provide products **10a–c** in 72–80% yields (Fig. 4). Difluoroenoxysilanes **2e**–**h** bearing a 2-napthyl, thienyl, 5-methylfuryl, or alkenyl group were also competent substrates, affording products **10d–g** in 71–87% yields. However, reactions with the aliphatic difluoroenoxysilanes **2i**, **j** failed. Notably, such acid catalysis was also workable for Markovnikov hydromonofluoroalkylation. Here, both acyclic and cyclic monofluorinated silyl enol ethers^61,62^ were viable substrates, as exemplified by the synthesis of monofluoroalkylated ketones **10h–j** in good yields, albeit with low diastereoselectivity (d.r.).

Fluorine substitution plays a key role in this olefin hydroalkylation; both the α,α-dichlorinated silyl enol ether **2n** and the nonfluorinated analog **2o** failed to produce any desired products, under the same conditions (Fig. 4). This observation is in accordance with our previous observations in the aldol and olefination reactions^50,52,53^. The failure of nonfluorinated silyl enol ether **2o** was not because it was hydrolyzed in a faster rate than difluoroenoxysilane **2a** under the standard condition (for details, see the Supplementary Information). It took 12 h for **2o** to be fully hydrolyzed, but no desired product could be detected by GC–MS analysis of the reaction mixture. However, at present, the origin of such dramatic fluorine effects^63–65^ is unclear.

### Late-stage functionalization

**Fig. 5:**
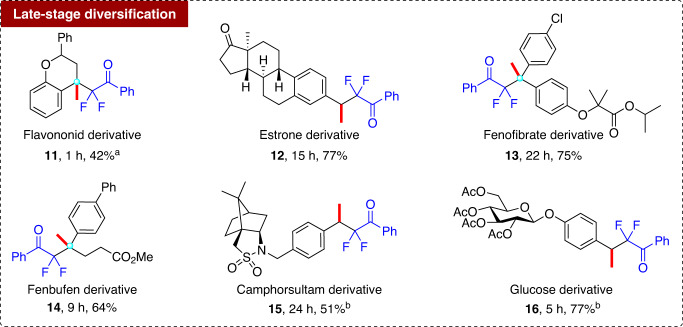
Late-stage hydrodifluoroalkylation of natural products and drug derivatives. Reaction conditions: alkenes (0.3 mmol), **2a** (0.45 mmol), Mg(ClO_4_)_2_‧6H_2_O (10 mol%), and ClCH_2_CH_2_Cl (3 mL) in air at room temperature. ^a^In all, 3 mol% Mg(ClO_4_)_2_‧6H_2_O, 3 equiv. **2a**. ^b^In all, 20 mol% Mg(ClO_4_)_2_‧6H_2_O, 3 equiv. **2a**.

The high functional group tolerance of this acid-mediated hydrodifluoroalkylation reaction offers a promising strategy for modifying olefinic derivatives of natural products, drugs, and carbohydrates. As shown in Fig. 5, treatment of a natural product flavononid analog with **2a** under the optimized conditions afforded the difluoroalkylated flavononid derivative **11** in 42% yield. The hydrodifluoroalkylation of several drug olefinic derivatives from estrone, fenofibrate, and fenbufen delivered the corresponding difluoro analogs **12–14** in 64–77% yields. Furthermore, the difluoroketone moiety could be smoothly introduced into the camphorsultam scaffold and a carbohydrate to afford **15** (51% yield) and **16** (77% yield) via this hydrodifluoroalkylation.

### Gram-scale synthesis and synthetic utility

**Fig. 6:**
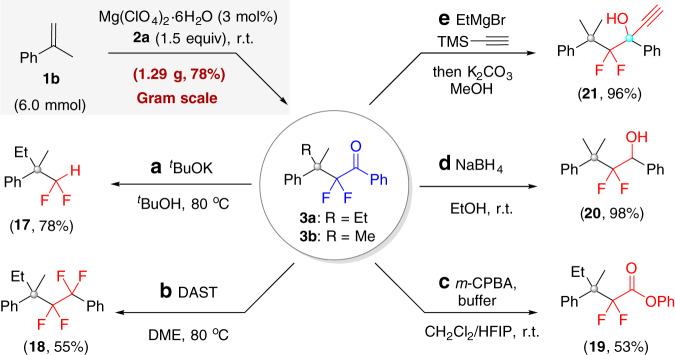
Gram-scale synthesis and transformations of products. DAST diethylaminosulfur trifluoride, DME 1,2-dimethoxyethane, *m*-CPBA *m*-chloroperoxybenzoic acid, HFIP 1,1,1,3,3,3-hexafluoro-2-propanol.

To showcase the synthetic application of this methodology further, a Gram-scale reaction of α-methylstyrene **1b** with **2a** was conducted (Fig. 6). Under the catalysis of 3 mol% Mg(ClO_4_)_2_·6H_2_O at r.t., 1.29 g of **3b** was obtained in 78% yield—better than the 62% yield of the small-scale reaction reported earlier (see Fig. 3). The resulting ketone **3** is a versatile difluoro synthon capable of undergoing a variety of diversifying reactions. For instance, upon treatment with *tert*-BuOK or diethylaminosulfur trifluoride, the α,α-difluorinated benzoyl moiety of **3a** could be converted to a CF_2_H or CF_2_CF_2_Ph moiety, which has important applications in drugs, agrochemicals, and advanced materials^15,66^. Furthermore, the ketone moiety of **3** could be transformed into an ester or an alcohol via oxidation, reduction, or a nucleophilic addition reaction, as demonstrated by the synthesis of ester **19**, secondary alcohol **20**, and tertiary alcohol **21** bearing an ethynyl group.

Considering that α,α-difluoroketones are interesting targets for the development of pharmaceutical agents^59^, we initially evaluated the in vitro cytotoxic activity of some samples in human colorectal cancer cells (HCT116) using CCK-8 assay. We were delighted to find that the selected compounds had good growth-inhibitory activity at 30 µM and the half-maximal inhibitory concentration (IC_50_) values of cytotoxic effects ranged from 4.22 to 58.25 µM, depending on their structures (see the Supplementary Information for details). These preliminary results demonstrate that the thus obtained α-branched difluorinated ketones are potentially valuable in anticancer drug development, and once again, lay stress on the importance of diversity-oriented synthesis of α,α-difluoroketones.

### Preliminary mechanistic study

The inexpensive catalyst, mild conditions, simple and easy-to-handle reaction system, broad scope, and good functional group tolerance make this Markovnikov hydrodifluoroalkylation reaction potentially useful. These attractive features intrigued us to investigate the possible reaction mechanism. First, to examine whether Mg(ClO_4_)_2_·6H_2_O acted as a hidden Brønsted acid^67,68^ in the reaction, the addition of 10 or 20 mol% noncoordinating base 2,6-di-*tert*-butylpyridine **22** to the reaction of **1a** and **2a** was undertaken. Almost no reaction occurred, even when the reaction time was extended to 20 h.

**Fig. 7:**
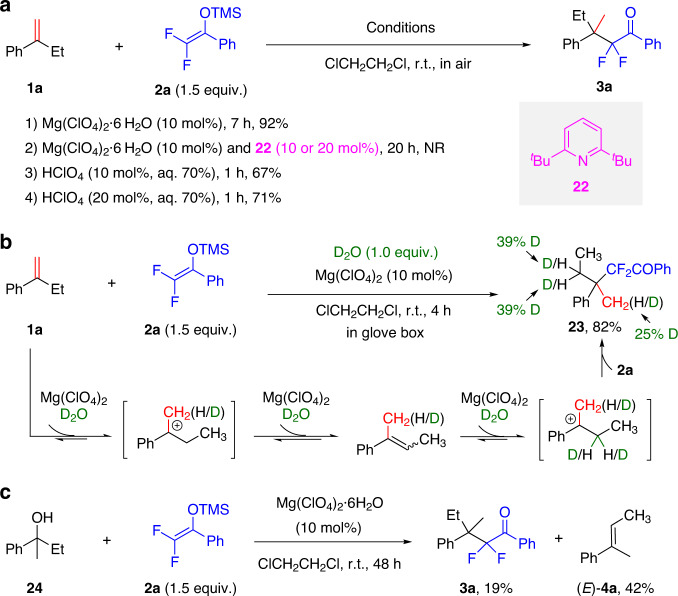
Mechanistic study. **a** Control experiments. **b** Experiments with D_2_O. **c** Experiments with tertiary alcohol **24**.

On the other hand, the model reaction proceeded smoothly in the presence of 10 or 20 mol% HClO_4_ (aq. 70%), affording product **3a** in 67% or 71% yield, respectively, within 1 h (Fig. 7a). The reaction catalyzed by 10 mol% HClO_4_ (aq. 70%) proceeded at a much faster rate than that catalyzed by Mg(ClO_4_)_2_·6H_2_O, but the yield of **3a** was lower (1 h, 67% yield vs. 7 h, 92% yield). These findings are fully in accordance with the concept of a hidden Brønsted acid catalyst^67,68^, supporting the idea that the HClO_4_ released from Mg(ClO_4_)_2_·6H_2_O is, in reality, the active catalyst. This decreases the hydrolysis of difluoroenoxysilane and the dimerization of alkenes by slowing down the reaction rate, through gradually releasing HClO_4_ from Mg(ClO_4_)_2_·6H_2_O, and thereby ensuring a higher yield, compared with the direct use of HClO_4_ (aq. 70%) as the catalyst.

The proton source for this hydrodifluoroalkylation was then investigated. Because of the absence of external agents as the proton source, we questioned whether the proton came from the crystal water of Mg(ClO_4_)_2_·6H_2_O. If so, the use of a combination of anhydrous Mg(ClO_4_)_2_ with deuterium oxide (D_2_O) would give the deuterated difluoroalkylated product. We indeed found that the reaction of α-ethylstyrene **1a** with **2a** worked well in the presence of 10 mol% anhydrous Mg(ClO_4_)_2_ and 1 equiv. D_2_O, to furnish the deuterated product **23** in 82% yield (Fig. 7b). This result suggested that the proton in this hydrodifluoroalkylation mainly originates from the crystal water of Mg(ClO_4_)_2_·6H_2_O. Meanwhile, partial proton transfer might originate from the trace amount of water in the reaction system, thus decreasing the ratio of deuterated product **23**. Unfortunately, when 2 equiv. of D_2_O was added, almost no alkylated adduct was isolated. Besides, under the standard conditions, tertiary alcohol **24** reacted with **2a** to afford product **3a** in 19% yield, as well as the elimination product olefin (*E*)-**4a** in 42% yield (Fig. 7c), further demonstrating that the reaction under consideration proceeds via the carbocation intermediate. The lower yield in this case was in accordance with the above-observed deleterious effect of water, because one equiv. of water was in situ-generated from the elimination. Based on these results, we came to a conclusion that the reaction could only tolerate trace amount of water, the crystal water of the catalyst, and that in the atmosphere of the reaction vessel (totally less than 2 equiv.), possibly because the presence of excess water will trap the carbocation intermediate, or lead to the hydrolysis of difluoroenoxysilane dominated.

## Discussion

In conclusion, we have realized a regioselective Markovnikov hydrodifluoroalkylation of simple alkenes with difluoroenoxysilanes, which is fully complementary to the well-established anti-Markovnikov radical processes. This represents a straightforward and effective strategy to access valuable α-difluoroalkylated ketones with a quaternary or tertiary carbon at the β-position. Remarkably, the mild reaction conditions, the inexpensive and easy-to-handle catalyst, and the broad scope of alkenes, including mono-, di-, tri-, and tetrasubstituted alkenes, make our methodology potentially very useful. The value of this methodology is further demonstrated by the late-stage diversification of natural products and drug derivatives, as well as versatile product transformations to functionalized fluorine-containing molecules. Preliminary biological studies indicate that these difluorinated ketones are promising therapeutic agents for colorectal cancer. The application of this methodology and the development of a catalytic enantioselective version is currently ongoing in our laboratory.

## Methods

General procedure for the hydrodifluoroalkylation of simple alkenes. Under an air atmosphere, to a 5.0-mL vial were added Mg(ClO_4_)_2_·6H_2_O (9.9 mg, 0.03 mmol, 10 mol%) and anhydrous ClCH_2_CH_2_Cl (3.0 mL), followed by the addition of alkenes (0.3 mmol, 1.0 equiv.) and fluorinated silyl enol ether **2** (0.45 mmol, 1.5 equiv.). The resulting mixture was stirred at room temperature until full consumption of alkenes by TLC analysis. The reaction mixture was then concentrated under reduced pressure. The crude residue was purified by flash column chromatography to provide the desired products. Full experimental details and characterization of compounds can be found in the Supplementary Information.

### Caution

Attention should be paid when handling perchlorate salts because they are potentially explosive when used in the presence of combustible substances at high temperature. It has been documented that magnesium perchlorate has high thermal stability (>300–500 °C), and actually can be dried under vacuum at 160 °C without any accident^69,70^. In addition, exposure to nominal levels of such perchlorate does not adversely affect health and safety, as evidenced by The National Fire Protection Association that ranks magnesium perchlorate as barely hazardous for health, and as an oxidizing product but not as an explosive one.

## Supplementary information

Supplementary information

## Data Availability

X-ray crystallographic data for compound **3s′** (CCDC 1938421) and **8k** (CCDC 1938418), are freely available from the Cambridge Crystallographic Data Centre. Copies of the data can be obtained free of charge via https://www.ccdc.cam.ac.uk/structures/. All other data in support of the findings of this study are available within the article and its Supplementary Information or from the corresponding author upon reasonable request.
