# Supplementary material for: Regioselective Markovnikov hydrodifluoroalkylation of alkenes using difluoroenoxysilanes
Source: Nat Commun. 2020 Oct 30;11:5500. doi: 10.1038/s41467-020-19387-4 (PMC7599245; doi:10.1038/s41467-020-19387-4)
Supplement: Supplementary file 1 — Supplementary information [file 41467_2020_19387_MOESM1_ESM.pdf]

Supplementary Information

Regioselective Markovnikov hydrodifluoroalkylation of alkenes using difluoroenoxysilanes

Hu et al.

Supplementary Methods

General information

Reactions were monitored by thin layer chromatography using UV light to visualize the course of reaction. Purification of reaction products was carried out by flash column chromatography on silica gel. Chemical yields refer to pure isolated substances. The infrared (IR) spectra were obtained using a SHIMADZU IRTracer-100 Fourier transform infrared spectrometer. ^{1}H, ^{13}C, ^{19}F NMR spectra were obtained using a Bruker DPX-500 or Bruker DPX-400 spectrometer. Chemical shifts are reported in ppm from $CDCl_3$ or CD_3OD with the solvent resonance as the internal standard. The following abbreviations were used to designate chemical shift multiplicities: s = singlet, d = doublet, t = triplet, q = quartet, h = heptet, m = multiplet, br = broad.

All reactions were carried out under N_2 atmosphere except noted. Anhydrous $ClCH_2CH_2Cl$, CH_2Cl_2, CH_3CN were prepared by first distillation over P_2O_5 and then from CaH_2. Anhydrous EtOAc were prepared by first distillation over activated $CaSO_4$ and MS 5Å prior to use; anhydrous THF and toluene by distillation over sodium-benzophenone ketyl prior to use. The difluoroenoxysilanes **2a-j**[1,2], monofluorinated silyl enol ethers **2k**[2], **2l**[3], **2m**[4], and nonfluorinated silyl enol ethers **2n**[5] and **2o**[6] were prepared according to the literature reports. Unless otherwise noted, all the di-, and tri-substituted alkenes **1**, **4** and **9** were synthesized followed by the literature procedure[7]. Alkenes **1ad**[8], **1ae**[9], **4a**[10], **4e**[11], **7f**[12], **9c**[13], **9e**[14], **S1**[15], **S2**[16], **S5**[17], **S6**[18], and tetrasubstituted alkenes **6**[19] were prepared according to the indicated literature procedures. Alkenes **4f**, **7a-e**, **9a** and **9b** were purchased from TCI or Energy Chemical, and used as received. $Mg(ClO_4)_2{\cdot}6H_2O$ was purchased from Strem Chemicals, and used as received.

Selected condition optimization

The performance of several typical Brønsted acids in the coupling of 2-phenyl-1-butene **1a** with difluoroenoxysilane **2a** was examined by running the reaction in $ClCH_2CH_2Cl$ (Supplementary Table 1). All the following condition optimization reactions were run **in air**, except that mediated by HOTf. Two superacids were first examined. HOTf afforded the desired branch product **3a** in 36% yield (entry 1), and $HClO_4$, used as 70% aqueous solution, produced **3a** in a higher 55% yield, albeit with longer reaction time, possibly due to the presence of some water (entry 2). Ordinary strong acids exhibited much lower catalytic properties. Only H_2SO_4 gave **3a** in only 15% yield (entry 3), *p*-toluenesulfonic acid and CF_3CO_2H were unable to mediate this reaction (entries 4 and 5). Then we tried a range of metal triflates, and found $Fe(OTf)_3$, $Sc(OTf)_3$, $Ph_3PAuOTf$ and $Ga(OTf)_3$ could mediate this reaction (entries 6-9). In particular, $Ga(OTf)_3$ afforded **3a** in 75% yield (entry 9).

Supplementary Table 1. Initial screening of Brønsted acids and metal triflates

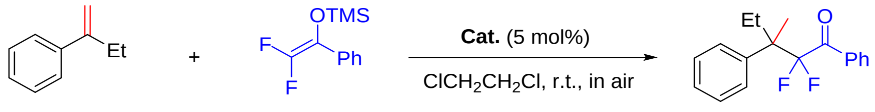

Entry[a]	**Cat.**	Time (h)	Yield[b]
1	HOTf	5	36
2	$HClO_4$ (aq. 70%)	24	55
3	H_2SO_4	24	15
4	$PTSA{\cdot}H_2O$	24	NR
5	CF_3CO_2H	24	NR
6	$Fe(OTf)_3$	3	40
7	$Sc(OTf)_3$	5	47
8	$Ph_3PAuOTf$	3	72
9	$Ga(OTf)_3$	3	75

[a]Conditions: **1a** (0.2 mmol), **2a** (1.5 equiv.), **Cat.** (5 mol%) in a concentration of 0.1 M at room temperature. [b]Yield of isolated product is reported.

However, the highly hydroscopic nature of metal triflates decreased the reproducibility of the reaction. Because the use of $HClO_4$ (aq. 70%) mediated the reaction well, we speculated the presence of some water might facilitate the reaction via scavenging the trimethyl group. In light of this, we considered using easy-to-handle metal perchlorate hydrates as the catalyst. We are pleased to find that

several metal perchlorate hydrates worked well, and some typical results were shown in Supplementary Table 2. Of those we tried (entries 1-5), $Zn(ClO_4)_2·6H_2O$ and $Mg(ClO_4)_2·6H_2O$ were identified to be best choice, delivering **3a** in 80% and 82% yield, respectively (entries 2 and 5). Then we examined the solvent effects by using $Mg(ClO_4)_2·6H_2O$ as the catalyst. Only halogenated solvents could mediate the reaction, $ClCH_2CH_2Cl$ afforded **3a** in better yield than CH_2Cl_2 (entry 5 *vs* 6). Other screened solvents, toluene, EtOAc, CH_3CN and THF, could not produce product **3a** at all (entries 7-10). Further increasing the loading of $Mg(ClO_4)_2·6H_2O$ to 10 mol%, the reaction time could be decreased to 7 h, and the yield of **3a** could be increased to 92% (entry 11).

Supplementary Table 2. Screening of metal perchlorate hydrates and solvents

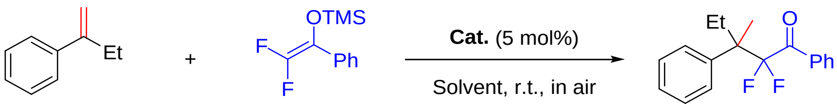

1a 2a 3a

Entry[a]	**Cat.**	Solvent	Time (h)	Yield[b]
1	$Cu(ClO_4)_2·6H_2O$	$ClCH_2CH_2Cl$	24	76
2	$Zn(ClO_4)_2·6H_2O$	$ClCH_2CH_2Cl$	24	80
3	$In(ClO_4)_3·8H_2O$	$ClCH_2CH_2Cl$	24	79
4	$Co(ClO_4)_2·6H_2O$	$ClCH_2CH_2Cl$	3	72
5	$Mg(ClO_4)_2·6H_2O$	$ClCH_2CH_2Cl$	12	82
6	$Mg(ClO_4)_2·6H_2O$	CH_2Cl_2	12	74
7	$Mg(ClO_4)_2·6H_2O$	Toluene	24	trace
8	$Mg(ClO_4)_2·6H_2O$	EtOAc	24	NR[c]
9	$Mg(ClO_4)_2·6H_2O$	CH_3CN	24	trace
10	$Mg(ClO_4)_2·6H_2O$	THF	24	NR[c]
**11[d]**	**$Mg(ClO_4)_2·6H_2O$**	**$ClCH_2CH_2Cl$**	**7**	**92**

[a]Conditions: **1a** (0.2 mmol), **2a** (1.5 equiv.), **Cat.** (5 mol%) in a concentration of 0.1 M at room temperature. [b]Yield of isolated product is reported. [c]No reaction. [d]10 mol% $Mg(ClO_4)_2·6H_2O$ was used.

The correlation of serial number of alkenes and their structure

Because the space limitation of the manuscript, we showed the structure of each alkene substrate and its serial number in this section.

Supplementary Table 3. The structure and serial number of 1,1-disubstituted olefins **1**.

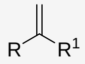

1

1a: R = Ph, R^1 = Et
1b: R = Ph, R^1 = Me
1c: R = Ph, R^1 = ^{n}Pr
1d: R = Ph, R^1 = *c*-hexyl
1e: R = 4-FC_6H_4, R^1 =4-$MeOC_6H_4$
1f: R = 4-FC_6H_4, R^1 = Et
1g: R = 4-ClC_6H_4, R^1 = Et
1h: R = 4-BrC_6H_4, R^1 = Et
1i: R = 4-$CF_3C_6H_4$, R^1 = Et
1j: R = 4-MeC_6H_4, R^1 = Et
1k: R = 4-$MeOC_6H_4$, R^1 = Et
1l: R = 3-$MeOC_6H_4$, R^1 = Et
1m: R = 2-$MeOC_6H_4$, R^1 = Et
1v: R = $PhCH_2CH_2$, R^1 = Me
1w: R = 4-$MeOC_6H_4CH_2CH_2$, R^1 = Me
1x: R = 2-naphthylCH_2CH_2, R^1 = Me
1y: R = 4-$MeC_6H_4CH_2$, R^1 = Me
1z: R = 4-$MeOC_6H_4CH_2$, R^1 = Me
1aa: R = *n*-propyl, R^1 = Me
1ab: R = *n*-hexyl, R^1 = Me
1ac: R = *c*-hexyl, R^1 = Me

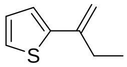

1n

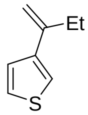

1o

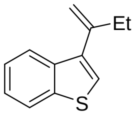

1p

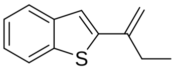

1q

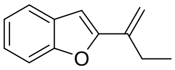

1r

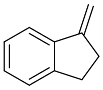

1s

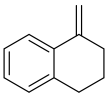

1t

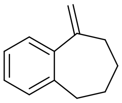

1u

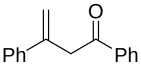

1ad

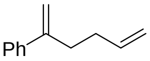

1ae

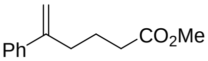

1af

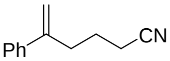

1ag

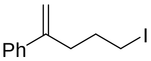

1ah

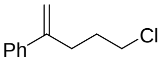

1ai

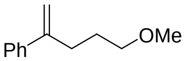

1aj

Supplementary Table 4. The structure and serial number of trisubstituted olefins **4**.

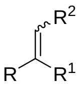

4a-d

4a: R = Ph, R^1 = Me, R^2 = Me
4b: R = Ph, R^1 = Me, R^2 = Bn
4c: R = Ph, R^1 = Me, R^2 = Ph
4d: R = 4-FC_6H_4, R^1 = Ph, R^2 = Me

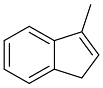

4e

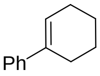

4f

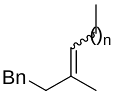

4g-j

4g: n = 0
4h: n = 1
4i: n = 2
4j: n = 3

Supplementary Table 5. The structure and serial number of tetrasubstituted olefins **6**.

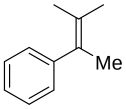

6a

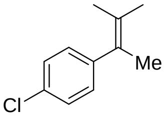

6b

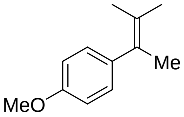

6c

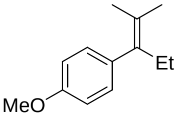

6d

Supplementary Table 6. The structure and serial number of monosubstituted olefins **7**.

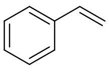

7a

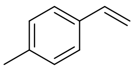

7b

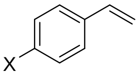

7c-e

7c: X = F
7d: X = Cl
7e: X = Br

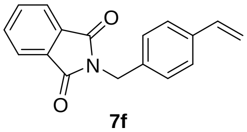

7f

Supplementary Table 7. The structure and serial number of disubstituted olefins **9**.

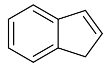

9a

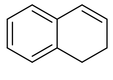

9b

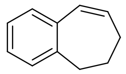

9c

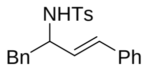

9d

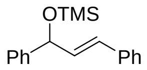

9e

Supplementary Table 8. The structure of natural product and drug derivatives **S1-S6**.

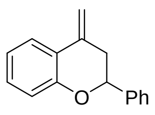

S1

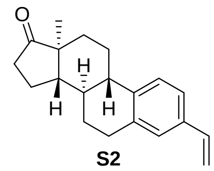

S2

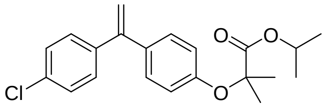

S3

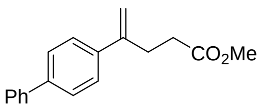

S4

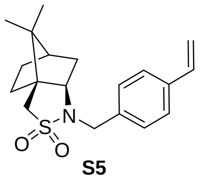

S5

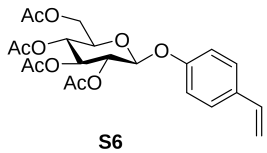

S6

The preparation of unknown alkenes

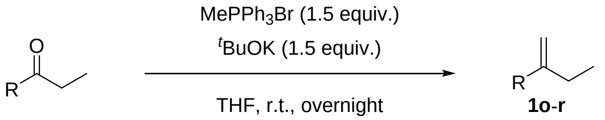

To an oven-dried flask were added $MePPh_3Br$ (1.5 equiv.) and anhydrous THF (0.5 M), followed by the addition of ^{t}BuOK (1.5 equiv.). After being stirred at room temperature for 1 h, the indicated ketones (1.0 equiv.) was added to the resulting mixture, and continue to stir at room temperature overnight. The reaction mixture was then filtered through a short pad of silica gel, and the residue was washed with hexanes. The filtrates were evaporated under reduced pressure to give the crude residue, which was purified by flash column chromatography with PE to afford the desired alkenes.

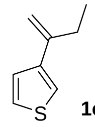
1o

3-(But-1-en-2-yl)thiophene (1o) was prepared from 1-(thiophen-3-yl)propan-1-one[20] in 53% yield as colorless oil; ^{1}H NMR (400 MHz, $CDCl_3$): δ 7.27-7.23 (m, 2H), 7.20-7.19 (m, 1H), 5.35-5.34 (m, 1H), 5.02-5.01 (m, 1H), 2.50-2.44 (m, 2H), 1.16 (t, *J* = 7.2 Hz, 3H); ^{13}C NMR (100 MHz, $CDCl_3$): δ 144.44, 142.83, 125.75, 125.27, 119.97, 109.64, 28.01, 12.87; IR (ATR): 2970, 1751, 1626, 1466, 1377, 1258, 983, 852, 785； GC-MS (EI): 138 (M^+, 100), 123 (62), 109 (53), 97 (22), 84 (41), 77 (11), 65 (18); HRMS (EI): Exact mass calcd for $C_8H_{10}S$ $[M]^+$: 138.0503, Found: 138.0505.

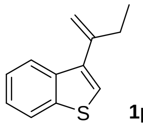
1p

3-(But-1-en-2-yl)benzo[*b*]thiophene (1p) was prepared from 1-(benzo[*b*]thiophen-3-yl)propan-1-one[21] in 59% yield as colorless oil; ^{1}H NMR (400 MHz, $CDCl_3$): δ 7.92-7.90 (m, 1H), 7.86-7.84 (m, 1H), 7.39-7.31 (m, 2H), 7.23 (s, 1H), 5.32-5.31 (m, 1H), 5.27-5.26 (m, 1H), 2.56-2.50 (m, 2H), 1.10 (t, *J* = 7.6 Hz, 3H); ^{13}C NMR (100 MHz, $CDCl_3$): δ 145.28, 140.47, 138.72, 138.14, 124.17, 124.03, 123.25, 122.75, 122.08, 113.21, 30.27, 12.82; IR (ATR): 2938, 1746, 1628, 1456, 1315, 1256, 1173, 1024, 899, 789； GC-MS (EI): 188 (M^+, 100), 173 (49), 159 (34), 147 (12), 134 (34), 115 (65), 89 (8), 77(4); HRMS (EI): Exact mass calcd for $C_{12}H_{12}S$ $[M]^+$: 188.0660, Found: 188.0659.

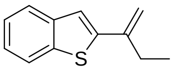
1q

2-(But-1-en-2-yl)benzo[*b*]thiophene (1q) was prepared from 1-(benzo[*b*]thiophen-2-yl)propan-1-one[21] in 65% yield as white solid. m.p. 37-39 °C; ^{1}H NMR (400 MHz, $CDCl_3$): δ 7.76-7.72 (m, 1H), 7.71-7.66 (m, 1H), 7.32-7.25 (m, 2H), 7.24-7.23 (m, 1H), 5.50 (s, 1H), 5.12 (s, 1H), 2.60-2.54 (m, 2H), 1.23 (t, *J* = 7.6 Hz, 3H); ^{13}C NMR (100 MHz, $CDCl_3$): δ

145.35, 143.59, 140.36, 138.87, 124.47, 124.23, 123.44, 122.01, 119.88, 112.28, 27.72, 13.00; IR (ATR): 2970, 1746, 1456, 1377, 1256, 1076, 984, 880, 741； GC-MS (EI): 188 (M^+, 100), 173 (51), 159 (28), 147 (13), 134 (30), 115 (44), 89 (6), 77 (3); HRMS (EI): Exact mass calcd for $C_{12}H_{12}S$ $[M]^+$: 188.0660, Found: 188.0662.

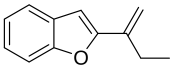

1r

2-(But-1-en-2-yl)benzofuran (1r) was prepared from 1-(benzofuran-2-yl)propan-1-one[22] in 58% yield as colorless oil; ^{1}H NMR (400 MHz, $CDCl_3$): δ 7.53-7.51 (m, 1H), 7.45-7.43 (m, 1H), 7.27-7.23 (m, 1H), 7.20-7.16 (m, 1H), 6.66 (s, 1H), 5.82 (s, 1H), 5.20-5.19 (m, 1H), 2.51-2.46 (m, 2H), 1.23 (t, J = 7.6 Hz, 3H); ^{13}C NMR (100 MHz, $CDCl_3$): δ 156.57, 154.62, 139.09, 128.98, 124.37, 122.60, 120.85, 111.42, 110.92, 102.28, 25.78, 13.02; IR (ATR): 2970, 1714, 1630, 1553, 1256, 1171, 1007, 941, 891, 798； GC-MS (EI): 172 (M^+, 100), 157 (55), 143 (27), 128 (20), 115 (30), 107 (5), 89 (7), 77 (6); HRMS (EI): Exact mass calcd for $C_{12}H_{12}O$ $[M]^+$: 172.0888, Found: 172.0889.

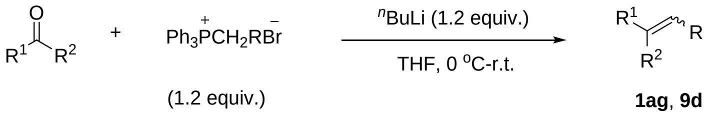

1ag, **9d**

To an oven-dried flask were added Ph_3PCH_2RBr (1.2 equiv.), anhydrous THF (0.5 M). The resulting mixture was cooled to 0 °C (under ice-water bath condition), and *n*-BuLi (1.2 equiv., 2.5 M in hexanes) was added slowly over 15 min at 0 °C. After being stirred at 0 °C for 0.5 h, to the resulting orange mixture was added a solution of indicated ketones (1.0 equiv.) in anhydrous THF (1.0 M) by dropwise over 15 min at the same temperature. After stirring at room temperature overnight, the reaction mixture was quenched with saturated aqueous NH_4Cl, and then extracted with pentane or diethyl ether for three times. The combined organic phases were washed with brine, dried over Na_2SO_4, and concentrated under reduced pressure. The crude residue was purified by flash column chromatography to afford the desired alkenes.

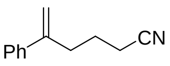

1ag

5-Phenylhex-5-enenitrile (1ag) was prepared from 5-*oxo*-5-phenylpentanenitrile[23] in 44% yield as light yellow oil. ^{1}H NMR (400 MHz, $CDCl_3$): δ 7.39-7.25 (m, 5H), 5.35 (s, 1H), 5.12 (s, 1H), 2.68 (t, J = 7.2 Hz, 2H), 2.31 (t, J = 7.2 Hz, 2H), 1.83-1.76 (m, 2H); ^{13}C NMR (100 MHz, $CDCl_3$): δ 146.10, 140.04, 128.47, 127.73, 126.04, 119,42, 114.00, 33.96, 23.67, 16.33; IR (ATR): 2974, 1628, 1599, 1574, 1495, 1051, 901,779; HRMS (ESI): Exact mass calcd for $C_{12}H_{13}NNa$ $[M+Na]^+$: 194.0940, Found: 194.0939.

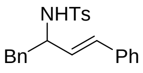

9d

(*E*)-*N*-(1,4-diphenylbut-3-en-2-yl)-4-methylbenzenesulfonamide (9d) was prepared from 4-methyl-*N*-(1-*oxo*-3-phenylpropan-2-yl) benzenesulfonamide[24] in 40 % yield as white solid. m.p. 110-112 °C; ^{1}H NMR (400 MHz, $CDCl_3$): δ 7.63 (d, *J* = 8.0 Hz, 2H), 7.25-7.16 (m, 6H), 7.12-7.06 (m, 6H), 6.21 (d, *J* = 15.6 Hz, 1H), 5.83 (dd, *J* = 16.0, 7.2 Hz, 1H), 4.99-4.96 (m, 1H), 4.21-4.14 (m, 1H), 2.85 (d, *J* = 6.8 Hz, 2H), 2.28 (s, 3H); ^{13}C NMR (100 MHz, $CDCl_3$): δ 143.09, 137.60, 136.21, 136.19, 131.47, 129.48, 129.42, 128.47, 128.30, 128.27, 127.57, 127.13, 126.71, 126.32, 56.90, 42.23, 21.31; IR (ATR): 1708, 1601, 1493, 1298, 1092, 806, 739; HRMS (ESI): Exact mass calcd for $C_{23}H_{23}NNaO_2S$ $[M+Na]^+$: 400.1342, Found: 400.1343.

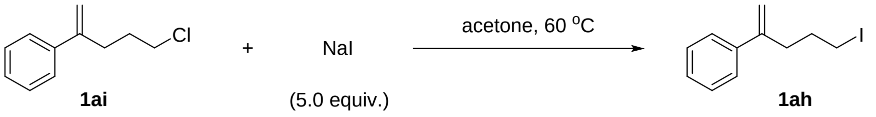

To a sealed tube were added **1ai** (1.807 g, 10 mmol) and acetone (20 mL), followed by the addition of NaI (7.495 g, 50.0 mmol). The resulting mixture was stirred at 60 °C until full conversion of **1ai** by TLC analysis (about 12 h). After cooling to the room temperature, the reaction mixture was filtered, and washed the residue with *n*-hexane. The obtained filtrate was concentrated under reduced pressure. The crude mixture was purified by flash column chromatography with PE to afford product **1ah** as colorless oil (2.204 g, 81% yield). ^{1}H NMR (400 MHz, $CDCl_3$): δ 7.41-7.39 (m, 2H), 7.35-7.31 (m, 2H), 7.29-7.27 (m, 1H), 5.32 (s, 1H), 5.13-5.12 (m, 1H), 3.18 (t, *J* = 6.8 Hz, 2H), 2.64 (t, *J* = 7.2 Hz, 2H), 1.98-1.91 (m, 2H); ^{13}C NMR (100 MHz, $CDCl_3$): δ 146.48, 140.52, 128.35, 127.53, 126.08, 113.43, 35.79, 31.59, 6.45; IR (ATR): 2887, 1626, 1599, 1494, 1215, 1167, 1051, 897, 777; GC-MS (EI): 272 (M^+, 9.4), 244 (3.3), 145 (58), 128 (6.7), 118 (100), 103 (20), 91 (31), 77 (14), 51 (8.7); HRMS (EI): Exact mass calcd for $C_{11}H_{13}I$ $[M]^+$: 272.0062, Found: 272.0058.

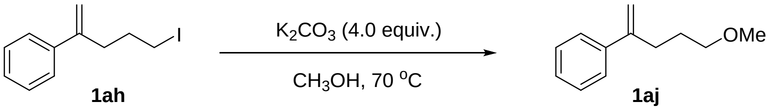

To a sealed tube were added **1ah** (0.544 g, 2.0 mmol) and MeOH (4.0 mL), followed by the addition of K_2CO_3 (1.106 g, 8.0 mmol). The resulting mixture was stirred at 70 °C until full conversion of **1ah** by TLC analysis (about 10 h). After cooling to the room temperature, water (10 mL) was added. The resulting solution was then extracted with CH_2Cl_2 (3 × 20 mL). The combined organic phases were washed with brine, dried over Na_2SO_4, concentrated under reduced pressure. The residue was purified by flash column chromatography (PE/CH_2Cl_2 = 10:1, v/v) to afford **1aj** as colorless oil (0.295 g, 84% yield). ^{1}H NMR (400 MHz, $CDCl_3$): δ 7.42-7.39 (m, 2H), 7.34-7.30 (m,

2H), 7.28-7.24 (m, 1H), 5.29 (d, *J* = 1.2 Hz, 1H), 5.08 (d, *J* = 1.6 Hz, 1H), 3.39 (t, *J* = 6.4 Hz, 2H), 3.32 (s, 3H), 2.58 (t, *J* = 7.6 Hz, 2H), 1.77-1.70 (m, 2H); ^{13}C NMR (100 MHz, $CDCl_3$): δ 148.01, 141.17, 128.25, 127.32, 126.11, 112.38, 72.12, 58.52, 31.78, 28.23; IR (ATR): 2978, 1626, 1599, 1574, 1495, 1447, 1385, 1119, 1053, 895, 777; GC-MS (EI): 176 (M^+, 1.0), 144 (12), 129 (11), 118 (100), 103 (16), 91 (13), 78 (12), 65 (3.0); HRMS (EI): Exact mass calcd for $C_{12}H_{16}O[M]^+$: 176.1201, Found: 176.1203.

The synthetic procedure for difluoroenoxysilanes

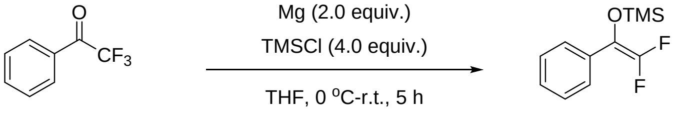

(10 mmol) **2a**

Except **2l**, all the difluoroenoxysilanes **2** employed in this reaction are known compounds, which could be readily prepared according to the indicated literatures shown in the general information. In the following, we took **2a** as an example to describe their synthetic procedure: To an oven-dried flask were added magnesium turning (0.480 g, 20.0 mmol), TMSCl (4.346 g, 40 mmol) and anhydrous THF (40 mL). The resulting mixture was cooled to 0 °C (under ice-water bath), and a solution of trifluoroacetophenone (1.741 g, 10 mmol) in anhydrous THF (10 mL) was added slowly over 15 min at 0 °C, and then slowly warmed to room temperature. After full conversion of ketone by TLC analysis (about 5 h), the reaction mixture was concentrated under reduced pressure to afford the residue, which was then washed with anhydrous hexane (60 mL). The white solid was removed by suction filtration, and the obtained filtrates were evaporated under vacuum to provide the crude product, which was further distilled under reduced pressure to afford the analytically pure product **2a** in 68% yield as colorless oil[1]. ^{1}H NMR (400 MHz, $CDCl_3$): δ 7.49-7.47 (m, 2H), 7.37-7.33 (m, 2H), 7.28-7.22 (m, 1H), 0.18 (s, 9H); ^{13}C NMR (100 MHz, $CDCl_3$): δ 154.86 (dd, *J* = 285.9, 284.0 Hz), 132.71 (dd, *J* = 7.1, 1.7 Hz), 128.26, 127.74 (t, *J* = 1.7 Hz), 125.92 (dd, *J* = 7.1, 3.7 Hz), 114.03 (dd, *J* = 34.4, 19.1 Hz), 0.01; ^{19}F NMR (376 MHz, $CDCl_3$): δ -99.93 (d, *J* = 68.0 Hz, 1F), -111.72 (d, *J* = 67.7 Hz, 1F). Difluoroenoxysilane **2a** can be stored at -20 ^{o}C (refrigerator) under nitrogen atmosphere for one month without obvious deterioration. And other difluoroenoxysilanes was prepared according the same procedure before use.

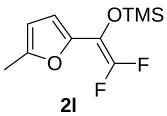

2l

((2,2-Difluoro-1-(5-methylfuran-2-yl)vinyl)oxy)trimethylsilane (2l) was prepared from 2,2,2-trifluoro-1-(5-methylfuran-2-yl)ethan-1-one[25] (10 mmol) according to the above procedure, 15% yield, light yellow oil. ^{1}H NMR (400 MHz, $CDCl_3$): δ 6.06 (d, *J* = 3.6 Hz, 1H), 5.84-5.81 (m, 1H), 2.13 (s, 3H), 0.04 (s, 9H); ^{13}C NMR (100 MHz, $CDCl_3$): δ 154.03 (dd, *J* = 287.1, 284.3 Hz), 152.27 (t, *J* = 3.2 Hz), 144.31 (dd, *J* = 8.6, 2.2 Hz), 109.09 (t, *J* = 6.1 Hz), 107.13, 13.52, -0.13; ^{19}F NMR (376 MHz, $CDCl_3$): δ -103.14 (d, *J* = 64.7 Hz, 1F), -110.89 (d, *J* = 65.0 Hz, 1F); GC-MS (EI): 232 (M^+, 63), 217 (20), 189 (15), 181 (69), 166 (20), 121 (14), 109 (20), 93 (14), 73(100); HRMS (EI): Exact mass calcd for $C_{10}H_{14}O_2SiF_2$ $[M]^+$: 232.0731, Found: 232.0729.

Substrate scope for the Markovnikov hydrodifluoroalkylation of simple alkenes

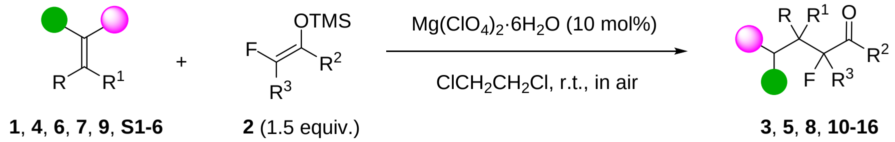

All the reactions were carried out **in air**. Unless otherwise noted, 10 mol% of $Mg(ClO_4)_2 \cdot 6H_2O$ and 1.5 equiv. of **2** were used. To a 5.0 mL vial were added $Mg(ClO_4)_2 \cdot 6H_2O$ (0.03 mmol, 10 mol%) and anhydrous $ClCH_2CH_2Cl$ (3.0 mL), followed by the sequential addition of alkenes (0.3 mmol, 1.0 equiv.) and fluorinated silyl enol ethers **2** (0.45 mmol, 1.5 equiv.). The resulting mixture was stirred at room temperature until full consumption of alkenes by TLC analysis. The reaction mixture was then concentrated under reduced pressure. The crude residue was purified by flash column chromatography using the indicated eluent to afford the desired products.

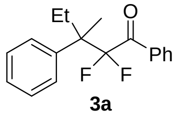

3a

Column chromatography (PE/CH_2Cl_2 = 1:0 to 20:1, v/v) afforded product **3a** as colorless oil (79.6 mg, 92% yield). ^{1}H NMR (400 MHz, $CDCl_3$): δ 7.55 (d, J = 7.6 Hz, 2H), 7.45-7.41 (m, 1H), 7.35 (d, J = 7.6 Hz, 2H), 7.26-7.15 (m, 5H), 2.46-2.37 (m, 1H), 1.99-1.90 (m, 1H), 1.60 (s, 3H), 0.75 (t, J = 7.2 Hz, 3H); ^{13}C NMR (100 MHz, $CDCl_3$): δ 191.28 (t, J = 30.3 Hz), 138.10 (t, J = 2.4 Hz), 134.31 (t, J = 1.8 Hz), 133.16, 129.73 (t, J = 4.0 Hz), 128.75, 128.05, 127.94, 127.19, 120.88 (t, J = 260.1 Hz), 48.28 (t, J = 20.3 Hz), 26.19 (t, J = 3.8 Hz), 18.09 (t, J = 4.3 Hz), 7.62; ^{19}F NMR (376 MHz, $CDCl_3$): δ -104.95 (d, J = 257.9 Hz, 1F), -106.29 (d, J = 257.9 Hz, 1F); IR (ATR): 2972, 1684, 1597, 1449, 1385, 1273, 1182, 1098, 905, 866, 770, 654; HRMS (ESI): Exact mass calcd for $C_{18}H_{18}F_2NaO$ $[M+Na]^+$: 311.1218, Found: 311.1221.

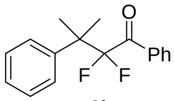

3b

The reaction used 3 mol% of $Mg(ClO_4)_2 \cdot 6H_2O$ and 3.0 equiv. of **2a**. Column chromatography (PE/CH_2Cl_2 = 1:0 to 20:1, v/v) afforded product **3b** as colorless oil (51.2 mg, 62% yield). ^{1}H NMR (400 MHz, $CDCl_3$): δ 7.60 (d, J = 7.6 Hz, 2H), 7.44-7.42 (m, 3H), 7.27-7.18 (m, 5H), 1.64 (s, 6H); ^{13}C NMR (100 MHz, $CDCl_3$): δ 191.06 (t, J = 30.3 Hz), 140.90 (t, J = 2.3 Hz), 134.19 (t, J = 1.9 Hz), 133.30, 129.82 (t, J = 4.1 Hz), 128.06, 128.04, 128.02, 127.31, 120.62 (t, J = 259.5 Hz), 44.49 (t, J = 21.2 Hz), 22.88 (t, J = 4.3 Hz); ^{19}F NMR (376 MHz, $CDCl_3$): δ -105.43 (s, 2F); IR (ATR): 2976, 1749, 1688, 1449, 1393, 1277, 1134, 1051, 881, 756; HRMS (ESI): Exact mass calcd for $C_{17}H_{16}F_2NaO$ $[M+Na]^+$: 297.1061, Found: 297.1059.

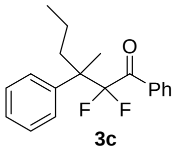

3c

Column chromatography (PE/CH_2Cl_2 = 1:0 to 20:1, v/v) afforded product **3c** as colorless oil (75.6 mg, 83% yield). ^1H NMR (400 MHz, $CDCl_3$): δ 7.54 (d, *J* = 8.0 Hz, 2H), 7.43-7.39 (m, 1H), 7.35 (d, *J* = 8.0 Hz, 2H), 7.23-7.13 (m, 5H), 2.29 (td, *J* = 13.2, 3.6 Hz, 1H), 1.94-1.86 (m, 1H), 1.61 (s, 3H), 1.30-1.19 (m, 1H), 1.06-0.94 (m, 1H), 0.90 (t, *J* = 6.8 Hz, 3H); ^{13}C NMR (100 MHz, $CDCl_3$): δ 191.31 (t, *J* = 30.2 Hz), 138.63 (t, *J* = 2.6 Hz), 134.33 (t, *J* = 1.8 Hz), 133.17, 129.76 (t, *J* = 4.1 Hz), 128.60, 128.07, 127.95, 127.20, 120.77 (t, *J* = 260.2 Hz), 48.09 (t, *J* = 20.4 Hz), 35.81 (t, *J* = 3.2 Hz), 18.74 (t, *J* = 4.4 Hz), 16.55, 14.63; ^{19}F NMR (376 MHz, $CDCl_3$): δ -104.88 (d, *J* = 258.3 Hz, 1F), -106.20 (d, *J* = 258.7 Hz, 1F); IR (ATR): 2967, 1695, 1595, 1449, 1387, 1265, 1132, 941, 893, 772, 696; HRMS (ESI): Exact mass calcd for $C_{19}H_{20}F_2NaO$ $[M+Na]^+$: 325.1374, Found: 325.1376.

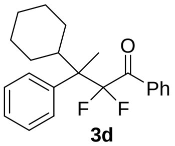

3d

Column chromatography (PE/CH_2Cl_2 = 1:0 to 20:1, v/v) afforded product **3d** as colorless oil (100.1 mg, 97% yield). ^1H NMR (400 MHz, $CDCl_3$): δ 7.44 (d, *J* = 7.6 Hz, 2H), 7.40-7.36 (m, 1H), 7.33-7.30 (m, 2H), 7.22-7.18 (m, 2H), 7.14-7.05 (m, 3H), 2.42-2.36 (m, 1H), 2.30-2.27 (m, 1H), 1.87-1.83 (m, 1H), 1.67-1.60 (m, 5H), 1.42-1.33 (m, 2H), 1.19-1.07 (m, 2H), 1.02-0.99 (m, 1H), 0.95-0.86 (m, 1H); ^{13}C NMR (100 MHz, $CDCl_3$): δ 191.85 (dd, *J* = 32.5, 29.3 Hz), 140.17 (d, *J* = 6.0 Hz), 135.03 (d, *J* = 2.8 Hz), 132.63, 129.23 (dd, *J* = 5.4, 3.0 Hz), 128.11, 128.00, 127.75, 127.00, 121.77 (dd, *J* = 265.0, 263.0 Hz), 51.35 (t, *J* = 19.5 Hz), 43.38 (d, *J* = 5.8 Hz), 28.89 (dd, *J* = 4.7, 1.9 Hz), 27.49, 27.06, 26.95, 26.59, 13.49 (t, *J* = 5.3 Hz); ^{19}F NMR (376 MHz, $CDCl_3$): δ -94.16 (d, *J* = 260.9 Hz, 1F), -106.43 (d, *J* = 260.6 Hz, 1F); IR (ATR): 2932, 1662, 1578, 1476, 1242, 1165, 868, 858, 719, 694; HRMS (ESI): Exact mass calcd for $C_{22}H_{24}F_2NaO$ $[M+Na]^+$: 365.1687, Found: 365.1692.

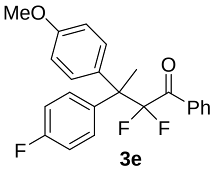

3e

Column chromatography (PE/CH_2Cl_2 = 1:2, v/v) afforded product **3e** as colorless oil (93.3 mg, 81% yield). ^1H NMR (400 MHz, $CDCl_3$): δ 7.68 (d, *J* = 8.0 Hz, 2H), 7.48 (t, *J* = 7.6 Hz, 1H), 7.32-7.25 (m, 4H), 7.19 (d, *J* = 8.4 Hz, 2H), 6.95-6.90 (m, 2H), 6.77-6.74 (m, 2H), 3.75 (s, 3H), 2.00 (s, 3H); ^{13}C NMR (100 MHz, $CDCl_3$): δ 190.28 (t, *J* = 31.0 Hz), 161.51 (d, *J* = 245.0 Hz), 158.44, 138.94 (t, *J* = 2.3 Hz), 134.16 (d, *J* = 3.0 Hz), 134.05 (t, *J* = 2.3 Hz), 133.32, 130.76 (d, *J* = 7.9 Hz), 130.32, 129.70 (t, *J* = 3.9 Hz), 128.10, 120.46 (t, *J* = 262.7 Hz), 114.63 (d, *J* = 20.9 Hz), 113.29, 55.17 (d, *J* = 3.9 Hz), 53.23 (t, *J* = 20.3 Hz), 25.16; ^{19}F NMR (376 MHz, $CDCl_3$): δ -98.33 (d, *J* = 275.6 Hz, 1F), -99.65 (d, *J* = 274.9 Hz, 1F),

-115.99 (s, 1F); IR (ATR): 3065, 1670, 1611, 1466, 1449, 1346, 1238, 1105, 1082, 899, 756; HRMS (ESI): Exact mass calcd for $C_{23}H_{19}F_3NaO_2$ $[M+Na]^+$: 407.1229, Found: 407.1236.

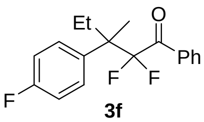

3f

Column chromatography (PE/CH_2Cl_2 = 1:0 to 20:1, v/v) afforded product **3f** as colorless oil (76.1 mg, 83% yield). ^1H NMR (400 MHz, $CDCl_3$): δ 7.63 (d, *J* = 7.6 Hz, 2H), 7.47 (t, *J* = 7.2 Hz, 1H), 7.33-7.29 (m, 4H), 6.91 (t, *J* = 8.8 Hz, 2H), 2.40-2.31 (m, 1H), 1.98-1.89 (m, 1H), 1.58 (s, 3H), 0.73 (t, *J* = 7.6 Hz, 3H); ^{13}C NMR (100 MHz, $CDCl_3$): δ 190.93 (t, *J* = 30.2 Hz), 161.88 (d, *J* = 245.3 Hz), 134.22 (t, *J* = 1.9 Hz), 133.98 (dd, *J* = 4.8, 3.2 Hz), 133.40, 130.49 (d, *J* = 7.9 Hz), 129.75 (t, *J* = 4.1 Hz), 128.11, 120.72 (td, *J* = 260.4, 0.6 Hz), 114.82 (d, *J* = 20.8 Hz), 47.90 (t, *J* = 20.4 Hz), 26.40 (t, *J* = 3.7 Hz), 18.34 (t, *J* = 4.3 Hz), 7.54; ^{19}F NMR (376 MHz, $CDCl_3$): δ -105.14 (d, *J* = 260.9 Hz, 1F), -106.42 (d, *J* = 261.3 Hz, 1F), -115.66 (s, 1F); IR (ATR): 2978, 1726, 1609, 1476, 1383, 1285, 1134, 1016, 912, 872, 721, 629; HRMS (ESI): Exact mass calcd for $C_{18}H_{17}F_3NaO$ $[M+Na]^+$: 329.1124, Found: 329.1125.

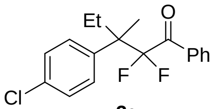

3g

Column chromatography (PE/CH_2Cl_2 = 1:0 to 20:1, v/v) afforded product **3g** as colorless oil (82.1 mg, 85% yield). ^1H NMR (400 MHz, $CDCl_3$): δ 7.66 (d, *J* = 8.0 Hz, 2H), 7.48 (t, *J* = 7.2 Hz, 1H), 7.31-7.24 (m, 4H), 7.19 (d, *J* = 8.8 Hz, 2H), 2.38-2.29 (m, 1H), 1.97-1.88 (m, 1H), 1.56 (s, 3H), 0.72 (t, *J* = 7.6 Hz, 3H); ^{13}C NMR (100 MHz, $CDCl_3$): δ 190.60 (t, *J* = 30.3 Hz), 136.91 (t, *J* = 2.4 Hz), 134.11 (t, *J* = 2.1 Hz), 133.46, 133.22, 130.18, 129.77 (t, *J* = 4.1 Hz), 128.16, 128.11, 120.58 (t, *J* = 260.2 Hz), 48.02 (t, *J* = 20.3 Hz), 26.36 (t, *J* = 3.8 Hz), 18.19 (t, *J* = 4.3 Hz), 7.55; ^{19}F NMR (376 MHz, $CDCl_3$): δ -104.88 (d, *J* = 264.0 Hz, 1F), -105.99 (d, *J* = 264.0 Hz, 1F); IR (ATR): 2976, 1611, 1578, 1516, 1477, 1304, 1204, 1173, 1051, 907, 837, 745, 687; GC-MS (EI): 324 $[M(^{37}Cl)^+$, 1.2], 322 $[M(^{35}Cl)^+$, 3.4], 167 (45), 125 (28), 105 (100), 77 (31), 55 (5.4); HRMS (EI): Exact mass calcd for $C_{18}H_{17}OF_2{}^{35}Cl$ $[M]^+$: 322.0936, Found: 322.0933.

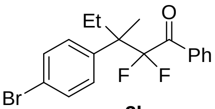

3h

The reaction was carried out at 50 ^oC. Column chromatography (PE/CH_2Cl_2 = 1:0 to 20:1, v/v) afforded product **3h** as colorless oil (96.9 mg, 88% yield). ^1H NMR (400 MHz, $CDCl_3$): δ 7.67 (d, *J* = 8.0 Hz, 2H), 7.50-7.46 (m, 1H), 7.36-7.34 (m, 2H), 7.31-7.27 (m, 2H), 7.24-7.21 (m, 2H), 2.37-2.28 (m, 1H), 1.97-1.88 (m, 1H), 1.56 (s, 3H), 0.72 (t, *J* = 7.2 Hz, 3H); ^{13}C NMR (100 MHz, $CDCl_3$): δ 190.55 (t, *J* = 30.4 Hz), 137.49 (t, *J* = 2.5 Hz), 134.12 (t, *J* = 2.1 Hz), 133.46, 131.08, 130.54, 129.77 (t, *J* = 4.1 Hz), 128.17, 121.48, 120.51 (t, *J* =

260.2 Hz), 48.11 (t, J = 20.4 Hz), 26.34 (t, J = 3.7 Hz), 18.15 (t, J = 4.2 Hz), 7.56; ^{19}F NMR (376 MHz, $CDCl_3$): δ -104.84 (d, J = 264.7 Hz, 1F), -105.93 (d, J = 264.3 Hz, 1F); IR (ATR): 2976, 1612, 1514, 1466, 1279, 1136, 1080, 907, 800, 760, 687; HRMS (ESI): Exact mass calcd for $C_{18}H_{17}BrF_2NaO$ $[M+Na]^+$: 389.0323, Found: 389.0332.

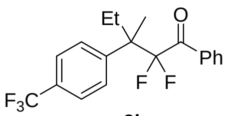

3i

Column chromatography (PE/CH_2Cl_2 = 1:0 to 20:1, v/v) afforded product **3i** as colorless oil (30.8 mg, 29% yield). ^{1}H NMR (400 MHz, $CDCl_3$): δ 7.68-7.65 (m, 2H), 7.50-7.46 (m, 5H), 7.31-7.26 (m, 2H), 2.43-2.34 (m, 1H), 2.03-1.94 (m, 1H), 1.62 (s, 3H), 0.73 (t, J = 7.6 Hz, 3H); ^{13}C NMR (125 MHz, $CDCl_3$): δ 190.28 (t, J = 30.3 Hz), 142.74, 134.08 (t, J = 2.0 Hz), 133.60, 129.75 (t, J = 4.4 Hz), 129.18, 128.22, 124.85 (q, J = 3.9 Hz), 123.99 (q, J = 270.1 Hz), 120.53 (t, J = 260.2 Hz), 48.54 (t, J = 20.2 Hz), 26.50 (t, J = 3.6 Hz), 18.21 (t, J = 4.2 Hz), 7.60; ^{19}F NMR (376 MHz, $CDCl_3$): δ -62.75 (s, 3F), -104.77 (d, J = 267.0 Hz, 1F), -105.82 (d, J = 267.3 Hz, 1F); IR (ATR): 2978, 1612, 1477, 1468, 1449, 1283, 1171, 1016, 845, 745； HRMS (ESI): Exact mass calcd for $C_{19}H_{17}F_5NaO$ $[M+Na]^+$: 379.1092, Found: 379.1101.

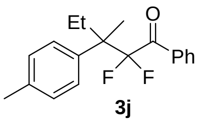

3j

Column chromatography (PE/CH_2Cl_2 = 1:0 to 20:1, v/v) afforded product **3j** as colorless oil (69.0 mg, 76% yield). ^{1}H NMR (400 MHz, $CDCl_3$): δ 7.56 (d, J = 7.6 Hz, 2H), 7.42 (t, J = 7.6 Hz, 1H), 7.25-7.21 (m, 4H), 7.02 (d, J = 8.4 Hz, 2H), 2.43-2.34 (m, 1H), 2.25 (s, 3H), 1.96-1.87 (m, 1H), 1.57 (s, 3H), 0.74 (t, J = 7.6 Hz, 3H); ^{13}C NMR (100 MHz, $CDCl_3$): δ 191.43 (t, J = 30.3 Hz), 136.85, 135.00 (t, J = 2.4 Hz), 134.40, 133.07, 129.80 (t, J = 4.1 Hz), 128.77, 128.65, 127.91, 120.97 (t, J = 259.9 Hz), 47.97 (t, J = 20.3 Hz), 26.15 (t, J = 4.0 Hz), 20.80, 18.15 (t, J = 4.4 Hz), 7.65; ^{19}F NMR (376 MHz, $CDCl_3$): δ -105.00 (d, J = 257.6 Hz, 1F), -106.30 (d, J = 257.9 Hz, 1F); IR (ATR): 2976, 1691, 1670, 1516, 1477, 1341, 1242, 1138, 1038, 907, 880, 752, 687; GC-MS (EI): 302 (M^+, 3.9), 147 (88), 119 (4.0), 106 (8.0), 105 (100), 91 (4.7), 77 (23); HRMS (EI): Exact mass calcd for $C_{19}H_{20}OF_2$ $[M]^+$: 302.1482, Found: 302.1478.

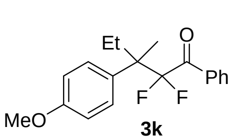

3k

Column chromatography (PE/CH_2Cl_2 = 10:1, v/v) afforded product **3k** as colorless oil (93.3 mg, 98% yield). ^{1}H NMR (400 MHz, $CDCl_3$): δ 7.57 (d, J = 7.6 Hz, 2H), 7.44-7.40 (m, 1H), 7.26-7.22 (m, 4H), 6.76-6.72 (m, 2H), 3.72 (s, 3H), 2.40-2.31 (m, 1H), 1.96-1.86 (m, 1H), 1.56 (s, 3H), 0.74 (t, J = 7.6 Hz, 3H); ^{13}C NMR (100 MHz, $CDCl_3$): δ 191.49 (t, J = 30.2 Hz), 158.62, 134.44 (t, J = 2.0 Hz), 133.09, 129.96, 129.90, 129.77 (t, J = 4.1 Hz), 127.95, 120.98 (t, J = 259.9 Hz), 113.40, 55.09 (d, J = 3.6 Hz), 47.67 (t, J = 20.4 Hz),

26.19 (t, J = 3.4 Hz), 18.23 (t, J = 4.2 Hz), 7.59; ^{19}F NMR (376 MHz, $CDCl_3$): δ -105.13 (d, J = 256.4 Hz, 1F), -106.57 (d, J = 256.8 Hz, 1F); IR (ATR): 2976, 1653, 1514, 1476, 1275, 1136, 907, 872, 837, 754; HRMS (ESI): Exact mass calcd for $C_{19}H_{20}F_2NaO_2$ $[M+Na]^+$: 341.1324, Found: 341.1325.

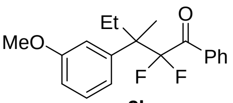

3l

Column chromatography (PE/CH_2Cl_2 = 10:1 to 6:1, v/v) afforded product **3l** as colorless oil (77.5 mg, 81% yield). ^{1}H NMR (400 MHz, $CDCl_3$): δ 7.58 (d, J = 8.0 Hz, 2H), 7.44-7.40 (m, 1H), 7.25-7.21 (m, 2H), 7.13 (t, J = 8.0 Hz, 1H), 6.94 (d, J = 8.0 Hz, 1H), 6.88 (t, J = 2.0 Hz, 1H), 6.70 (dd, J = 8.4, 2.8 Hz, 1H), 3.68 (s, 3H), 2.41-2.32 (m, 1H), 1.97-1.88 (m, 1H), 1.57 (s, 3H), 0.75 (t, J = 7.6 Hz, 3H); ^{13}C NMR (100 MHz, $CDCl_3$): δ 191.25 (t, J = 30.0 Hz), 159.42, 139.86 (t, J = 2.5 Hz), 134.49, 133.13, 129.71 (t, J = 4.1 Hz), 128.97, 127.94, 121.28, 120.92 (t, J = 260.3 Hz), 115.50, 112.09, 55.10 (d, J = 4.6 Hz), 48.43 (t, J = 20.4 Hz), 26.35 (t, J = 4.5 Hz), 18.08 (t, J = 3.9 Hz), 7.68; ^{19}F NMR (376 MHz, $CDCl_3$): δ -104.80 (d, J = 257.9 Hz, 1F), -106.26 (d, J = 257.9 Hz, 1F); IR (ATR): 2976, 1692, 1599, 1582, 1491, 1449, 1385, 1256, 1177, 1099, 912, 777; HRMS (ESI): Exact mass calcd for $C_{19}H_{20}F_2NaO_2$ $[M+Na]^+$: 341.1324, Found: 341.1317.

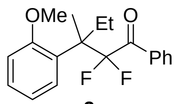

3m

Column chromatography (PE/CH_2Cl_2 = 10:1 to 6:1, v/v) afforded product **3m** as white solid (77.4 mg, 81% yield). m.p. 46-48 ^oC; ^{1}H NMR (400 MHz, $CDCl_3$): δ 7.47-7.36 (m, 4H), 7.21-7.14 (m, 3H), 7.00-6.95 (m, 1H), 6.39 (d, J = 8.0 Hz, 1H), 3.05 (s, 3H), 2.89-2.80 (m, 1H), 1.78-1.73 (m, 1H), 1.70 (s, 3H), 0.67 (t, J = 7.6 Hz, 3H); ^{13}C NMR (100 MHz, $CDCl_3$): δ 189.80 (t, J = 28.8 Hz), 158.31, 134.02, 132.86, 131.22, 129.71 (t, J = 4.1 Hz), 129.19, 127.60, 125.85 (t, J = 2.8 Hz), 121.13 (t, J = 258.5 Hz), 120.61, 110.73, 53.22 (d, J = 1.2 Hz), 49.72 (t, J = 21.6 Hz), 26.08 (t, J = 3.2 Hz), 19.70 (t, J = 5.4 Hz), 8.26; ^{19}F NMR (376 MHz, $CDCl_3$): δ -102.39 (d, J = 248.2 Hz, 1F), -103.12 (d, J = 248.2 Hz, 1F); IR (ATR): 2974, 1692, 1597, 1493, 1449, 1381, 1246, 1173, 1088, 907, 881, 748; HRMS (ESI): Exact mass calcd for $C_{19}H_{20}F_2NaO_2$ $[M+Na]^+$: 341.1324, Found: 341.1323.

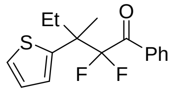

3n

The reaction used 5 mol% of $Mg(ClO_4)_2\cdot 6H_2O$ and 3.0 equiv. of **2a**. Column chromatography (PE/CH_2Cl_2 = 1:0 to 20:1, v/v) afforded product **3n** as colorless oil (48.0 mg, 54% yield). ^{1}H NMR (400 MHz, $CDCl_3$): δ 7.58 (d, J = 7.6 Hz, 2H), 7.41-7.36 (m, 1H), 7.23-7.17 (m, 2H), 7.07 (dd, J = 4.8, 0.8 Hz, 1H), 6.81 (d, J = 3.2 Hz, 2H), 2.18-2.09 (m, 1H), 2.00-1.91 (m, 1H), 1.54 (s, 3H), 0.77 (t, J = 7.6 Hz, 3H); ^{13}C NMR (125 MHz,

CDCl$_3$): δ 191.11 (t, *J* = 30.0 Hz), 143.39 (t, *J* = 2.9 Hz), 134.28 (t, *J* = 2.0 Hz), 133.35, 129.61 (t, *J* = 4.0 Hz), 128.08, 127.25, 126.71, 125.22, 120.02 (t, *J* = 260.8 Hz), 47.55, 27.93 (dd, *J* = 5.0, 3.0 Hz), 18.94 (t, *J* = 4.0 Hz), 7.87; ^{19}F NMR (376 MHz, CDCl$_3$): δ -105.06 (d, *J* = 258.3 Hz, 1F), -106.49 (d, *J* = 257.9 Hz, 1F); IR (ATR): 2970, 1597, 1578, 1464, 1449, 1306, 1242, 1132, 1059, 935, 851, 662; GC-MS (EI): 294 (M$^+$, 4.5), 140 (9.7), 139 (100), 105 (38), 97 (20), 77 (24), 55 (8); HRMS (EI): Exact mass calcd for $C_{16}H_{16}OSF_2$ [M]$^+$: 294.0890, Found: 294.0891.

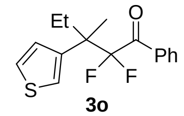

3o

The reaction used 1 mol% of $Mg(ClO_4)_2{\cdot}6H_2O$ and 3.0 equiv. of **2a**. Column chromatography (PE/CH_2Cl_2 = 20:1, v/v) afforded product **3o** as colorless oil (18.3 mg, 21% yield). ^{1}H NMR (400 MHz, CDCl$_3$): δ 7.61-7.59 (m, 2H), 7.48-7.44 (m, 1H), 7.30-7.26 (m, 2H), 7.18-7.16 (m, 1H), 7.04-7.02 (m, 2H), 2.29-2.20 (m, 1H), 1.99-1.90 (m, 1H), 1.55 (s, 3H), 0.78 (t, *J* = 7.2 Hz, 3H); ^{13}C NMR (125 MHz, CDCl$_3$): δ 191.36 (t, *J* = 29.8 Hz), 140.38 (t, *J* = 3.0 Hz), 134.37, 133.22, 129.64 (t, *J* = 4.0 Hz), 128.09, 128.07, 125.29, 124.08, 120.64 (t, *J* = 259.8 Hz), 46.88 (t, *J* = 21.2 Hz), 26.83 (dd, *J* = 4.9, 3.1 Hz), 18.34 (t, *J* = 4.2 Hz), 7.83; ^{19}F NMR (376 MHz, CDCl$_3$): δ -105.49 (d, *J* = 257.2 Hz, 1F), -106.92 (d, *J* = 257.2 Hz, 1F); IR (ATR): 2976, 1692, 1597, 1464, 1385, 1273, 1124, 1011, 922, 880, 737； HRMS (ESI): Exact mass calcd for $C_{16}H_{16}F_2NaOS$ [M+Na]$^+$: 317.0782, Found: 317.0765.

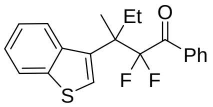

3p

Column chromatography (PE/CH_2Cl_2 = 20:1 to 10:1, v/v) afforded product **3p** as colorless oil (44.2 mg, 43% yield). ^{1}H NMR (400 MHz, CDCl$_3$): δ 8.20-8.18 (m, 1H), 7.73-7.71 (m, 1H), 7.48 (d, *J* = 7.6 Hz, 2H), 7.37-7.26 (m, 3H), 7.14 (s, 1H), 7.08-7.04 (m, 2H), 2.87-2.78 (m, 1H), 2.02-1.93 (m, 1H), 1.71 (s, 3H), 0.75 (t, *J* = 7.6 Hz, 3H); ^{13}C NMR (100 MHz, CDCl$_3$): δ 191.00 (dd, *J* = 30.1, 28.3 Hz), 140.93, 138.42, 134.19 (t, *J* = 4.8 Hz), 133.09, 129.25 (dd, *J* = 5.1, 3.4 Hz), 127.79, 127.53, 124.92 (dd, *J* = 6.0, 2.9 Hz), 124.03, 123.74, 122.77, 121.56 (t, *J* = 261.0 Hz), 48.70 (t, *J* = 21.9 Hz), 25.98 (t, *J* = 3.1 Hz), 20.65 (t, *J* = 4.2 Hz), 7.92; ^{19}F NMR (376 MHz, CDCl$_3$): δ -103.56 (d, *J* = 253.0 Hz, 1F), -105.08 (d, *J* = 253.4 Hz, 1F); IR (ATR): 2972, 1786, 1628, 1404, 1168, 966, 854, 733, 633； HRMS (ESI): Exact mass calcd for $C_{20}H_{18}F_2NaOS$ [M+Na]$^+$: 367.0939, Found: 367.0917.

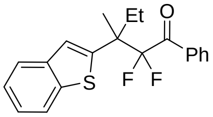

3q

The reaction used 20 mol% of $Mg(ClO_4)_2{\cdot}6H_2O$ and 3.0 equiv. of **2a**. Column chromatography (PE/CH_2Cl_2 = 1:0 to 20:1, v/v) afforded product **3q** as colorless oil (20.5 mg, 20% yield). ^1H NMR (400 MHz, $CDCl_3$): δ 7.78-7.75 (m, 2H), 7.72-7.70 (m, 1H), 7.63-7.61 (m, 1H), 7.41-7.37 (m, 1H), 7.28-7.24 (m, 2H), 7.23-7.19 (m, 2H), 7.13 (s, 1H), 2.31-2.22 (m, 1H), 2.11-2.02 (m, 1H), 1.68 (s, 3H), 0.88 (t, J = 7.2 Hz, 3H); ^{13}C NMR (125 MHz, $CDCl_3$): δ 190.38 (t, J = 30.4 Hz), 144.60, 139.58, 139.37, 134.17 (t, J = 2.2 Hz), 133.43, 129.66 (t, J = 4.0 Hz), 128.06, 124.15, 124.11, 124.05, 123.31, 121.84, 120.03 (t, J = 261.0 Hz), 48.23 (t, J = 21.4 Hz), 28.13 (t, J = 3.4 Hz), 18.86 (t, J = 4.0 Hz), 7.93; ^{19}F NMR (376 MHz, $CDCl_3$): δ -104.56 (d, J = 266.2 Hz, 1F), -105.56 (d, J = 265.8 Hz, 1F); IR (ATR):1751, 1578, 1435, 1219, 1128, 937, 827, 783, 611; HRMS (ESI): Exact mass calcd for $C_{20}H_{18}F_2NaOS$ $[M+Na]^+$: 367.0939, Found: 367.0952.

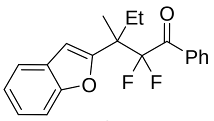

3r

The reaction used 20 mol% of $Mg(ClO_4)_2{\cdot}6H_2O$ and 3.0 equiv. of **2a**. Column chromatography (PE/CH_2Cl_2 = 20:1, v/v) afforded product **3r** as colorless oil (34.2 mg, 35% yield). ^1H NMR (400 MHz, $CDCl_3$): δ 7.69-7.67 (m, 2H), 7.42-7.38 (m, 1H), 7.32-7.27 (m, 1H), 7.20-7.08 (m, 5H), 6.57 (d, J = 0.8 Hz, 1H), 2.43-2.34 (m, 1H), 1.99-1.90 (m, 1H), 1.59 (s, 3H), 0.81 (t, J = 7.6 Hz, 3H); ^{13}C NMR (125 MHz, $CDCl_3$): δ 190.40 (t, J = 29.0 Hz), 155.72 (t, J = 2.9 Hz), 154.40, 133.70 (t, J = 1.8 Hz), 133.25, 129.45 (t, J = 4.2 Hz), 127.95, 127.92, 124.00, 122.60, 120.62, 119.98 (t, J = 260.9 Hz), 110.97, 106.69, 47.36 (t, J = 22.2 Hz), 25.25 (t, J = 3.2 Hz), 16.11 (t, J = 4.1 Hz), 8.06; ^{19}F NMR (376 MHz, $CDCl_3$): δ -105.65 (d, J = 257.2 Hz, 1F), -106.48 (d, J = 257.2 Hz, 1F); IR (ATR): 2997, 1784, 1597, 1404, 1348, 1126, 1028, 941, 785; HRMS (ESI): Exact mass calcd for $C_{20}H_{18}F_2NaO_2$ $[M+Na]^+$: 351.1167, Found: 351.1159.

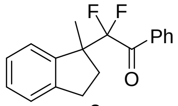

3s

Column chromatography (PE/CH_2Cl_2 = 1:0 to 20:1, v/v) afforded product **3s** as colorless oil (66.8 mg, 78% yield). ^1H NMR (400 MHz, $CDCl_3$): δ 7.83 (d, J = 8.0 Hz, 2H), 7.49 (t, J = 7.6 Hz, 1H), 7.35-7.29 (m, 3H), 7.21-7.10 (m, 3H), 3.02-2.85 (m, 2H), 2.71-2.64 (m, 1H), 2.03-1.95 (m, 1H), 1.57 (s, 3H); ^{13}C NMR (100 MHz, $CDCl_3$): δ 190.38 (t, J = 31.6 Hz), 144.46, 143.53 (t, J = 2.5 Hz), 133.59, 130.00 (t, J = 3.9 Hz), 128.22, 127.90, 126.37, 125.20 (t, J = 1.4 Hz), 124.63, 121.05 (t, J = 257.7 Hz), 53.81 (t, J = 21.5 Hz), 34.91 (t, J = 3.3 Hz), 30.87, 22.57 (t, J = 4.7 Hz); ^{19}F NMR (376 MHz, $CDCl_3$): δ -104.45 (s, 1F), -104.47 (s, 1F); IR

(ATR): 2976, 1693, 1599, 1580, 1514, 1449, 1271, 1134, 1067, 1001, 978, 810, 758, 687; HRMS (ESI): Exact mass calcd for $C_{18}H_{16}F_2NaO$ $[M+Na]^+$: 309.1061, Found: 309.1061.

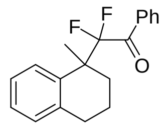

3t

Column chromatography (PE/CH_2Cl_2 = 1:0 to 20:1, v/v) afforded product **3t** as white solid (71.3 mg, 79% yield). m.p. 48-50 °C; ^{1}H NMR (400 MHz, $CDCl_3$): δ 7.85 (d, *J* = 8.4 Hz, 2H), 7.52-7.47 (m, 1H), 7.42 (d, *J* = 8.0 Hz, 1H), 7.34 (t, *J* = 8.4 Hz, 2H), 7.11-7.01 (m, 3H), 2.85-2.69 (m, 2H), 2.26-2.18 (m, 1H), 1.95-1.89 (m, 1H), 1.79-1.74 (m, 1H), 1.69-1.65 (m, 1H), 1.57 (s, 3H); ^{13}C NMR (100 MHz, $CDCl_3$): δ 190.18 (t, *J* = 32.2 Hz), 138.38, 137.16 (d, *J* = 4.1 Hz), 134.15 (t, *J* = 2.6 Hz), 133.44, 129.89 (t, *J* = 3.9 Hz), 129.14, 128.31 (d, *J* = 1.9 Hz), 128.22, 126.59, 125.84, 121.25 (dd, *J* = 260.8, 258.7 Hz), 43.95 (dd, *J* = 20.5, 18.8 Hz), 32.47 (t, *J* = 3.4 Hz), 30.48, 24.85 (dd, *J* = 6.9, 3.1 Hz), 19.48; ^{19}F NMR (376 MHz, $CDCl_3$): δ -99.83 (d, *J* = 283.5 Hz), -99.84 (dd, *J* = 283.9, 2.6 Hz), -102.80 (d, *J* = 283.5 Hz), -102.81 (dd, *J* = 283.1, 2.6 Hz); IR (ATR): 2978, 1597, 1580, 1448, 1325, 1283, 1179, 1086, 920, 800, 733; HRMS (ESI): Exact mass calcd for $C_{19}H_{19}F_2O$ $[M+H]^+$: 301.1398, Found: 301.1410.

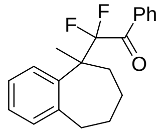

3u

Column chromatography (PE/CH_2Cl_2 = 1:0 to 20:1, v/v) afforded product **3u** as colorless oil (93.4 mg, 99% yield). ^{1}H NMR (400 MHz, $CDCl_3$): δ 7.54 (d, *J* = 8.0 Hz, 2H), 7.40 (t, *J* = 7.6 Hz, 1H), 7.31 (d, *J* = 7.6 Hz, 1H), 7.23-7.18 (m, 2H), 7.09-7.01 (m, 2H), 6.95 (d, *J* = 6.8 Hz, 1H), 3.27-3.19 (m, 1H), 2.59-2.53 (m, 1H), 2.43-2.39 (m, 1H), 1.79-1.76 (m, 3H), 1.67-1.60 (m, 4H), 1.43-1.35 (m, 1H); ^{13}C NMR (100 MHz, $CDCl_3$): δ 191.71 (t, *J* = 29.8 Hz), 141.48, 137.19 (t, *J* = 2.4 Hz), 134.23, 133.21, 130.98, 130.82, 129.84 (t, *J* = 4.1 Hz), 128.03, 127.92, 126.30, 121.52 (t, *J* = 259.4 Hz), 50.69 (t, *J* = 20.7 Hz), 32.41, 31.53, 26.30, 25.18 (t, *J* = 4.4 Hz), 18.92; ^{19}F NMR (376 MHz, $CDCl_3$): δ -104.37 (d, *J* = 256.1 Hz, 1F), -105.38 (d, *J* = 256.4 Hz, 1F); IR (ATR): 2976, 1597, 1579, 1483, 1449, 1325, 1236, 1165, 1028, 910, 800, 752; HRMS (ESI): Exact mass calcd for $C_{20}H_{20}F_2NaO$ $[M+Na]^+$: 337.1374, Found: 337.1381.

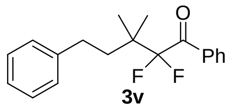

3v

Column chromatography (PE/CH_2Cl_2 = 1:0 to 20:1, v/v) afforded product **3v** as colorless oil (53.7 mg, 59% yield). ^{1}H NMR (400 MHz, $CDCl_3$): δ 8.07 (d, *J* = 8.0 Hz, 2H), 7.59 (t, *J* = 7.6 Hz, 1H), 7.46 (t, *J* = 8.0 Hz, 2H), 7.29-7.24 (m, 2H), 7.19-7.15 (m, 3H), 2.68-2.63 (m, 2H), 1.86-1.82 (m, 2H), 1.21(s, 6H); ^{13}C NMR (100 MHz, $CDCl_3$): δ 190.76 (t, *J* = 31.3 Hz), 142.33, 134.46 (t, *J* = 2.3 Hz), 133.77, 130.13 (t, *J* = 4.4 Hz), 128.52, 128.37, 128.29, 125.80, 121.98 (t, *J* = 257.4 Hz), 40.78 (t, *J* = 20.5 Hz), 38.46 (t, *J* = 3.3 Hz), 30.36, 21.32;

^{19}F NMR (376 MHz, $CDCl_3$): δ -106.74 (s, 2F); IR (ATR): 2978, 1611, 1514, 1449, 1242, 1136, 1053, 904, 880; HRMS (ESI): Exact mass calcd for $C_{19}H_{20}F_2NaO$ $[M+Na]^+$: 325.1374, Found: 325.1376.

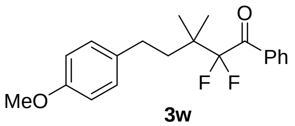

3w

The reaction used 20 mol% of $Mg(ClO_4)_2·6H_2O$ and 3.0 equiv. of **2a**. Column chromatography (PE/CH_2Cl_2 = 10:1, v/v) afforded product **3w** as colorless oil (33.0 mg, 33% yield). ^1H NMR (400 MHz, $CDCl_3$): δ 8.08-8.05 (m, 2H), 7.61-7.57 (m, 1H), 7.48-7.44 (m, 2H), 7.08-7.04 (m, 2H), 6.83-6.79 (m, 2H), 3.77 (s, 3H), 2.62-2.57 (m, 2H), 1.83-1.78 (m, 2H), 1.20 (s, 6H); ^{13}C NMR (100 MHz, $CDCl_3$): δ 190.83 (t, *J* = 30.1 Hz), 157.79, 134.57 (t, *J* = 2.2 Hz), 134.43, 133.73, 130.13 (t, *J* = 4.3 Hz), 129.16, 128.52, 122.02 (t, *J* = 257.2 Hz), 113.84, 55.25, 40.78 (t, *J* = 20.5 Hz), 38.71 (t, *J* = 3.2 Hz), 29.43, 21.33 (t, *J* = 4.4 Hz); ^{19}F NMR (376 MHz, $CDCl_3$): δ -106.77 (s, 2F); IR (ATR): 2958, 1612, 1578, 1448, 1175, 1016, 980, 822, 750; HRMS (ESI): Exact mass calcd for $C_{20}H_{22}F_2NaO_2$ $[M+Na]^+$: 355.1480, Found: 355.1469.

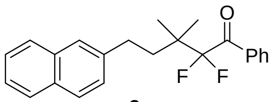

3x

The reaction used 20 mol% of $Mg(ClO_4)_2·6H_2O$ and 3.0 equiv. of **2a**. Column chromatography (PE/CH_2Cl_2 = 1:0 to 20:1, v/v) afforded product **3x** as white solid (30.7 mg, 29% yield). m.p. 45-47 °C; ^1H NMR (400 MHz, $CDCl_3$): δ 8.08 (d, *J* = 7.6 Hz, 2H), 7.79-7.74 (m, 3H), 7.61-7.57 (m, 2H), 7.48-7.40 (m, 4H), 7.30-7.28 (m, 1H), 2.84-2.80 (m, 2H), 1.95-1.90 (m, 2H), 1.25 (s, 6H); ^{13}C NMR (100 MHz, $CDCl_3$): δ 190.82 (t, *J* =31.4 Hz), 139.85, 134.58 (t, *J* = 2.0 Hz), 133.76, 133.67, 132.00, 130.14 (t, *J* = 4.4 Hz), 128.54, 127.94, 127.58, 127.36, 127.23, 126.22, 125.89, 125.13, 122.03 (t, *J* = 257.4 Hz), 40.89 (t, *J* = 20.5 Hz), 38.44 (t, *J* = 3.1 Hz), 30.57 (t, *J* = 1.8 Hz), 21.41 (t, *J* = 4.4 Hz); ^{19}F NMR (376 MHz, $CDCl_3$): δ -106.65 (s, 2F); IR (ATR): 2974, 1686, 1597, 1447, 1275, 1140, 1051, 864, 756; HRMS (ESI): Exact mass calcd for $C_{23}H_{22}F_2NaO$ $[M+Na]^+$: 375.1531, Found: 375.1522.

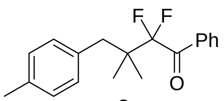

3y

Column chromatography (PE/CH_2Cl_2 = 1:0 to 20:1, v/v) afforded product **3y** as colorless oil (46.6 mg, 51% yield). ^1H NMR (400 MHz, $CDCl_3$): δ 7.87 (d, *J* = 8.4 Hz, 2H), 7.58-7.54 (m, 1H), 7.43-7.38 (m, 2H), 7.02 (s, 4H), 3.38-3.27 (m, 1H), 2.43-2.35 (m, 1H), 2.28 (s, 3H), 1.09 (d, *J* = 6.4 Hz, 3H), 0.80 (d, *J* = 6.4 Hz, 3H); ^{13}C NMR (100 MHz, $CDCl_3$): δ 190.21 (t, *J* = 29.9 Hz), 137.05, 133.58, 133.21, 132.10 (d, *J* = 6.2 Hz), 129.96, 129.66 (t, *J* = 3.4 Hz), 128.92, 128.41, 120.41 (t, *J* = 257.1 Hz), 55.76 (t, *J* = 20.6 Hz), 28.83 (d, *J* =

3.5 Hz), 22.30 (d, *J* = 3.2 Hz), 20.99; ^{19}F NMR (376 MHz, $CDCl_3$): δ -98.32 (d, *J* = 272.2 Hz, 1F), -104.10 (d, *J* = 271.8 Hz, 1F); IR (ATR): 2972, 1705, 1599, 1580, 1516, 1449, 1267, 1179, 1092, 916, 864, 795; HRMS (ESI): Exact mass calcd for $C_{19}H_{20}F_2NaO$ $[M+Na]^+$: 325.1374, Found: 325.1372.

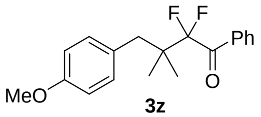

3z

Column chromatography (PE/CH_2Cl_2 = 6:1, v/v) afforded product **3z** as colorless oil (78.8 mg, 83% yield). ^{1}H NMR (400 MHz, $CDCl_3$): δ 7.85 (d, *J* = 7.6 Hz, 2H), 7.56-7.52 (m, 1H), 7.41-7.37 (m, 2H), 7.04 (d, *J* = 8.4 Hz, 2H), 6.76-6.73 (m, 2H), 3.73 (s, 3H), 3.36-3.25 (m, 1H), 2.44-2.32 (m, 1H), 1.09 (dd, *J* = 6.8, 2.0 Hz, 3H), 0.80 (d, *J* = 6.8 Hz, 3H); ^{13}C NMR (100 MHz, $CDCl_3$): δ 190.17 (t, *J* = 29.6 Hz), 158.85, 133.57, 133.17, 131.09, 129.56 (t, *J* = 3.3 Hz), 128.40, 127.01 (d, *J* = 6.8 Hz), 120.39 (t, *J* = 257.2 Hz), 113.59, 55.37 (t, *J* = 20.8 Hz), 55.03 (d, *J* = 2.3 Hz), 28.86 (d, *J* = 3.3 Hz), 22.23 (d, *J* = 3.4 Hz), 20.93; ^{19}F NMR (376 MHz, $CDCl_3$): δ -97.71 (d, *J* = 270.3 Hz, 1F), -105.25 (d, *J* = 270.3 Hz, 1F); IR (ATR): 2974, 1701, 1599, 1582, 1514, 1449, 1261, 1171, 1051, 910, 800, 707; HRMS (ESI): Exact mass calcd for $C_{19}H_{20}F_2NaO_2$ $[M+Na]^+$: 341.1324, Found: 341.1327.

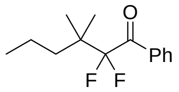

3aa

The reaction used 20 mol% of $Mg(ClO_4)_2{\cdot}6H_2O$ and 3.0 equiv. of **2a**. Column chromatography (PE) afforded product **3aa** as colorless oil (20.1 mg, 28% yield). ^{1}H NMR (400 MHz, $CDCl_3$): δ 8.07-8.05 (m, 2H), 7.61-7.57 (m, 1H), 7.48-7.44 (m, 2H), 1.51-1.46 (m, 2H), 1.36-1.30 (m, 2H), 1.10 (s, 6H), 0.88 (t, *J* = 7.2 Hz, 3H); ^{13}C NMR (100 MHz, $CDCl_3$): δ 191.05 (t, *J* = 31.1 Hz), 134.71, 133.66, 130.13 (t, *J* = 4.4 Hz), 128.48, 122.23 (t, *J* = 256.9 Hz), 40.73 (t, *J* = 20.3 Hz), 38.34 (t, *J* = 3.2 Hz), 21.07 (t, *J* = 4.6 Hz), 16.93, 14.81; ^{19}F NMR (376 MHz, $CDCl_3$): δ -107.46 (s, 2F); IR (ATR): 1751, 1597, 1448, 1134, 1109, 1003, 783, 688; HRMS (ESI): Exact mass calcd for $C_{14}H_{18}F_2NaO$ $[M+Na]^+$: 263.1218, Found: 263.1210.

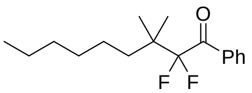

3ab

Column chromatography (PE) afforded product **3ab** as colorless oil (43.1 mg, 51% yield). ^{1}H NMR (400 MHz, $CDCl_3$): δ 8.07-8.05 (m, 2H), 7.61-7.56 (m, 1H), 7.48-7.44 (m, 2H), 1.52-1.47 (m, 2H), 1.33-1.25 (m, 8H), 1.10 (s, 6H), 0.87 (t, *J* = 6.8 Hz, 3H); ^{13}C NMR (100 MHz, $CDCl_3$): δ 191.05 (t, *J* = 31.2 Hz), 134.75 (t, *J* = 2.1 Hz), 133.63, 130.12 (t, *J* = 4.4 Hz), 128.46, 122.26 (t, *J* = 257.0 Hz), 40.67 (t, *J* = 20.4 Hz), 36.01 (t, *J* = 3.2 Hz), 31.74, 30.04, 23.59, 22.60, 21.03 (t, *J* = 4.4 Hz), 14.03; ^{19}F NMR (376 MHz, $CDCl_3$): δ -107.45 (s, 2F); IR (ATR): 1749, 1709, 1597, 1448, 1217, 1190, 1132, 901, 825, 715; HRMS (ESI): Exact mass calcd for $C_{17}H_{24}F_2NaO$ $[M+Na]^+$: 305.1687, Found: 305.1682.

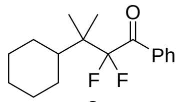
3ac

The reaction used 20 mol% of $Mg(ClO_4)_2·6H_2O$ and 3.0 equiv. of **2a**. Column chromatography (PE) afforded product **3ac** as colorless oil (50.2 mg, 60% yield). ^1H NMR (400 MHz, $CDCl_3$): δ 8.05-8.02 (m, 2H), 7.61-7.57 (m, 1H), 7.48-7.44 (m, 2H), 1.78-1.71 (m, 4H), 1.67-1.60 (m, 2H), 1.18-1.02 (m, 11H); ^{13}C NMR (125 MHz, $CDCl_3$): δ 191.38 (t, *J* = 31.4 Hz), 134.81 (t, *J* = 2.4 Hz), 133.53, 130.04 (t, *J* = 4.2 Hz), 128.45, 122.73 (t, *J* = 258.0 Hz), 43.77 (t, *J* = 19.1 Hz), 42.80 (t, *J* = 1.8 Hz), 28.24 (t, *J* = 2.0 Hz), 26.99, 26.47, 19.62 (t, *J* = 5.4 Hz); ^{19}F NMR (376 MHz, $CDCl_3$): δ -101.92 (s, 2F); IR (ATR): 2853, 1717, 1597, 1448, 1288, 1130, 1003, 949, 826, 717; HRMS (ESI): Exact mass calcd for $C_{17}H_{22}F_2NaO$ $[M+Na]^+$: 303.1531, Found: 303.1538.

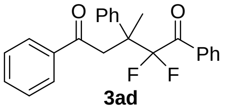
3ad

Column chromatography (PE/EtOAc = 40:1, v/v) afforded product **3ad** as colorless oil (88.5 mg, 78% yield). ^1H NMR (400 MHz, $CDCl_3$): δ 7.94 (d, *J* = 7.6 Hz, 2H), 7.57-7.53 (m, 1H), 7.50-7.41 (m, 5H), 7.33 (d, *J* = 7.6 Hz, 2H), 7.24-7.13 (m, 5H), 4.40 (d, *J* = 18.4 Hz, 1H), 3.66 (d, *J* = 18.4 Hz, 1H), 1.94 (s, 3H); ^{13}C NMR (100 MHz, $CDCl_3$): δ 195.78, 191.45 (t, *J* = 30.0 Hz), 138.25 (t, *J* = 2.2 Hz), 137.52, 134.09 (t, *J* = 2.1 Hz), 133.40, 133.07, 129.75 (t, *J* = 4.0 Hz), 128.55, 128.20, 128.01, 127.81, 127.32, 119.96 (t, *J* = 261.3 Hz), 47.37 (t, *J* = 20.4 Hz), 41.59, 18.91 (t, *J* = 3.7 Hz); ^{19}F NMR (376 MHz, $CDCl_3$): δ -105.06 (d, *J* = 257.9 Hz, 1F), -105.93 (d, *J* = 258.3 Hz, 1F); IR (ATR): 2976, 1689, 1598, 1580, 1499, 1447, 1221, 1157, 1001, 843, 754; HRMS (ESI): Exact mass calcd for $C_{24}H_{20}F_2NaO_2$ $[M+Na]^+$: 401.1324, Found: 401.1326.

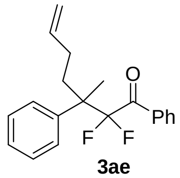
3ae

Column chromatography (PE/CH_2Cl_2 = 1:0 to 20:1, v/v) afforded product **3ae** as colorless oil (67.0 mg, 71% yield). ^1H NMR (400 MHz, $CDCl_3$): δ 7.55 (d, *J* = 8.4 Hz, 2H), 7.43-7.39 (m, 1H), 7.37-7.35 (m, 2H), 7.23-7.14 (m, 5H), 5.85-5.74 (m, 1H), 5.00-4.92 (m, 2H), 2.44 (td, *J* = 13.2, 3.6 Hz, 1H), 2.07-1.92 (m, 2H), 1.79-1.69 (m, 1H), 1.63 (s, 3H); ^{13}C NMR (100 MHz, $CDCl_3$): δ 191.09 (t, *J* = 30.2 Hz), 138.24 (t, *J* = 2.4 Hz), 138.19, 134.31 (t, *J* = 1.9 Hz), 133.20, 129.74 (t, *J* = 4.0 Hz), 128.60, 128.16, 127.98, 127.34, 120.63 (t, *J* = 260.3 Hz), 114.56, 48.00 (t, *J* = 20.4 Hz), 32.84 (t, *J* = 4.0 Hz), 27.59, 18.68 (t, *J* = 4.3 Hz); ^{19}F NMR (376 MHz, $CDCl_3$): δ -104.78 (dd, *J* = 259.8, 2.6 Hz, 1F), -106.15 (dd, *J* = 259.4, 2.6 Hz, 1F); IR (ATR): 2976, 1689, 1599, 1580, 1499, 1449, 1269, 1049, 908, 833, 714, 698; HRMS (ESI): Exact mass calcd for $C_{20}H_{20}F_2NaO$ $[M+Na]^+$: 337.1374, Found: 337.1373.

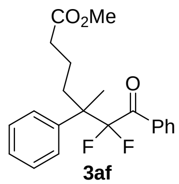

3af

Column chromatography (PE/CH_2Cl_2 = 2:1, v/v) afforded product **3af** as colorless oil (89.0 mg, 82% yield). ^1H NMR (400 MHz, $CDCl_3$): δ 7.52 (d, J = 7.6 Hz, 2H), 7.42-7.38 (m, 1H), 7.35 (d, J = 7.6 Hz, 2H), 7.23-7.13 (m, 5H), 3.63 (s, 3H), 2.42-2.29 (m, 3H), 1.94 (td, J = 12.8, 4.8 Hz, 1H), 1.64 (s, 3H), 1.60-1.51 (m, 1H), 1.38-1.26 (m, 1H); ^{13}C NMR (100 MHz, $CDCl_3$): δ 191.05 (t, J = 30.2 Hz), 173.49, 138.03 (t, J = 2.4 Hz), 134.22 (t, J = 1.9 Hz), 133.15, 129.67 (t, J = 3.9 Hz), 128.54, 128.14, 127.91, 127.34, 120.50 (t, J = 260.5 Hz), 51.36 (d, J = 2.5 Hz), 47.96 (t, J = 20.4 Hz), 34.12, 33.03 (t, J = 3.4 Hz), 18.84, 18.58 (t, J = 4.3 Hz); ^{19}F NMR (376 MHz, $CDCl_3$): δ -104.83 (d, J = 259.4 Hz, 1F), -106.17 (d, J = 259.4 Hz, 1F); IR (ATR): 2974, 1692, 1597, 1578, 1499, 1447, 1271, 1169, 1049, 937, 897, 716; HRMS (ESI): Exact mass calcd for $C_{21}H_{22}F_2NaO_3$ $[M+Na]^+$: 383.1429, Found: 383.1421.

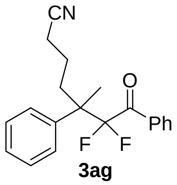

3ag

Column chromatography (PE/CH_2Cl_2 = 2:1 to 1:1, v/v) afforded product **3ag** as colorless oil (68.8 mg, 70% yield). ^1H NMR (400 MHz, $CDCl_3$): δ 7.54 (d, J = 8.0 Hz, 2H), 7.42 (t, J = 7.2 Hz, 1H), 7.35 (d, J = 7.6 Hz, 2H), 7.25-7.16 (m, 5H), 2.47 (td, J = 13.2, 3.6 Hz, 1H), 2.31 (t, J = 6.8 Hz, 2H), 2.09 (td, J = 13.2, 5.2 Hz, 1H), 1.63 (s, 3H), 1.61-1.56 (m, 1H), 1.40-1.33 (m, 1H); ^{13}C NMR (100 MHz, $CDCl_3$): δ 190.70 (t, J = 30.3 Hz), 137.58, 133.99, 133.32, 129.64 (t, J = 4.0 Hz), 128.35, 128.29, 127.99, 127.59, 120.18 (t, J = 260.8 Hz), 119.18, 47.75 (t, J = 20.6 Hz), 33.00 (t, J = 4.2 Hz), 19.84, 18.55 (t, J = 4.4 Hz), 17.44; ^{19}F NMR (376 MHz, $CDCl_3$): δ -104.51 (d, J = 262.1 Hz, 1F), -105.91 (d, J = 262.4 Hz, 1F); IR (ATR): 2974, 1690, 1597, 1578, 1499, 1449, 1273, 1103, 1049, 937, 895, 716; HRMS (ESI): Exact mass calcd for $C_{20}H_{19}F_2NNaO$ $[M+Na]^+$: 350.1327, Found: 350.1320.

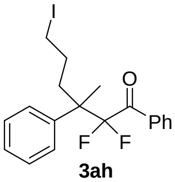

3ah

Column chromatography (PE/CH_2Cl_2 = 20:1 to 10:1, v/v) afforded product **3ah** as colorless oil (122.3 mg, 95% yield). ^1H NMR (400 MHz, $CDCl_3$): δ 7.54 (d, J = 8.0 Hz, 2H), 7.43-7.39 (m, 1H), 7.35 (d, J = 7.2 Hz, 2H), 7.24-7.14 (m, 5H), 3.18-3.08 (m, 2H), 2.46 (td, J = 13.2, 4.4 Hz, 1H), 2.08-2.00 (m, 1H), 1.79-1.68 (m, 1H), 1.62 (s, 3H), 1.56-1.45 (m, 1H); ^{13}C NMR (100 MHz, $CDCl_3$): δ 190.88 (t, J = 30.3 Hz), 137.96 (t, J = 2.5 Hz), 134.15 (t, J = 2.1 Hz), 133.24, 129.68 (t, J = 4.0 Hz), 128.48, 128.22, 127.97, 127.47, 120.33 (t, J = 260.7 Hz), 47.64 (t, J = 20.5 Hz), 34.81 (t, J = 3.7 Hz), 27.54, 18.83 (t, J = 4.3 Hz), 6.53; ^{19}F NMR (376 MHz, $CDCl_3$): δ -104.60 (d, J = 260.9 Hz, 1F), -105.99 (d, J = 260.9 Hz, 1F); IR (ATR): 2974,

1690, 1597, 1578, 1499, 1447, 1273, 1101, 1049, 932, 891, 714; HRMS (ESI): Exact mass calcd for $C_{19}H_{19}F_2INaO$ [M+Na]$^+$: 451.0341, Found: 451.0336.

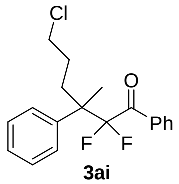
3ai

Column chromatography (PE/CH_2Cl_2 = 20:1 to 10:1, v/v) afforded product **3ai** as colorless oil (80.4 mg, 80% yield). ^1H NMR (400 MHz, $CDCl_3$): δ 7.54 (d, *J* = 7.2 Hz, 2H), 7.44-7.39 (m, 1H), 7.37-7.35 (m, 2H), 7.24-7.15 (m, 5H), 3.55-3.47 (m, 2H), 2.51 (td, *J* = 13.2, 4.0 Hz, 1H), 2.10-2.03 (m, 1H), 1.76-1.65 (m, 1H), 1.63 (s, 3H), 1.52-1.43 (m, 1H); ^{13}C NMR (100 MHz, $CDCl_3$): δ 190.96 (t, *J* = 30.2 Hz), 137.93 (t, *J* = 2.5 Hz), 134.17 (t, *J* = 1.9 Hz), 133.27, 129.71 (t, *J* = 3.9 Hz), 128.54, 128.25, 127.99, 127.48, 120.43 (t, *J* = 260.7 Hz), 47.74 (t, *J* = 20.4 Hz), 45.18, 31.24, 26.78, 18.72 (t, *J* = 4.3 Hz); ^{19}F NMR (376 MHz, $CDCl_3$): δ -104.68 (d, *J* = 260.9 Hz, 1F), -106.05 (d, *J* = 260.6 Hz, 1F); IR (ATR): 2976, 1690, 1599, 1578, 1499, 1447, 1271, 1101, 1051, 714, 698; HRMS (ESI): Exact mass calcd for $C_{19}H_{19}ClF_2NaO$ [M+Na]$^+$: 359.0985, Found: 359.0981.

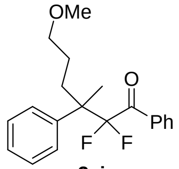
3aj

Column chromatography (PE/CH_2Cl_2 = 2:1, v/v) afforded product **3aj** as colorless oil (78.0 mg, 78% yield). ^1H NMR (400 MHz, $CDCl_3$): δ 7.53 (d, *J* = 7.6 Hz, 2H), 7.42-7.35 (m, 3H), 7.24-7.12 (m, 5H), 3.34 (t, *J* = 6.4 Hz, 2H), 3.28 (s, 3H), 2.42 (td, *J* = 13.2, 4.0 Hz, 1H), 1.99 (td, *J* = 12.8, 4.8 Hz, 1H), 1.62 (s, 3H), 1.54-1.43 (m, 1H), 1.30-1.24 (m, 1H); ^{13}C NMR (100 MHz, $CDCl_3$): δ 191.12 (t, *J* = 30.2 Hz), 138.27 (t, *J* = 2.3 Hz), 134.27 (t, *J* = 2.2 Hz), 133.13, 129.70 (t, *J* = 4.0 Hz), 128.61, 128.11, 127.92, 127.26, 120.65 (t, *J* = 260.3 Hz), 72.79, 58.45, 47.84 (t, *J* = 20.4 Hz), 30.23 (t, *J* = 3.9 Hz), 23.65, 18.65 (t, *J* = 4.4 Hz); ^{19}F NMR (376 MHz, $CDCl_3$): δ -104.71 (d, *J* = 259.4 Hz, 1F), -106.13 (d, *J* = 259.4 Hz, 1F); IR (ATR): 2974, 1692, 1597, 1580, 1499, 1449, 1275, 1115, 1098, 903, 714; HRMS (ESI): Exact mass calcd for $C_{20}H_{22}F_2NaO_2$ [M+Na]$^+$: 355.1480, Found: 355.1475.

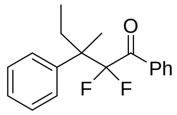
5a = 3a

The *E*/*Z* ratio of alkene **4a** used in this reaction is 4.6:1. Column chromatography (PE/CH_2Cl_2 = 1:0 to 20:1, v/v) afforded product **5a** as colorless oil (67.6 mg, 78% yield). ^1H NMR (400 MHz, $CDCl_3$): δ 7.55 (d, *J* = 8.0 Hz, 2H), 7.43 (t, *J* = 7.2 Hz, 1H), 7.35 (d, *J* = 7.6 Hz, 2H), 7.24-7.15 (m, 5H), 2.46-2.37 (m, 1H), 1.99-1.89 (m, 1H), 1.60 (s, 3H), 0.75 (t, *J* = 7.6 Hz, 3H); ^{13}C NMR (100 MHz, $CDCl_3$): δ 191.35 (t, *J* = 30.2 Hz), 138.12 (t, *J* = 2.4 Hz), 134.32, 133.19, 129.77 (t, *J* = 4.0 Hz), 128.78, 128.08, 127.97, 127.22, 120.91 (t, *J* = 260.1 Hz), 48.30 (t, *J* = 20.2 Hz), 26.20 (t, *J* = 3.9 Hz), 18.13 (t, *J* = 4.4 Hz), 7.65; ^{19}F NMR (376 MHz, $CDCl_3$):

δ -104.97 (d, *J* = 257.9 Hz, 1F), -106.30 (d, *J* = 257.9 Hz, 1F); IR (ATR): 2978, 1598, 1500, 1448, 1200, 1134, 1024, 905, 714, 681; HRMS (ESI): Exact mass calcd for $C_{18}H_{18}F_2NaO$ $[M+Na]^+$: 311.1218, Found: 311.1225.

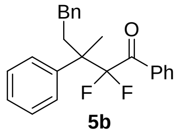

5b

The *E*/*Z* ratio of alkene **4b** used in this reaction is 1:2.4. Column chromatography (PE/CH_2Cl_2 = 10:1, v/v) afforded product **5b** as colorless oil (135.9 mg, 92% yield). ^1H NMR (400 MHz, $CDCl_3$): δ 7.53 (d, *J* = 8.0 Hz, 2H), 7.43 (d, *J* = 7.6 Hz, 2H), 7.37 (t, *J* = 7.6 Hz, 1H), 7.27-7.22 (m, 4H), 7.20-7.12 (m, 6H), 2.66-2.50 (m, 2H), 2.33-2.23 (m, 2H), 1.72 (s, 3H); ^{13}C NMR (100 MHz, $CDCl_3$): δ 190.99 (t, *J* = 30.2 Hz), 141.97, 138.19 (t, *J* = 2.3 Hz), 134.15 (t, *J* = 2.1 Hz), 133.20, 129.71 (t, *J* = 4.0 Hz), 128.62, 128.38, 128.26, 128.22, 127.95, 127.42, 125.86, 120.54 (t, *J* = 260.5 Hz), 48.15 (t, *J* = 20.4 Hz), 35.73, 29.70, 18.73 (t, *J* = 4.3 Hz); ^{19}F NMR (376 MHz, $CDCl_3$): δ -104.46 (d, *J* = 260.6 Hz, 1F), -105.94 (d, *J* = 260.6 Hz, 1F); IR (ATR): 2936, 1670, 1449, 1277, 1105, 1047, 905, 745, 687; HRMS (ESI): Exact mass calcd for $C_{24}H_{22}F_2NaO$ $[M+Na]^+$: 387.1531, Found: 387.1534.

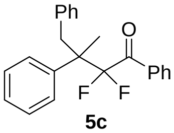

5c

The *E*/*Z* ratio of alkene **4c** used in this reaction is 1.5:1. Column chromatography (PE/ CH_2Cl_2 = 10:1, v/v) afforded product **5c** as white solid (87.0 mg, 83% yield). m.p. 75-77 °C; ^1H NMR (400 MHz, $CDCl_3$): δ 7.67 (d, *J* = 7.6 Hz, 2H), 7.46-7.41 (m, 3H), 7.27 (t, *J* = 7.6 Hz, 2H), 7.23-7.16 (m, 3H), 7.11-7.04 (m, 3H), 6.81-6.79 (m, 2H), 3.68 (d, *J* = 13.6 Hz, 1H), 3.26 (d, *J* = 14.0 Hz, 1H), 1.47 (s, 3H); ^{13}C NMR (100 MHz, $CDCl_3$): δ 190.77 (t, *J* = 30.6 Hz), 138.56 (d, *J* = 3.4 Hz), 136.34, 134.32, 133.30, 130.73, 129.76 (t, *J* = 3.9 Hz), 128.76, 128.08, 128.02, 127.61, 127.40, 126.32, 120.79 (t, *J* = 261.5 Hz), 48.88 (t, *J* = 19.8 Hz), 40.32, 18.50 (t, *J* = 4.3 Hz); ^{19}F NMR (376 MHz, $CDCl_3$): δ -102.70 (d, *J* = 266.6 Hz, 1F), -104.97 (d, *J* = 266.6 Hz, 1F); IR (ATR): 1703, 1597, 1447, 1283, 1171, 1082, 1040, 901, 853, 739, 669; HRMS (ESI): Exact mass calcd for $C_{23}H_{20}F_2NaO$ $[M+Na]^+$: 373.1374, Found: 373.1377.

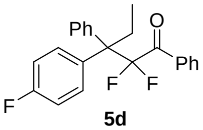

5d

The *E*/*Z* ratio of alkene **4d** used in this reaction is 1:1. Column chromatography (PE/CH_2Cl_2 = 1:0 to 20:1, v/v) afforded product **5d** as colorless oil (73.1 mg, 66% yield). ^1H NMR (400 MHz, $CDCl_3$): δ 7.43 (d, *J* = 8.0 Hz, 2H), 7.39 (d, *J* = 7.6 Hz, 1H), 7.33-7.30 (m, 4H), 7.24-7.17 (m, 5H), 6.92-6.88 (m, 2H), 2.60-2.47 (m, 2H), 0.75 (t, *J* = 7.2 Hz, 3H); ^{13}C NMR (100 MHz, $CDCl_3$): δ 191.24 (t, *J* = 29.9 Hz), 161.67 (d, *J* = 245.6 Hz), 139.68, 135.70, 134.41, 133.01, 132.26 (t, *J* = 2.1 Hz), 132.18 (t, *J* = 2.2 Hz), 130.38 (t, *J* = 2.0 Hz), 129.45 (t, *J* = 4.2

Hz), 127.83, 127.22, 121.28 (t, J = 265.5 Hz), 114.49 (d, J = 21.0 Hz), 58.19 (t, J = 19.0 Hz), 28.42, 9.24; ^{19}F NMR (376 MHz, $CDCl_3$): δ -98.13 (s, 2F), -115.55 (s, 1F); IR (ATR): 2976, 1692, 1599, 1510, 1447, 1265, 1166, 1049, 912, 829, 700; HRMS (ESI): Exact mass calcd for $C_{23}H_{19}F_3NaO$ $[M+Na]^+$: 391.1280, Found: 391.1283.

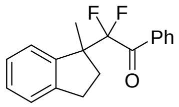

5e = 3n

Column chromatography (PE/CH_2Cl_2 = 1:0 to 20:1, v/v) afforded product **5e** as colorless oil (71.8 mg, 84% yield). ^{1}H NMR (400 MHz, $CDCl_3$): δ 7.83 (d, J = 8.0 Hz, 2H), 7.51-7.47 (m, 1H), 7.35-7.30 (m, 3H), 7.20-7.10 (m, 3H), 3.02-2.85 (m, 2H), 2.71-2.64 (m, 1H), 2.03-1.95 (m, 1H), 1.57 (s, 3H); ^{13}C NMR (100 MHz, $CDCl_3$): δ 190.34 (t, J = 31.5 Hz), 144.44, 143.48 (t, J = 2.5 Hz), 133.61, 129.99 (t, J = 3.9 Hz), 128.20, 127.88, 126.35, 125.17 (t, J = 1.5 Hz), 124.61, 121.01 (t, J = 257.6 Hz), 53.75 (t, J = 21.4 Hz), 34.86 (t, J = 3.3 Hz), 30.85, 22.52 (t, J = 4.7 Hz); ^{19}F NMR (376 MHz, $CDCl_3$): δ -104.43 (d, J = 2.3 Hz, 1F), -104.45 (d, J = 3.0 Hz, 1F); IR (ATR): 2978, 1670, 1514, 1449, 1379, 1256, 1091, 914, 802, 758; HRMS (ESI): Exact mass calcd for $C_{18}H_{16}F_2NaO$ $[M+Na]^+$: 309.1061, Found: 309.1064.

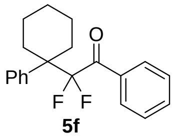

5f

Column chromatography (PE/CH_2Cl_2 = 1:0 to 20:1, v/v) afforded product **5f** as white solid (74.4 mg, 79% yield). m.p. 102-104 ^{o}C; ^{1}H NMR (400 MHz, $CDCl_3$): δ 7.42-7.36 (m, 5H), 7.24-7.13 (m, 5H), 2.60 (d, J = 13.6 Hz, 2H), 1.97-1.89 (m, 2H), 1.63-1.54 (m, 3H), 1.31-1.22 (m, 3H); ^{13}C NMR (100 MHz, $CDCl_3$): δ 191.84 (t, J = 29.6 Hz), 135.91 (t, J = 2.4 Hz), 134.47, 133.04, 129.65, 129.60, 128.47, 127.86, 127.27, 120.44 (t, J = 259.7 Hz), 49.09 (t, J = 20.3 Hz), 28.38 (t, J = 4.0 Hz), 25.97, 21.11; ^{19}F NMR (376 MHz, $CDCl_3$): δ -107.95 (s, 2F); IR (ATR): 2976, 1597, 1580, 1500, 1449, 1201, 1113, 1001, 881, 768, 681; HRMS (ESI): Exact mass calcd for $C_{20}H_{20}F_2NaO$ $[M]^+$: 337.1374, Found: 337.1369.

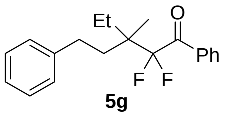

5g

The E/Z ratio of alkene **4g** used in this reaction is 1:1. Column chromatography (PE/CH_2Cl_2 = 1:0 to 20:1, v/v) afforded product **5g** as colorless oil (48.9 mg, 52% yield). ^{1}H NMR (400 MHz, $CDCl_3$): δ 8.07 (d, J = 8.0 Hz, 2H), 7.62-7.58 (m, 1H), 7.49-7.45 (m, 2H), 7.29-7.25 (m, 2H), 7.19-7.14 (m, 3H), 2.71-2.60 (m, 2H), 1.90-1.60 (m, 4H), 1.14(s, 3H), 0.95 (t, J = 7.6 Hz, 3H); ^{13}C NMR (100 MHz, $CDCl_3$): δ 191.22 (t, J = 31.0 Hz), 142.70, 134.84 (t, J = 2.1 Hz), 133.67, 130.08 (t, J = 4.4 Hz), 128.54, 128.38, 128.28, 125.78, 122.73 (t, J = 258.2 Hz), 43.45 (t, J = 19.5 Hz), 36.21 (t, J = 3.0 Hz), 30.61, 26.42 (t, J = 3.7 Hz), 19.47 (t, J = 4.7 Hz), 8.25; ^{19}F NMR (376 MHz, $CDCl_3$): δ -103.62 (s, 1F), -103.63 (s, 1F); IR (ATR): 2976, 1694,

1599, 1578, 1514, 1497, 1386, 1269, 1049, 713, 688; HRMS (ESI): Exact mass calcd for $C_{20}H_{22}F_2NaO$ $[M+Na]^+$: 339.1531, Found: 339.1530.

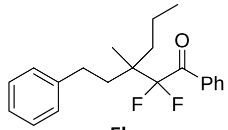

5h

The *E*/*Z* ratio of alkene **4h** used in this reaction is 1:1, and the reaction used 20 mol% of $Mg(ClO_4)_2{\cdot}6H_2O$ and 3.0 equiv. of **2a**. Column chromatography (PE/CH_2Cl_2 = 1:0 to 20:1, v/v) afforded product **5h** as colorless oil (62.3 mg, 63% yield). ^1H NMR (400 MHz, $CDCl_3$): δ 8.06 (d, *J* = 7.6 Hz, 2H), 7.58 (t, *J* = 7.2 Hz, 1H), 7.45 (t, *J* = 7.6 Hz, 2H), 7.28-7.24 (m, 2H), 7.18-7.13 (m, 3H), 2.67-2.63 (m, 2H), 1.91-1.76 (m, 2H), 1.69-1.61 (m, 1H), 1.59-1.52 (m, 1H), 1.43-1.34 (m, 2H), 1.15 (s, 3H), 0.89 (t, *J* = 7.2 Hz, 3H); ^{13}C NMR (100 MHz, $CDCl_3$): δ 191.15 (t, *J* = 31.2 Hz), 142.64, 134.84, 133.63, 130.05 (t, *J* = 4.4 Hz), 128.50, 128.36, 128.26, 125.77, 122.57 (t, *J* = 258.3 Hz), 43.48 (t, *J* = 19.7 Hz), 36.73 (t, *J* = 3.0 Hz), 36.40 (t, *J* = 3.4 Hz), 30.62 (t, *J* = 2.1 Hz), 19.96 (t, *J* = 4.7 Hz), 17.04 (t, *J* = 1.5 Hz), 14.87; ^{19}F NMR (376 MHz, $CDCl_3$): δ -103.55 (s, 1F), -103.56 (s, 1F); IR (ATR): 2968, 1694, 1597, 1578, 1497, 1449, 1385, 1275, 1128, 1099, 980, 716; HRMS (ESI): Exact mass calcd for $C_{21}H_{24}F_2NaO$ $[M+Na]^+$: 353.1687, Found: 353.1684.

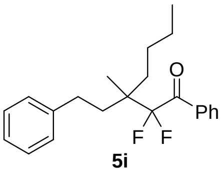

5i

The *E*/*Z* ratio of alkene **4i** used in this reaction is 1:1, and the reaction used 20 mol% of $Mg(ClO_4)_2{\cdot}6H_2O$ and 3.0 equiv. of **2a**. Column chromatography (PE/CH_2Cl_2 = 1:0 to 20:1, v/v) afforded product **5i** as colorless oil (48.1 mg, 47% yield). ^1H NMR (400 MHz, $CDCl_3$): δ 8.06 (d, *J* = 8.0 Hz, 2H), 7.59 (t, *J* = 7.2 Hz, 1H), 7.46 (t, *J* = 7.6 Hz, 2H), 7.28-7.22 (m, 2H), 7.18-7.13 (m, 3H), 2.67-2.63 (m, 2H), 1.90-1.78 (m, 2H), 1.71-1.63 (m, 1H), 1.61-1.53 (m, 1H), 1.36-1.25 (m, 4H), 1.15 (s, 3H), 0.89 (t, *J* = 6.8 Hz, 3H); ^{13}C NMR (100 MHz, $CDCl_3$): δ 191.21 (t, *J* = 31.1 Hz), 142.68, 134.89, 133.63, 130.06 (t, *J* = 4.4 Hz), 128.51, 128.38, 128.28, 125.78, 122.62 (t, *J* = 258.4 Hz), 43.39 (t, *J* = 19.6 Hz), 36.68 (t, *J* = 3.1 Hz), 33.76 (t, *J* = 3.6 Hz), 30.64 (t, *J* = 2.1 Hz), 25.88, 23.50, 19.97 (t, *J* = 4.7 Hz), 13.98; ^{19}F NMR (376 MHz, $CDCl_3$): δ -103.58 (s, 1F), -103.59 (s, 1F); IR (ATR): 2959, 1694, 1597, 1578, 1497, 1449, 1385, 1275, 1128, 1047, 899, 714; HRMS (ESI): Exact mass calcd for $C_{22}H_{26}F_2NaO$ $[M+Na]^+$: 367.1844, Found: 367.1829.

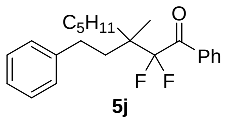

5j

The *E*/*Z* ratio of alkene **4j** used in this reaction is 1:1. Column chromatography (PE/CH_2Cl_2 = 1:0 to 20:1, v/v) afforded product **5j** as colorless oil (37.3 mg, 35% yield). ^1H NMR (400 MHz, $CDCl_3$): δ 8.06 (d, *J* = 7.6 Hz, 2H), 7.61-7.57 (m, 1H), 7.48-7.44 (m, 2H), 7.28-7.23 (m, 2H), 7.19-7.13 (m, 3H), 2.71-2.59 (m, 2H), 1.90-1.76 (m, 2H), 1.70-1.52 (m, 2H), 1.39-1.21 (m, 6H), 1.15 (s, 3H), 0.88 (t, *J* = 7.2 Hz, 3H); ^{13}C NMR (100 MHz, $CDCl_3$): δ 191.22 (t, *J* = 31.2 Hz), 142.68, 134.90 (t, *J* = 2.1 Hz), 133.63, 130.06 (t, *J* = 4.3 Hz), 128.51, 128.38, 128.28, 125.78, 122.62 (t, *J* = 258.4 Hz), 43.43 (t, *J* = 19.6 Hz), 36.68 (t, *J* = 3.2 Hz), 34.00 (t, *J* = 3.4 Hz), 32.65, 30.65 (t, *J* = 2.0 Hz), 23.35, 22.52, 19.98 (t, *J* = 4.7 Hz), 14.02; ^{19}F NMR (376 MHz, $CDCl_3$): δ -103.60 (s, 1F), 103.62 (s, 1F); IR (ATR): 2872, 1694, 1597, 1578, 1497, 1449, 1273, 1128, 1049, 901, 716; HRMS (ESI): Exact mass calcd for $C_{23}H_{28}F_2NaO$ $[M+Na]^+$: 381.2000, Found: 381.2002.

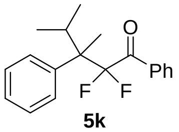

5k

Column chromatography (PE/CH_2Cl_2 = 1:0 to 20:1, v/v) afforded product **5k** as colorless oil (39.9 mg, 44% yield). ^1H NMR (400 MHz, $CDCl_3$): δ 7.47-7.45 (m, 2H), 7.43-7.39 (m, 1H), 7.34-7.32 (m, 2H), 7.24-7.20 (m, 2H), 7.16-7.07 (m, 3H), 2.87-2.77 (m, 1H), 1.57 (d, *J* = 2.0 Hz, 3H), 1.26 (dd, *J* = 6.8, 2.4 Hz, 3H), 0.59 (d, *J* = 7.2 Hz, 3H); ^{13}C NMR (100 MHz, $CDCl_3$): δ 191.86 (t, *J* = 32.1 Hz), 140.37 (d, *J* = 5.9 Hz), 132.69, 129.27 (dd, *J* = 5.1, 3.0 Hz), 128.12, 128.01, 127.79, 127.08, 121.72 (t, *J* = 263.0 Hz), 50.99 (t, *J* = 19.6 Hz), 32.30 (d, *J* = 6.4 Hz), 18.69 (d, *J* = 2.8 Hz), 18.27, 11.94 (t, *J* = 5.2 Hz); ^{19}F NMR (376 MHz, $CDCl_3$): δ -95.69 (d, *J* = 261.3 Hz, 1F), -106.88 (d, *J* = 262.1 Hz, 1F); IR (ATR): 2974, 1726, 1611, 1514, 1477, 1395, 1242, 1134,1049, 1043, 717, 687; HRMS (ESI): Exact mass calcd for $C_{19}H_{20}F_2NaO$ $[M+Na]^+$: 325.1374, Found: 325.1378.

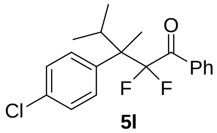

5l

Column chromatography (PE/CH_2Cl_2 = 1:0 to 20:1, v/v) afforded product **5l** as colorless oil (53.0 mg, 52% yield). ^1H NMR (400 MHz, $CDCl_3$): δ 7.54 (d, *J* = 7.6 Hz, 2H), 7.49-7.44 (m, 1H), 7.30-7.27 (m, 3H), 7.25-7.24 (m, 1H), 7.13-7.10 (m, 2H), 2.79-2.69 (m, 1H), 1.54 (d, *J* = 1.6 Hz, 3H), 1.24 (dd, *J* = 6.8, 2.8 Hz, 3H), 0.58 (d, *J* = 7.2 Hz, 3H); ^{13}C NMR (125 MHz, $CDCl_3$): δ 191.31 (dd, *J* = 32.5, 29.5 Hz), 139.27 (d, *J* = 6.3 Hz), 134.71 (d, *J* = 2.6 Hz), 133.06, 133.00, 129.39 (d, *J* = 1.4 Hz), 129.31 (dd, *J* = 5.3, 3.1 Hz), 128.19, 128.00, 121.50 (t, *J* = 263.6 Hz), 50.79 (t, *J* = 19.6 Hz), 32.59 (d, *J* = 6.6 Hz), 18.69 (d, *J* = 5.0 Hz), 18.20, 12.12 (t, *J* = 5.1 Hz); ^{19}F NMR (376 MHz, $CDCl_3$): δ -95.83 (d, *J* = 267.0 Hz, 1F), -106.77 (d, *J* =

267.0 Hz, 1F); IR (ATR): 2974, 1701, 1597, 1578, 1495, 1449, 1271, 1184, 1096, 1011, 916, 887, 710; HRMS (ESI): Exact mass calcd for $C_{19}H_{19}ClF_2NaO$ [M+Na]$^+$: 359.0985, Found: 359.0978.

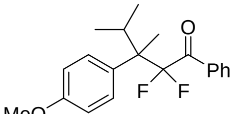
5m

Column chromatography (PE/CH_2Cl_2 = 6:1, v/v) afforded product **5m** as colorless oil (69.8 mg, 70% yield). ^{1}H NMR (400 MHz, $CDCl_3$): δ 7.38 (d, *J* = 7.6 Hz, 2H), 7.31 (t, *J* = 7.2 Hz, 1H), 7.16-7.12 (m, 4H), 6.57 (d, *J* = 9.2 Hz, 2H), 3.60 (s, 3H), 2.75-2.65 (m, 1H), 1.44 (s, 3H), 1.16 (dd, *J* = 6.8, 2.8 Hz, 3H), 0.51 (d, *J* = 6.8 Hz, 3H); ^{13}C NMR (100 MHz, $CDCl_3$): δ 192.08 (dd, *J* = 32.1, 29.2 Hz), 158.46, 135.09 (d, *J* = 2.5 Hz), 132.56, 132.05 (d, *J* = 6.1 Hz), 129.26, 129.21 (t, *J* = 2.6 Hz), 127.73, 121.83 (t, *J* = 263.8 Hz), 113.40, 55.04 (d, *J* = 2.2 Hz), 50.27 (t, *J* = 19.8 Hz), 32.13 (d, *J* = 6.4 Hz), 18.59 (dd, *J* = 5.3, 1.8 Hz), 18.20, 12.04 (t, *J* = 5.1 Hz); ^{19}F NMR (376 MHz, $CDCl_3$): δ -95.93 (d, *J* = 258.3 Hz, 1F), -107.94 (d, *J* = 258.3 Hz, 1F); IR (ATR): 2970, 1611, 1580, 1541, 1446, 1296, 1157, 1007, 937, 837, 781; GC-MS (EI): 332 (M^+, 5), 289 (3), 177 (33), 121 (7), 105 (100), 77 (18), 69(3); HRMS (EI): Exact mass calcd for $C_{20}H_{22}O_2F_2$ [M]$^+$: 322.1588, Found: 322.1590.

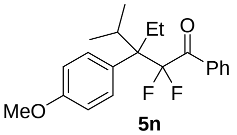
5n

Column chromatography (PE/CH_2Cl_2 = 10:1, v/v) afforded product **5n** as colorless oil (76.8 mg, 74% yield). ^{1}H NMR (400 MHz, $CDCl_3$): δ 7.49 (d, *J* = 7.6 Hz, 2H), 7.42-7.38 (m, 1H), 7.25-7.19 (m, 4H), 6.70 (d, *J* = 9.2 Hz, 2H), 3.71 (s, 3H), 2.91-2.80 (m, 1H), 2.37-2.19 (m, 2H), 1.26 (d, *J* = 6.8 Hz, 3H), 1.01 (t, *J* = 7.6 Hz, 3H), 0.86 (d, *J* = 6.8 Hz, 3H); ^{13}C NMR (100 MHz, $CDCl_3$): δ 191.57 (t, *J* = 31.3 Hz), 158.31, 134.66 (t, *J* = 2.1 Hz), 132.73, 130.69, 129.99 (dd, *J* = 4.5, 1.1 Hz), 129.64 (t, *J* = 4.2 Hz), 127.77, 122.02 (t, *J* = 264.2 Hz), 113.29, 55.08 (d, *J* = 2.5 Hz), 53.78 (t, *J* = 18.5 Hz), 31.02 (d, *J* = 5.2 Hz), 20.44, 20.19 (t, *J* = 3.7 Hz), 18.96 (t, *J* = 3.5 Hz), 10.32; ^{19}F NMR (376 MHz, $CDCl_3$): δ -96.81 (d, *J* = 266.2 Hz, 1F), -100.60 (d, *J* = 266.2 Hz, 1F); IR (ATR): 2974, 1599, 1580, 1514, 1449, 1288, 1141, 1001, 842, 775; HRMS (ESI): Exact mass calcd for $C_{21}H_{24}F_2NaO_2$ [M+Na]$^+$: 369.1637, Found: 369.1635.

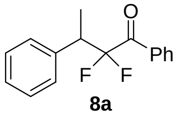
8a

Column chromatography (PE) afforded product **8a** as colorless oil (38.3 mg, 49% yield). ^{1}H NMR (400 MHz, $CDCl_3$): δ 7.92 (d, *J* = 8.0 Hz, 2H), 7.60-7.55 (m, 1H), 7.42 (t, *J* = 7.6 Hz, 2H), 7.29-7.24 (m, 5H), 3.81-3.67 (m, 1H), 1.50 (d, *J* = 7.2 Hz, 3H); ^{13}C NMR (100 MHz, $CDCl_3$): δ 190.22 (t, *J* = 29.9 Hz), 137.25 (t, *J* = 4.2 Hz), 133.94, 132.86 (t, *J* = 2.1 Hz), 129.85 (t, *J* = 3.5 Hz), 129.13, 128.50, 128.40, 127.67, 119.49 (t, *J* = 255.8 Hz), 43.91 (td, *J* = 21.8, 2.4 Hz), 14.30 (t, *J* = 4.7 Hz); ^{19}F NMR (376 MHz, $CDCl_3$): δ -104.83 (d, *J* = 272.2 Hz, 1F),

-106.20 (d, *J* = 272.2 Hz, 1F); IR (ATR): 2970, 1595, 1495, 1449, 1229, 1190, 1001, 974, 789, 625; GC-MS (EI): 260 (M^+, 0.4), 240 (27), 127 (1.0), 106 (8.0), 105 (100),77 (32), 51(6); HRMS (EI): Exact mass calcd for $C_{16}H_{14}OF_2$ $[M]^+$: 260.1013, Found: 260.1016.

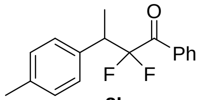
8b

Column chromatography (PE) afforded product **8b** as colorless oil (27.9 mg, 34% yield). ^{1}H NMR (400 MHz, $CDCl_3$): δ 7.93 (d, *J* = 7.2 Hz, 2H), 7.60-7.56 (m, 1H), 7.45-7.41 (m, 2H), 7.16 (d, *J* = 8.0 Hz, 2H), 7.09 (d, *J* = 8.0 Hz, 2H), 3.77-3.63 (m, 1H), 2.30 (s, 3H), 1.47 (d, *J* = 7.2 Hz, 3H); ^{13}C NMR (100 MHz, $CDCl_3$): δ 190.28 (t, *J* = 30.0 Hz), 137.35, 134.21 (dd, *J* = 4.3, 1.0 Hz), 133.89, 132.95 (t, *J* = 2.0 Hz), 129.88 (t, *J* = 3.5 Hz), 129.12, 128.98, 128.50, 119.54 (t, *J* = 255.7 Hz), 43.55 (t, *J* = 21.6 Hz), 21.03 (d, *J* = 2.0 Hz), 14.36 (t, *J* = 4.6 Hz); ^{19}F NMR (376 MHz, $CDCl_3$): δ -104.96 (d, *J* = 271.1 Hz, 1F), -106.30 (d, *J* = 271.5 Hz, 1F); IR (ATR): 2976, 1599, 1580, 1516, 1449, 1381, 1207, 1138, 1001, 980, 819, 714; HRMS (ESI): Exact mass calcd for $C_{17}H_{16}F_2NaO$ $[M+Na]^+$: 297.1061, Found: 297.1059.

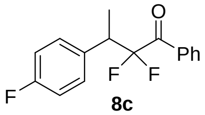
8c

Column chromatography (PE) afforded product **8c** as colorless oil (41.9 mg, 50% yield). ^{1}H NMR (400 MHz, $CDCl_3$): δ 7.94 (d, *J* = 7.2 Hz, 2H), 7.61-7.57 (m, 1H), 7.44 (t, *J* = 7.6 Hz, 2H), 7.27-7.23 (m, 2H), 7.00-6.94 (m, 2H), 3.81-3.67 (m, 1H), 1.47 (d, *J* = 7.2 Hz, 3H); ^{13}C NMR (100 MHz, $CDCl_3$): δ 189.99 (t, *J* = 30.1 Hz), 162.25 (d, *J* = 244.8 Hz), 134.06, 133.06, 132.78 (t, *J* = 2.2 Hz), 130.71 (d, *J* = 8.0 Hz), 129.84 (t, *J* = 3.5 Hz), 128.58, 119.31 (t, *J* = 256.0 Hz), 115.28 (d, *J* = 21.2 Hz), 43.12 (t, *J* = 22.1 Hz), 14.43 (t, *J* = 4.5 Hz); ^{19}F NMR (376 MHz, $CDCl_3$): δ -104.97 (d, *J* = 274.5 Hz, 1F), -106.32 (d, *J* = 274.1 Hz, 1F), -114.79 (s, 1F); IR (ATR): 2976, 1599, 1580, 1510, 1449, 1325, 1207, 1107, 1001, 937, 885, 787; HRMS (ESI): Exact mass calcd for $C_{16}H_{13}F_3NaO$ $[M+Na]^+$: 301.0811, Found: 301.0812.

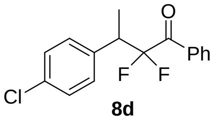
8d

Column chromatography (PE) afforded product **8d** as colorless oil (55.9 mg, 63% yield). ^{1}H NMR (400 MHz, $CDCl_3$): δ 7.95 (d, *J* = 7.6 Hz, 2H), 7.59 (t, *J* = 7.6 Hz, 1H), 7.44 (t, *J* = 7.6 Hz, 2H), 7.27-7.21 (m, 4H), 3.80-3.67 (m, 1H), 1.47 (d, *J* = 7.2 Hz, 3H); ^{13}C NMR (125 MHz, $CDCl_3$): δ 189.70 (t, *J* = 30.0 Hz), 135.82 (t, *J* = 2.9 Hz), 134.17, 133.53, 132.51 (t, *J* = 2.3 Hz), 130.46, 129.87 (t, *J* = 3.4 Hz), 128.61, 128.55, 119.14 (t, *J* = 256.1 Hz), 43.14 (t, *J* = 22.1 Hz), 14.35 (t, *J* = 4.6 Hz); ^{19}F NMR (376 MHz, $CDCl_3$): δ -104.88 (d, *J* = 276.7 Hz, 1F), -105.80 (d, *J* = 276.7 Hz, 1F); IR (ATR): 1599, 1578, 1491, 1449, 1279, 1136, 1086, 984, 826,

773; GC-MS (EI): 294 [M(^{35}Cl)$^+$, 1.2], 274 (18), 139 (8), 105 (100), 77 (32), 51 (6); HRMS (EI): Exact mass calcd for $C_{16}H_{13}O^{35}ClF_2$ [M]$^+$: 294.0623, Found: 294.0625.

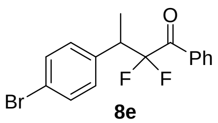

8e

Column chromatography (PE) afforded product **8e** as white solid (36.7 mg, 36% yield). m.p. 55-57 ^{o}C; ^{1}H NMR (400 MHz, $CDCl_3$): δ 7.96 (d, *J* = 7.6 Hz, 2H), 7.61 (t, *J* = 7.2 Hz, 1H), 7.47-7.41 (m, 4H), 7.17 (d, *J* = 8.0 Hz, 2H), 3.79-3.66 (m, 1H), 1.47 (d, *J* = 7.2 Hz, 3H); ^{13}C NMR (100 MHz, $CDCl_3$): δ 189.68 (t, *J* = 30.1 Hz), 136.41 (t, *J* = 3.1 Hz), 134.17, 132.56 (t, *J* = 2.2 Hz), 131.52, 130.82, 129.88 (t, *J* = 3.5 Hz), 128.61, 121.72, 119.11 (t, *J* = 256.1 Hz), 43.22 (t, *J* = 21.6 Hz), 14.33 (t, *J* = 4.3 Hz); ^{19}F NMR (376 MHz, $CDCl_3$): δ -104.92 (d, *J* = 277.1 Hz, 1F), -105.75 (d, *J* = 277.1 Hz, 1F); IR (ATR): 2974, 1740, 1599, 1408, 1263, 1136, 1055, 984, 748; GC-MS (EI): 340 [M(^{81}Br)$^+$, 1], 338 [M(^{79}Br)$^+$, 1], 318 (3), 185 (3), 105 (100), 77 (28), 51 (5); HRMS (EI): Exact mass calcd for $C_{16}H_{13}OF_2{}^{79}Br$ [M]$^+$: 338.0118, Found: 338.0121.

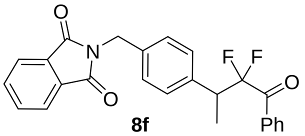

8f

The reaction used 20 mol% of $Mg(ClO_4)_2{\cdot}6H_2O$ and 3.0 equiv. of **2a**. Column chromatography (PE/EtOAc = 10:1, v/v) afforded product **8f** as white solid (76.9 mg, 61% yield). m.p. 81-83 ^{o}C; ^{1}H NMR (400 MHz, $CDCl_3$): δ 7.90 (d, *J* = 8.0 Hz, 2H), 7.85-7.82 (m, 2H), 7.71-7.68 (m, 2H), 7.54-7.49 (m, 1H), 7.40-7.35 (m, 4H), 7.26-7.23 (m, 2H), 4.80 (s, 2H), 3.77-3.64 (m, 1H), 1.45 (d, *J* = 7.2 Hz, 3H); ^{13}C NMR (100 MHz, $CDCl_3$): δ 190.00 (t, *J* = 30.0 Hz), 167.91, 136.92 (t, *J* = 3.0 Hz), 135.77, 133.95, 133.90, 132.72 (t, *J* = 2.0 Hz), 132.04, 129.83 (t, *J* = 3.5 Hz), 129.45, 128.69, 128.46, 123.29, 119.36 (t, *J* = 256.0 Hz), 43.49 (t, *J* = 22.3 Hz), 41.13, 14.26 (t, *J* = 4.6 Hz); ^{19}F NMR (376 MHz, $CDCl_3$) δ -104.99 (dd, *J* = 273.4, 3.8 Hz, 1F), -105.84 (dd, *J* = 273.7, 2.3 Hz, 1F); IR (ATR): 1711, 1597, 1578, 1516, 1449, 1393, 1055, 937, 887, 712; HRMS (ESI): Exact mass calcd for $C_{25}H_{19}F_2NNaO_3$ [M+Na]$^+$: 442.1225, Found: 442.1225.

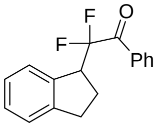

8g

Column chromatography (PE/CH_2Cl_2 = 1:0 to 20:1, v/v) afforded product **8g** as colorless oil (37.1 mg, 45% yield). ^{1}H NMR (400 MHz, $CDCl_3$): δ 8.08 (d, *J* = 7.2 Hz, 2H), 7.64-7.59 (m, 1H), 7.47 (t, *J* = 8.0 Hz, 2H), 7.35 (d, *J* = 7.6 Hz, 1H), 7.26-7.14 (m, 3H), 4.21-4.09 (m, 1H), 3.12-3.04 (m, 1H), 2.94-2.87 (m, 1H), 2.40-2.30 (m, 1H), 2.27-2.19 (m, 1H); ^{13}C NMR (100 MHz, $CDCl_3$): δ 189.90 (t, *J* = 31.2 Hz), 145.51, 137.95 (t, *J* = 3.6 Hz), 134.14, 132.66 (t, *J* = 2.5 Hz), 130.12 (t, *J* = 3.4 Hz), 128.61, 127.95, 126.28, 126.20 (d, *J* = 2.4 Hz), 124.70, 120.23 (t, *J* = 254.3 Hz), 48.64 (dd, *J* = 23.3, 21.1 Hz), 31.62, 26.03 (dd, *J* = 5.3, 3.2 Hz); ^{19}F NMR (376

MHz, $CDCl_3$): δ -101.65 (d, *J* = 285.0 Hz, 1F), -106.30 (d, *J* = 285.0 Hz, 1F); IR (ATR): 2976, 1699, 1597, 1580, 1449, 1277, 1121, 1057, 1003, 922, 814, 754; HRMS (ESI): Exact mass calcd for $C_{17}H_{14}F_2NaO$ $[M+Na]^+$: 295.0905, Found: 295.0898.

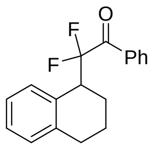

8h

Column chromatography (PE/CH_2Cl_2 = 1:0 to 20:1, v/v) afforded product **8h** as colorless oil (70.9 mg, 83% yield). ^{1}H NMR (400 MHz, $CDCl_3$): δ 8.01 (d, *J* = 7.6 Hz, 2H), 7.61-7.57 (m, 1H), 7.45 (t, *J* = 8.0 Hz, 2H), 7.28 (d, *J* = 7.6 Hz, 1H), 7.20-7.16 (m, 1H), 7.13-7.08 (m, 2H), 3.91-3.80 (m, 1H), 2.86-2.69 (m, 2H), 2.04-1.90 (m, 3H), 1.69-1.62 (m, 1H); ^{13}C NMR (100 MHz, $CDCl_3$): δ 190.56 (t, *J* = 30.0 Hz), 139.42, 134.02, 133.03, 130.63 (d, *J* = 3.2 Hz), 130.52 (dd, *J* = 2.8, 1.4 Hz), 129.97 (t, *J* = 3.6 Hz), 129.29, 128.60, 127.26, 125.46, 120.34 (dd, *J* = 257.7, 254.4 Hz), 41.53 (t, *J* = 22.2 Hz), 29.01, 23.09 (t, *J* = 3.4 Hz), 19.89; ^{19}F NMR (376 MHz, $CDCl_3$): δ -96.76 (d, *J* = 271.8 Hz, 1F), -103.55 (d, *J* = 271.5 Hz, 1F); IR (ATR): 2879, 1701, 1597, 1580, 1491, 1449, 1267, 1148, 1065, 912, 745; HRMS (ESI): Exact mass calcd for $C_{18}H_{16}F_2NaO$ $[M+Na]^+$: 309.1061, Found: 309.1055.

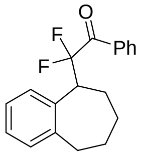

8i

Column chromatography (PE/CH_2Cl_2 = 1:0 to 20:1, v/v) afforded product **8i** as colorless oil (68.4 mg, 76% yield). ^{1}H NMR (400 MHz, $CDCl_3$): δ 8.02 (d, *J* = 8.0 Hz, 2H), 7.61-7.56 (m, 1H), 7.45 (t, *J* = 8.0 Hz, 2H), 7.18-7.04 (m, 4H), 4.03-3.91 (m, 1H), 2.96-2.80 (m, 2H), 2.06-1.90 (m, 2H), 1.78-1.56 (m, 4H); ^{13}C NMR (100 MHz, $CDCl_3$): δ 190.29 (t, *J* = 30.3 Hz), 142.76, 136.73, 133.94, 132.96, 130.03, 129.92 (t, *J* = 3.3 Hz), 128.59, 127.11, 126.01, 120.81 (t, *J* = 255.9 Hz), 47.61 (t, *J* = 20.0 Hz), 35.09, 28.03, 27.37, 26.79; ^{19}F NMR (376 MHz, $CDCl_3$): δ -101.52(br, 2F); IR (ATR): 2974, 1701, 1597, 1578, 1493, 1449, 1275, 1180, 1043, 918, 853, 748; HRMS (ESI): Exact mass calcd for $C_{19}H_{18}F_2NaO$ $[M+Na]^+$: 323.1218, Found: 323.1211.

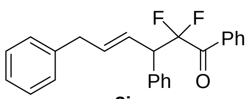

8j

Column chromatography (PE/CH_2Cl_2 = 20:1 to 6:1, v/v) afforded product **8j** as colorless oil (98.9 mg, 91% yield). ^{1}H NMR (400 MHz, $CDCl_3$): δ 7.91 (d, *J* = 7.6 Hz, 2H), 7.60-7.56 (m, 1H), 7.43 (t, *J* = 7.6 Hz, 2H), 7.32-7.23 (m, 7H), 7.20-7.16 (m, 1H), 7.08 (d, *J* = 7.2 Hz, 2H), 5.93-5.87 (m, 1H), 5.78-5.71 (m, 1H), 4.34-4.24 (m, 1H), 3.41-3.31 (m, 2H); ^{13}C NMR (125 MHz, $CDCl_3$): δ 190.15 (t, *J* = 29.1 Hz), 139.57, 135.56 (d, *J* = 3.3 Hz), 135.49, 134.07, 133.00, 129.89 (t, *J* = 3.5 Hz), 129.57, 128.66, 128.57, 128.48, 127.86, 126.22, 125.27 (dd, *J* = 5.5, 2.8 Hz), 118.73 (t, *J* = 256.9 Hz), 53.22 (t, *J* = 21.9 Hz), 39.05; ^{19}F NMR (376

MHz, $CDCl_3$): δ -102.85 (d, *J* = 270.3 Hz, 1F), -104.44 (d, *J* = 270.0 Hz, 1F); IR (ATR): 2970, 1738, 1597, 1495, 1449, 1366, 1217, 1171, 1049, 920, 716; GC-MS (EI): 271 (17), 251 (47), 206 (53), 129 (24), 117 (16), 105 (74), 91 (100), 77 (40), 51 (5); HRMS (EI): Exact mass calcd for $C_{24}H_{20}OF_2$ $[M]^+$: 362.1482, Found: 362.1486.

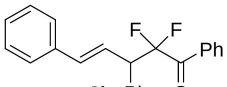

8k

Column chromatography (PE/CH_2Cl_2 = 20:1 to 6:1, v/v) afforded product **8k** as white solid (97.0 mg, 93% yield). m.p. 82-84 °C; ^{1}H NMR (400 MHz, $CDCl_3$): δ 7.95 (d, *J* = 7.2 Hz, 2H), 7.62-7.57 (m, 1H), 7.46-7.42 (m, 2H), 7.39-7.27 (m, 9H), 7.24-7.20 (m, 1H), 6.53 (d, *J* = 4.0 Hz, 2H), 4.47 (tt, *J* = 16.4, 4.0 Hz, 1H); ^{13}C NMR (100 MHz, $CDCl_3$): δ 189.83 (t, *J* = 29.6 Hz), 136.43, 135.36 (t, *J* = 2.2 Hz), 135.24, 133.97, 132.84 (t, *J* = 2.1 Hz), 129.78 (t, *J* = 3.4 Hz), 129.55, 128.62, 128.53, 128.46, 127.85, 127.83, 126.44, 123.52 (t, *J* = 4.0 Hz), 118.65 (t, *J* = 257.5 Hz), 53.29 (t, *J* = 21.8 Hz); ^{19}F NMR (376 MHz, $CDCl_3$): δ -102.55 (d, *J* = 273.7 Hz, 1F), -103.39 (d, *J* = 273.7 Hz, 1F); IR (ATR): 2976, 1695, 1599, 1580, 1449, 1387, 1275, 1186, 1049, 907, 748; HRMS (ESI): Exact mass calcd for $C_{23}H_{18}F_2NaO$ $[M+Na]^+$: 371.1218, Found: 371.1222.

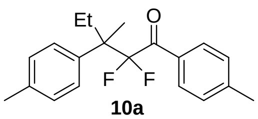

10a

Column chromatography (PE/CH_2Cl_2 = 1:0 to 20:1, v/v) afforded product **10a** as white solid (68.7 mg, 72% yield). m.p. 38-40 °C; ^{1}H NMR (400 MHz, $CDCl_3$): δ 7.50 (d, *J* = 8.0 Hz, 2H), 7.22 (d, *J* = 8.0 Hz, 2H), 7.03 (d, *J* = 7.6 Hz, 4H), 2.42-2.33 (m, 1H), 2.31 (s, 3H), 2.25 (s, 3H), 1.95-1.86 (m, 1H), 1.56 (s, 3H), 0.73 (t, *J* = 7.6 Hz, 3H); ^{13}C NMR (100 MHz, $CDCl_3$): δ 190.83 (t, *J* = 30.0 Hz), 144.09, 136.73, 135.24 (t, *J* = 2.4 Hz), 131.98, 130.01 (t, *J* = 4.1 Hz), 128.71, 128.66, 121.02 (t, *J* = 259.9 Hz), 48.00 (t, *J* = 20.3 Hz), 26.28 (t, *J* = 3.9 Hz), 21.49, 20.75, 18.20 (t, *J* = 4.2 Hz), 7.64; ^{19}F NMR (376 MHz, $CDCl_3$): δ -104.77 (d, *J* = 258.3 Hz, 1F), -105.87 (d, *J* = 257.9 Hz, 1F); IR (ATR): 2976, 1678, 1607, 1568, 1516, 1452, 1271, 1134, 1092, 905, 820, 733; HRMS (ESI): Exact mass calcd for $C_{20}H_{23}F_2O$ $[M+H]^+$: 317.1711, Found: 317.1730.

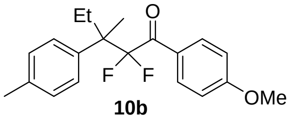

10b

Column chromatography (PE/CH_2Cl_2 = 9:1 to 6:1, v/v) afforded product **10b** as colorless oil (79.9 mg, 80% yield). ^{1}H NMR (400 MHz, $CDCl_3$): δ 7.60 (d, *J* = 8.4 Hz, 2H), 7.22 (d, *J* = 8.0 Hz, 2H), 7.03 (d, *J* = 8.0 Hz, 2H), 6.72-6.69 (m, 2H), 3.80 (s, 3H), 2.42-2.33 (m, 1H), 2.26 (s, 3H), 1.95-1.86 (m, 1H), 1.56 (s, 3H), 0.74 (t, *J* = 7.6 Hz, 3H); ^{13}C NMR (100 MHz, $CDCl_3$): δ 189.43 (t, *J* = 29.7 Hz), 163.56, 136.69, 135.31, 132.47 (t, *J*

= 4.4 Hz), 128.69, 127.33, 121.20 (t, *J* = 259.5 Hz), 113.21, 55.32, 48.03 (t, *J* = 20.3 Hz), 26.22 (t, *J* = 4.0 Hz), 20.76, 18.21 (t, *J* = 4.4 Hz), 7.66; ^{19}F NMR (376 MHz, $CDCl_3$): δ -104.64 (d, *J* = 257.2 Hz, 1F), -105.68 (d, *J* = 256.4 Hz, 1F); IR (ATR): 2976, 1682, 1597, 1574, 1508, 1460, 1261, 1169, 1096, 907, 800, 737; HRMS (ESI): Exact mass calcd for $C_{20}H_{22}F_2NaO_2$ $[M+Na]^+$: 355.1480, Found: 355.1474.

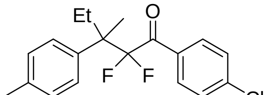

10c

Column chromatography (PE/CH_2Cl_2 = 1:0 to 20:1, v/v) afforded product **10c** as colorless oil (79.5 mg, 79% yield). ^{1}H NMR (400 MHz, $CDCl_3$): δ 7.45 (d, *J* = 8.4 Hz, 2H), 7.23-7.16 (m, 4H), 7.01 (d, *J* = 8.0 Hz, 2H), 2.41-2.32 (m, 1H), 2.25 (s, 3H), 1.96-1.87 (m, 1H), 1.56 (s, 3H), 0.75 (t, *J* = 7.6 Hz, 3H); ^{13}C NMR (100 MHz, $CDCl_3$): δ 190.34 (t, *J* = 30.6 Hz), 139.69, 137.11, 134.88 (t, *J* = 2.5 Hz), 132.76 (t, *J* = 1.8 Hz), 131.19 (t, *J* = 4.3 Hz), 128.87, 128.67, 128.21, 120.98 (t, *J* = 260.0 Hz), 48.03 (t, *J* = 20.2 Hz), 26.01 (dd, *J* = 4.9, 3.1 Hz), 20.75, 17.99 (t, *J* = 4.2 Hz), 7.60; ^{19}F NMR (376 MHz, $CDCl_3$): δ -105.15 (d, *J* = 257.2 Hz, 1F), -106.74 (d, *J* = 256.8 Hz, 1F); IR (ATR): 2976, 1694, 1587, 1487, 1385, 1271, 1092, 907, 822, 797; HRMS (ESI): Exact mass calcd for $C_{19}H_{19}ClF_2NaO$ $[M+Na]^+$: 359.0985, Found: 359.0982.

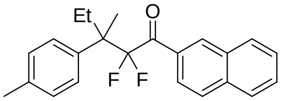

10d

Column chromatography (PE/CH_2Cl_2 = 20:1 to 10:1, v/v) afforded product **10d** as white solid (74.8 mg, 85% yield). m.p. 76-78 °C; ^{1}H NMR (400 MHz, $CDCl_3$): δ 7.83 (s, 1H), 7.78-7.76 (m, 1H), 7.73-7.66 (m, 3H), 7.56-7.52 (m, 1H), 7.49-7.45 (m, 1H), 7.26-7.24 (m, 2H), 6.98-6.96 (m, 2H), 2.47-2.38 (m, 1H), 2.12 (s, 3H), 2.02-1.93 (m, 1H), 1.61 (s, 3H), 0.78 (t, *J* = 7.2 Hz, 3H); ^{13}C NMR (100 MHz, $CDCl_3$): δ 191.34 (t, *J* = 29.8 Hz), 137.10, 135.26, 135.18 (t, *J* = 2.4 Hz), 132.65 (t, *J* = 5.5 Hz), 131.98, 131.59, 129.98, 128.88, 128.86, 128.78, 127.61, 127.46, 126.44, 124.84 (t, *J* = 2.5 Hz), 121.30 (t, *J* = 260.1 Hz), 48.21 (t, *J* = 20.5 Hz), 26.02 (t, *J* = 3.6 Hz), 20.67, 18.16 (t, *J* = 4.2 Hz), 7.71; ^{19}F NMR (376 MHz, $CDCl_3$): δ -104.80 (d, *J* = 254.6 Hz, 1F), -106.14 (d, *J* = 254.6 Hz, 1F); IR (ATR): 2970, 1746, 1628, 1354, 1256, 1136, 984, 876, 789; HRMS (ESI): Exact mass calcd for $C_{23}H_{22}F_2NaO$ $[M+Na]^+$: 375.1531, Found: 375.1517.

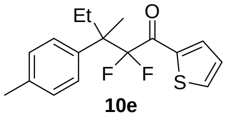

10e

Column chromatography (PE/CH_2Cl_2 = 20:1 to 10:1, v/v) afforded product **10e** as colorless oil (80.5 mg, 87% yield). ^{1}H NMR (400 MHz, $CDCl_3$): δ 7.56-7.55 (m, 1H), 7.33-7.32 (m, 1H), 7.22 (d, *J* = 8.0 Hz, 2H), 7.01 (d, *J* = 8.0 Hz, 2H), 6.88 (dd, *J* = 4.8, 4.4 Hz, 1H), 2.44-2.35 (m, 1H), 2.24 (s, 3H), 1.93-1.84 (m, 1H), 1.56 (s, 3H), 0.74 (t,

J = 7.2 Hz, 3H); ^{13}C NMR (100 MHz, $CDCl_3$): δ 183.89 (t, J = 31.2 Hz), 140.55 (t, J = 1.9 Hz), 136.84, 135.45, 135.39 (t, J = 6.0 Hz), 134.83 (t, J = 2.3 Hz), 128.66, 128.60, 128.13, 120.64 (t, J = 259.1 Hz), 47.96 (t, J = 20.4 Hz), 25.99 (t, J = 3.8 Hz), 20.74, 18.08 (t, J = 4.1 Hz), 7.63; ^{19}F NMR (376 MHz, $CDCl_3$): δ -107.02 (d, J = 250.4 Hz, 1F), -108.27 (d, J = 249.7 Hz, 1F); IR (ATR): 2974, 1661, 1514, 1410, 1356, 1285, 1175, 1065, 837, 739, 698; HRMS (ESI): Exact mass calcd for $C_{17}H_{18}OSF_2Na$ $[M+Na]^+$: 331.0944, Found: 331.0941.

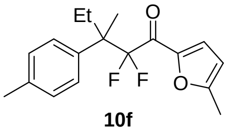

10f

Column chromatography (PE/CH_2Cl_2 = 10:1, v/v) afforded product **10f** as colorless oil (70.6 mg, 77% yield). ^{1}H NMR (400 MHz, $CDCl_3$): δ 7.22 (d, J = 8.0 Hz, 2H), 7.04 (d, J = 8.0 Hz, 2H), 6.70-6.68 (m, 1H), 5.96-5.95 (m, 1H), 2.42-2.35 (m, 1H), 2.32 (s, 3H), 2.27 (s, 3H), 1.92-1.83 (m, 1H), 1.55 (s, 3H), 0.74 (t, J = 7.6 Hz, 3H); ^{13}C NMR (100 MHz, $CDCl_3$): δ 178.03 (t, J = 31.2 Hz), 159.72, 148.72, 136.78, 135.01 (t, J = 2.5 Hz), 128.64, 128.60, 124.68 (t, J = 6.8 Hz), 120.26 (t, J = 258.2 Hz), 109.35, 47.99 (t, J = 20.6 Hz), 25.88 (t, J = 3.8 Hz), 20.80, 18.04 (t, J = 4.2 Hz), 13.96, 7.66; ^{19}F NMR (376 MHz, $CDCl_3$): δ -108.93 (d, J = 250.4 Hz, 1F), -110.22 (d, J = 251.2 Hz, 1F); IR (ATR): 1662, 1456, 1304, 1217, 1175, 1040, 974, 851, 733; HRMS (ESI): Exact mass calcd for $C_{18}H_{20}F_2NaO_2$ $[M+Na]^+$: 329.1324, Found: 329.1303.

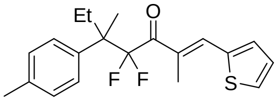

10g

Column chromatography (PE/CH_2Cl_2 = 10:1, v/v) afforded product **10g** as light yellow oil (74.0 mg, 71% yield). ^{1}H NMR (400 MHz, $CDCl_3$): δ 7.50 (d, J = 4.8 Hz, 1H), 7.26-7.24 (m, 2H), 7.07-7.05 (m, 3H), 7.00-6.99 (m, 2H), 2.39-2.30 (m, 1H), 2.18 (s, 3H), 1.96 (s, 3H), 1.95-1.88 (m, 1H), 1.55 (s, 3H), 0.76 (t, J = 7.6 Hz, 3H); ^{13}C NMR (100 MHz, $CDCl_3$): δ 191.71 (t, J = 28.1Hz), 139.15, 137.12, 136.18 (t, J = 7.7 Hz), 135.58 (t, J = 2.5 Hz), 133.19, 130.56, 130.34, 128.96, 128.80, 127.31, 121.59 (t, J = 260.8 Hz), 48.39 (t, J = 20.5 Hz), 25.91 (t, J = 4.0 Hz), 20.75, 18.10 (t, J = 4.3 Hz), 14.03, 7.79; ^{19}F NMR (376 MHz, $CDCl_3$): δ -102.77 (d, J = 251.9 Hz, 1F), -104.30 (d, J = 251.9 Hz, 1F); IR (ATR): 2976, 1533, 1456, 1340, 1227, 1194, 1020, 928, 870, 706, 681; HRMS (ESI): Exact mass calcd for $C_{20}H_{22}F_2NaOS$ $[M+Na]^+$: 371.1252, Found: 371.1250.

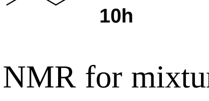

10h

The E/Z ratio of fluorinated silyl enol ether **2k** used in this reaction is 1:2.3. Column chromatography (PE/CH_2Cl_2 = 2:1, v/v) afforded product **10h** as colorless oil (66.7 mg, 78% yield). ^{19}F NMR of crude mixture indicated the d.r. value was 1.3:1. ^{1}H NMR for mixture of diastereomers (400 MHz, $CDCl_3$): δ 7.61 (d, J = 7.6 Hz, 0.86H), 7.50 (d, J = 7.6

Hz, 1.12H), 7.44 (t, J = 7.6 Hz, 0.47H), 7.38 (t, J = 7.2 Hz, 0.60H), 7.29-7.18 (m, 3.16H), 7.11 (d, J = 8.0 Hz, 1.17H), 7.04 (d, J = 8.0 Hz, 0.87H), 6.95 (d, J = 8.0 Hz, 1.12H), 5.50 (d, J = 48.4 Hz, 0.55H), 5.46 (d, J = 48.8 Hz, 0.55H), 2.26 (s, 1.29H), 2.21 (s, 1.74H), 2.19-2.08 (m, 1.05H), 1.95-1.86 (m, 0.58H), 1.74-1.65 (m, 0.51H), 1.45 (s, 1.30H), 1.43 (s, 1.71H), 0.75-0.70 (m, 3.00H); ^{13}C NMR for mixture of diastereomers (100 MHz, $CDCl_3$): δ 197.24 (d, J = 21.8 Hz), 197.06 (d, J = 21.1 Hz), 138.42 (d, J = 1.9 Hz), 137.76 (d, J = 4.0 Hz), 136.67, 136.64, 136.18, 136.13, 132.78, 132.50, 128.89, 128.82, 128.70, 128.66, 128.05, 127.86, 127.40, 127.33, 99.62 (dd, J = 191.5, 2.4 Hz), 99.05 (dd, J = 191.7, 2.7 Hz), 45.42 (d, J = 18.9 Hz), 45.36 (d, J = 18.8 Hz), 29.90 (d, J = 3.6 Hz), 29.54 (d, J = 5.1 Hz), 20.77 (d, J = 2.2 Hz), 20.72 (d, J = 2.1 Hz),19.69 (d, J = 4.8 Hz), 18.32 (d, J = 4.7 Hz), 7.95, 7.93; ^{19}F NMR for mixture of diastereomers (376 MHz, $CDCl_3$): δ -188.09 (s), -189.34 (s); IR (ATR): 2974, 1690, 1597, 1578, 1514, 1447, 1267, 1051, 883, 714, 687; HRMS (ESI): Exact mass calcd for $C_{19}H_{21}FNaO$ $[M+Na]^+$: 307.1469, Found: 307.1485.

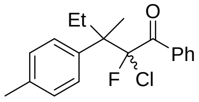

10i

The fluorinated silyl enol ether **2l** used in this reaction is the mixture of *Z*- and *E*-isomer (5:1). Column chromatography (PE/CH_2Cl_2 = 1:0 to 20:1, v/v) afforded product **10i** as colorless oil (62.4 mg, 65% yield). ^{19}F NMR of crude mixture indicated the d.r. value was 1.1:1. ^{1}H NMR for mixture of diastereomers (400 MHz, $CDCl_3$): δ 7.44-7.37 (m, 3H), 7.29-7.20 (m, 4H), 7.08-7.01 (m, 2H), 2.72-2.63 (m, 0.48H), 2.51-2.42 (m, 0.52H), 2.28 (s, 1.44H), 2.24 (s, 1.54H), 2.18-2.09 (m, 0.54H), 1.98-1.89 (m, 0.50H), 1.76 (s, 1.51H), 1.72 (s, 1.45H), 0.79-0.73 (m, 3H); ^{13}C NMR for mixture of diastereomers (100 MHz, $CDCl_3$): δ 194.15 (d, J = 32.3 Hz), 193.74 (d, J = 32.4 Hz), 137.00, 136.91, 135.32, 135.30, 135.15 (d, J = 3.9 Hz), 134.99 (d, J = 3.8 Hz), 132.67, 132.38, 129.70, 129.63, 129.44, 129.33, 129.26, 129.21, 129.20, 128.75, 127.81, 127.70, 115.39 (d, J = 262.1 Hz), 115.22 (d, J = 263.8 Hz), 51.61 (d, J = 18.1 Hz), 51.03 (d, J = 18.0 Hz), 27.55 (d, J = 2.7 Hz), 26.89 (d, J = 6.9 Hz), 20.83 (d, J = 1.8 Hz), 20.79 (d, J = 1.8 Hz), 20.51 (d, J = 3.4 Hz), 19.65 (d, J = 5.7 Hz), 8.27, 7.72; ^{19}F NMR for mixture of diastereomers (376 MHz, $CDCl_3$): δ -111.39 (s), -113.61 (s); IR (ATR): 2976, 1699, 1597, 1578, 1514, 1447, 1252, 1053, 895, 793, 688; HRMS (ESI): Exact mass calcd for $C_{19}H_{20}ClFNaO$ $[M+Na]^+$: 341.1079, Found: 341.1079.

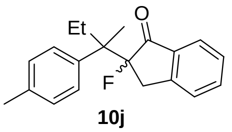

10j

Column chromatography (PE/CH_2Cl_2 = 2:1, v/v) afforded product **10j** as colorless oil (63.5 mg, 71% yield). ^{19}F NMR of crude mixture indicated the d.r. value was 1.1:1. ^{1}H NMR (400 MHz, $CDCl_3$) for mixture of diastereomers: δ 7.73 (d, J = 8.0 Hz, 0.36H), 7.68 (d, J = 7.6 Hz, 0.61H), 7.55 (t, J = 7.6 Hz, 0.39H), 7.48 (t, J = 7.6 Hz, 0.63H),

7.36-7.18 (m, 4.23H), 7.12 (d, *J* = 8.0 Hz, 0.79H), 7.01 (d, *J* = 8.0 Hz, 1.24H), 3.43-3.36 (m, 0.61H), 3.20-3.13 (m, 0.37H), 3.02-2.81 (m, 1H), 2.45-2.33 (m, 2.57H), 2.23 (s, 1.86H), 1.72-1.63 (m, 0.75H), 1.57 (s, 1.90H), 1.14 (s, 1.16H), 0.71 (t, *J* = 7.6 Hz, 1.86H), 0.61 (t, *J* = 7.6Hz, 1.12H); ^{13}C NMR (100 MHz, $CDCl_3$) for mixture of diastereomers: δ 201.61 (d, *J* = 19.4 Hz), 201.35 (d, *J* = 19.6 Hz), 150.68 (d, *J* = 4.0 Hz), 150.20 (d, *J* = 4.2 Hz), 138.98, 137.24 (d, *J* = 3.4 Hz), 136.10, 136.01, 135.78, 135.73 (d, *J* = 2.1 Hz), 135.55, 135.46 (d, *J* = 1.9 Hz), 128.73, 128.53, 128.15, 127.86, 127.83, 127.70, 125.87, 124.45, 124.11, 101.65 (d, *J* = 193.1 Hz), 101.25 (d, *J* = 192.8 Hz), 47.50 (d, *J* = 21.2 Hz), 47.06 (d, *J* = 20.6 Hz), 38.19 (d, *J* = 24.8 Hz), 37.35 (d, *J* = 25.0 Hz), 26.30 (d, *J* = 3.6 Hz), 25.55 (d, *J* = 7.6 Hz), 20.86 (d, *J* = 2.8 Hz), 20.76 (d, *J* = 2.7 Hz), 18.57, 18.51, 8.14, 8.03; ^{19}F NMR (376 MHz, $CDCl_3$) for mixture of diastereomers: δ -159.39 (s), -161.39 (s); IR (ATR): 2976, 1684, 1572, 1514, 1476, 1304, 1153, 1140, 1018, 907, 800, 716, 694; HRMS (ESI): Exact mass calcd for $C_{20}H_{21}FNaO$ $[M+Na]^+$: 319.1469, Found: 319.1466.

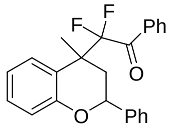

11

The reaction used 3.0 mol% of $Mg(ClO_4)_2{\cdot}6H_2O$ and 3.0 equiv. of **2a**. Column chromatography (PE/CH_2Cl_2 = 10:1, v/v) afforded product **11** as colorless oil (47.3 mg, 42% yield). The d.r. value could not be determined by the NMR analysis. ^{1}H NMR (400 MHz, $CDCl_3$): δ 7.91 (d, *J* = 7.6 Hz, 2H), 7.59-7.55 (m, 1H), 7.42-7.35 (m, 7H), 7.34-7.29 (m, 1H), 7.23-7.19 (m, 1H), 6.95 (dd, *J* = 8.4, 1.2 Hz, 1H), 6.90-6.86 (m, 1H), 5.37 (dt, *J* = 12.4, 2.0 Hz, 1H), 2.63 (dd, *J* = 15.2, 2.8 Hz, 1H), 1.92-1.84 (m, 1H), 1.57 (s, 3H); ^{13}C NMR (125 MHz, $CDCl_3$): δ 191.27 (dd, *J* = 31.3, 28.5 Hz), 155.33, 141.44, 134.31 (d, *J* = 2.5 Hz), 133.99, 130.32 (dd, *J* = 5.1, 3.1 Hz), 129.29, 129.19 (d, *J* = 1.4Hz), 128.54, 128.51, 127.96, 125.76, 120.90 (dd, *J* = 2.8, 0.9 Hz), 120.85 (t, *J* = 258.6 Hz), 120.42, 117.56, 74.18 (t, *J* = 4.0 Hz), 41.37 (t, *J* = 21.3 Hz), 39.95 (d, *J* = 3.0 Hz), 24.14 (t, *J* = 4.5 Hz); ^{19}F NMR (376 MHz, $CDCl_3$): δ -98.42 (d, *J* = 266.2 Hz, 1F), -102.48 (d, *J* = 266.2 Hz, 1F); IR (ATR): 2976, 2361, 2342, 1694, 1597, 1578, 1449, 1231, 1051, 754; HRMS (ESI): Exact mass calcd for $C_{24}H_{20}F_2NaO_2$ $[M+Na]^+$: 401.1324, Found: 401.1318.

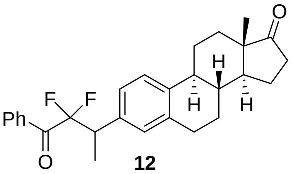

12

Column chromatography (PE/CH_2Cl_2 = 2:1, v/v) afforded product **12** as colorless oil (101.4 mg, 77% yield). ^{19}F NMR of crude mixture indicated the d.r. value was 1:1. ^{1}H NMR for mixture of diastereomers (400 MHz, $CDCl_3$): δ 7.96 (d, *J* = 8.0 Hz, 2H), 7.59 (t, *J* = 7.6 Hz, 1H), 7.44 (t, *J* = 8.0 Hz, 2H), 7.21 (d, *J* = 7.2 Hz, 1H), 7.06 (t, *J* = 8.8 Hz, 1H), 7.00 (d, *J* = 8.0 Hz, 1H), 3.74-3.61 (m, 1H),

2.88-2.84 (m, 2H), 2.53-2.46 (m, 1H), 2.41-2.36 (m, 1H), 2.28-2.23 (m, 1H), 2.18-1.94 (m, 4H), 1.67-1.34 (m, 9H), 0.90 (s, 3H); ^{13}C NMR for mixture of diastereomers (100 MHz, $CDCl_3$): δ 220.74, 190.03 (t, J = 29.9 Hz), 139.09, 136.40 (d, J = 2.8 Hz), 134.51 (t, J = 2.9 Hz), 133.87, 132.81, 129.83 (t, J = 3.7 Hz), 129.73, 129.61, 128.44, 126.47, 126.34, 125.34, 125.31, 119.48 (t, J = 255.4 Hz), 50.39, 47.87, 44.19, 43.37 (t, J = 19.9 Hz), 37.92, 35.77, 31.49, 29.27, 26.37, 25.51, 21.49, 14.30, 14.25, 13.76, 13.74; ^{19}F NMR for mixture of diastereomers (376 MHz, $CDCl_3$): δ -104.94 (d, J = 271.5 Hz), -105.06 (d, J = 271.1 Hz), -105.88 (d, J = 271.8 Hz); IR (ATR): 2932, 1738, 1697, 1597, 1503, 1450, 1279, 1196, 1051, 912, 752; HRMS (ESI): Exact mass calcd for $C_{28}H_{30}F_2NaO_2$ $[M+Na]^+$: 459.2106, Found: 459.2113.

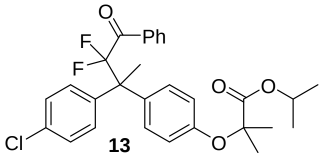

13

Column chromatography (PE/CH_2Cl_2 = 2:1, v/v) afforded product **13** as colorless oil (116.1 mg, 75% yield). ^{1}H NMR (400 MHz, $CDCl_3$): δ 7.65 (d, J = 7.6 Hz, 2H), 7.46 (t, J = 7.6 Hz, 1H), 7.30-7.18 (m, 6H), 7.12 (d, J = 8.4 Hz, 2H), 6.68 (d, J = 8.8 Hz, 2H), 5.10-5.01 (m, 1H), 1.97 (s, 3H), 1.54 (s, 6H), 1.18 (dd, J = 6.4, 3.6 Hz, 6H); ^{13}C NMR (100 MHz, $CDCl_3$): δ 190.11 (t, J = 31.0 Hz), 173.37, 154.58, 141.85 (d, J = 2.9 Hz), 134.67 (d, J = 4.0 Hz), 133.91 (t, J = 2.2 Hz), 133.27, 132.66, 130.36 (t, J = 2.1 Hz), 130.01, 129.59 (t, J = 3.9 Hz), 128.06, 127.88, 120.28 (t, J = 263.0 Hz), 117.89, 78.86, 68.81, 53.37 (t, J = 20.3 Hz), 25.27 (d, J = 7.7 Hz), 24.93 (t, J = 4.5 Hz), 21.43 (d, J = 2.4 Hz); ^{19}F NMR (376 MHz, $CDCl_3$) δ -97.76 (d, J = 274.5 Hz, 1F), -100.15 (d, J = 274.5 Hz, 1F). IR (ATR): 2984, 1726, 1597, 1508, 1448, 1383, 1248, 1177, 1013, 932, 804, 768; HRMS (ESI): Exact mass calcd for $C_{29}H_{29}ClF_2NaO_4$ $[M+Na]^+$: 537.1615, Found: 537.1622.

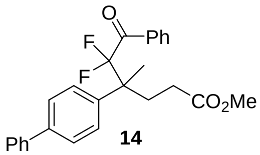

14

Column chromatography (PE/EtOAc = 20:1, v/v) afforded product **14** as colorless oil (81.6 mg, 64% yield). ^{1}H NMR (400 MHz, $CDCl_3$): δ 7.60 (d, J = 7.6 Hz, 2H), 7.50-7.47 (m, 2H), 7.44-7.37 (m, 7H), 7.34-7.30 (m, 1H), 7.23-7.19 (m, 2H), 3.63 (s, 3H), 2.81-2.74 (m, 1H), 2.37-2.23 (m, 2H), 2.14-2.06 (m, 1H), 1.64 (s, 3H); ^{13}C NMR (100 MHz, $CDCl_3$): δ 190.57 (t, J = 30.1 Hz), 173.51, 140.38, 140.25, 136.50, 134.10, 133.23, 129.70 (t, J = 4.0 Hz), 128.93, 128.67, 127.98, 127.35, 126.91, 126.88, 120.34 (t, J = 261.0 Hz), 51.57, 47.49 (t, J = 20.5 Hz), 28.91 (t, J = 4.2 Hz), 28.73, 18.46 (t, J = 4.2 Hz); ^{19}F NMR (376 MHz, $CDCl_3$): δ -104.58 (d, J = 262.4 Hz, 1F), -106.16 (d, J = 262.4 Hz, 1F); IR (ATR): 2976, 1690, 1597, 1578, 1489, 1449, 1202, 1173, 1049, 930, 899, 766; HRMS (ESI): Exact mass calcd for $C_{26}H_{24}F_2NaO_3$ $[M+Na]^+$: 445.1586, Found: 445.1580.

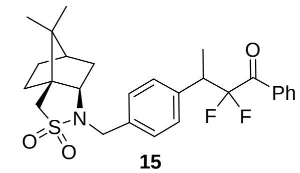

15

The reaction used 20 mol% of $Mg(ClO_4)_2 \cdot 6H_2O$ and 3.0 equiv. of **2a**. Column chromatography (PE/EtOAc = 6:1, v/v) afforded product **15** as colorless oil (74.1 mg, 51% yield). ^{19}F NMR of crude mixture indicated the d.r. value was 1:1. ^{1}H NMR for mixture of diastereomers (400 MHz, $CDCl_3$): δ 7.91 (t, *J* = 6.8 Hz, 2H), 7.58 (t, *J* = 7.6 Hz, 1H), 7.43 (t, *J* = 8.0 Hz, 2H), 7.29-7.28 (m, 2H), 7.23-7.20 (m, 2H), 4.37 (d, *J* = 14.4 Hz, 1H), 3.80-3.66 (m, 2H), 3.15 (s, 2H), 3.06 (dd, *J* = 8.0, 4.8 Hz, 1H), 1.83-1.81 (m, 2H), 1.72-1.70 (m, 1H), 1.49 (dd, *J* = 7.2, 2.8 Hz, 3H), 1.44-1.41 (m, 1H), 1.34-1.26 (m, 2H), 1.22-1.17 (m, 1H), 1.04 (d, *J* = 5.6 Hz, 3H), 0.88 (s, 3H); ^{13}C NMR for mixture of diastereomers (100 MHz, $CDCl_3$): δ 190.11 (t, *J* = 29.1 Hz), 190.10 (t, *J* = 30.4 Hz), 136.87 (t, *J* = 3.6 Hz), 135.12, 135.07, 133.95, 132.84, 129.76 (t, *J* = 3.0 Hz), 129.27, 129.25, 129.18, 128.51, 119.39 (t, *J* = 256.1 Hz), 67.84, 49.76, 49.09, 47.57, 47.30, 44.57, 44.55, 43.74 (t, *J* = 2.2 Hz), 43.68 (t, *J* = 2.2 Hz), 35.34, 32.20, 26.73, 20.23, 19.93, 14.19 (t, *J* = 4.5 Hz), 14.14 (t, *J* = 5.4 Hz); ^{19}F NMR for mixture of diastereomers (376 MHz, $CDCl_3$) δ -104.31 (dd, *J* = 270.3, 4.1 Hz), -104.52 (dd, *J* = 271.5, 3.8 Hz), -106.69 (d, *J* = 271.5 Hz), -107.34 (d, *J* = 270.3 Hz); IR (ATR): 2974, 1701, 1599, 1580, 1516, 1449, 1306, 1128, 1051, 912, 887; HRMS (ESI): Exact mass calcd for $C_{27}H_{31}F_2NNaO_3S$ $[M+Na]^+$: 510.1885, Found: 510.1897.

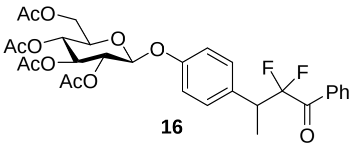

16

The reaction used 20 mol% of $Mg(ClO_4)_2 \cdot 6H_2O$ and 3.0 equiv. of **2a**. Column chromatography (PE/EtOAc = 3:1, v/v) afforded product **16** as white foam (140.8 mg, 77% yield). ^{19}F NMR of crude mixture indicated the d.r. value was 1.2:1. m.p. 116-118 ^{o}C; ^{1}H NMR for mixture of diastereomers (400 MHz, $CDCl_3$): δ 7.94 (d, *J* = 8.4 Hz, 2H), 7.61-7.57 (m, 1H), 7.47-7.42 (m, 2H), 7.22 (d, *J* = 8.4 Hz, 2H), 6.92 (d, *J* = 8.4 Hz, 2H), 5.33-5.24 (m, 2H), 5.17 (t, *J* = 9.6 Hz, 1H), 5.08 (d, *J* = 7.2 Hz, 1H), 4.31 (d, *J* = 5.2 Hz, 0.37H), 4.28 (d, *J* = 5.2 Hz, 0.59H), 4.17 (d, *J* = 1.2 Hz, 0.57H), 4.14 (d, *J* = 1.6 Hz, 0.40H), 3.89-3.85 (m, 1H), 3.79-3.65 (m, 1H), 2.07-2.03 (m, 12H),1.47-1.44 (m, 3H); ^{13}C NMR for mixture of diastereomers (100 MHz, $CDCl_3$): δ 189.83 (t, *J* = 30.2 Hz), 170.37, 170.04, 169.25, 169.11, 156.14, 133.94, 132.63, 132.07, 130.19, 129.72 (t, *J* = 3.3 Hz), 128.46, 119.25 (t, *J* = 255.7 Hz), 116.71, 116.66, 98.80, 98.75, 72.59, 71.87, 71.03, 68.19, 61.81, 42,97 (t, *J* = 22.1 Hz), 42.96 (t, *J* = 22.1 Hz), 20.49, 20.44, 14.29 (t, *J* = 5.3 Hz); ^{19}F NMR for mixture of diastereomers (376 MHz, $CDCl_3$): δ -105.01 (d, *J* = 274.5 Hz), -105.04 (dd, *J* = 273.4, 1.5 Hz), -106.11 (d, *J* = 273.7 Hz), -106.12 (d, *J* = 273.7 Hz); IR (ATR): 2974, 1701, 1599, 1514, 1211, 1047, 908, 885, 716; HRMS (ESI): Exact mass calcd for $C_{30}H_{32}F_2NaO_{11}$ $[M+Na]^+$: 629.1805, Found: 629.1796.

Gram-scale synthesis and elaborations of products

Gram-scale synthesis of 3b

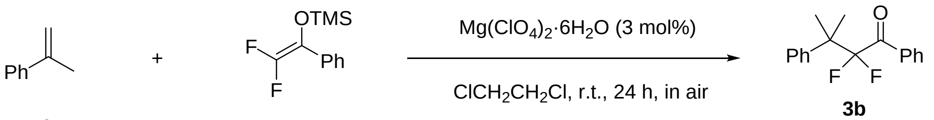

1b **2a** (1.5 equiv.) **3b** **1.290 g**, 78%

Under an air atmosphere, to a 100 mL flask were added $Mg(ClO_4)_2$·$6H_2O$ (59.6 mg, 0.18 mmol, 3 mol%) and $ClCH_2CH_2Cl$ (30.0 mL), followed by the addition of α-methyl styrene **1b** (0.709 g, 6.0 mmol) and difluoroenoxysilane **2a** (2.054 g, 9.0 mmol). The resulting mixture was stirred at room temperature until full conversion of **1b** by TLC analysis (about 24 h). After being concentrated under reduced pressure, the crude residue was purified by flash column chromatography (PE/CH_2Cl_2 = 1:0 to 20:1, v/v) to afford 1.290 g of product **3b** in 78% yield as colorless oil.

Transformations of products 3a and 3b

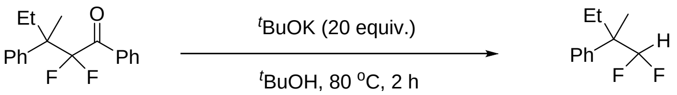

3a **17,** 78%

To a solution of **3a** (57.7 mg, 0.2 mmol), ^{t}BuOH (2.0 mL) in a 10 mL sealed tube was added ^{t}BuOK (449.0 mg, 4.0 mmol), and the resulting mixture was stirred at 80 °C. After full consumption of **3a** by TLC analysis (about 2 h), EtOAc (10 mL) and saturated aqueous NH_4Cl (5.0 mL) were added subsequently. The mixture was transferred to a separating funnel. The organic layer was separated, and the aqueous phase was extracted with EtOAc (2 × 10 mL). The combined organic layers were dried over Na_2SO_4 and concentrated under reduced pressure. The crude residue was purified by flash column chromatography with PE to provide **17** as colorless oil (28.7 mg, 78% yield). ^{1}H NMR (400 MHz, $CDCl_3$): δ 7.36-7.35 (m, 4H), 7.30-7.25 (m, 1H), 5.73 (t, *J* = 56.8 Hz, 1H), 2.07-1.98 (m, 1H), 1.77-1.68 (m, 1H), 1.41 (s, 3H), 0.72 (t, *J* = 7.6 Hz, 3H); ^{13}C NMR (100 MHz, $CDCl_3$): δ 140.03, 128.32, 127.39, 126.88, 120.01 (t, *J* = 246.3 Hz), 46.01 (t, *J* = 17.7 Hz), 27.80 (t, *J* = 3.4 Hz), 16.83 (t, *J* = 4.4 Hz), 7.61; ^{19}F NMR (376 MHz, $CDCl_3$): δ -125.16 (d, *J* = 270.3 Hz, 1F), -126.83 (d, *J* = 270.0 Hz, 1F); IR (ATR): 2976, 1500, 1464, 1391, 1368, 1204, 1117, 1057, 926, 762; HRMS (ESI): Exact mass calcd for $C_{11}H_{15}F_2$ $[M+H]^+$: 185.1136, Found: 185.1146.

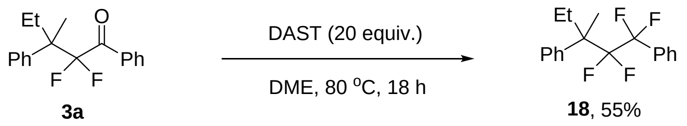

To a 10 mL sealed tube were added **3a** (57.7 mg, 0.2 mmol) and anhydrous 1,2-dimethoxyethane (DME) (1.0 mL), followed by the addition of DAST (0.6 mL, 4.0 mmol) at -78 °C. The reaction mixture was slowly heated to 80 °C, and stirred at 80 °C until full conversion of **3a** by TLC analysis (about 18 h). After cooling to room temperature, saturated aqueous $NaHCO_3$ (5.0 mL) was poured. The resulting solution was extracted with EtOAc (3 × 10 mL). The combined organic phases were washed with aqueous HCl (4.0 mL, 2 M), dried over Na_2SO_4, and concentrated under reduced pressure. The crude residue was purified by flash column chromatography with PE to afford product **18** as colorless oil (34.7 mg, 55% yield). ^{1}H NMR (400 MHz, $CDCl_3$): δ 7.43-7.21 (m, 10H), 2.40-2.31 (m, 1H), 2.02-1.93 (m, 1H), 1.61 (s, 3H), 0.67 (t, *J* = 7.6 Hz, 3H); ^{13}C NMR (125 MHz, $CDCl_3$): δ 138.92, 132.43 (t, *J* = 24.9 Hz), 130.44 (t, *J* = 1.6 Hz), 128.62, 127.86, 127.69, 126.84, 126.72 (tt, *J* = 6.8, 1.9 Hz), 119.26 (tt, *J* = 256.8, 35.8 Hz), 118.63 (tt, *J* = 253.5, 37.8 Hz), 48.24 (tt, *J* = 19.8, 2.1 Hz), 27.36 (tt, *J* = 4.1, 2.4 Hz), 18.45 (m), 7.60; ^{19}F NMR (376 MHz, $CDCl_3$): δ -105.04 (d, *J* = 3.8 Hz, 2F), -112.0 (d, *J* = 4.5 Hz, 1F), -112.05 (d, *J* = 4.5 Hz, 1F); IR (ATR): 2978, 1601, 1499, 1452, 1279, 1130, 1070, 934, 897, 762; HRMS (ESI): Exact mass calcd for $C_{18}H_{18}F_4Na$ $[M+Na]^+$: 333.1237, Found: 333.1248.

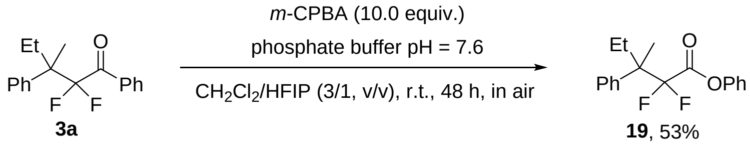

Under an air atmosphere, to a solution of **3a** (144.2 mg, 0.5 mmol) in a mixed solvent of CH_2Cl_2 and HFIP (5.0 mL, 3/1, v/v) were added *m*-chloroperoxybenzoic acid (*m*-CPBA) (1.015 g, 5.0 mmol, 85%wt), and phosphate buffer (0.5 mL, pH = 7.6) at room temperature[26]. The resulting mixture was stirred at the same temperature until the complete consumption of **3a** as indicated by TLC analysis (about 48 h). After quenching with saturated aqueous $Na_2S_2O_3$ (3.0 mL), the mixture was extracted with CH_2Cl_2 (3 × 10 mL). The combined organic layer was washed sequentially with saturated $NaHCO_3$ and brine, then dried over anhydrous Na_2SO_4 and concentrated under reduced pressure. The residue was purified by flash column chromatography with PE to give product **19** as colorless oil (80.7 mg, 53% yield). ^{1}H NMR (400 MHz, $CDCl_3$): δ 7.48 (d, *J* = 7.6 Hz, 2H), 7.41-7.37 (m, 2H), 7.35-7.27 (m, 3H), 7.21-7.17 (m, 1H), 6.72-6.68 (m, 2H), 2.46-2.37 (m, 1H), 2.00-1.91 (m, 1H), 1.65

(s, 3H), 0.77 (t, J = 7.6 Hz, 3H); ^{13}C NMR (100 MHz, $CDCl_3$): δ 162.33 (t, J = 34.1 Hz), 149.53, 138.09 (d, J = 3.6 Hz), 129.42, 128.44, 128.34, 127.56, 126.45, 120.81, 118.28 (t, J = 259.1 Hz), 48.24 (t, J = 20.5 Hz), 26.50 (t, J = 5.5 Hz), 17.83 (t, J = 4.6 Hz), 7.75; ^{19}F NMR (376 MHz, $CDCl_3$): δ -109.87 (d, J = 243.6 Hz, 1F), -112.61 (d, J = 243.6 Hz, 1F); IR (ATR): 2976, 1773, 1493, 1387, 1281, 1186, 1092, 997, 853, 746; HRMS (ESI): Exact mass calcd for $C_{18}H_{18}F_2NaO_2$ $[M+Na]^+$: 327.1167, Found: 327.1167.

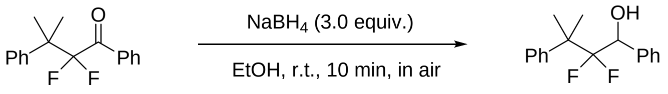

3b **20**, 98%

Under an air atmosphere, to a 5.0 mL vial were added **3b** (54.9 mg, 0.2 mmol) and anhydrous EtOH (2.5 mL), followed by the addition of $NaBH_4$ (23.4 mg, 0.6 mmol) in one portion at room temperature. The resulting mixture was stirred at the same temperature until the complete consumption of **3b** as indicated by TLC analysis (about 10 min). After quenching with saturated aqueous NH_4Cl (5.0 mL), the solution was extracted with CH_2Cl_2 (3 × 10 mL). Followed by work-up procedure, and column chromatography (PE/CH_2Cl_2 = 2/1, v/v) afforded product **20** as colorless oil (54.0 mg, 98% yield). ^{1}H NMR (400 MHz, $CDCl_3$): δ 7.53 (d, J = 7.6 Hz, 2H), 7.35 (t, J = 6.8 Hz, 2H), 7.30-7.27 (m, 3H), 7.23-7.21 (m, 3H), 4.63-4.56 (m, 1H), 2.02-2.01 (m, 1H), 1.61 (s, 3H), 1.42 (s, 3H); ^{13}C NMR (100 MHz, $CDCl_3$): δ 142.62 (t, J = 2.5 Hz), 137.48, 128.51, 128.22, 128.06, 127.62 (t, J = 1.8 Hz), 127.15, 123.25 (dd, J = 256.6, 251.2 Hz), 74.05 (dd, J = 32.5, 24.6 Hz), 45.09 (t, J = 22.2 Hz), 23.85 (t, J = 4.5 Hz), 23.66; ^{19}F NMR (376 MHz, $CDCl_3$): δ -108.77 (d, J = 255.3 Hz, 1F), -119.38 (d, J = 255.7 Hz, 1F); IR (ATR): 2978, 1601, 1497, 1456, 1393, 1192, 1030, 814, 727; HRMS (ESI): Exact mass calcd for $C_{17}H_{18}F_2NaO$ $[M+Na]^+$: 299.1218, Found: 299.1216.

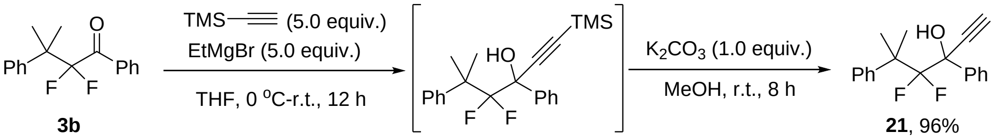

3b **21**, 96%

To a 25 mL oven-dried Schlenk tube were added trimethylsilylacetylene (147.0 μL, 1.0 mmol) and anhydrous THF (2.0 mL), followed by the addition of EtMgBr (1.0 mL, 1.0 mmol, 1.0 M in THF) at room temperature. The reaction was stirred at room temperature for 3 h. After **3b** (54.9 mg, 0.2 mmol) was added at 0 °C in one portion, the resulting mixture was stirred at room temperature till full consumption of **3b** by TLC analysis (about 12 h). The reaction was quenched with saturated aqueous NH_4Cl (5.0 mL), and extracted with CH_2Cl_2 (3 × 10 mL). The combined organic layers were washed

with brine, dried over Na_2SO_4 and concentrated under reduced pressure. The obtained crude tertiary alcohol was used directly in the next step without further purification.

To the crude tertiary alcohol was added K_2CO_3 (27.6 mg, 0.2 mmol) and MeOH (1.0 mL), and stirred at room temperature till full conversion by TLC analysis. The mixture was concentrated under vacuum, followed by the addition of saturated aqueous NH_4Cl (5.0 mL). The solution was extracted with EtOAc (3 × 10 mL). After work-up procedure, and column chromatography (PE/CH_2Cl_2 = 2:1, v/v) gave product **21** as colorless oil (57.4 mg, 96% yield). ^{1}H NMR (400 MHz, $CDCl_3$): δ 7.60-7.58 (m, 2H), 7.47 (d, *J* = 7.6 Hz, 2H), 7.29-7.27 (m, 4H), 7.25-7.23 (m, 2H), 2.54 (s, 1H), 2.46 (s, 1H), 1.52 (s, 3H), 1.48 (s, 3H); ^{13}C NMR (125 MHz, $CDCl_3$): δ 142.40 (t, *J* = 2.3 Hz), 138.14, 128.56, 128.18 (t, *J* = 1.6 Hz), 127.85, 127.60, 127.38 (t, *J* = 1.8 Hz), 127.03, 122.06 (dd, *J* = 260.8, 258.5 Hz), 83.13 (dd, *J* = 4.9, 1.4 Hz), 75.89 (t, *J* = 30.8 Hz), 75.78, 45.81 (t, *J* = 22.4 Hz), 25.04 (dd, *J* = 5.9, 4.1 Hz), 24.96 (t, *J* = 5.1 Hz); ^{19}F NMR (376 MHz, $CDCl_3$): δ -105.55 (d, *J* = 257.2 Hz, 1F), -107.11 (d, *J* = 257.6 Hz, 1F); IR (ATR): 2978, 1601, 1450, 1132, 1059, 1005, 845, 750; HRMS (ESI): Exact mass calcd for $C_{19}H_{18}F_2NaO$ $[M+Na]^+$: 323.1218, Found: 323.1212.

Supplementary Discussion

Comparison of the hydrolysis rate of difluoroenoxysilane 2a and nonfluorinated analogue 2o

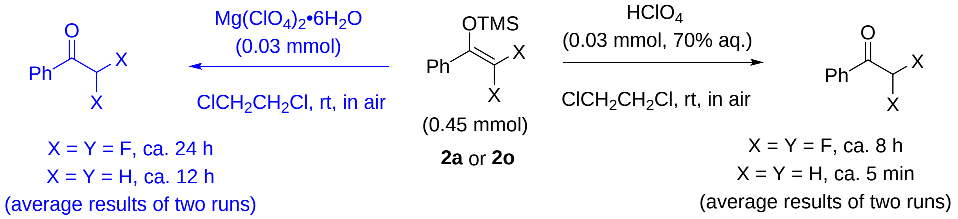

The rate of hydrolysis of difluoroenoxysilane **2a** and nonfluorinated silyl enol ether **2o** were evaluated under the catalysis of $Mg(ClO_4)_2{\cdot}6H_2O$. The general procedure for the determination of the time needed for the hydrolysis of **2a** and **2o** was as follows: Under an air atmosphere, to a 5.0 mL vial was added $Mg(ClO_4)_2{\cdot}6H_2O$ (9.9 mg, 0.03 mmol), anhydrous $ClCH_2CH_2Cl$ (3.0 mL) and silyl enol ether **2a** or **2o** (0.45 mmol). The resulting mixture was stirred at room temperature, which was monitored by TLC and GC-MS analysis of an aliquot take form the crude mixture. It was found that the full hydrolysis of difluoroenoxysilane **2a** requires 24 h (average time of two runs), which is much slower than nonfluorinated one **2o** (12 h, average time of two runs). Considering that $Mg(ClO_4)_2{\cdot}6H_2O$ is heterogeneous in reaction system, we further compared the rate of hydrolysis of both **2a** and **2o** in the presence of $HClO_4$ (70% aq.) using the similar procedure. The same trend was observed as expected. The time for full hydrolysis of difluoroenoxysilane **2a** is 8 h (average time of two runs), while that of nonfluorinated **2o** completely hydrolyzed within only 5 min (average time of two runs). Under the standard condition using $Mg(ClO_4)_2{\cdot}6H_2O$ as the catalyst, although nonfluorinated silyl enol ether **2o** is much easier to be hydrolyzed, it failed to produce detectable product by GC-MS analysis of the reaction mixture. This highlighted that the uniqueness of the difluoro substitution of **2a** in this hydroalkylation.

Mechanistic studies

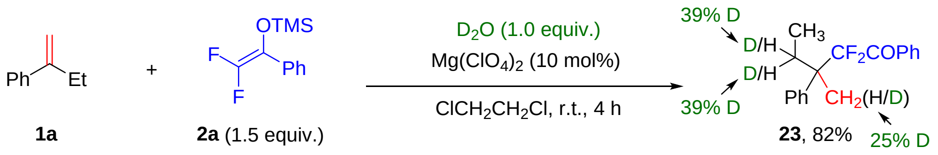

In a glovebox, to a 5.0 mL vial was added anhydrous $Mg(ClO_4)_2$ (6.7 mg, 0.03 mmol) and anhydrous $ClCH_2CH_2Cl$ (3.0 mL), followed by the addition of α-ethyl styrene **1a** (39.7 mg, 0.3 mmol), difluoroenoxysilane **2a** (102.6 mg, 0.45 mmol) and D_2O (6.0 mg, 0.3 mmol). The resulting mixture was stirred at room temperature in the glovebox. After full conversion of **1a** by TLC analysis (about 4 h), the mixture was concentrated under reduced pressure. The crude residue was purified by flash column chromatography (PE/CH_2Cl_2 = 1:0 to 20:1, v/v) to afford deuterated product **23** as colorless oil (70.8 mg, 82% yield). The deuterated ratio of **23** was determined by ^1H NMR analysis using CD_3OD as the NMR solvent. ^1H NMR (500 MHz, CD_3OD): δ 7.48 (d, J = 8.0 Hz, 2H), 7.45-7.41 (m, 1H), 7.36-7.34 (m, 2H), 7.25-7.14 (m, 5H), 2.47-2.40 (m, 0.61H, 39% D), 1.94-1.86 (m, 0.61H, 39% D), 1.57 (s, 2.75H, 25% D), 0.74-0.70 (m, 3H); ^{13}C NMR (125 MHz, CD_3OD): δ 192.78 (t, J = 29.8 Hz), 139.30, 135.80, 134.42 (d, J = 2.5 Hz), 130.71 (dd, J = 9.9, 5.9 Hz), 130.03, 129.26, 129.17 (d, J = 2.1 Hz), 128.44, 122.35 (t, J = 259.6 Hz), 49.67-48.58 (m), 27.28 (dd, J = 9.5, 7.5 Hz), 18.70-18.52 (m), 8.08-7.76 (m); ^{19}F NMR (376 MHz, CD_3OD): δ -106.35 (d, J = 255.3 Hz), 104.85 (d, J = 259.1 Hz), -107.96 (d, J = 258.7 Hz).

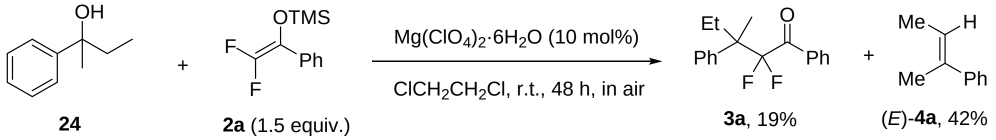

Under an air atmosphere, to a 5 mL vial were added $Mg(ClO_4)_2·6H_2O$ (6.6 mg, 0.02 mmol), $ClCH_2CH_2Cl$ (2.0 mL) and tertiary alcohol **24** (30.0 mg, 0.2 mmol), followed by the addition of difluoroenoxysilane **2a** (68.5 mg, 0.3 mmol). The resulting mixture was stirred at room temperature. Upon completion of the reaction by TLC analysis (48 h), the mixture was concentrated under reduced pressure. The crude residue was purified by silica gel column chromatography (PE/CH_2Cl_2 = 1:0 to 20:1, v/v) to provide the product **3a** (11.0 mg, 19% yield) and the alkene (*E*)-**4a**[10] (11.1 mg, 42% yield). ^1H NMR for (*E*)-**4a** (500 MHz, $CDCl_3$): δ 7.37-7.36 (m, 2H), 7.31-7.29 (m, 2H), 7.22-7.19 (m, 1H), 5.86 (qq, J = 7.0, 1.5 Hz, 1H), 2.03 (t, J = 1.5 Hz, 3H), 1.81(dd, J = 7.0, 1.5 Hz, 3H); ^{13}C NMR for (*E*)-**4a** (125 MHz, $CDCl_3$): δ 144.03, 135.49, 128.12, 126.37, 125.51, 122.44, 15.47, 14.32.

Biological activities study

Given that α,α-difluoroketones are interesting targets for the development of pharmaceutical agents, we initially evaluated the *in vitro* cytotoxic activity of the newly synthesized difluorinated ketones in human colorectal cancer cells (HCT116) and human osteosarcoma cells (Saos2) using CCK-8 assay. We first treated HCT116 cells with ten difluorinated products for 72 h and then measured the cell viability using CCK-8 assay. All these compounds showed good growth inhibitory activity at 30 μM, as shown in Supplementary Table 9. The IC_{50} values of cytotoxic effects was further measured in HCT116 cells, which ranged from 4.22 to 58.25 μM. Among them, products **3ag**, **3ai** and **12** exhibited the most potent inhibitory effects with IC_{50} of 6.7 μM, 5.7 μM and 4.2 μM, respectively. Meanwhile, the preliminary investigation of the cytotoxic activity of five of selected compounds in Saos2 cells using CCK-8 assay also provided good growth inhibitory activity. These preliminary results demonstrate that our difluorinated ketones are potentially valuable in anticancer drug development.

Supplementary Table 9. Inhibitory activity of selected compounds in HCT116 and Saos2 cells proliferation present as inhibition (%) and IC_{50} (μM)

Compound number.	HCT116		Saos2
	Inhibition (%)	IC_{50} (μM)	Inhibition (%)
**3a**	64.35	58.25	ND
**3e**	62.25	19.71	52.72
**3v**	63.41	ND	44.35
**3ad**	92.55	12.86	ND
**3ae**	82.46	33.58	ND
**3ag**	92.44	6.68	ND
**3ai**	90.21	5.74	ND
**5c**	97.32	11.34	76.03
**8j**	63.10	27.34	66.52
**12**	98.83	4.22	68.44

IC_{50}: half-maximal inhibitory concentrations. ND: Not detected.

General procedure: HCT116 cells or Saos2 cells were seeded in 96-well plates at 3000/well overnight and treated with every synthesized compound at 30 μM. The control cells were treated with the vehicle DMSO. After 72 h incubation, 10 μL of CCK8 solution (5 g/L; Yeasen) in the media was added to each well and incubated for additional 4 h. Finally, the optical density (OD) was measured at 450 nm using a microplate reader (spectraMax M5/M5e, Sunnyvale, CA, USA) and a reference wavelength at 620 nm. As for the compounds with significant growth inhibitory effects, the IC_{50} value was determined by testing the inhibitory effects of the compound with 10 gradient-dilution concentrations with at least three replicates per concentration.

X-ray Crystallography Analysis

Preparation and structure confirmation of product 3s’

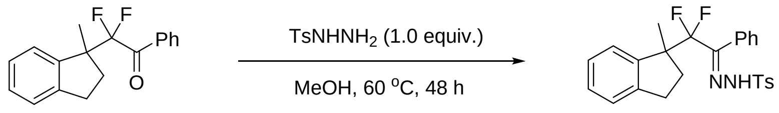

3s **3s′**, 56%

To a 10 mL sealed tube were added $TsNHNH_2$ (93.1 mg, 0.5 mmol), **3s** (143.2 mg, 0.5 mmol), followed by the addition of MeOH (4.0 mL), and the reaction mixture was stirred at 60°C. After full consumption of **3s** as indicated by TLC analysis (about 48 h), the mixture was cooled to room temperature, and quenched with water (5.0 mL). The resulting solution was extracted with EtOAc (3 × 20 mL), the combined organic phases were washed with brine, dried over Na_2SO_4 and concentrated under reduced pressure. The residue was purified by flash column chromatography (PE/EtOAc = 5:1, v/v) to afford hydrazine derivative **3s’** as white solid (127.3 mg, 56% yield). Its structure was determined by the X-ray crystallographic analysis, which further confirmed the structure of **3s**. m.p. 120-122 °C; ^{1}H NMR (400 MHz, $CDCl_3$): δ 7.74 (d, *J* = 8.4 Hz, 2H), 7.56 (s, 1H), 7.36-7.28 (m, 5H), 7.18-7.13 (m, 2H), 7.06 (d, *J* = 7.6 Hz, 1H), 6.99-6.95 (m, 1H), 6.73 (d, *J* = 7.2 Hz, 2H), 2.88-2.84 (m, 2H), 2.71-2.65 (m, 1H), 2.46 (s, 3H), 1.95-1.88 (m, 1H), 1.47 (s, 3H); ^{13}C NMR (125 MHz, $CDCl_3$): δ 149.37 (dd, *J* = 35.8, 30.6 Hz), 144.49, 144.41, 144.13 (d, *J* = 3.6 Hz), 135.08, 130.16, 129.68, 129.19, 127.93, 127.87, 127.80, 127.65, 126.27, 125.34 (t, *J* = 1.8 Hz), 124.60, 121.18 (dd, *J* = 250.9, 245.4 Hz), 55.10 (t, *J* = 23.4 Hz), 34.68 (t, *J* = 3.1 Hz), 30.67, 23.18 (dd, *J* = 5.5, 3.0 Hz), 21.64; ^{19}F NMR (376 MHz, $CDCl_3$): δ -99.03 (d, *J* = 263.6 Hz, 1F), -101.98 (d, *J* = 263.6 Hz, 1F); IR (ATR): 2976, 1597, 1495, 1348, 1169, 1053, 957, 814, 762; HRMS (ESI): Exact mass calcd for $C_{25}H_{24}F_2N_2NaO_2S$ $[M+Na]^+$: 477.1419, Found: 477.1405.

Data intensity of **3s’** was collected using a XtaLAB PRO MM003 (Cu radiation) at 293 K in a nitrogen stream. Data collection and reduction were done by using the XtaLAB software package. The structures were solved by direct methods and refined by full-matrix least-squares on F^2 with anisotropic displacement parameters for non-H atoms using SHELX-97. Hydrogen atoms were added at their geometrically idea positions and refined isotropically. Crystal data for **3s’**: $C_{25}H_{24}F_2N_2O_2S$, T = 293(2) K, triclinic, P-1, a = 10.3976(5) Å, b = 10.7081(4) Å, c = 12.8477(7) Å, α = 74.223(4)°, β = 66.674(5)°, γ = 65.186(4)°. V = 1182.63(11) Å^3. Z = 2, ρ_{calc} = 1.276 g/cm^3. Reflections collected 10877; independent reflections 4110 [Rint = 0.0313, Rsigma = 0.0391], R1 = 0.0912, wR2 = 0.2471 (all data), GOF = 1.032, and 309 parameter.

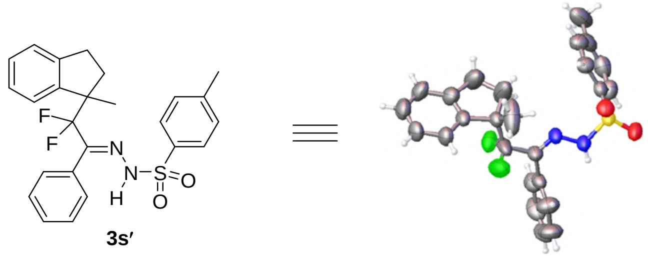

3s′

Supplementary Table 10. Crystal data and structure refinement for **3s’**.

Identification code	**3s’**
Empirical formula	$C_{25}H_{24}F_2N_2O_2S$
Formula weight	454.52
Temperature/K	293(2)
Crystal system	triclinic
Space group	P-1
a/Å	10.3976(5)
b/Å	10.7081(4)
c/Å	12.8477(7)
α/°	74.223(4)
β/°	66.674(5)
γ/°	65.186(4)
Volume/Å3	1182.63(11)
Z	2
ρcalcg/cm3	1.276
μ/mm-1	1.553
F(000)	476.0
Crystal size/mm3	0.52 × 0.46 × 0.42
Radiation	CuKα (λ = 1.54184)
2Θ range for data collection/°	9.172 to 134.134
Index ranges	-12 ≤ h ≤ 12, -12 ≤ k ≤ 9, -15 ≤ l ≤ 15
Reflections collected	10877
Independent reflections	4110 [Rint = 0.0313, Rsigma = 0.0391]
Data/restraints/parameters	4110/72/309
Goodness-of-fit on F2	1.032
Final R indexes [I>=2σ (I)]	R1 = 0.0837, wR2 = 0.2378
Final R indexes [all data]	R1 = 0.0912, wR2 = 0.2471
Largest diff. peak/hole / e Å-3	1.09/-0.72

Supplementary Table 11. Fractional Atomic Coordinates (×10^4) and Equivalent Isotropic Displacement Parameters (Å^2×10^3) for **3s’**. U_{eq} is defined as 1/3 of of the trace of the orthogonalised U_{IJ} tensor.

Atom	x	y	z	U(eq)
S1	-331.9(8)	7233.2(8)	5119.3(7)	58.5(3)
O1	-824(3)	8675(2)	4724(2)	70.9(7)
O2	-1278(3)	6456(3)	5379(3)	74.3(7)
N1	1153(3)	6458(3)	4096(3)	63.3(7)
N2	2259(3)	7027(3)	3663(3)	64.9(8)
C1	233(3)	6992(3)	6297(3)	59.6(8)
C2	397(5)	5745(4)	7023(4)	79.9(11)
C3	832(5)	5574(5)	7949(4)	90.1(13)
C4	1115(5)	6608(5)	8175(4)	85.0(12)
C5	983(5)	7815(5)	7427(4)	85.4(12)
C6	539(4)	8028(4)	6495(3)	69.8(9)
C7	1606(7)	6390(8)	9186(5)	119(2)
C8	6005(4)	8589(3)	2126(3)	64.5(9)
C9	6647(5)	8892(5)	964(4)	78.0(10)
C10	7973(5)	9144(5)	565(4)	91.3(13)
C11	8666(5)	9073(5)	1306(5)	90.8(13)
C12	8024(6)	8798(5)	2438(4)	91.3(13)
C13	6665(5)	8552(5)	2853(4)	82.8(12)
C14	5707(7)	8268(8)	4049(4)	114.4(19)
C15	4302(7)	8274(8)	3985(4)	117.9(18)
C16	4540(4)	8315(3)	2739(3)	62.1(8)
C17	3239(6)	9507(6)	2474(9)	158(4)
C18	4682(5)	7003(5)	2444(4)	109(2)

F1	4689(10)	7436(8)	1253(5)	111.6(18)
F2	5984(6)	6133(6)	2045(7)	93.9(16)
F1'	5537(7)	6624(7)	1461(5)	84.1(14)
F2'	5871(5)	6004(4)	2913(6)	84.6(13)
C19	3484(4)	6372(4)	2964(3)	67.8(9)
C20	3853(4)	5025(3)	2580(3)	64.5(9)
C21	3371(7)	5025(5)	1732(5)	94.7(14)
C22	3753(9)	3788(7)	1343(5)	120(2)
C23	4602(8)	2583(6)	1813(6)	117(2)
C24	5044(6)	2572(5)	2669(6)	106.3(18)
C25	4696(5)	3795(4)	3066(4)	83.7(12)

Supplementary Table 12. Anisotropic Displacement Parameters ($Å^2 \times 10^3$) for **3s'**. The Anisotropic displacement factor exponent takes the form: $-2\pi^2[h^2a^{*2}U_{11}+2hka^*b^*U_{12}+\ldots]$.

Atom	U11	U22	U33	U23	U13	U12
S1	45.4(4)	49.2(5)	75.5(6)	-17.4(3)	-5.5(3)	-19.1(3)
O1	67.5(15)	51.1(13)	87.4(16)	-15.3(11)	-20.2(12)	-15.3(11)
O2	51.6(13)	66.0(14)	105.0(19)	-24.7(13)	-7.9(12)	-28.1(11)
N1	55.5(15)	55.7(15)	77.8(18)	-22.3(13)	-1.9(13)	-28.6(12)
N2	57.2(16)	60.1(16)	75.9(18)	-23.5(13)	2.4(13)	-32.4(13)
C1	46.8(16)	53.6(17)	67.3(19)	-15.5(14)	-1.2(13)	-18.7(13)
C2	76(2)	67(2)	94(3)	0.4(19)	-22(2)	-33.5(19)
C3	72(3)	88(3)	84(3)	6(2)	-13(2)	-27(2)
C4	59(2)	102(3)	71(2)	-27(2)	-8.0(18)	-10(2)
C5	82(3)	83(3)	93(3)	-36(2)	-21(2)	-22(2)
C6	71(2)	56.6(19)	79(2)	-17.3(16)	-14.5(18)	-23.2(16)
C7	91(3)	160(5)	84(3)	-40(3)	-23(3)	-13(3)
C8	59.6(19)	54.7(17)	76(2)	-18.3(15)	-1.7(16)	-29.4(15)

C9	71(2)	87(3)	76(2)	-8.8(19)	-7.9(18)	-43(2)
C10	82(3)	108(3)	81(3)	-11(2)	3(2)	-59(3)
C11	72(2)	97(3)	107(3)	-11(3)	-10(2)	-51(2)
C12	96(3)	108(3)	98(3)	-3(2)	-34(3)	-66(3)
C13	94(3)	85(3)	78(2)	-11(2)	-12(2)	-54(2)
C14	132(5)	172(6)	76(3)	-15(3)	-21(3)	-100(5)
C15	122(4)	186(5)	70.1(19)	-17(3)	-12(3)	-95(4)
C16	60.6(18)	56.8(17)	65.0(16)	-14.4(14)	-0.8(15)	-31.2(15)
C17	68(3)	88(4)	256(9)	37(5)	-25(4)	-32(3)
C18	78(3)	99(3)	134(4)	-76(3)	42(3)	-50(3)
F1	134(4)	124(3)	86(3)	-28(2)	-4(3)	-75(3)
F2	79(3)	83(3)	108(3)	-23(3)	-8(3)	-31(2)
F1'	81(3)	84(3)	79(3)	-37(2)	10(2)	-39(2)
F2'	60(2)	60(2)	131(3)	-12(2)	-25(2)	-23.8(17)
C19	60.9(19)	58.9(19)	82(2)	-28.3(16)	0.6(17)	-29.2(16)
C20	63.4(19)	56.2(18)	67.1(19)	-22.4(15)	4.9(15)	-30.1(15)
C21	120(4)	75(3)	102(3)	-23(2)	-43(3)	-33(3)
C22	155(5)	111(5)	114(4)	-52(4)	-32(4)	-55(4)
C23	127(4)	79(3)	128(5)	-56(3)	19(4)	-52(3)
C24	102(4)	52(2)	135(5)	-14(2)	-6(3)	-28(2)
C25	90(3)	60(2)	91(3)	-3.7(19)	-14(2)	-34(2)

Supplementary Table 13. Bond Lengths for **3s’**.

Atom	Atom	Length/Å	Atom	Atom	Length/Å
S1	O1	1.421(2)	C12	C13	1.410(6)
S1	O2	1.431(2)	C13	C14	1.490(7)
S1	N1	1.646(3)	C14	C15	1.492(8)
S1	C1	1.751(4)	C15	C16	1.507(6)
N1	N2	1.379(4)	C16	C17	1.504(7)

N2	C19	1.268(4)	C16	C18	1.487(5)
C1	C2	1.393(5)	C18	F1	1.476(6)
C1	C6	1.382(5)	C18	F2	1.268(5)
C2	C3	1.375(7)	C18	F1'	1.278(5)
C3	C4	1.383(7)	C18	F2'	1.479(6)
C4	C5	1.376(7)	C18	C19	1.503(5)
C4	C7	1.504(7)	C19	C20	1.496(4)
C5	C6	1.379(6)	C20	C21	1.366(6)
C8	C9	1.382(6)	C20	C25	1.383(6)
C8	C13	1.345(6)	C21	C22	1.381(7)
C8	C16	1.525(4)	C22	C23	1.356(9)
C9	C10	1.383(6)	C23	C24	1.344(9)
C10	C11	1.375(7)	C24	C25	1.388(7)
C11	C12	1.346(7)			

Supplementary Table 14. Bond Angles for **3s'**.

Atom	Atom	Atom	Angle/˚	Atom	Atom	Atom	Angle/˚
O1	S1	O2	120.26(16)	C14	C15	C16	107.0(4)
O1	S1	N1	107.59(15)	C15	C16	C8	103.2(3)
O1	S1	C1	108.38(16)	C17	C16	C8	111.8(3)
O2	S1	N1	102.89(14)	C17	C16	C15	106.3(6)
O2	S1	C1	109.45(17)	C18	C16	C8	110.0(3)
N1	S1	C1	107.54(15)	C18	C16	C15	114.0(4)
N2	N1	S1	115.2(2)	C18	C16	C17	111.2(5)
C19	N2	N1	116.9(3)	C16	C18	C19	123.9(3)
C2	C1	S1	119.8(3)	F1	C18	C16	100.5(4)
C6	C1	S1	120.1(3)	F1	C18	C19	102.2(4)
C6	C1	C2	120.1(4)	F2	C18	C16	118.7(4)
C3	C2	C1	119.2(4)	F2	C18	F1	86.2(5)
C2	C3	C4	121.7(4)	F2	C18	C19	113.5(4)
C3	C4	C7	120.7(5)	F1'	C18	C16	119.8(4)

C5	C4	C3	117.9(4)	F1'	C18	F2'	88.3(5)
C5	C4	C7	121.4(5)	F1'	C18	C19	111.4(4)
C4	C5	C6	122.0(4)	F2'	C18	C16	100.3(4)
C5	C6	C1	119.1(4)	F2'	C18	C19	102.6(4)
C9	C8	C16	127.4(4)	N2	C19	C18	117.8(3)
C13	C8	C9	119.9(4)	N2	C19	C20	126.2(3)
C13	C8	C16	112.7(3)	C20	C19	C18	116.0(3)
C8	C9	C10	119.1(4)	C21	C20	C19	119.5(4)
C11	C10	C9	120.9(4)	C21	C20	C25	120.3(4)
C12	C11	C10	119.6(4)	C25	C20	C19	120.2(4)
C11	C12	C13	119.8(4)	C20	C21	C22	119.8(5)
C8	C13	C12	120.5(4)	C23	C22	C21	119.6(6)
C8	C13	C14	108.8(4)	C24	C23	C22	120.8(5)
C12	C13	C14	130.7(5)	C23	C24	C25	120.9(5)
C15	C14	C13	107.4(4)	C20	C25	C24	118.5(5)

Supplementary Table 15. Hydrogen Atom Coordinates (Å×10^4) and Isotropic Displacement Parameters (Å^2×10^3) for **3s’**.

Atom	x	y	z	U(eq)
H1	1232.92	5749.65	3849.93	76
H2	214.15	5037.02	6883.59	96
H3	938.57	4742.79	8434.94	108
H5	1198.9	8508.97	7554.73	102
H6	446.89	8856.98	6005.87	84
H7A	742.78	6603.41	9855.63	179
H7B	2149.72	6987.56	9047.12	179
H7C	2239.3	5441.35	9299.84	179
H9	6193.13	8926.57	456.38	94
H10	8400.81	9364.48	-215.14	110
H11	9574.43	9213.86	1026.04	109

H12	8474.65	8770.61	2944.86	110
H14A	6222.98	7374.19	4406.61	137
H14B	5477.67	8976.71	4496.66	137
H15A	3461.85	9080.26	4289.01	141
H15B	4097.03	7447.02	4425.21	141
H17A	2320.04	9398.46	2998.71	237
H17B	3291.45	9524.72	1707.05	237
H17C	3273.08	10361.09	2545.73	237
H21	2788.3	5852.15	1416.9	114
H22	3431.72	3782.13	765.22	144
H23	4879.6	1756.6	1539.02	140
H24	5591.25	1732.81	2998.87	128
H25	5021.27	3786.89	3646.61	100

Supplementary Table 16. Atomic Occupancy for **3s'**.

tom	Occupancy	Atom	Occupancy	Atom	Occupancy
F1	0.5	F2	0.5	F1'	0.5
F2'	0.5				

Structure confirmation of product 8k

Data intensity of **8k** was collected using a XtaLAB PRO MM003 (Cu radiation) at 100.00(10) K in a nitrogen stream. Data collection and reduction were done by using the XtaLAB software package. The structures were solved by direct methods and refined by full-matrix least-squares on F^2 with anisotropic displacement parameters for non-H atoms using SHELX-97. Hydrogen atoms were added at their geometrically idea positions and refined isotropically. Crystal data for **8k**: $C_{23}H_{18}F_2O$, T = 100.00(10) K, monoclinic, $P2_1/c$, a = 12.01170(10) Å, b = 5.55720(10) Å, c = 26.2897(2) Å, α = 90°, β = 94.8460(10) °, γ = 90°. V = 1748.60(4) Å^3. Z = 4, ρ_{calc} = 1.323 g/cm^3. Reflections collected 37845; independent reflections 3558 [R_{int} = 0.0600, R_{sigma} = 0.0273], R_1 = 0.0405, wR_2 = 0.0950 (all data), GOF = 1.061, and 235 parameter.

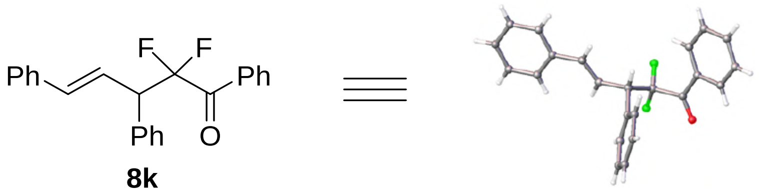

Supplementary Table 17. Crystal data and structure refinement for **8k**.

Identification code	**8k**
Empirical formula	$C_{23}H_{18}F_2O$
Formula weight	348.37
Temperature/K	100.00(10)
Crystal system	monoclinic
Space group	$P2_1/c$
a/Å	12.01170(10)
b/Å	5.55720(10)
c/Å	26.2897(2)
α/°	90
β/°	94.8460(10)
γ/°	90
Volume/Å^3	1748.60(4)
Z	4
ρ_{calc}g/cm^3	1.323
μ/mm^{-1}	0.773
F(000)	728.0
Crystal size/mm^3	0.32 × 0.26 × 0.24
Radiation	CuKα (λ = 1.54184)
2Θ range for data collection/°	7.386 to 149.082
Index ranges	$-15 \le h \le 15$, $-6 \le k \le 6$, $-32 \le l \le 32$
Reflections collected	37845
Independent reflections	3558 [R_{int} = 0.0600, R_{sigma} = 0.0273]
Data/restraints/parameters	3558/0/235
Goodness-of-fit on F^2	1.061

Final R indexes [I>=2σ (I)]	R_1 = 0.0372, wR_2 = 0.0928
Final R indexes [all data]	R_1 = 0.0405, wR_2 = 0.0950
Largest diff. peak/hole / e Å^{-3}	0.23/-0.21

Supplementary Table 18. Fractional Atomic Coordinates (×10^4) and Equivalent Isotropic Displacement Parameters (Å^2×10^3) for **8k**. U_{eq} is defined as 1/3 of of the trace of the orthogonalised U_{IJ} tensor.

Atom	*x*	*y*	*z*	U(eq)
F1	3337.4(6)	-1424.4(13)	6719.6(3)	23.41(18)
F2	4288.4(6)	959.1(14)	7246.1(3)	24.40(18)
O1	1610.1(7)	-825.9(16)	7216.3(3)	23.0(2)
C1	6448.9(11)	6476(3)	5801.1(5)	28.7(3)
C2	7254.6(12)	6677(3)	5452.4(6)	34.0(3)
C3	7323.1(11)	4960(3)	5076.6(5)	29.7(3)
C4	6596.3(12)	3035(3)	5056.9(5)	33.7(3)
C5	5792.7(12)	2828(3)	5402.3(5)	29.5(3)
C6	5703.1(10)	4563(2)	5779.5(4)	19.5(3)
C7	4836.8(10)	4461(2)	6144.5(4)	19.4(3)
C8	4132.6(10)	2667(2)	6200.7(5)	20.3(3)
C9	3196.8(9)	2768(2)	6550.0(4)	17.8(2)
C10	3291.8(10)	774(2)	6950.9(4)	18.5(3)
C11	2301.6(9)	747(2)	7292.7(4)	17.5(2)
C12	2197.0(10)	2719(2)	7667.6(4)	17.7(2)
C13	1167.9(10)	2953(2)	7878.5(5)	22.7(3)
C14	1011.7(11)	4768(3)	8225.6(5)	26.3(3)
C15	1870.8(11)	6352(3)	8370.3(5)	24.8(3)
C16	2888.6(11)	6147(3)	8161.8(5)	23.9(3)
C17	3053.7(10)	4345(2)	7811.1(4)	20.7(3)
C18	2059.4(10)	2686(2)	6243.0(4)	17.3(2)

C19	1301.2(10)	4558(2)	6284.5(5)	20.3(3)
C20	279.7(10)	4542(2)	5990.5(5)	23.4(3)
C21	7.4(11)	2659(3)	5658.1(5)	24.4(3)
C22	750.9(11)	769(2)	5621.4(5)	23.4(3)
C23	1772.6(10)	782(2)	5911.5(4)	20.4(3)

Supplementary Table 19. Anisotropic Displacement Parameters (Å^2×10^3) for **8k**. The Anisotropic displacement factor exponent takes the form: $-2\pi^2[h^2a^{*2}U_{11}+2hka^*b^*U_{12}+\ldots]$.

Atom	U_{11}	U_{22}	U_{33}	U_{23}	U_{13}	U_{12}
F1	28.3(4)	14.4(4)	28.2(4)	-2.3(3)	6.4(3)	4.3(3)
F2	15.8(3)	29.7(4)	27.4(4)	-0.2(3)	-0.2(3)	4.7(3)
O1	24.4(4)	20.4(5)	24.2(4)	-0.5(4)	2.3(3)	-4.5(4)
C1	30.1(7)	23.8(7)	33.2(7)	-1.2(6)	9.4(6)	-3.4(6)
C2	29.0(7)	32.2(8)	42.2(8)	7.7(7)	11.9(6)	-6.0(6)
C3	23.2(6)	39.8(9)	27.6(7)	14.2(6)	10.5(5)	7.1(6)
C4	35.2(7)	40.8(9)	26.8(7)	-4.8(6)	13.2(6)	2.0(7)
C5	30.1(7)	31.3(8)	28.6(7)	-5.6(6)	10.9(5)	-5.5(6)
C6	19.3(6)	20.0(6)	19.3(6)	4.2(5)	2.4(4)	2.9(5)
C7	20.8(6)	18.6(6)	19.1(6)	-1.4(5)	2.6(4)	2.5(5)
C8	21.2(6)	19.4(6)	21.0(6)	-3.4(5)	6.0(5)	0.9(5)
C9	19.2(6)	15.6(6)	19.1(5)	-1.8(5)	4.9(4)	-0.1(4)
C10	17.6(5)	16.6(6)	21.1(6)	-3.0(5)	0.6(4)	2.1(4)
C11	18.0(5)	17.2(6)	17.0(5)	3.2(5)	-0.1(4)	1.5(4)
C12	17.9(5)	19.7(6)	15.3(5)	2.3(5)	1.1(4)	1.8(5)
C13	19.6(6)	25.4(7)	23.3(6)	-0.8(5)	3.1(5)	-2.4(5)
C14	22.8(6)	31.5(8)	25.4(6)	-2.2(6)	7.1(5)	3.2(5)
C15	29.1(7)	25.1(7)	20.2(6)	-5.0(5)	2.1(5)	3.3(5)
C16	23.7(6)	26.2(7)	21.3(6)	-2.8(5)	-1.5(5)	-3.0(5)
C17	18.6(6)	24.4(7)	19.2(6)	-0.4(5)	1.7(4)	0.0(5)

C18	20.2(6)	16.6(6)	15.8(5)	2.8(4)	5.9(4)	-0.8(4)
C19	23.3(6)	17.6(6)	20.4(6)	-0.2(5)	5.1(5)	-0.2(5)
C20	22.0(6)	22.0(7)	26.5(6)	3.8(5)	4.3(5)	3.1(5)
C21	23.2(6)	28.7(7)	20.8(6)	4.8(5)	-0.5(5)	-1.4(5)
C22	29.8(6)	23.3(7)	16.9(6)	-1.5(5)	1.7(5)	-3.1(5)
C23	24.5(6)	19.3(6)	18.0(6)	0.1(5)	5.1(5)	1.4(5)

Supplementary Table 20. Bond Lengths for **8k**.

Atom	Atom	Length/Å	Atom	Atom	Length/Å
F1	C10	1.3678(14)	C10	C11	1.5502(16)
F2	C10	1.3745(13)	C11	C12	1.4864(17)
O1	C11	1.2109(15)	C12	C13	1.4028(16)
C1	C2	1.3926(19)	C12	C17	1.3969(17)
C1	C6	1.3885(18)	C13	C14	1.3834(18)
C2	C3	1.381(2)	C14	C15	1.3850(19)
C3	C4	1.379(2)	C15	C16	1.3861(18)
C4	C5	1.3848(18)	C16	C17	1.3871(18)
C5	C6	1.3936(18)	C18	C19	1.3930(17)
C6	C7	1.4750(16)	C18	C23	1.3953(17)
C7	C8	1.3238(18)	C19	C20	1.3940(17)
C8	C9	1.5114(15)	C20	C21	1.3847(19)
C9	C10	1.5268(17)	C21	C22	1.3872(19)
C9	C18	1.5277(16)	C22	C23	1.3892(18)

Supplementary Table 21. Bond Angles for **8k**.

Atom	Atom	Atom	Angle/˚	Atom	Atom	Atom	Angle/˚
C6	C1	C2	121.07(13)	O1	C11	C10	117.38(11)
C3	C2	C1	120.16(14)	O1	C11	C12	123.17(11)

C4	C3	C2	119.15(12)	C12	C11	C10	119.30(10)
C3	C4	C5	120.94(14)	C13	C12	C11	117.19(11)
C4	C5	C6	120.59(13)	C17	C12	C11	123.65(10)
C1	C6	C5	118.07(11)	C17	C12	C13	119.16(11)
C1	C6	C7	119.40(11)	C14	C13	C12	120.08(12)
C5	C6	C7	122.51(12)	C13	C14	C15	120.34(12)
C8	C7	C6	126.45(12)	C14	C15	C16	120.06(12)
C7	C8	C9	123.70(11)	C15	C16	C17	120.16(12)
C8	C9	C10	112.11(10)	C16	C17	C12	120.20(11)
C8	C9	C18	110.83(9)	C19	C18	C9	119.84(11)
C10	C9	C18	110.75(10)	C19	C18	C23	119.13(11)
F1	C10	F2	104.60(9)	C23	C18	C9	121.02(11)
F1	C10	C9	110.18(9)	C18	C19	C20	120.17(12)
F1	C10	C11	108.09(10)	C21	C20	C19	120.31(12)
F2	C10	C9	110.26(10)	C20	C21	C22	119.79(12)
F2	C10	C11	110.34(9)	C21	C22	C23	120.14(12)
C9	C10	C11	113.01(9)	C22	C23	C18	120.45(12)

Supplementary Table 22. Hydrogen Atom Coordinates (Å×10^4) and Isotropic Displacement Parameters (Å^2×10^3) for **8k**.

Atom	*x*	*y*	*z*	U(eq)
H1	6409.85	7642.83	6052.6	34
H2	7747.63	7970.2	5472.6	41
H3	7852.63	5100.28	4839.87	36
H4	6646.52	1859.83	4808.13	40
H5	5308.4	1519.39	5382.39	35
H7	4779.61	5788.5	6356.05	23

H8	4218.57	1265.17	6014.79	24
H9	3252.24	4312.03	6730.48	21
H13	589.98	1886.14	7784.9	27
H14	326.25	4924.75	8362.42	32
H15	1764.61	7553.78	8607.53	30
H16	3462.31	7221.14	8257.37	29
H17	3736.73	4217.7	7670.96	25
H19	1477.01	5821.06	6509.07	24
H20	-221.3	5802.17	6017.64	28
H21	-671.84	2661.07	5460.04	29
H22	564.97	-508.84	5402.1	28
H23	2268.79	-487.61	5884.64	25

NMR spectra

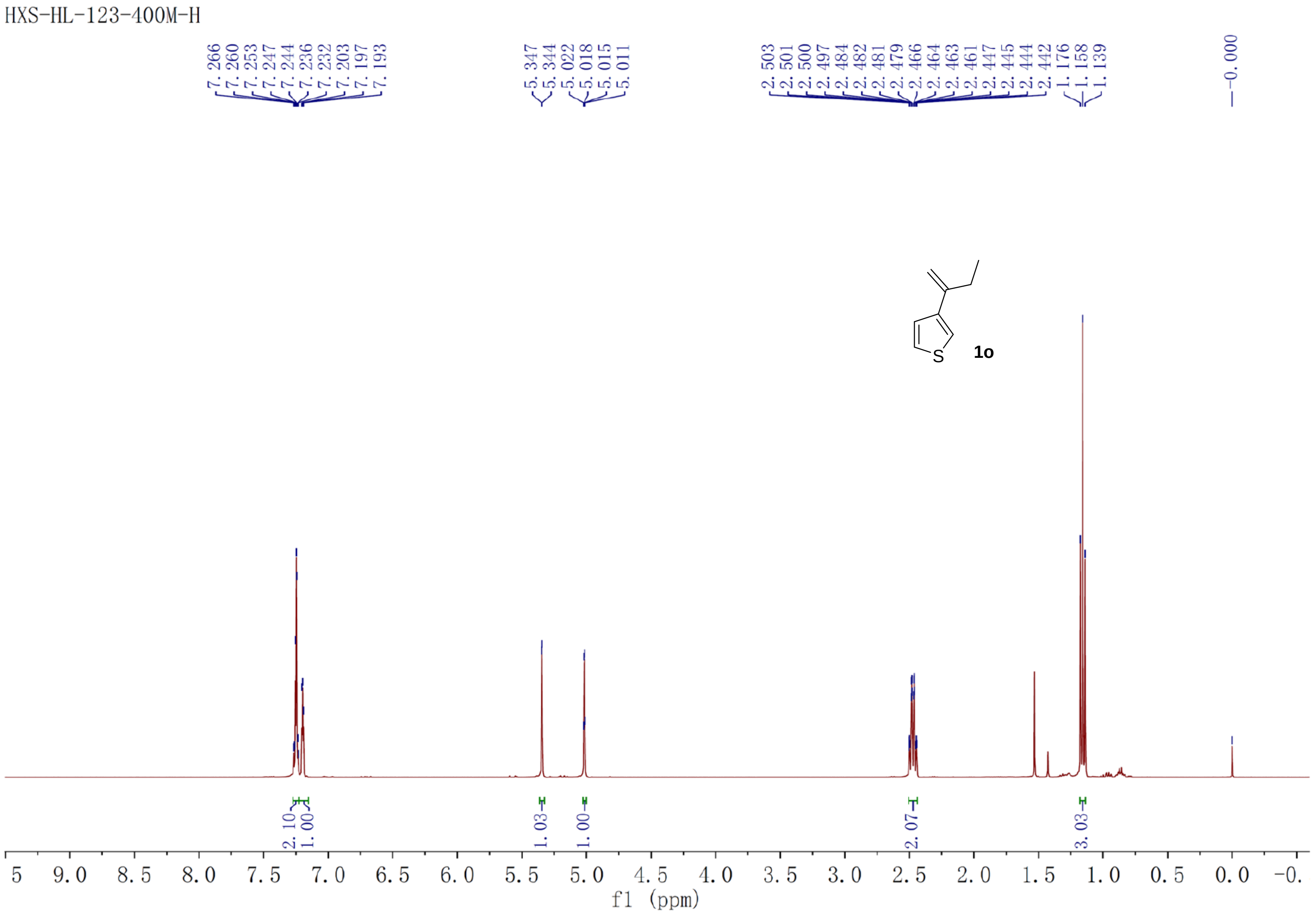

Supplementary Figure 1. ^{1}H NMR (400 MHz, $CDCl_3$) spectra for compound **1o**

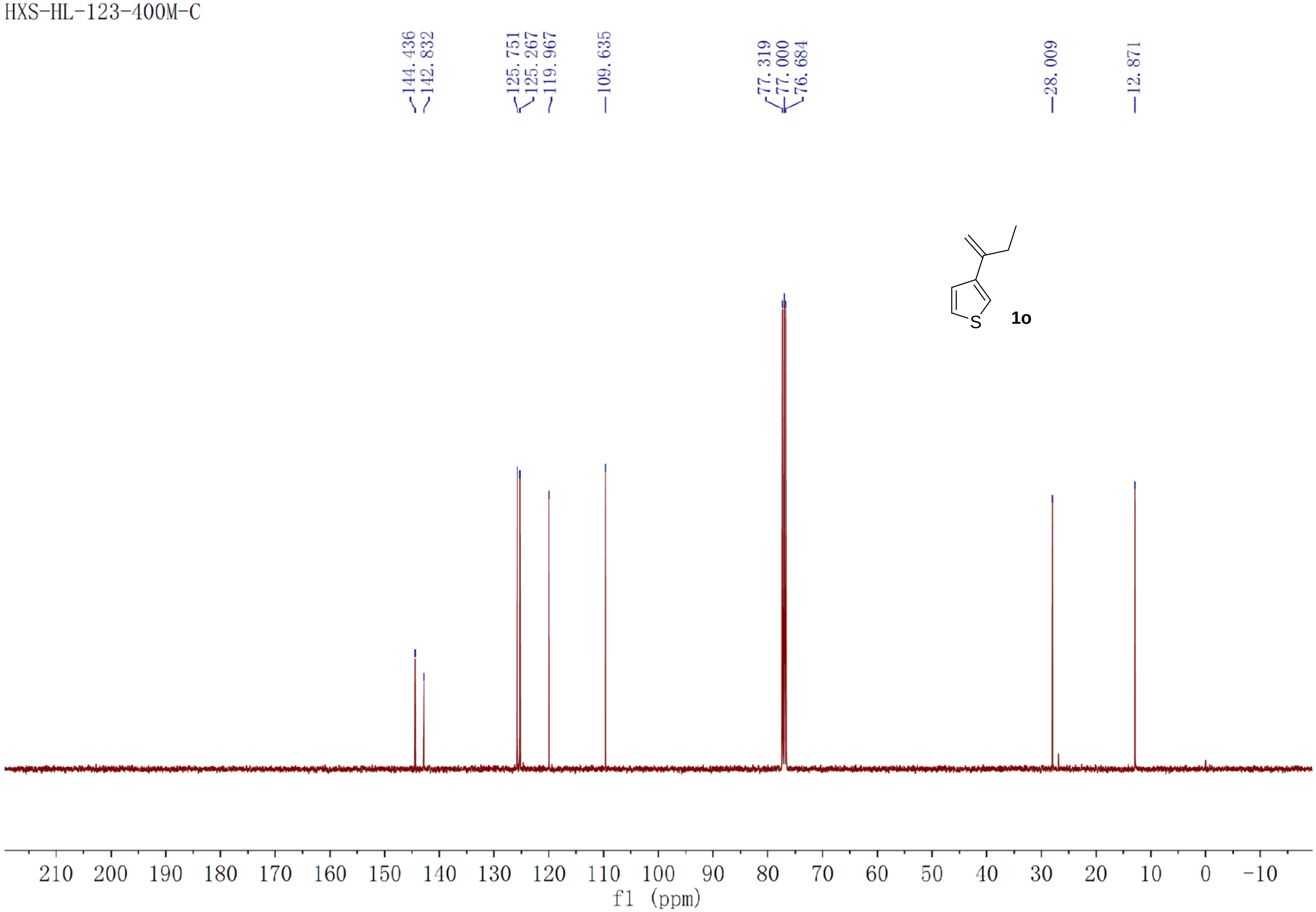

Supplementary Figure 2. ^{13}C NMR (100 MHz, $CDCl_3$) spectra for compound **1o**

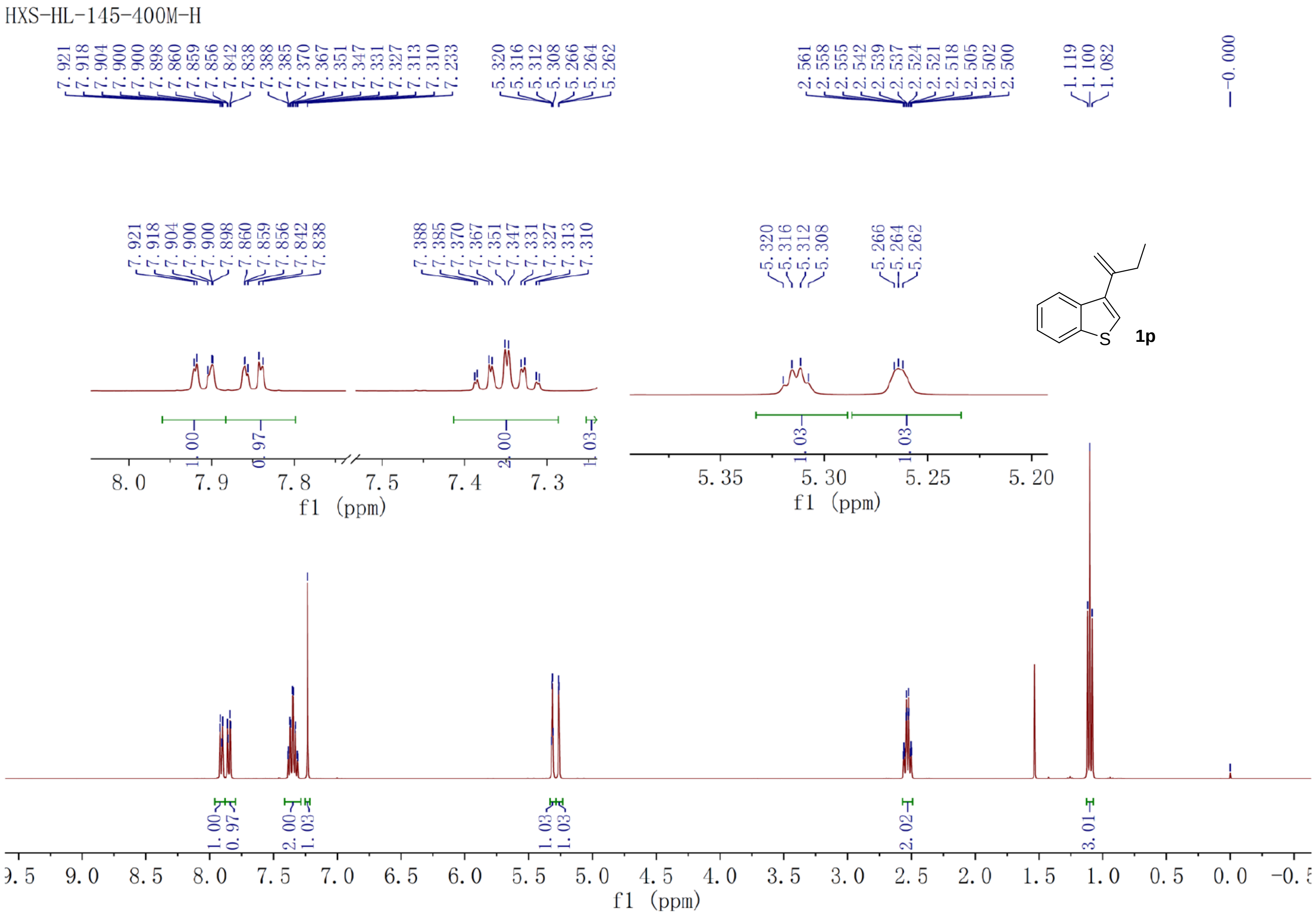

Supplementary Figure 3. ^{1}H NMR (400 MHz, $CDCl_3$) spectra for compound **1p**

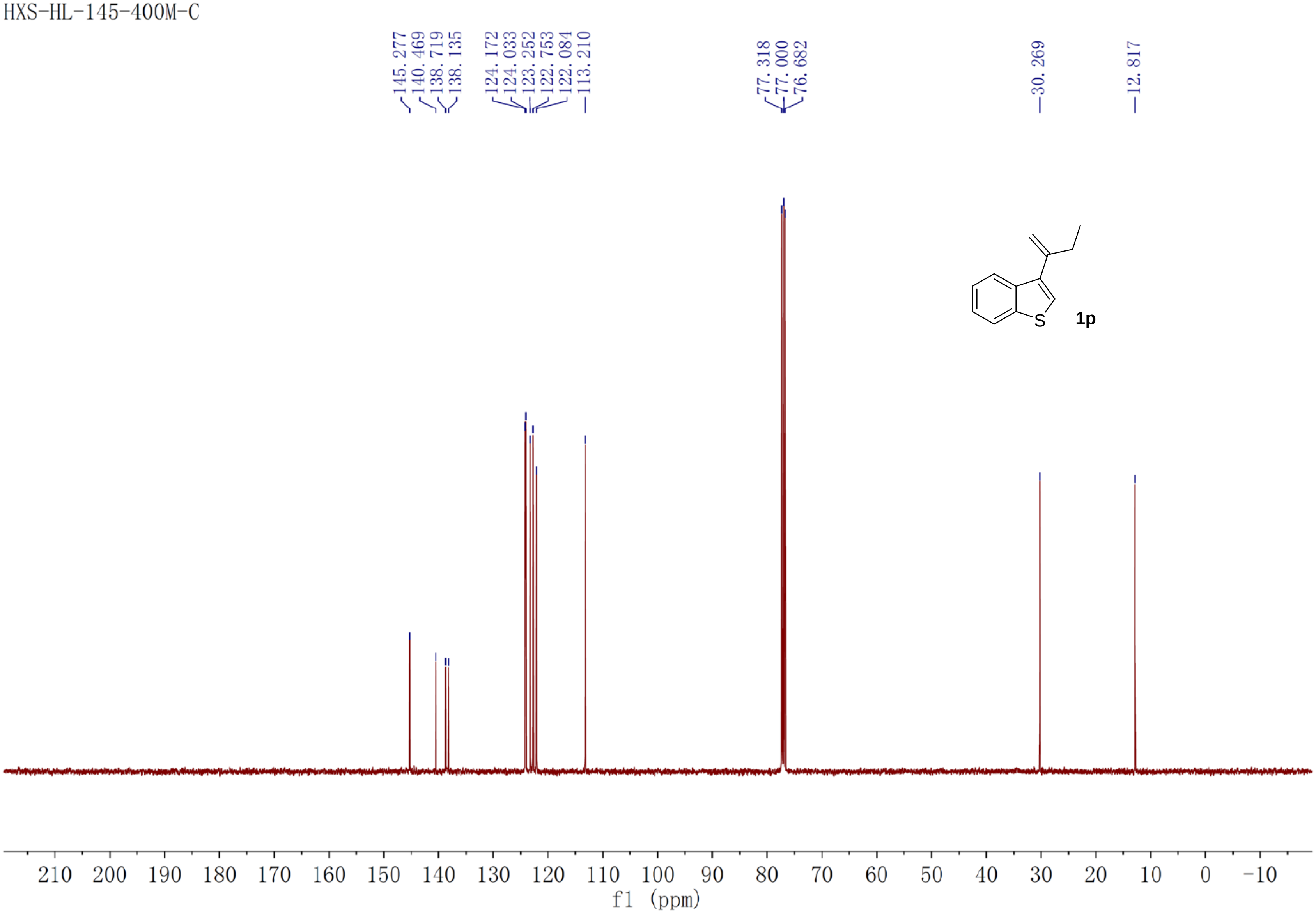

Supplementary Figure 4. ^{13}C NMR (100 MHz, $CDCl_3$) spectra for compound **1p**

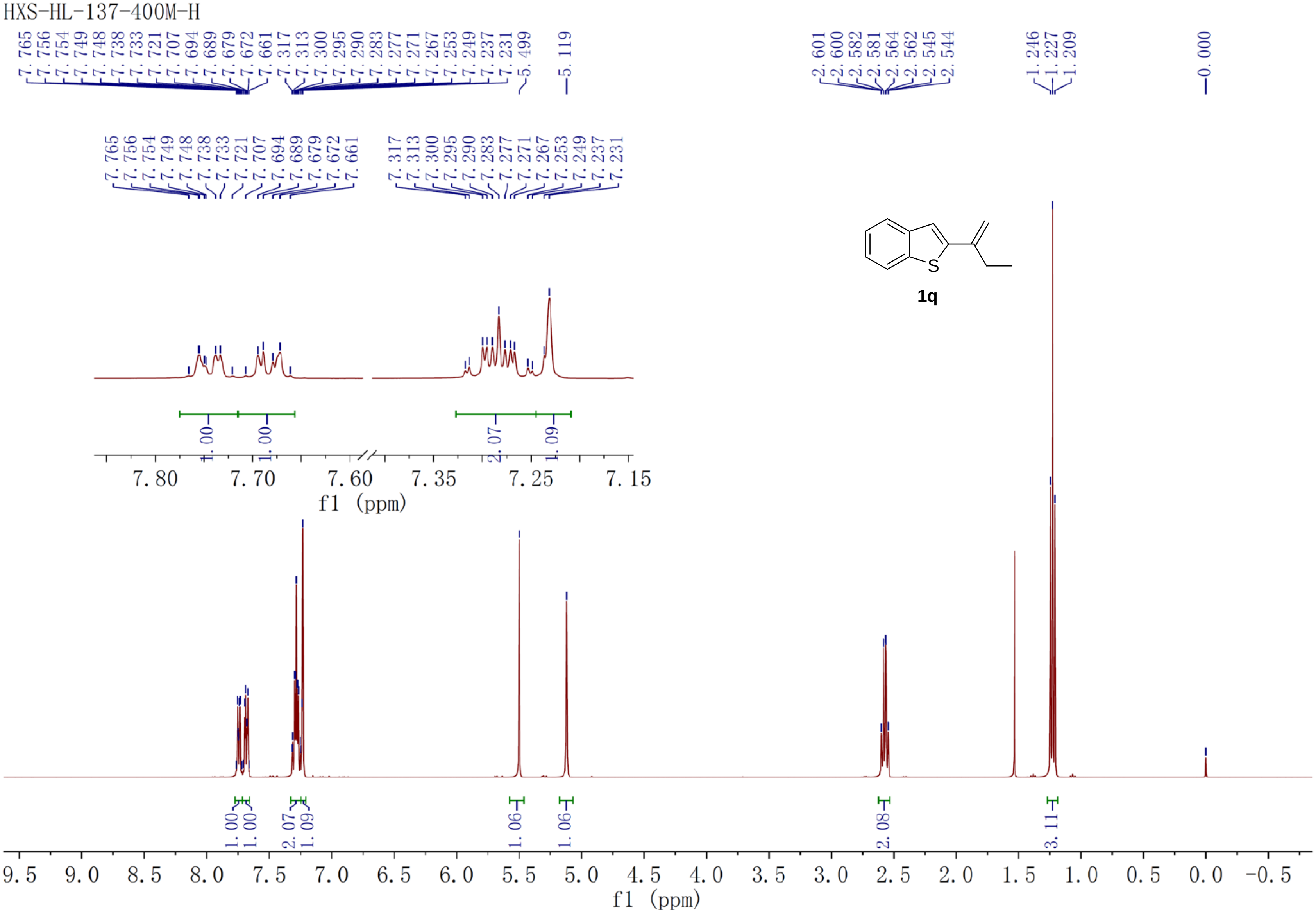

Supplementary Figure 5. ^{1}H NMR (400 MHz, $CDCl_3$) spectra for compound **1q**

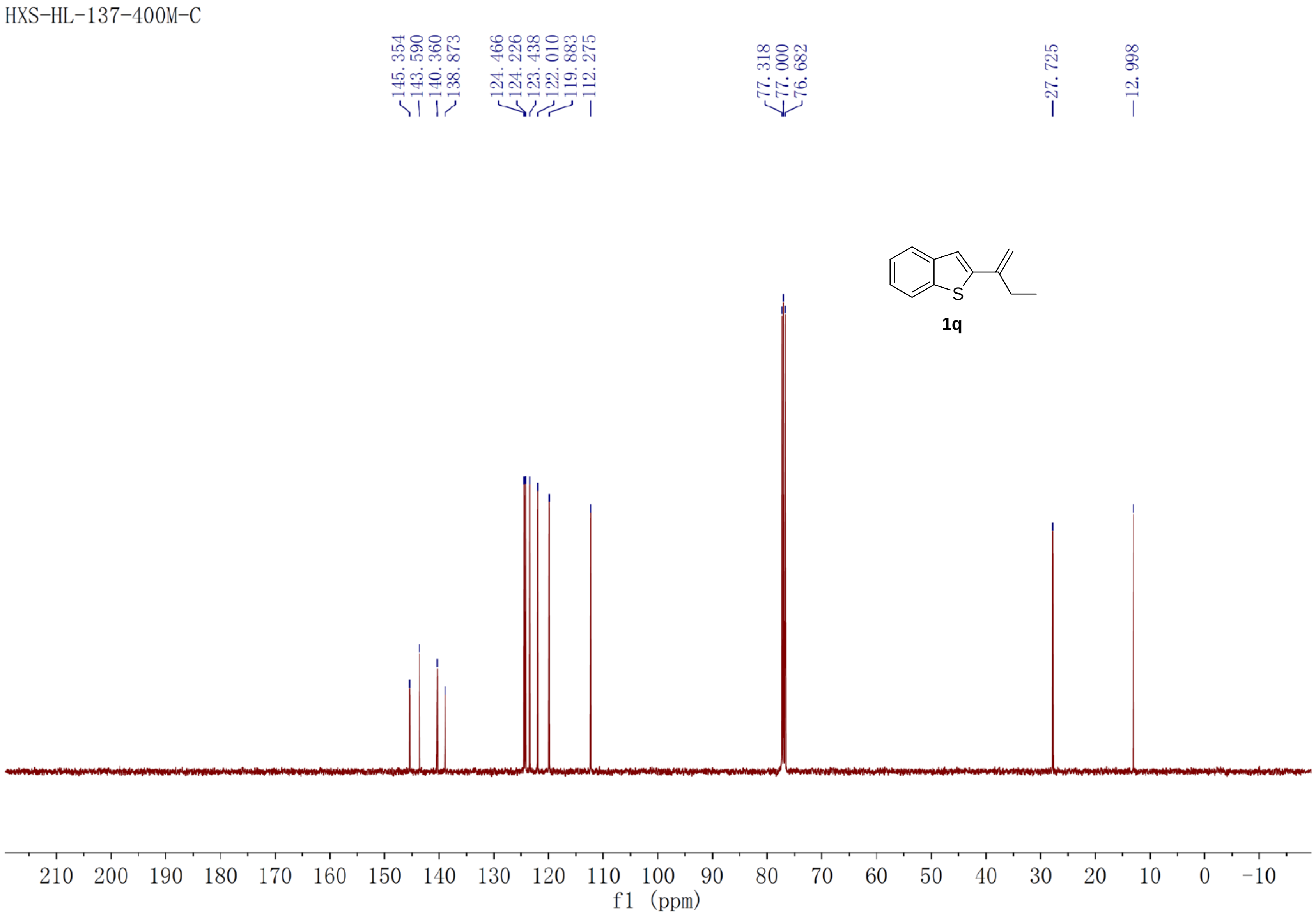

Supplementary Figure 6. ^{13}C NMR (100 MHz, $CDCl_3$) spectra for compound **1q**

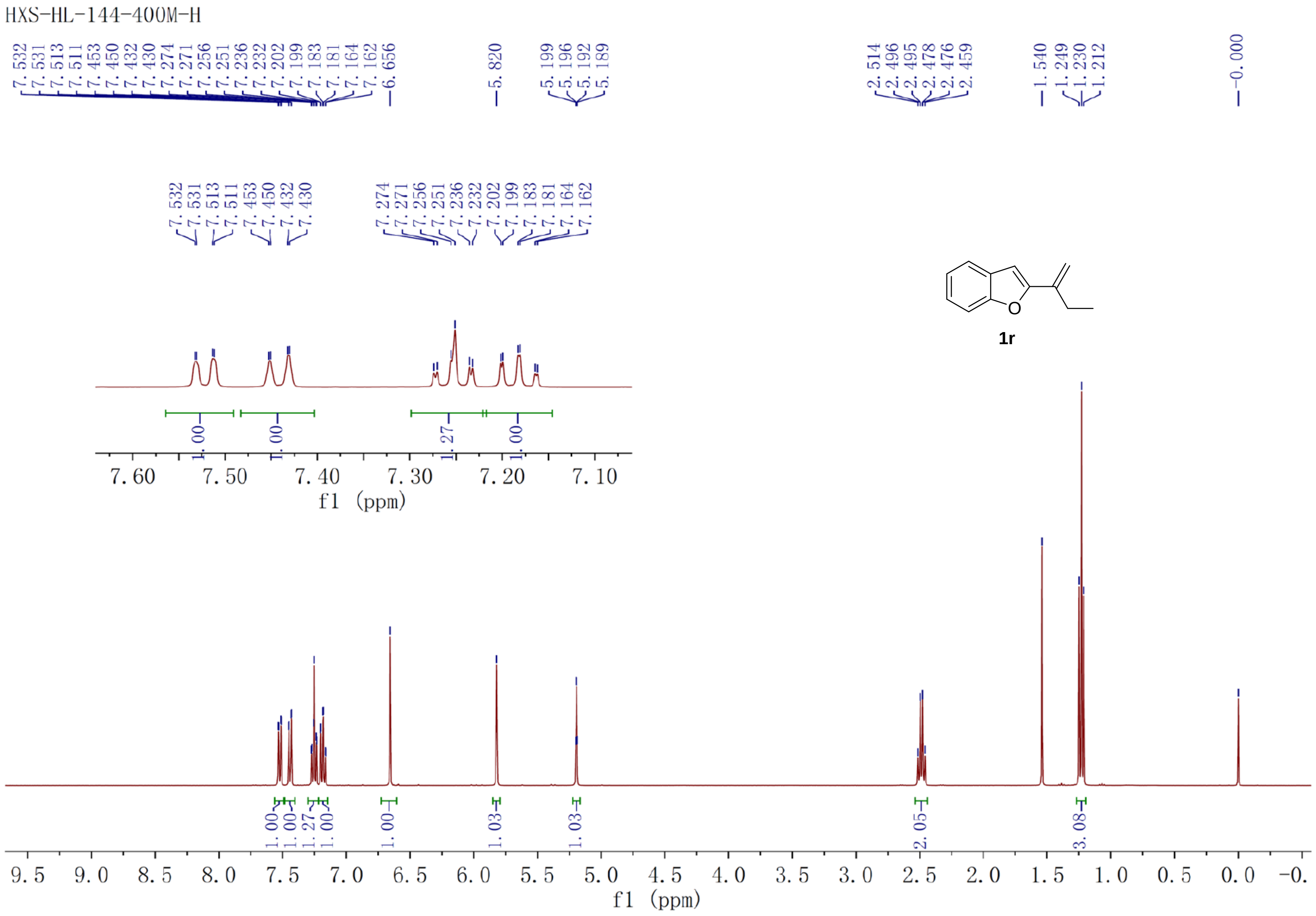

Supplementary Figure 7. ^{1}H NMR (400 MHz, $CDCl_3$) spectra for compound **1r**

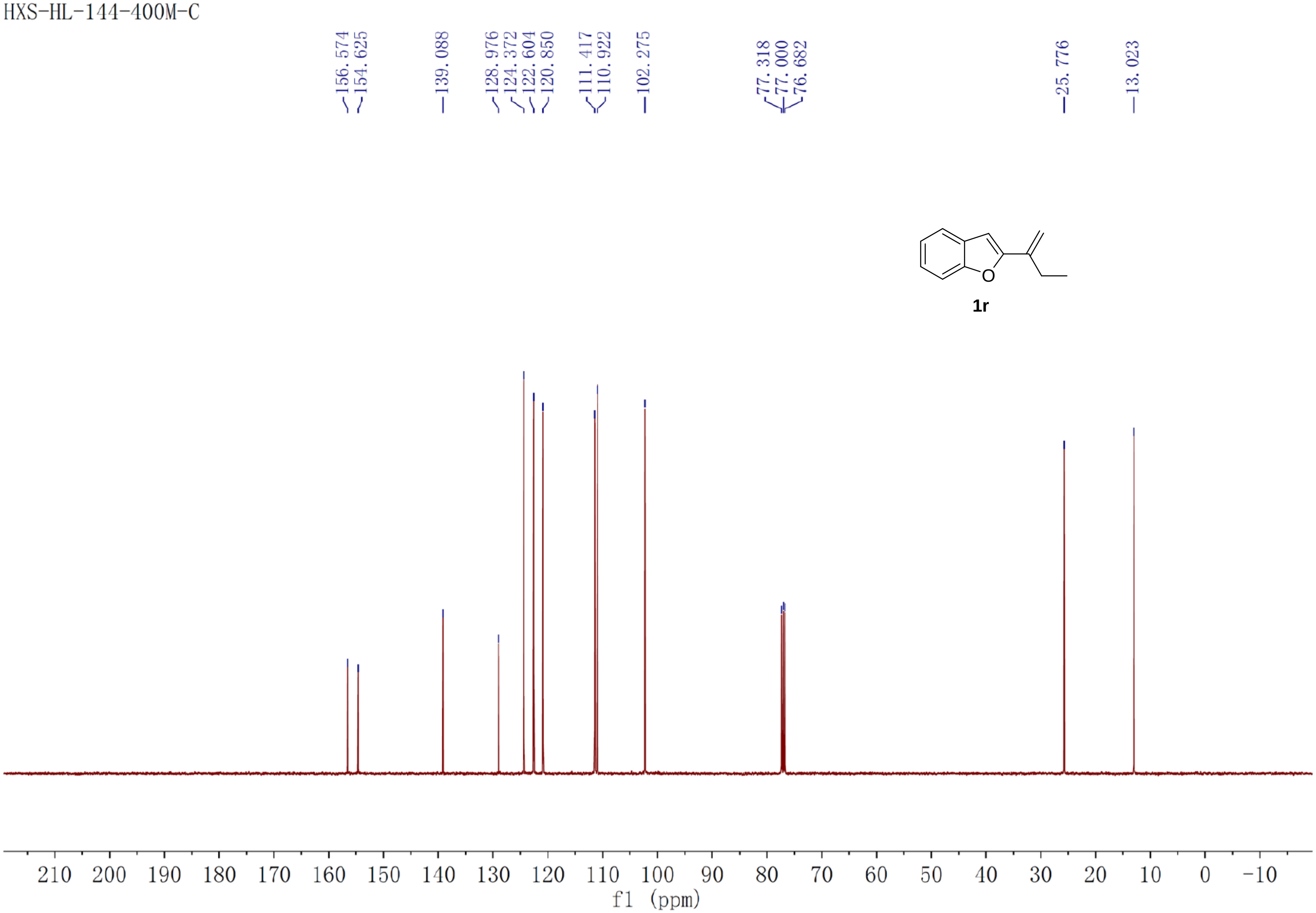

Supplementary Figure 8. ^{13}C NMR (100 MHz, $CDCl_3$) spectra for compound **1r**

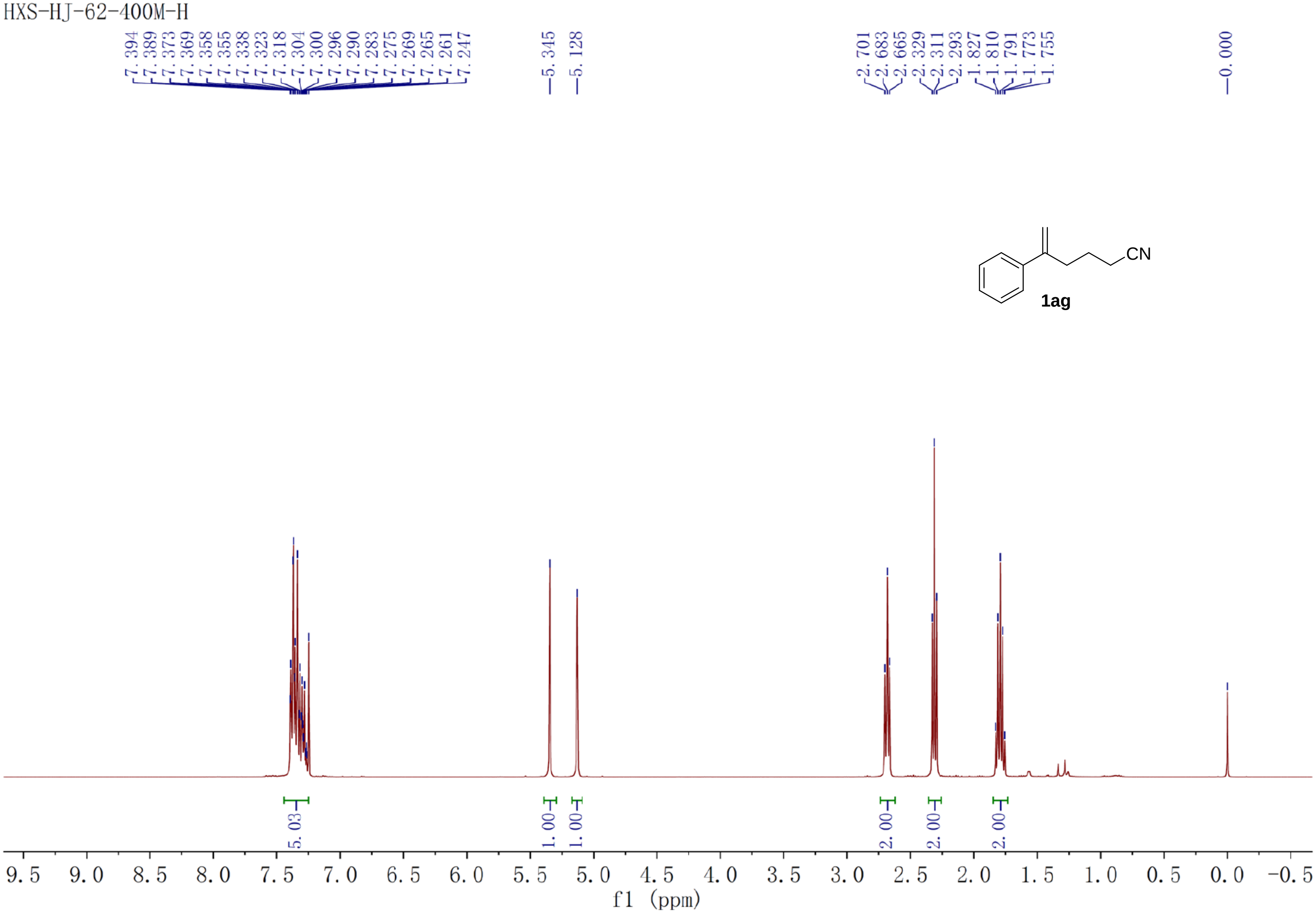

Supplementary Figure 9. ^{1}H NMR (400 MHz, $CDCl_3$) spectra for compound **1ag**

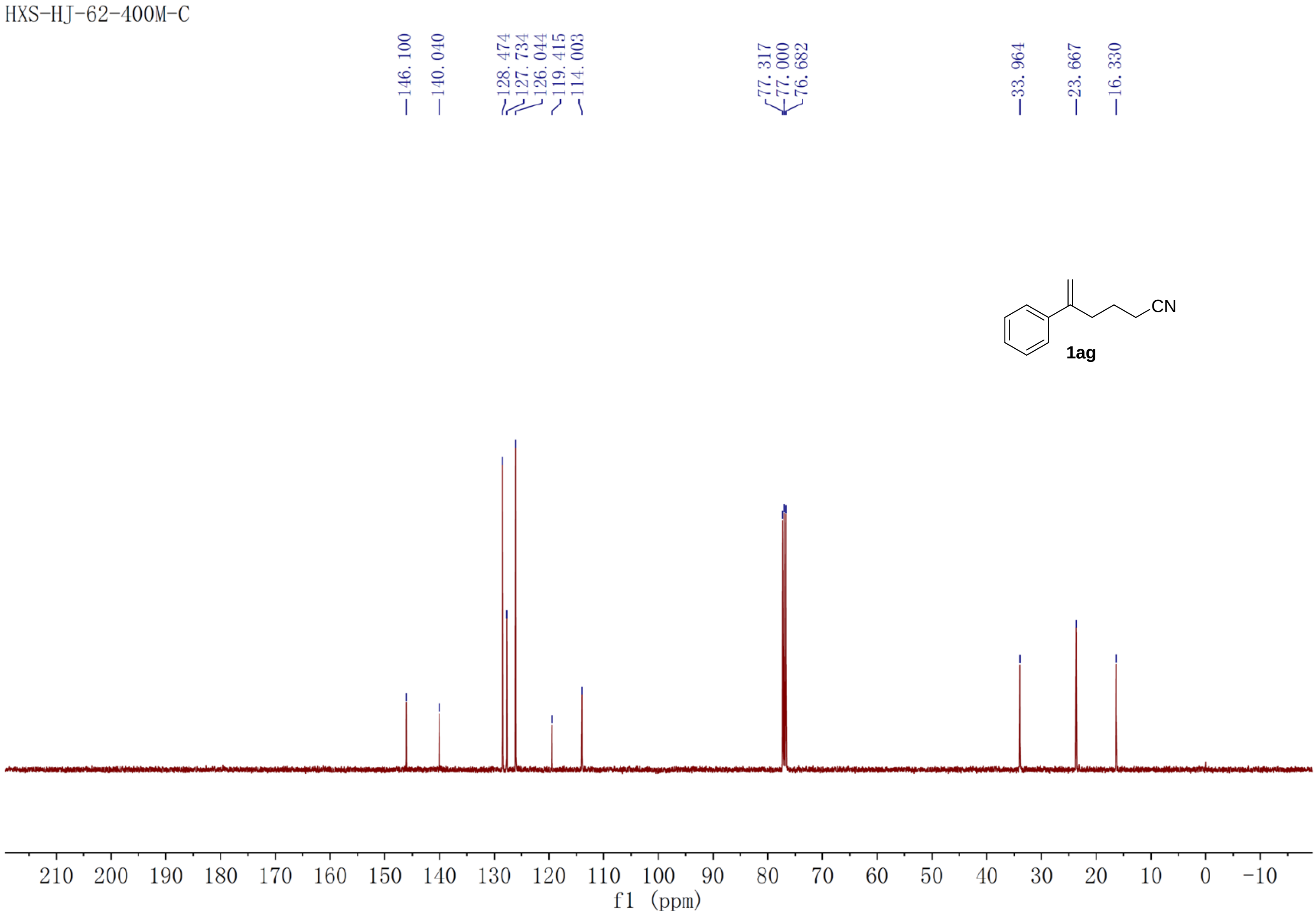

Supplementary Figure 10. ^{13}C NMR (100 MHz, $CDCl_3$) spectra for compound **1ag**

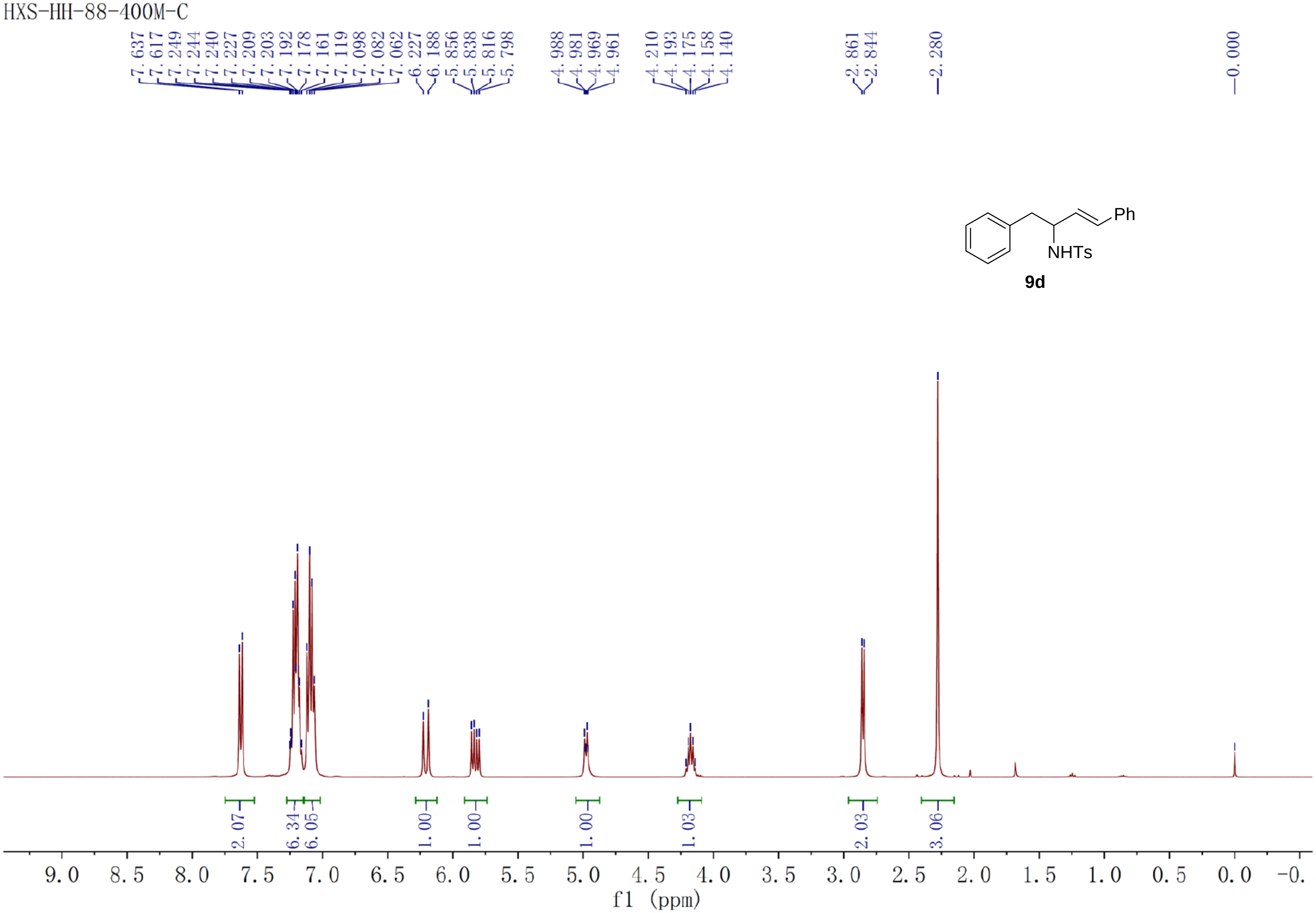

Supplementary Figure 11. ^{1}H NMR (400 MHz, $CDCl_3$) spectra for compound **9d**

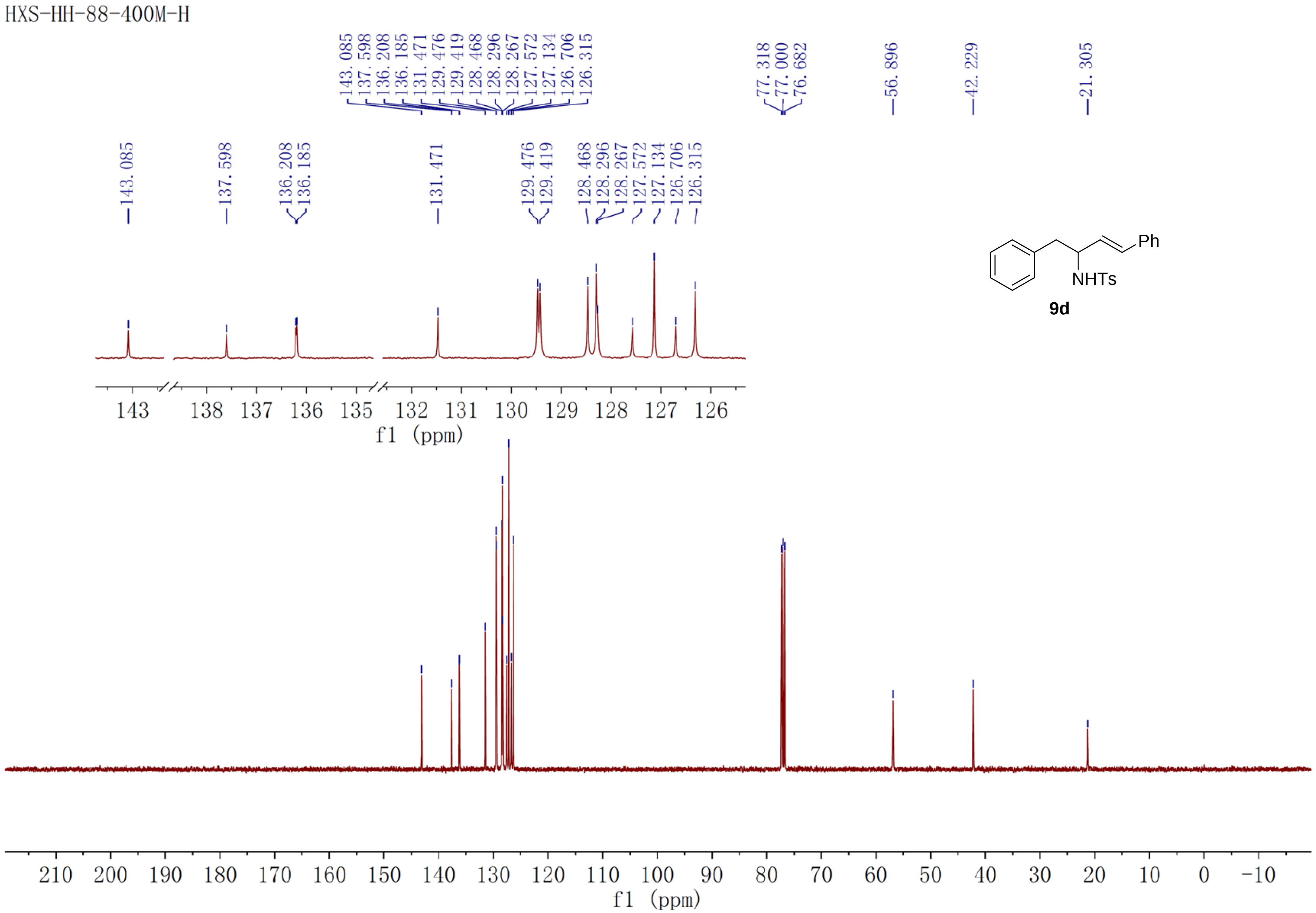

Supplementary Figure 12. ^{13}C NMR (100 MHz, $CDCl_3$) spectra for compound **9d**

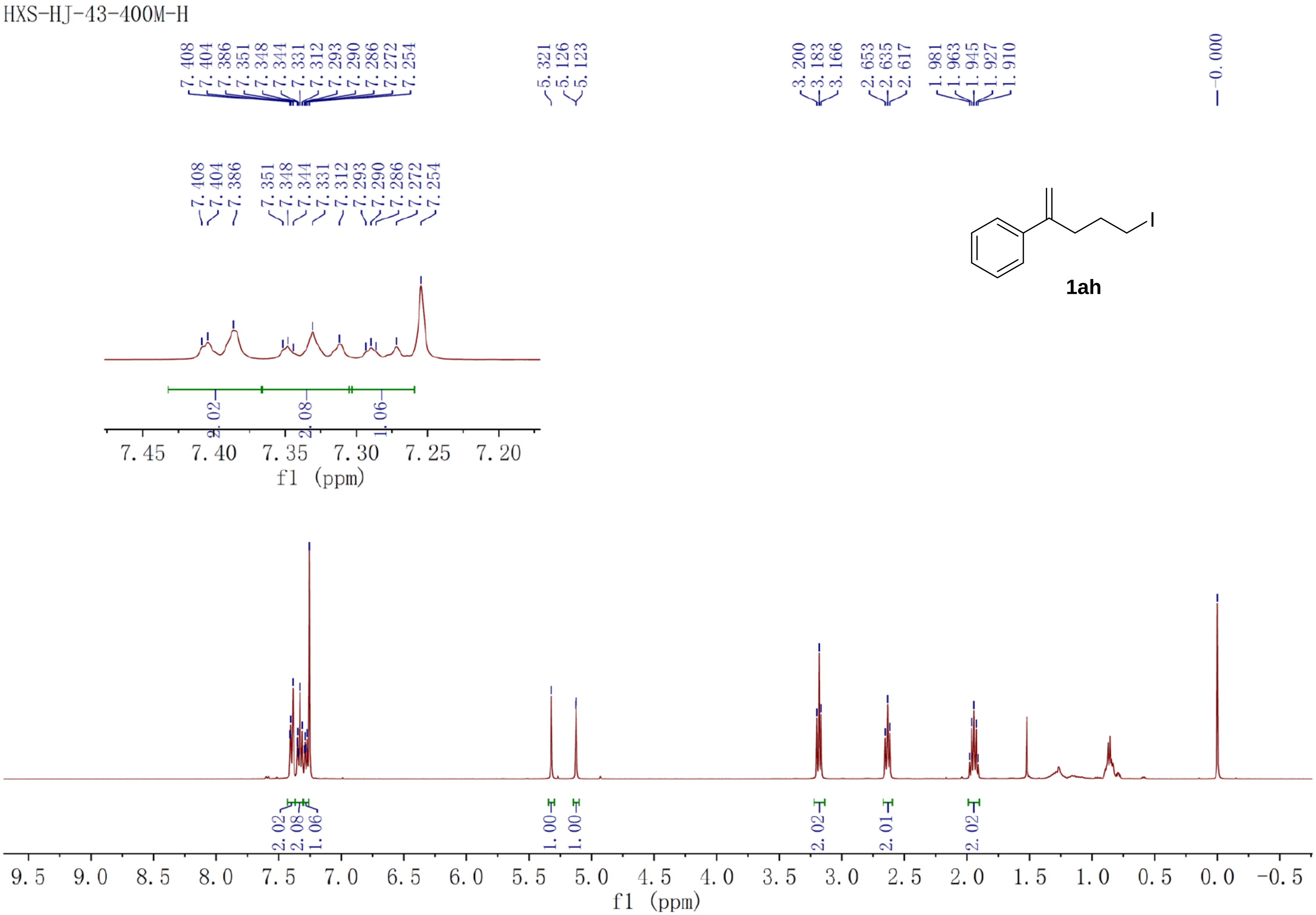

Supplementary Figure 13. ^{1}H NMR (400 MHz, $CDCl_3$) spectra for compound **1ah**

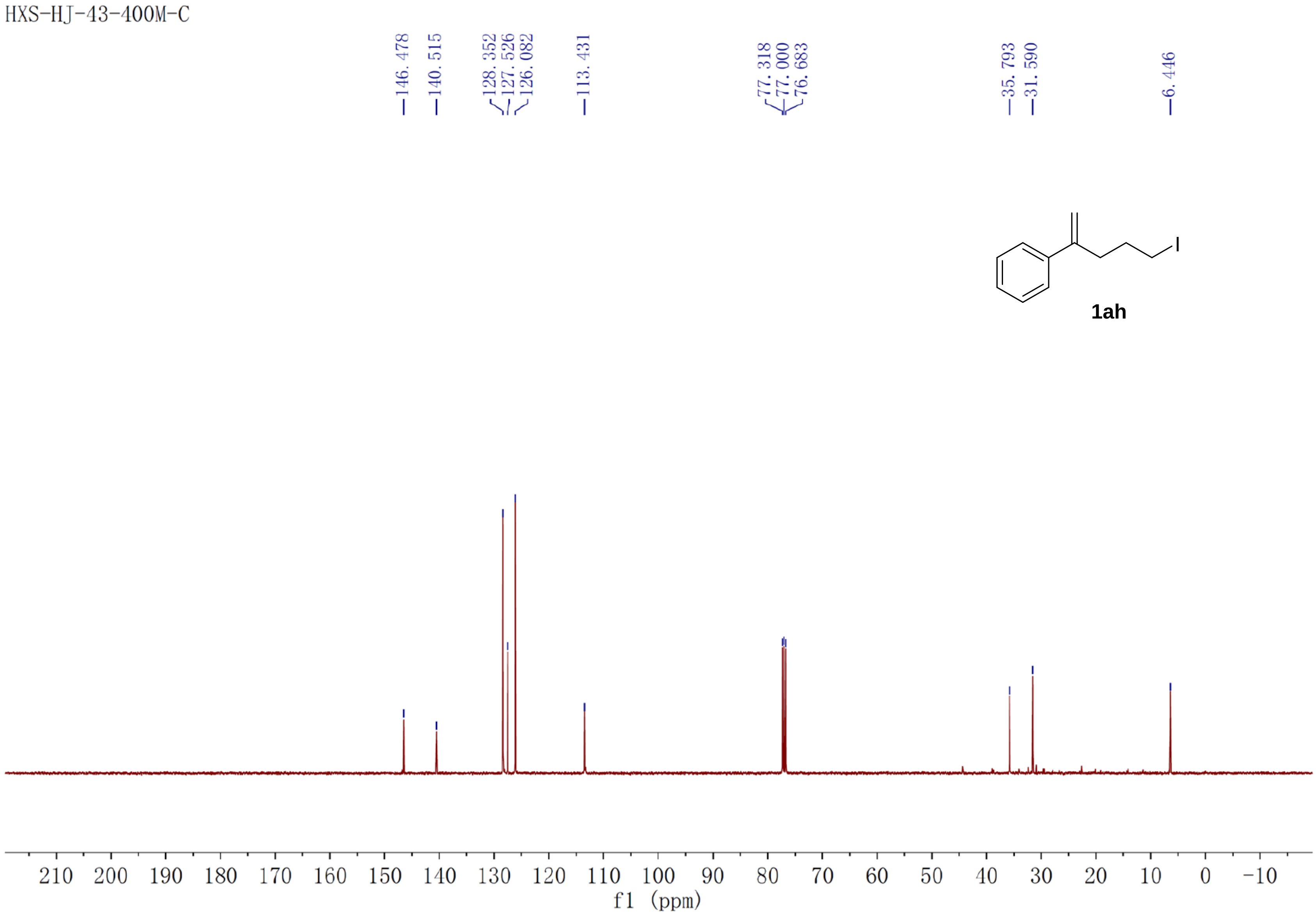

Supplementary Figure 14. ^{13}C NMR (100 MHz, $CDCl_3$) spectra for compound **1ah**

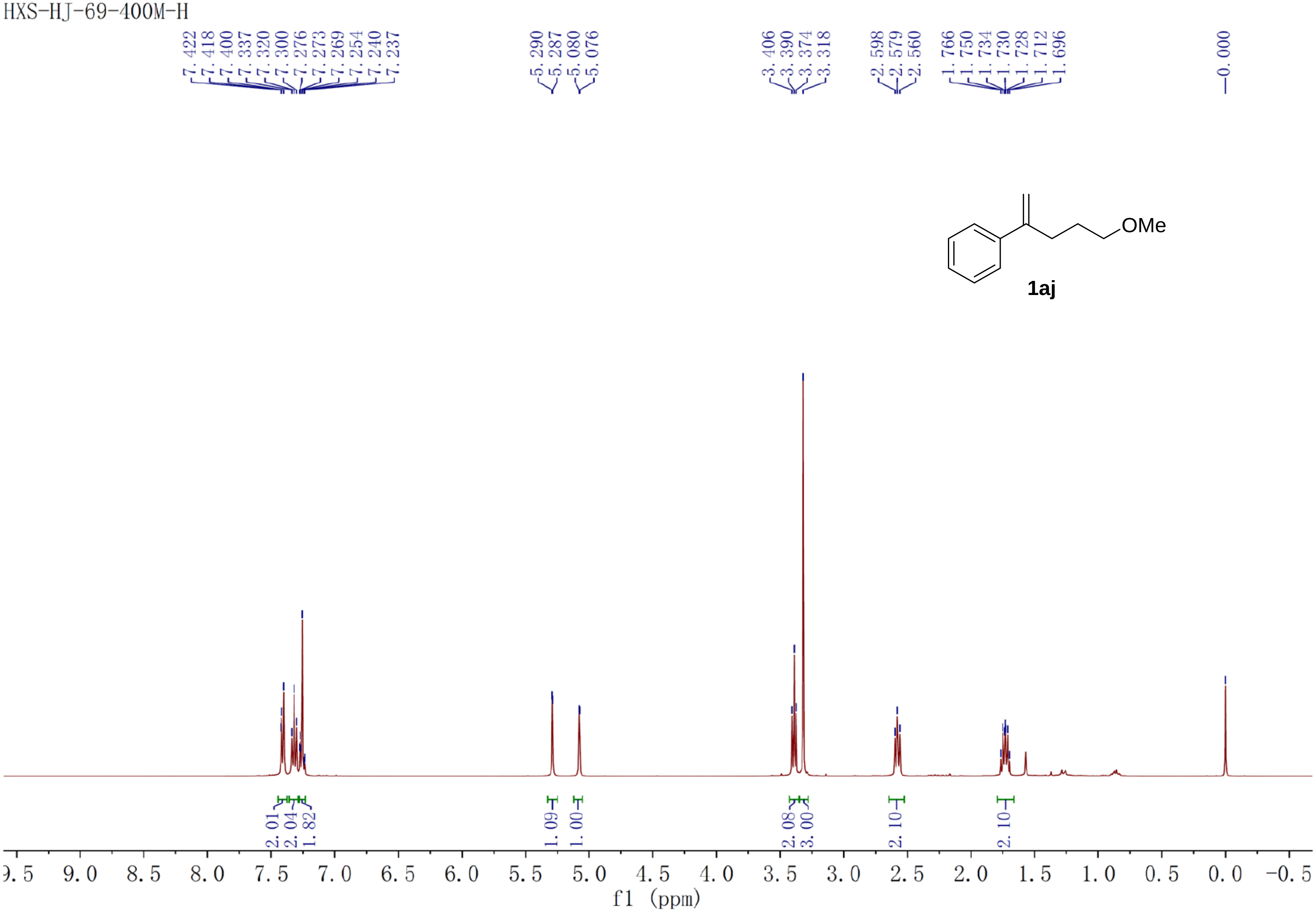

Supplementary Figure 15. ^{1}H NMR (400 MHz, $CDCl_3$) spectra for compound **1aj**

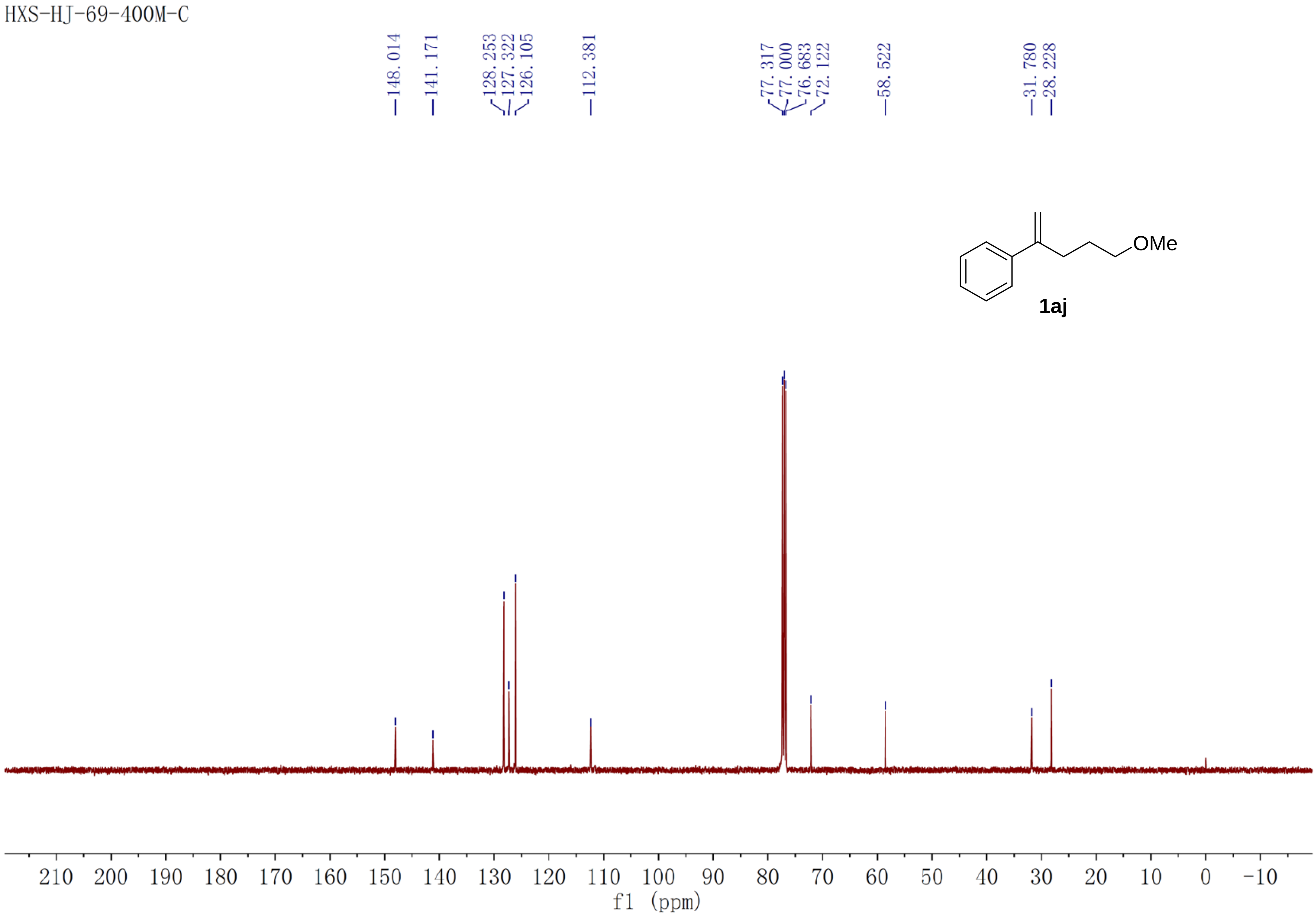

Supplementary Figure 16. ^{13}C NMR (100 MHz, $CDCl_3$) spectra for compound **1aj**

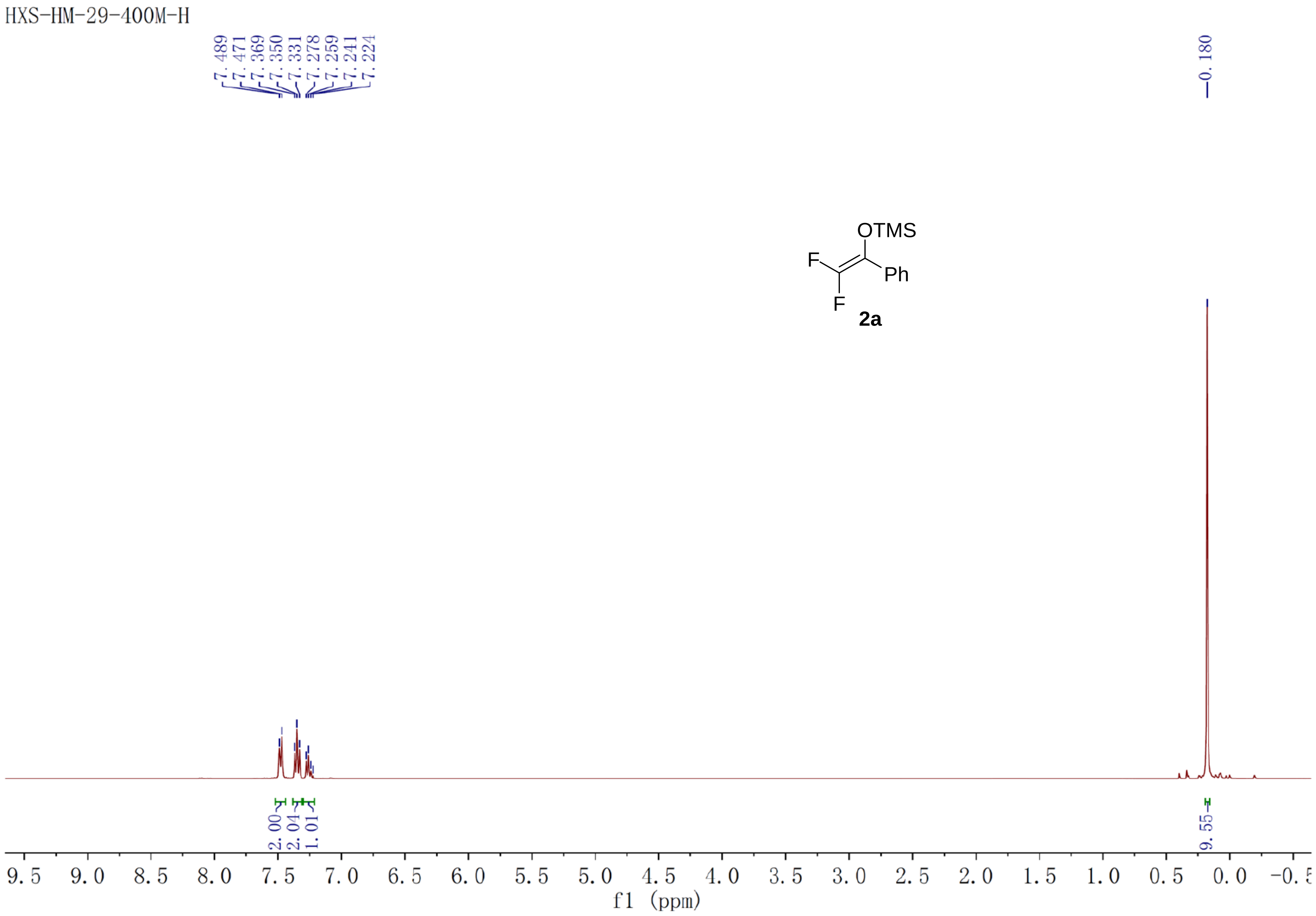

Supplementary Figure 17. ^{1}H NMR (400 MHz, $CDCl_3$) spectra for compound **2a**

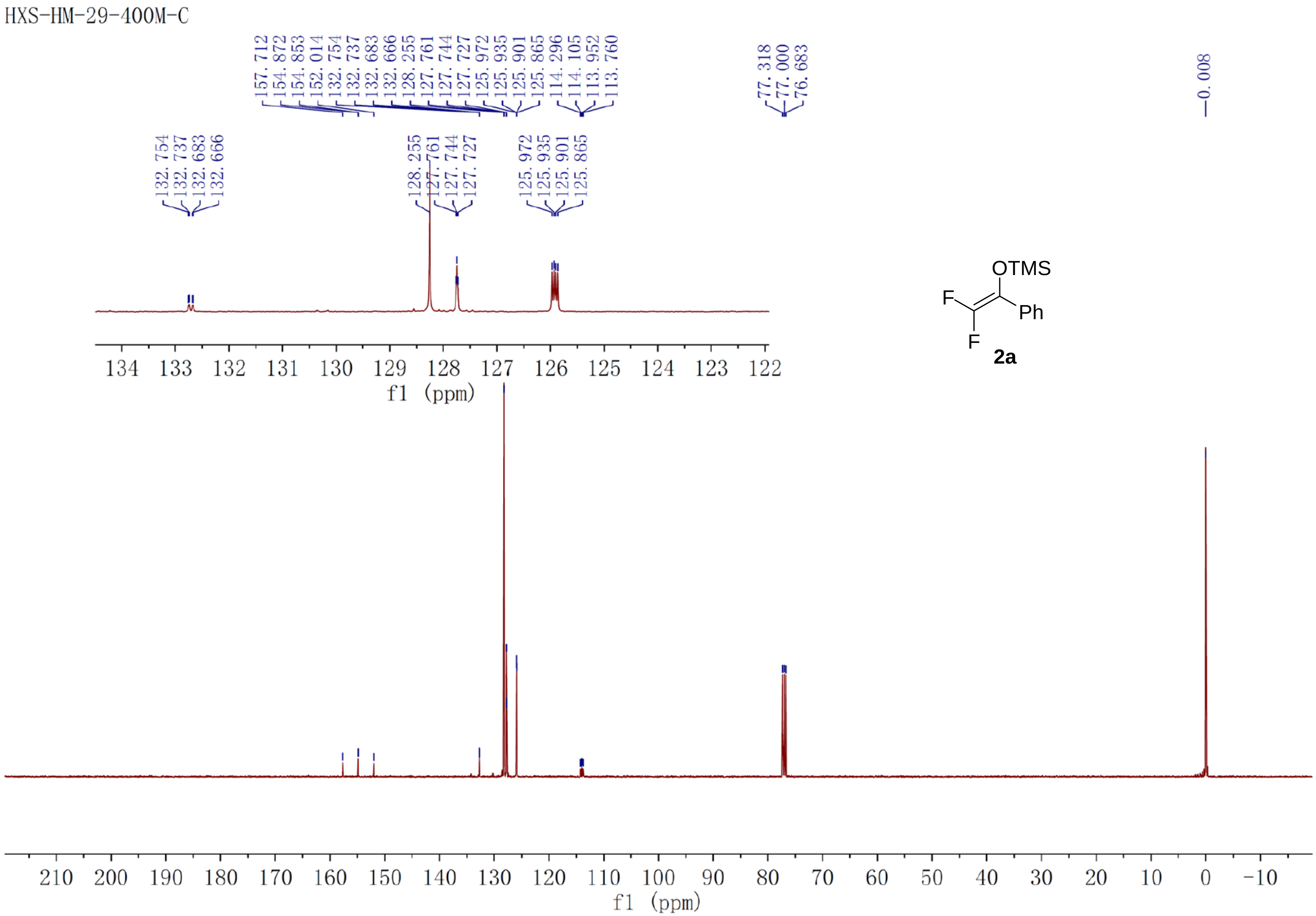

Supplementary Figure 18. ^{13}C NMR (100 MHz, $CDCl_3$) spectra for compound **2a**

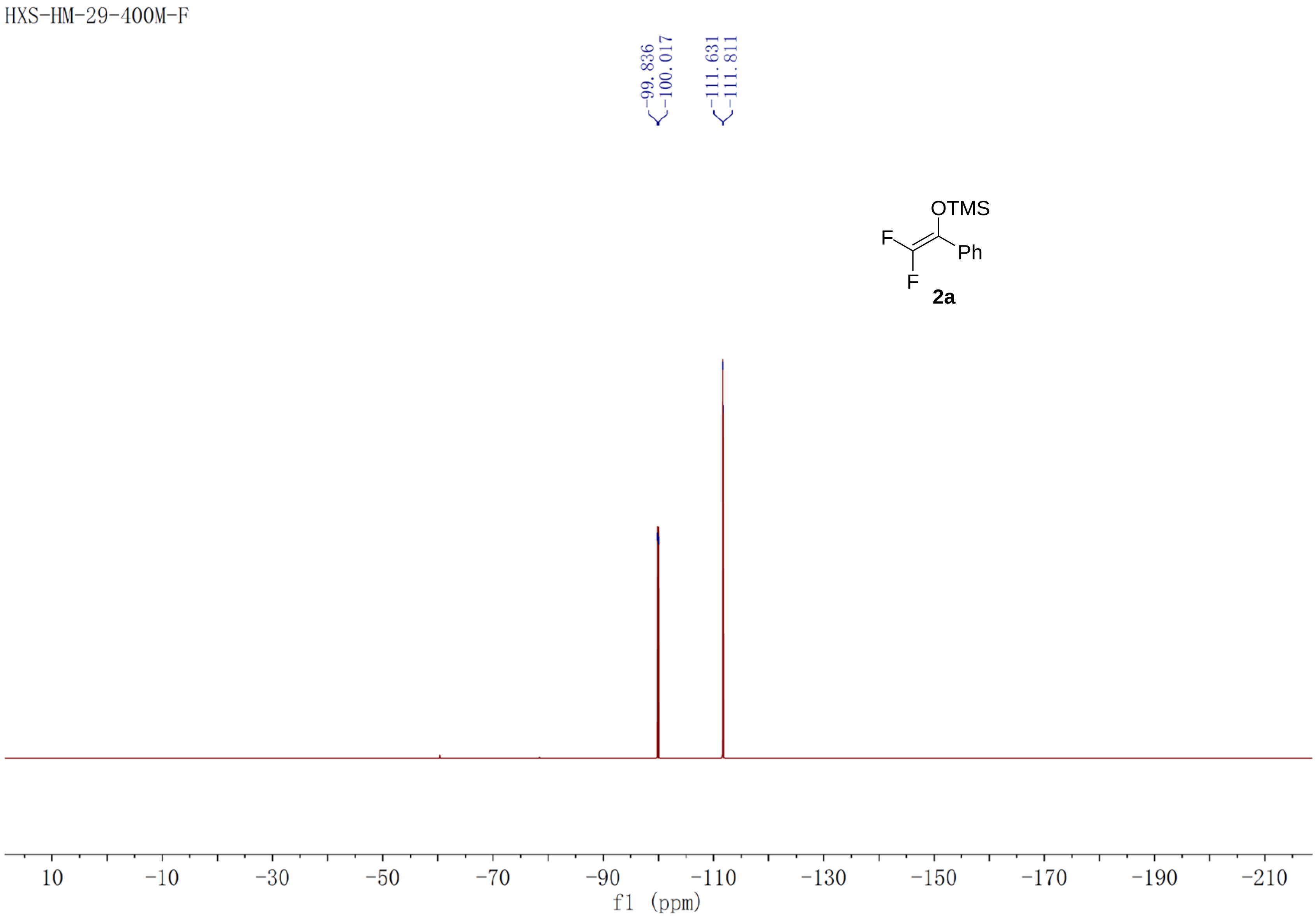

Supplementary Figure 19. ^{19}F NMR (376 MHz, $CDCl_3$) spectra for compound **2a**

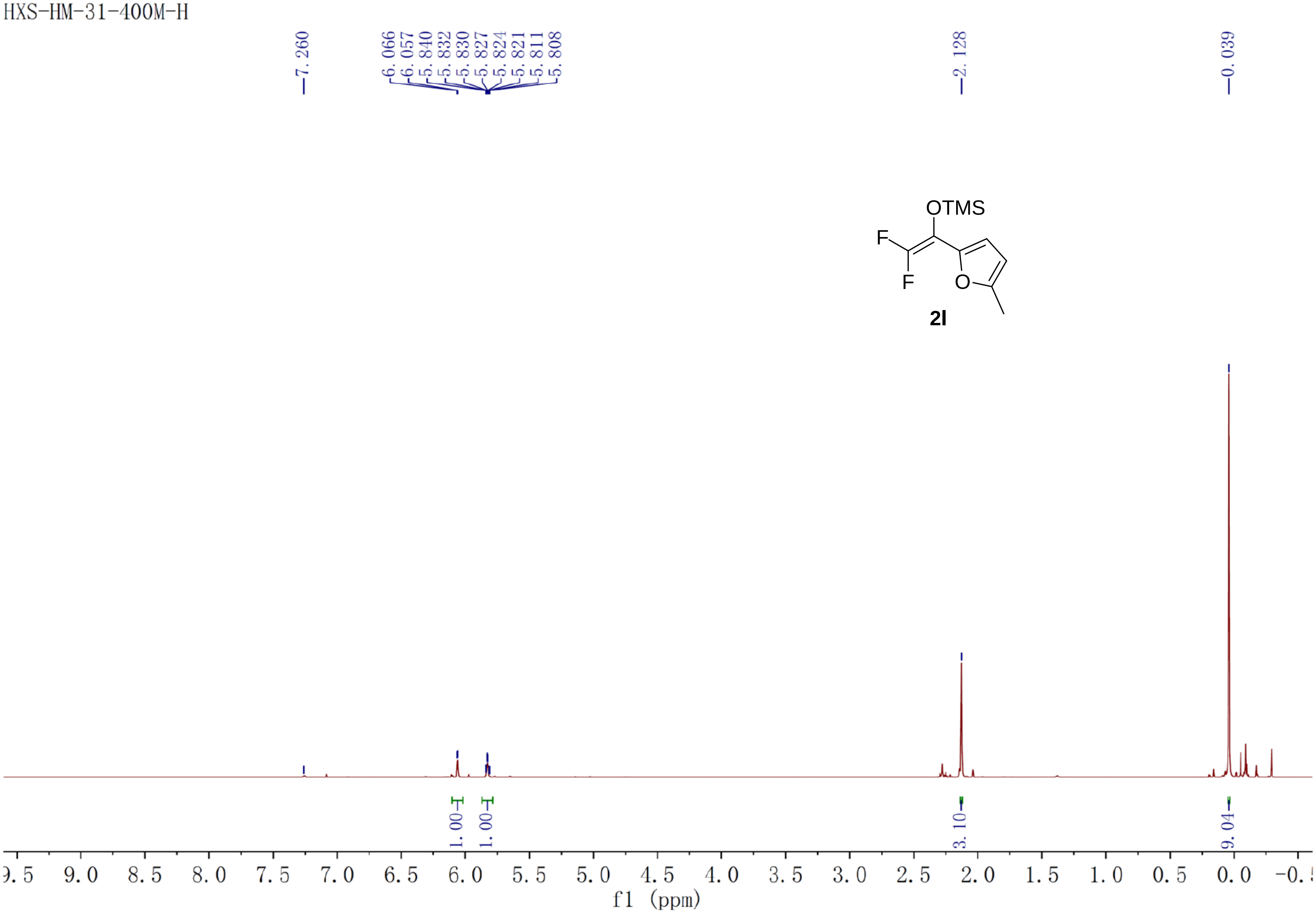

Supplementary Figure 20. ^{1}H NMR (400 MHz, $CDCl_3$) spectra for compound **2l**

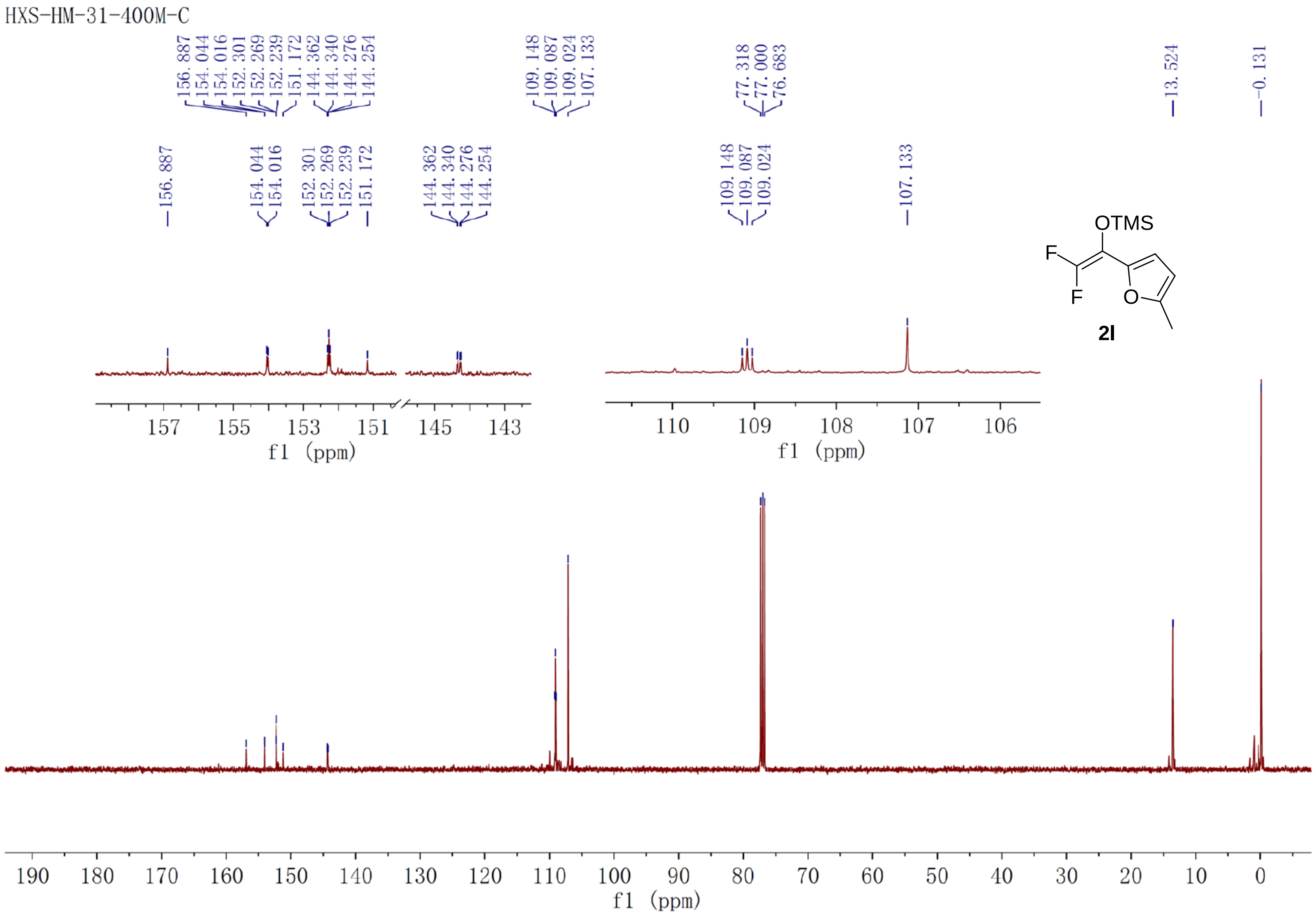

Supplementary Figure 21. ^{13}C NMR (100 MHz, $CDCl_3$) spectra for compound **2l**

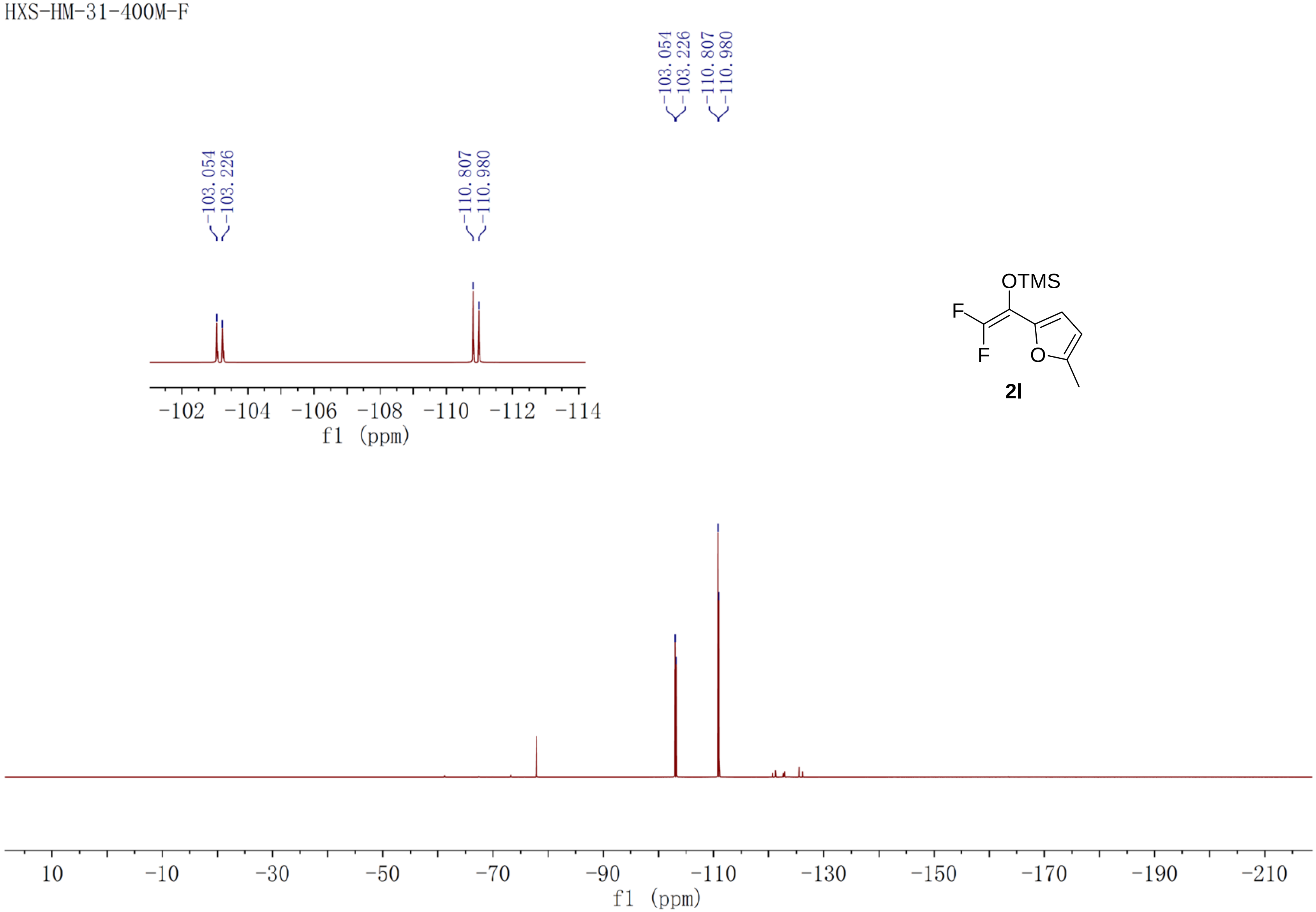

Supplementary Figure 22. ^{19}F NMR (376 MHz, $CDCl_3$) spectra for compound **2l**

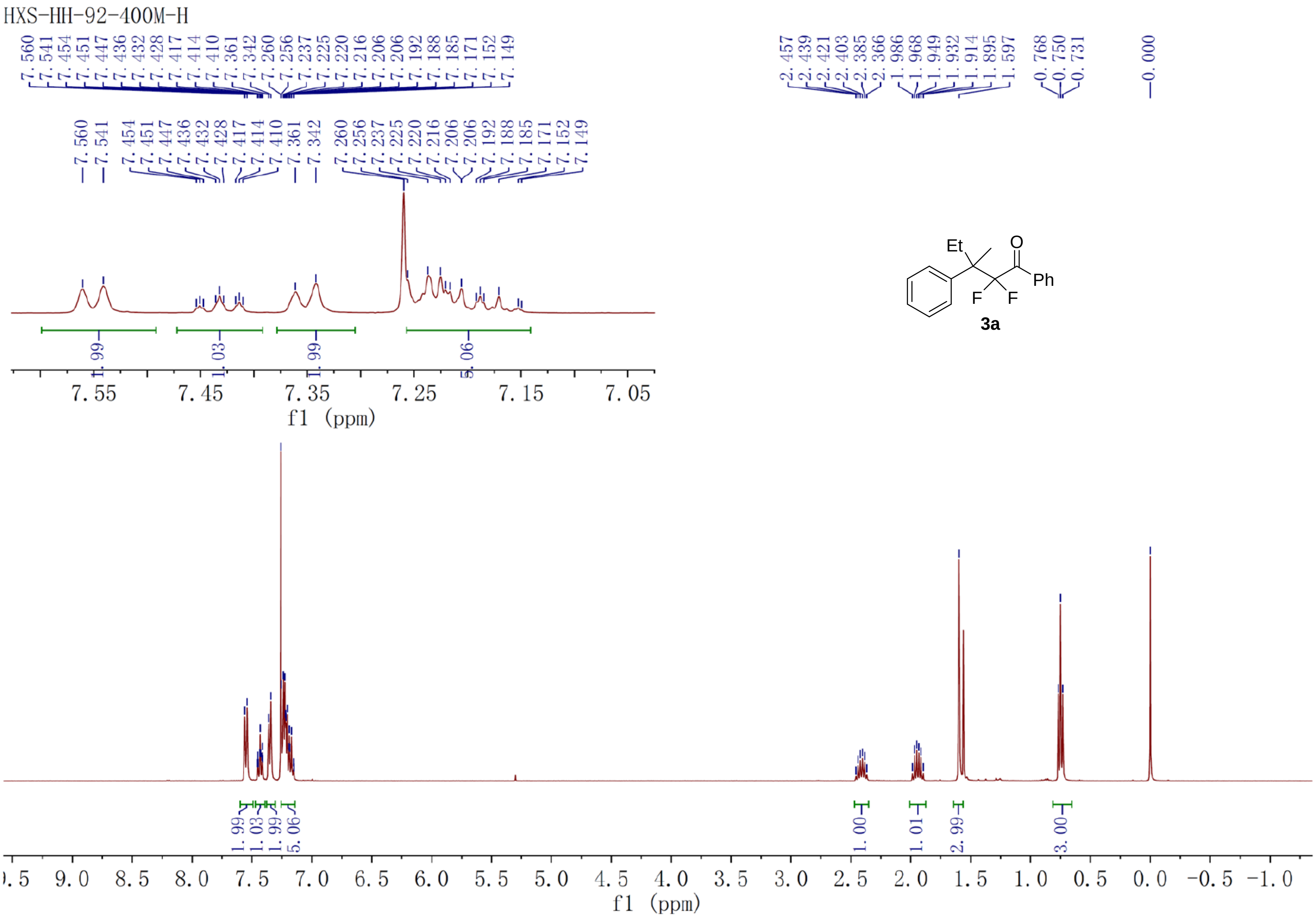

Supplementary Figure 23. ^{1}H NMR (400 MHz, $CDCl_3$) spectra for compound **3a**

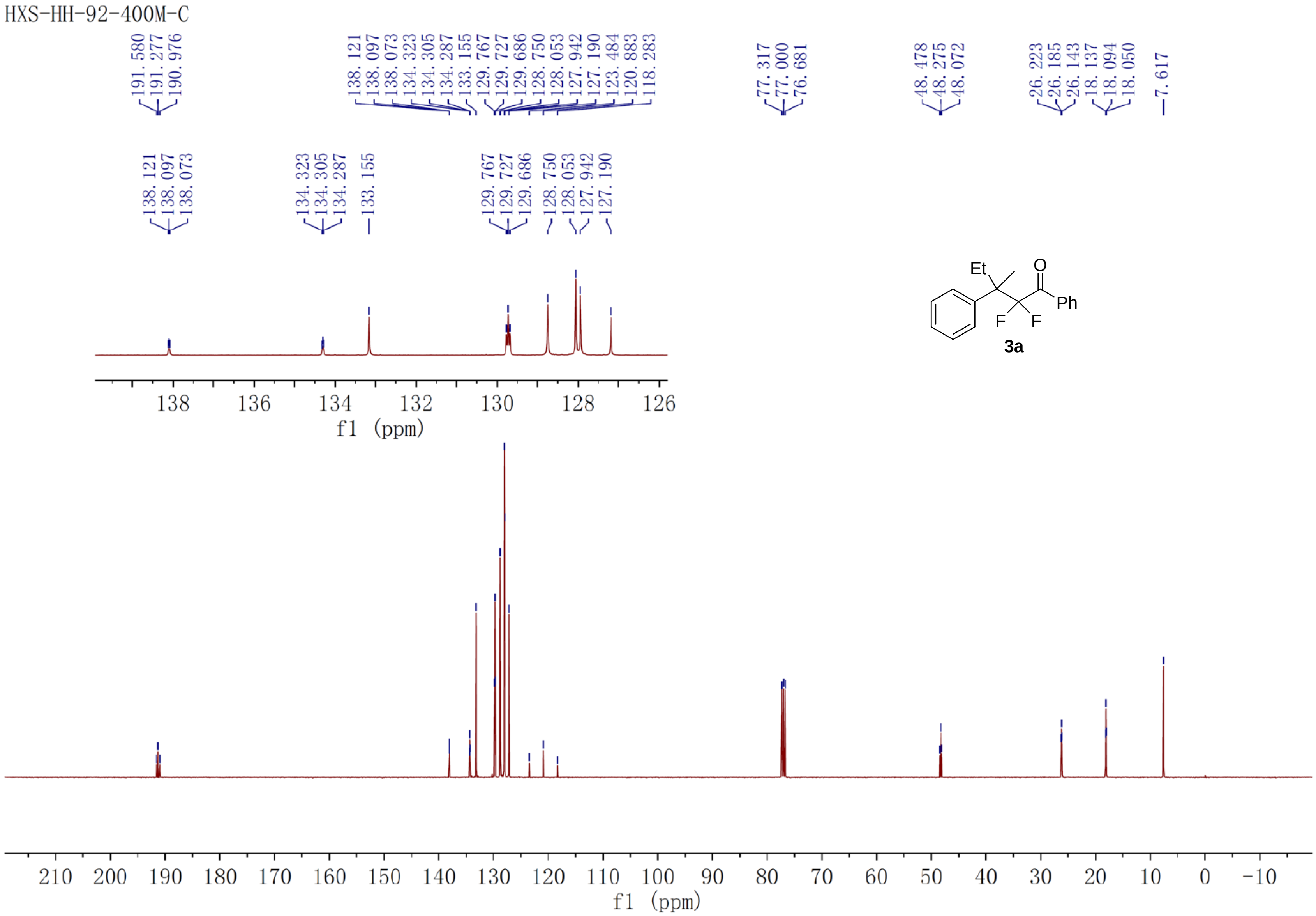

Supplementary Figure 24. ^{13}C NMR (100 MHz, $CDCl_3$) spectra for compound **3a**

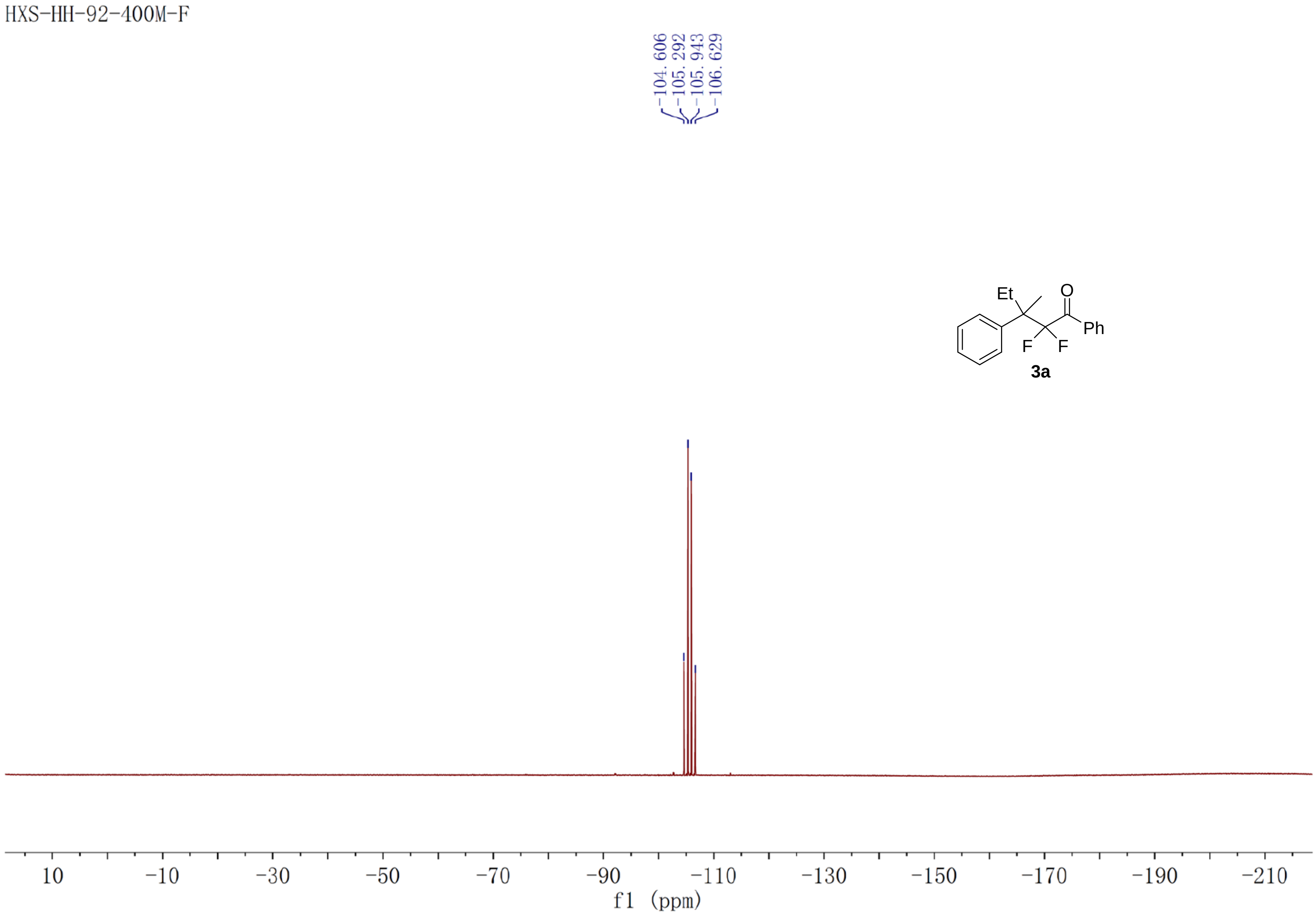

Supplementary Figure 25. ^{19}F NMR (376 MHz, $CDCl_3$) spectra for compound **3a**

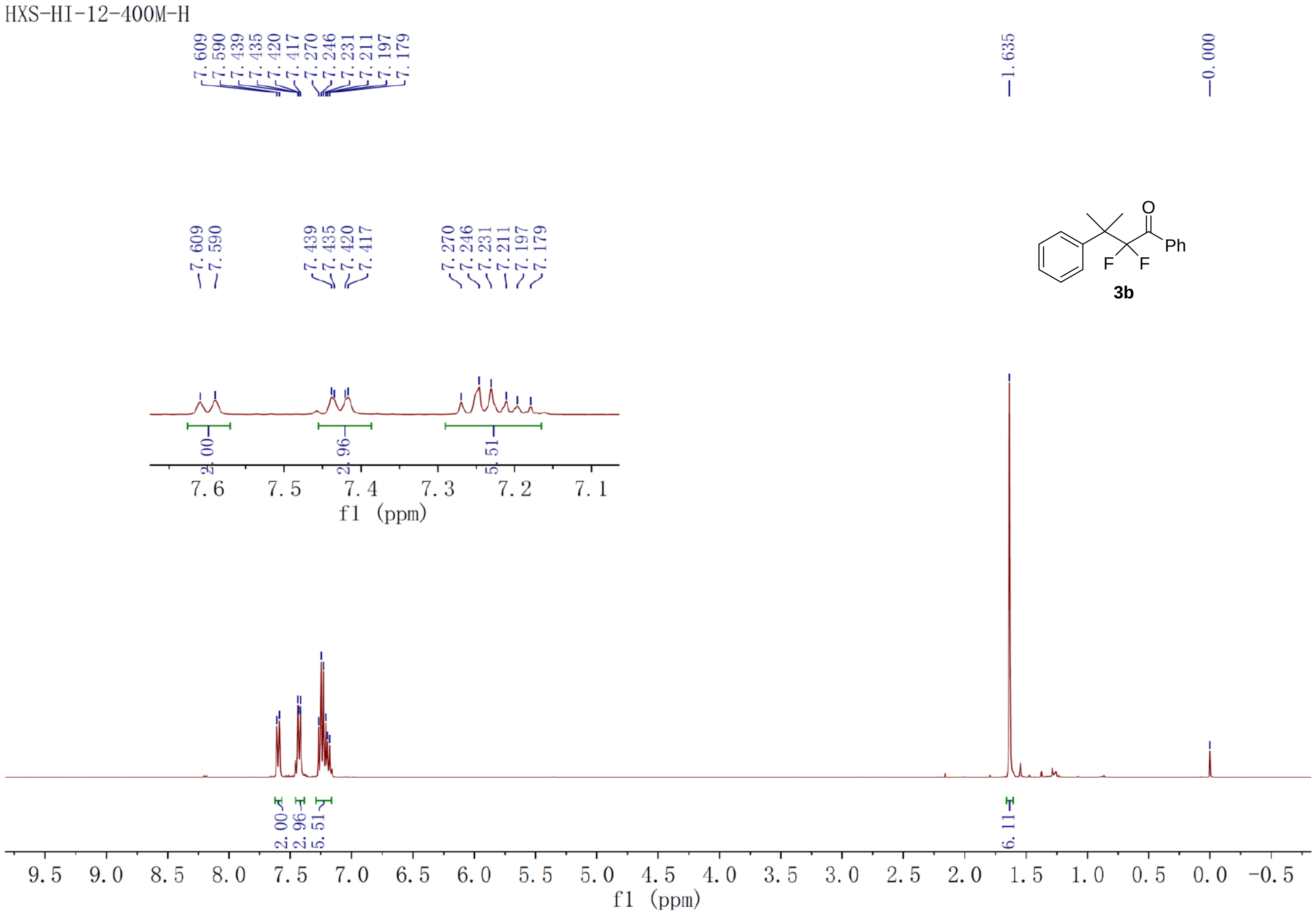

Supplementary Figure 26. ^{1}H NMR (400 MHz, $CDCl_3$) spectra for compound **3b**

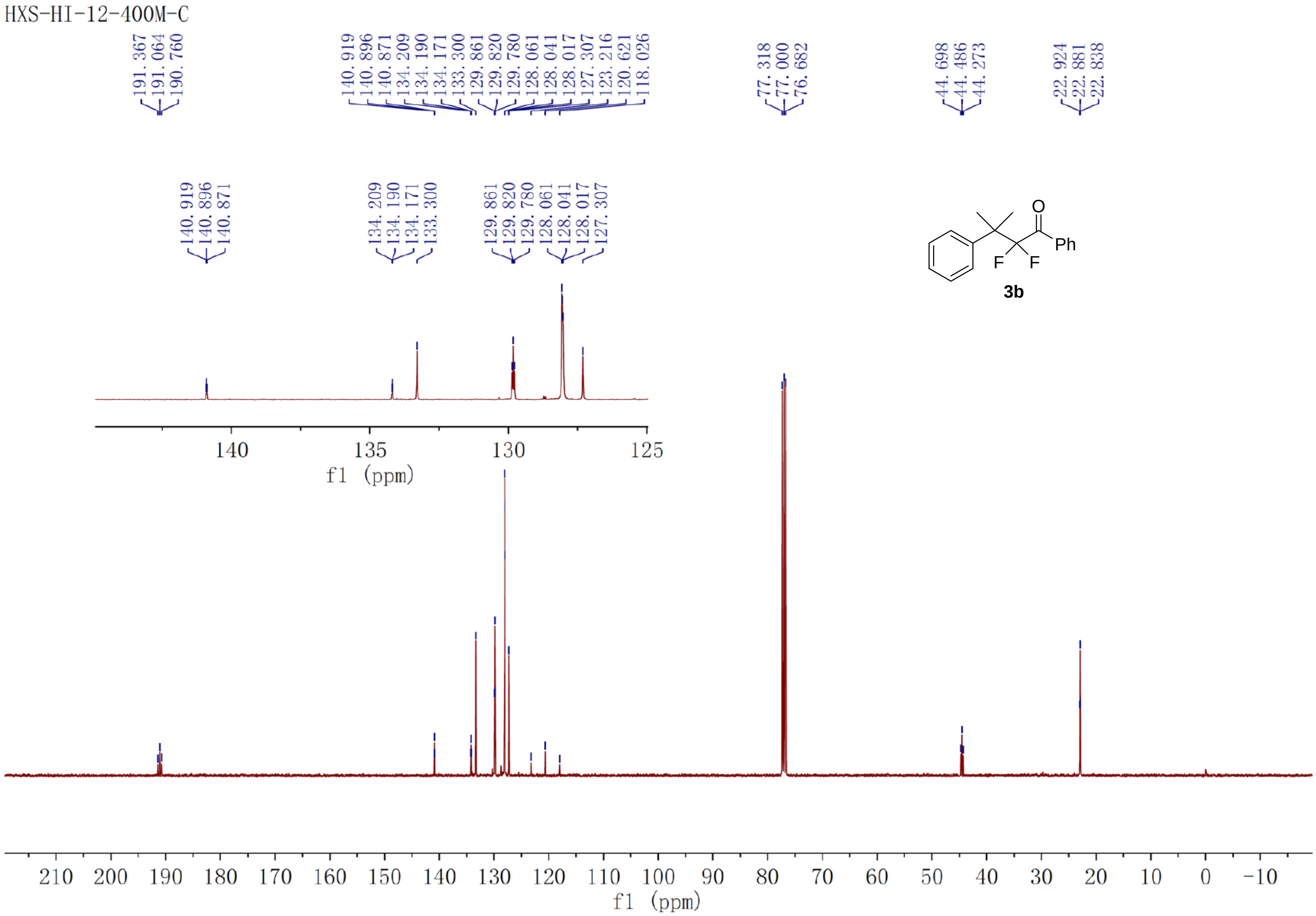

Supplementary Figure 27. ^{13}C NMR (100 MHz, $CDCl_3$) spectra for compound **3b**

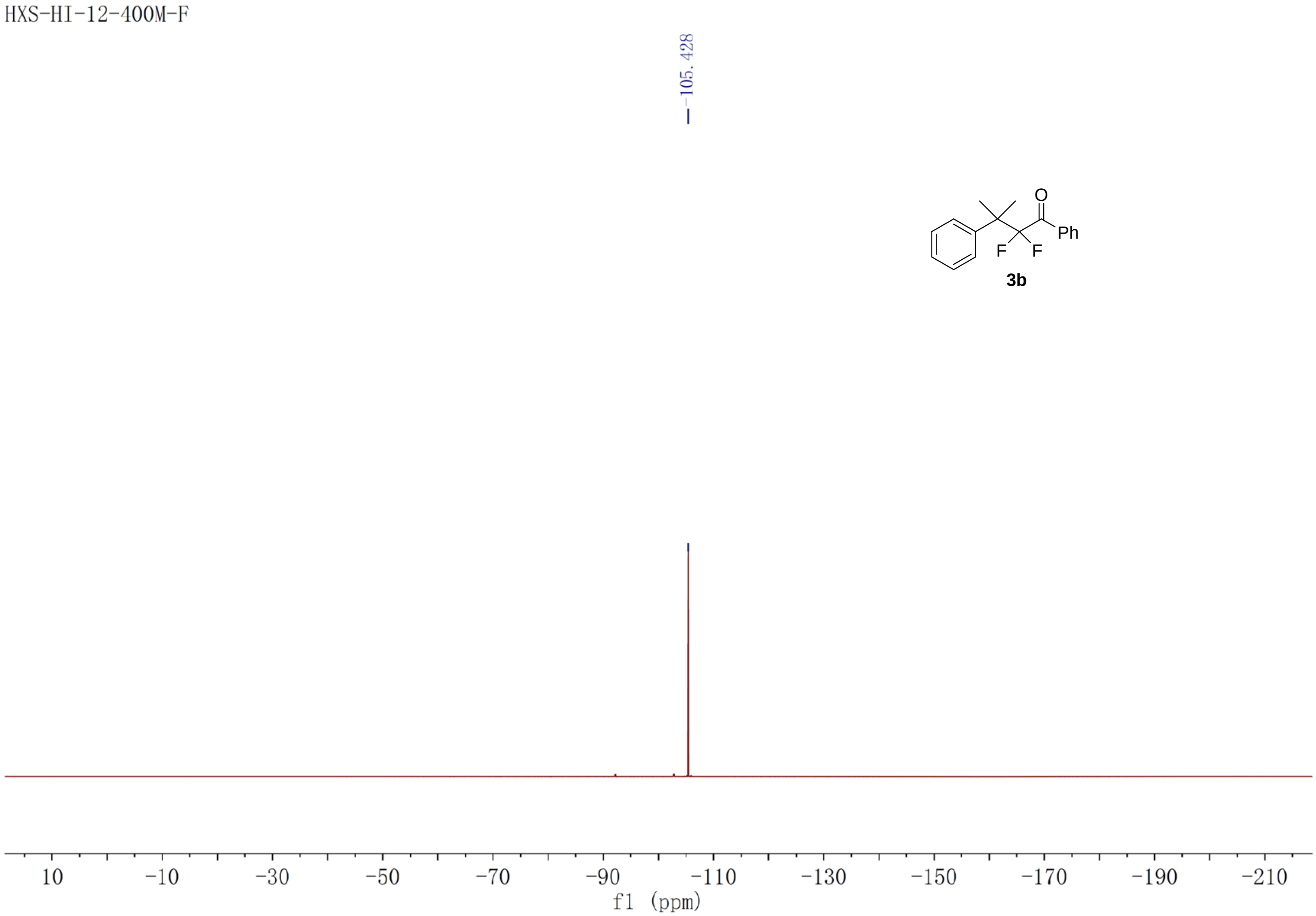

Supplementary Figure 28. ^{19}F NMR (376 MHz, $CDCl_3$) spectra for compound **3b**

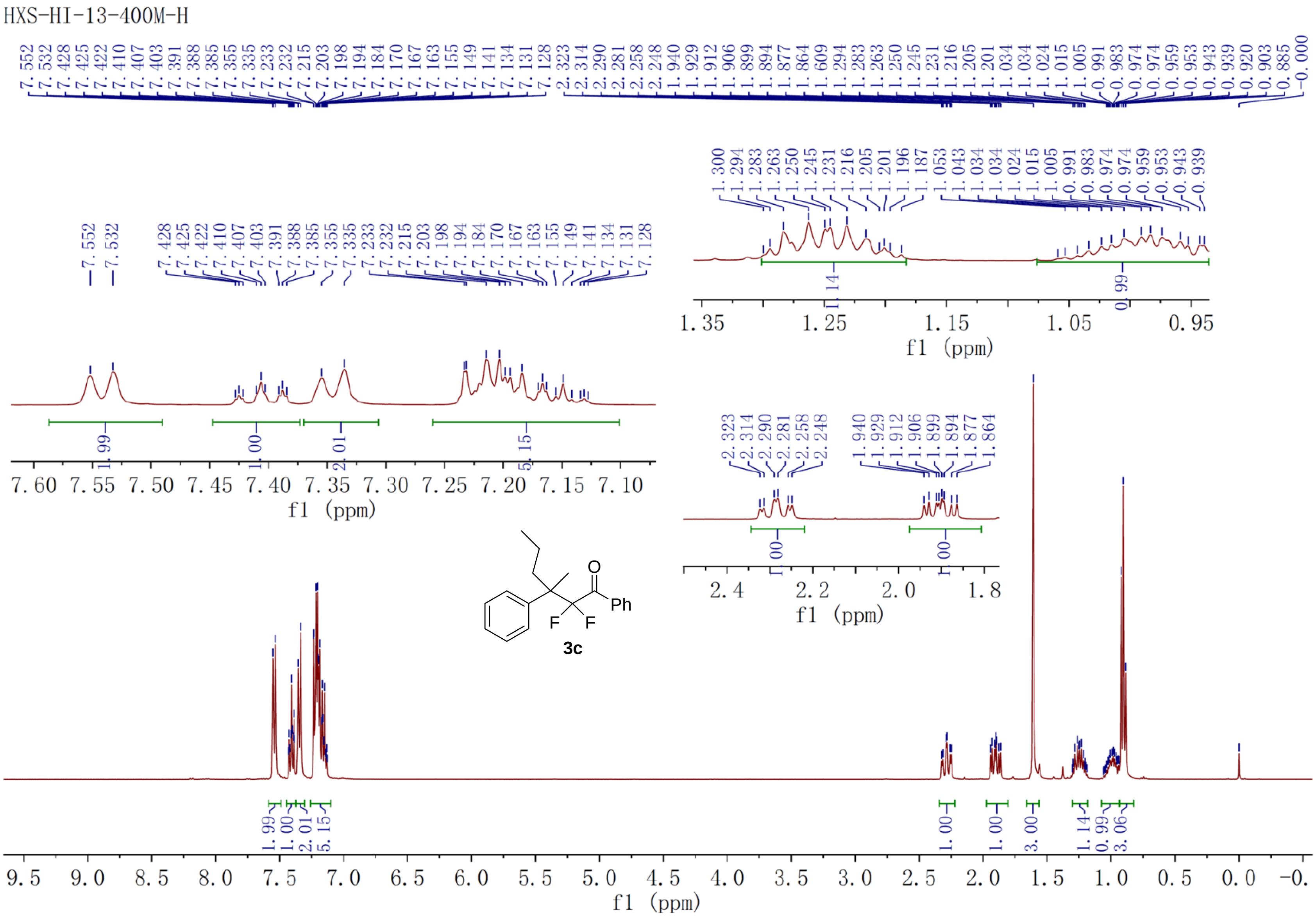

Supplementary Figure 29. ^{1}H NMR (400 MHz, $CDCl_3$) spectra for compound **3c**

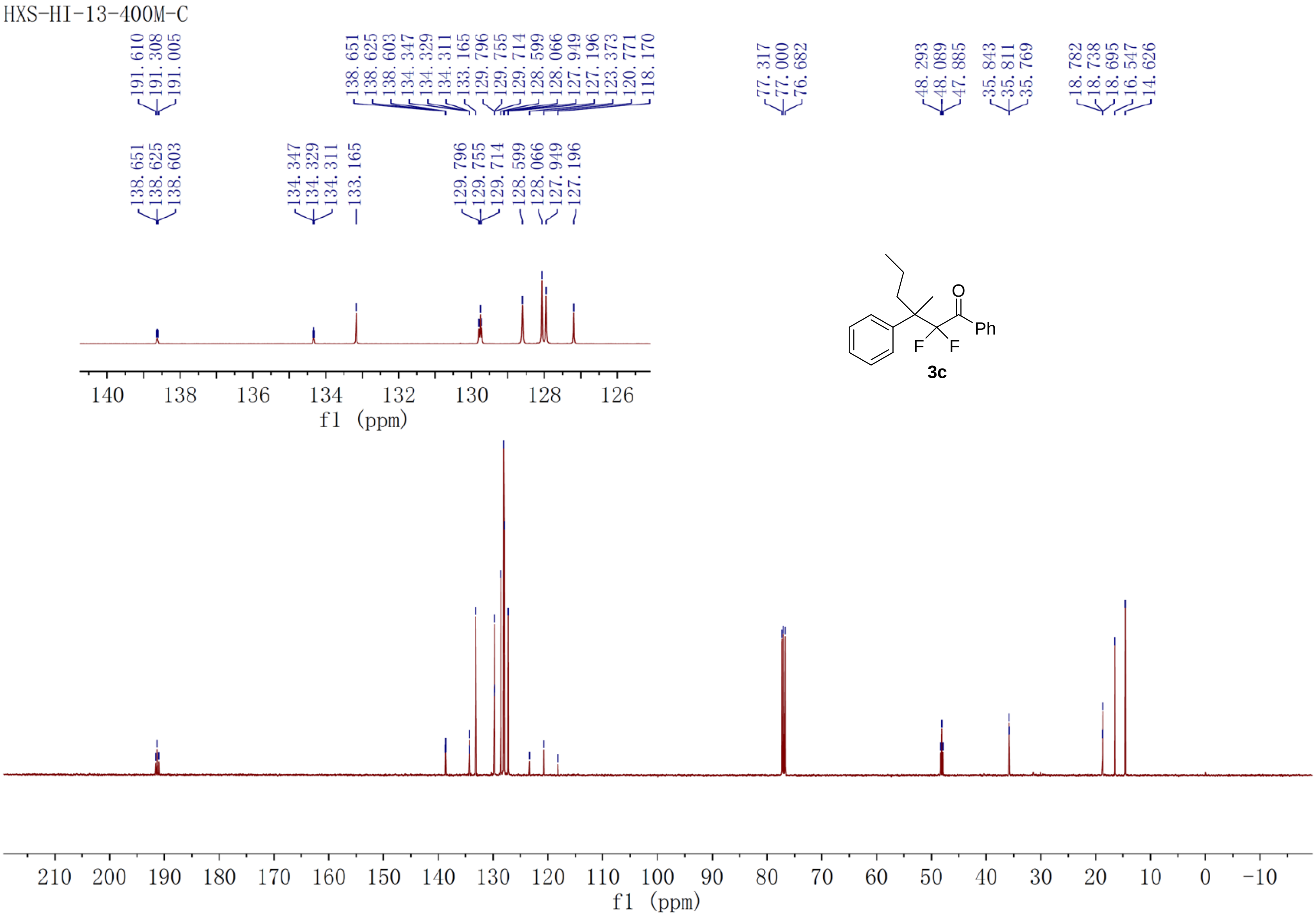

Supplementary Figure 30. ^{13}C NMR (100 MHz, $CDCl_3$) spectra for compound **3c**

HXS-HI-13-400M-F

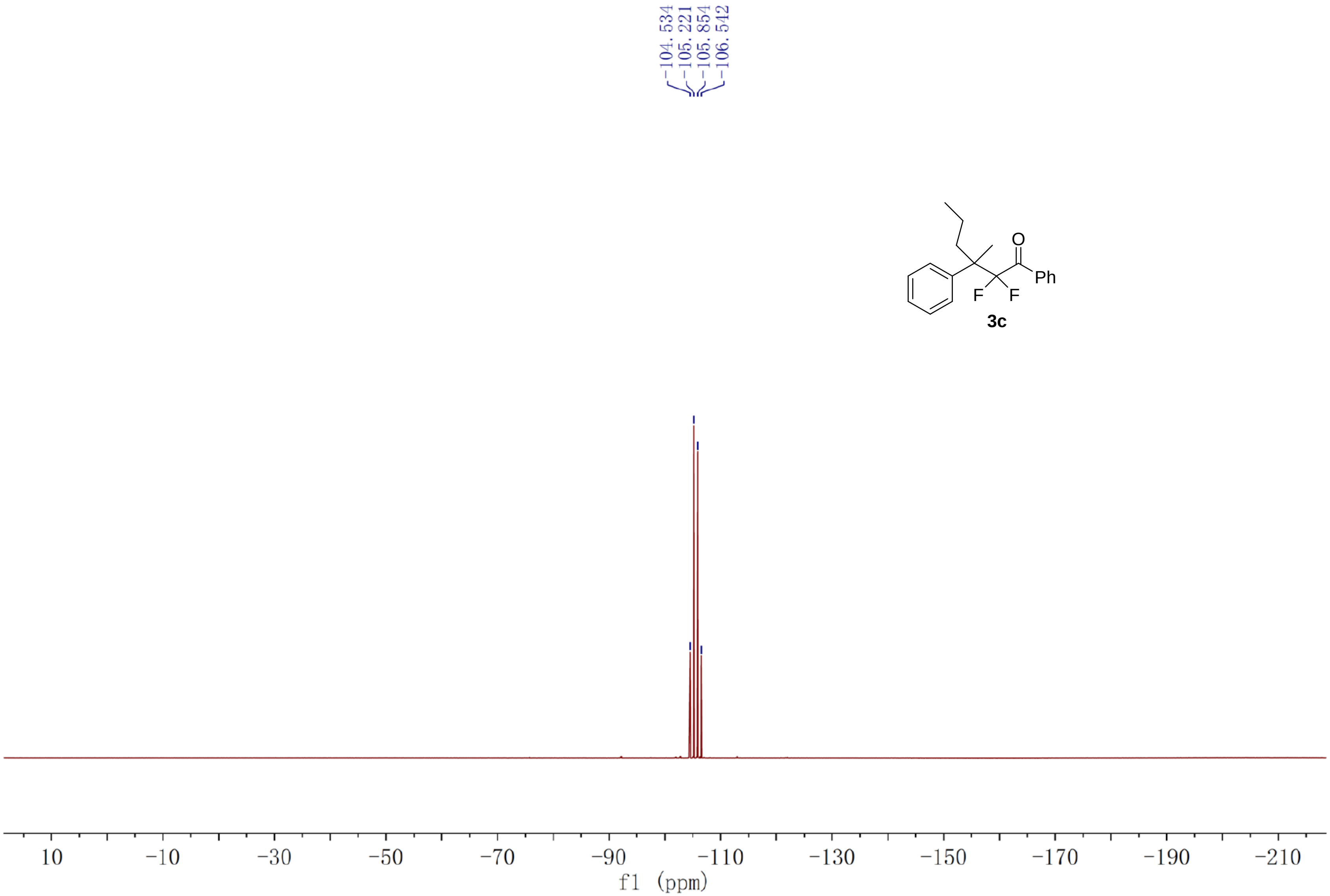

3c

Supplementary Figure 31. ^{19}F NMR (376 MHz, $CDCl_3$) spectra for compound **3c**

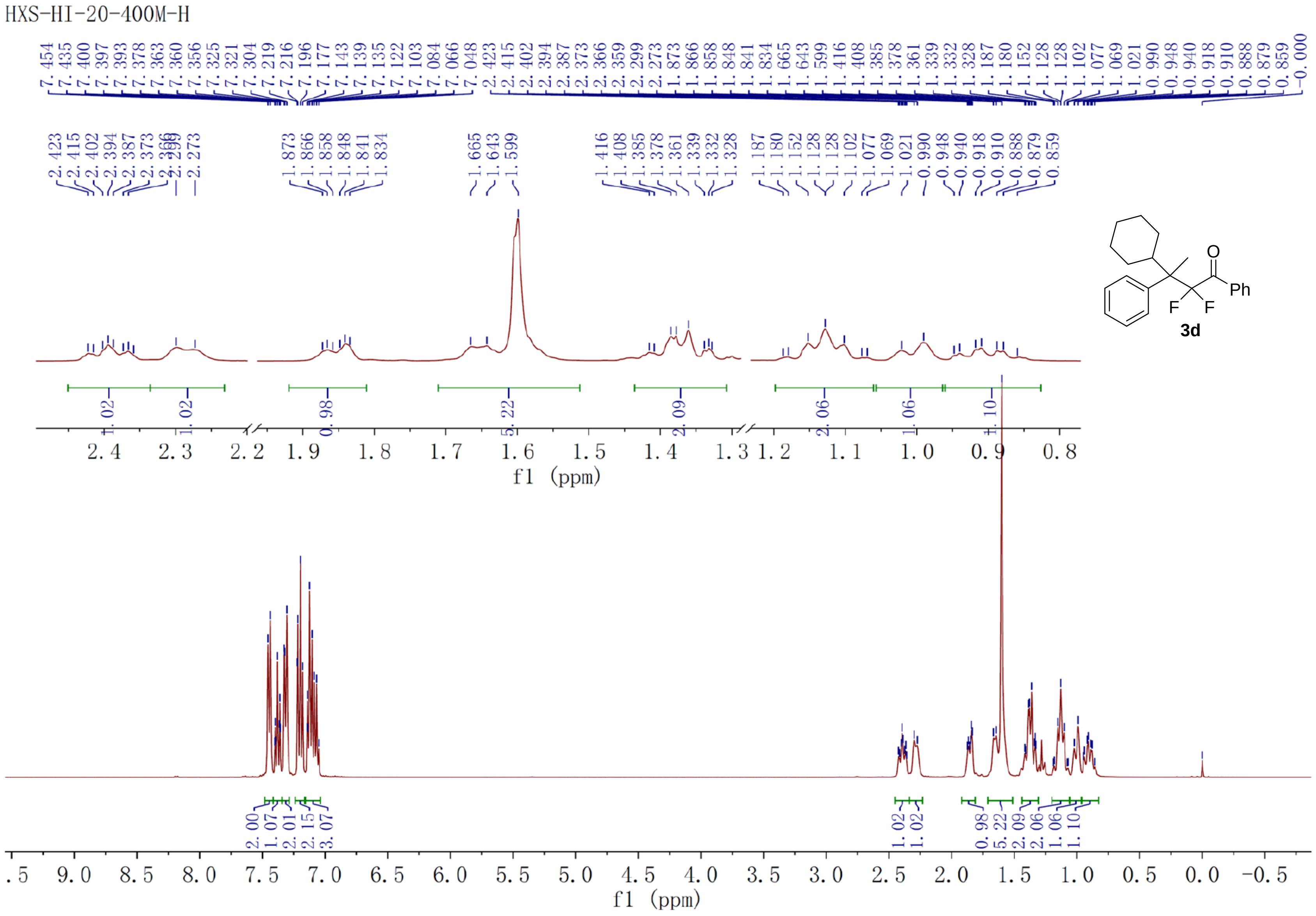

Supplementary Figure 32. ^{1}H NMR (400 MHz, $CDCl_3$) spectra for compound **3d**

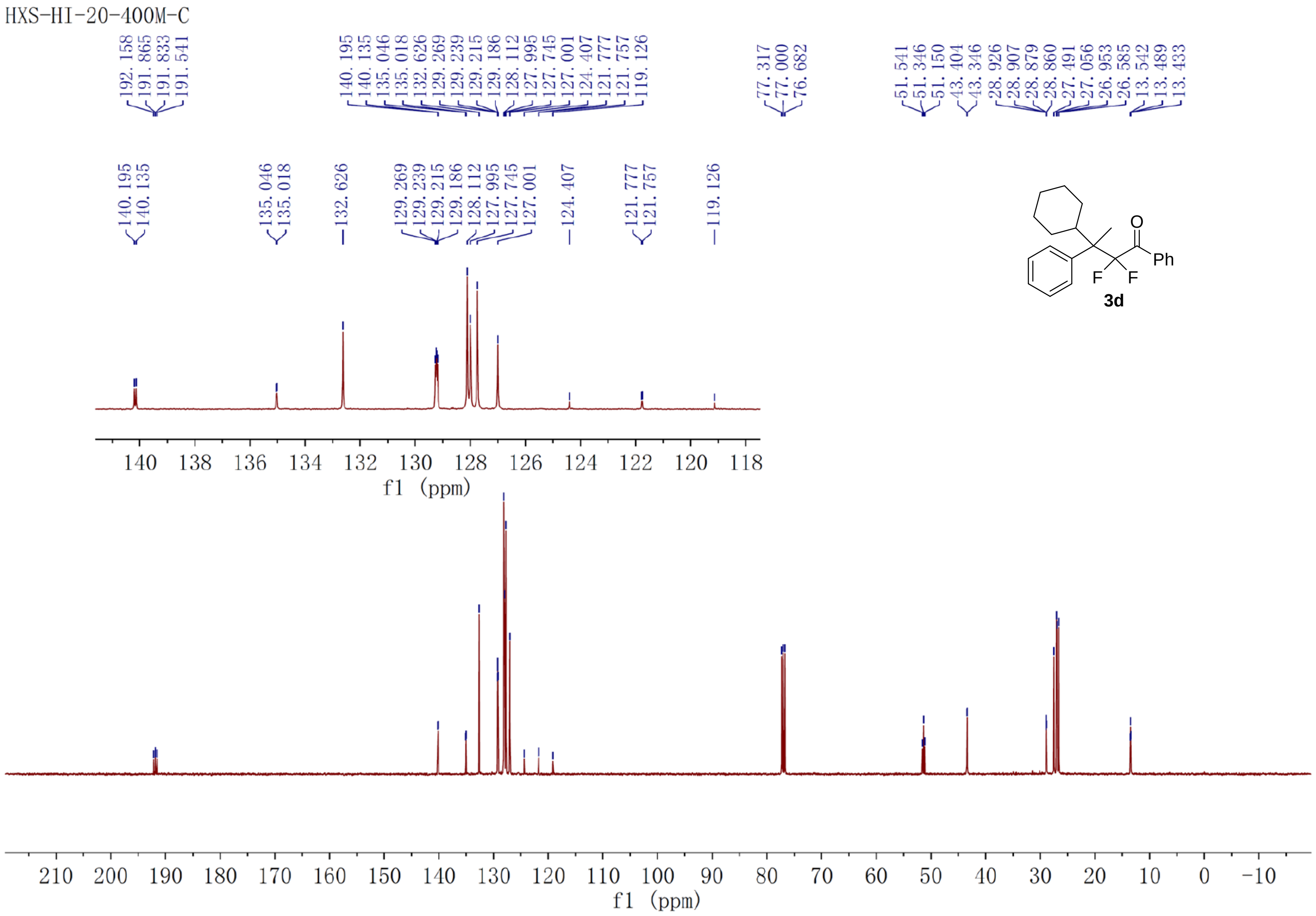

Supplementary Figure 33. ^{13}C NMR (100 MHz, $CDCl_3$) spectra for compound **3d**

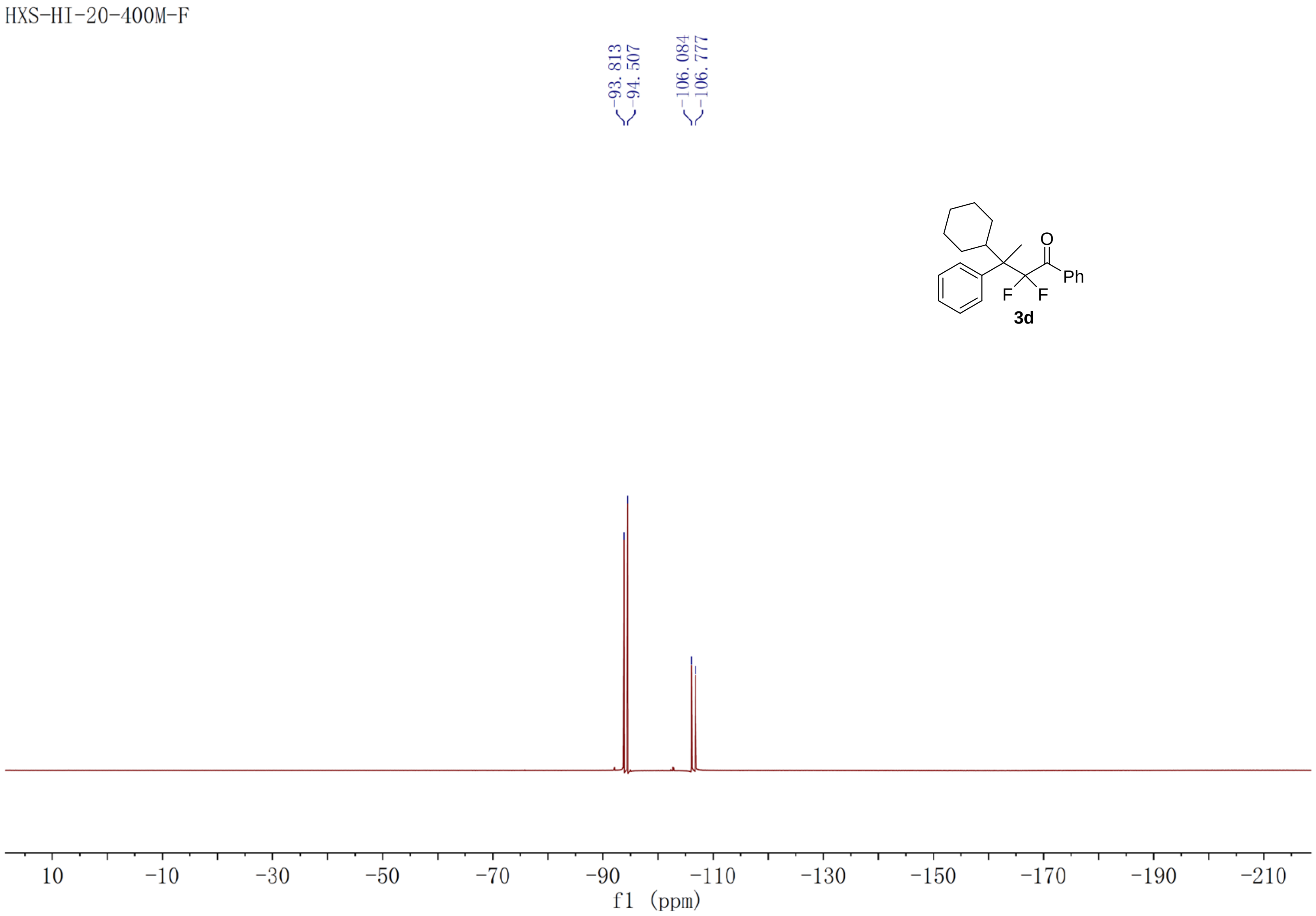

3d

Supplementary Figure 34. ^{19}F NMR (376 MHz, $CDCl_3$) spectra for compound **3d**

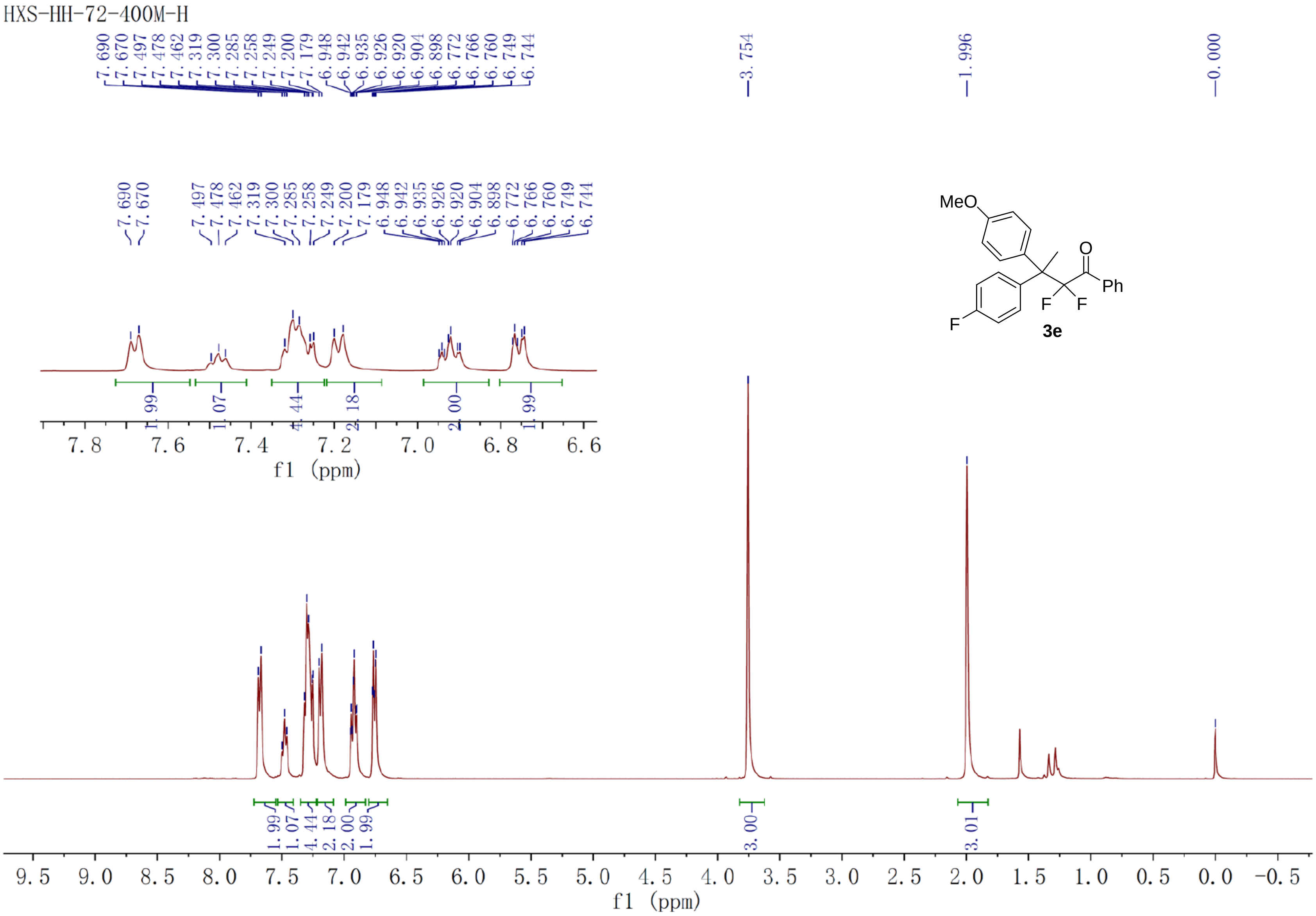

Supplementary Figure 35. ^{1}H NMR (400 MHz, $CDCl_3$) spectra for compound **3e**

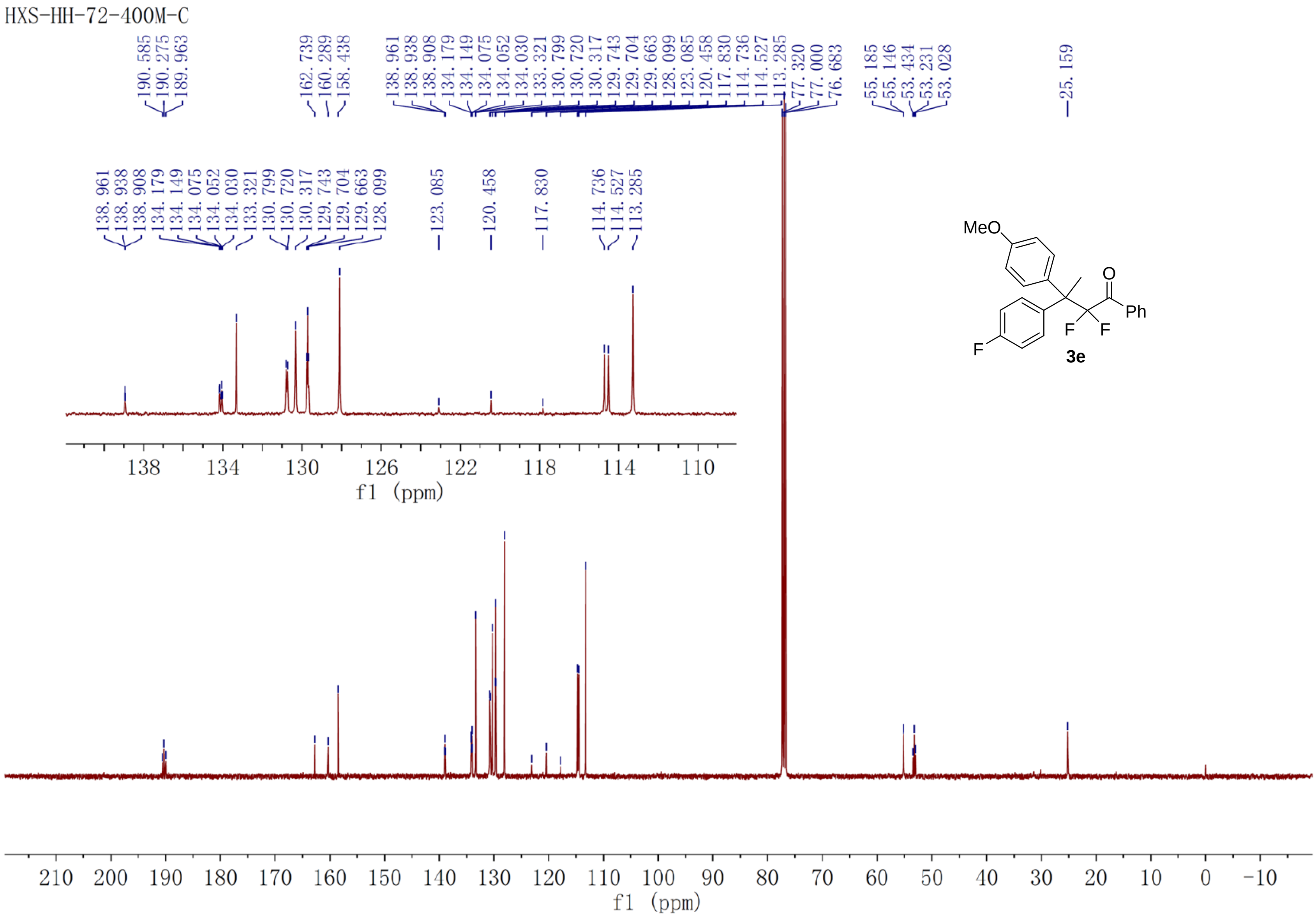

Supplementary Figure 36. ^{13}C NMR (100 MHz, $CDCl_3$) spectra for compound **3e**

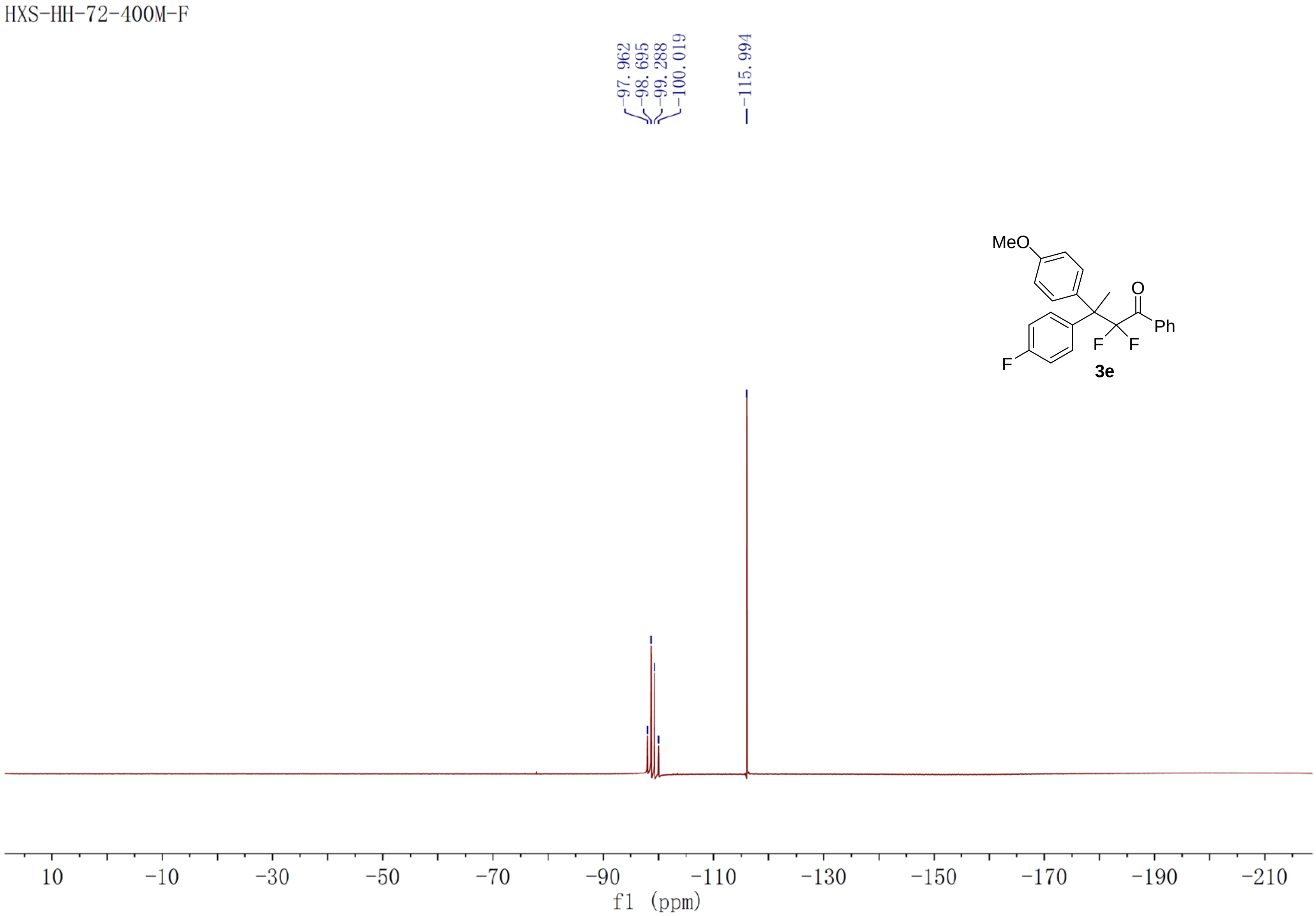

Supplementary Figure 37. ^{19}F NMR (376 MHz, $CDCl_3$) spectra for compound **3e**

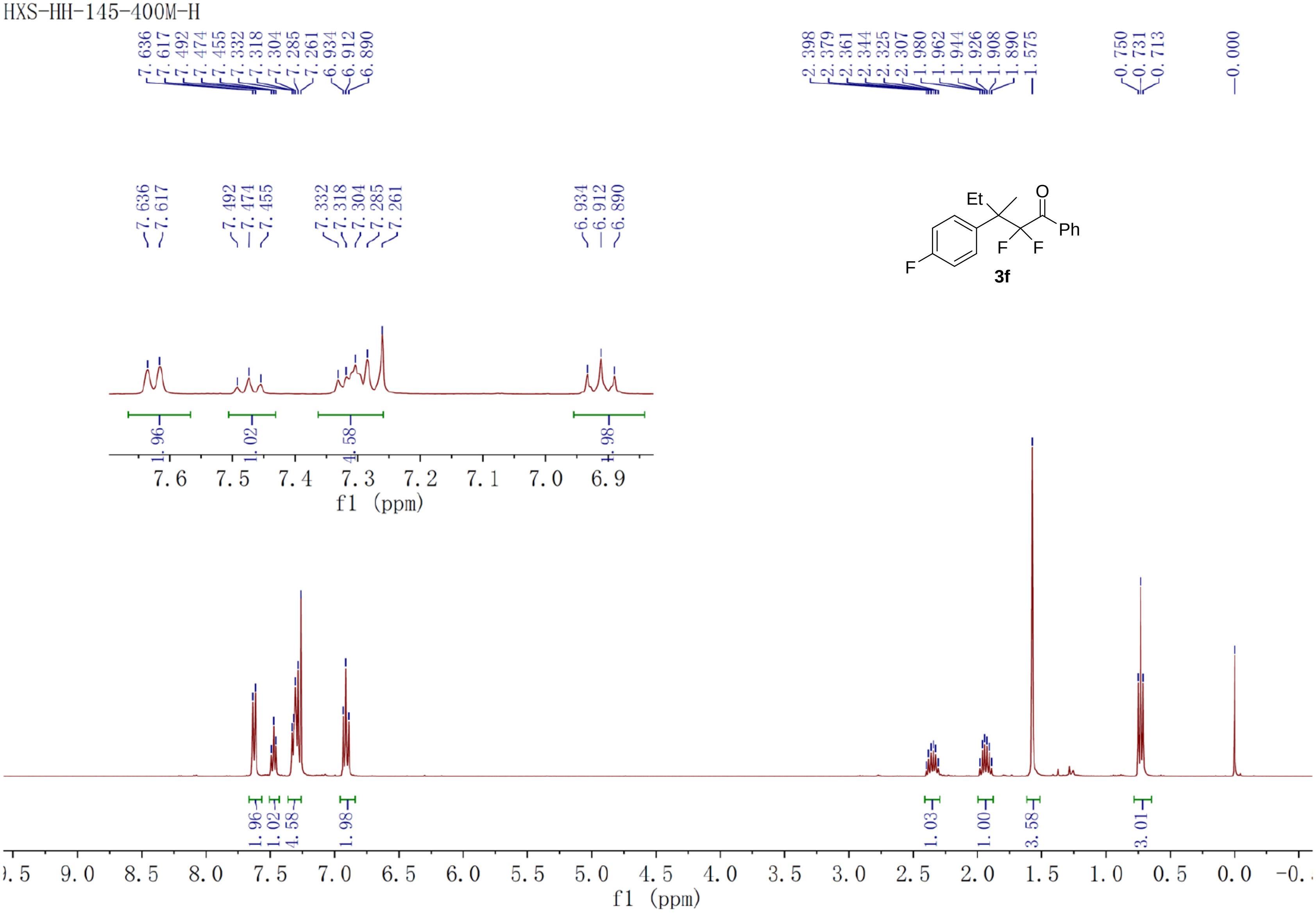

Supplementary Figure 38. ^{1}H NMR (400 MHz, $CDCl_3$) spectra for compound **3f**

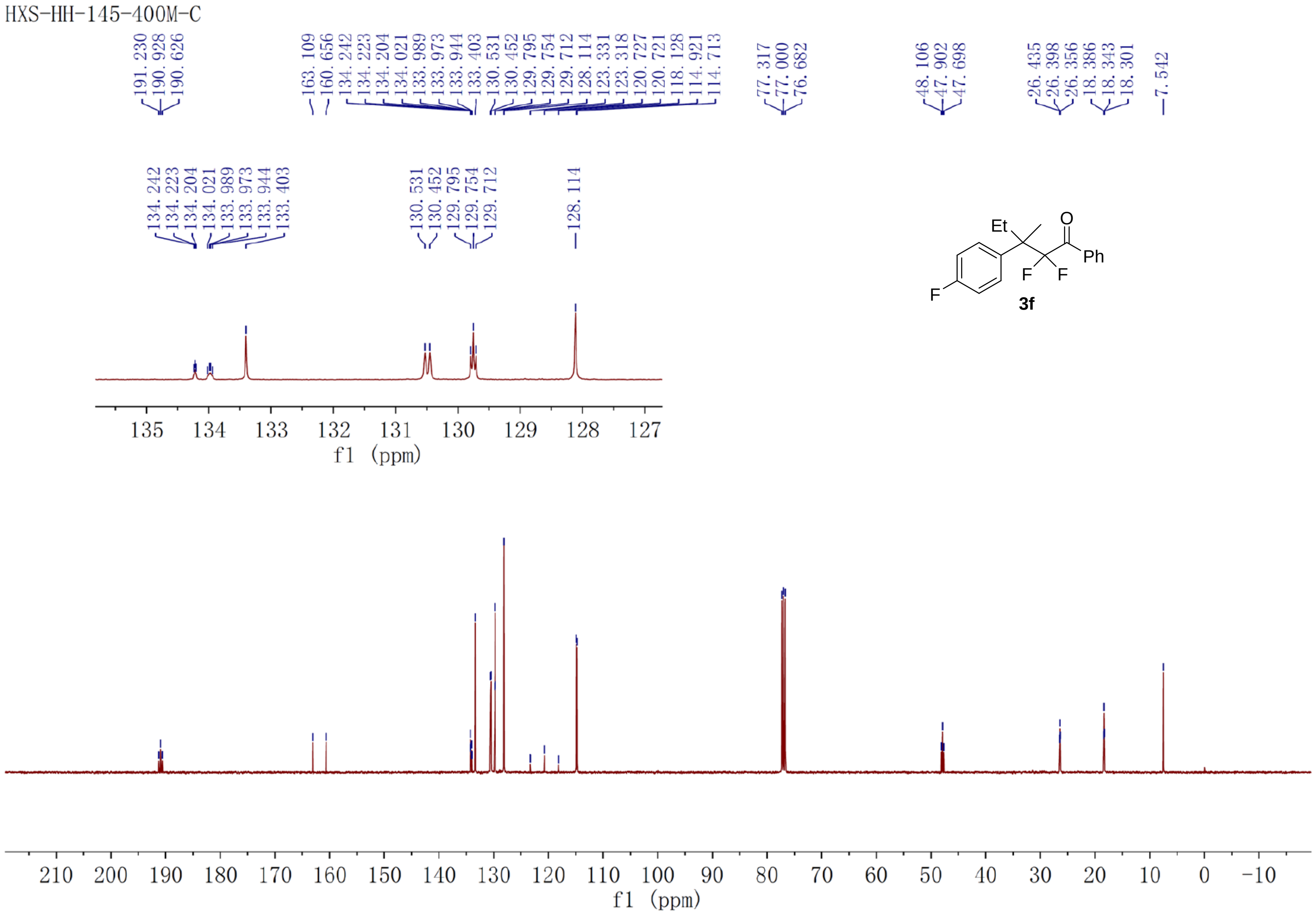

Supplementary Figure 39. ^{13}C NMR (100 MHz, $CDCl_3$) spectra for compound **3f**

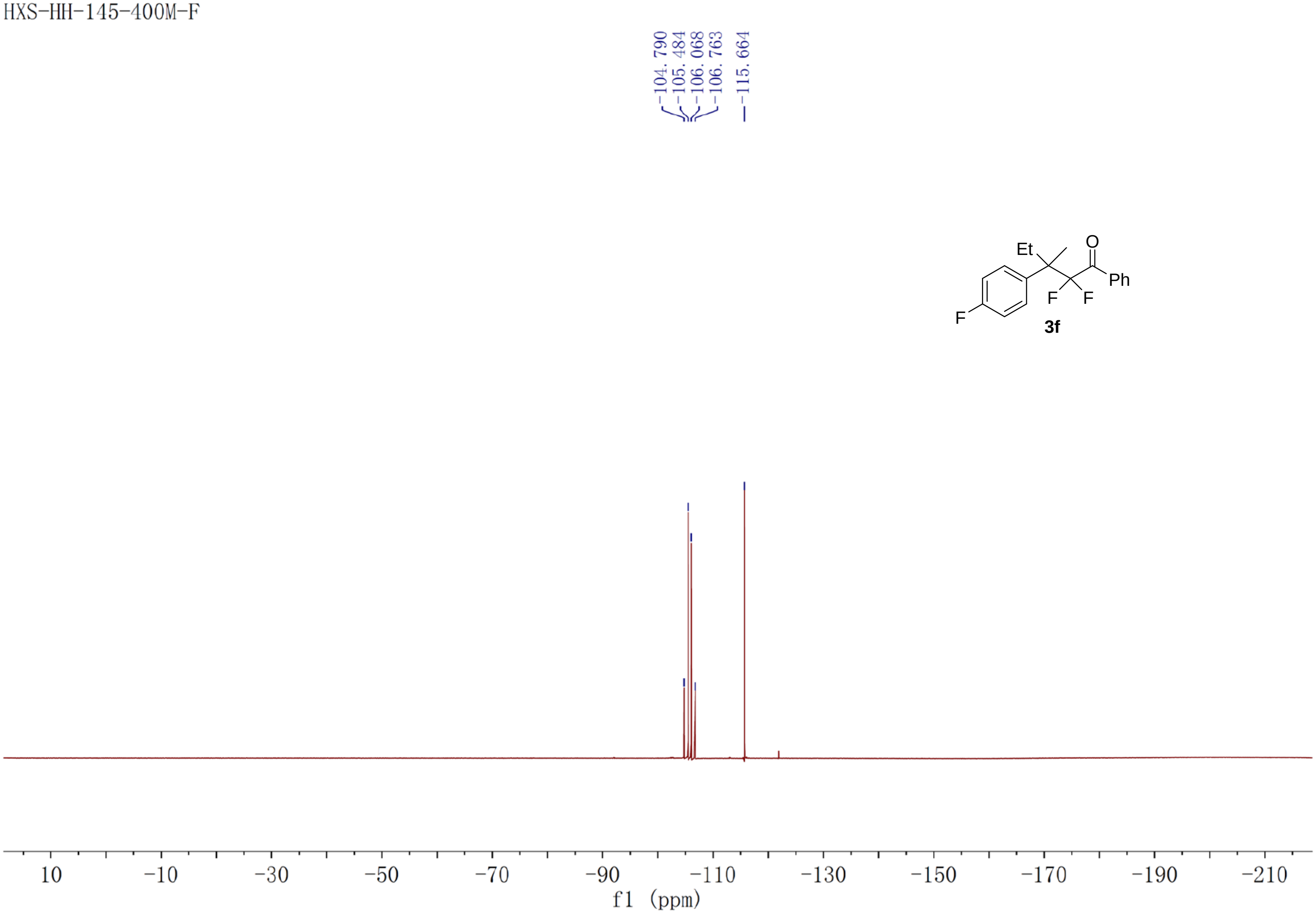

Supplementary Figure 40. ^{19}F NMR (376 MHz, $CDCl_3$) spectra for compound **3f**

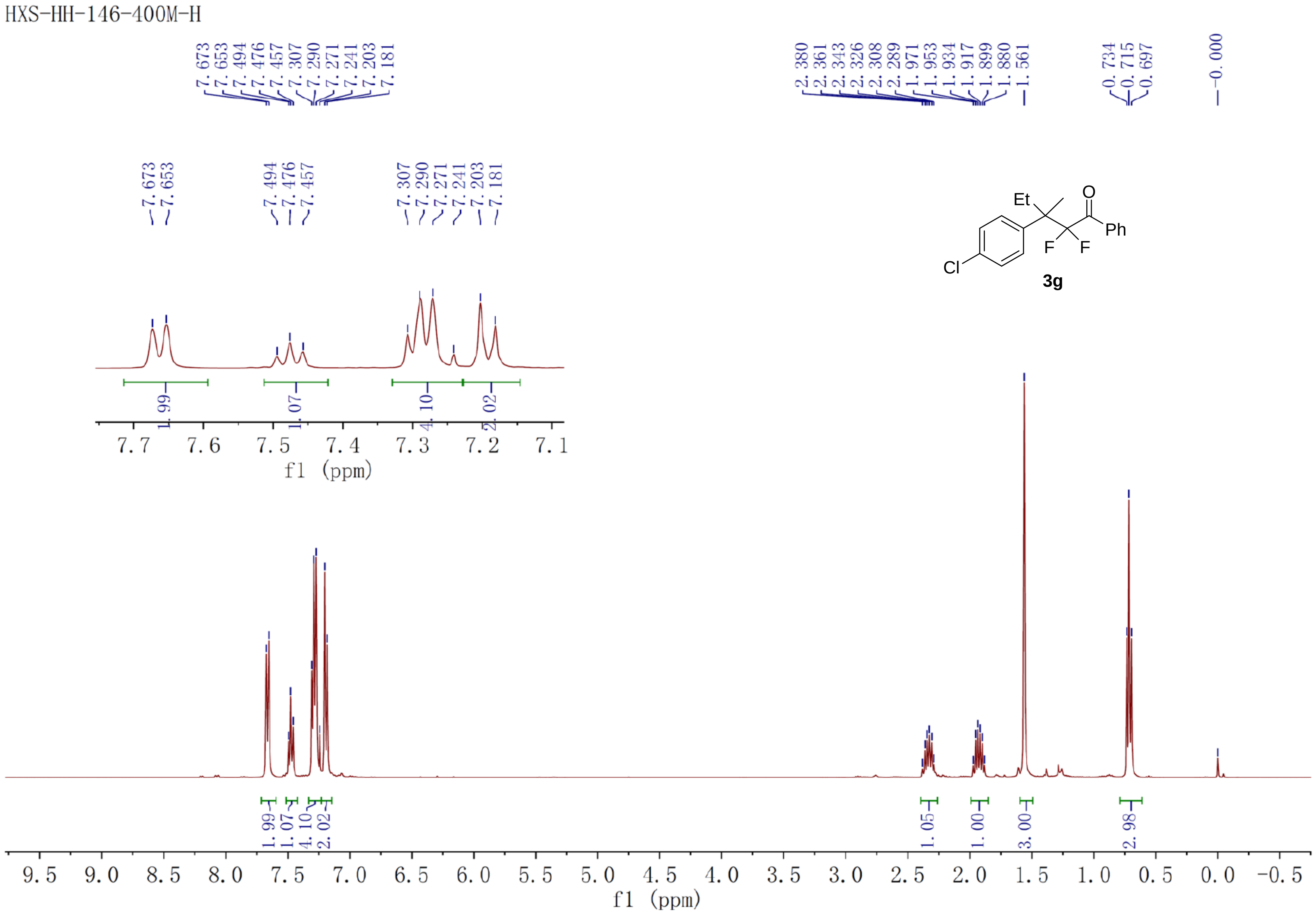

Supplementary Figure 41. ^{1}H NMR (400 MHz, $CDCl_3$) spectra for compound **3g**

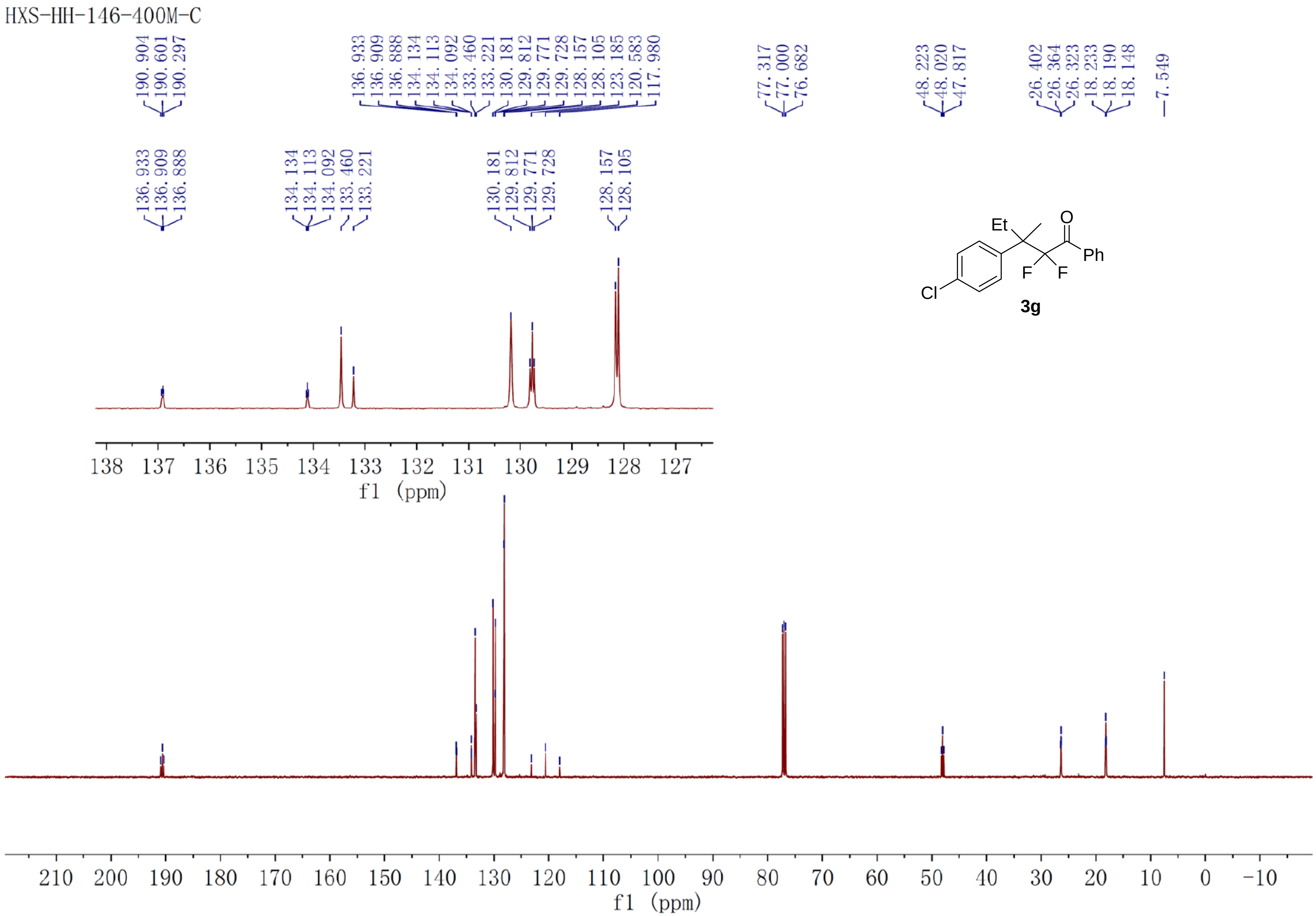

Supplementary Figure 42. ^{13}C NMR (100 MHz, $CDCl_3$) spectra for compound **3g**

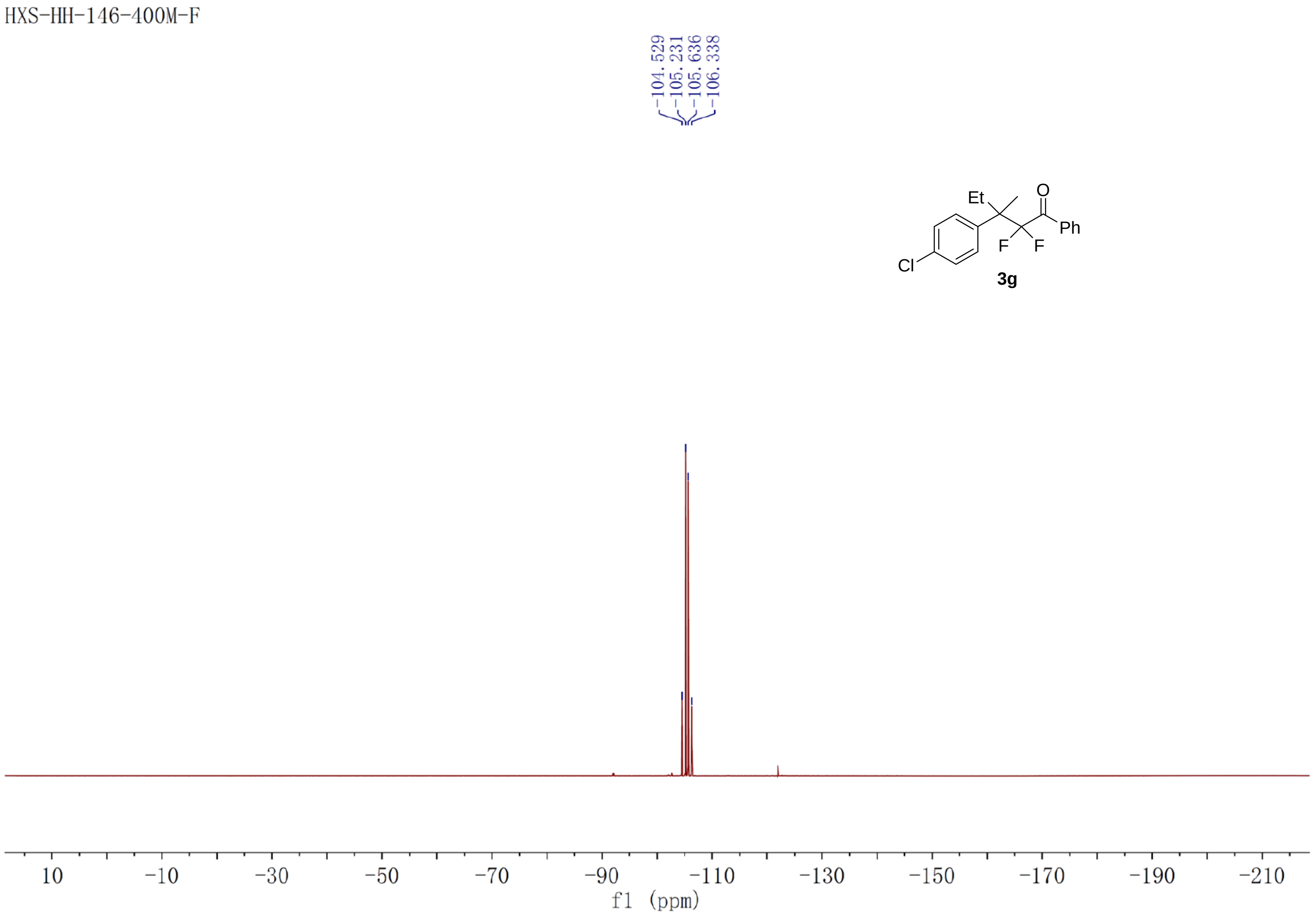

Supplementary Figure 43. ^{19}F NMR (376 MHz, $CDCl_3$) spectra for compound **3g**

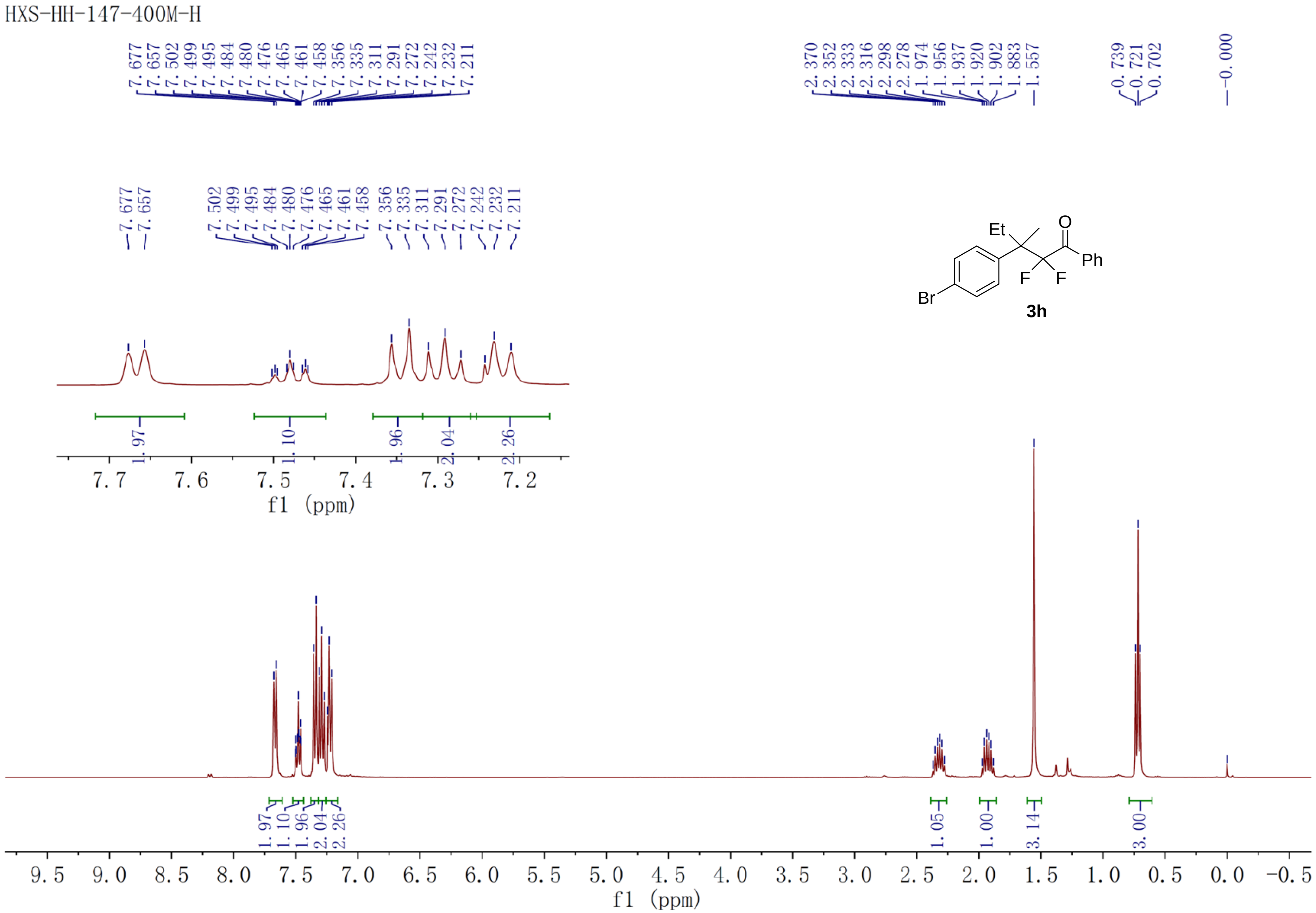

Supplementary Figure 44. ^{1}H NMR (400 MHz, $CDCl_3$) spectra for compound **3h**

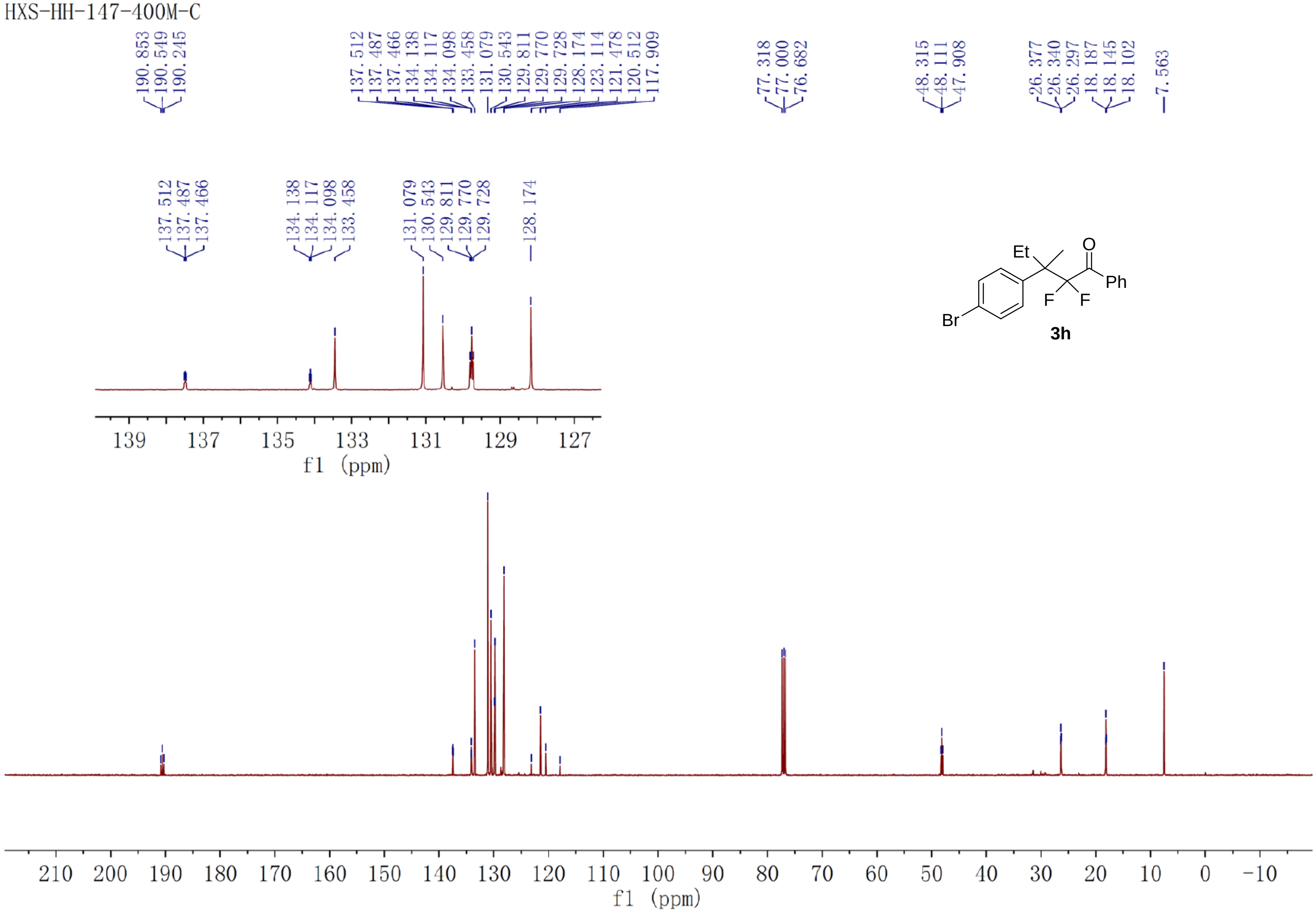

Supplementary Figure 45. ^{13}C NMR (100 MHz, $CDCl_3$) spectra for compound **3h**

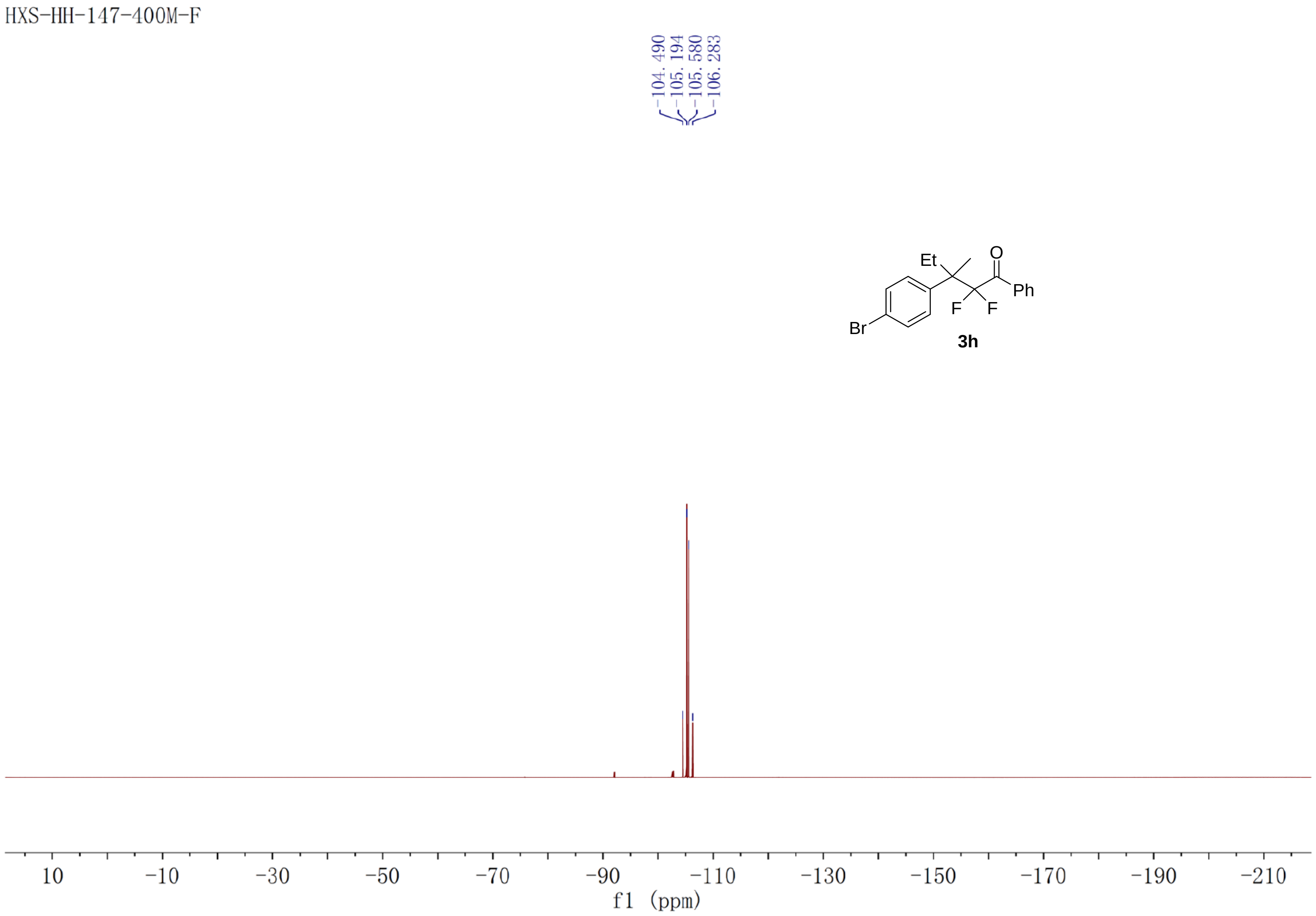

Supplementary Figure 46. ^{19}F NMR (376 MHz, $CDCl_3$) spectra for compound **3h**

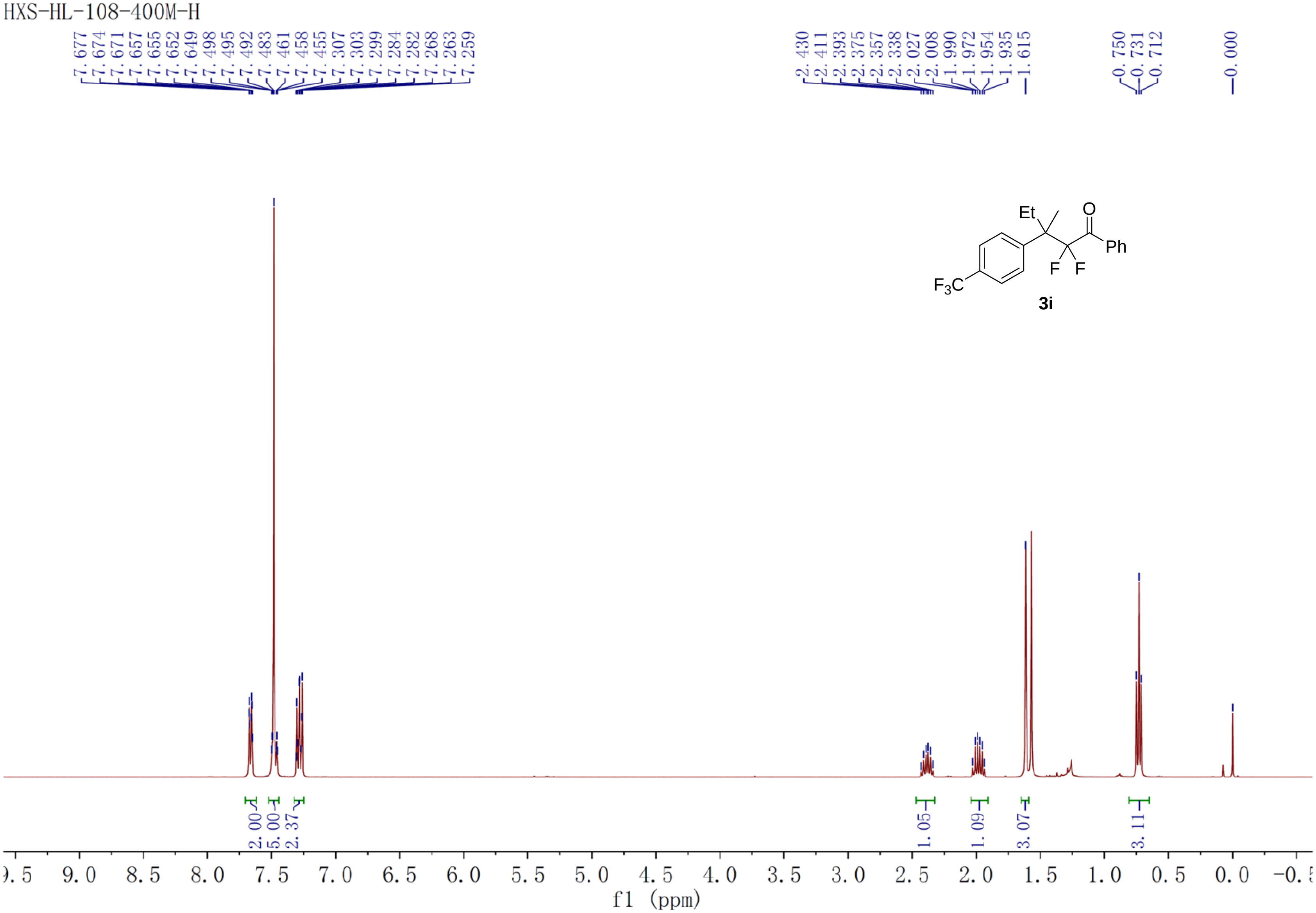

Supplementary Figure 47. ^{1}H NMR (400 MHz, $CDCl_3$) spectra for compound **3i**

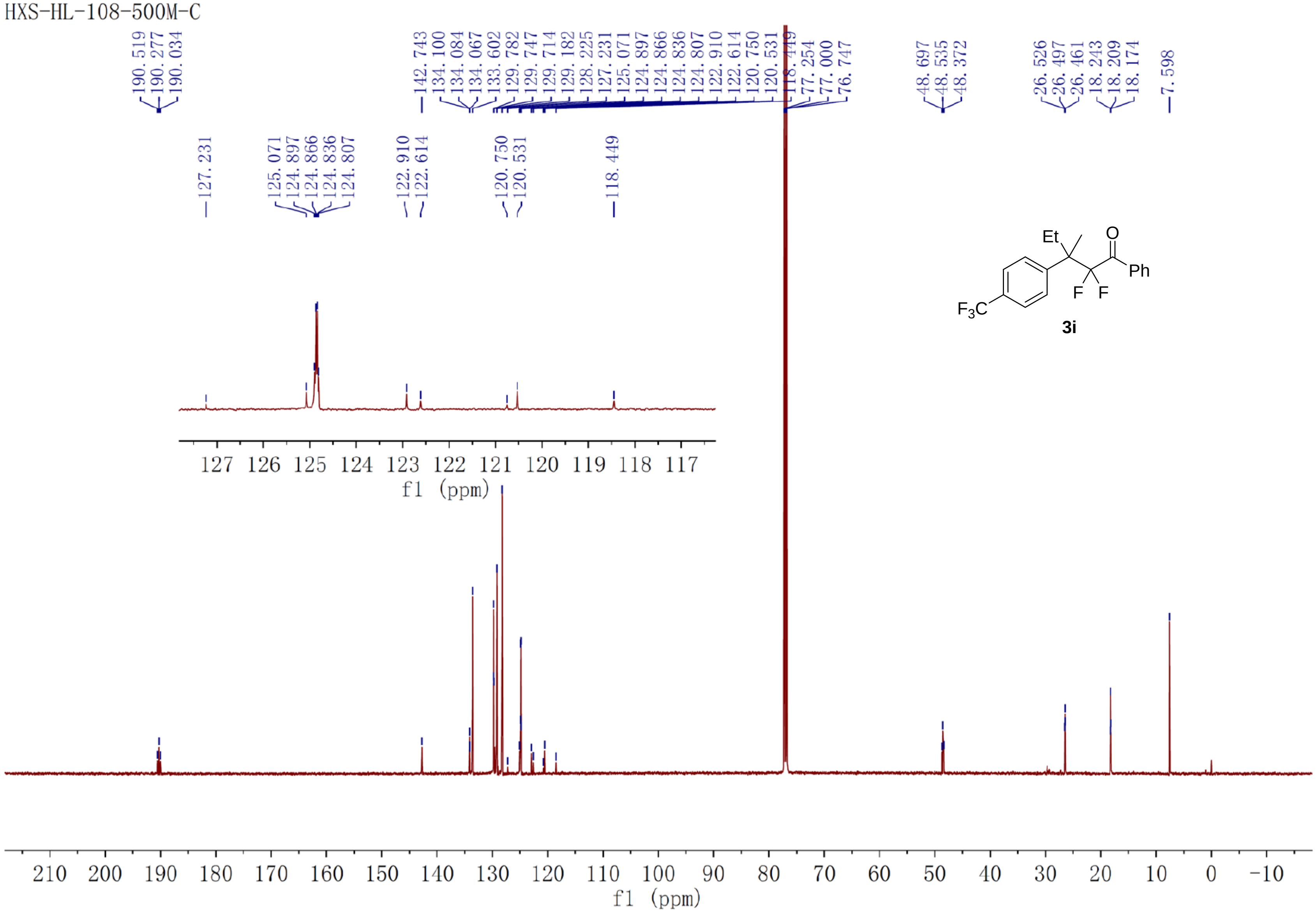

Supplementary Figure 48. ^{13}C NMR (125 MHz, $CDCl_3$) spectra for compound **3i**

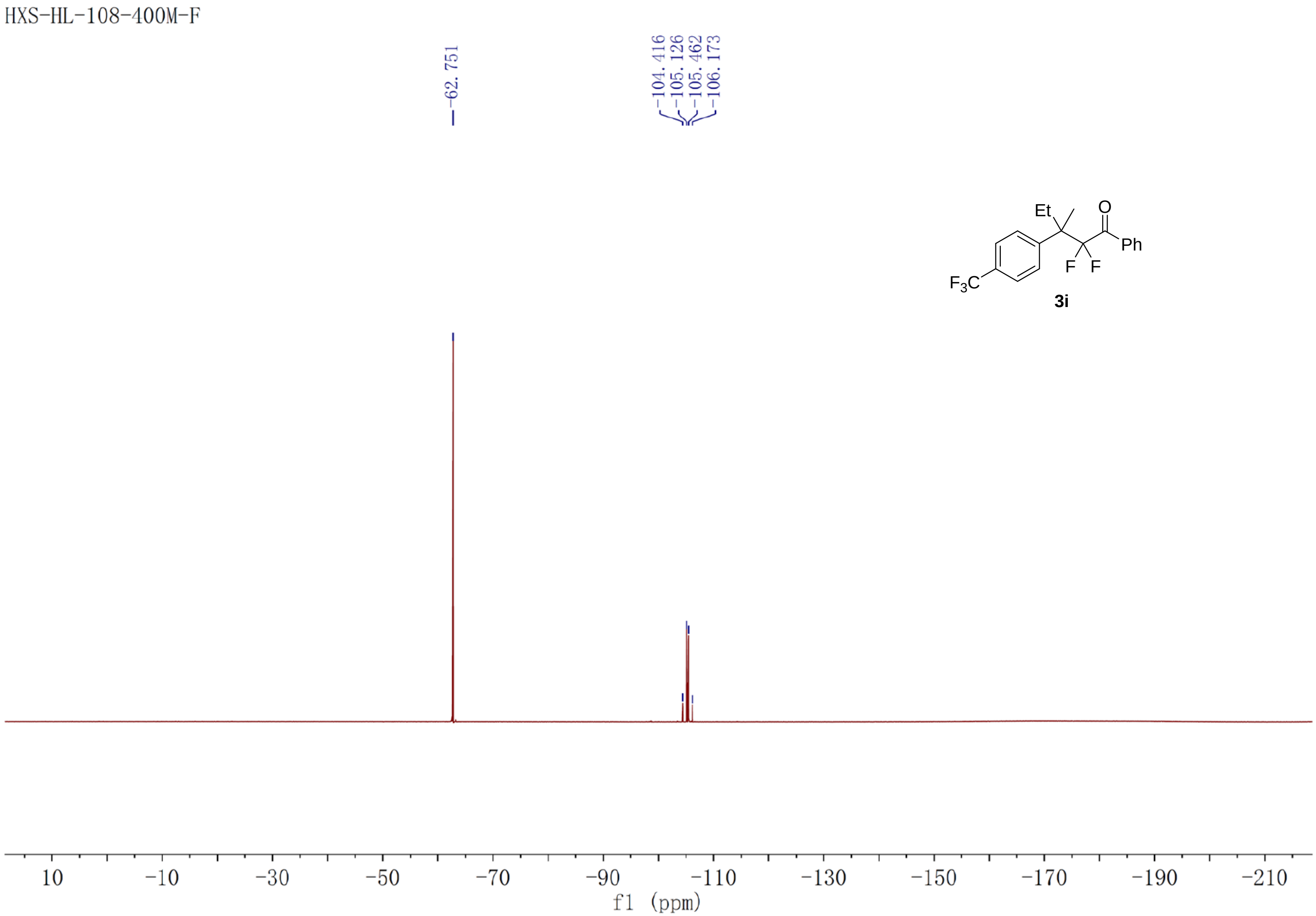

Supplementary Figure 49. ^{19}F NMR (376 MHz, $CDCl_3$) spectra for compound **3i**

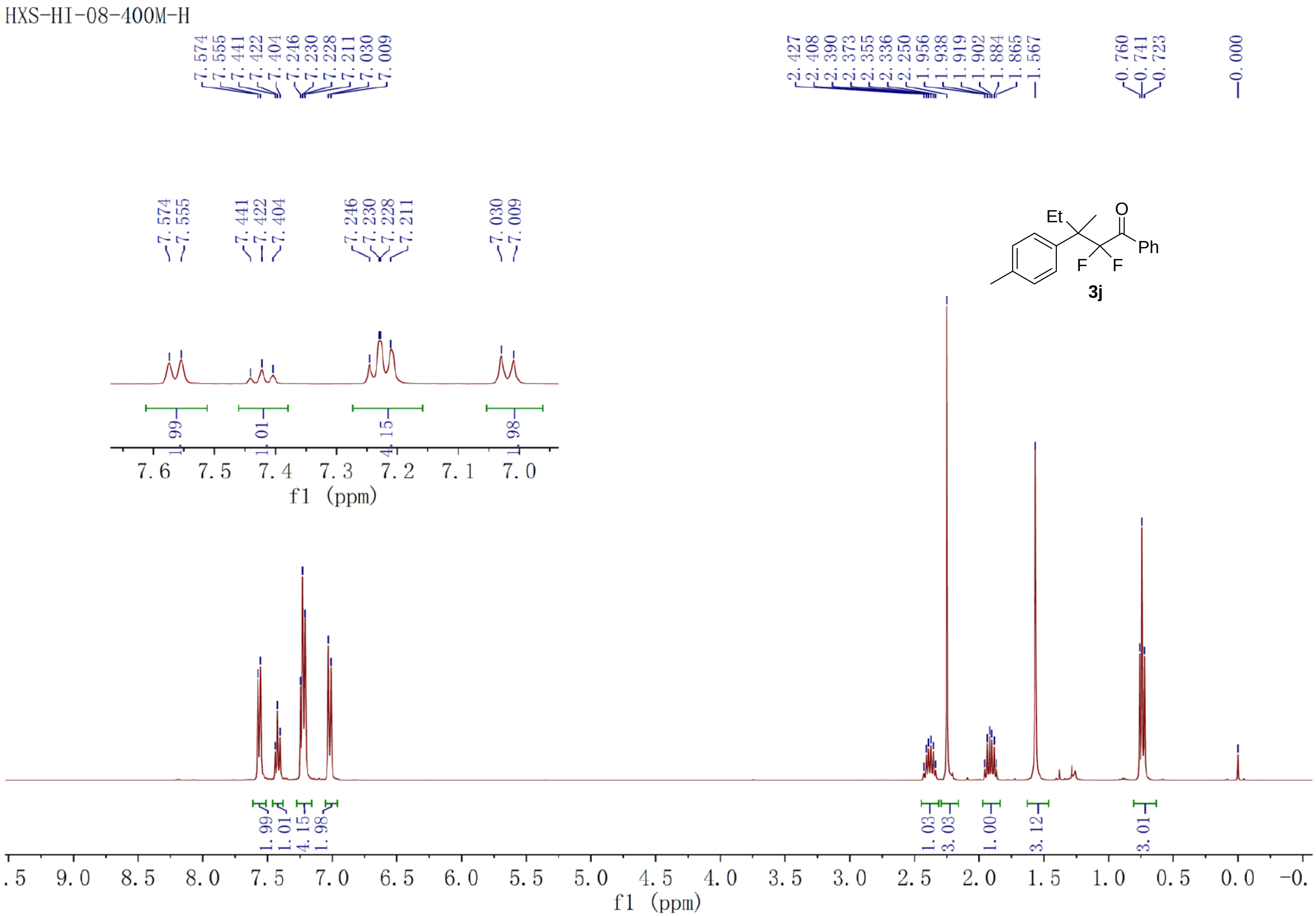

Supplementary Figure 50. ^{1}H NMR (400 MHz, $CDCl_3$) spectra for compound **3j**

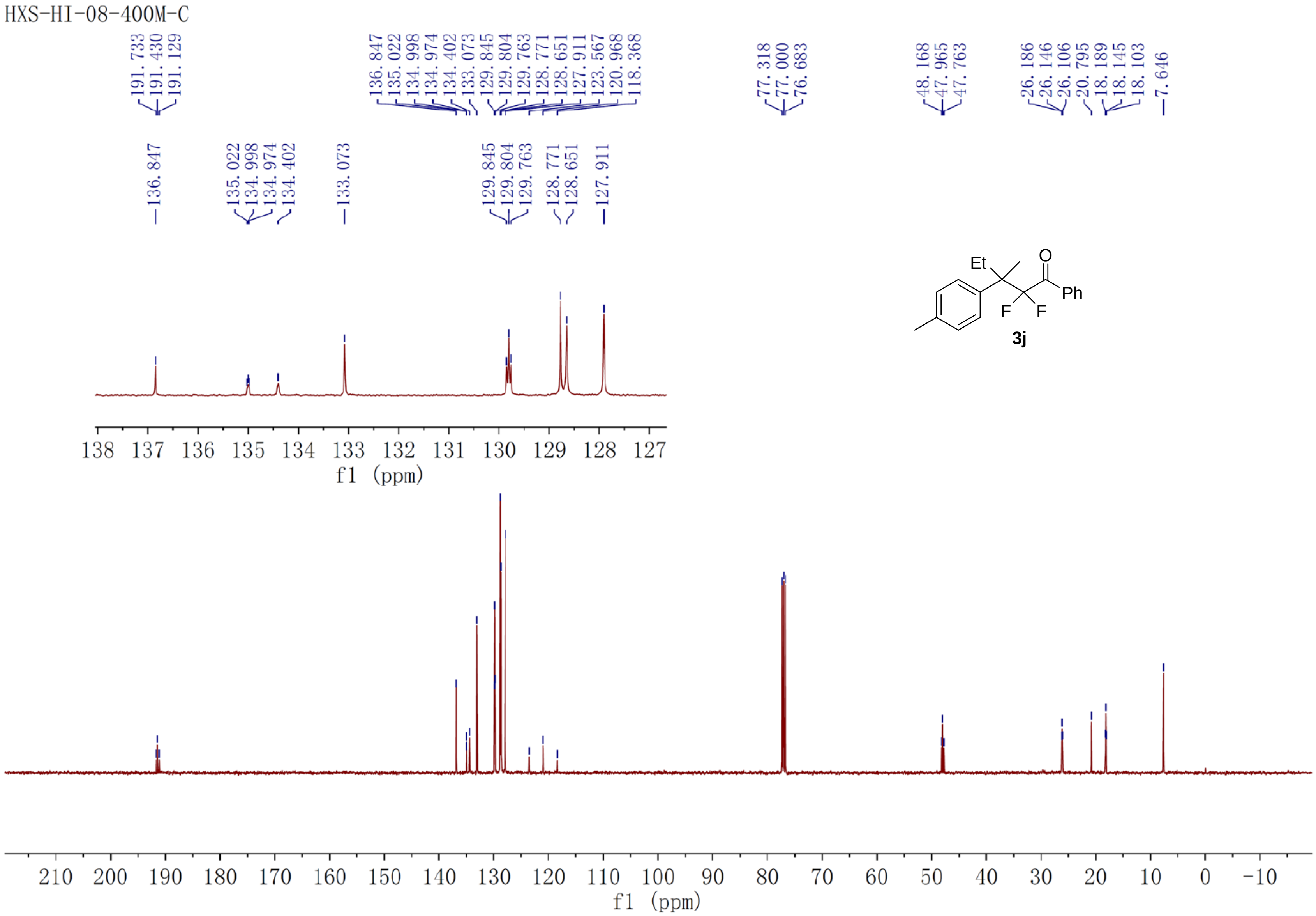

Supplementary Figure 51. ^{13}C NMR (100 MHz, $CDCl_3$) spectra for compound **3j**

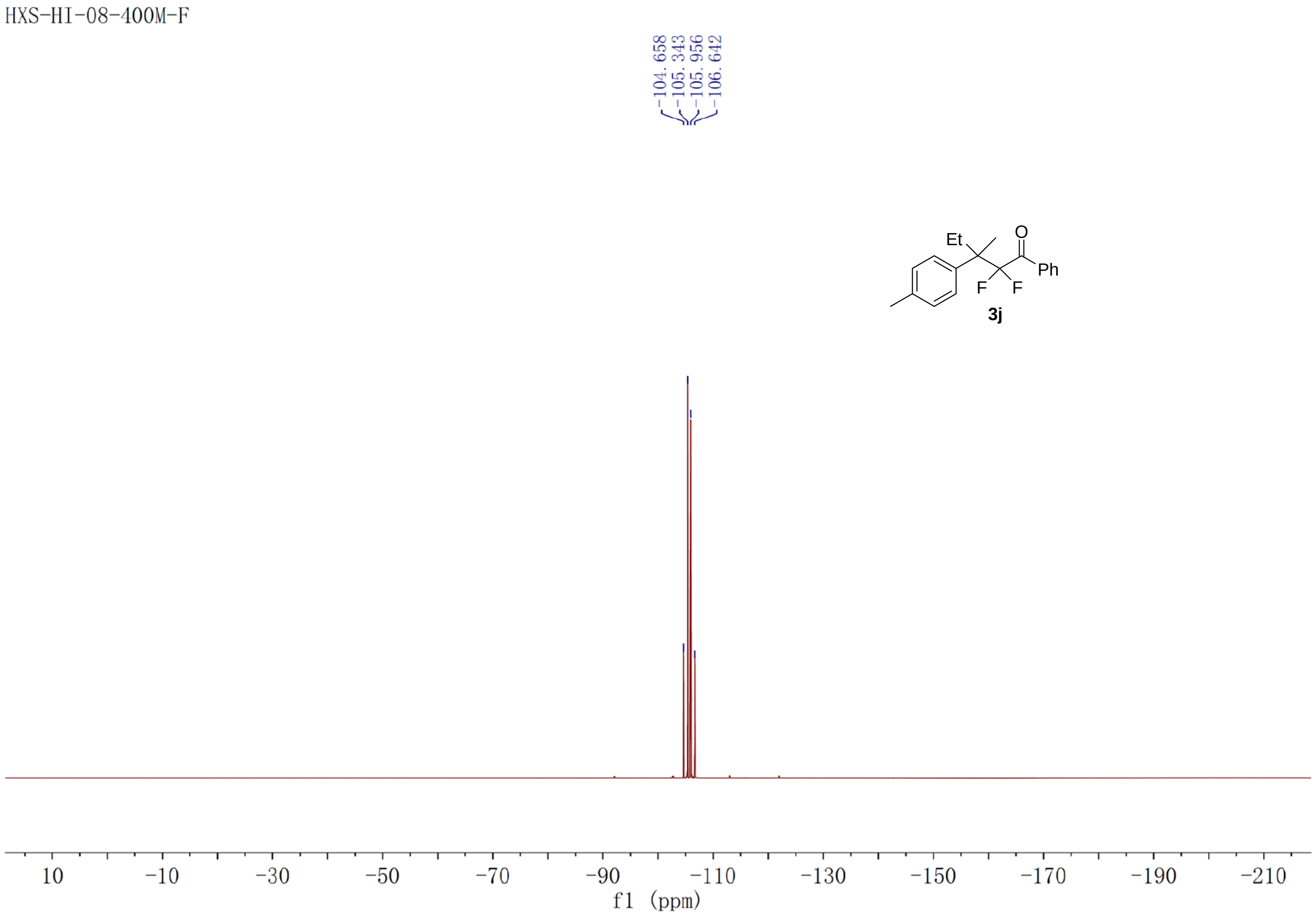

Supplementary Figure 52. ^{19}F NMR (376 MHz, $CDCl_3$) spectra for compound **3j**

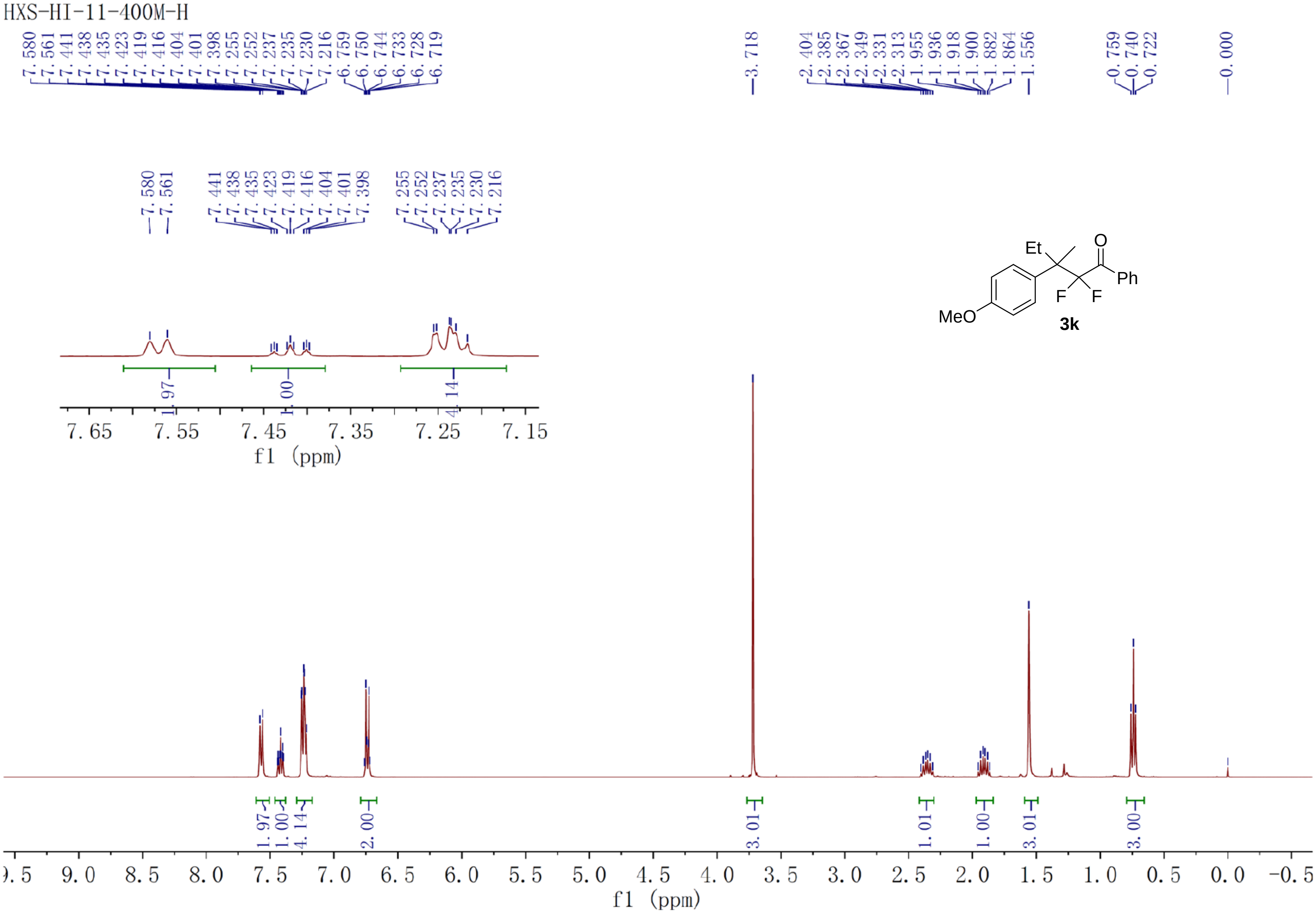

Supplementary Figure 53. ^{1}H NMR (400 MHz, $CDCl_3$) spectra for compound **3k**

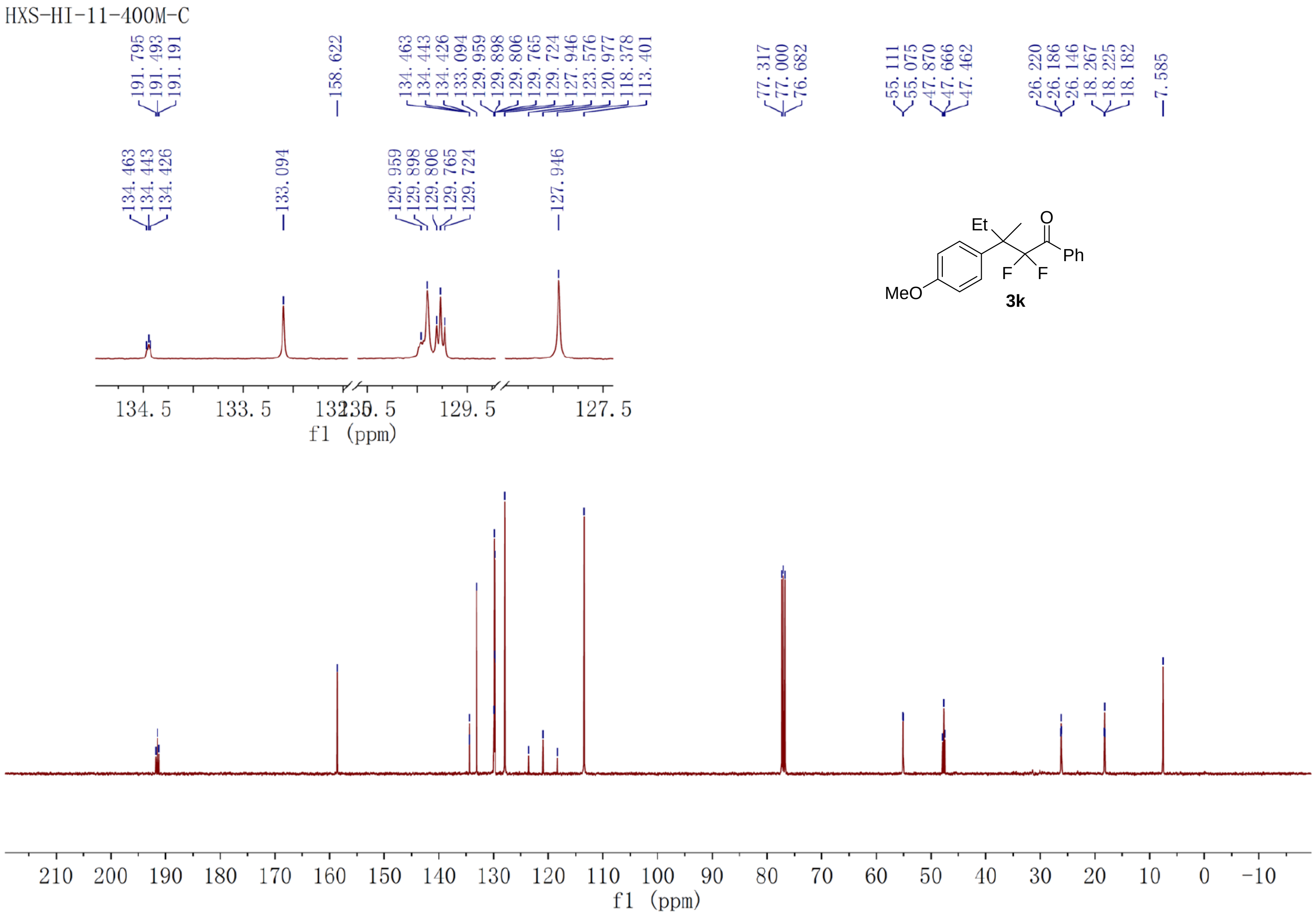

Supplementary Figure 54. ^{13}C NMR (100 MHz, $CDCl_3$) spectra for compound **3k**

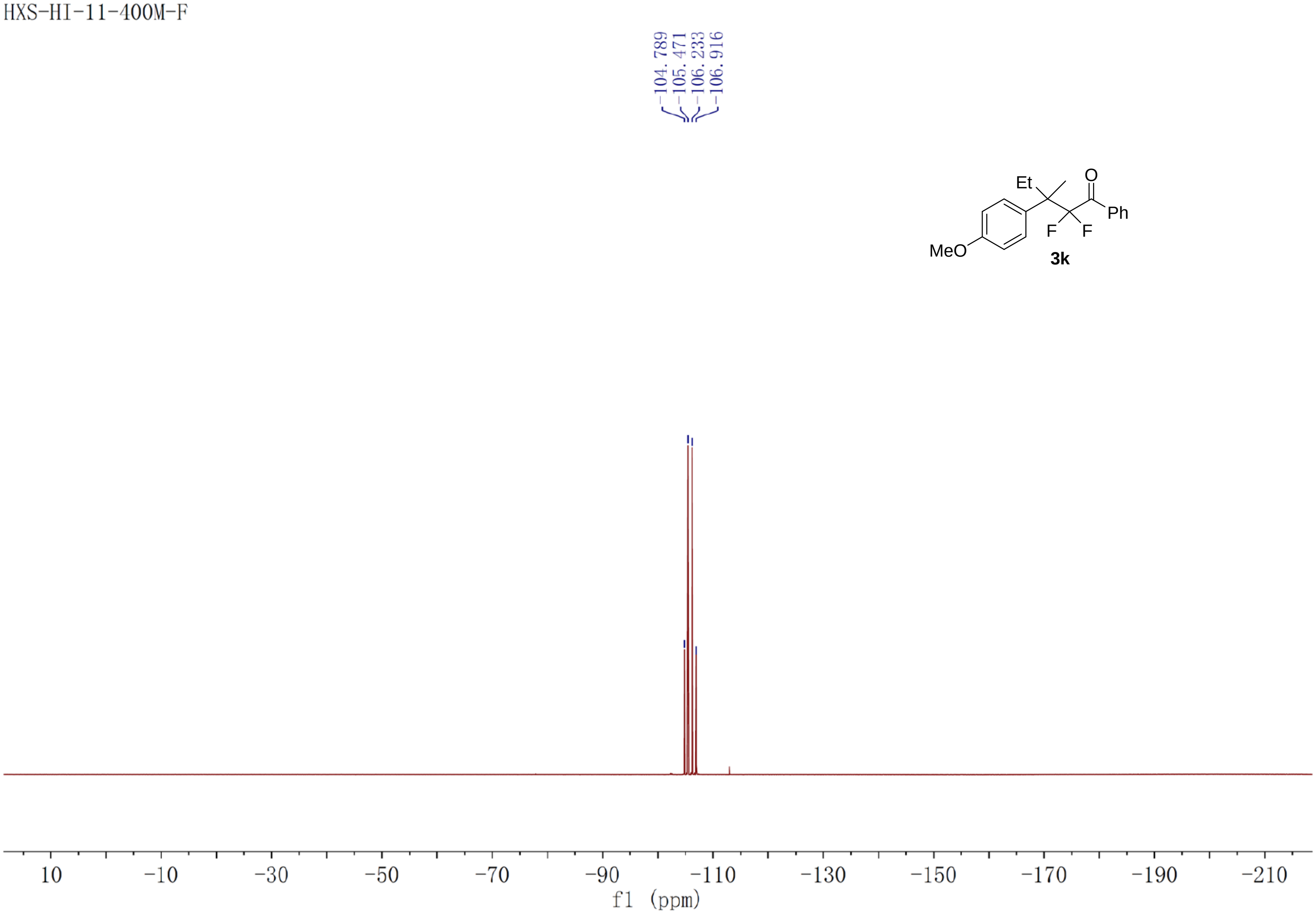

3k

Supplementary Figure 55. ^{19}F NMR (376 MHz, $CDCl_3$) spectra for compound **3k**

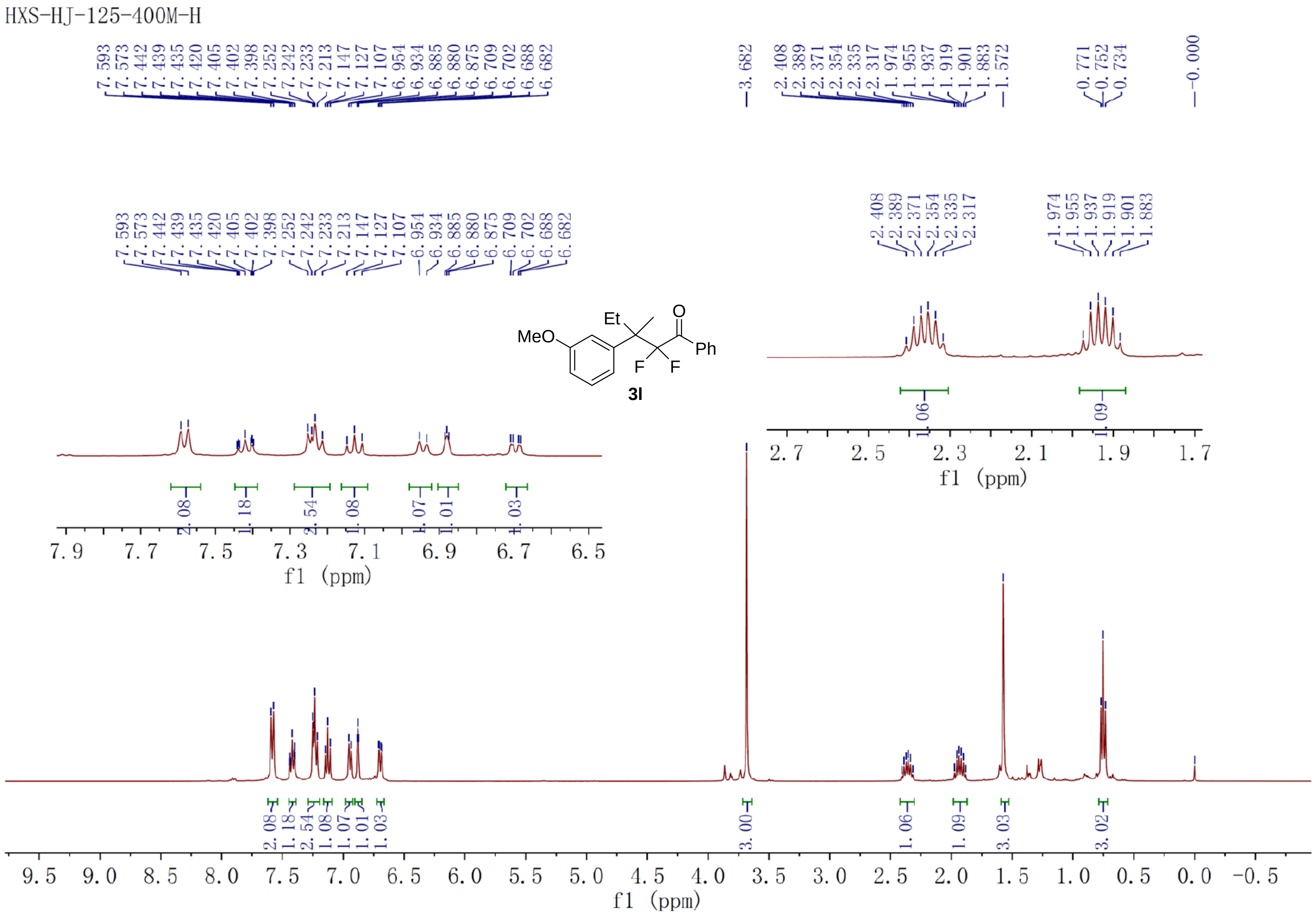

Supplementary Figure 56. ^{1}H NMR (400 MHz, $CDCl_3$) spectra for compound **3l**

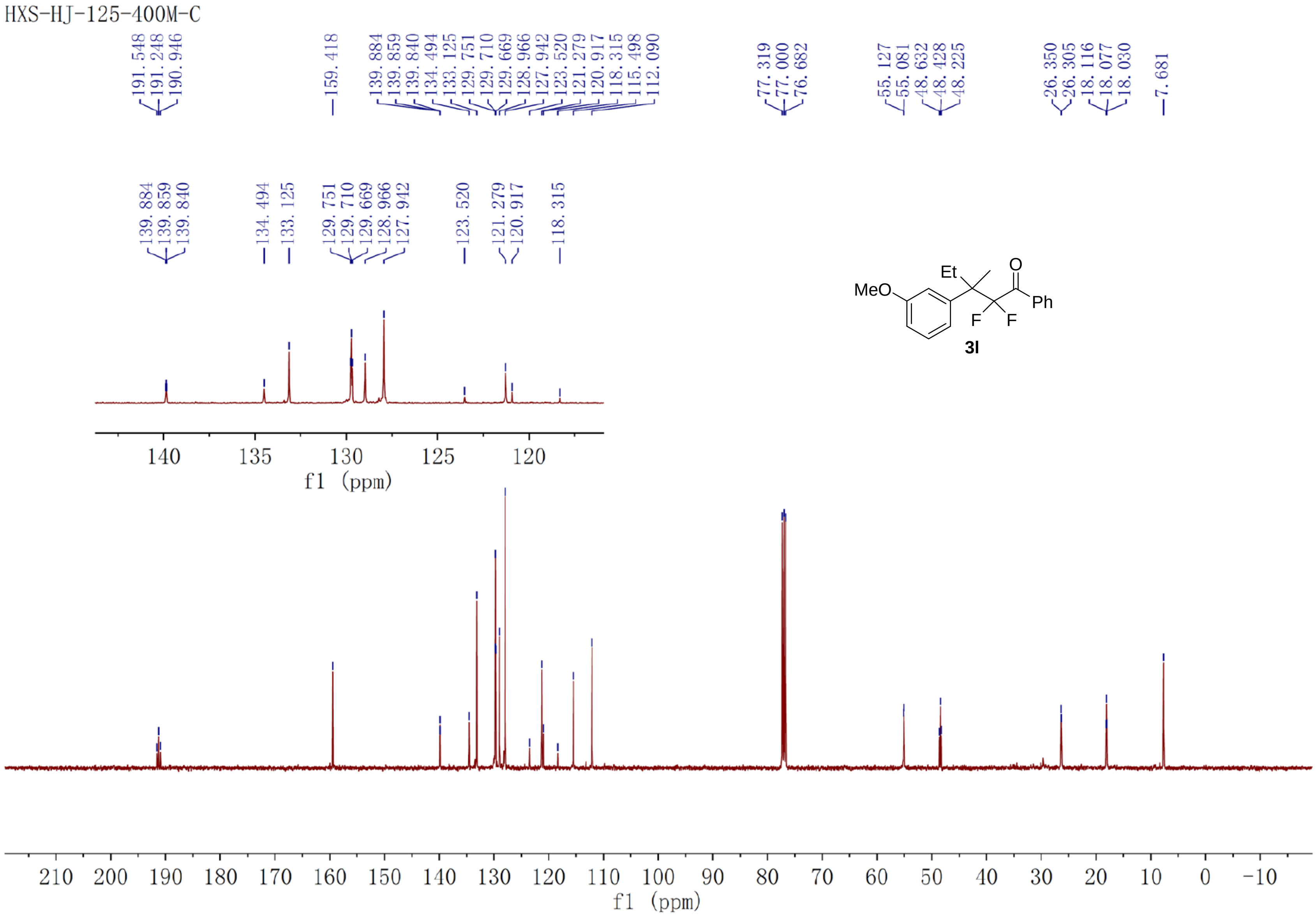

Supplementary Figure 57. ^{13}C NMR (100 MHz, $CDCl_3$) spectra for compound **3l**

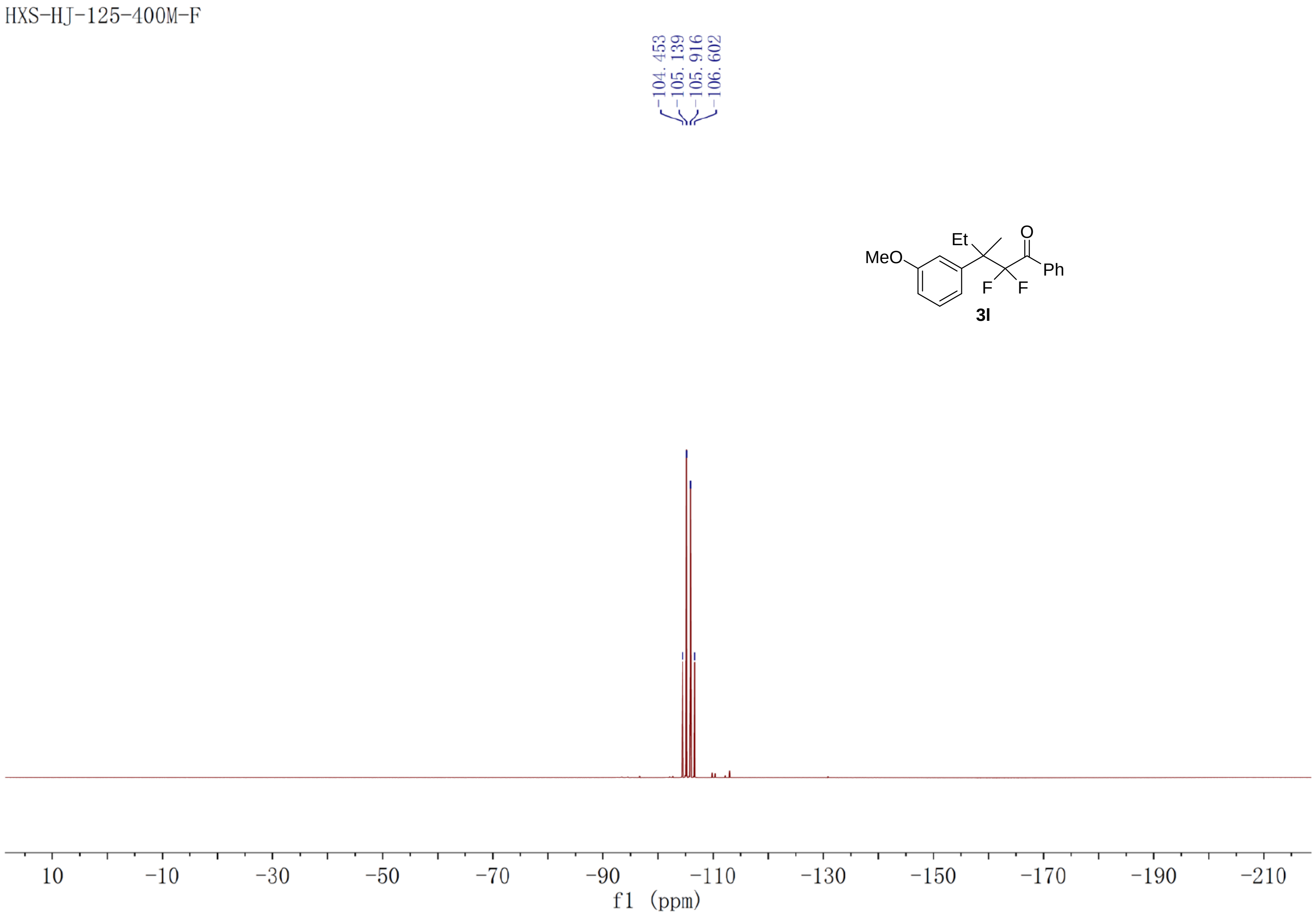

Supplementary Figure 58. ^{19}F NMR (376 MHz, $CDCl_3$) spectra for compound **3l**

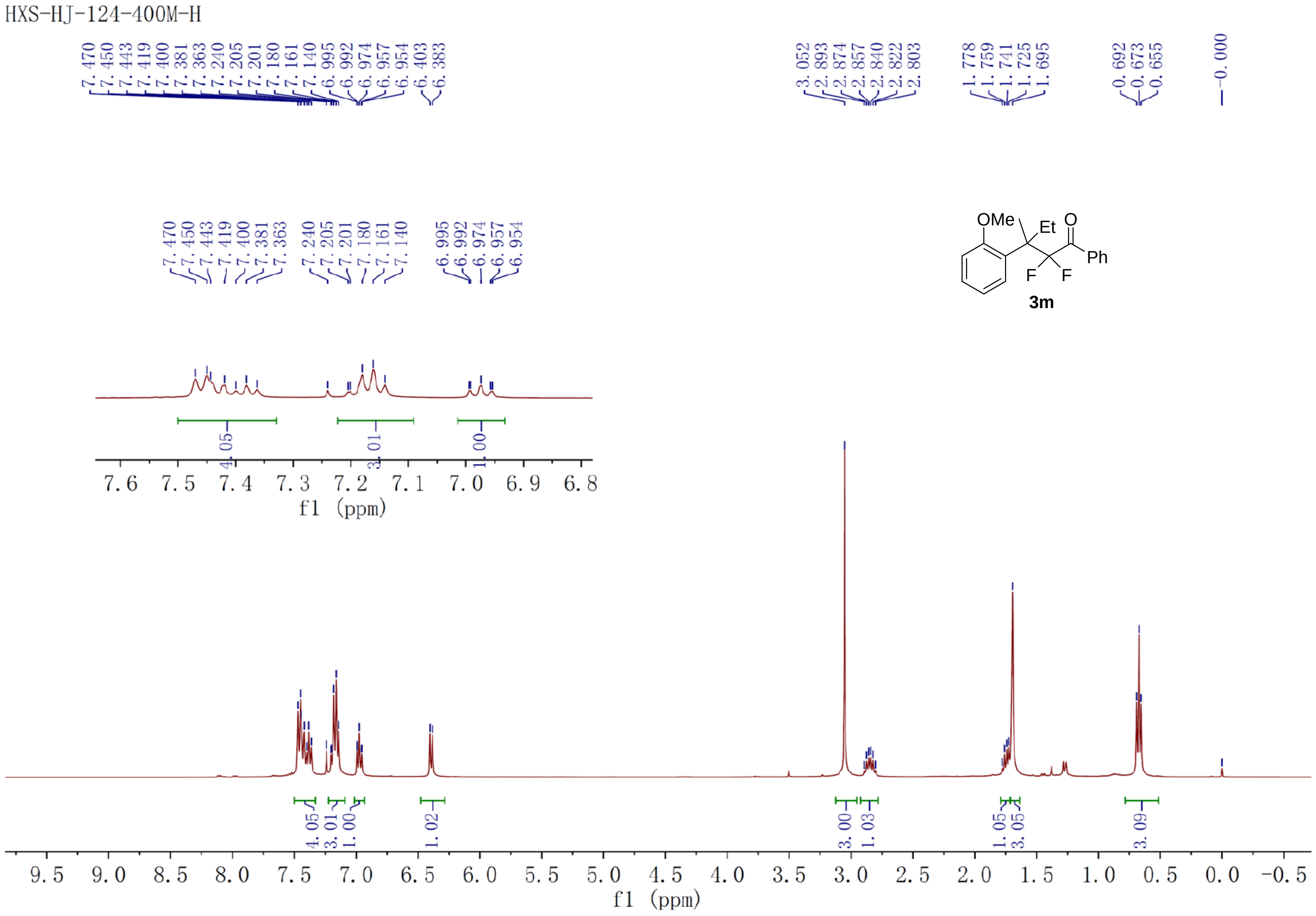

Supplementary Figure 59. ^{1}H NMR (400 MHz, $CDCl_3$) spectra for compound **3m**

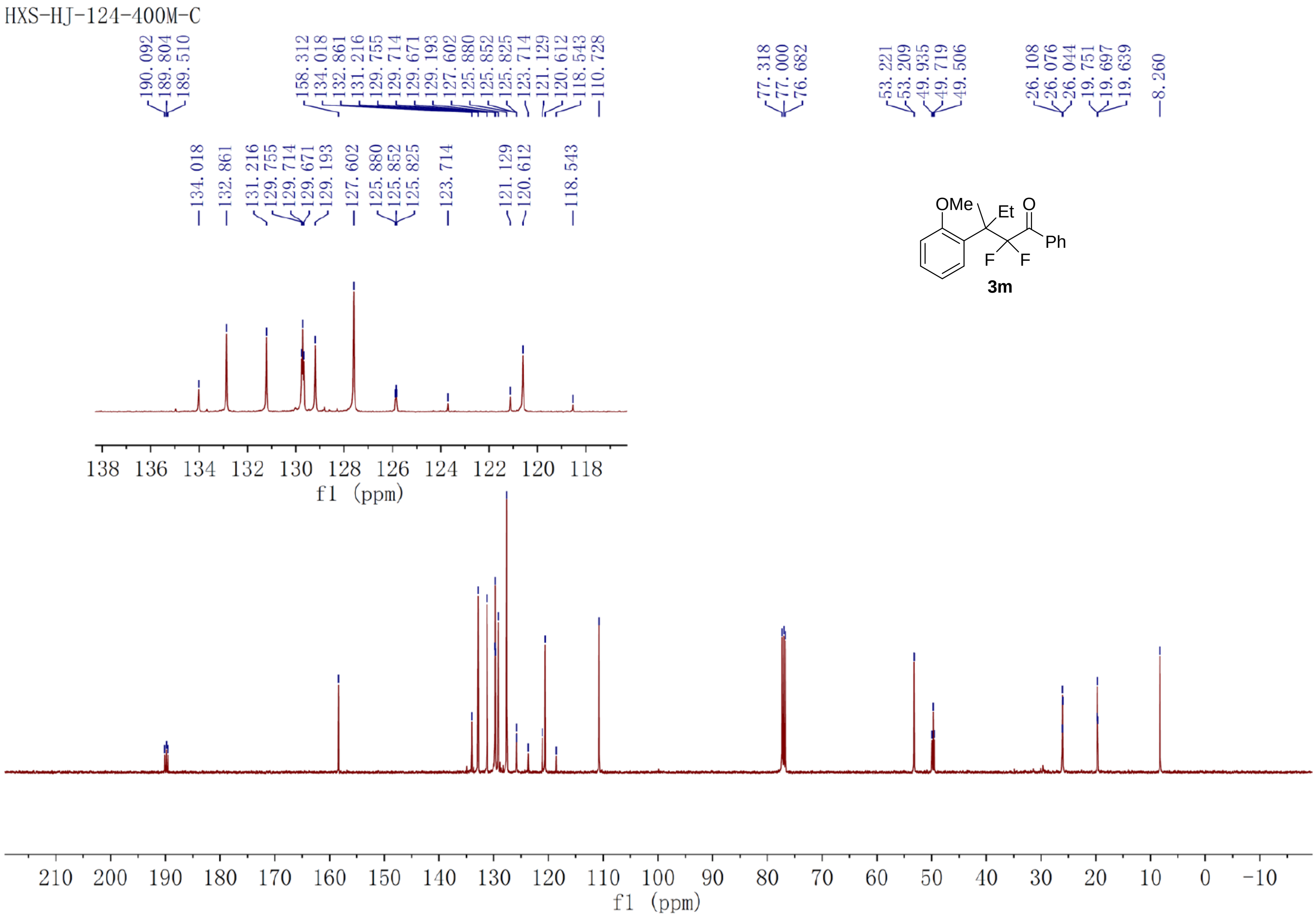

Supplementary Figure 60. ^{13}C NMR (100 MHz, $CDCl_3$) spectra for compound **3m**

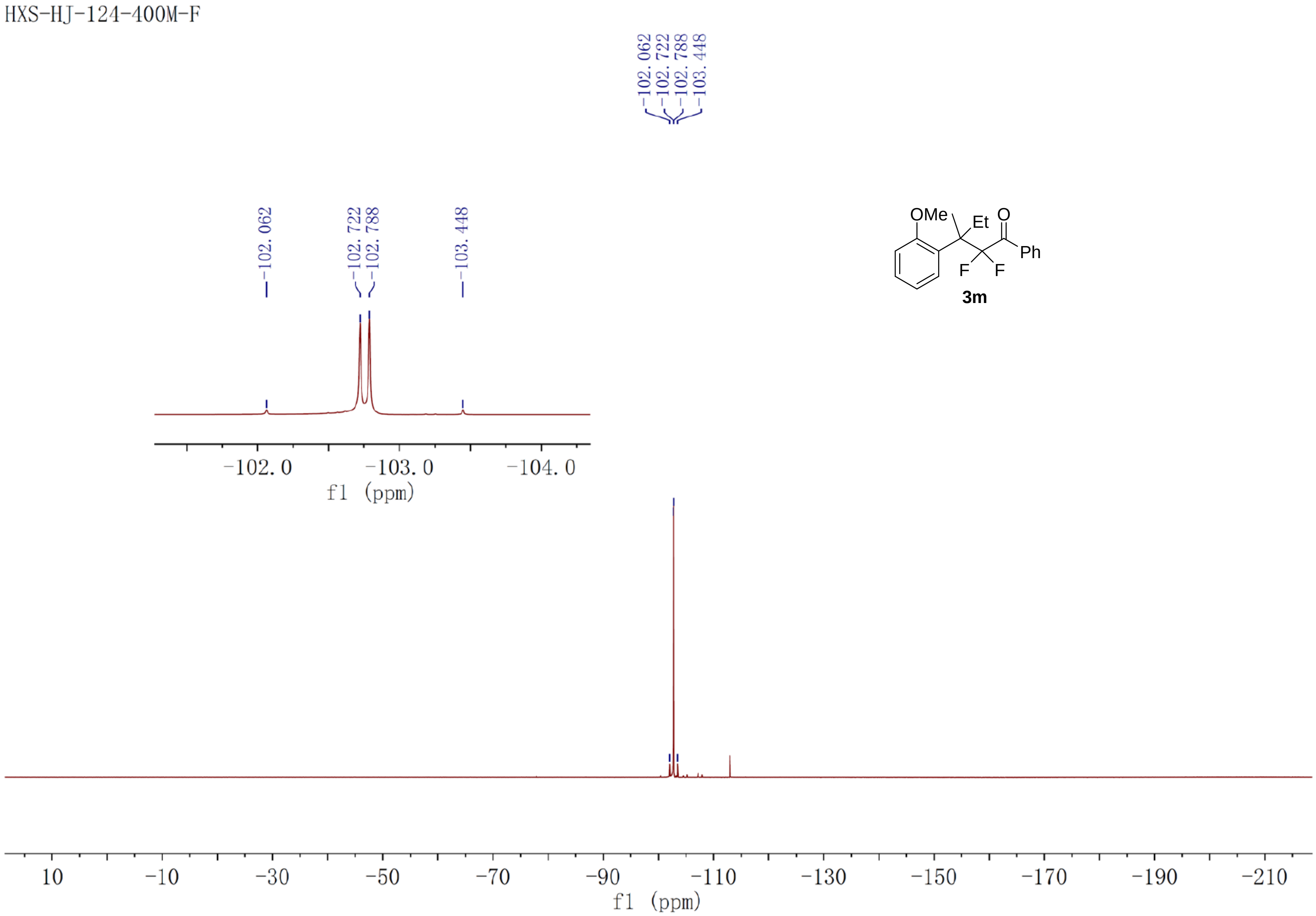

Supplementary Figure 61. ^{19}F NMR (376 MHz, $CDCl_3$) spectra for compound **3m**

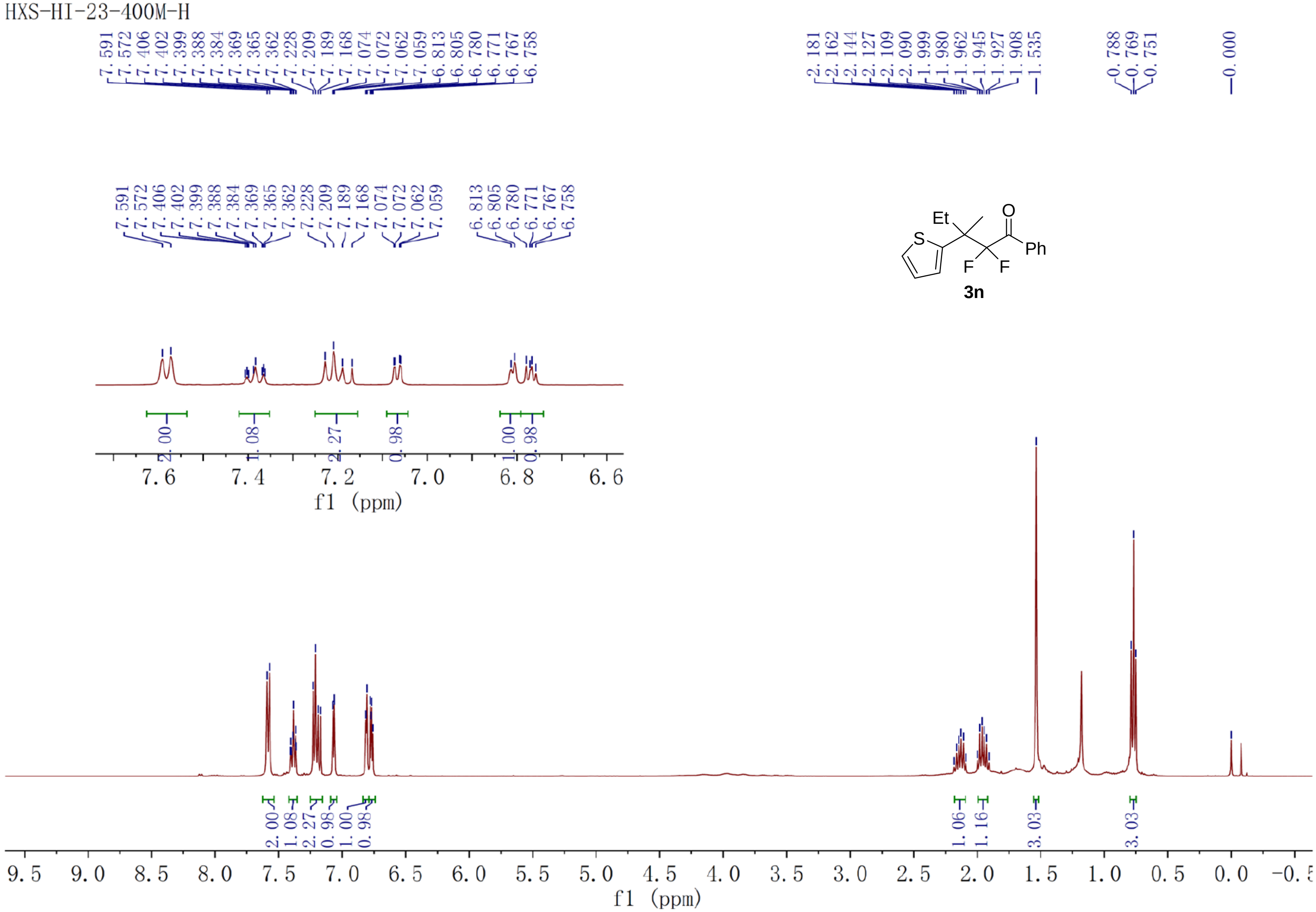

Supplementary Figure 62. ^{1}H NMR (400 MHz, $CDCl_3$) spectra for compound **3n**

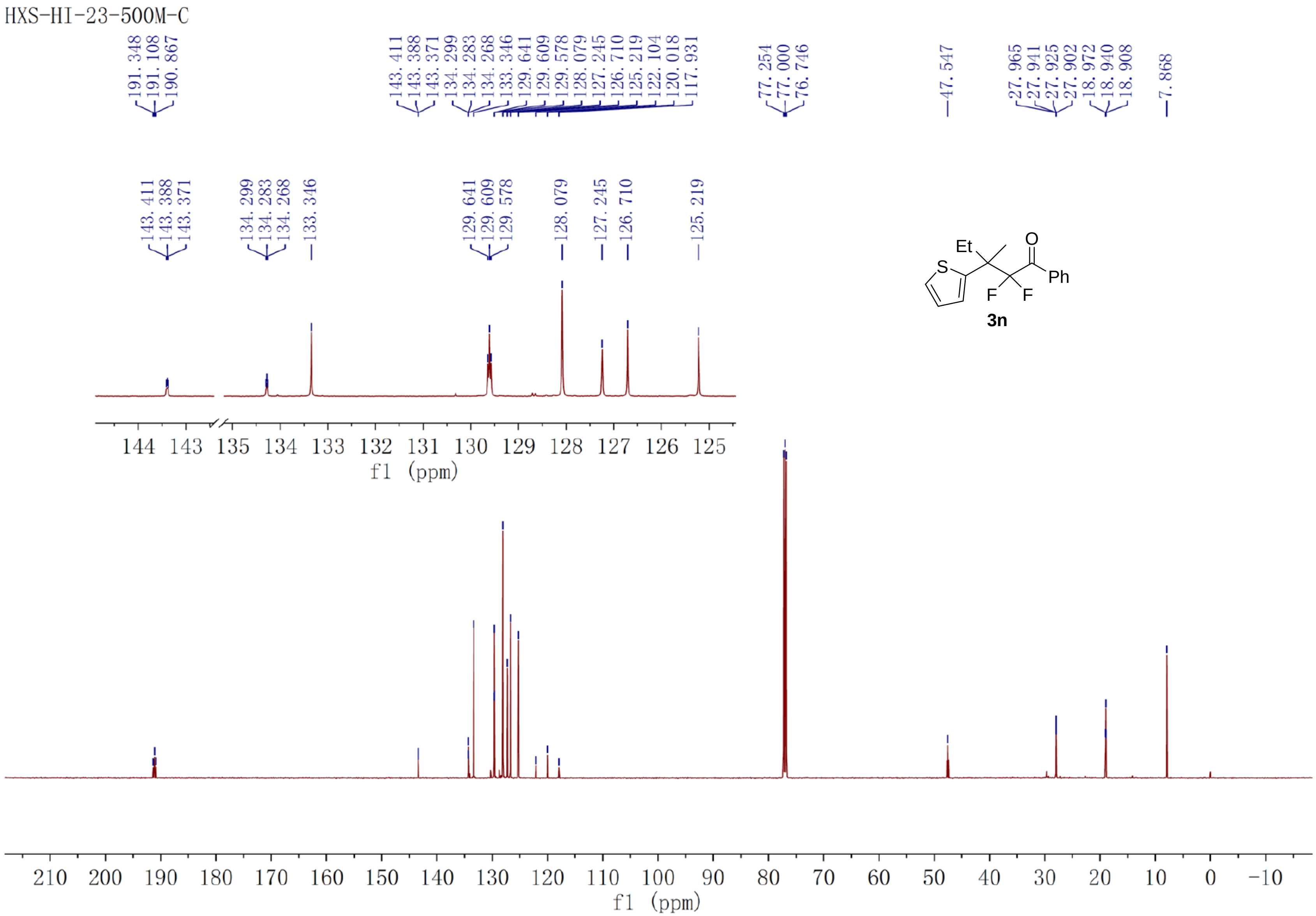

Supplementary Figure 63. ^{13}C NMR (125 MHz, $CDCl_3$) spectra for compound **3n**

HXS-HI-23-400M-F

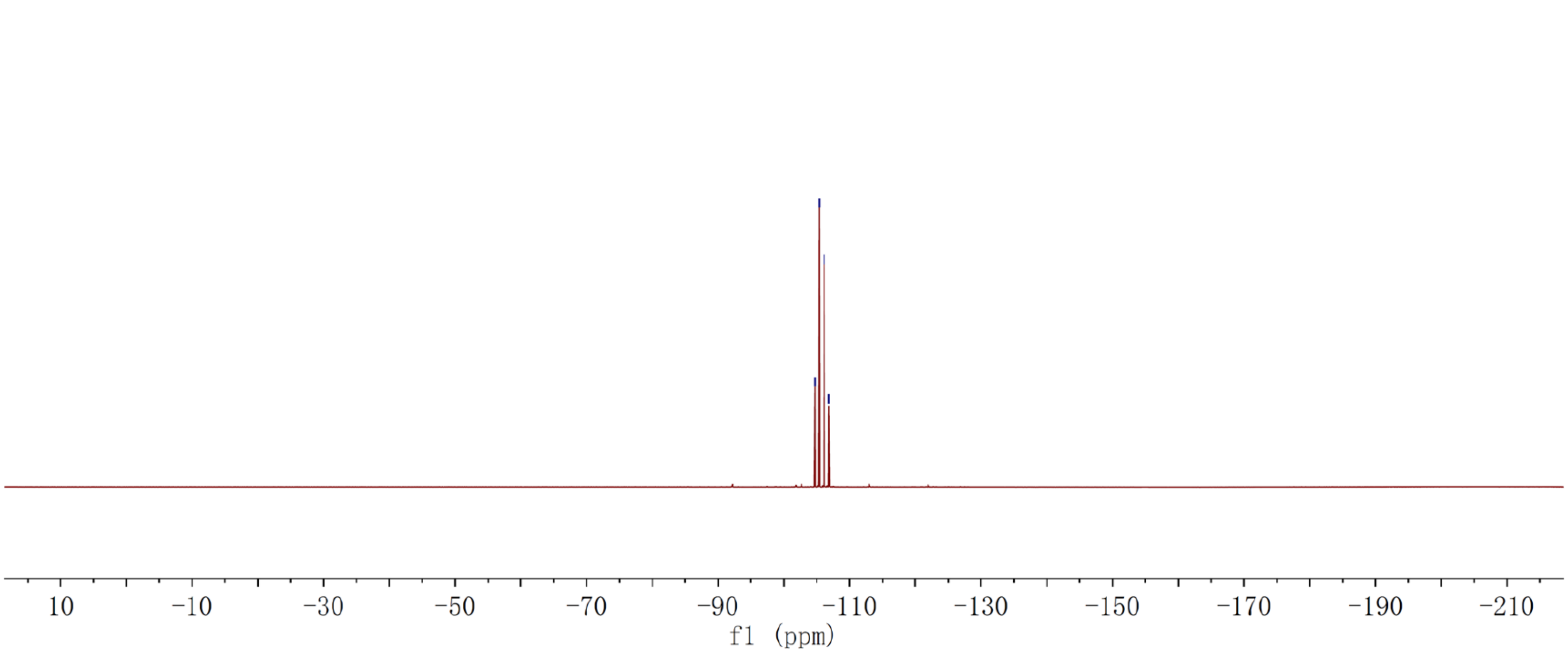

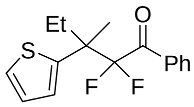

3n

Supplementary Figure 64. ^{19}F NMR (376 MHz, $CDCl_3$) spectra for compound **3n**

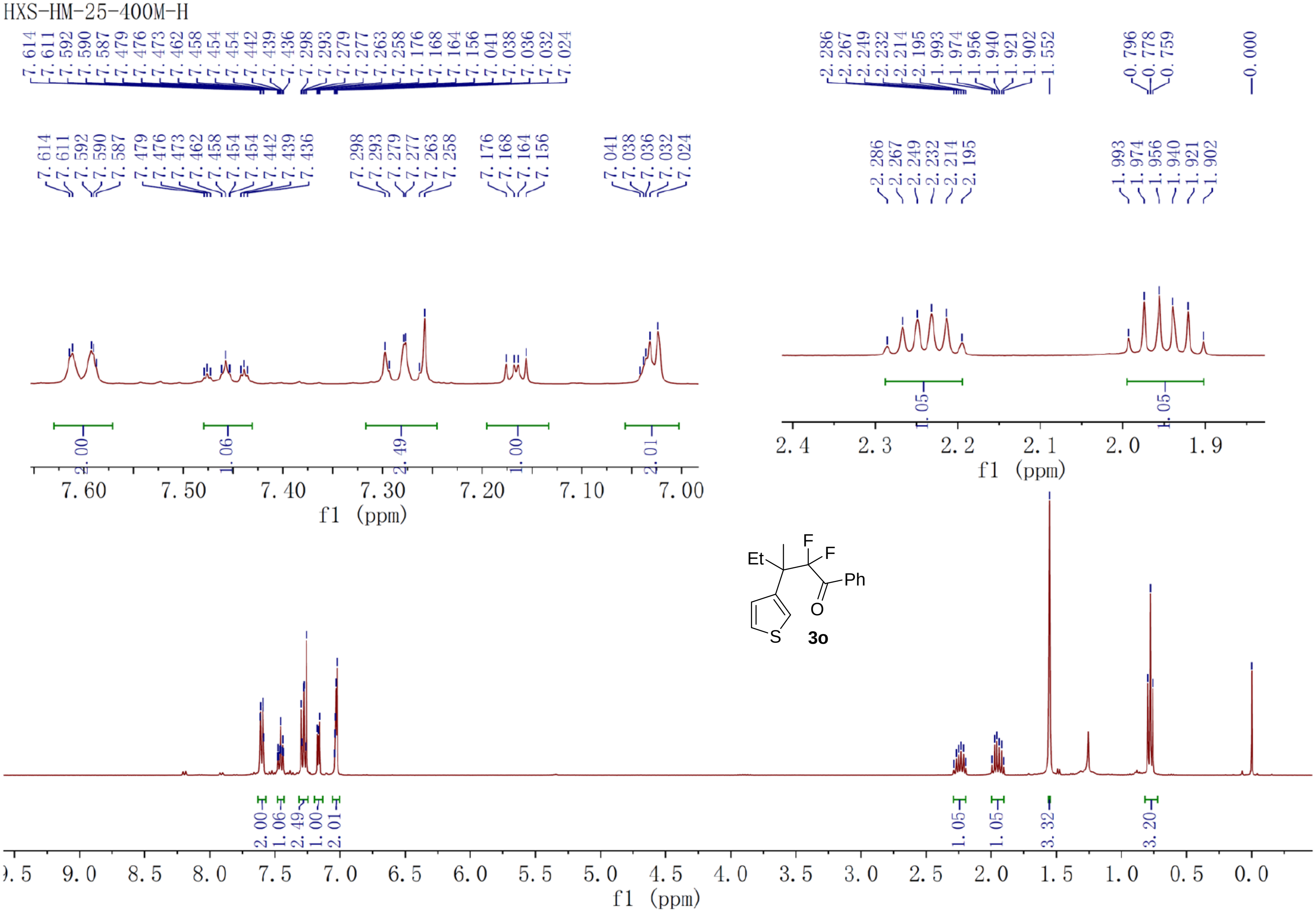

Supplementary Figure 65. ^{1}H NMR (400 MHz, $CDCl_3$) spectra for compound **3o**

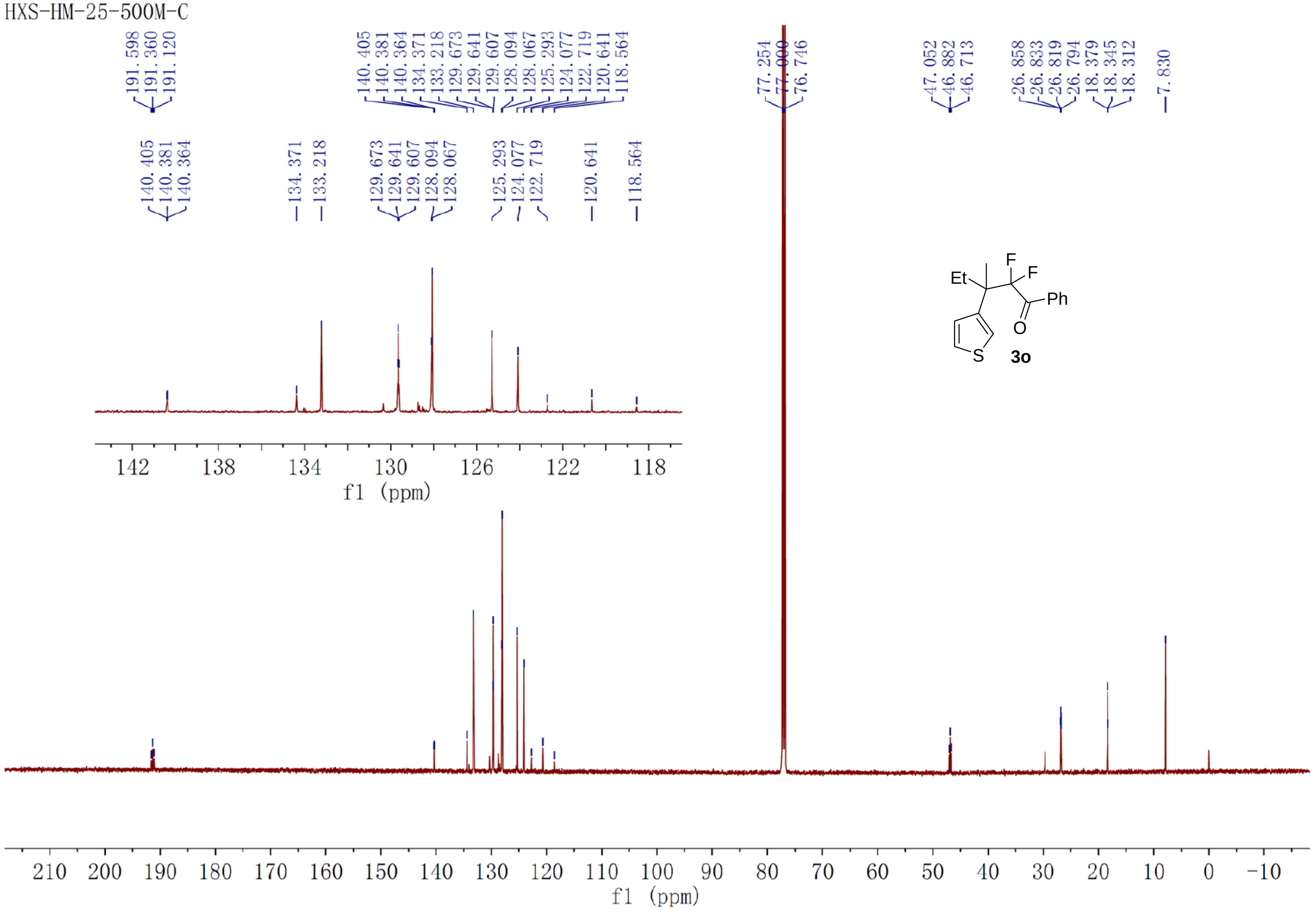

Supplementary Figure 66. ^{13}C NMR (125 MHz, $CDCl_3$) spectra for compound **3o**

HXS-HM-25-400M-F

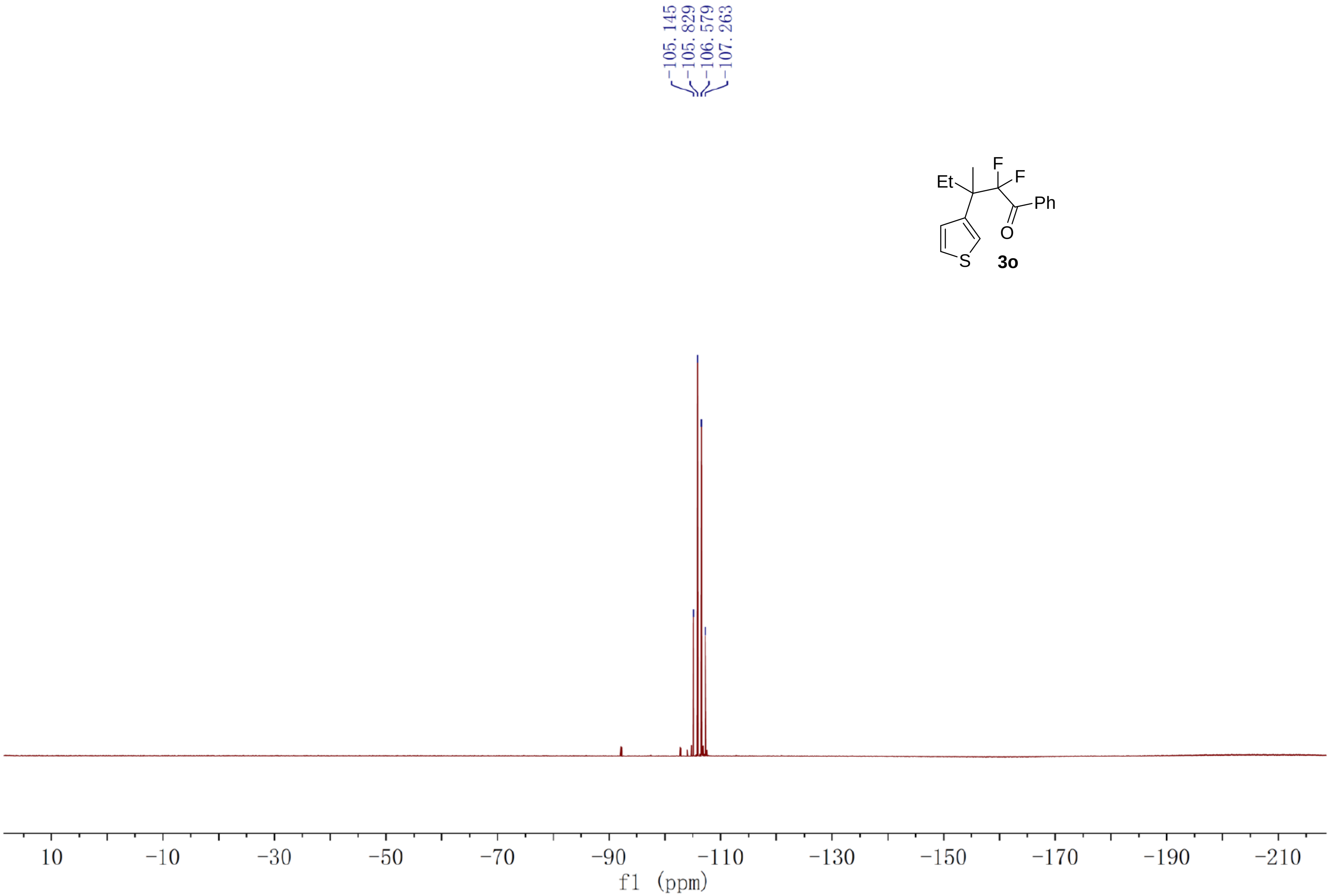

3o

Supplementary Figure 67. ^{19}F NMR (376 MHz, $CDCl_3$) spectra for compound **3o**

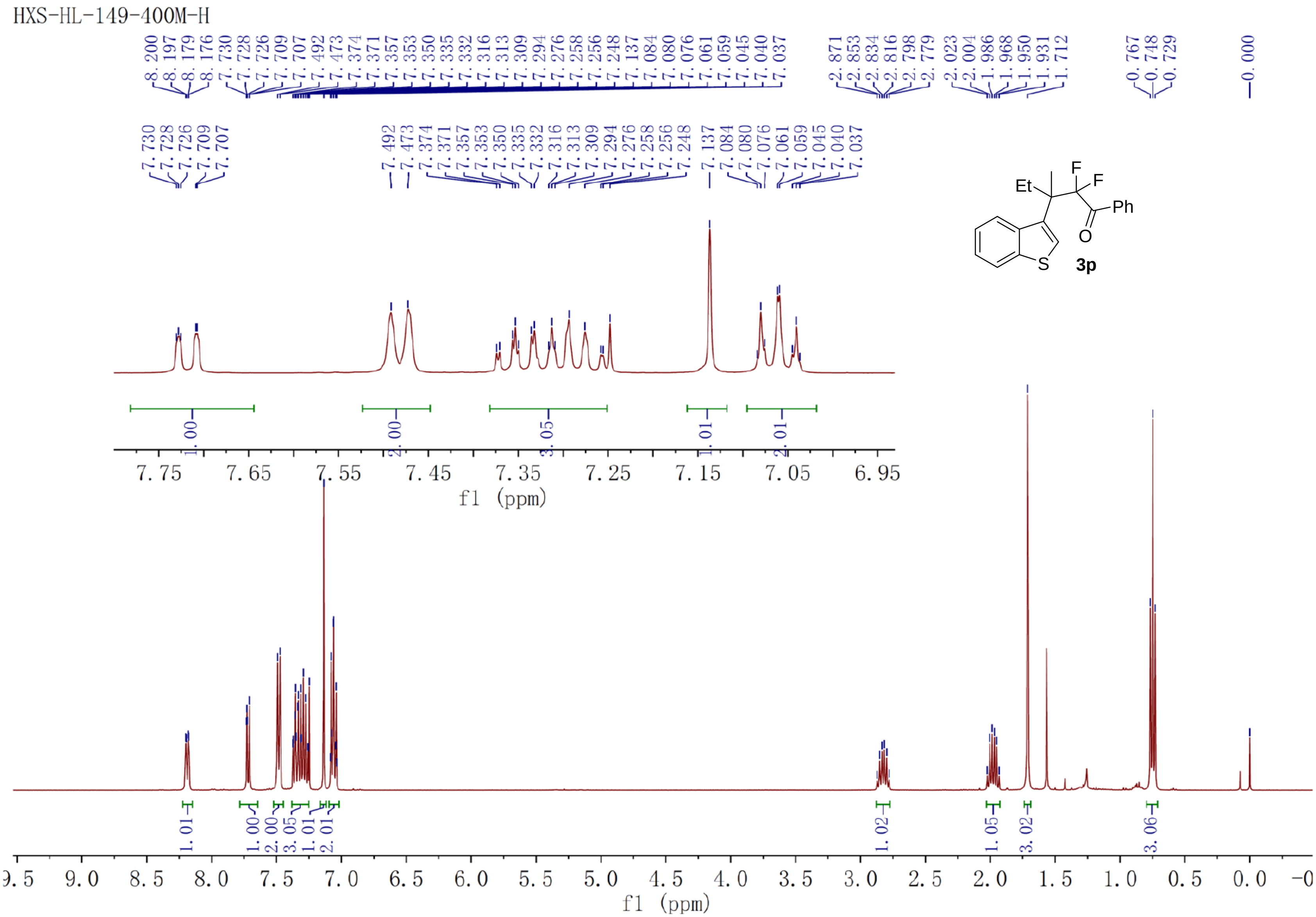

Supplementary Figure 68. ^{1}H NMR (400 MHz, $CDCl_3$) spectra for compound **3p**

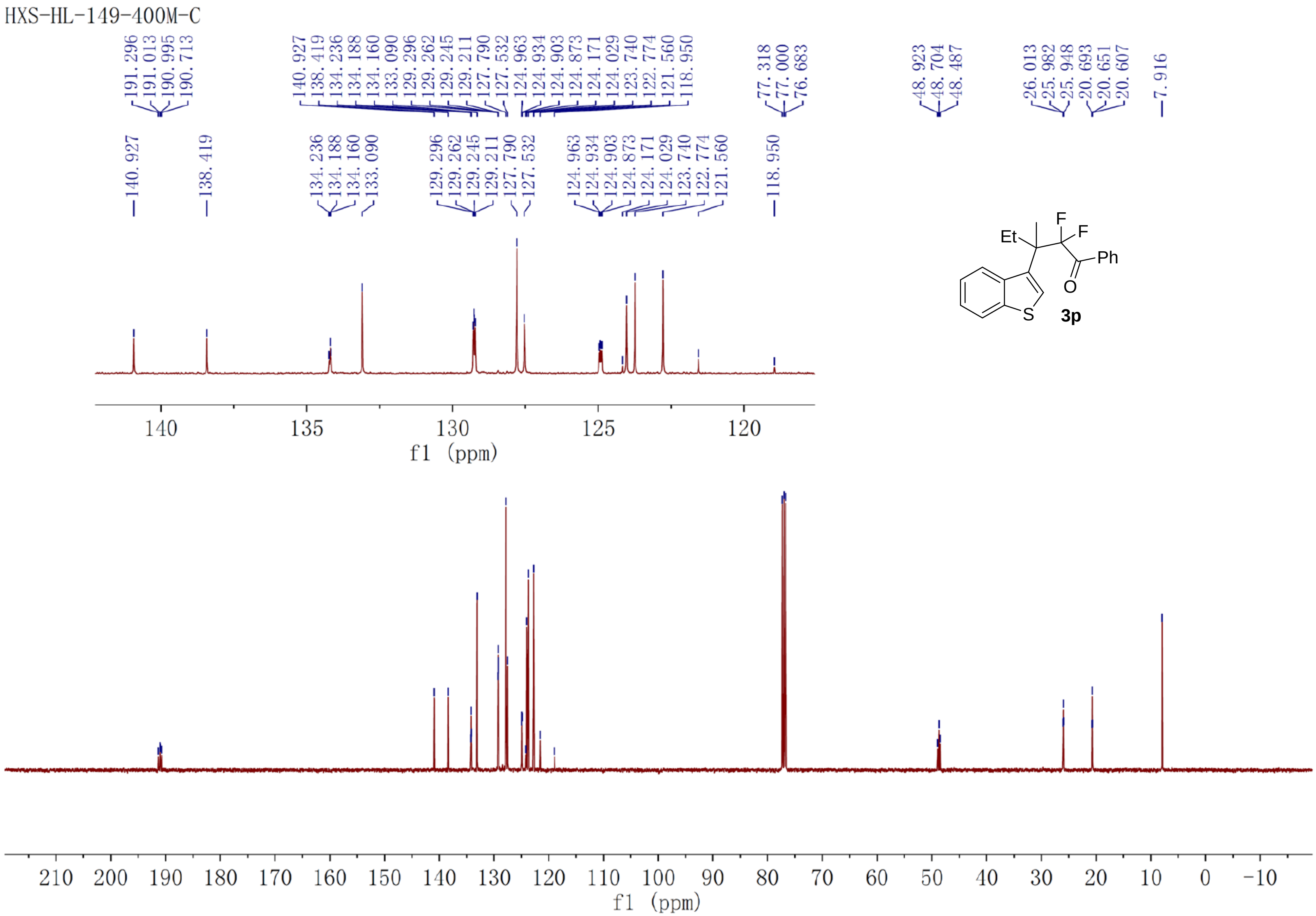

Supplementary Figure 69. ^{13}C NMR (100 MHz, $CDCl_3$) spectra for compound **3p**

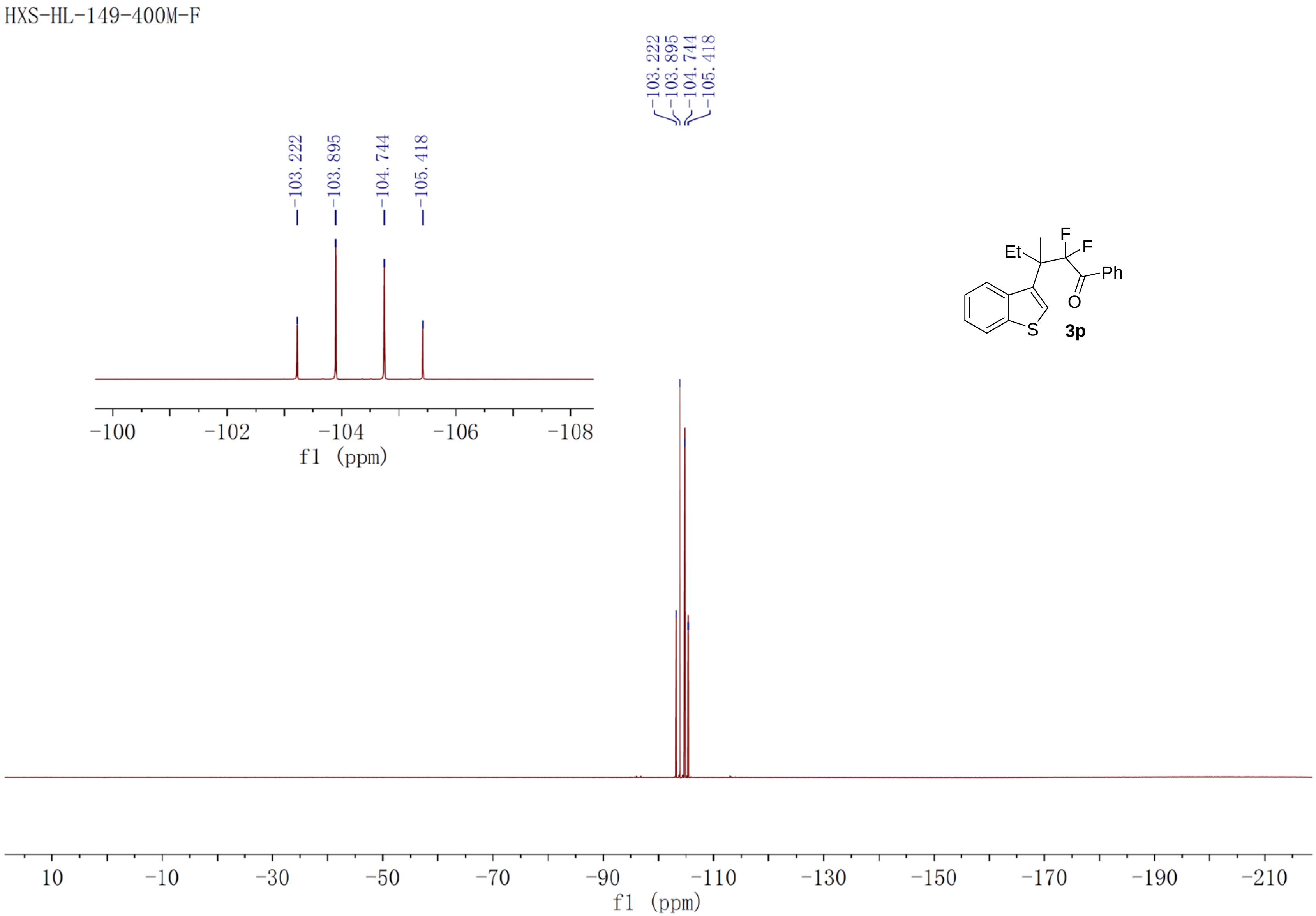

Supplementary Figure 70. ^{19}F NMR (376 MHz, $CDCl_3$) spectra for compound **3p**

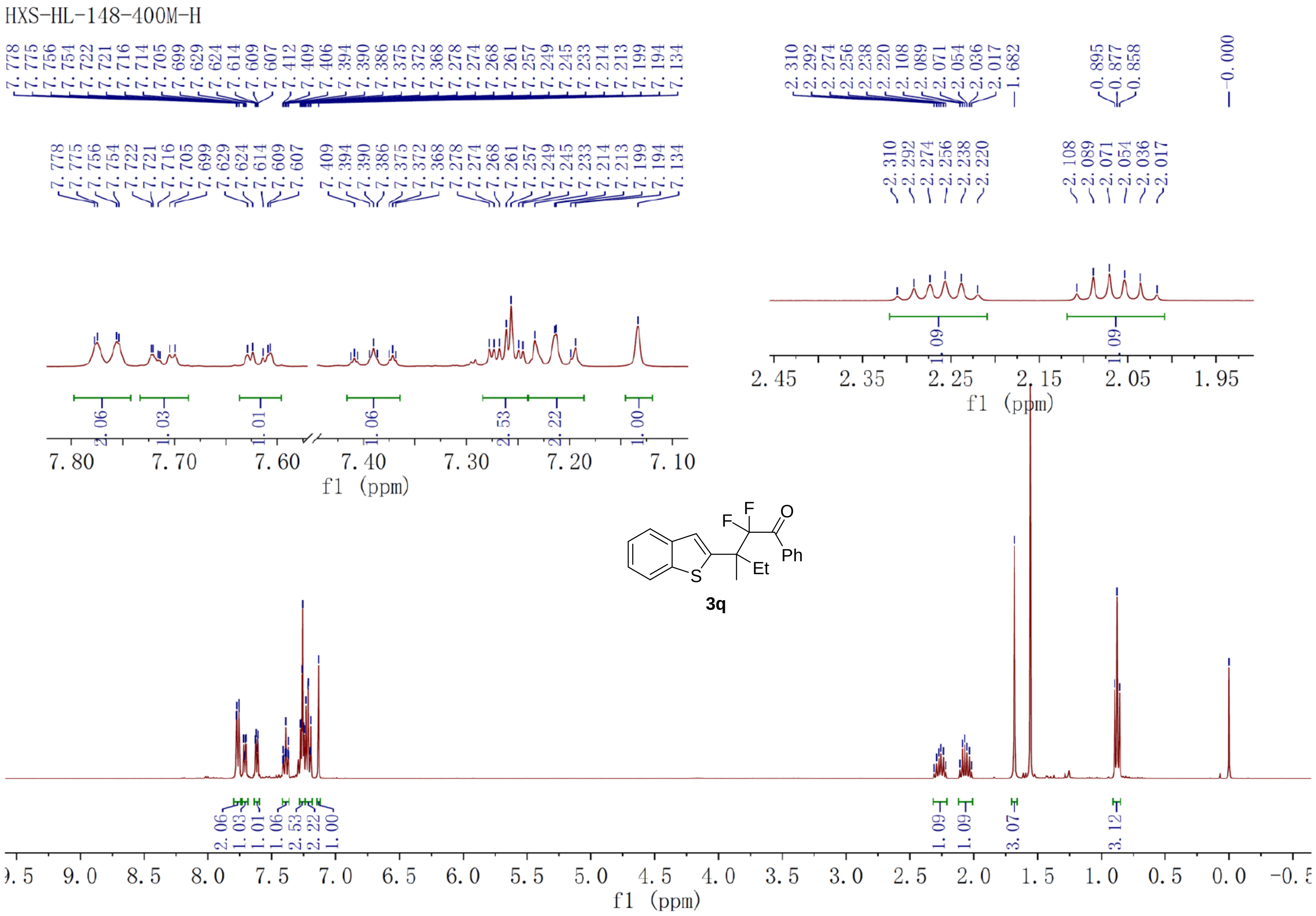

Supplementary Figure 71. ^{1}H NMR (400 MHz, $CDCl_3$) spectra for compound **3q**

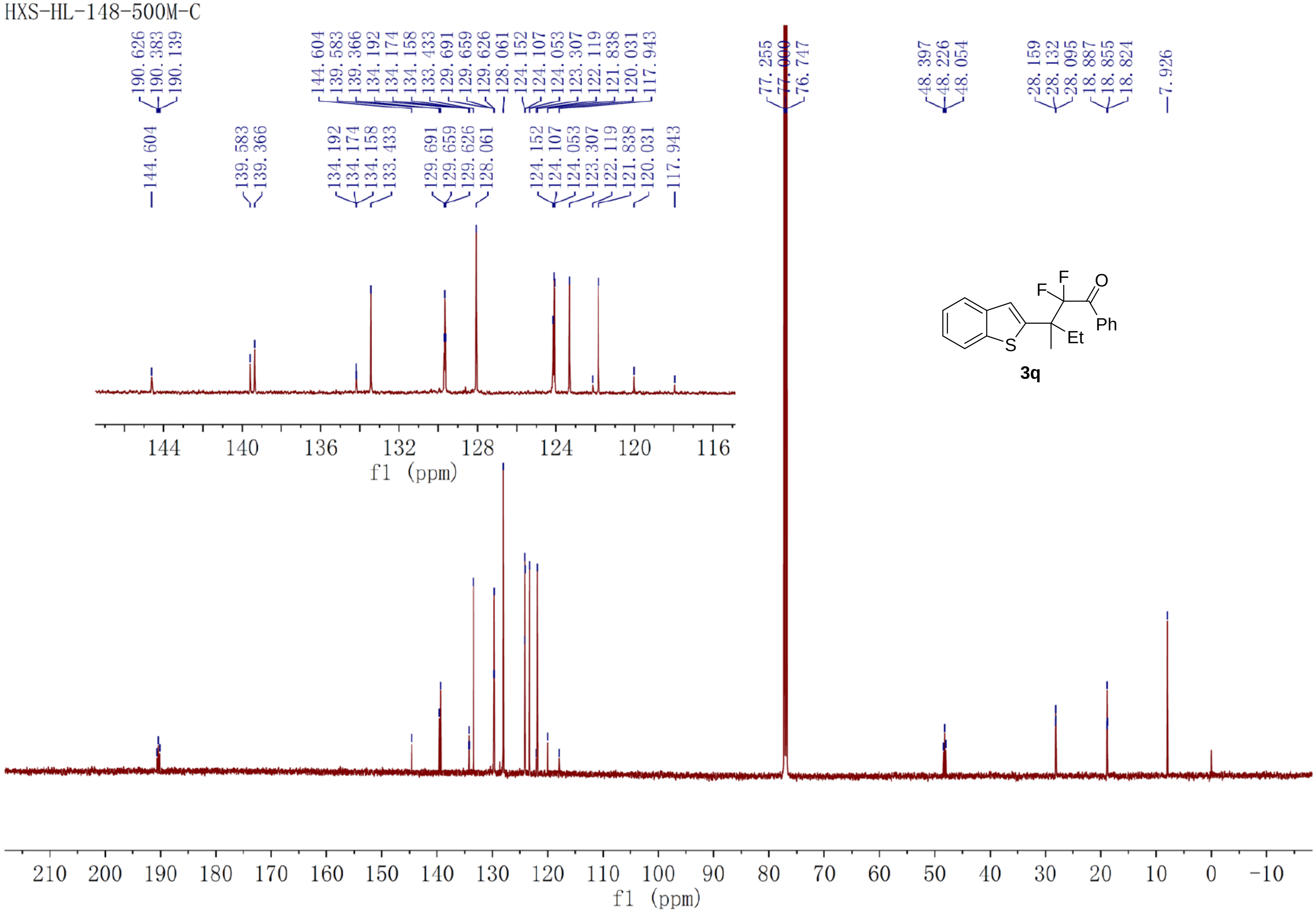

Supplementary Figure 72. ^{13}C NMR (125 MHz, $CDCl_3$) spectra for compound **3q**

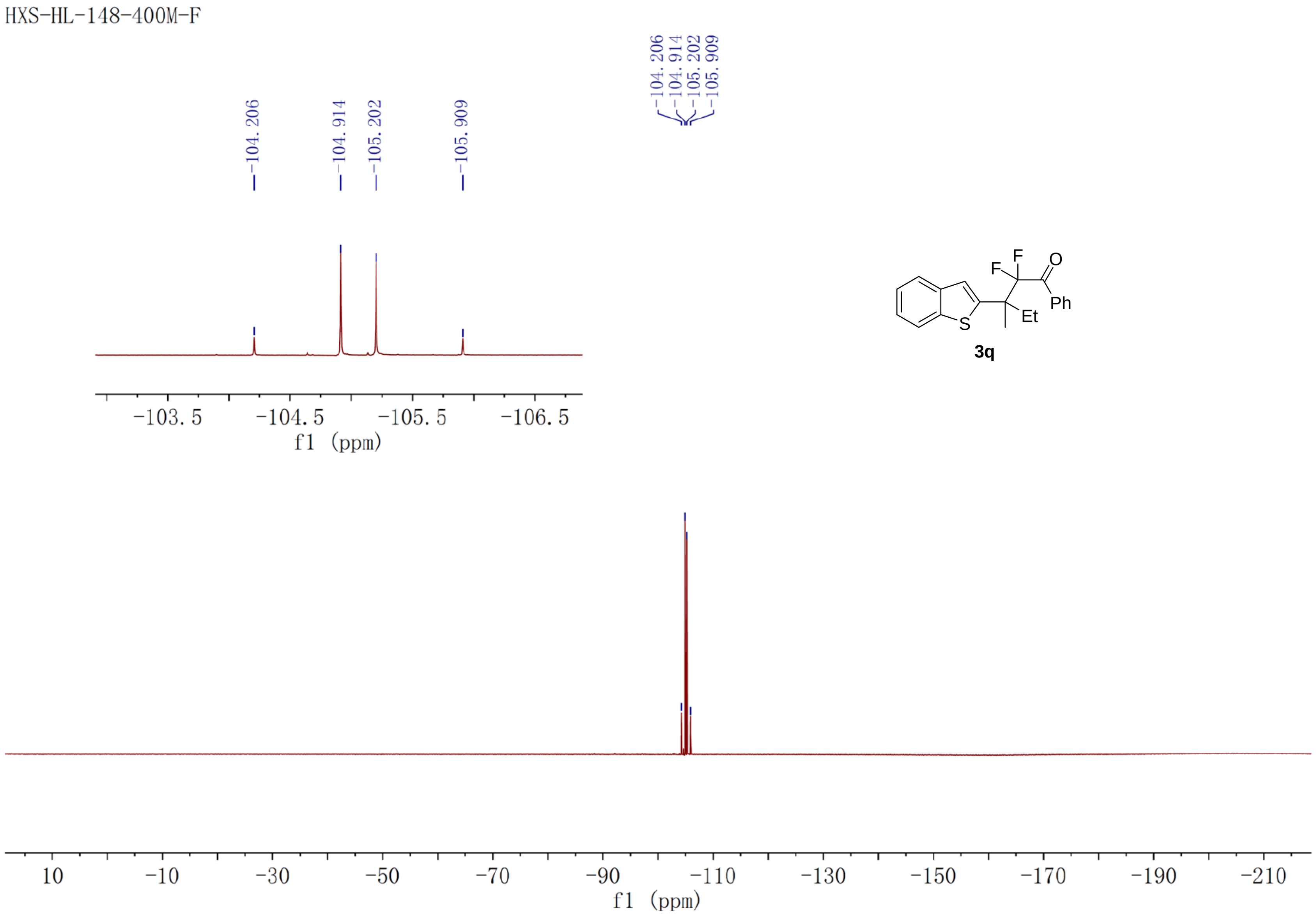

3q

Supplementary Figure 73. ^{19}F NMR (376 MHz, $CDCl_3$) spectra for compound **3q**

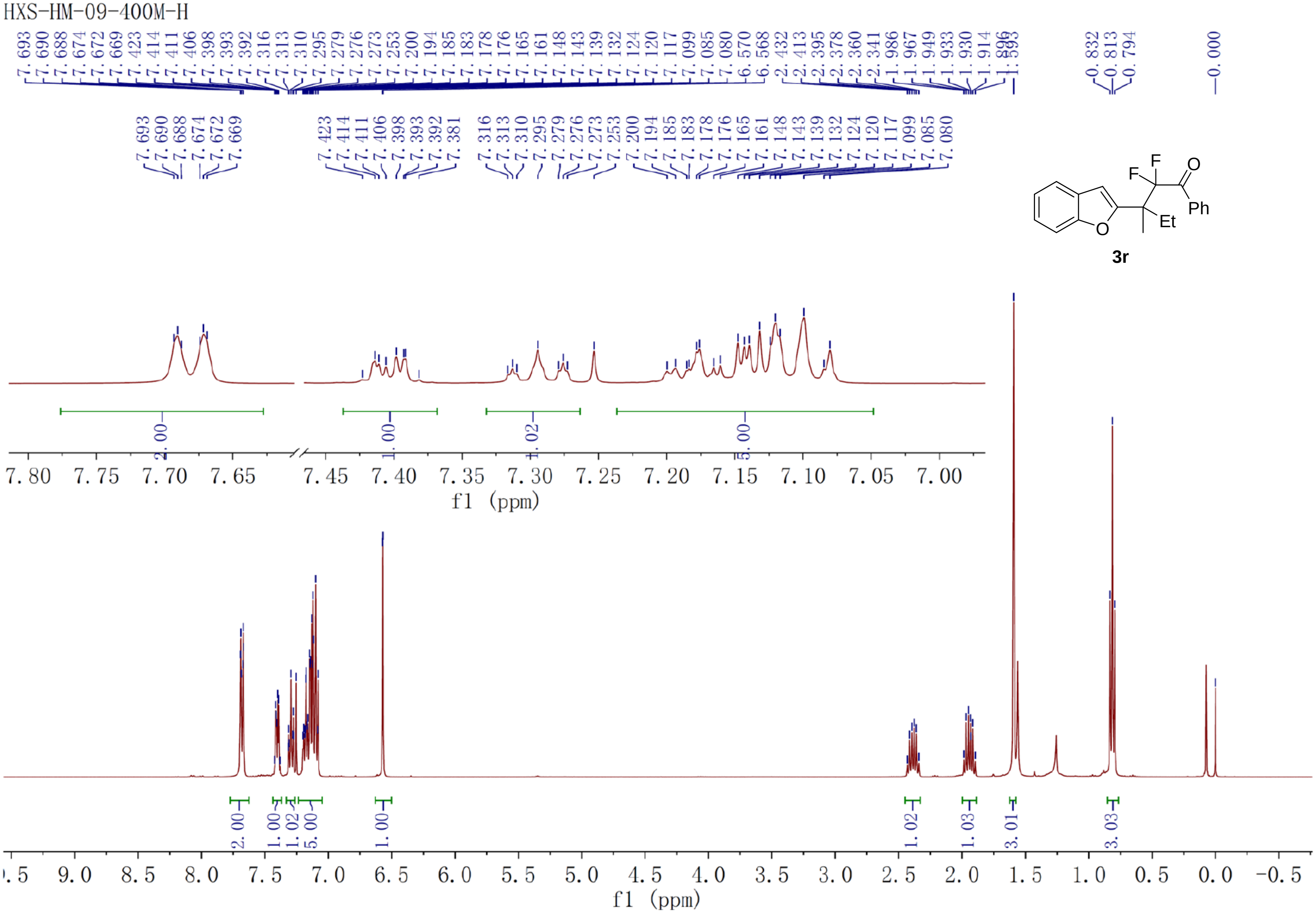

3r

Supplementary Figure 74. ^{1}H NMR (400 MHz, $CDCl_3$) spectra for compound **3r**

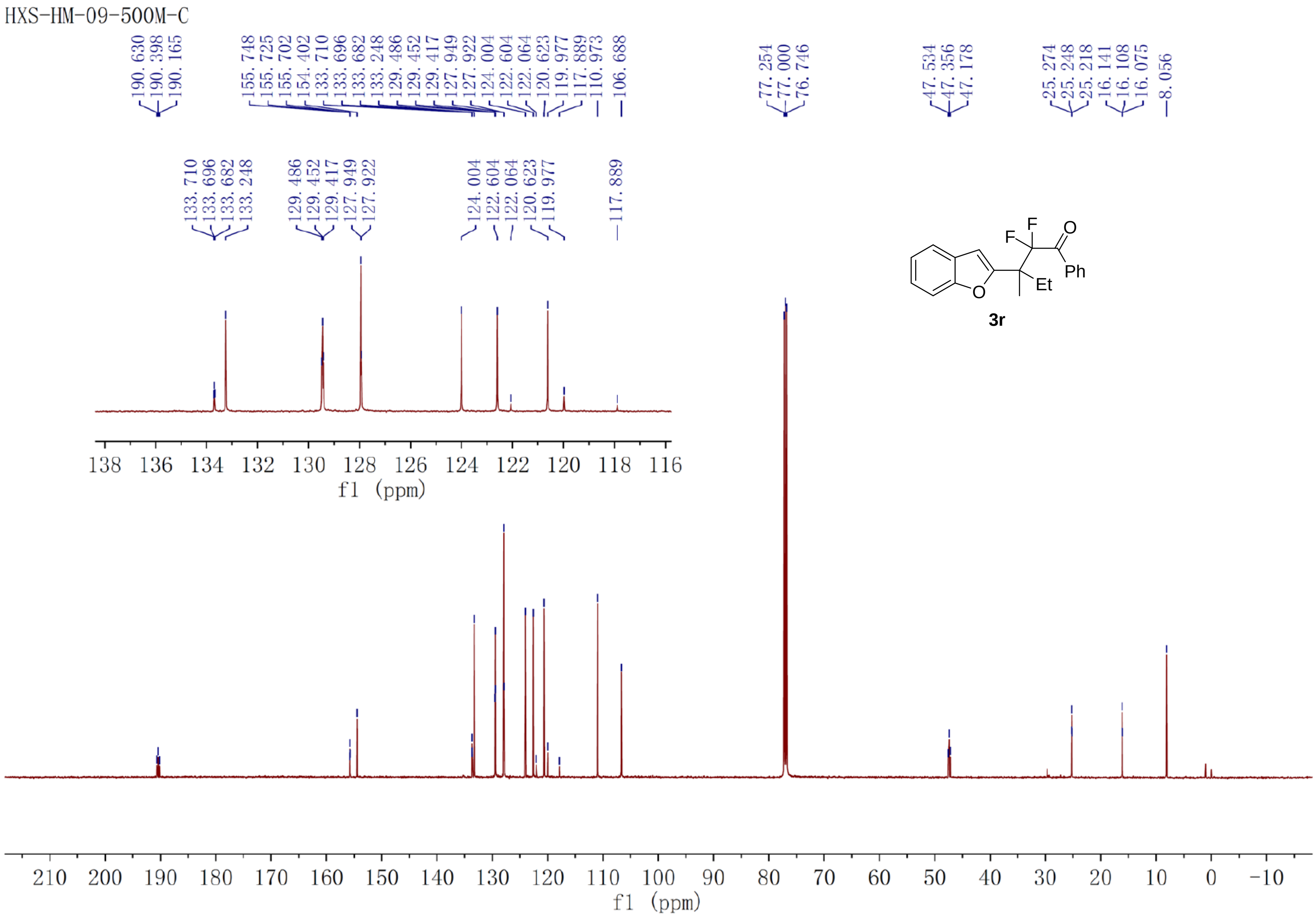

Supplementary Figure 75. ^{13}C NMR (125 MHz, $CDCl_3$) spectra for compound **3r**

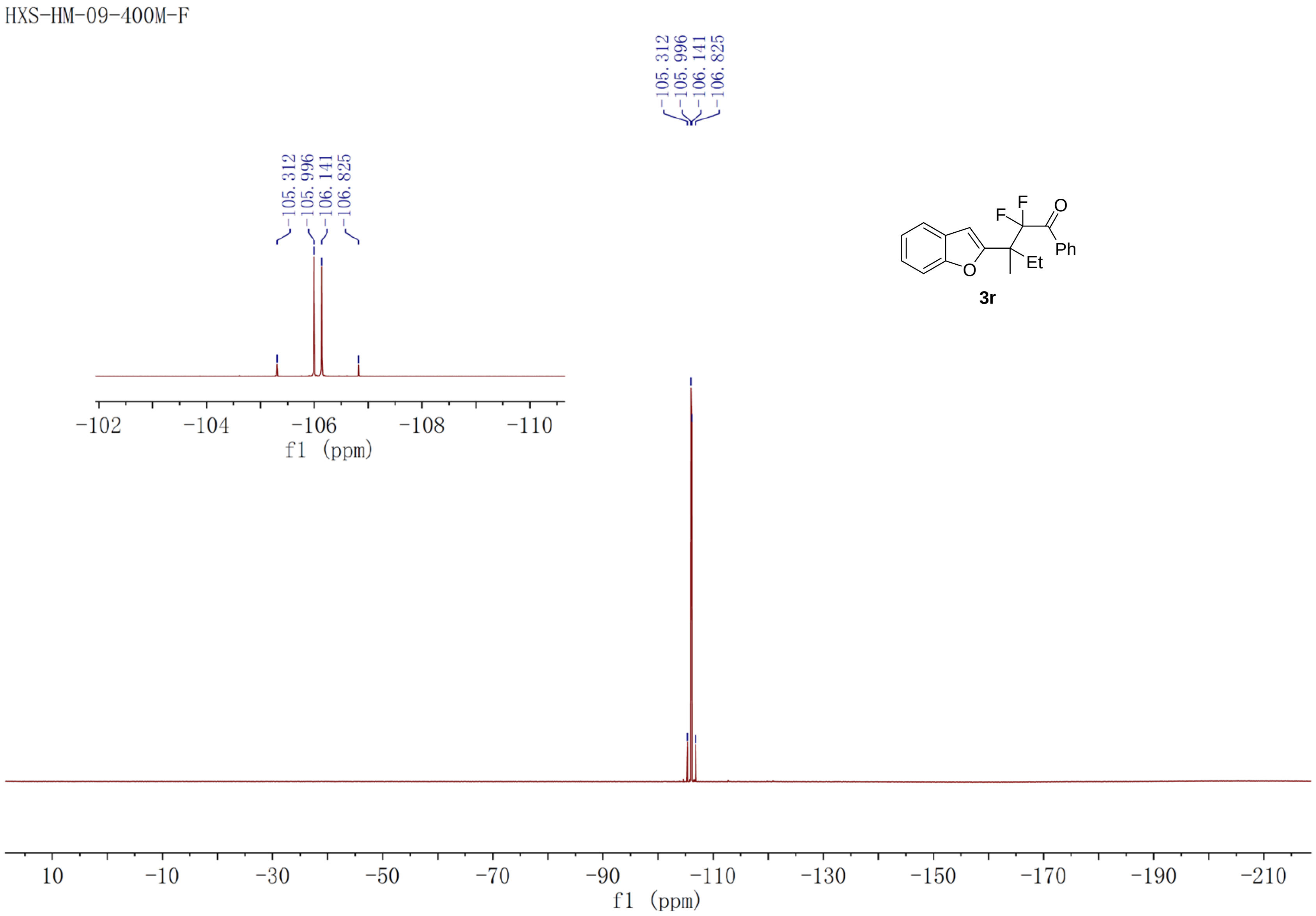

Supplementary Figure 76. ^{19}F NMR (376 MHz, $CDCl_3$) spectra for compound **3r**

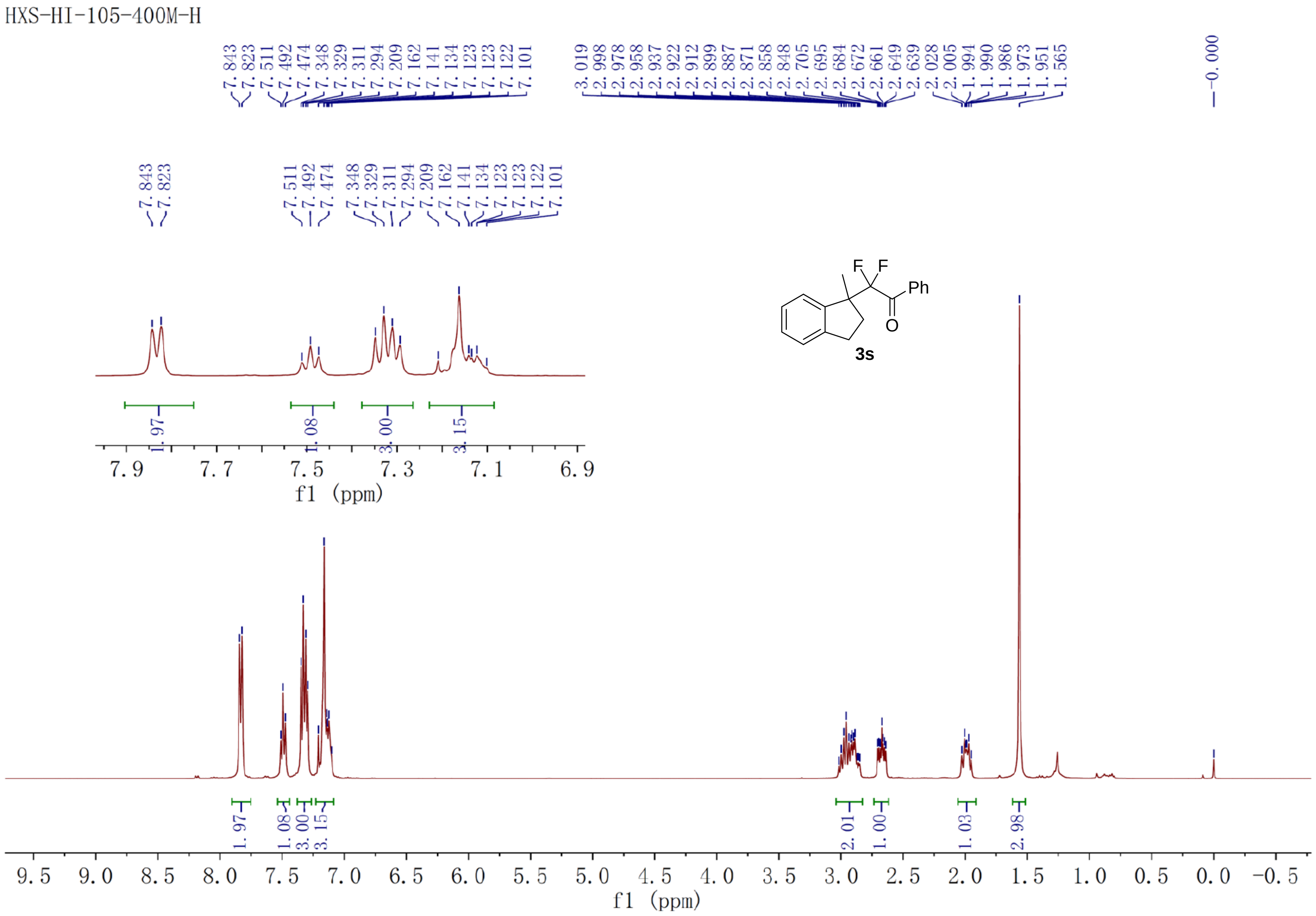

Supplementary Figure 77. ^{1}H NMR (400 MHz, $CDCl_3$) spectra for compound **3s**

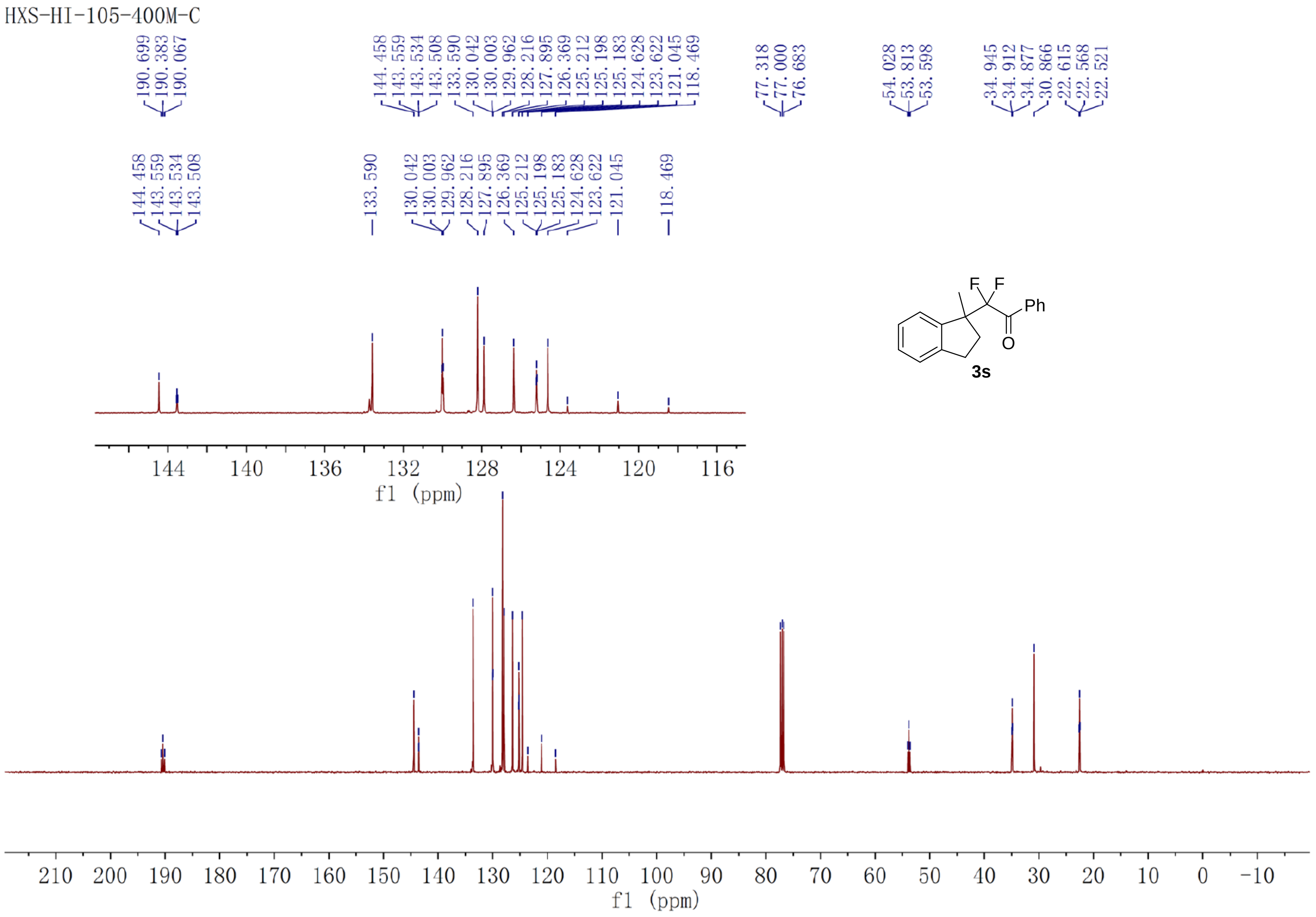

Supplementary Figure 78. ^{13}C NMR (100 MHz, $CDCl_3$) spectra for compound **3s**

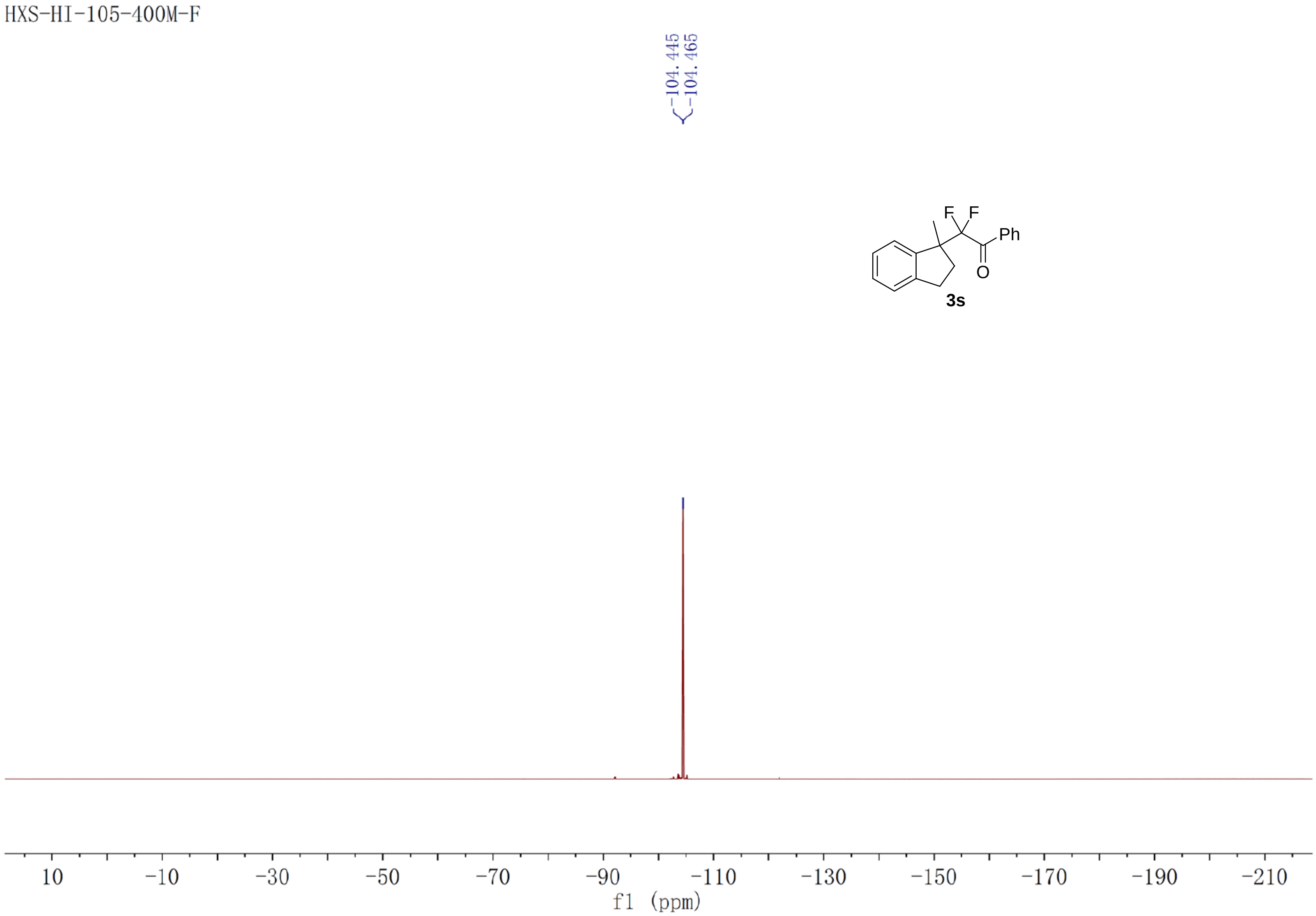

Supplementary Figure 79. ^{19}F NMR (376 MHz, $CDCl_3$) spectra for compound **3s**

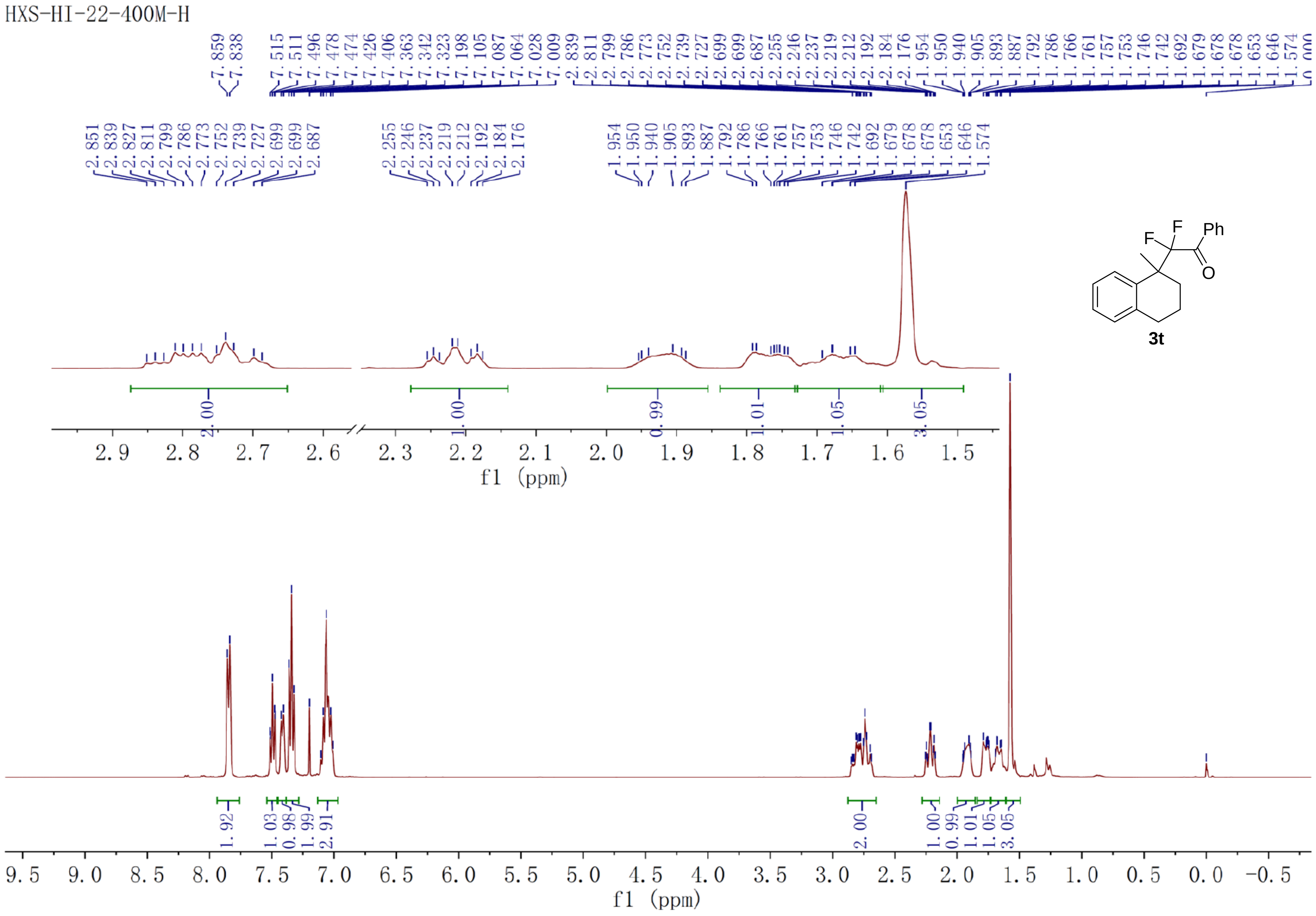

Supplementary Figure 80. ^{1}H NMR (400 MHz, $CDCl_3$) spectra for compound **3t**

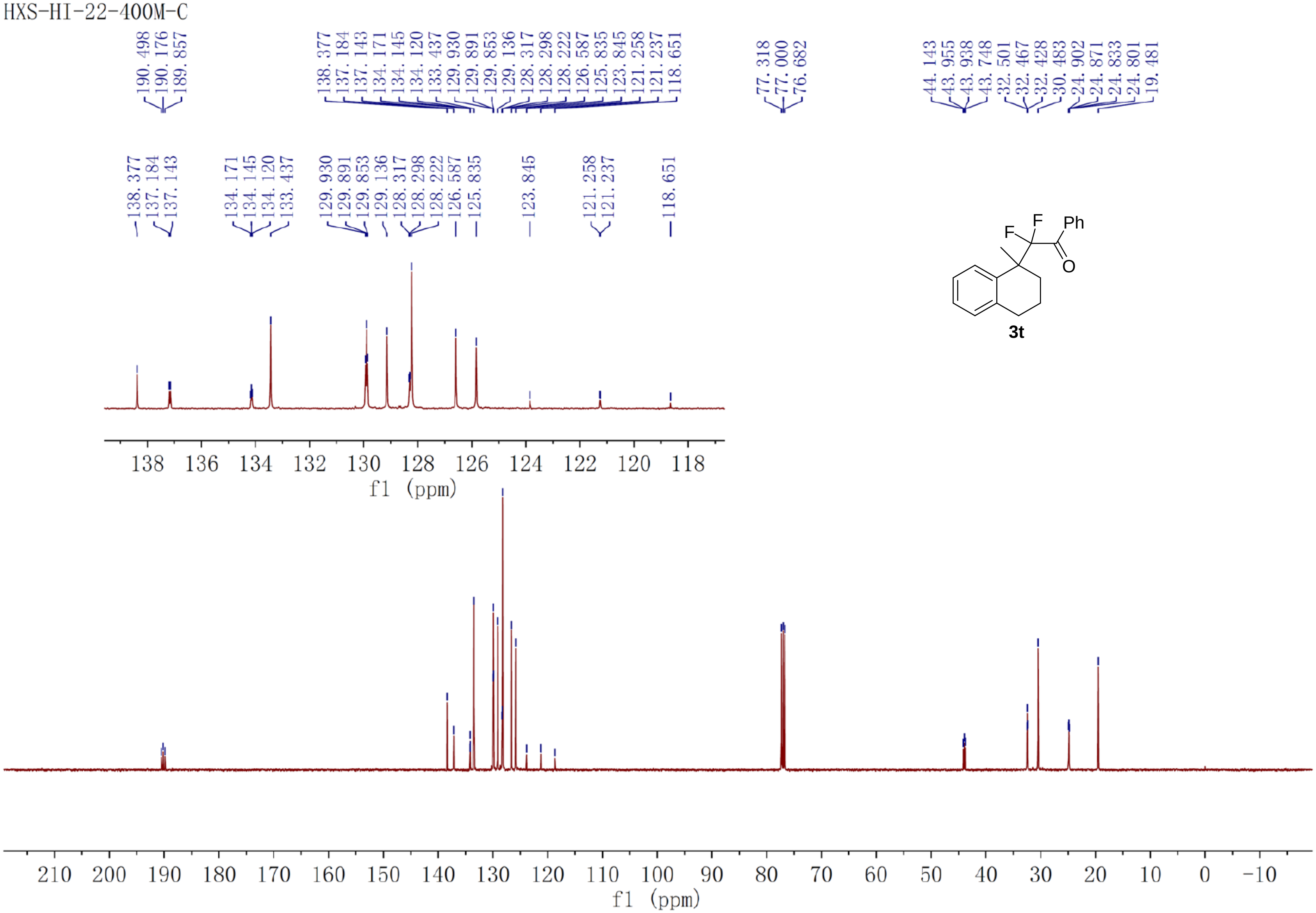

Supplementary Figure 81. ^{13}C NMR (100 MHz, $CDCl_3$) spectra for compound **3t**

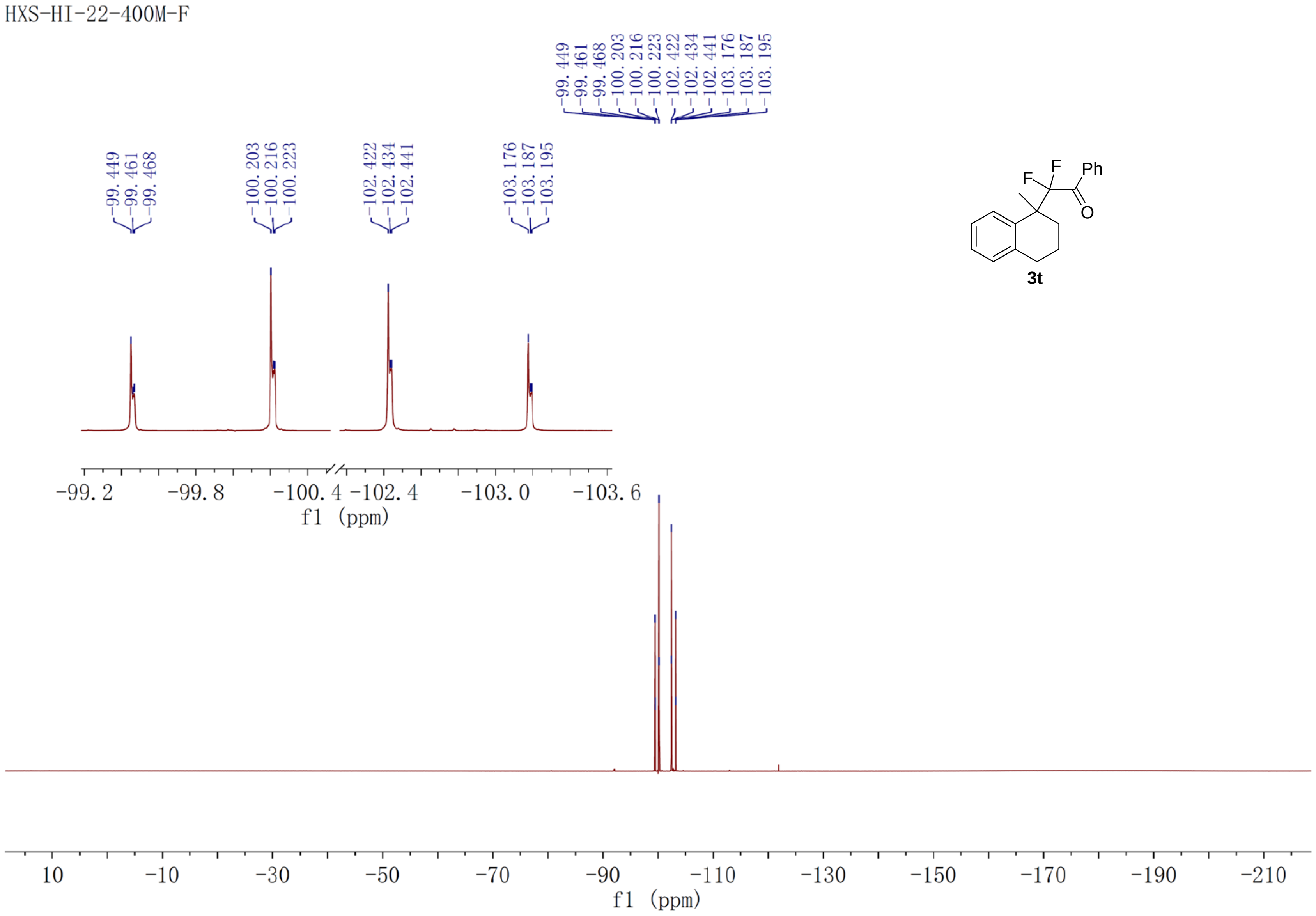

Supplementary Figure 82. ^{19}F NMR (376 MHz, $CDCl_3$) spectra for compound **3t**

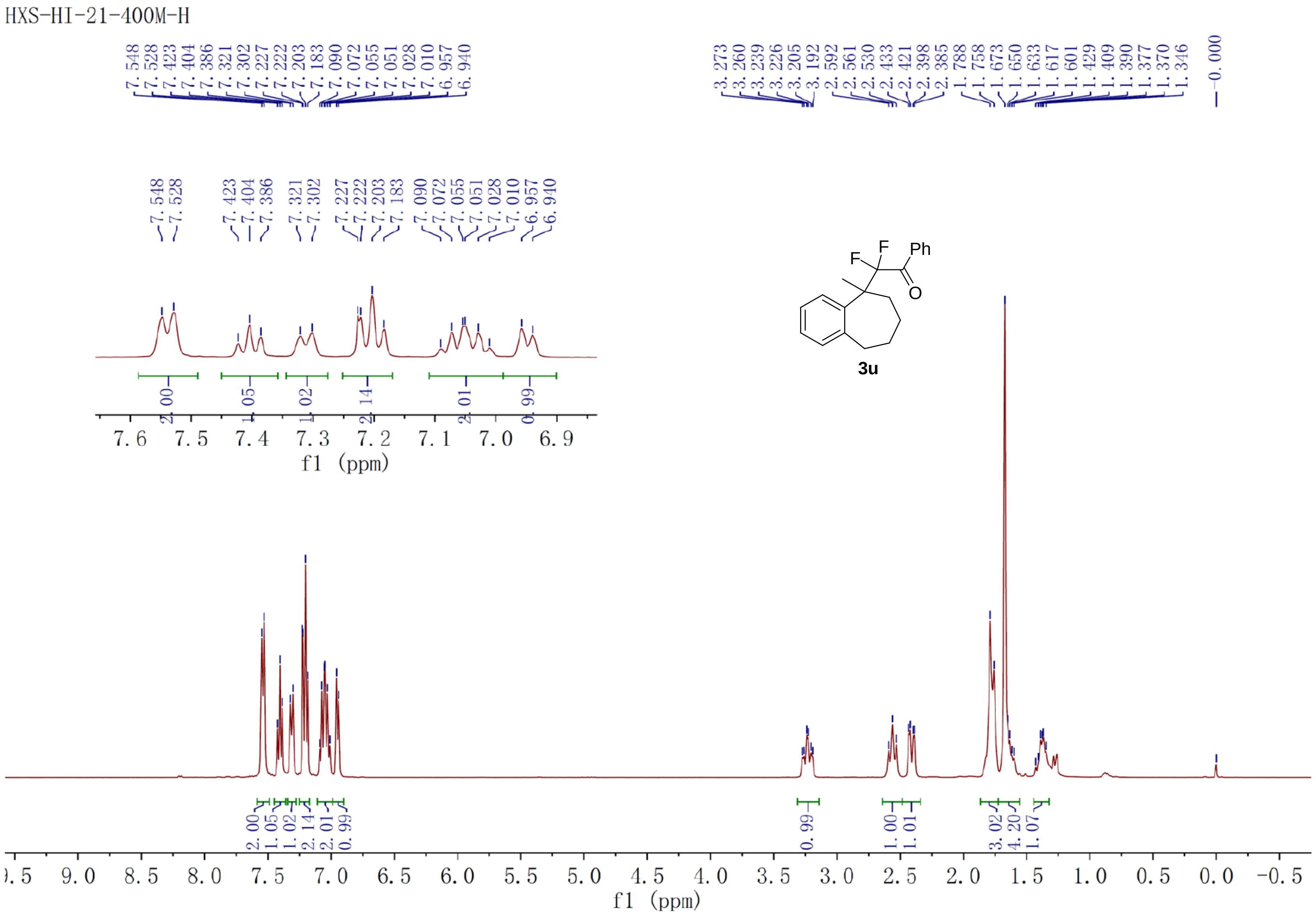

Supplementary Figure 83. ^{1}H NMR (400 MHz, $CDCl_3$) spectra for compound **3u**

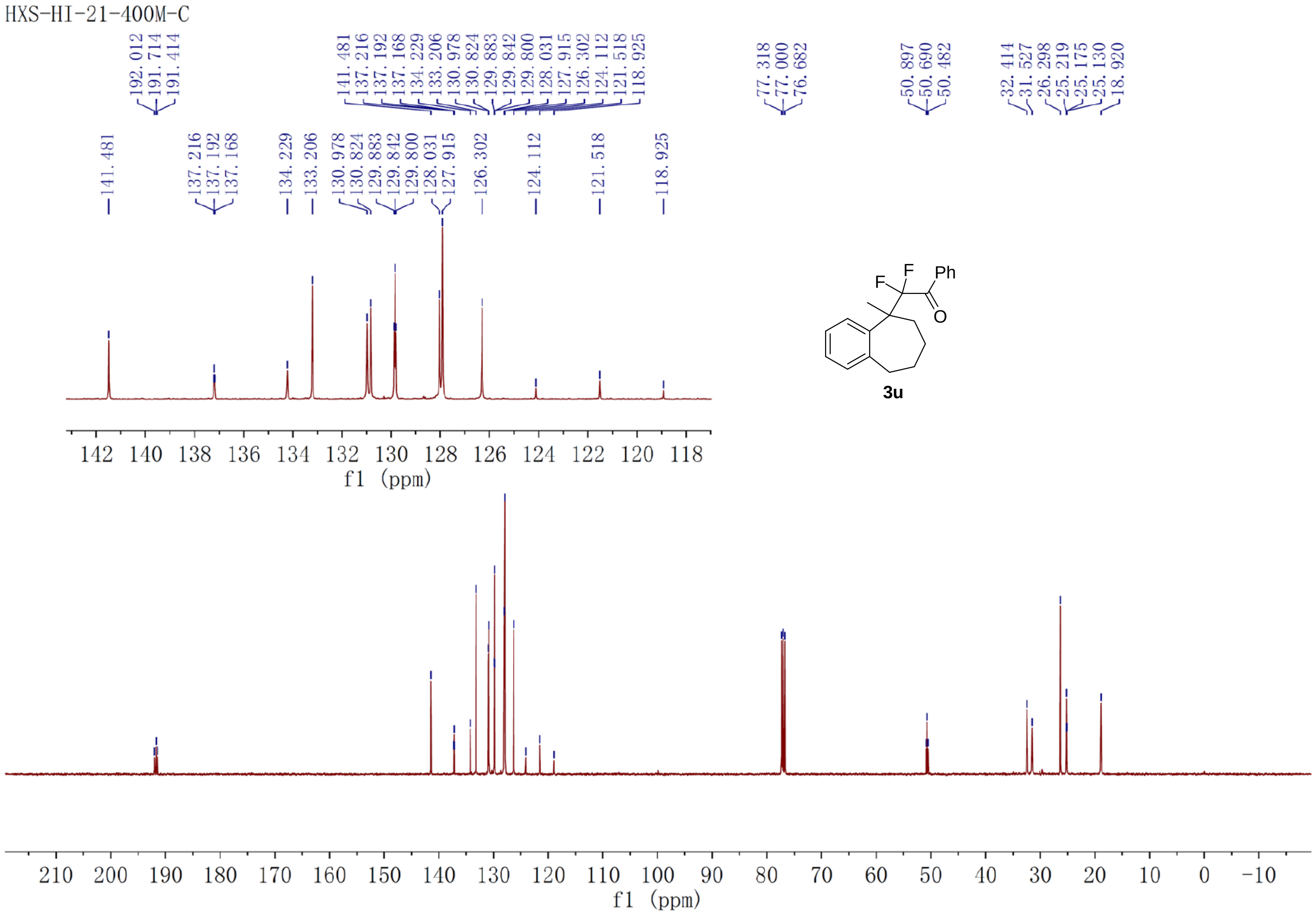

Supplementary Figure 84. ^{13}C NMR (100 MHz, $CDCl_3$) spectra for compound **3u**

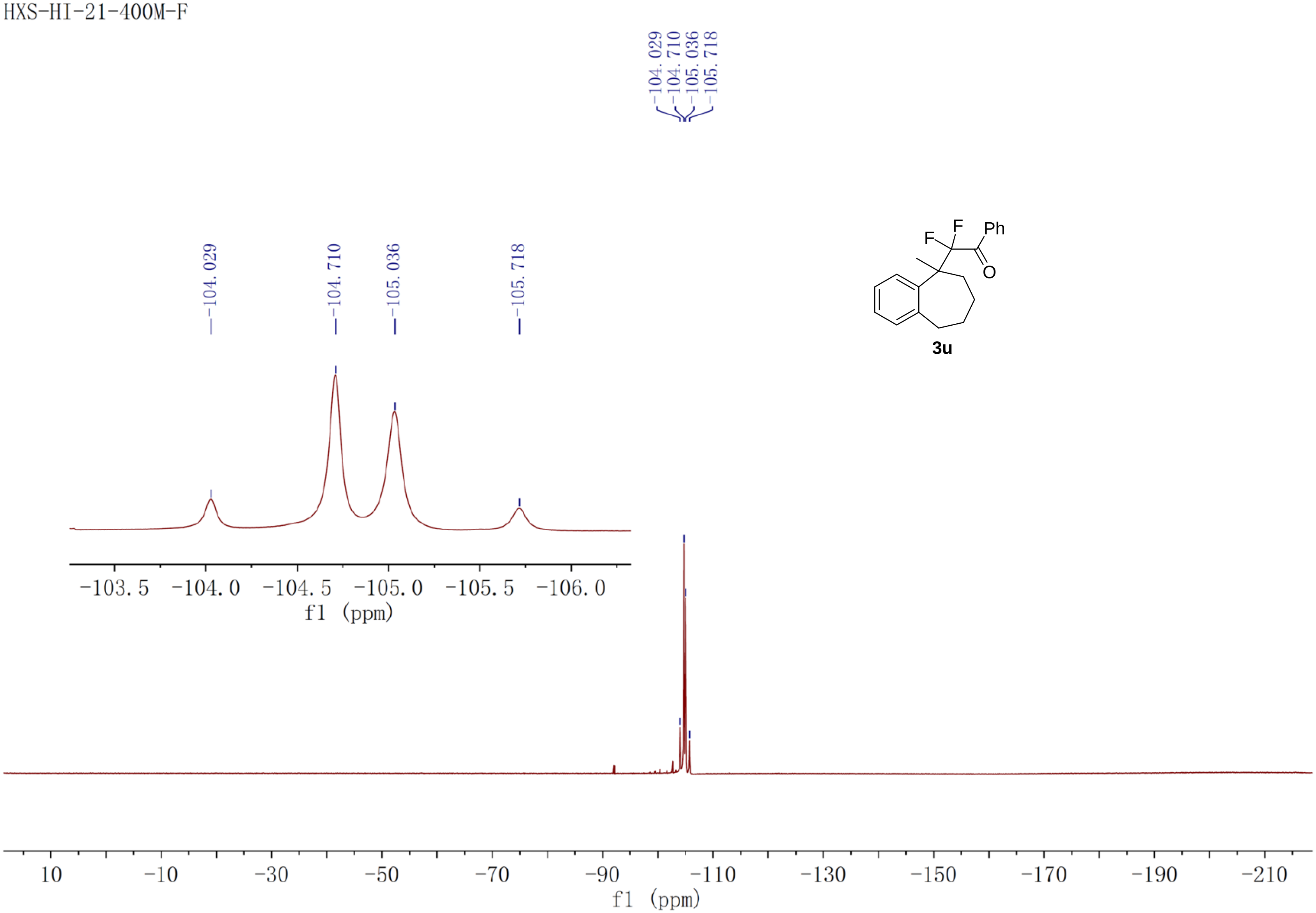

3u

Supplementary Figure 85. ^{19}F NMR (376 MHz, $CDCl_3$) spectra for compound **3u**

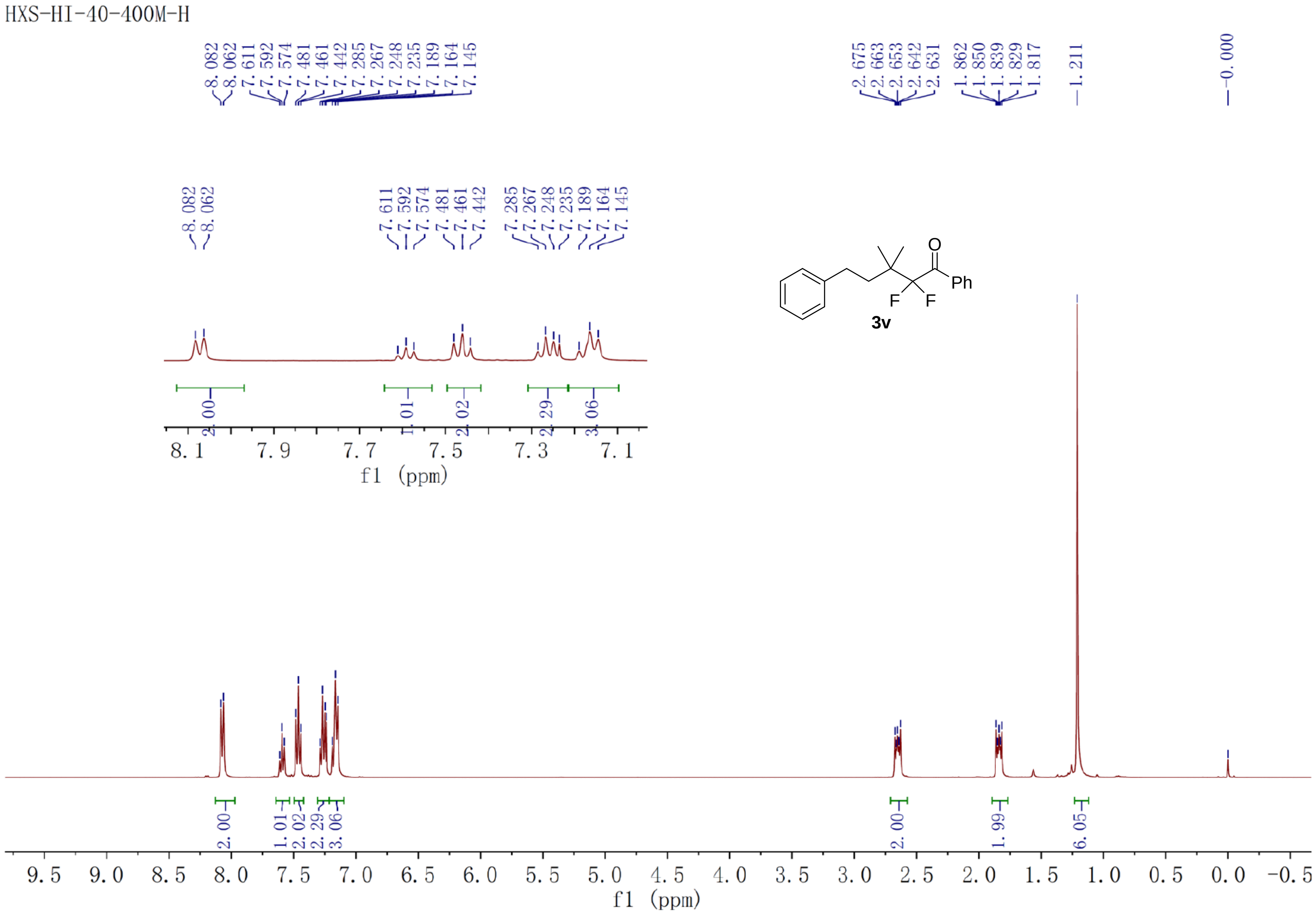

Supplementary Figure 86. ^{1}H NMR (400 MHz, $CDCl_3$) spectra for compound **3v**

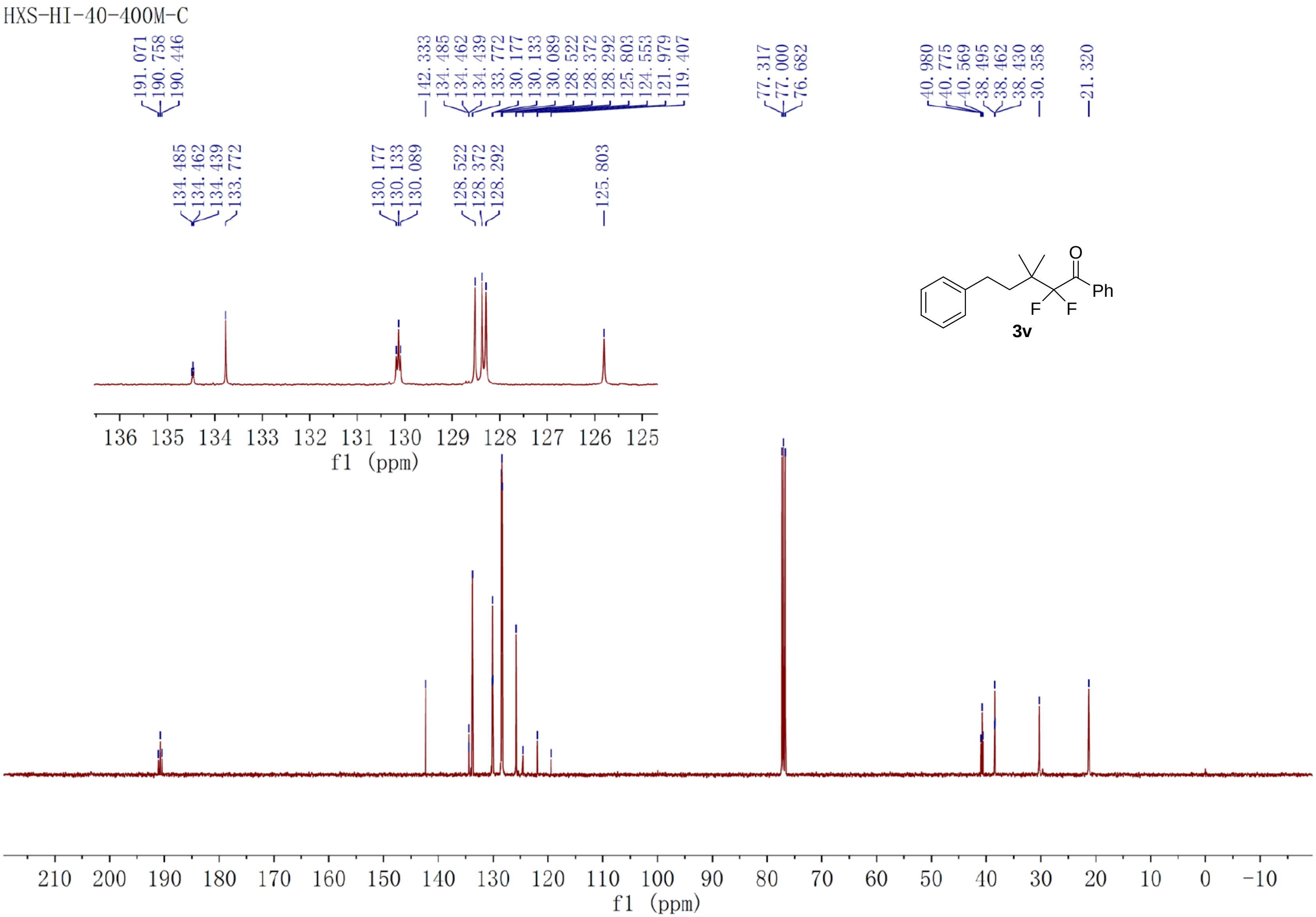

Supplementary Figure 87. ^{13}C NMR (100 MHz, $CDCl_3$) spectra for compound **3v**

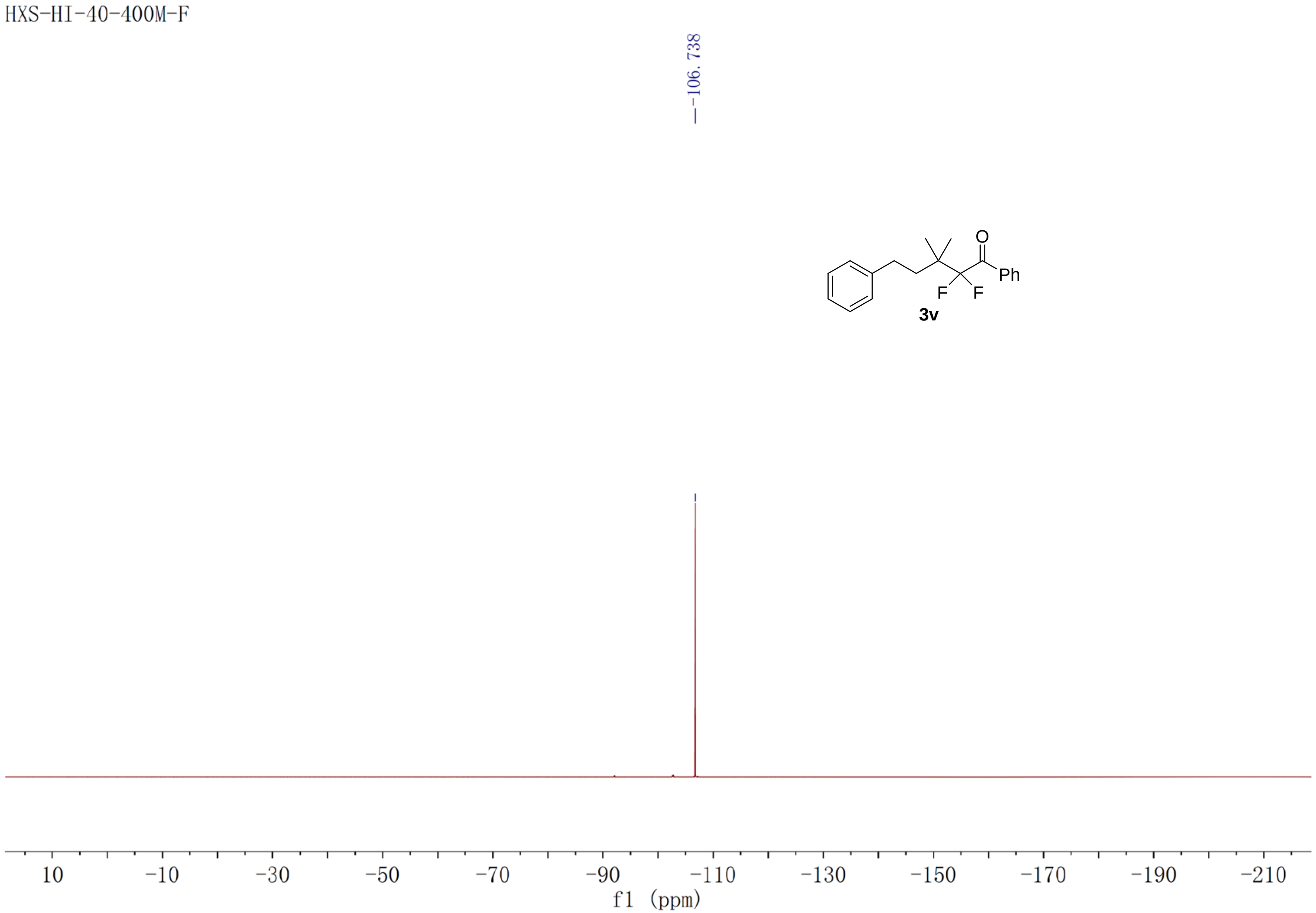

Supplementary Figure 88. ^{19}F NMR (376 MHz, $CDCl_3$) spectra for compound **3v**

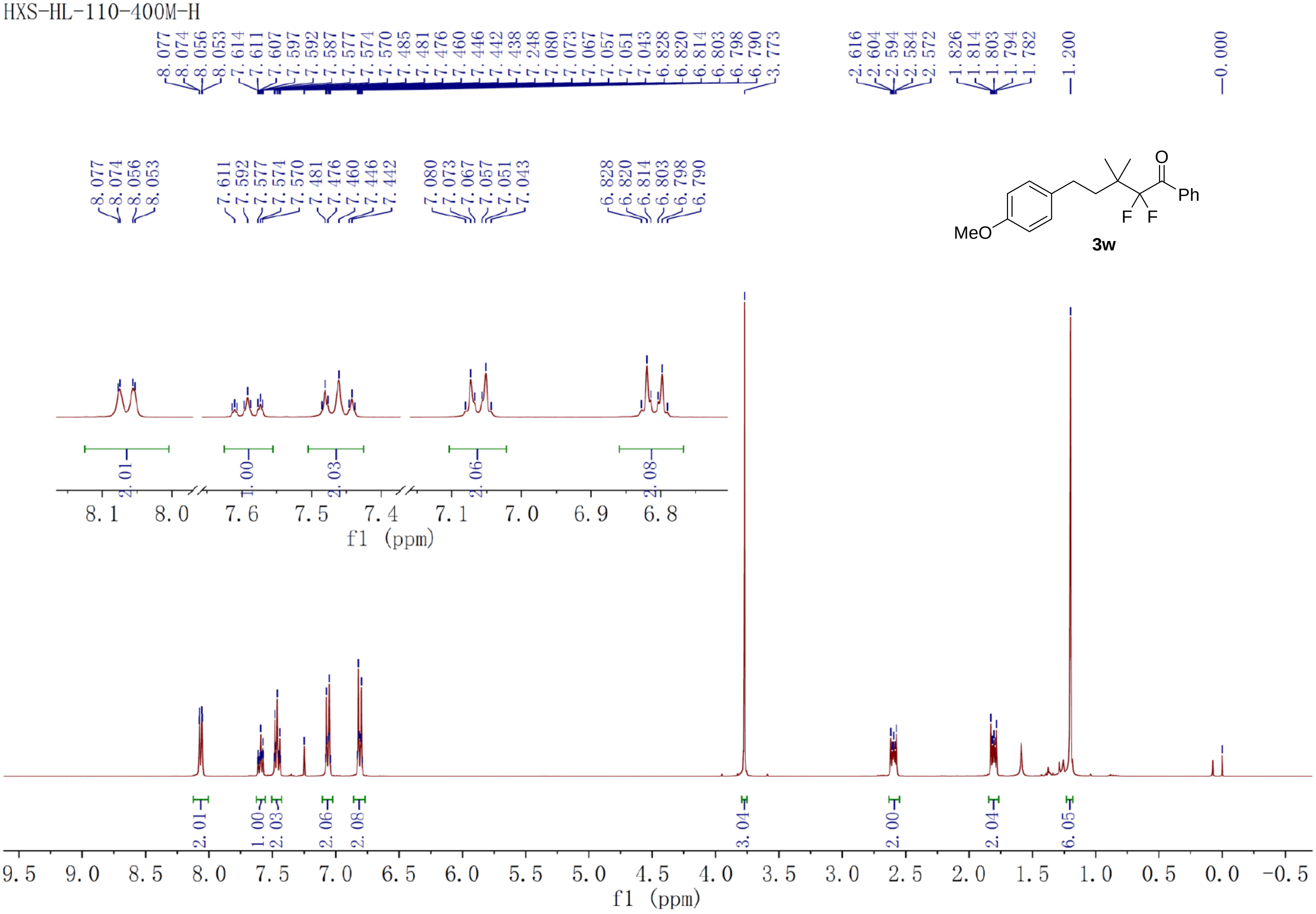

Supplementary Figure 89. ^{1}H NMR (400 MHz, $CDCl_3$) spectra for compound **3w**

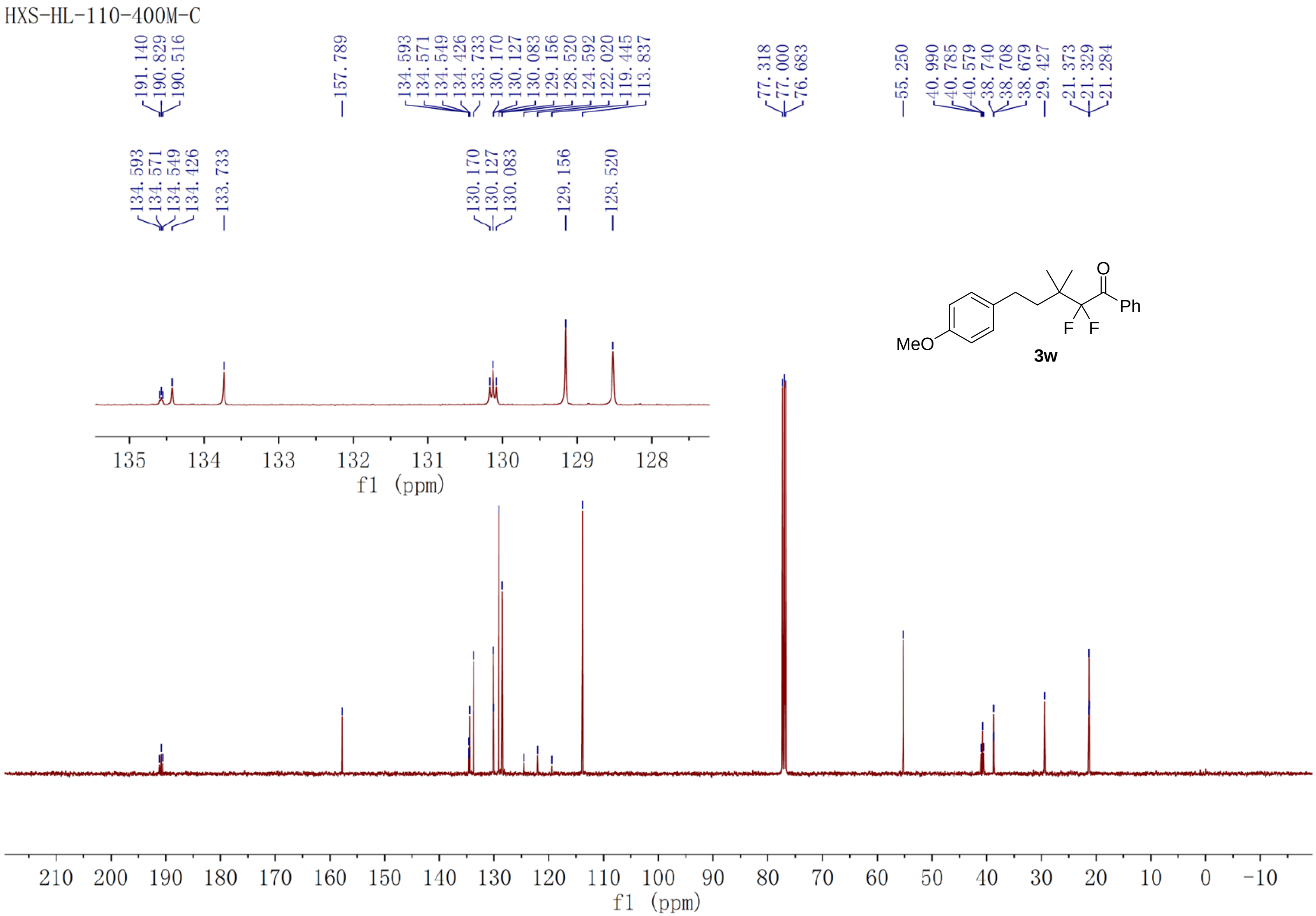

Supplementary Figure 90. ^{13}C NMR (100 MHz, $CDCl_3$) spectra for compound **3w**

HXS-HL-110-400M-F

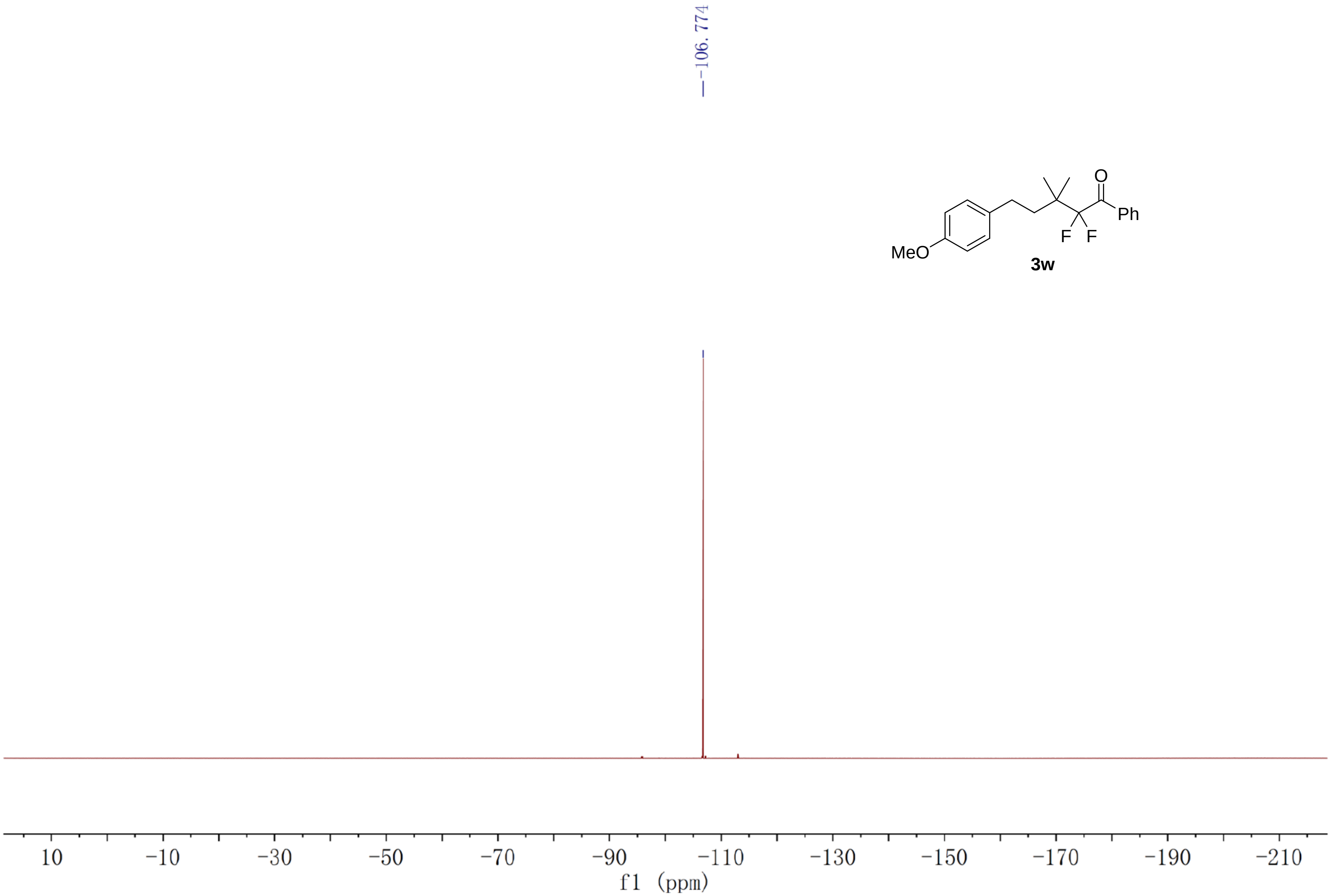

Supplementary Figure 91. ^{19}F NMR (376 MHz, $CDCl_3$) spectra for compound **3w**

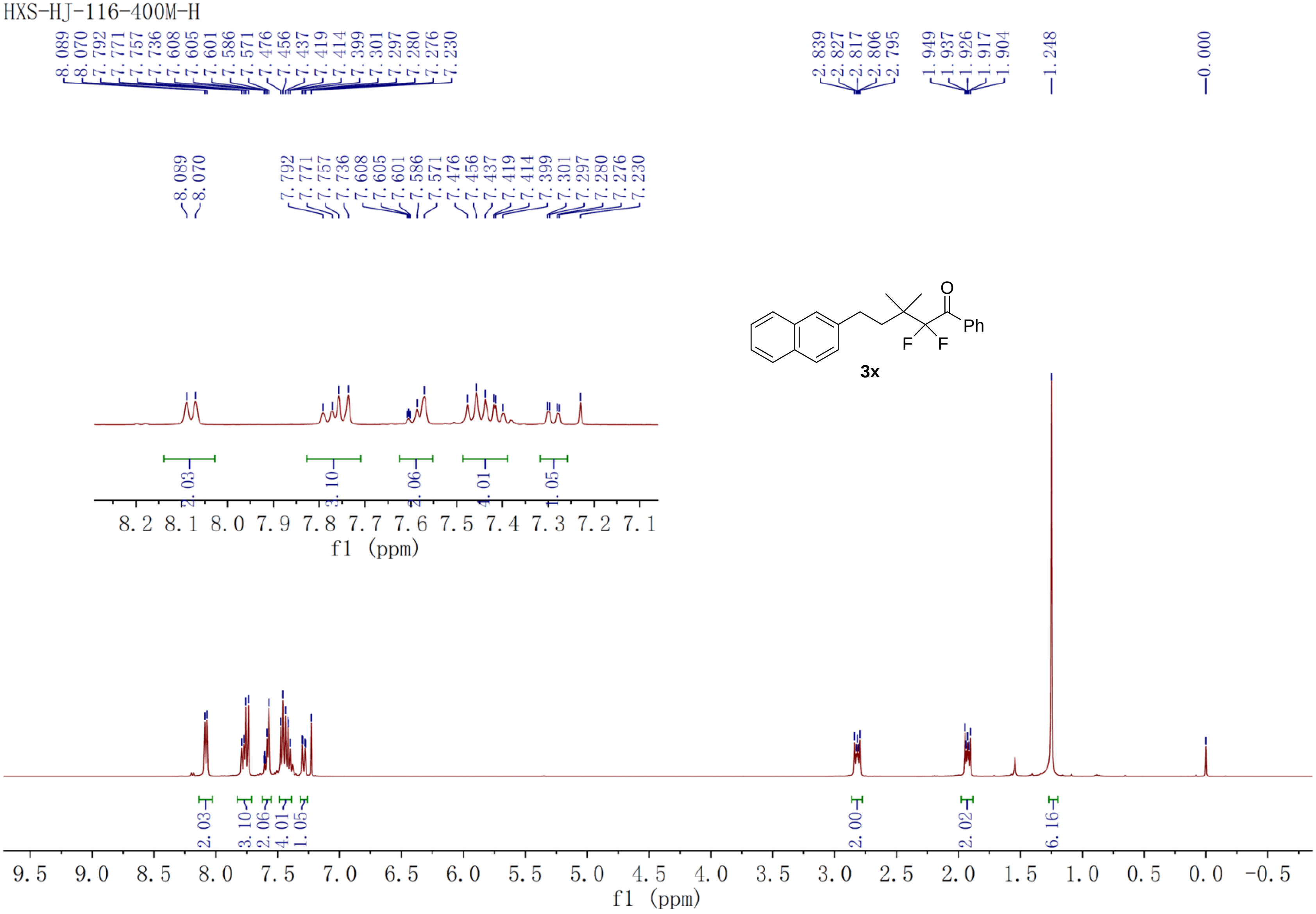

Supplementary Figure 92. ^{1}H NMR (400 MHz, $CDCl_3$) spectra for compound **3x**

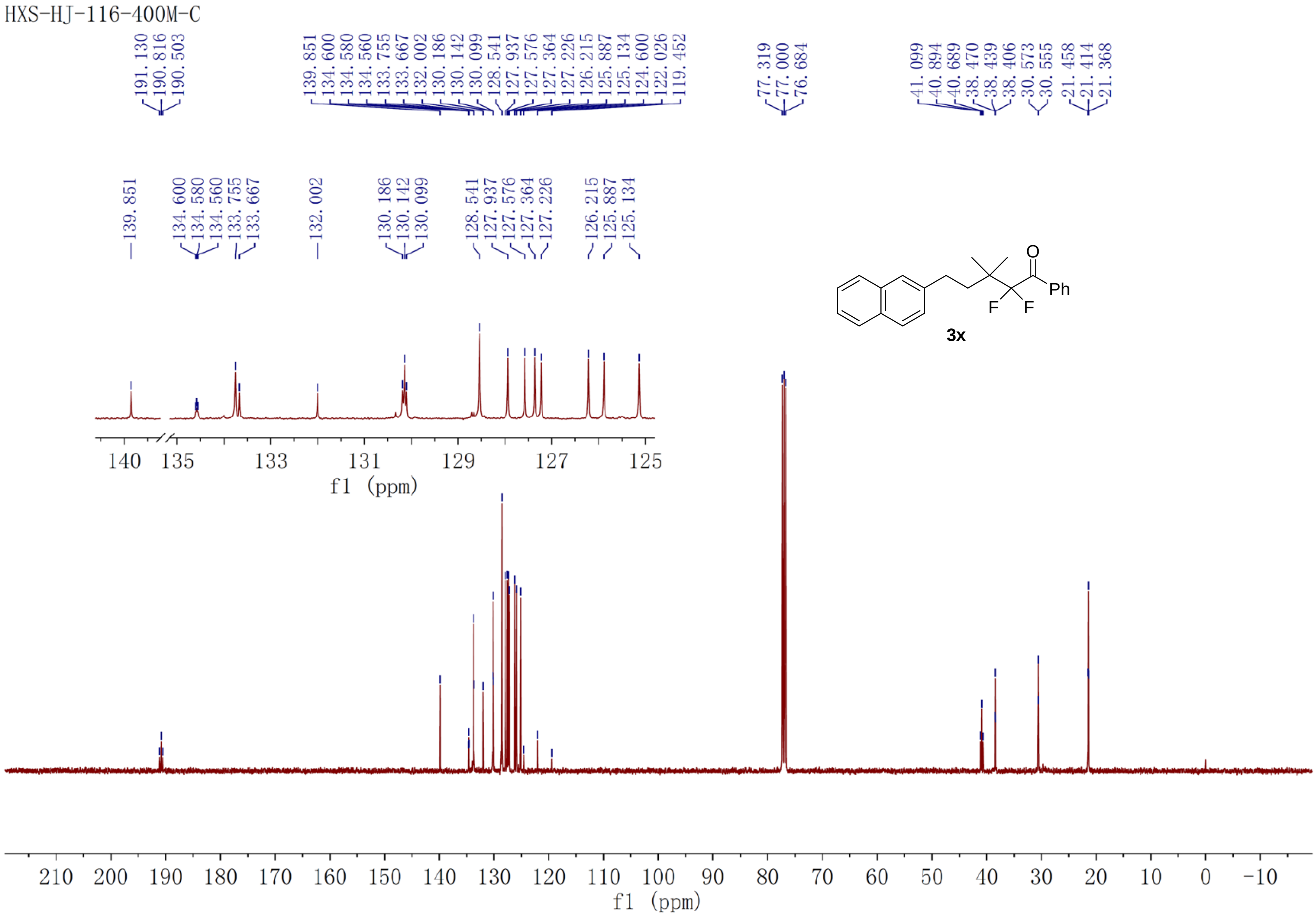

Supplementary Figure 93. ^{13}C NMR (100 MHz, $CDCl_3$) spectra for compound **3x**

HXS-HJ-116-400M-F

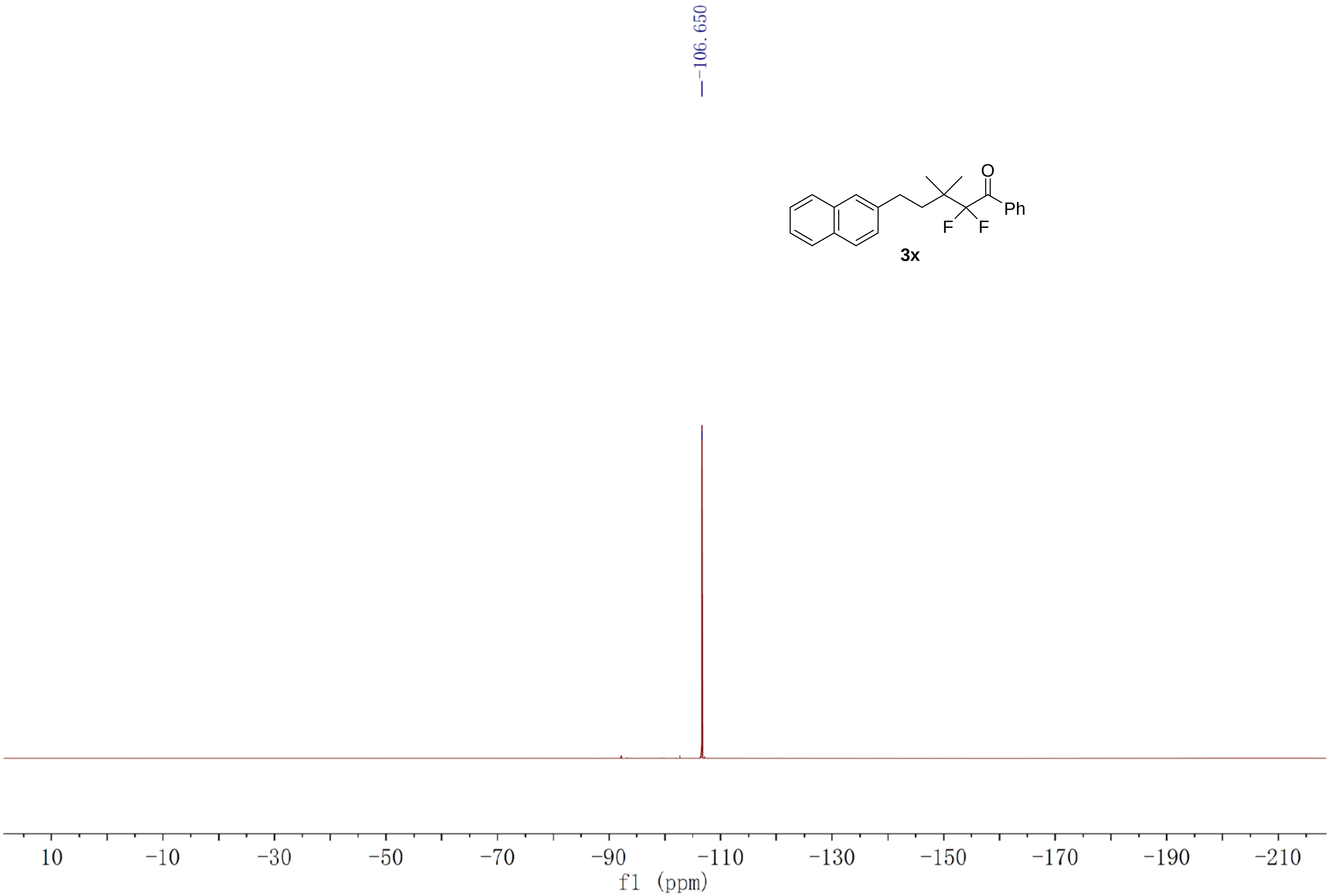

3x

Supplementary Figure 94. ^{19}F NMR (376 MHz, $CDCl_3$) spectra for compound **3x**

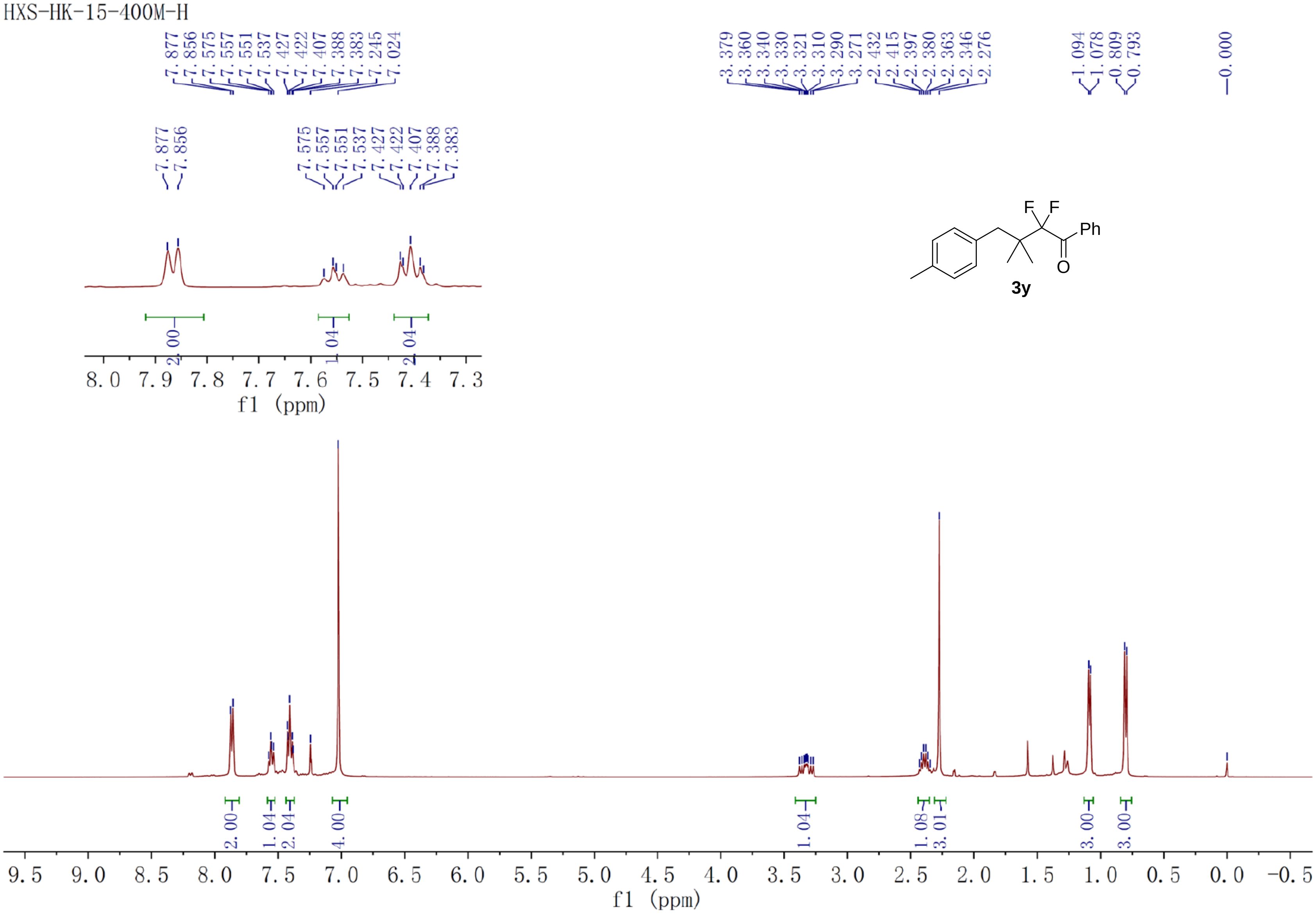

Supplementary Figure 95. ^{1}H NMR (400 MHz, $CDCl_3$) spectra for compound **3y**

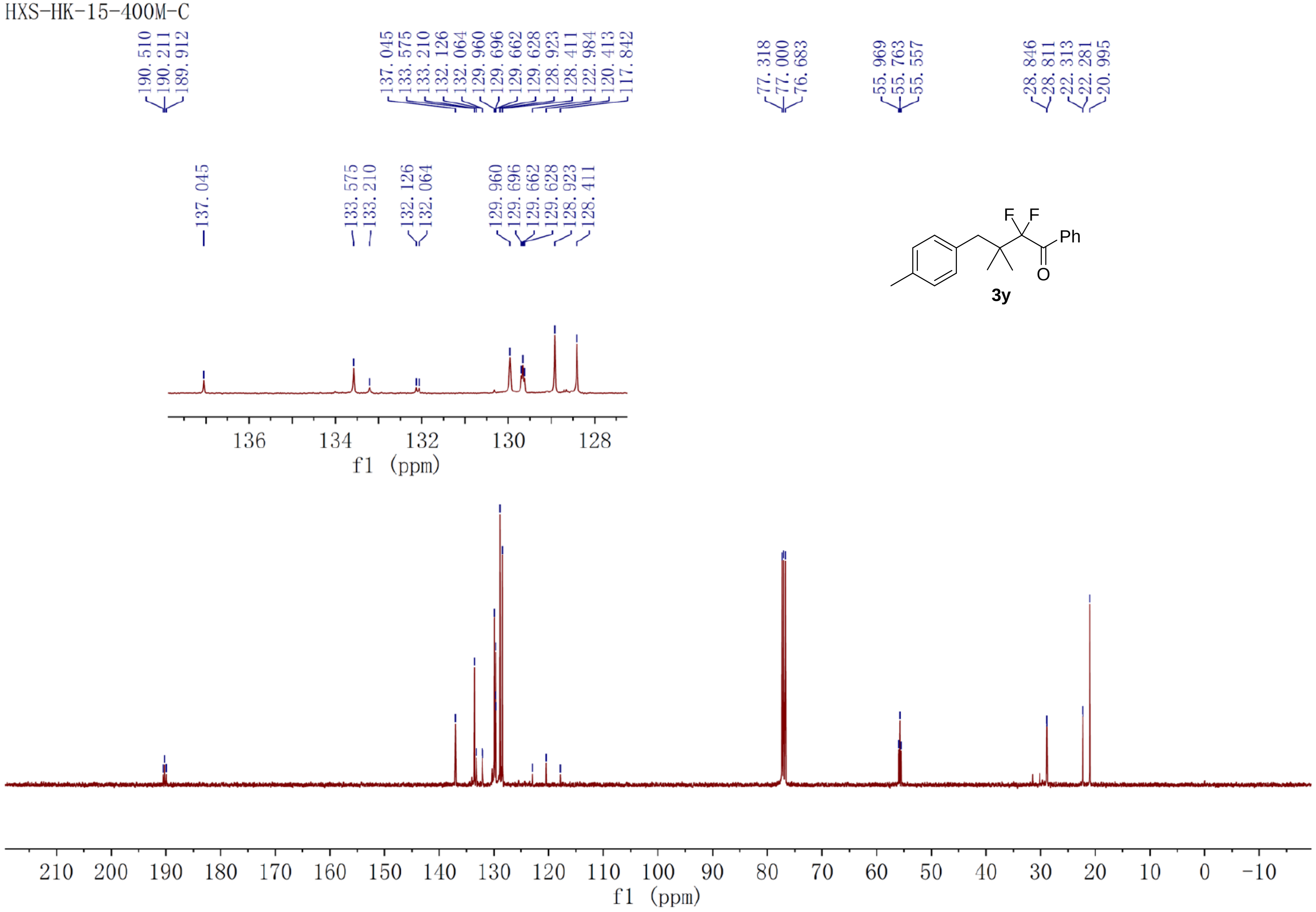

Supplementary Figure 96. ^{13}C NMR (100 MHz, $CDCl_3$) spectra for compound **3y**

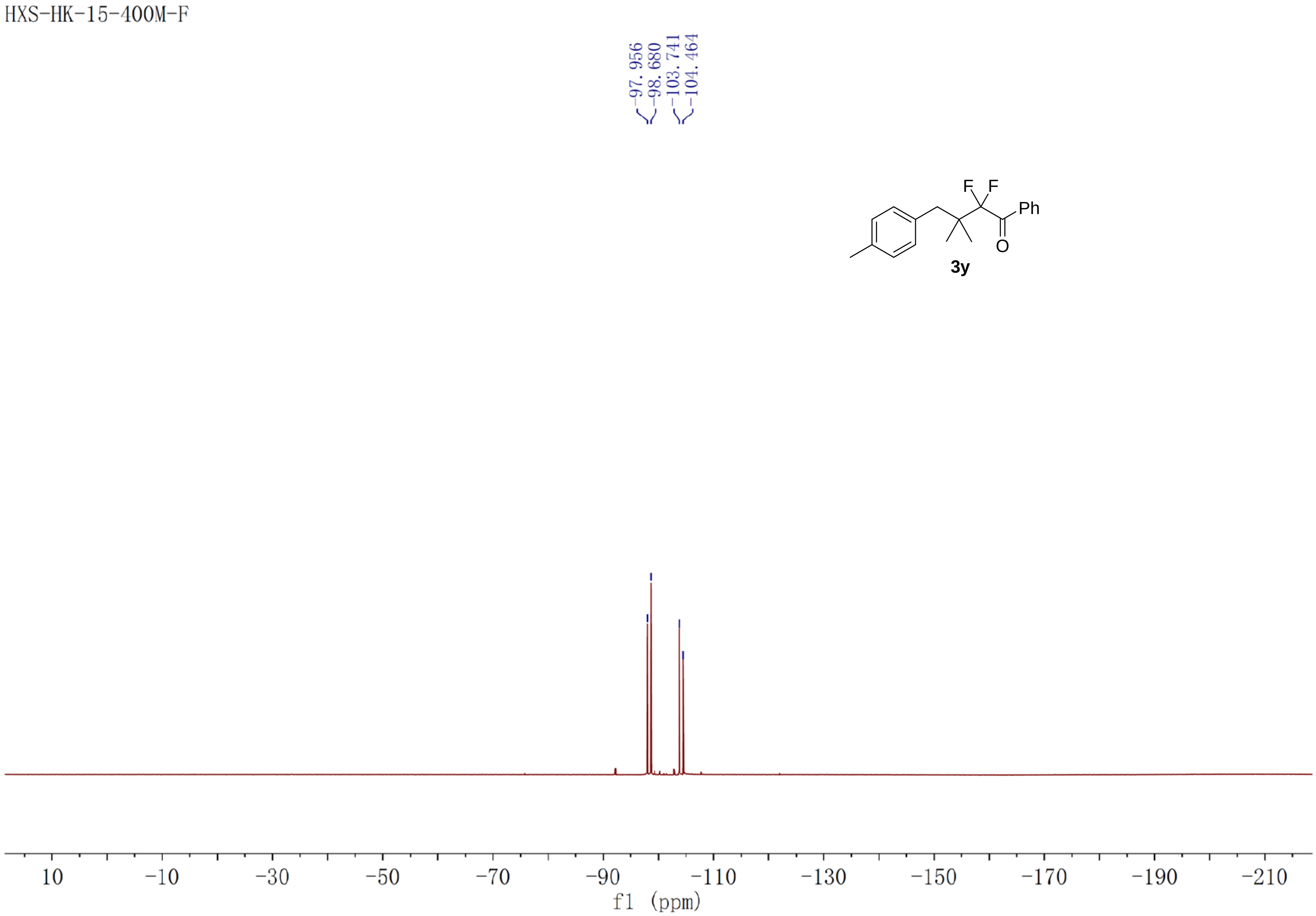

Supplementary Figure 97. ^{19}F NMR (376 MHz, $CDCl_3$) spectra for compound **3y**

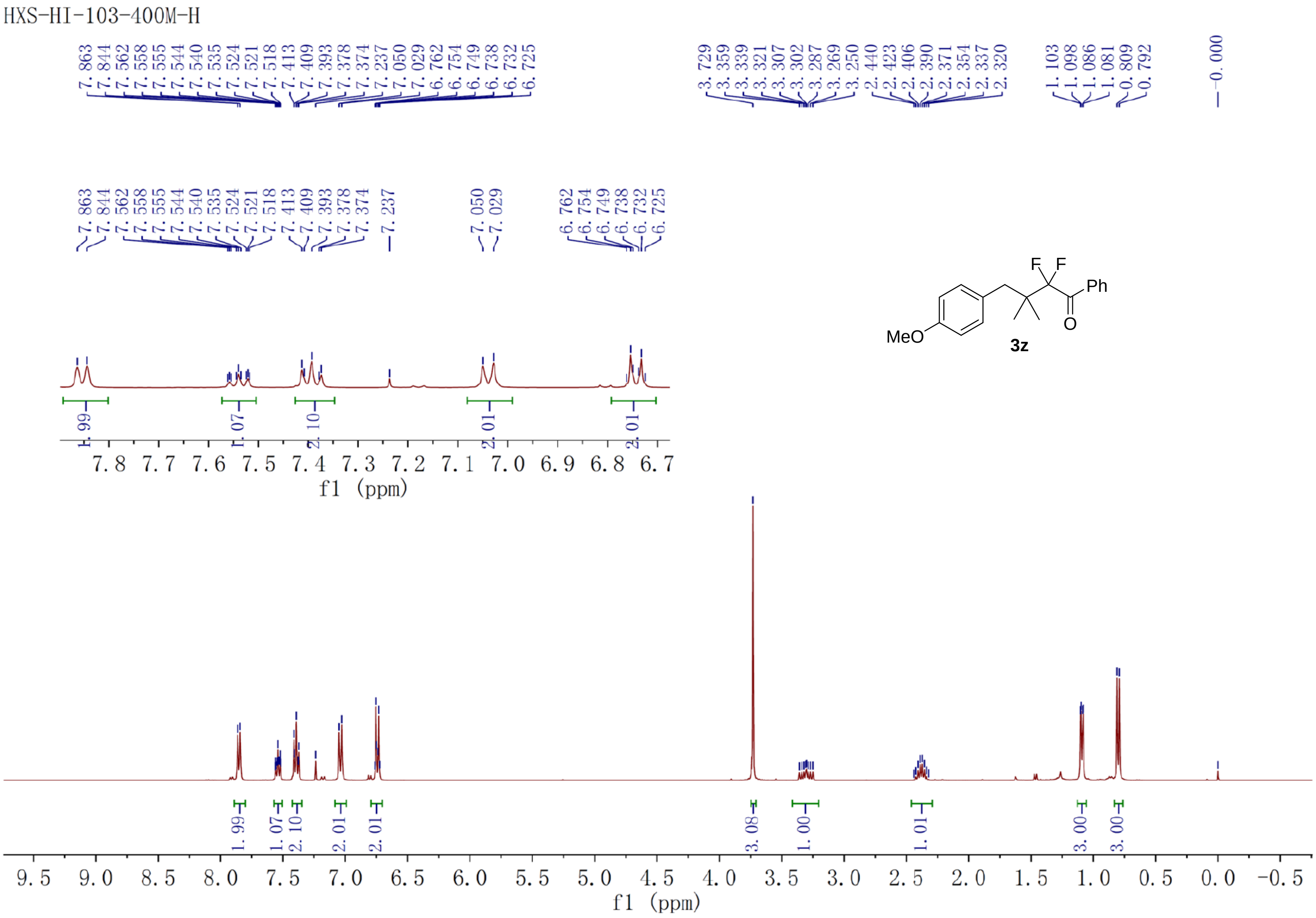

Supplementary Figure 98. ^{1}H NMR (400 MHz, $CDCl_3$) spectra for compound **3z**

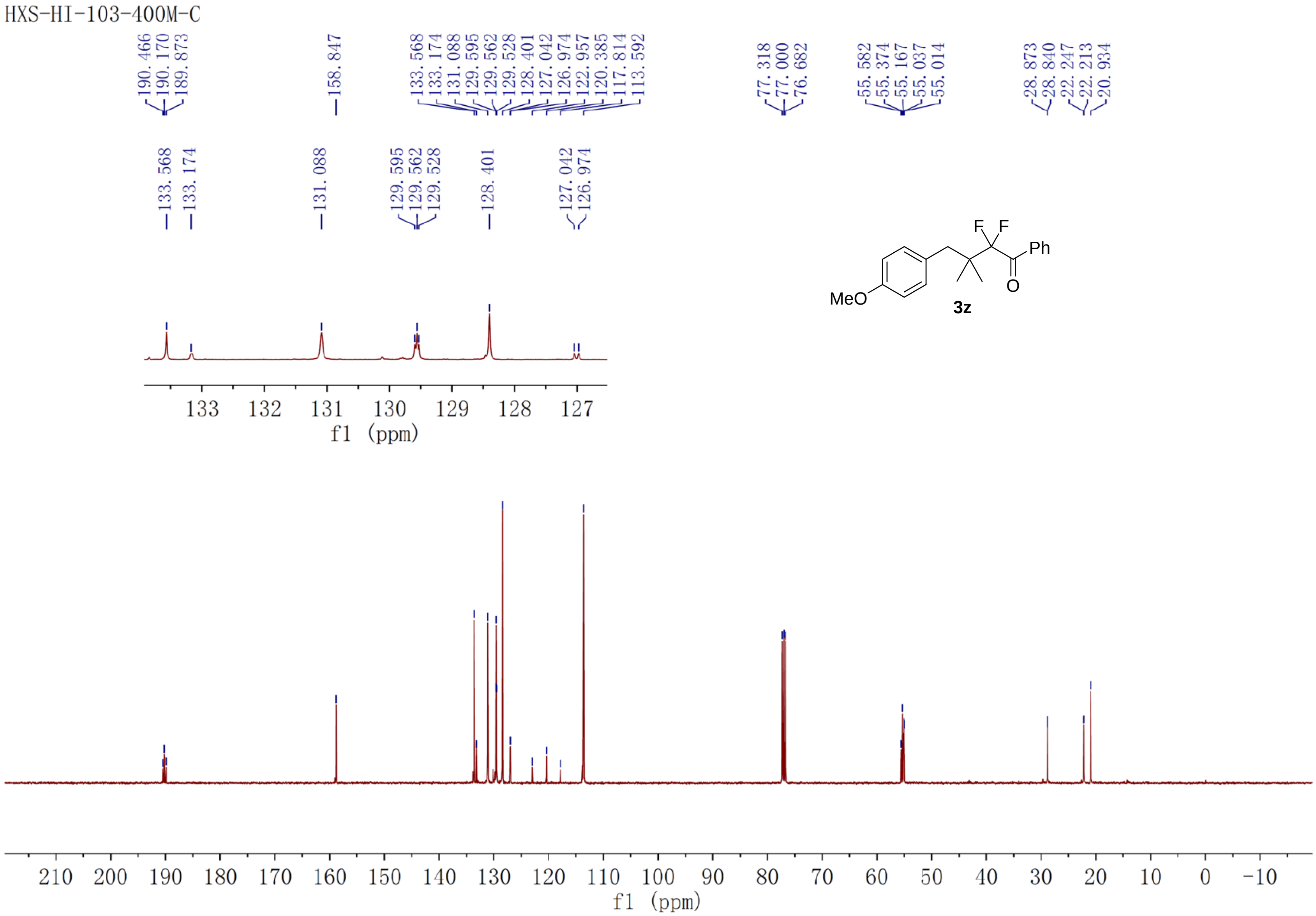

Supplementary Figure 99. ^{13}C NMR (100 MHz, $CDCl_3$) spectra for compound **3z**

HXS-HI-103-400M-F

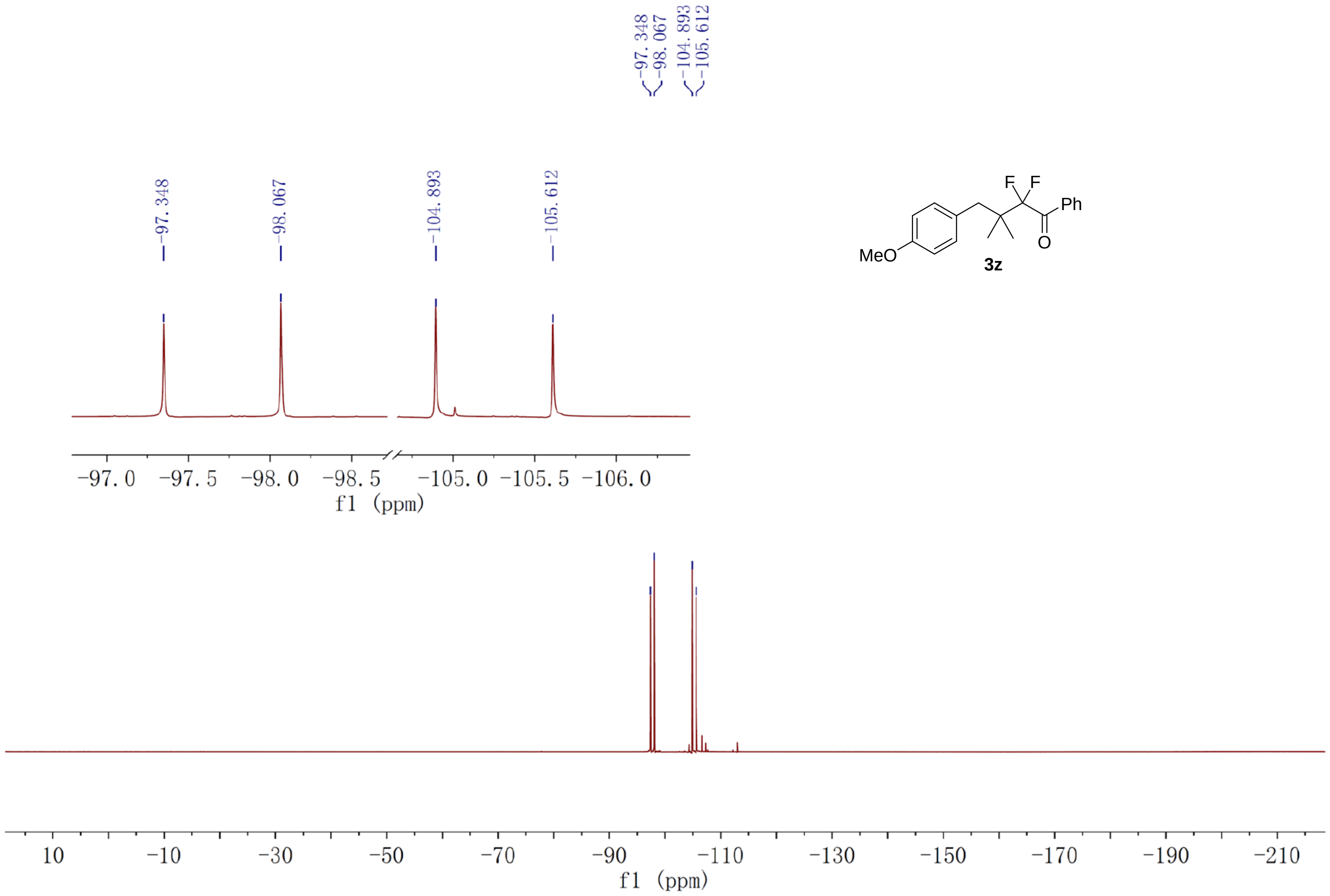

Supplementary Figure 100. ^{19}F NMR (376 MHz, $CDCl_3$) spectra for compound **3z**

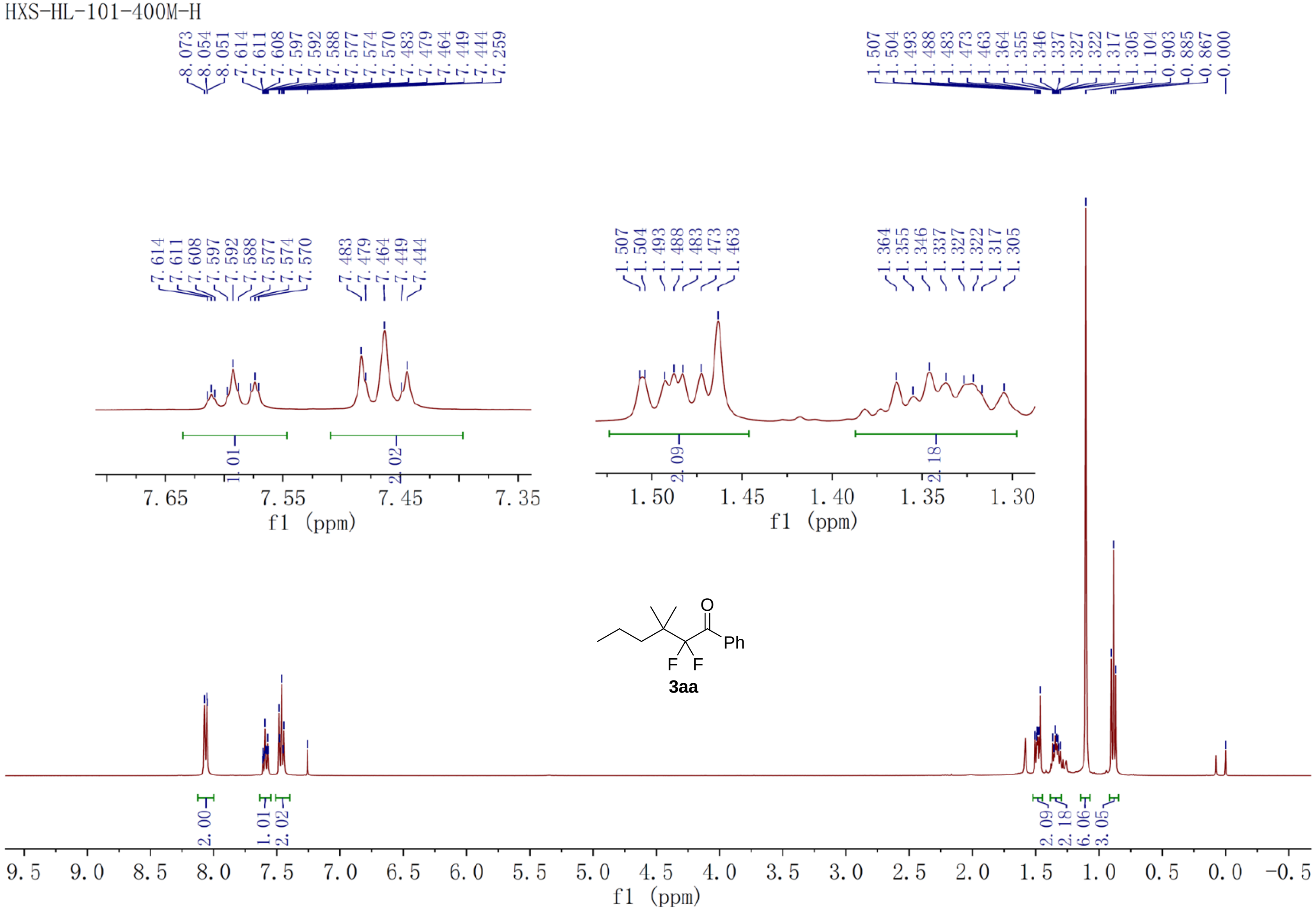

Supplementary Figure 101. ^{1}H NMR (400 MHz, $CDCl_3$) spectra for compound **3aa**

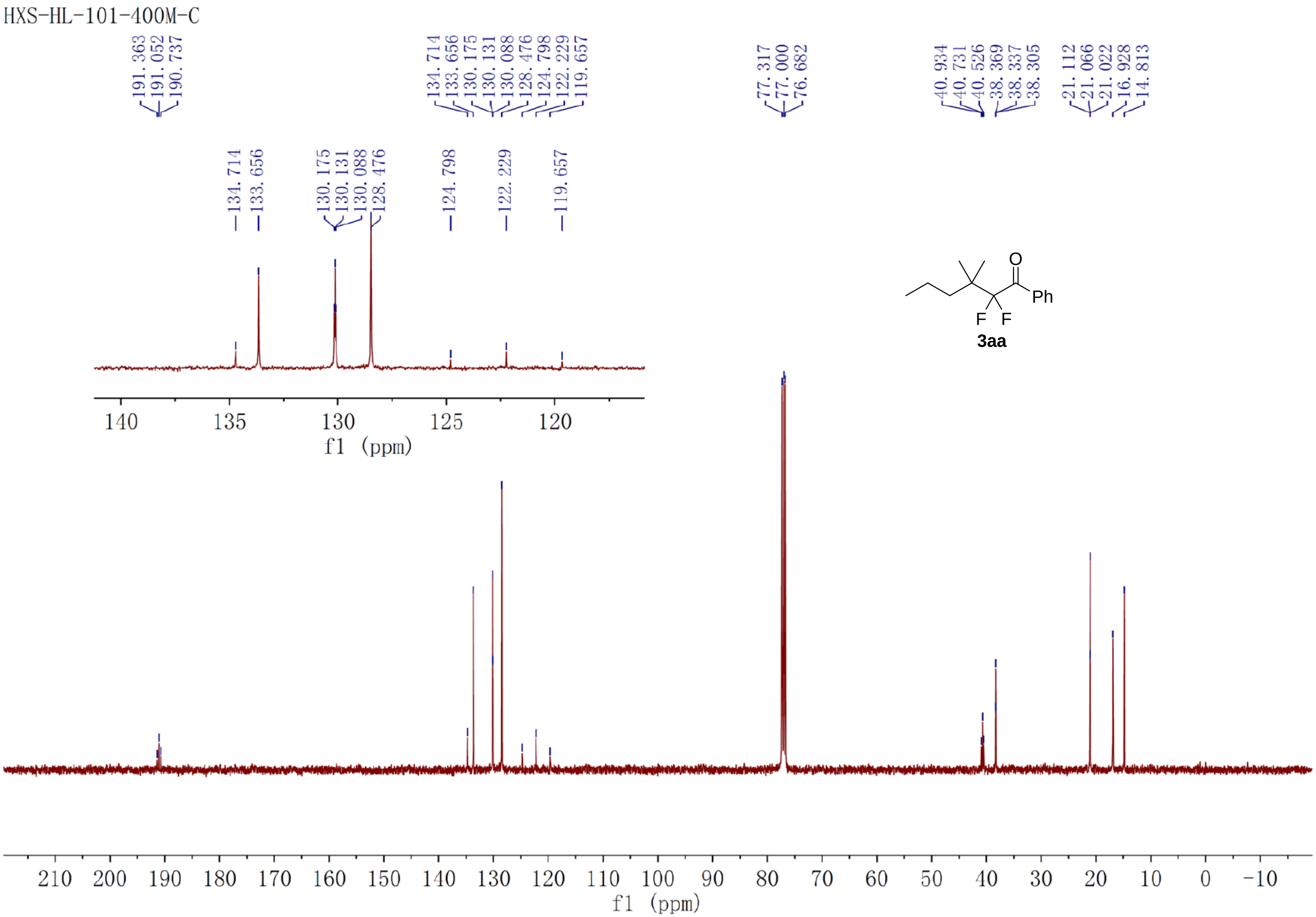

Supplementary Figure 102. ^{13}C NMR (100 MHz, $CDCl_3$) spectra for compound **3aa**

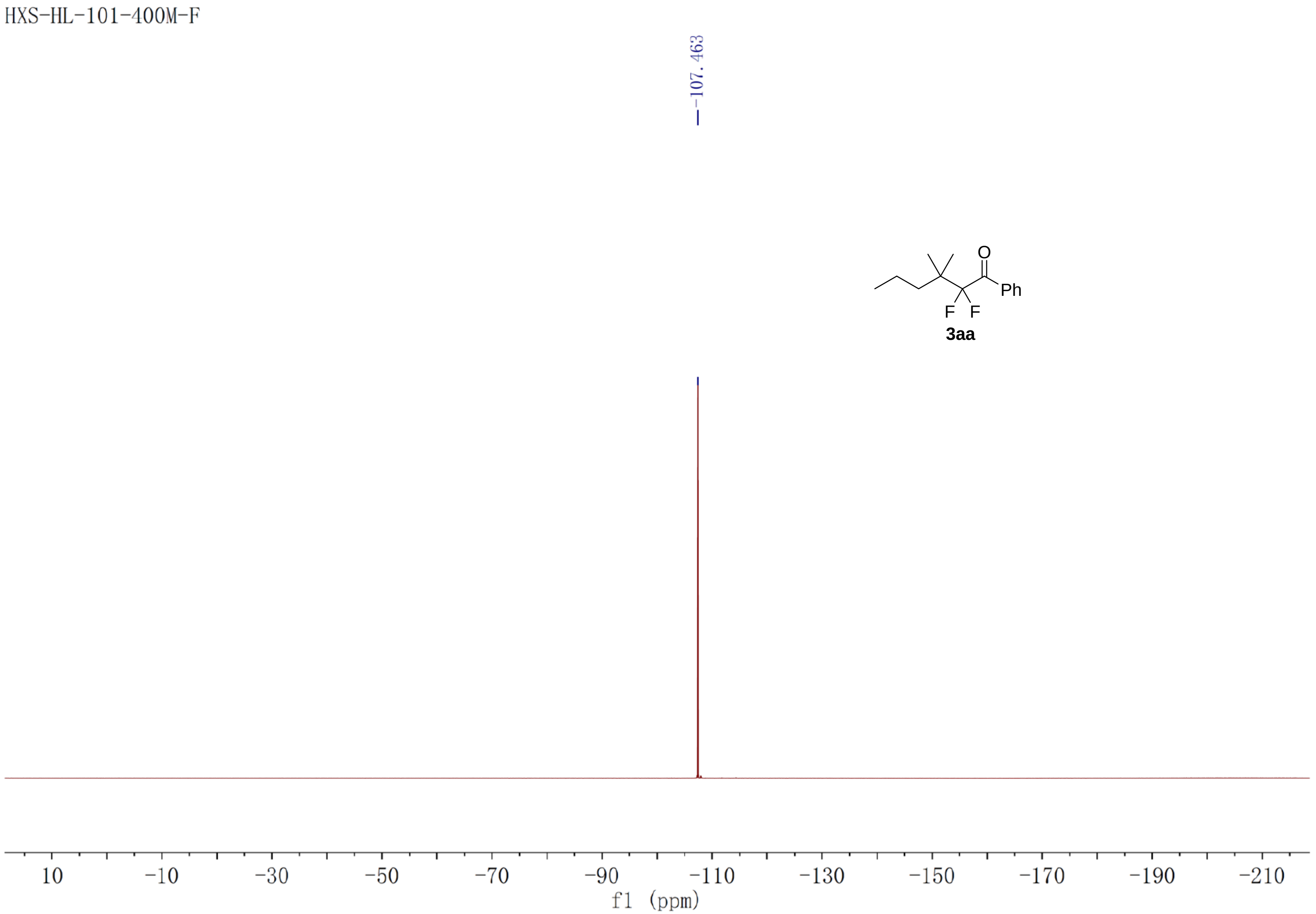

Supplementary Figure 103. ^{19}F NMR (376 MHz, $CDCl_3$) spectra for compound **3aa**

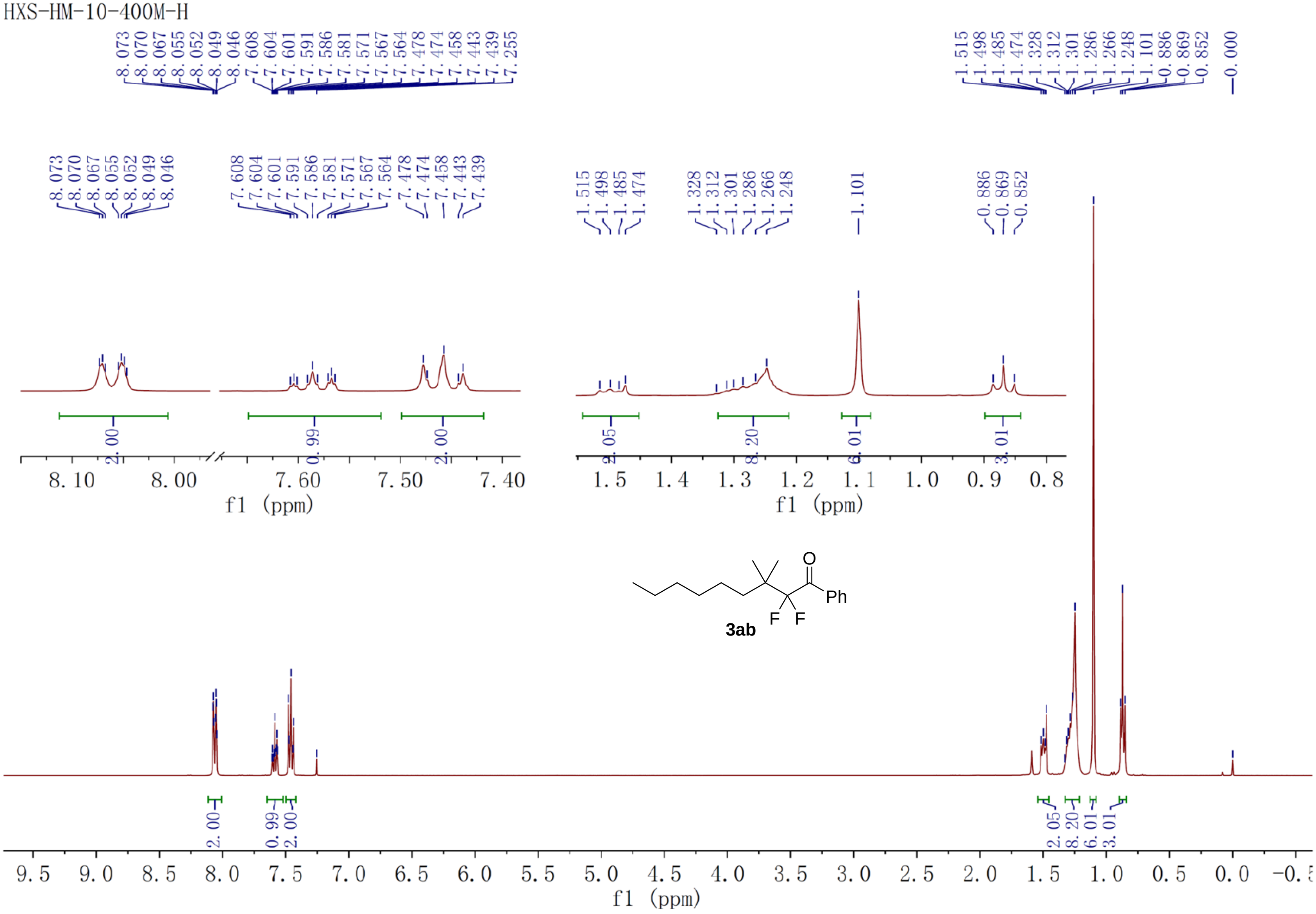

Supplementary Figure 104. ^{1}H NMR (400 MHz, $CDCl_3$) spectra for compound **3ab**

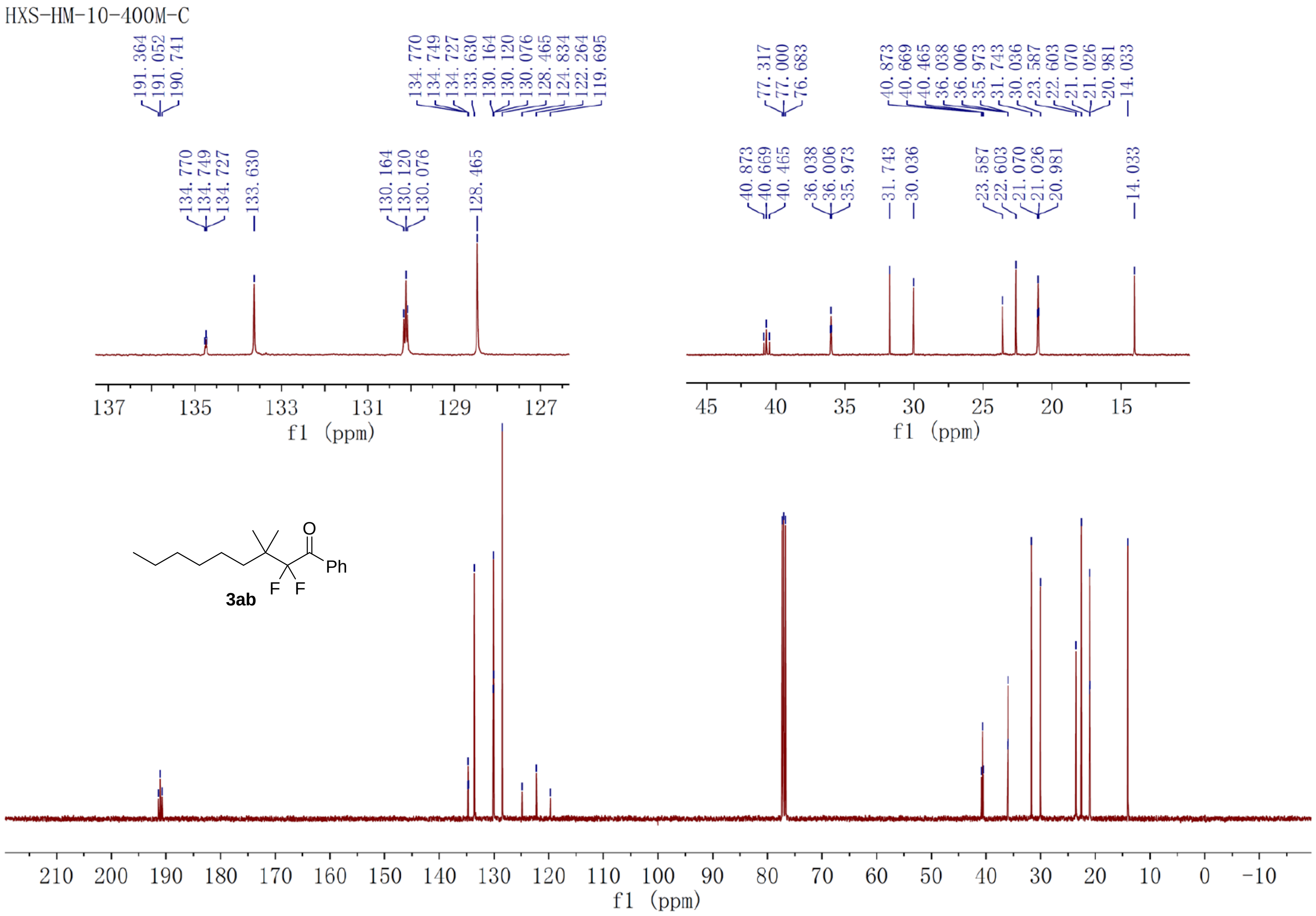

Supplementary Figure 105. ^{13}C NMR (100 MHz, $CDCl_3$) spectra for compound **3ab**

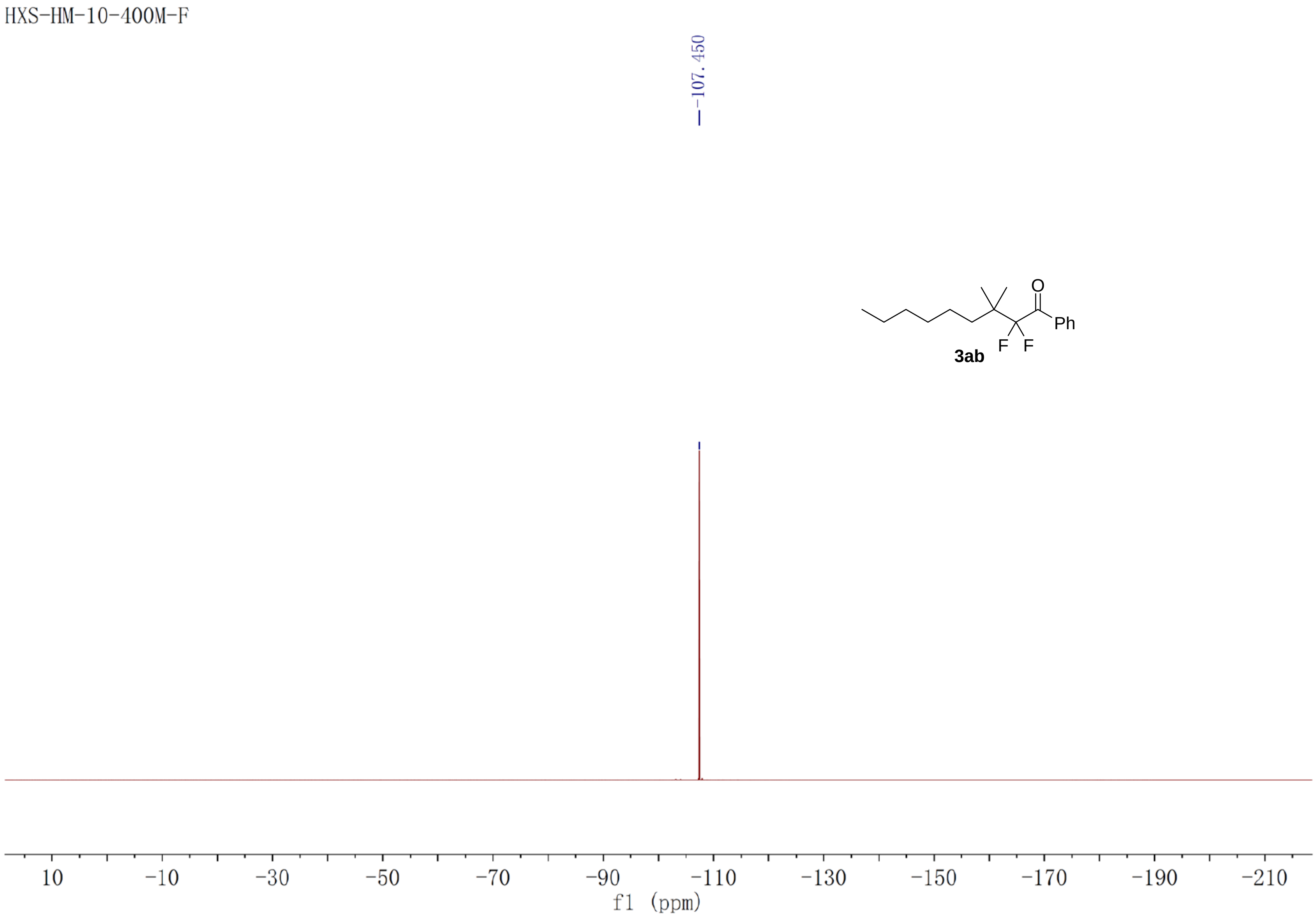

Supplementary Figure 106. ^{19}F NMR (376 MHz, $CDCl_3$) spectra for compound **3ab**

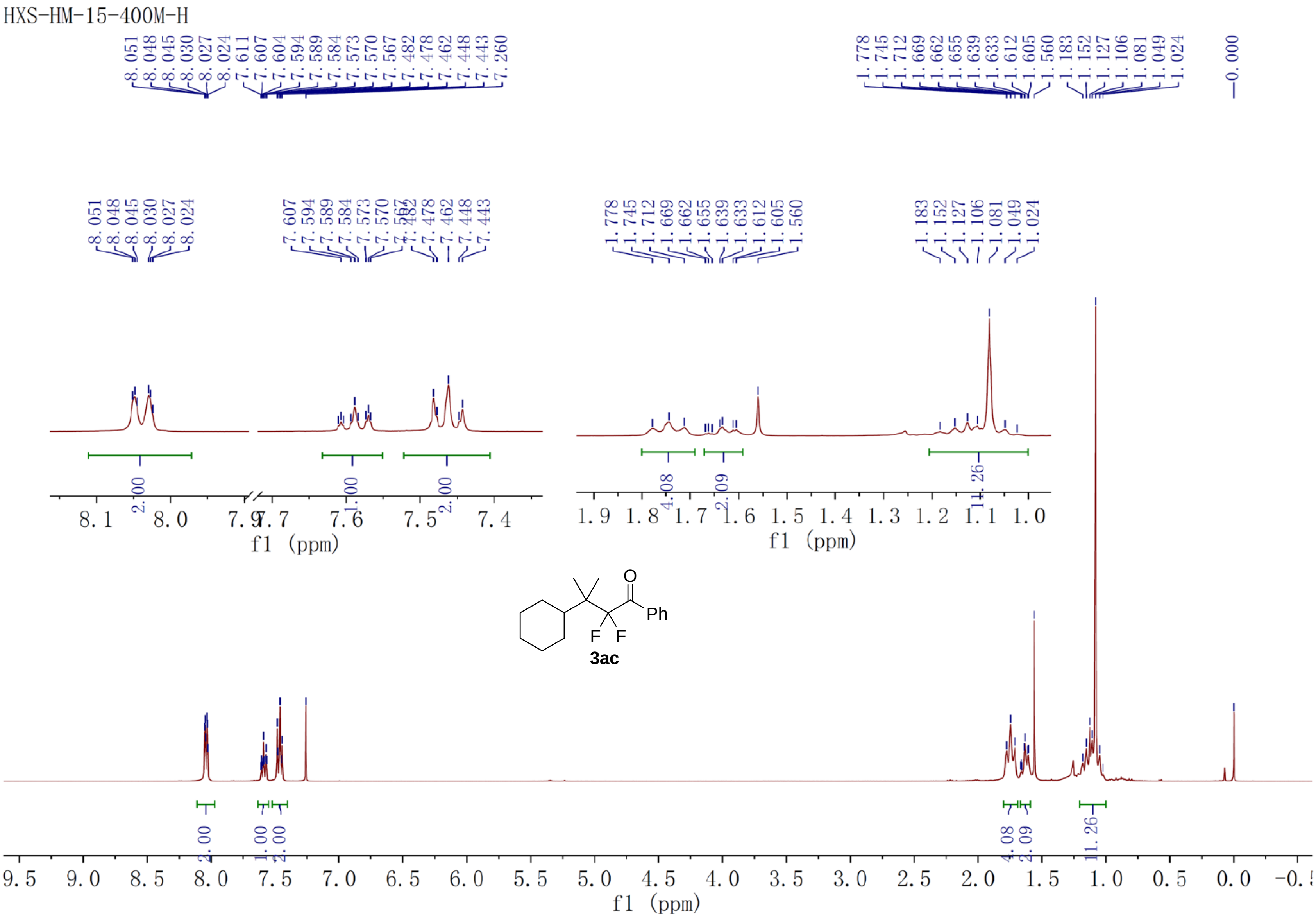

Supplementary Figure 107. ^{1}H NMR (400 MHz, $CDCl_3$) spectra for compound **3ac**

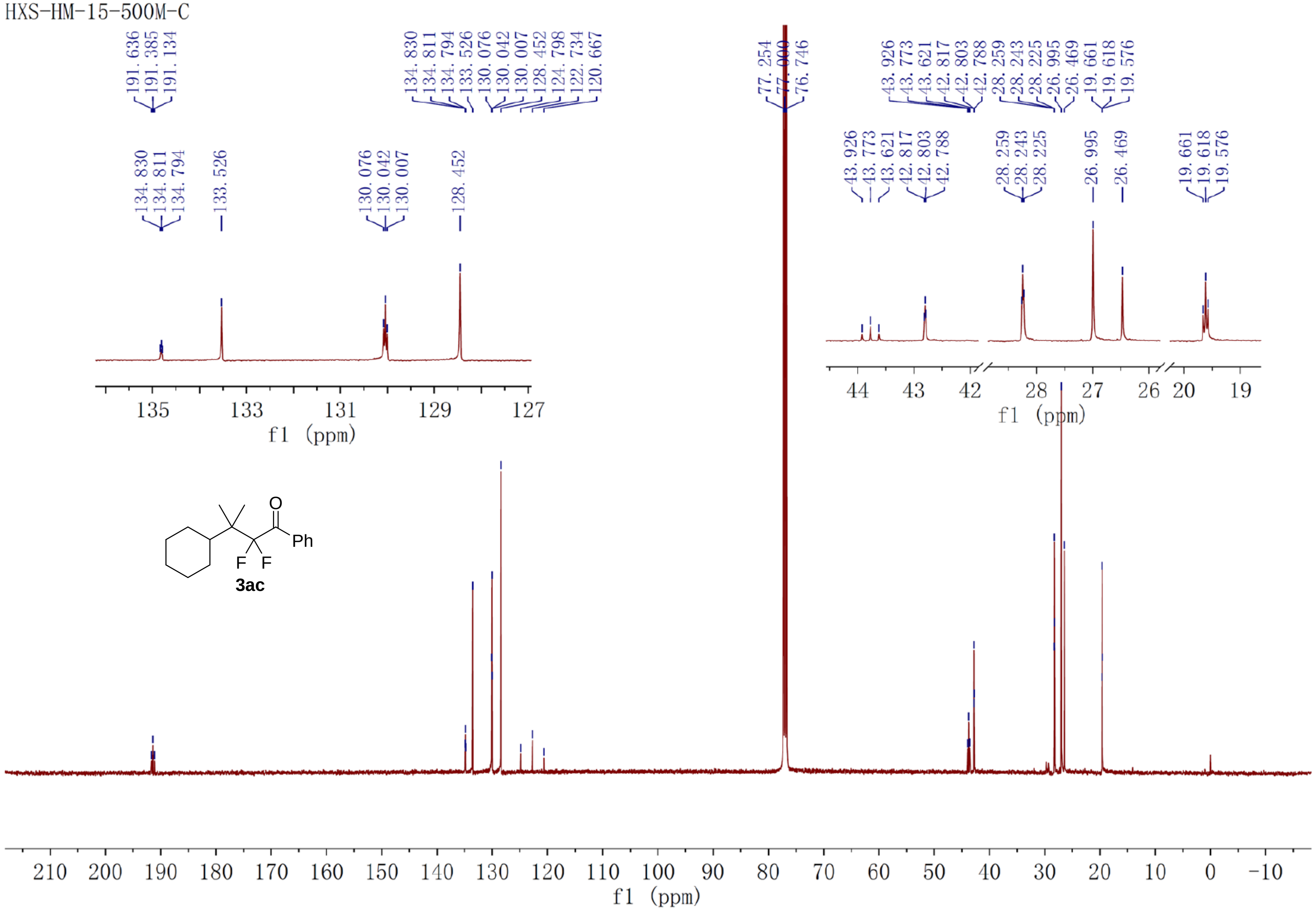

Supplementary Figure 108. ^{13}C NMR (125 MHz, $CDCl_3$) spectra for compound **3ac**

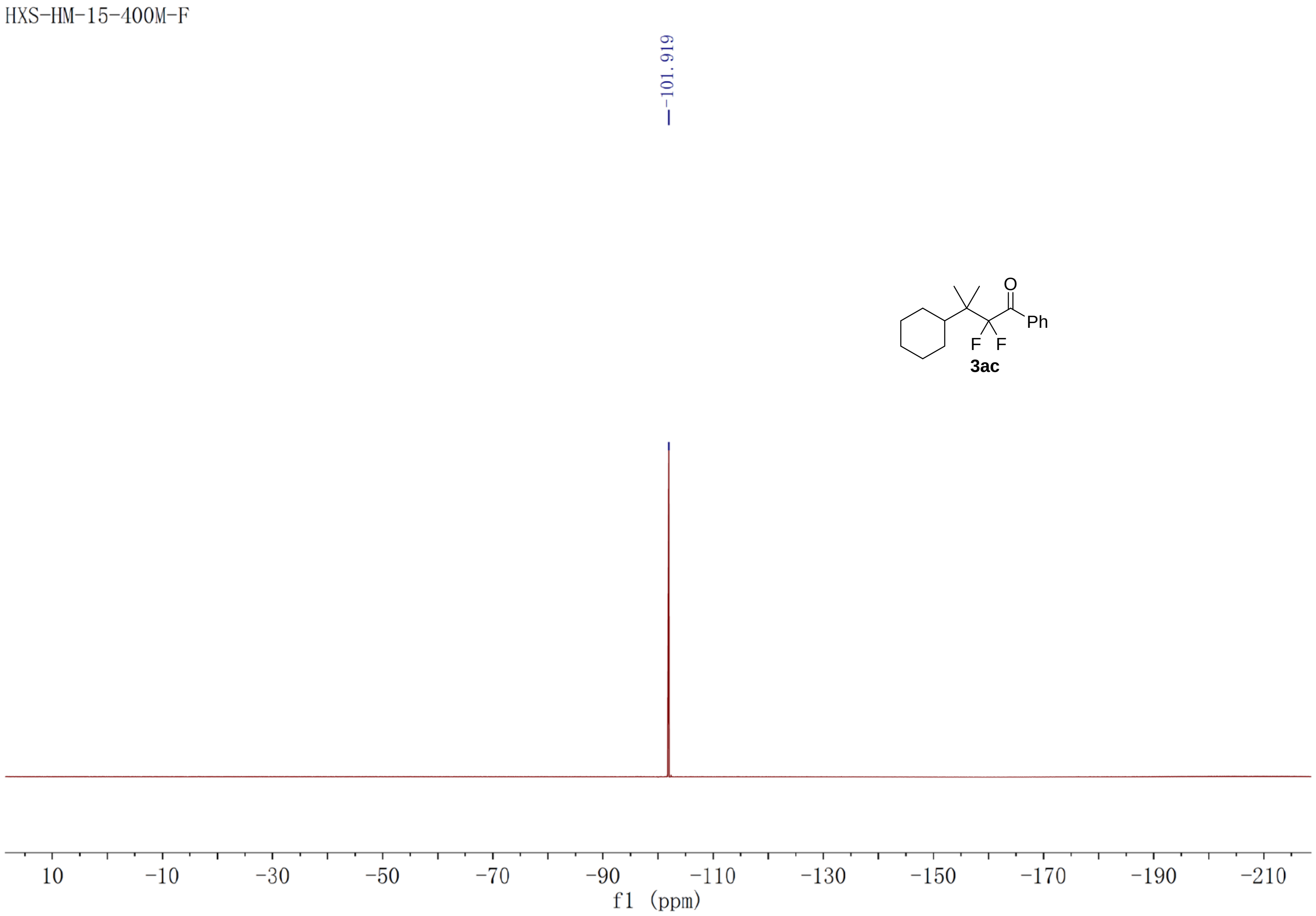

Supplementary Figure 109. ^{19}F NMR (376 MHz, $CDCl_3$) spectra for compound **3ac**

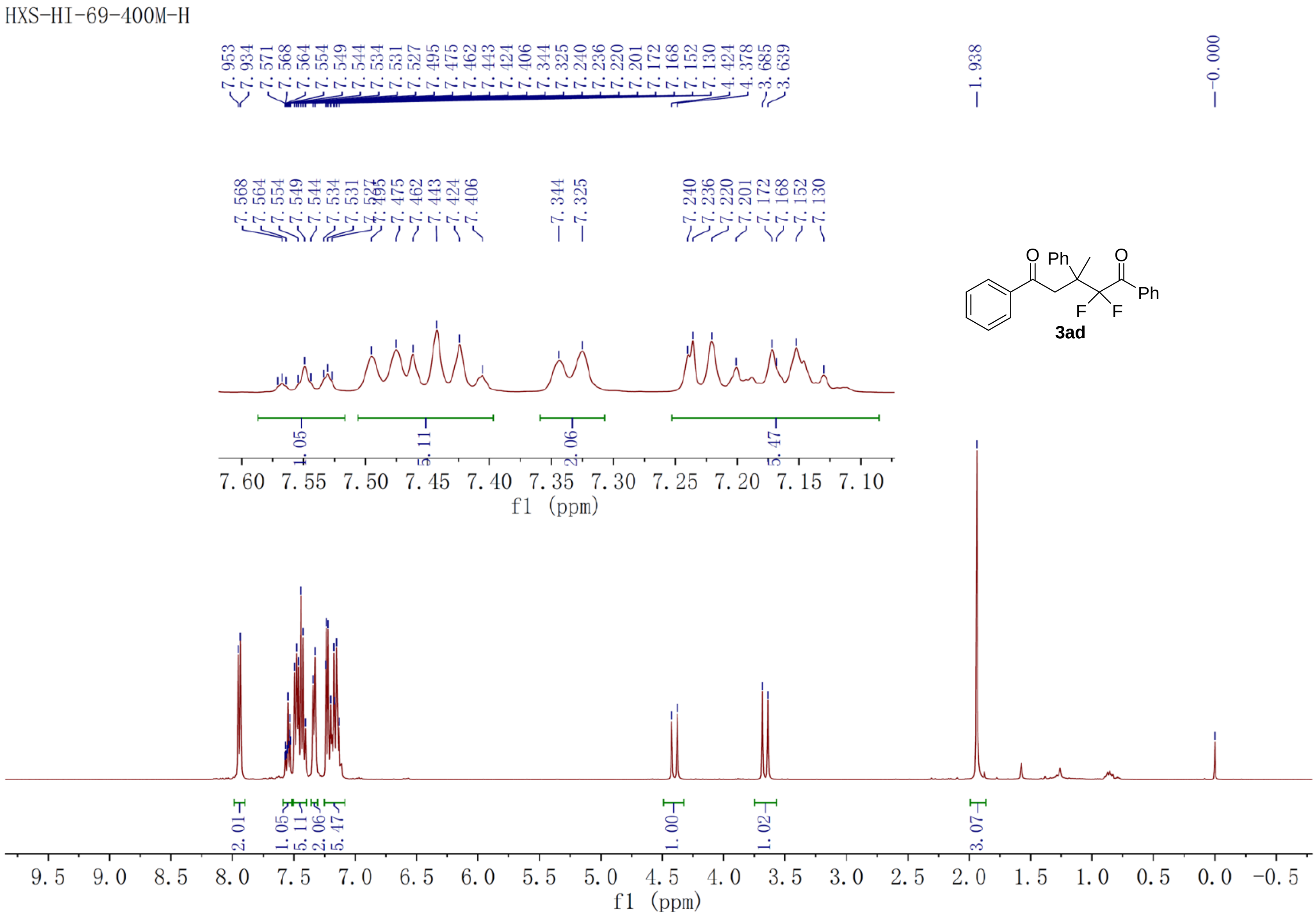

Supplementary Figure 110. ^{1}H NMR (400 MHz, $CDCl_3$) spectra for compound **3ad**

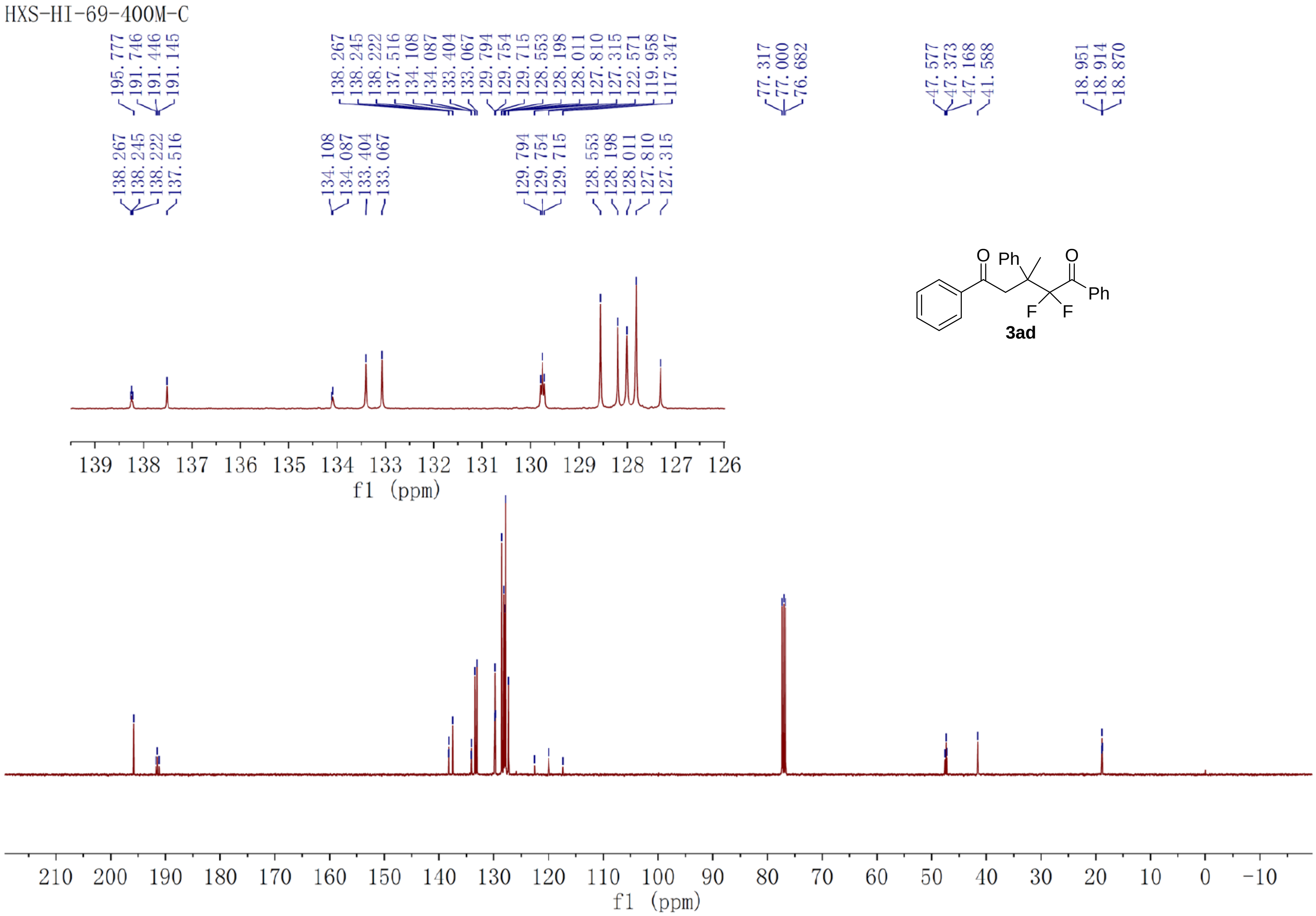

Supplementary Figure 111. ^{13}C NMR (100 MHz, $CDCl_3$) spectra for compound **3ad**

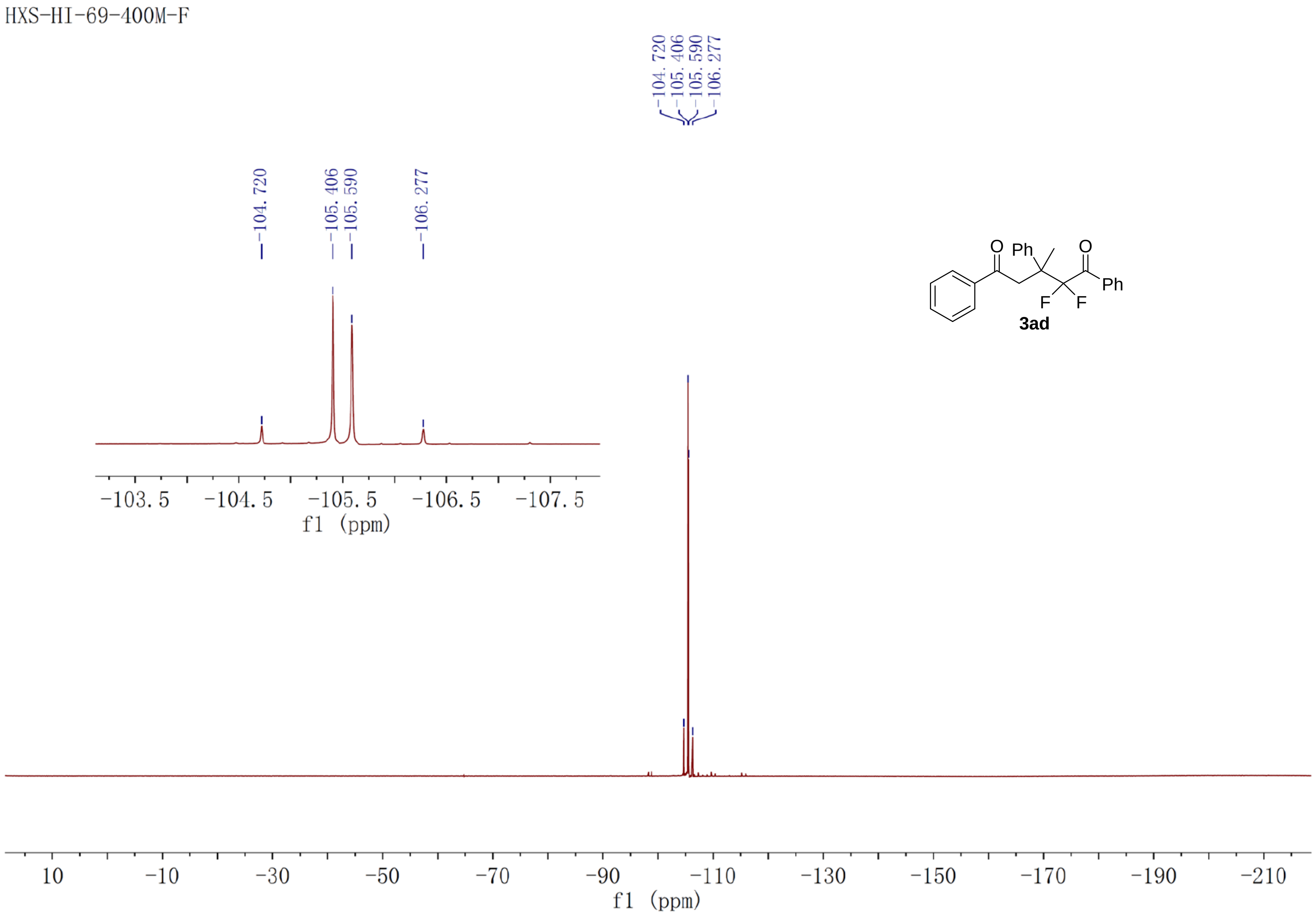

Supplementary Figure 112. ^{19}F NMR (376 MHz, $CDCl_3$) spectra for compound **3ad**

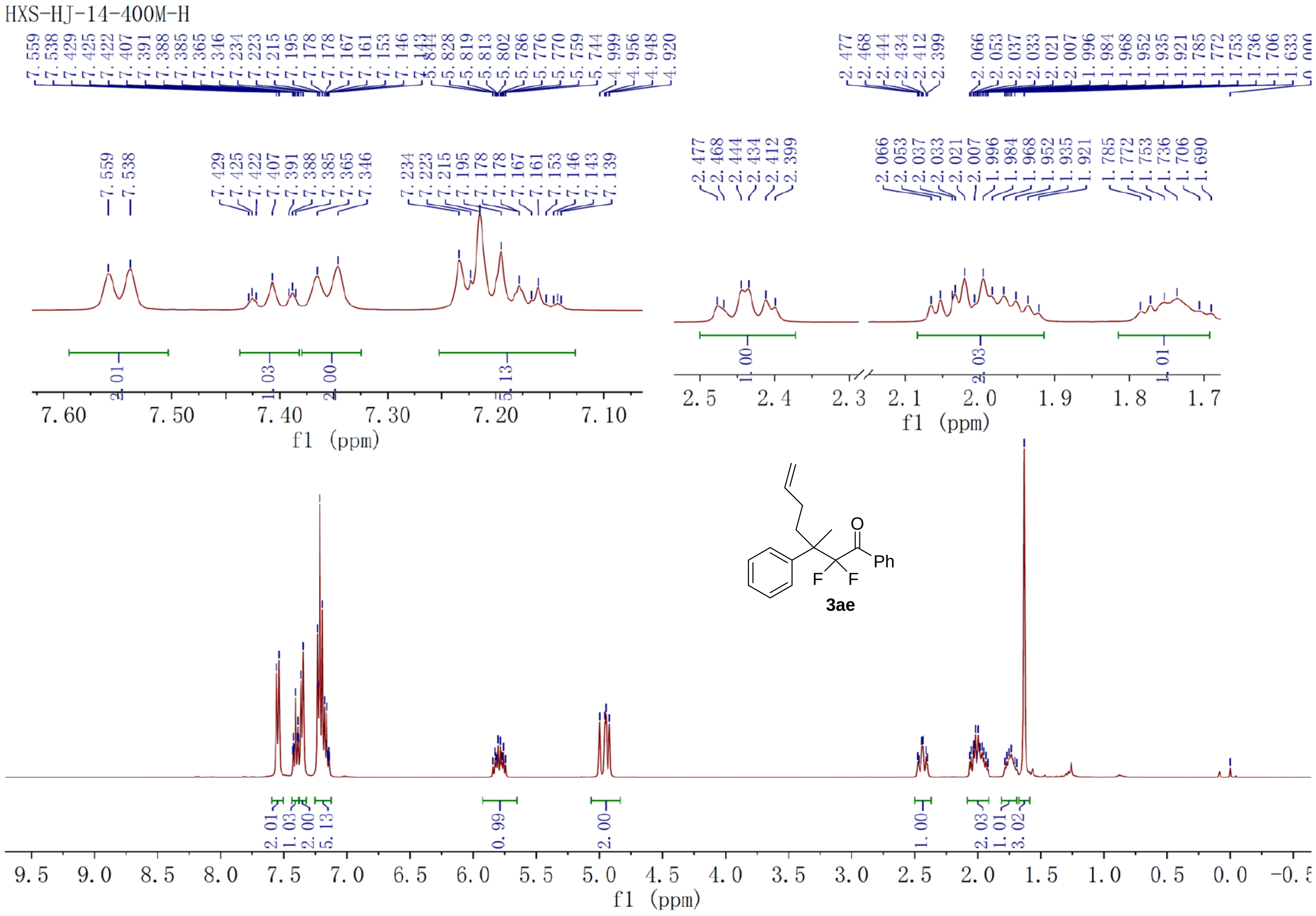

Supplementary Figure 113. ^{1}H NMR (400 MHz, $CDCl_3$) spectra for compound **3ae**

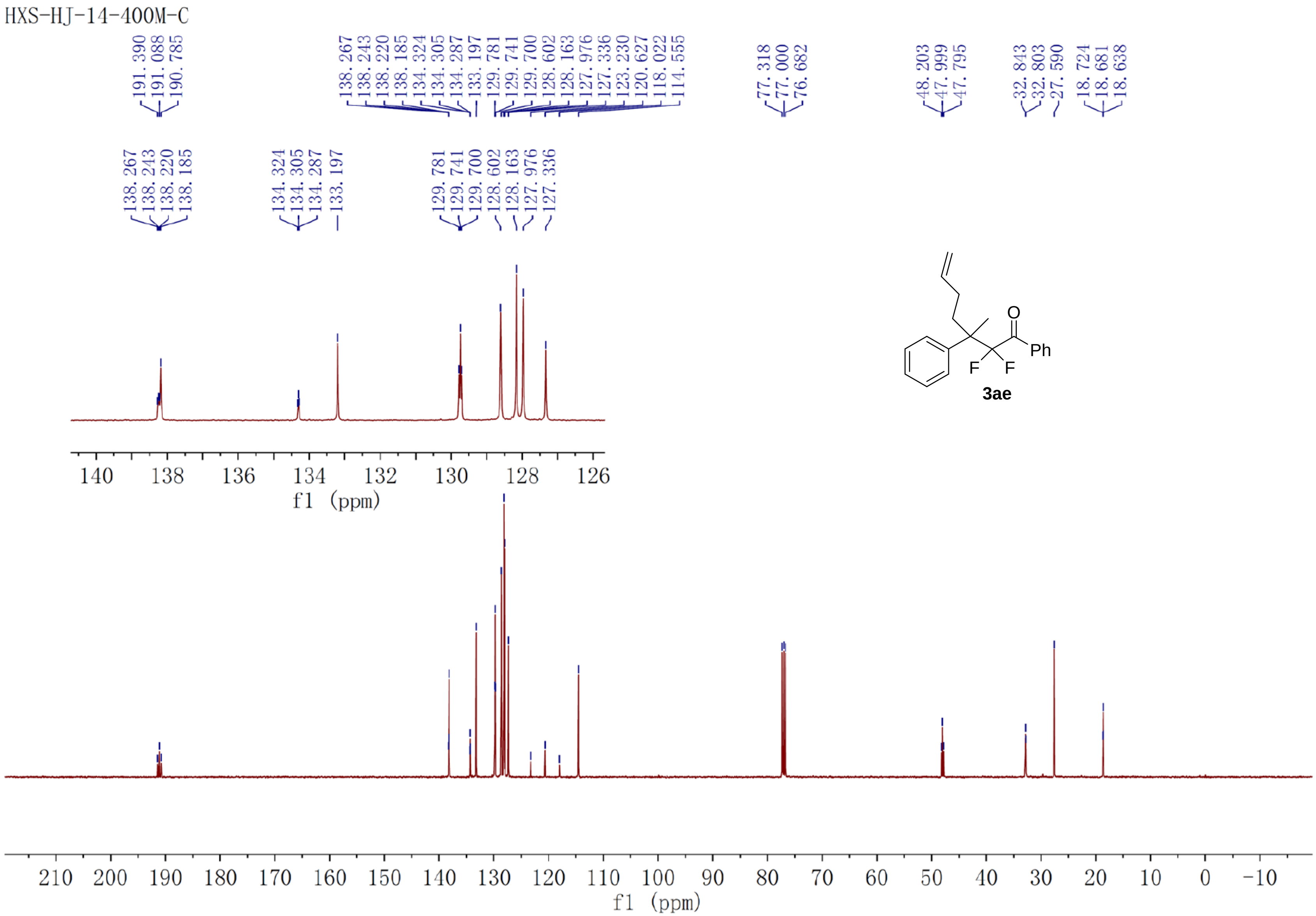

Supplementary Figure 114. ^{13}C NMR (100 MHz, $CDCl_3$) spectra for compound **3ae**

HXS-HJ-14-400M-F

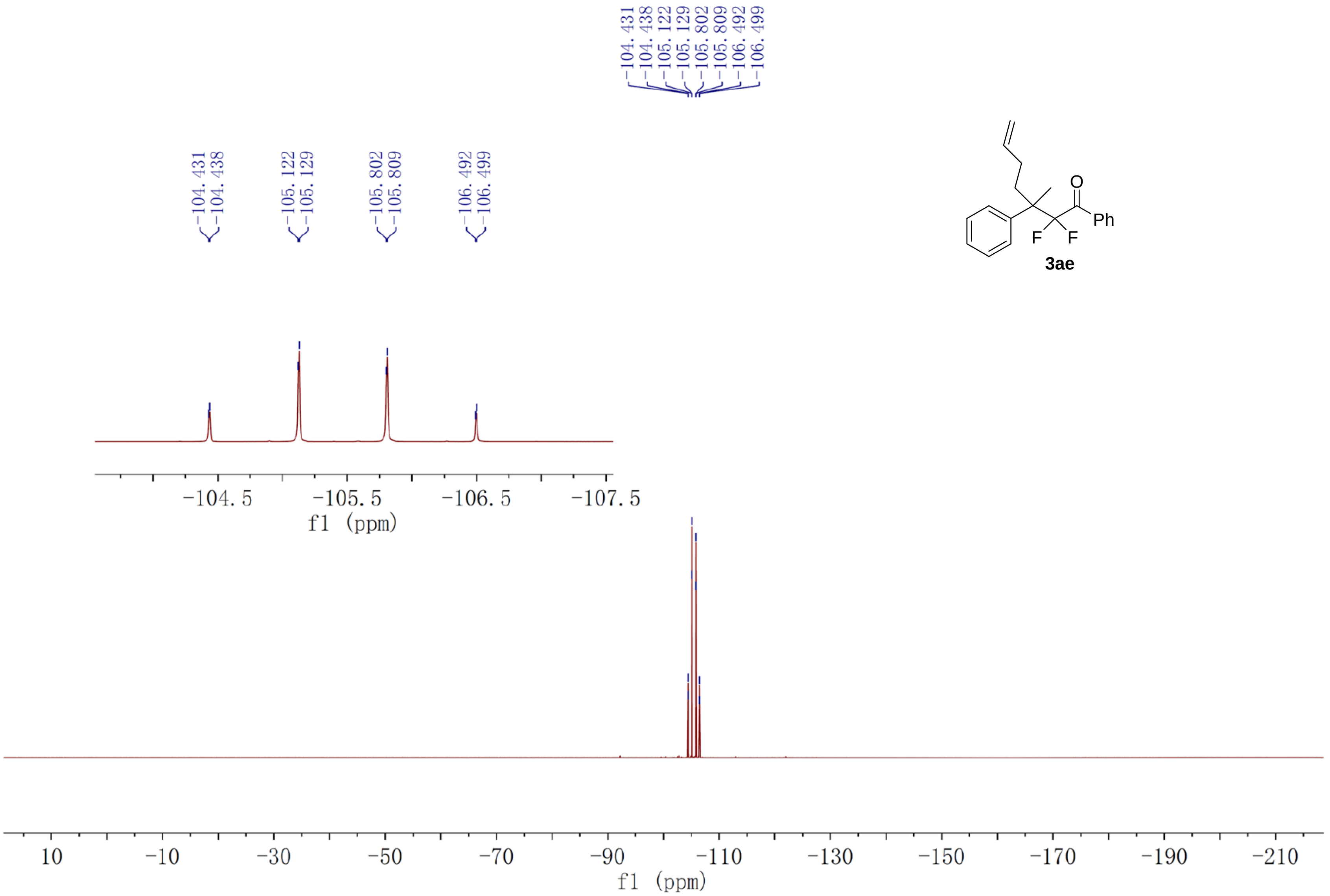

3ae

Supplementary Figure 115. ^{19}F NMR (376 MHz, $CDCl_3$) spectra for compound **3ae**

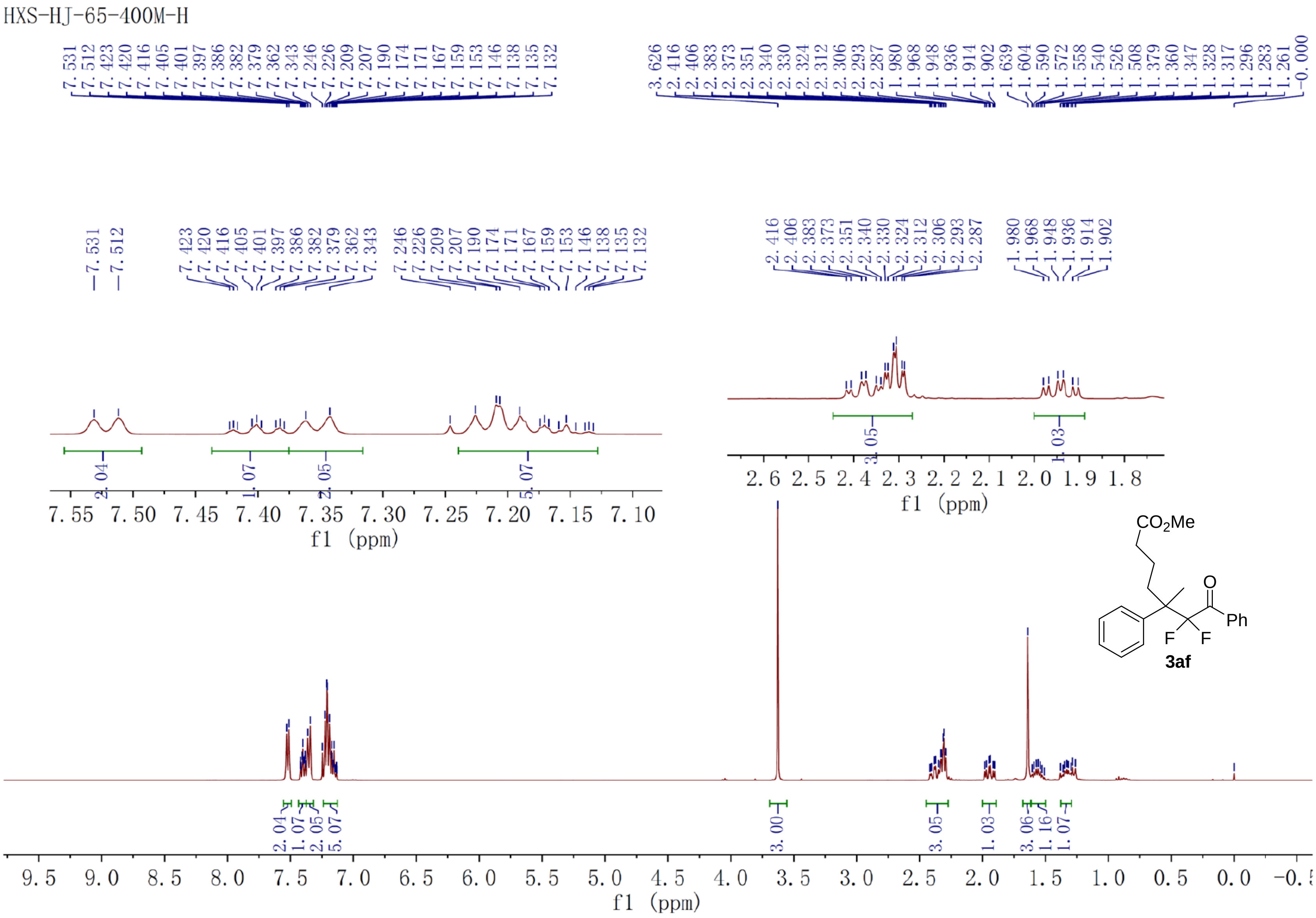

Supplementary Figure 116. ^{1}H NMR (400 MHz, $CDCl_3$) spectra for compound **3af**

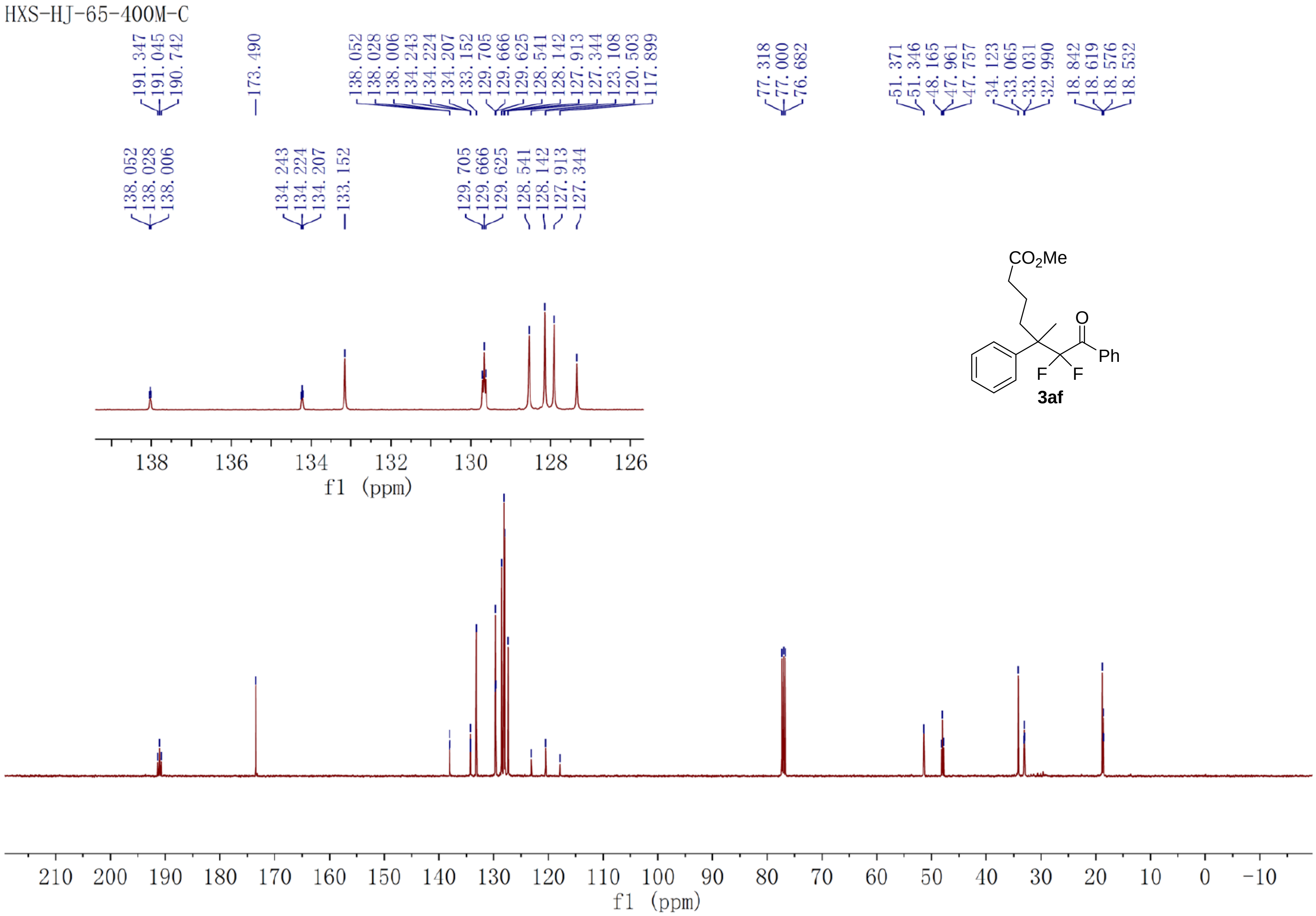

Supplementary Figure 117. ^{13}C NMR (100 MHz, $CDCl_3$) spectra for compound **3af**

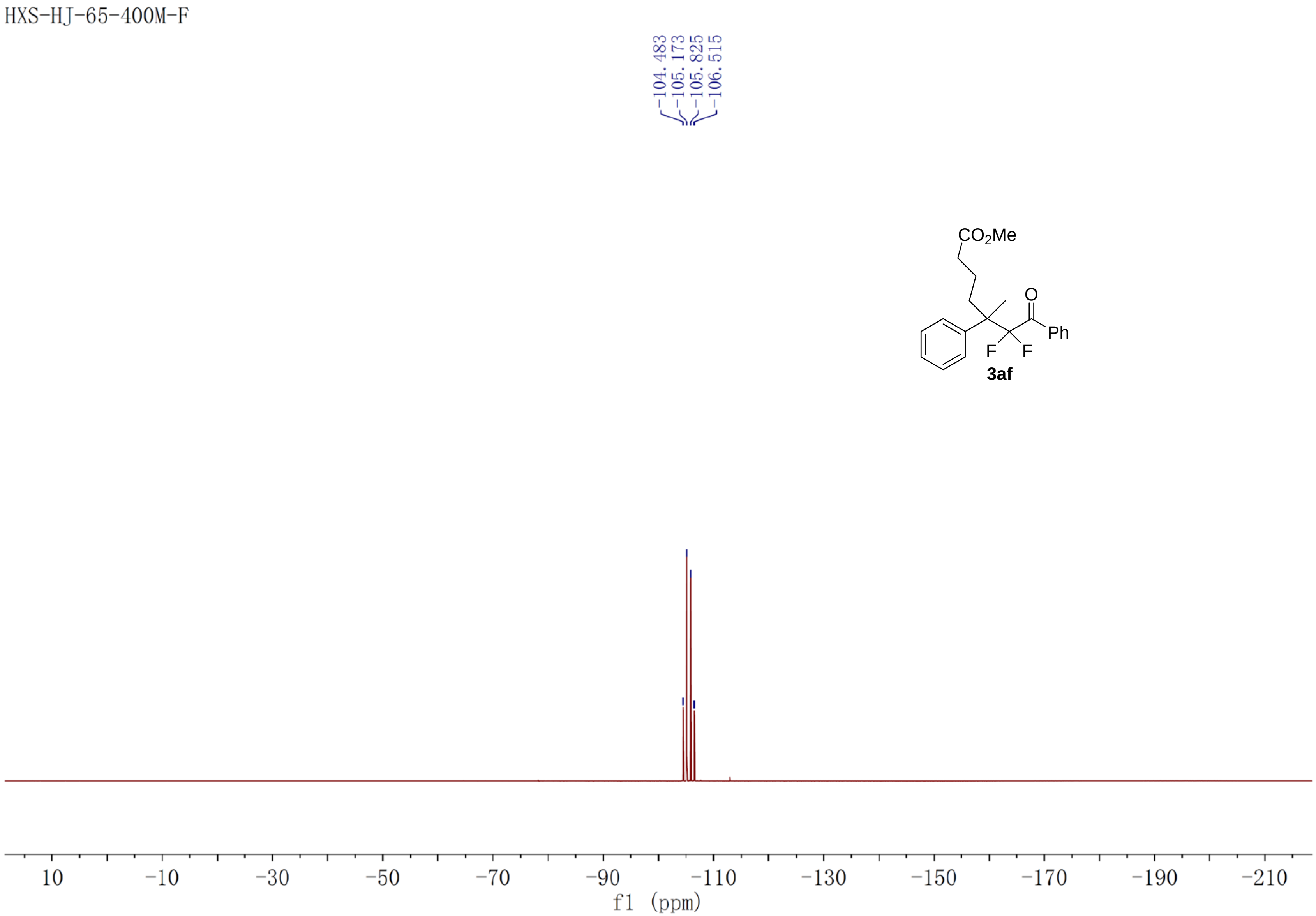

Supplementary Figure 118. ^{19}F NMR (376 MHz, $CDCl_3$) spectra for compound **3af**

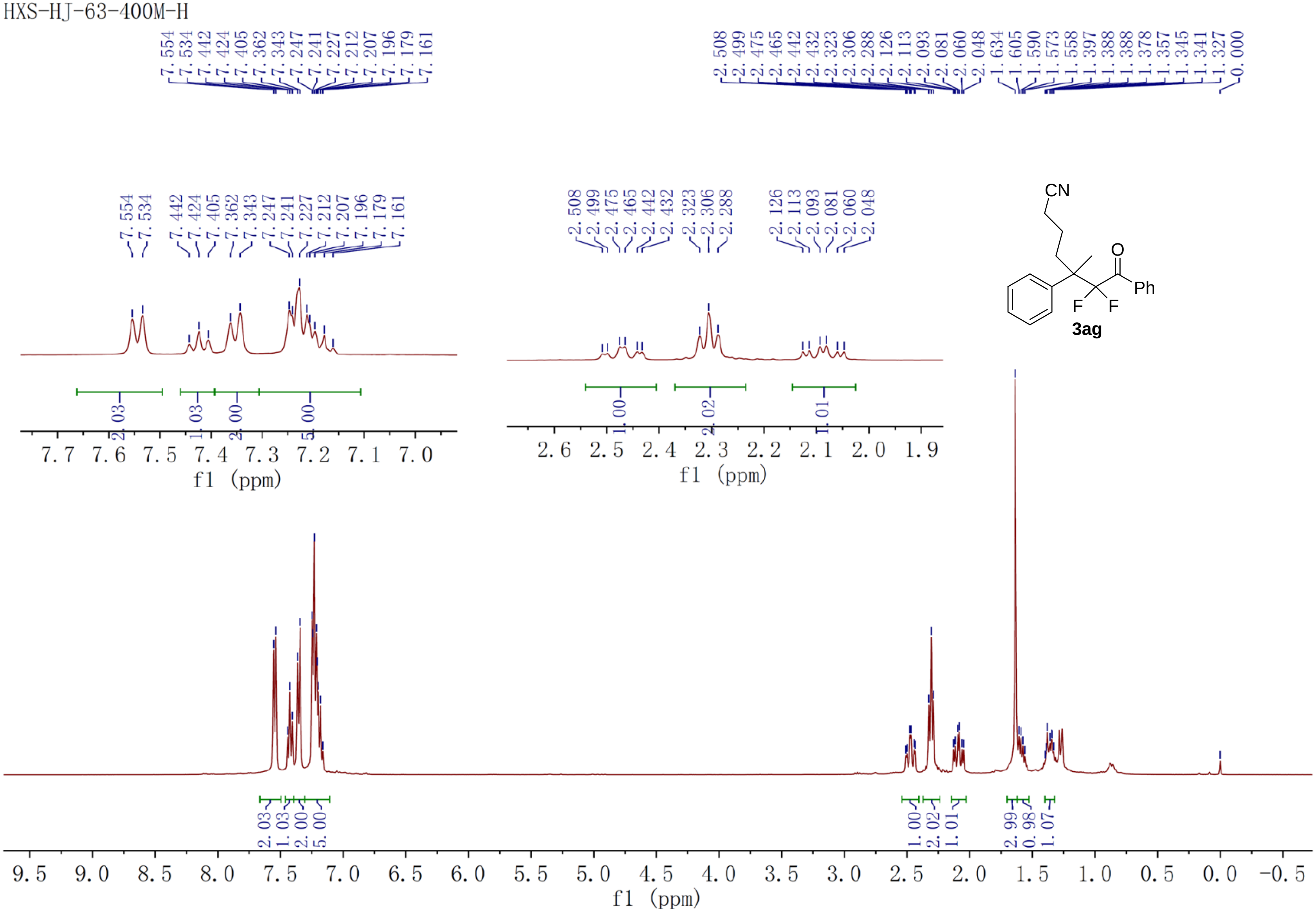

Supplementary Figure 119. ^{1}H NMR (400 MHz, $CDCl_3$) spectra for compound **3ag**

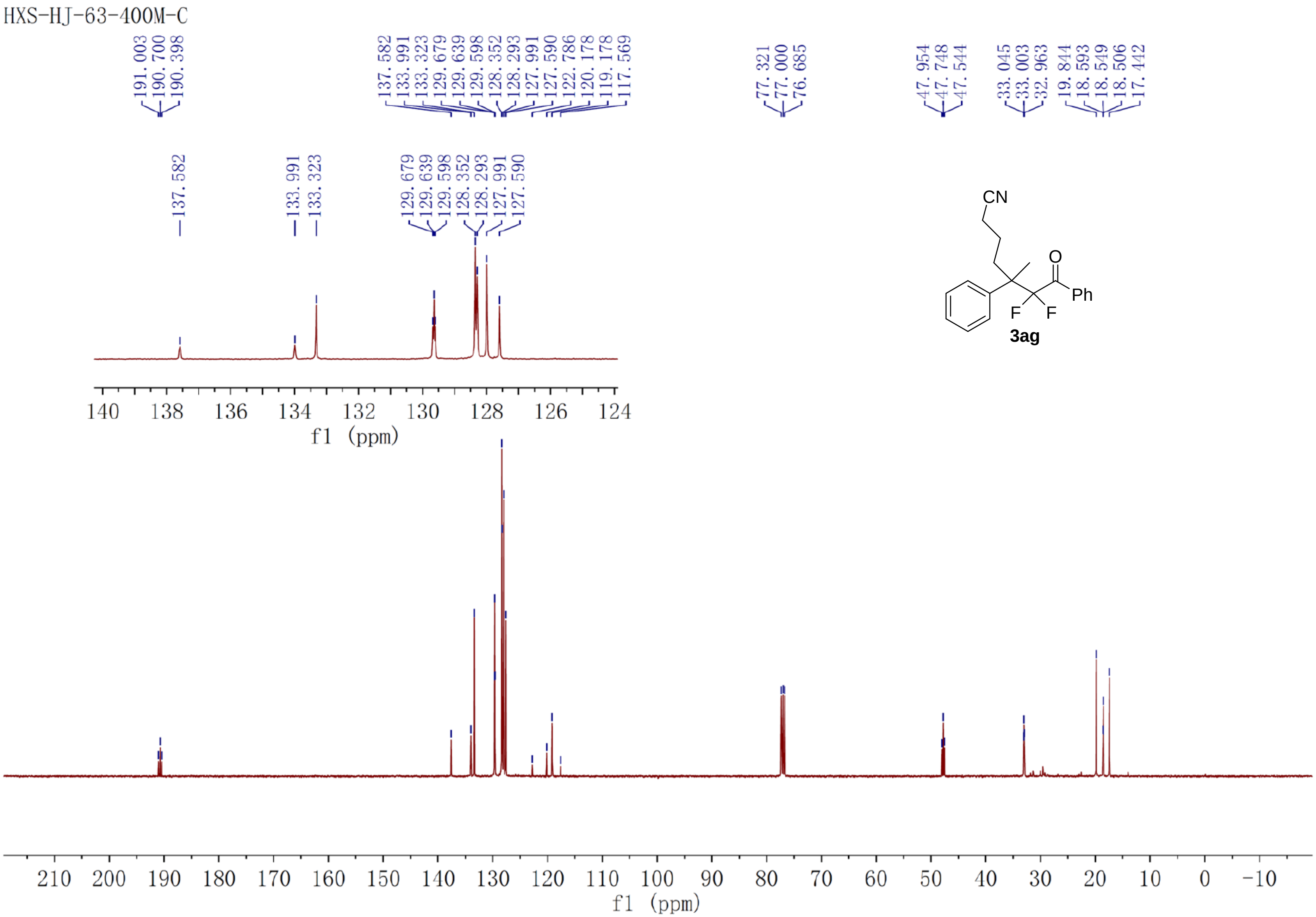

Supplementary Figure 120. ^{13}C NMR (100 MHz, $CDCl_3$) spectra for compound **3ag**

HXS-HJ-63-400M-F

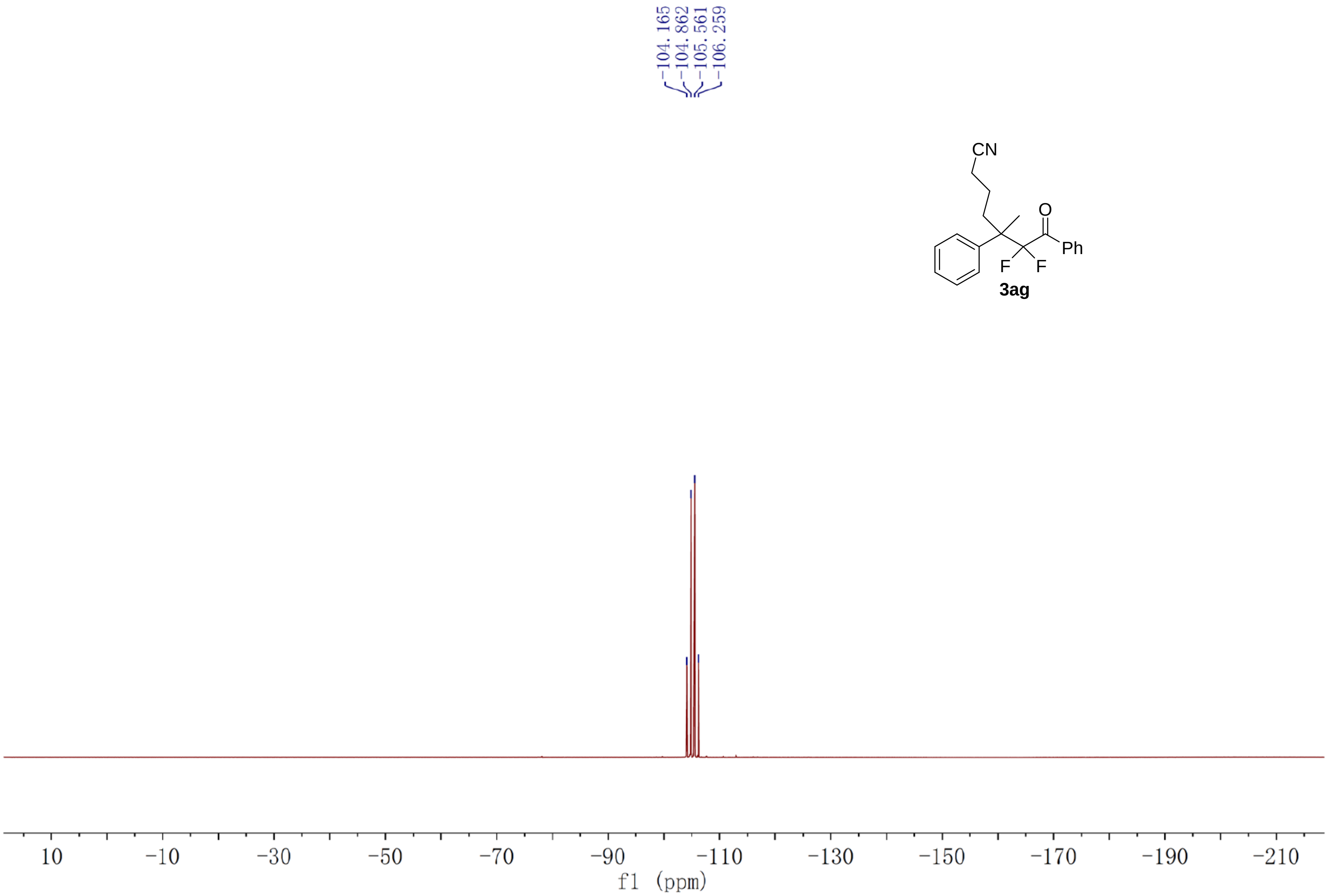

Supplementary Figure 121. ^{19}F NMR (376 MHz, $CDCl_3$) spectra for compound **3ag**

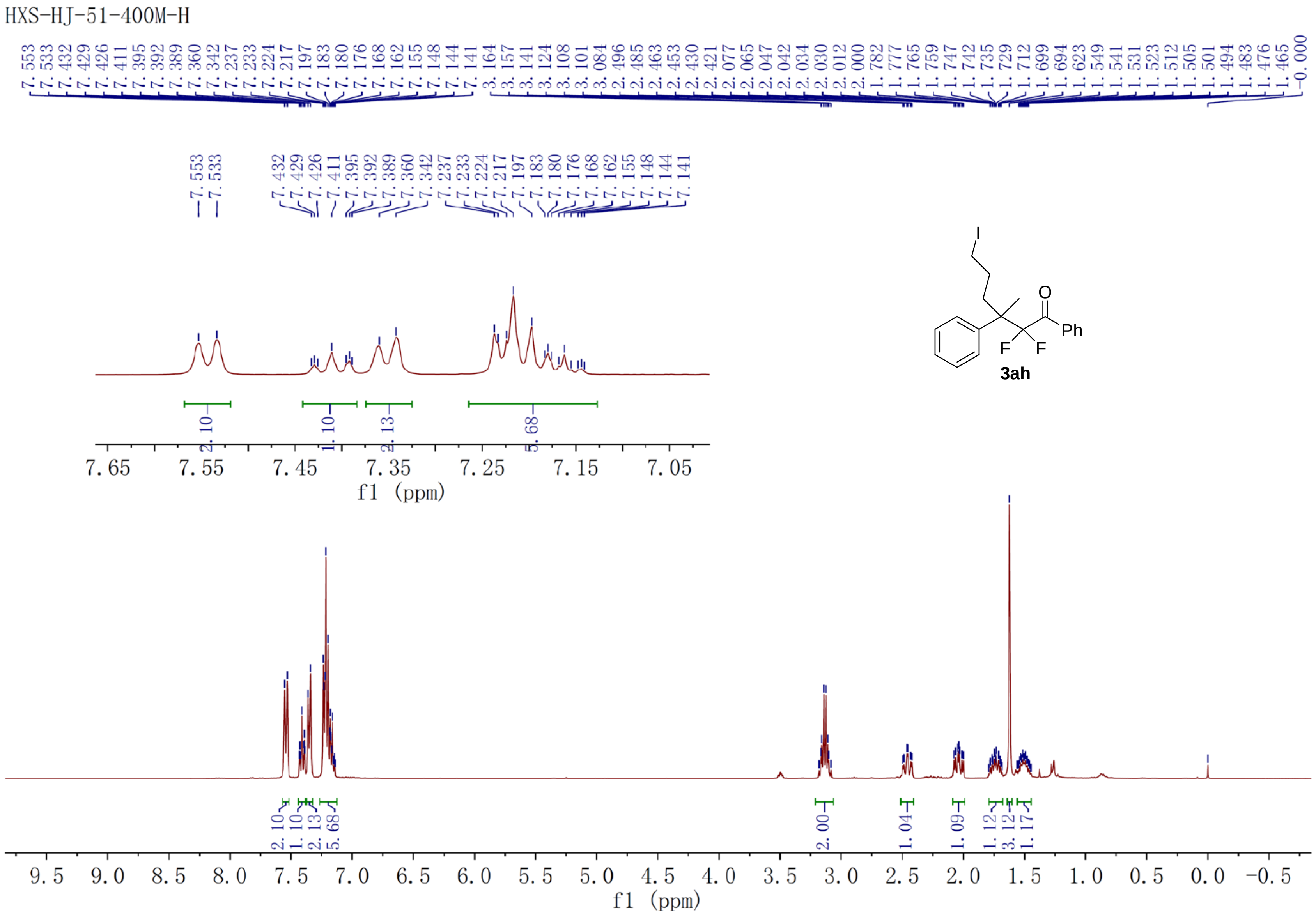

Supplementary Figure 122. ^{1}H NMR (400 MHz, $CDCl_3$) spectra for compound **3ah**

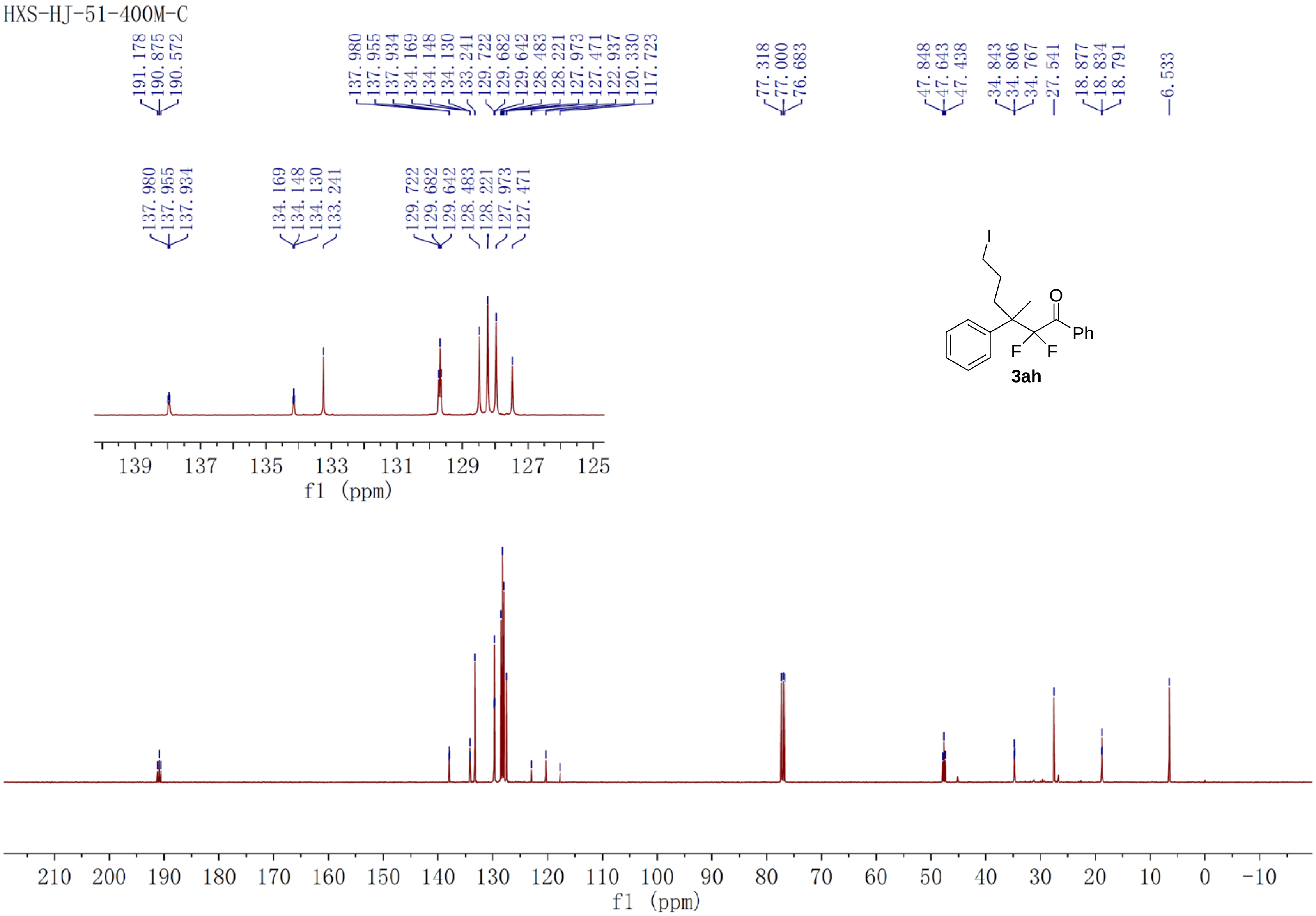

Supplementary Figure 123. ^{13}C NMR (100 MHz, $CDCl_3$) spectra for compound **3ah**

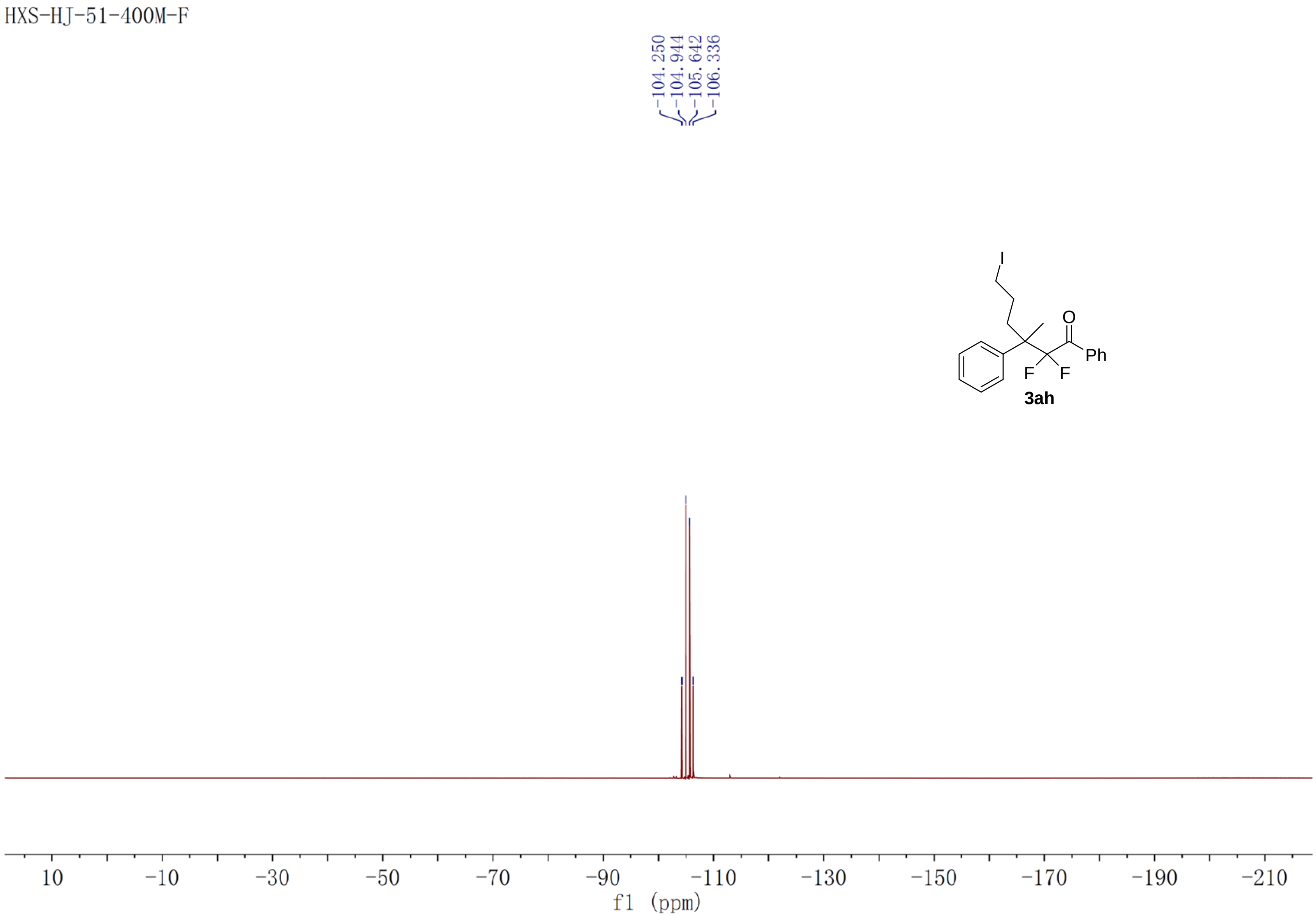

Supplementary Figure 124. ^{19}F NMR (376 MHz, $CDCl_3$) spectra for compound **3ah**

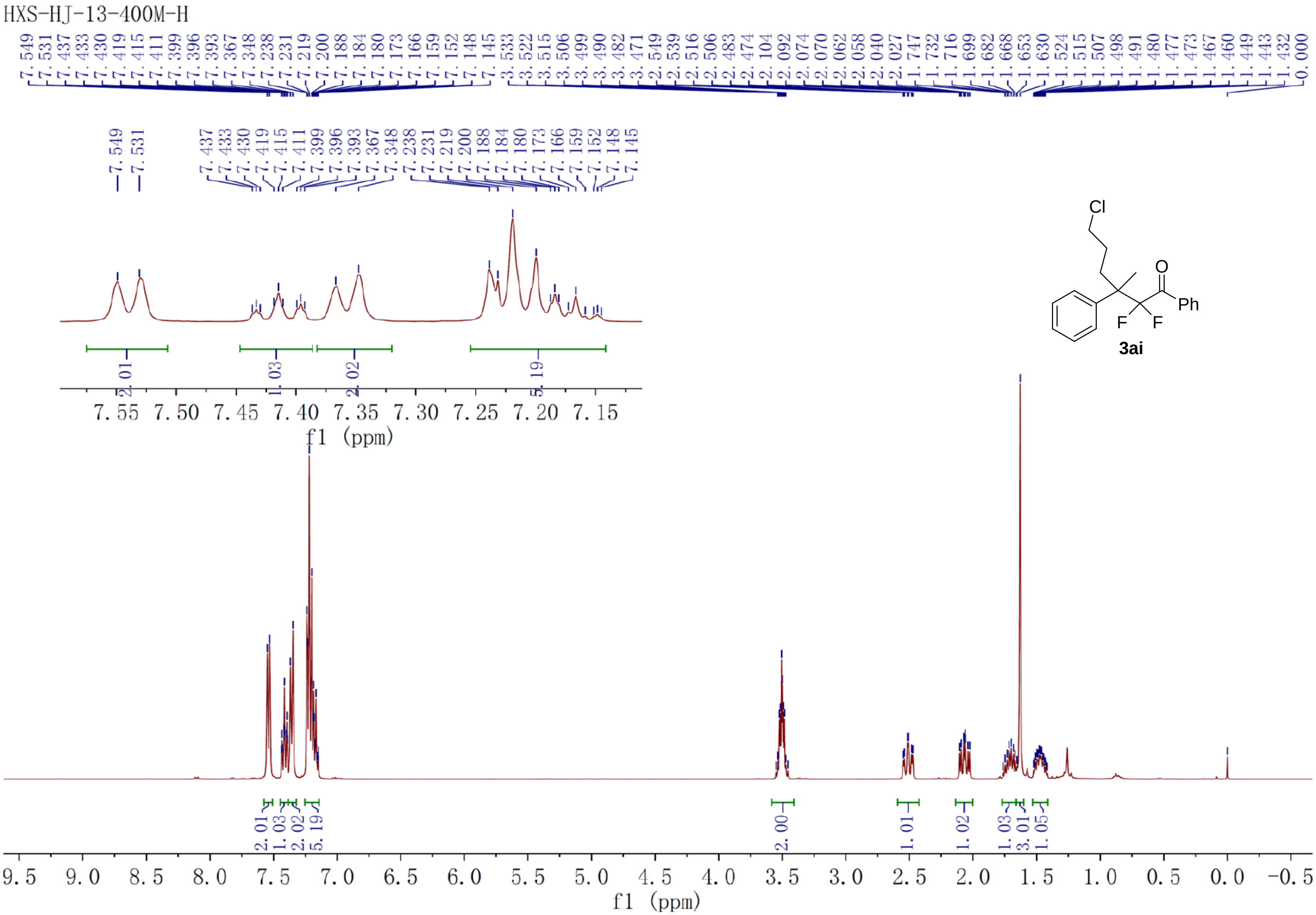

Supplementary Figure 125. ^{1}H NMR (400 MHz, $CDCl_3$) spectra for compound **3ai**

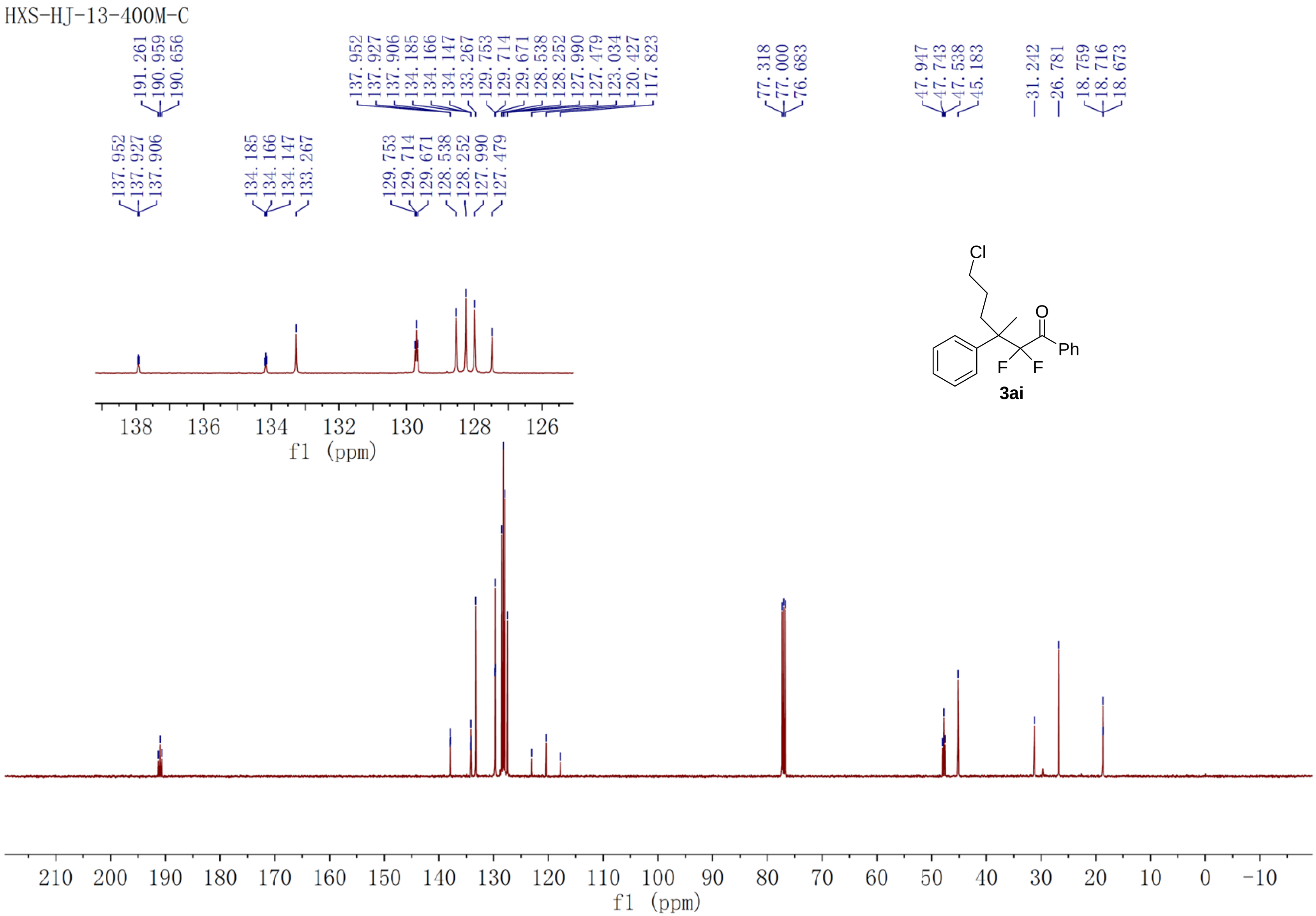

Supplementary Figure 126. ^{13}C NMR (100 MHz, $CDCl_3$) spectra for compound **3ai**

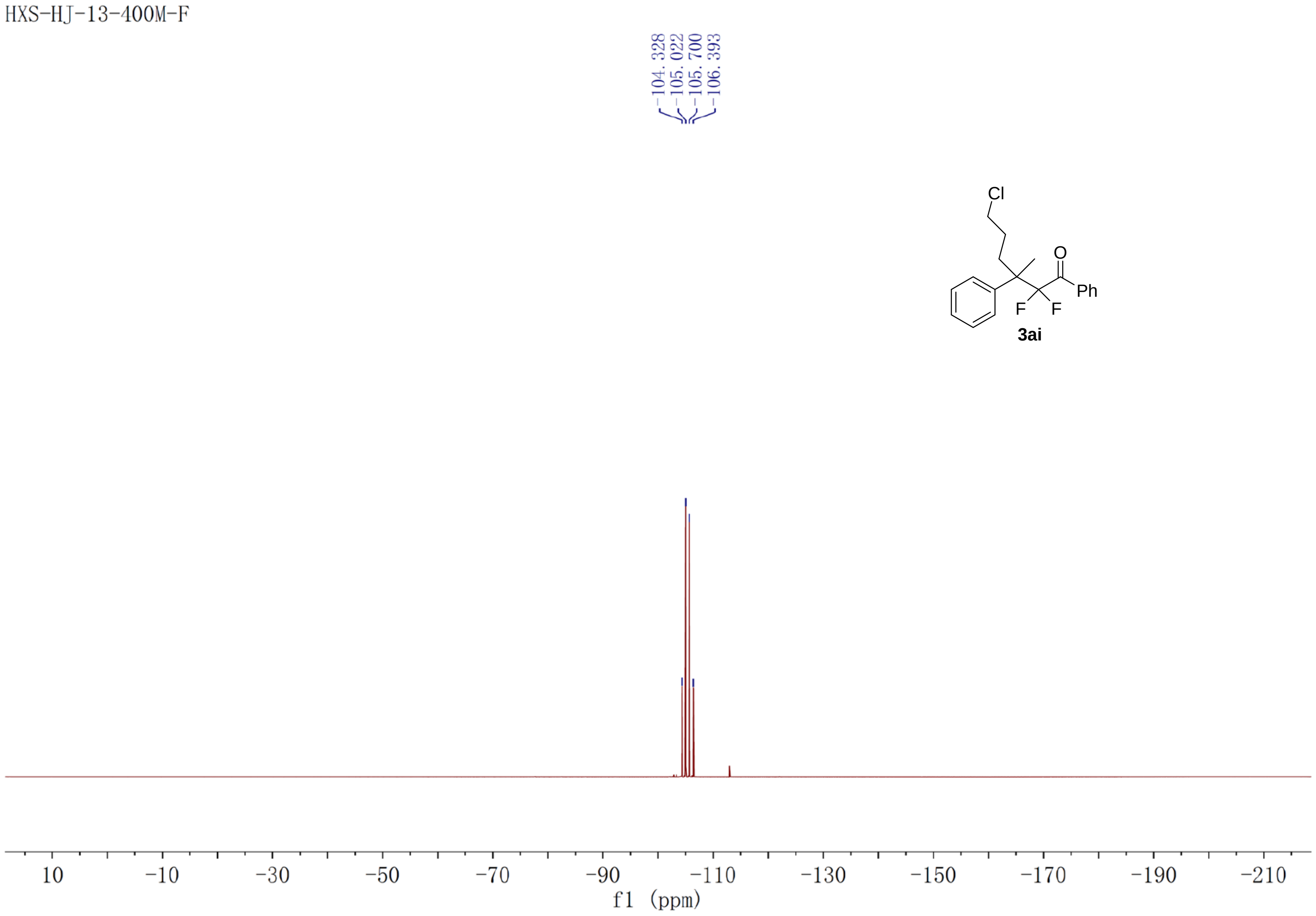

Supplementary Figure 127. ^{19}F NMR (376 MHz, $CDCl_3$) spectra for compound **3ai**

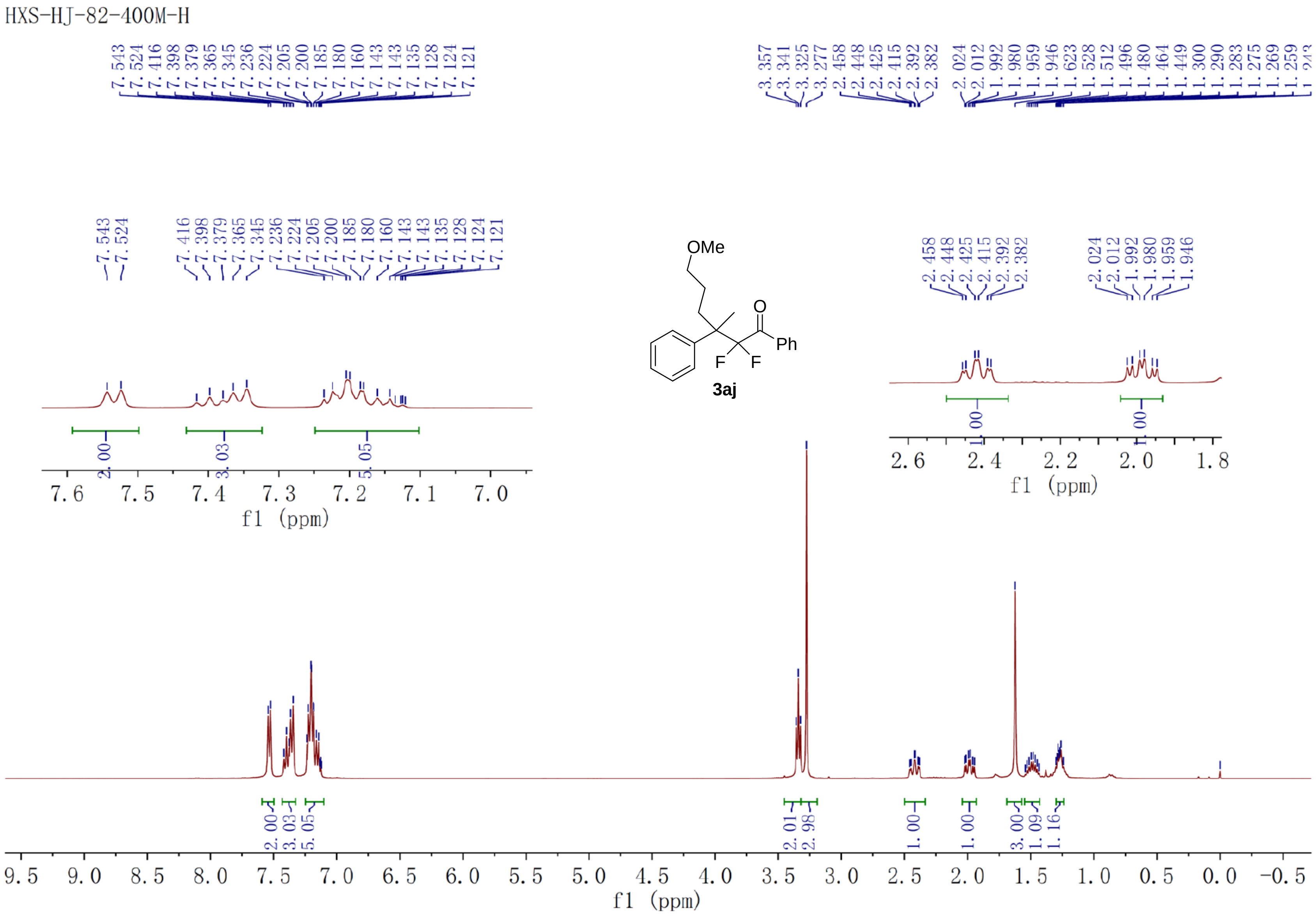

Supplementary Figure 128. ^{1}H NMR (400 MHz, $CDCl_3$) spectra for compound **3aj**

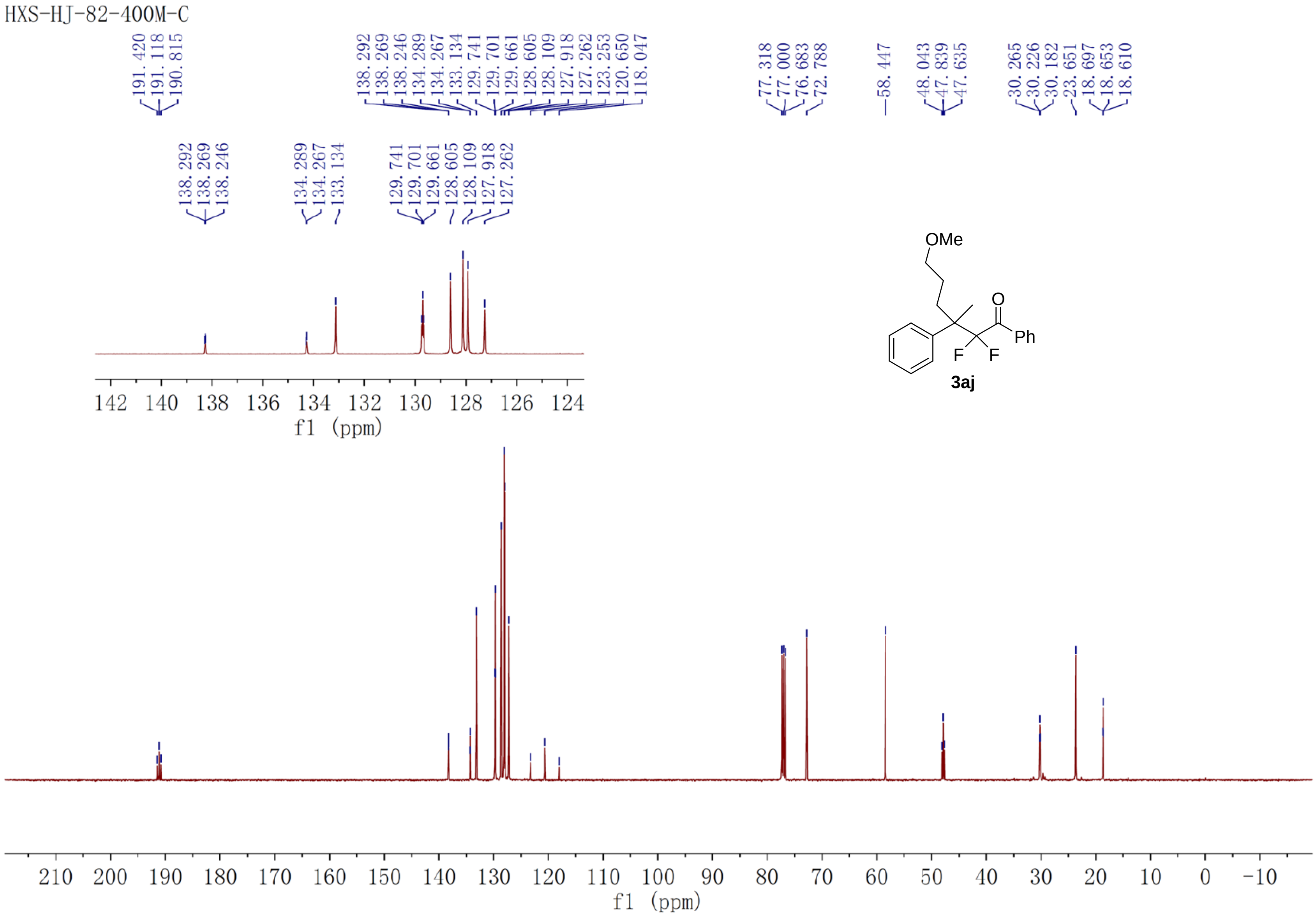

Supplementary Figure 129. ^{13}C NMR (100 MHz, $CDCl_3$) spectra for compound **3aj**

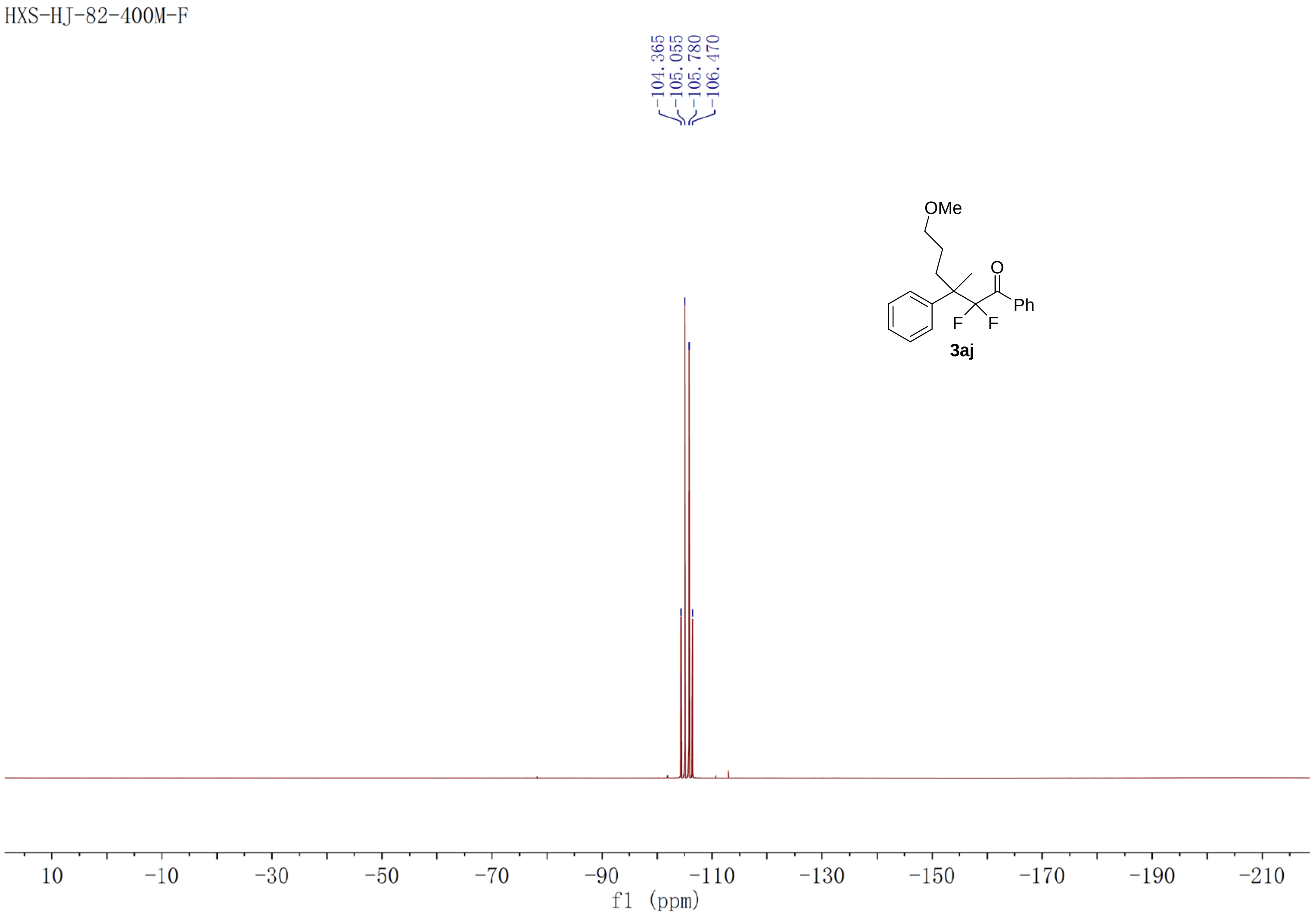

Supplementary Figure 130. ^{19}F NMR (376 MHz, $CDCl_3$) spectra for compound **3aj**

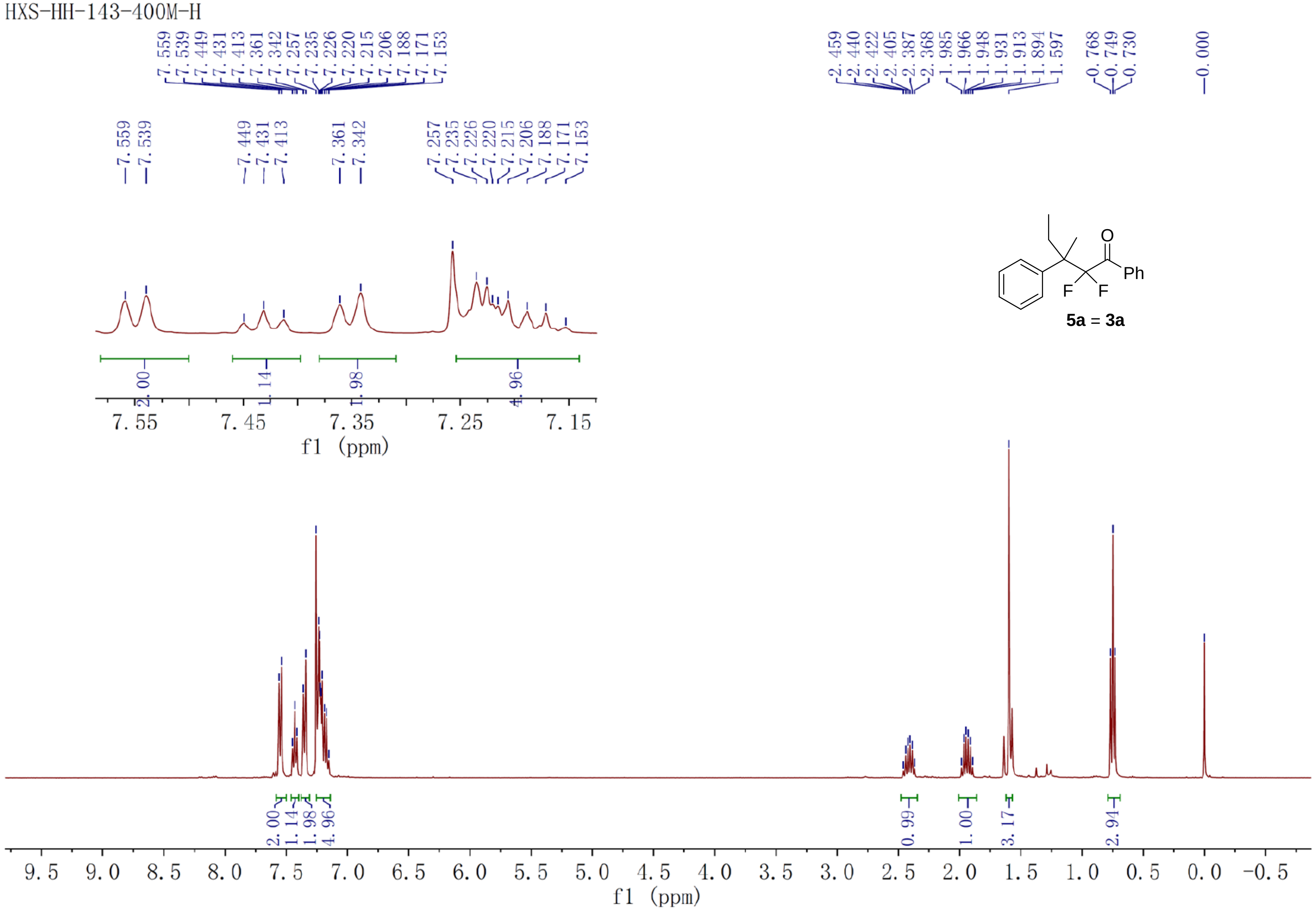

Supplementary Figure 131. ^{1}H NMR (400 MHz, $CDCl_3$) spectra for compound **5a**

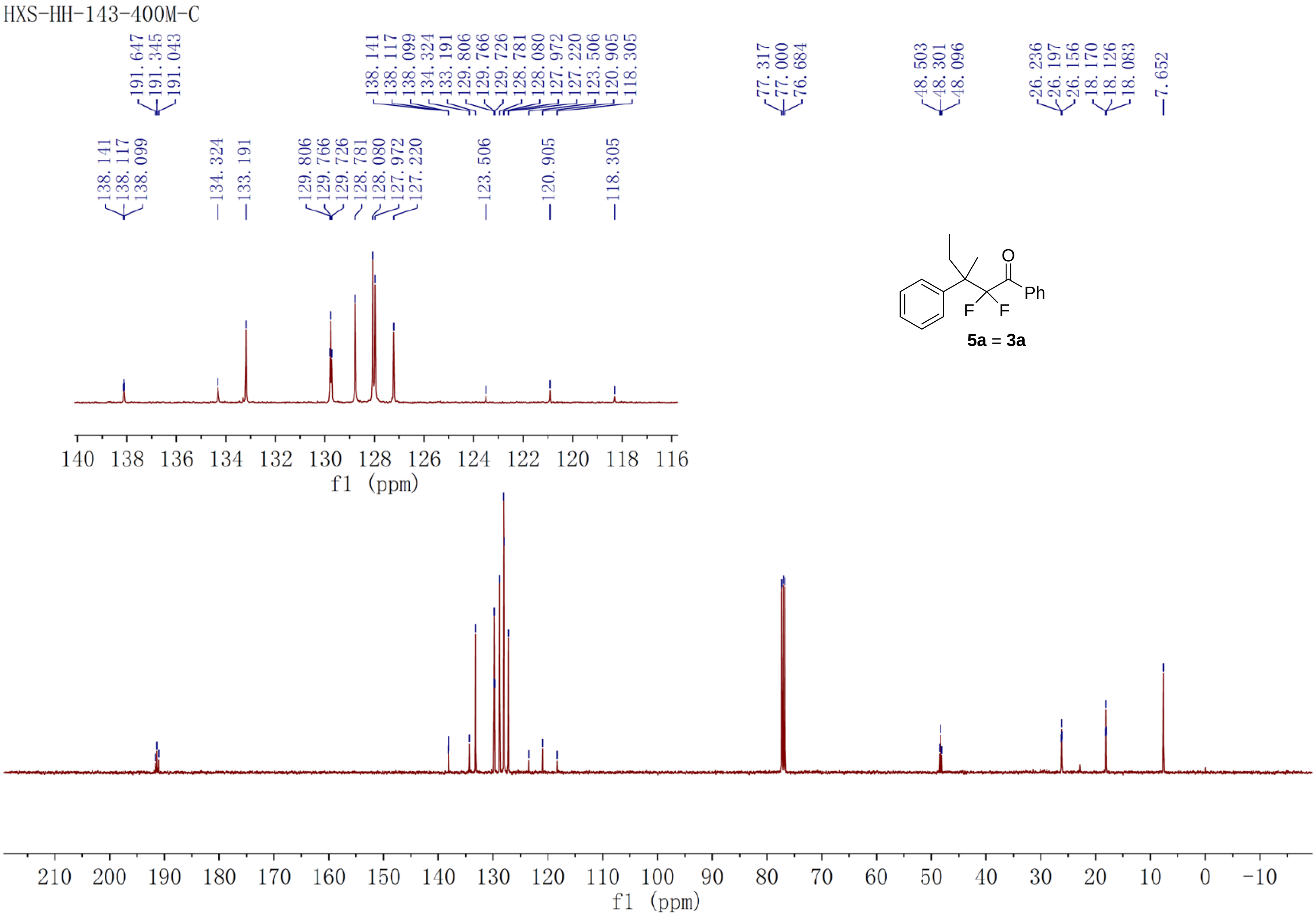

Supplementary Figure 132. ^{13}C NMR (100 MHz, $CDCl_3$) spectra for compound **5a**

HXS-HH-143-400M-F

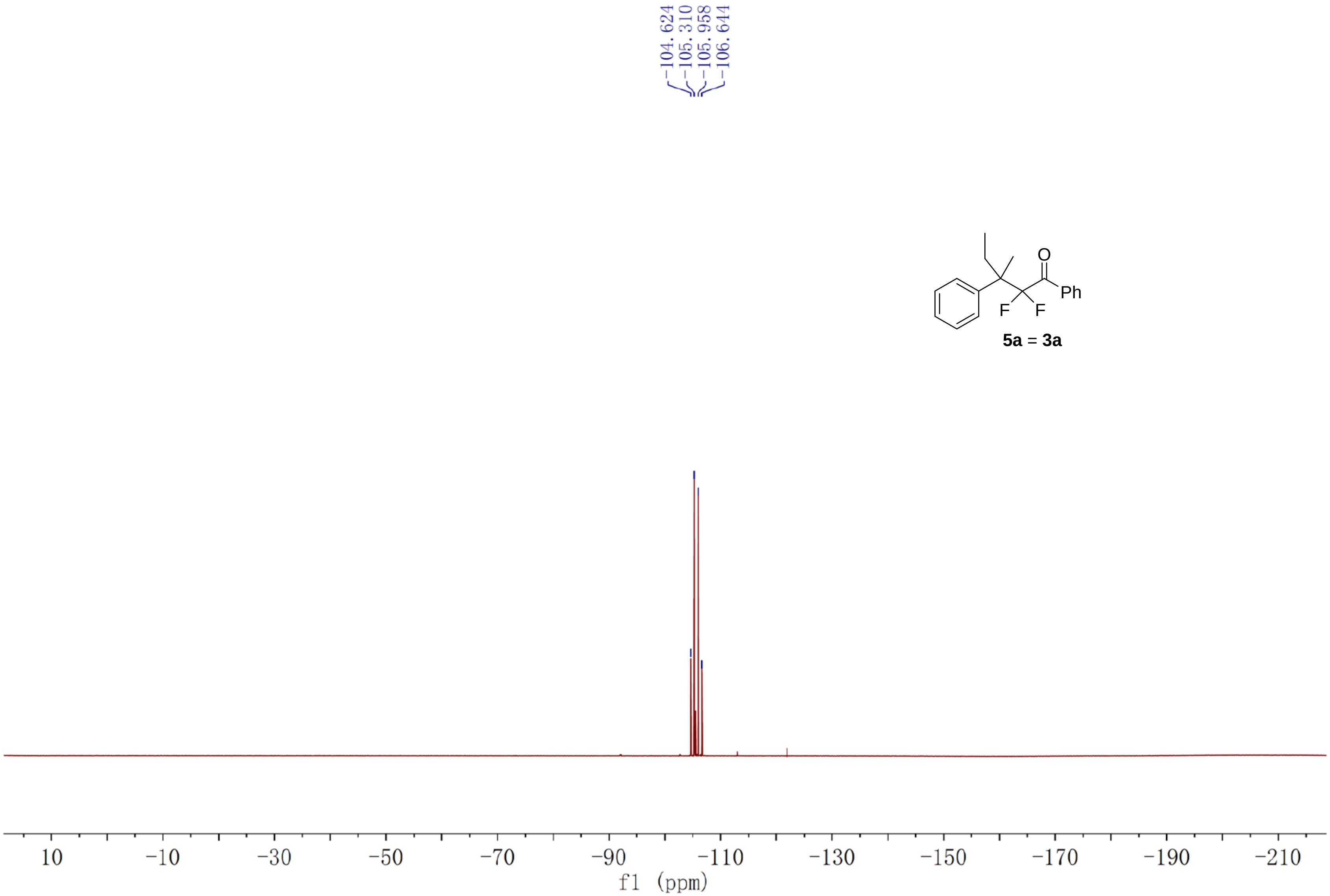

Supplementary Figure 133. ^{19}F NMR (376 MHz, $CDCl_3$) spectra for compound **5a**

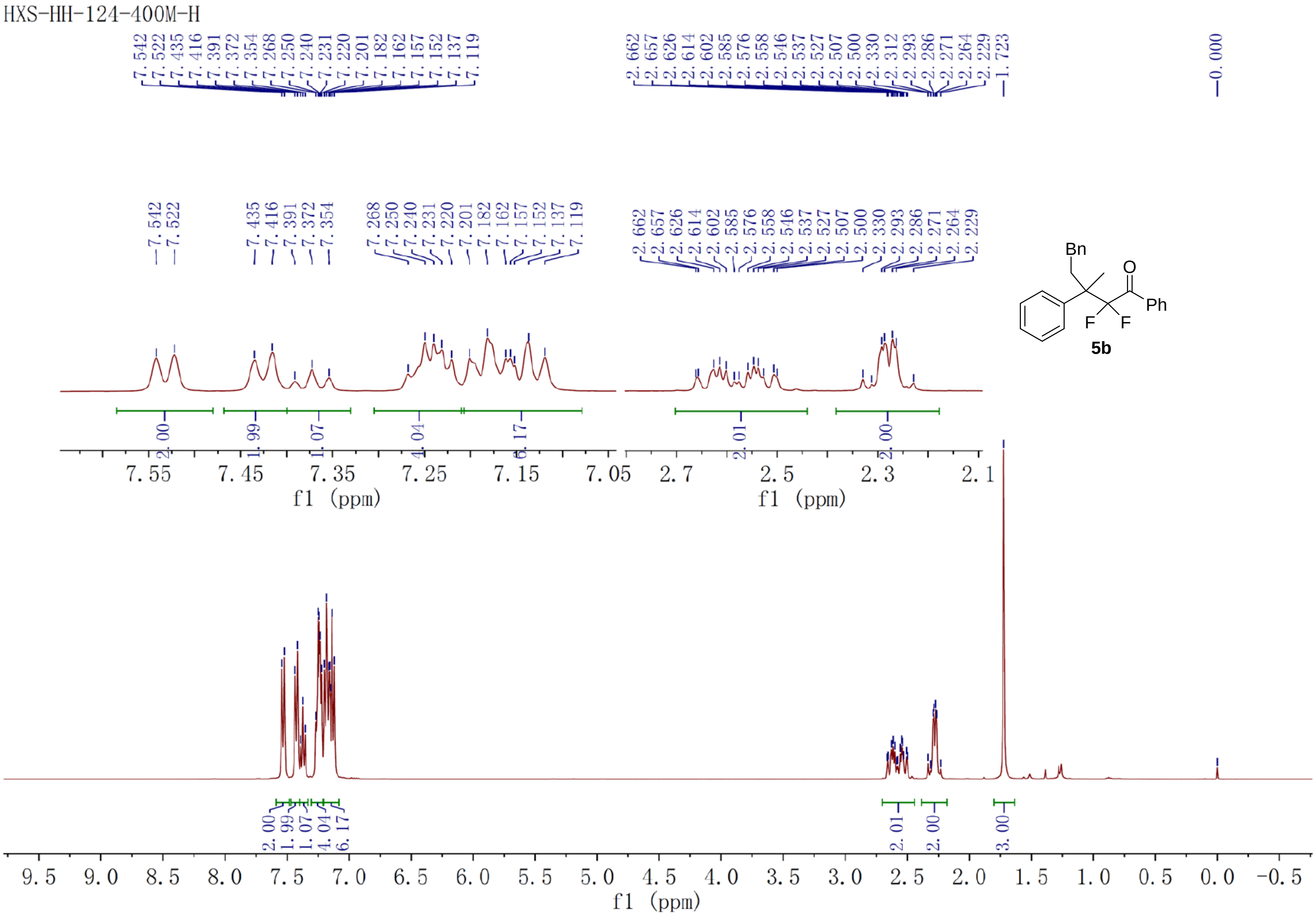

Supplementary Figure 134. ^{1}H NMR (400 MHz, $CDCl_3$) spectra for compound **5b**

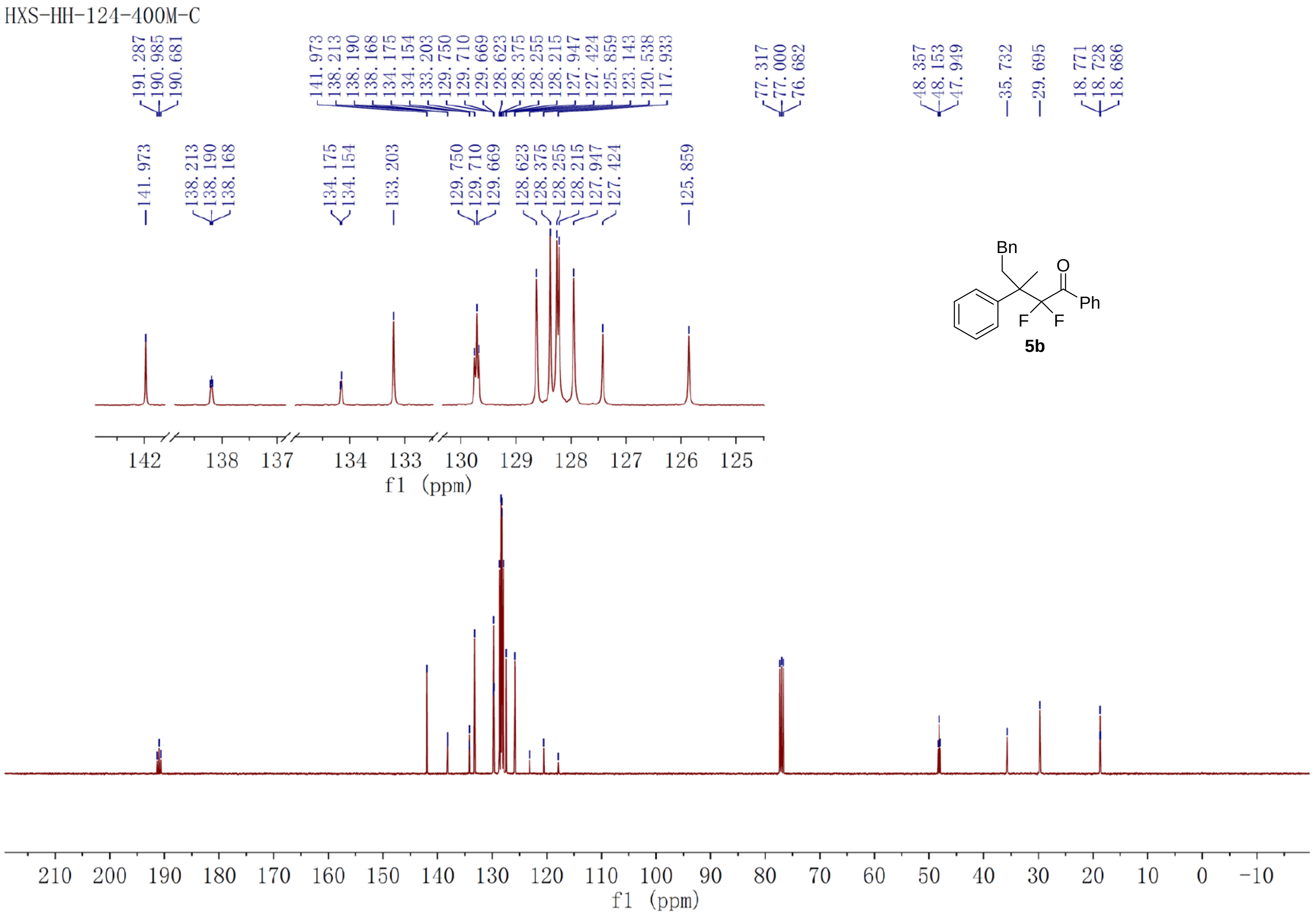

Supplementary Figure 135. ^{13}C NMR (100 MHz, $CDCl_3$) spectra for compound **5b**

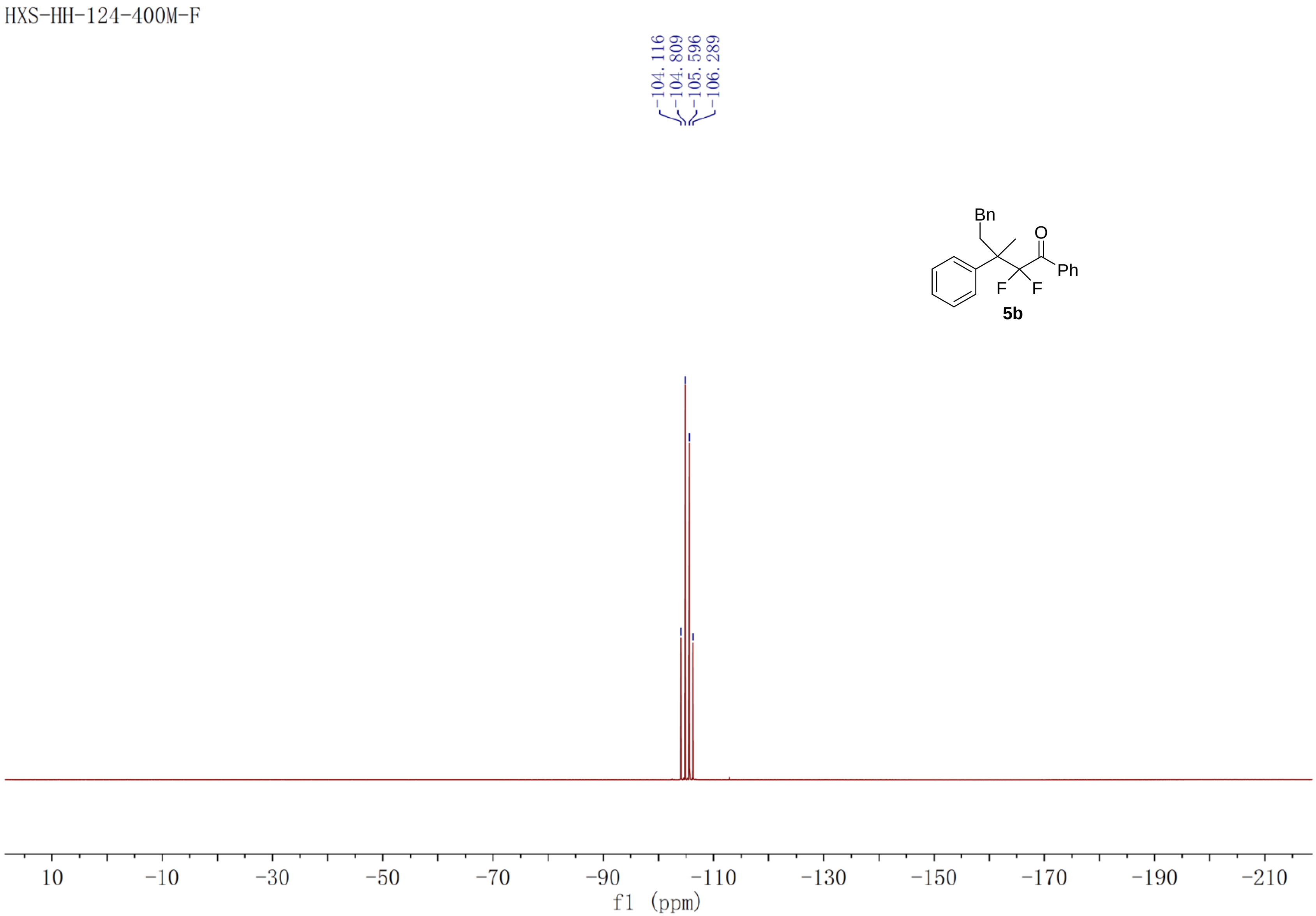

5b

Supplementary Figure 136. ^{19}F NMR (376 MHz, $CDCl_3$) spectra for compound **5b**

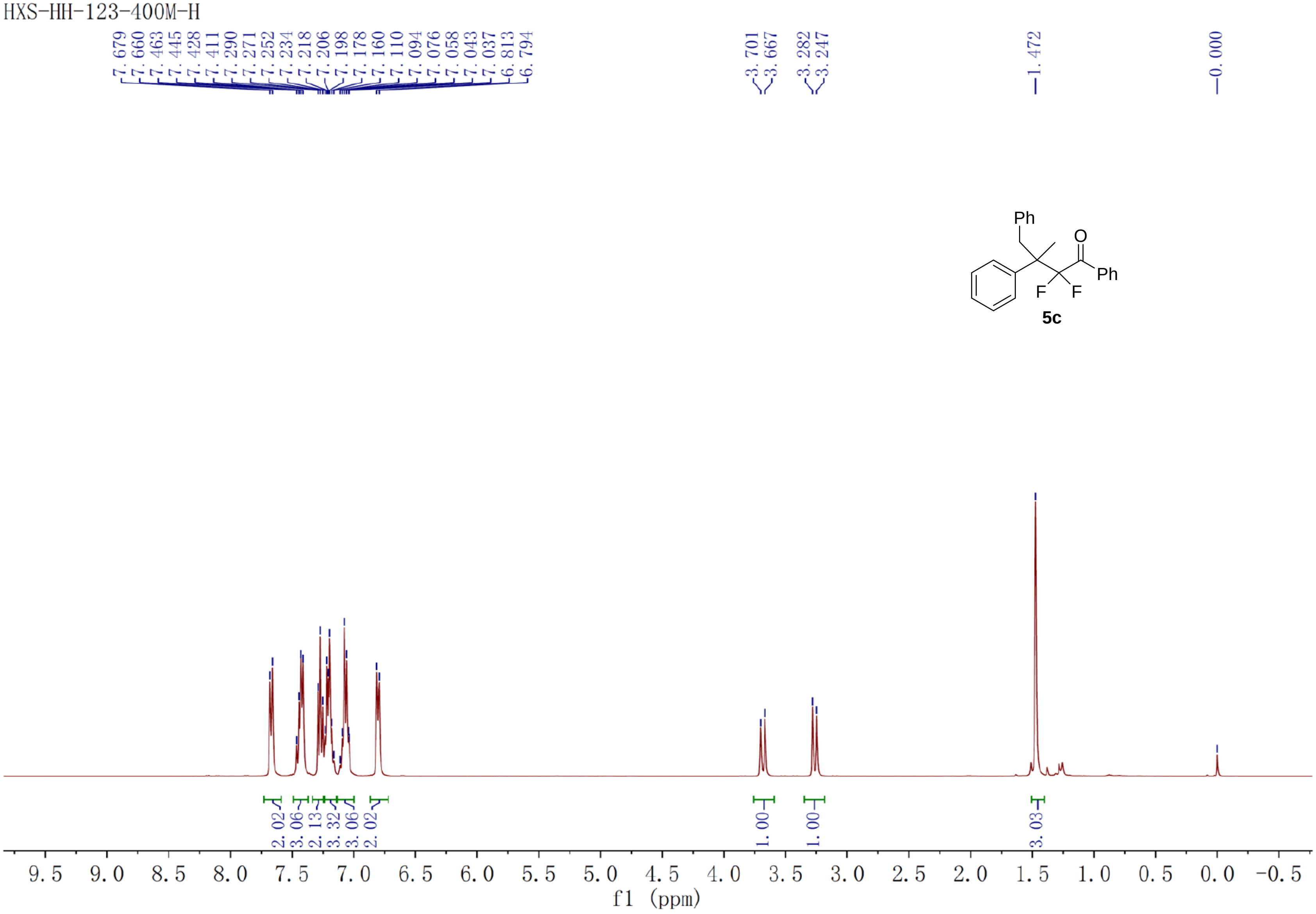

Supplementary Figure 137. ^{1}H NMR (400 MHz, $CDCl_3$) spectra for compound **5c**

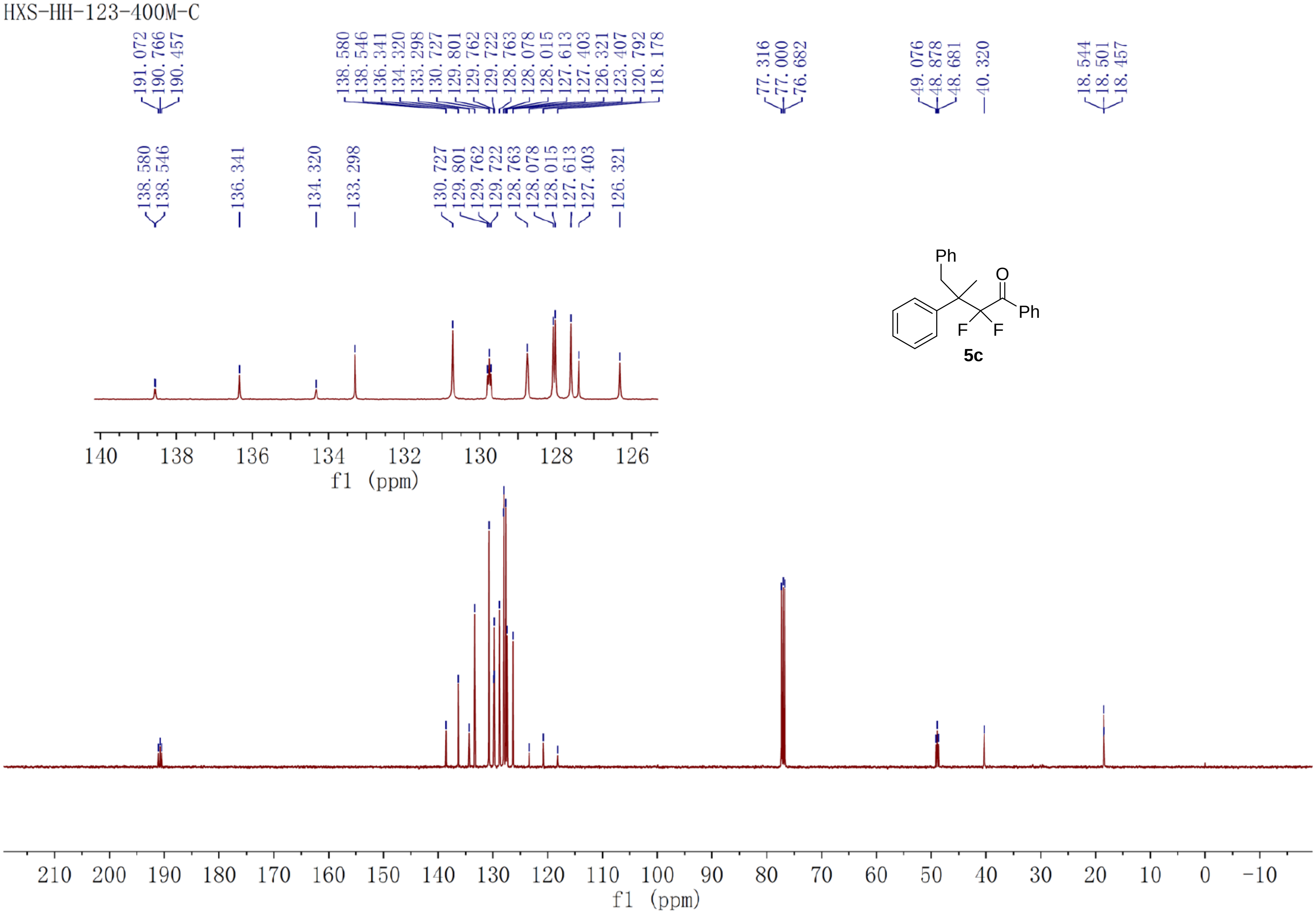

5c

Supplementary Figure 138. ^{13}C NMR (100 MHz, $CDCl_3$) spectra for compound **5c**

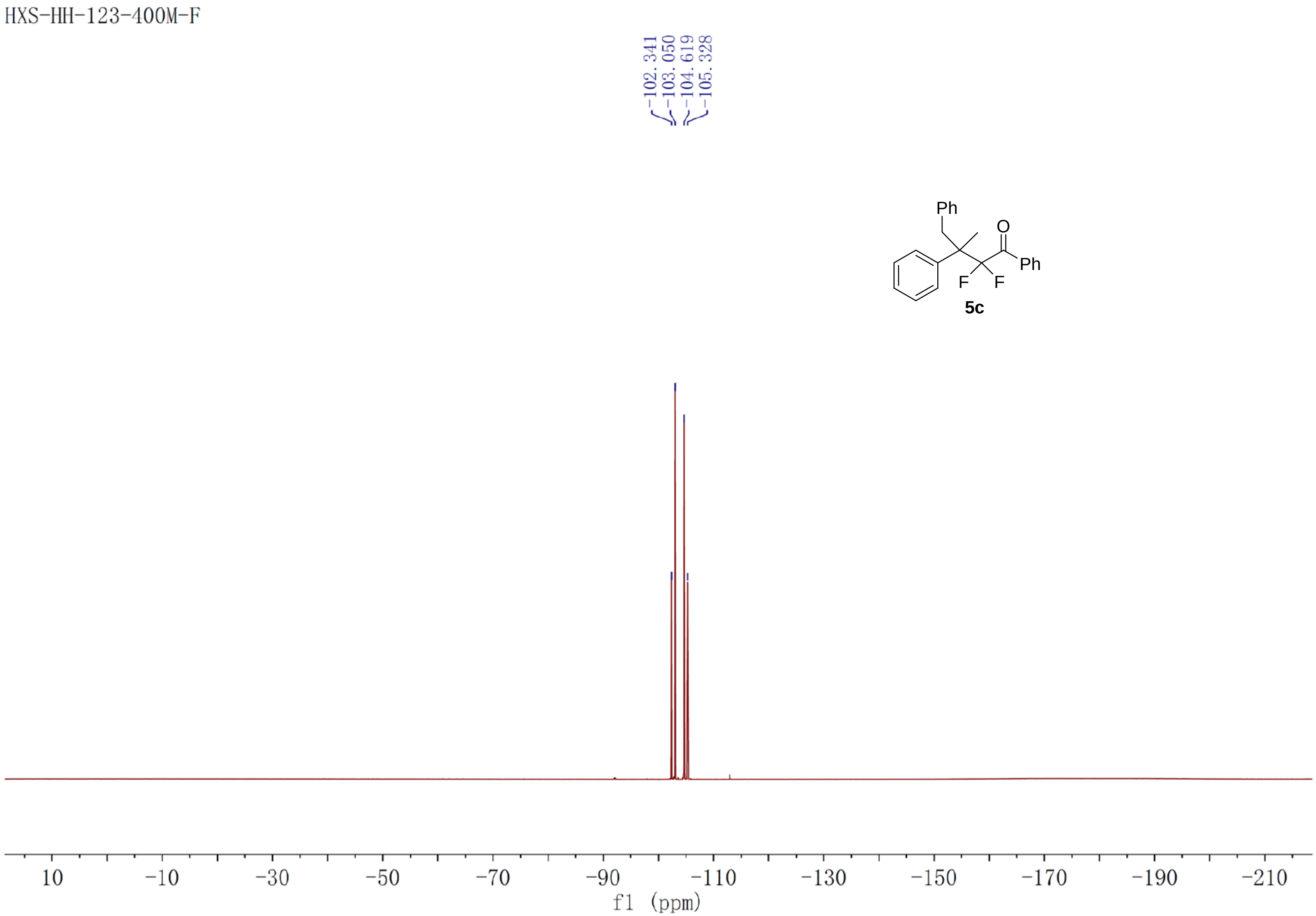

5c

Supplementary Figure 139. ^{19}F NMR (376 MHz, $CDCl_3$) spectra for compound **5c**

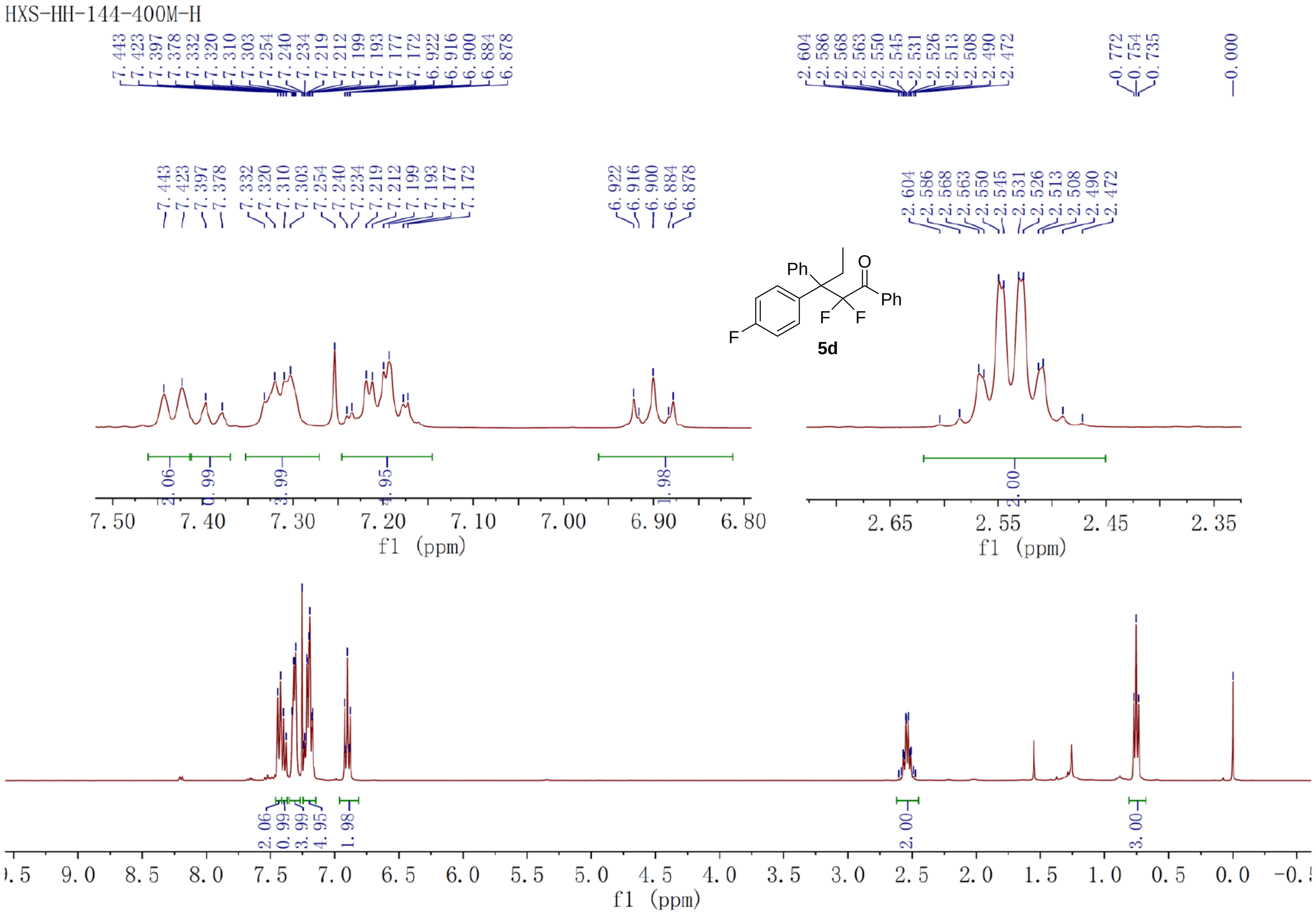

Supplementary Figure 140. ^{1}H NMR (400 MHz, $CDCl_3$) spectra for compound **5d**

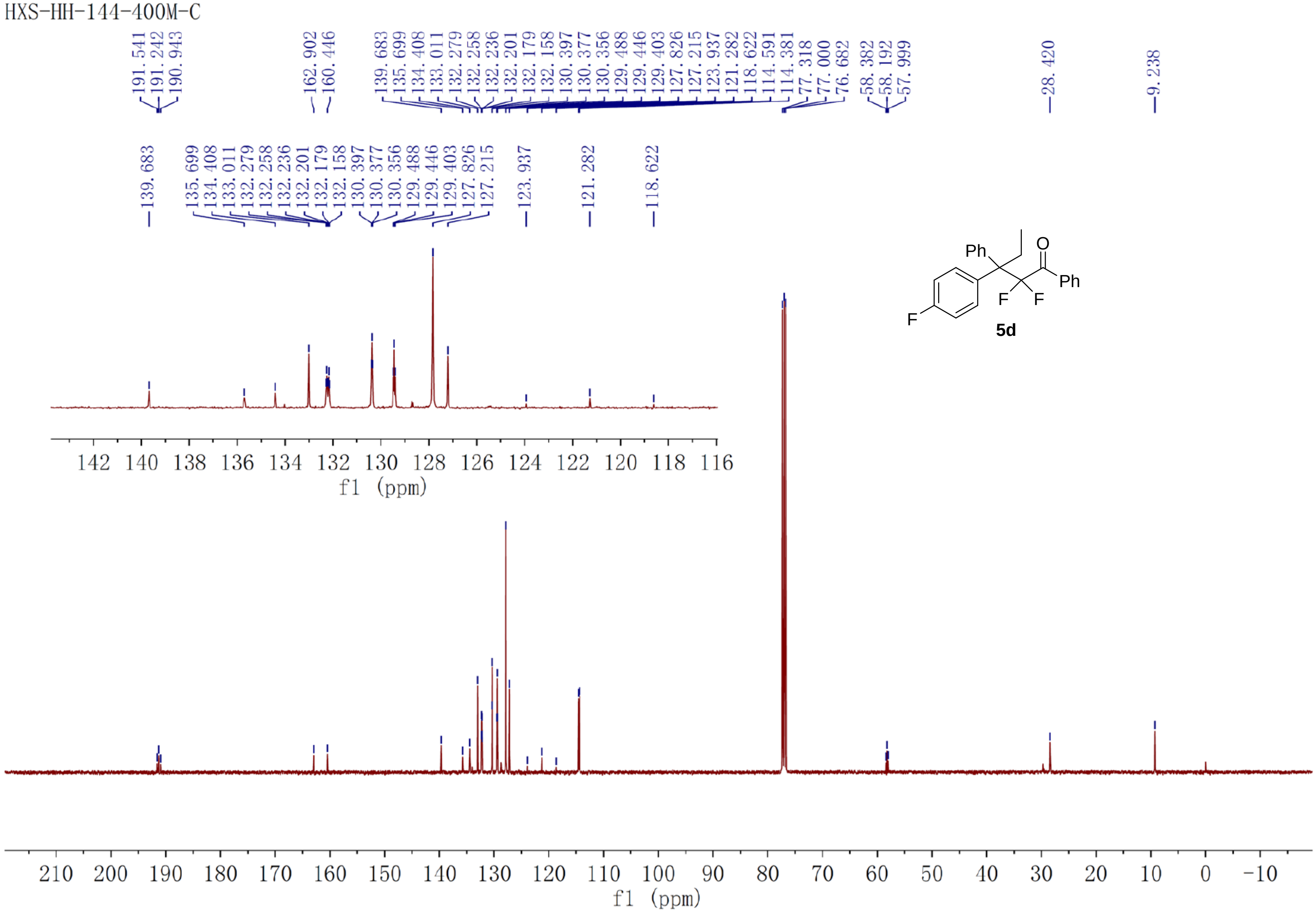

Supplementary Figure 141. ^{13}C NMR (100 MHz, $CDCl_3$) spectra for compound **5d**

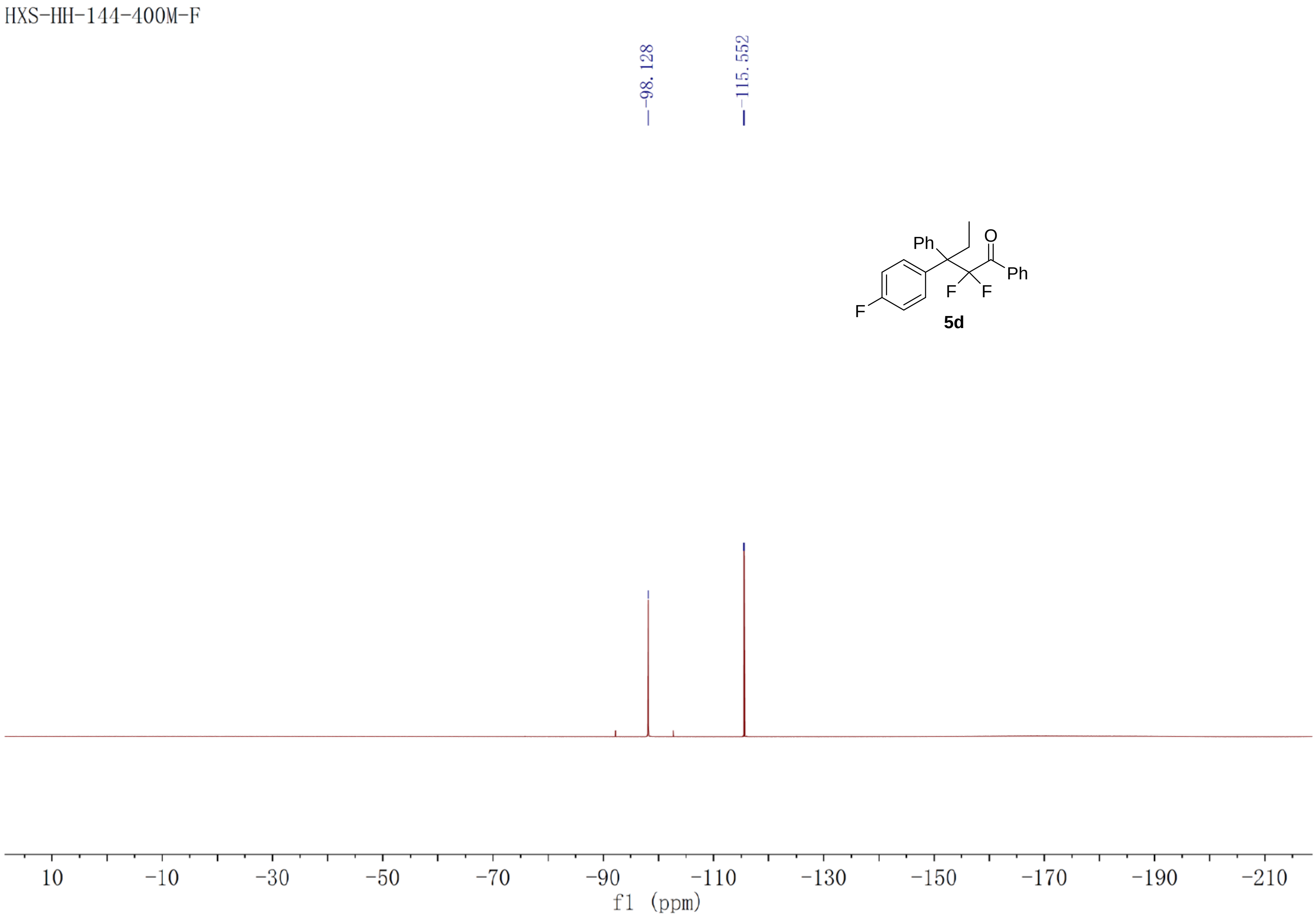

Supplementary Figure 142. ^{19}F NMR (376 MHz, $CDCl_3$) spectra for compound **5d**

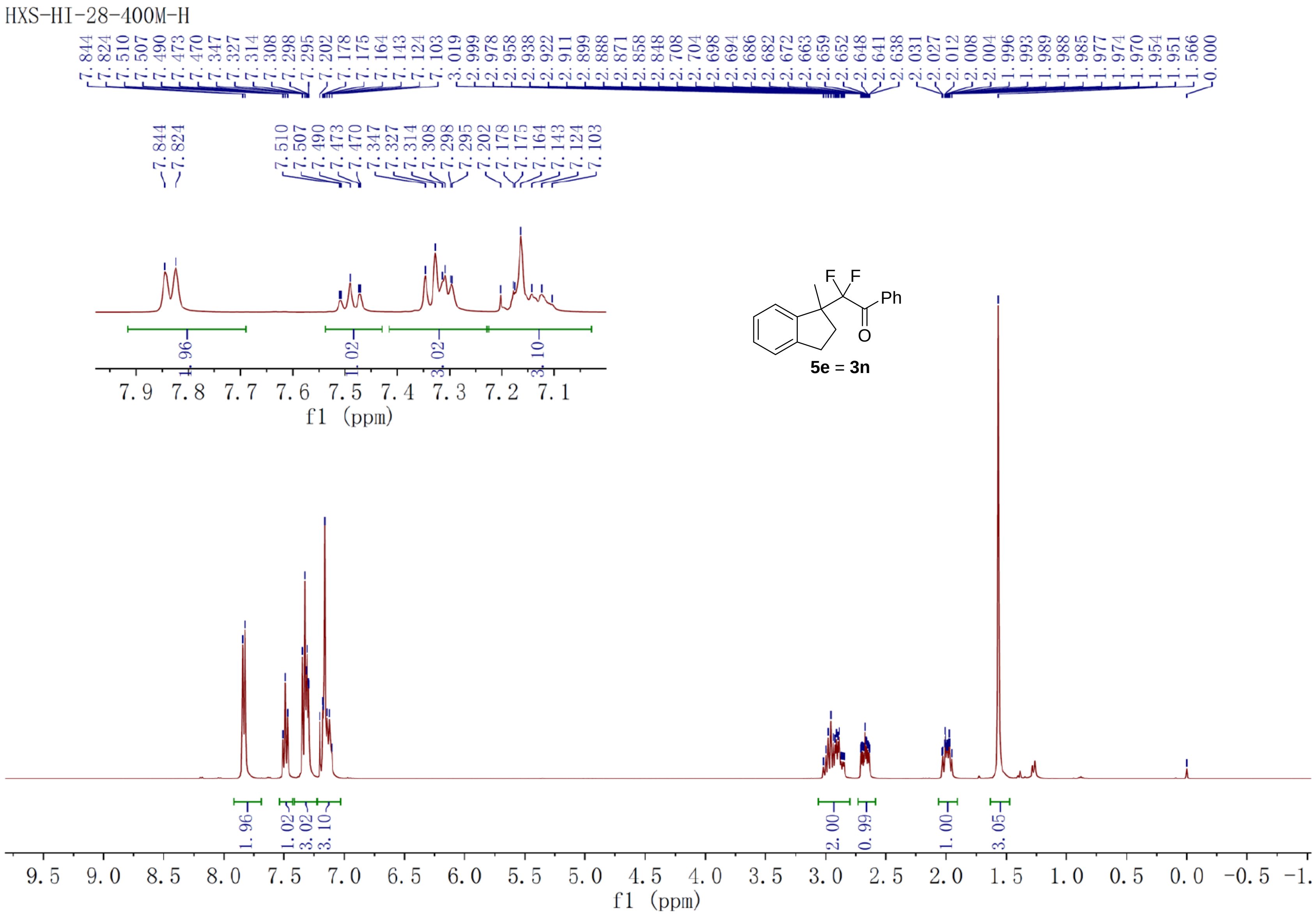

Supplementary Figure 143. ^{1}H NMR (400 MHz, $CDCl_3$) spectra for compound **5e**

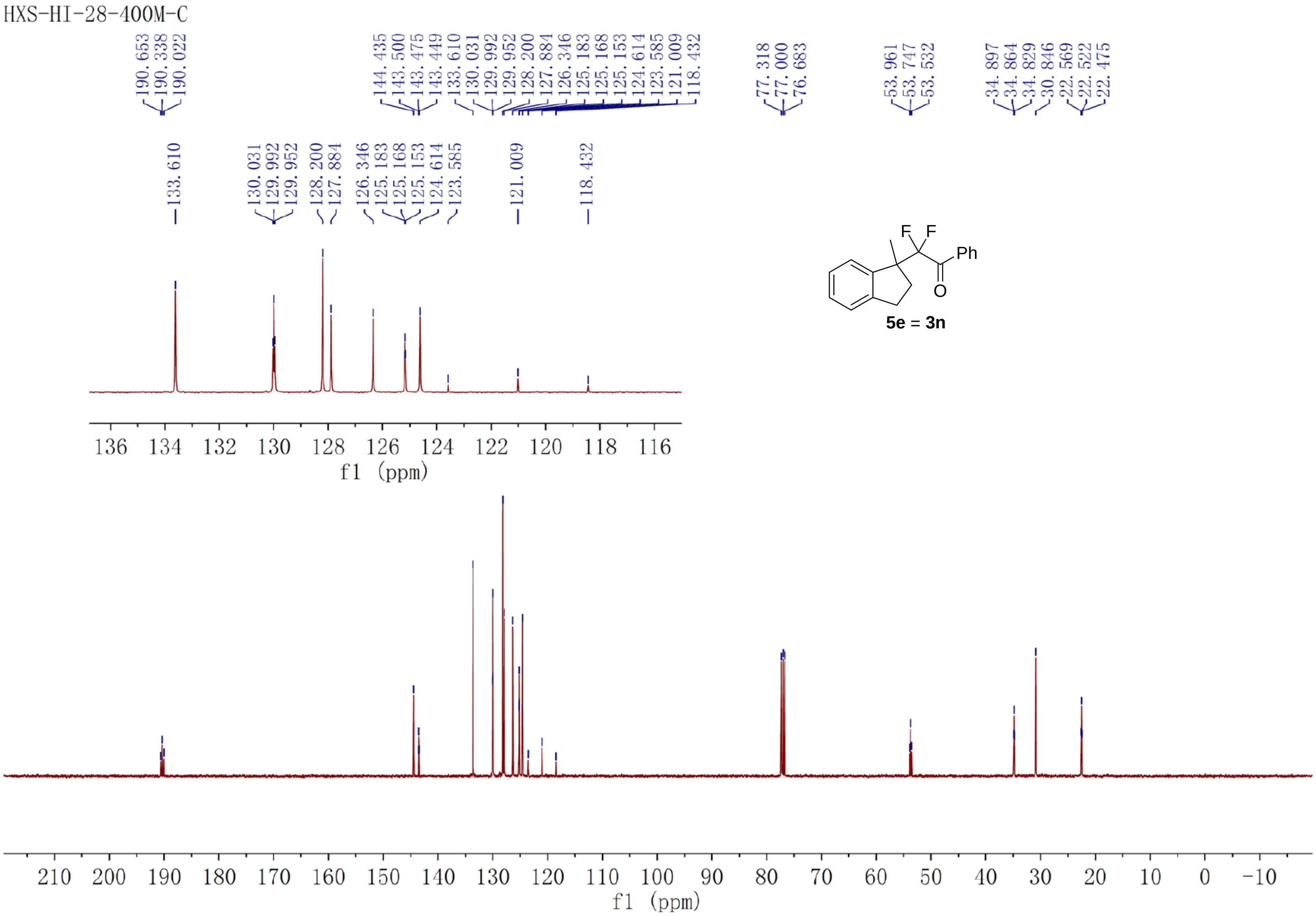

5e = 3n

Supplementary Figure 144. ^{13}C NMR (100 MHz, $CDCl_3$) spectra for compound **5e**

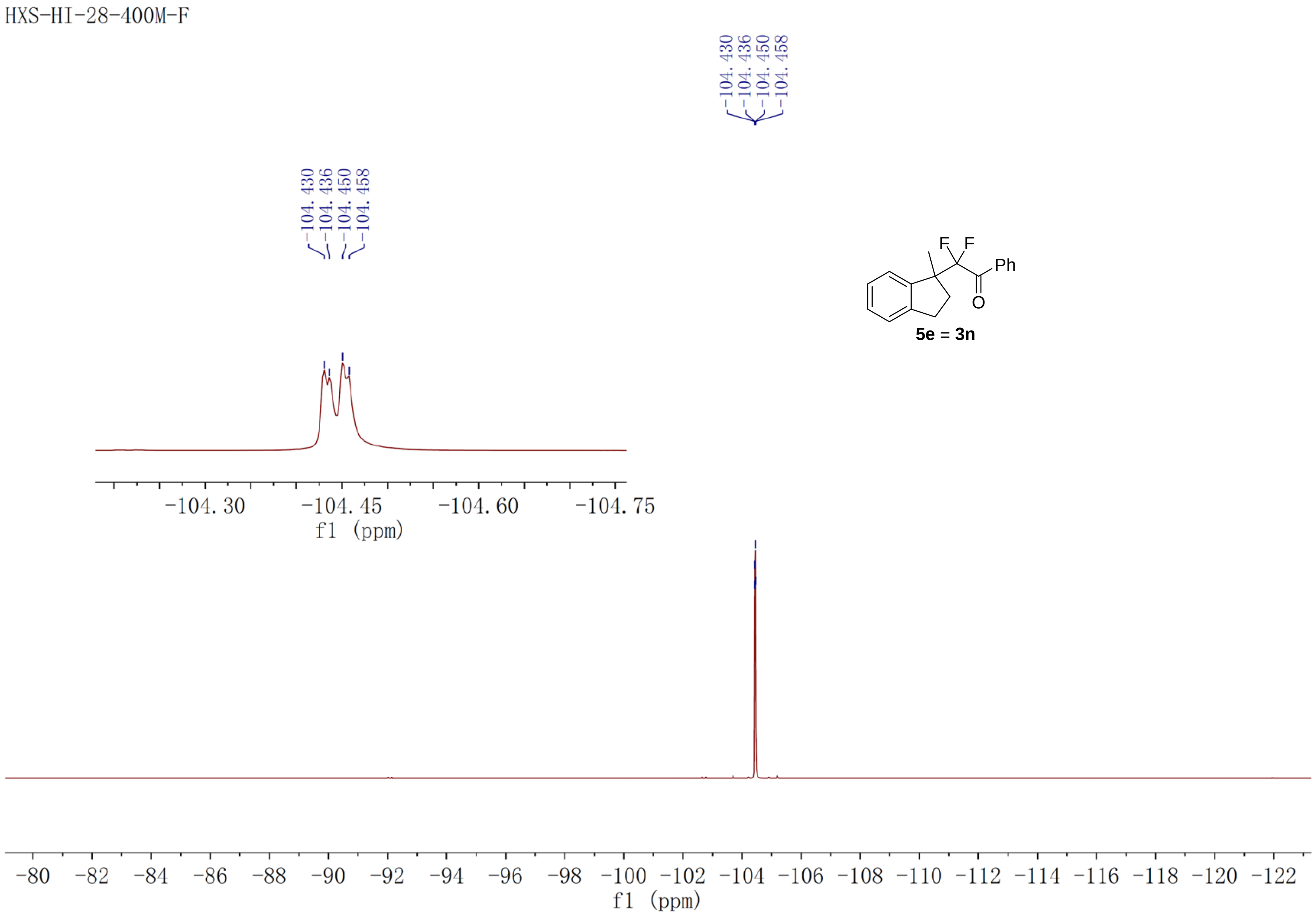

Supplementary Figure 145. ^{19}F NMR (376 MHz, $CDCl_3$) spectra for compound **5e**

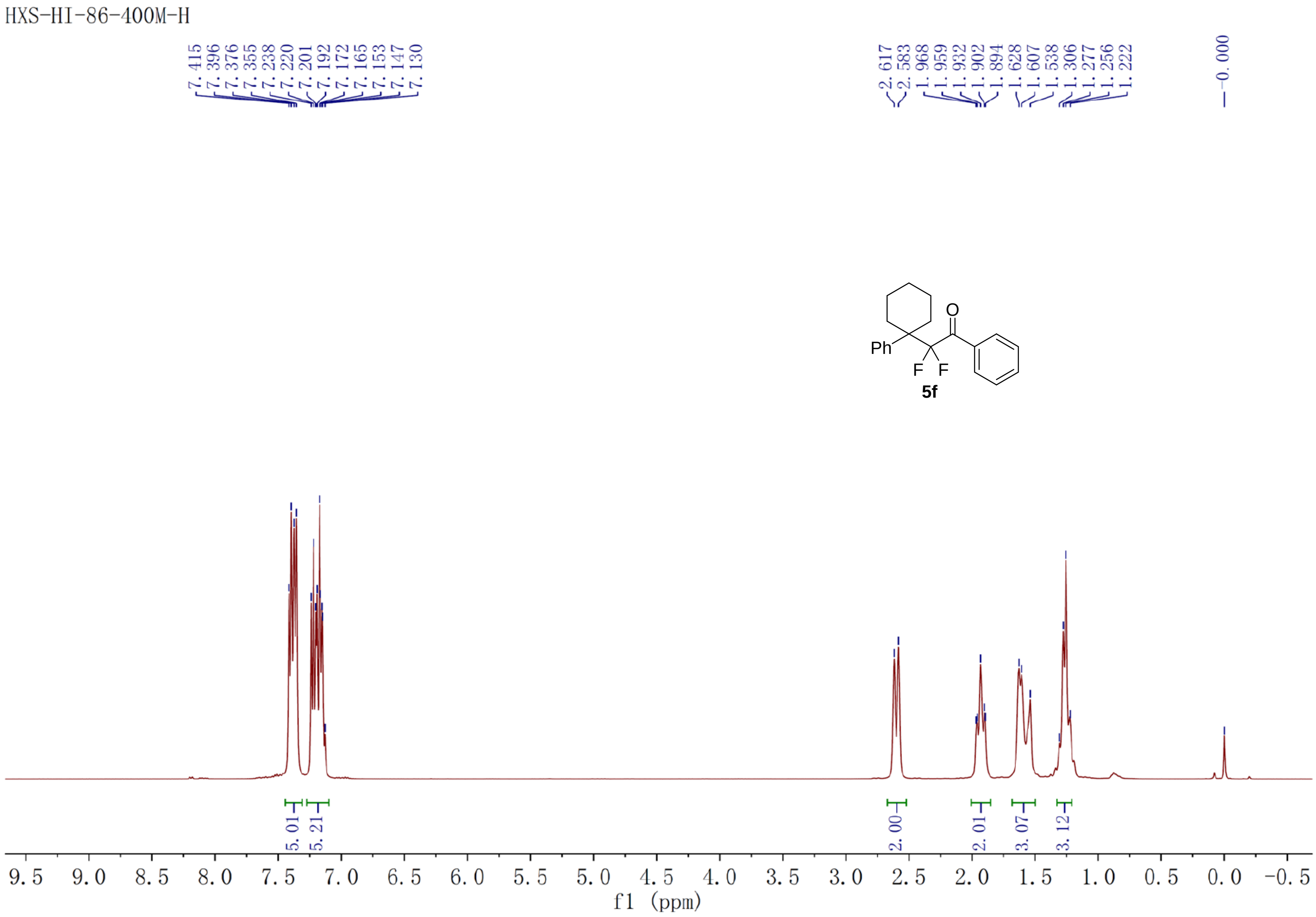

Supplementary Figure 146. ^{1}H NMR (400 MHz, $CDCl_3$) spectra for compound **5f**

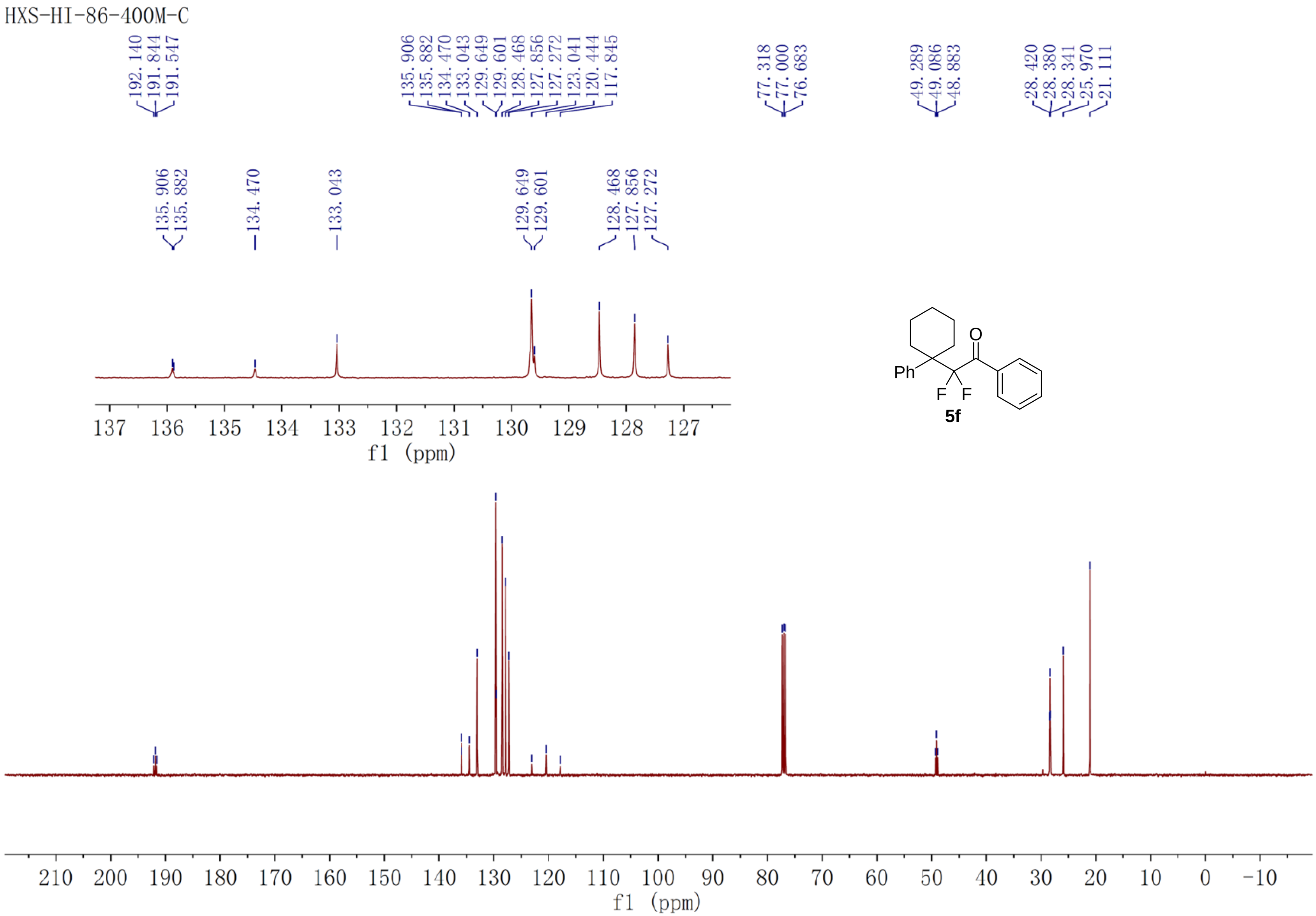

Supplementary Figure 147. ^{13}C NMR (100 MHz, $CDCl_3$) spectra for compound **5f**

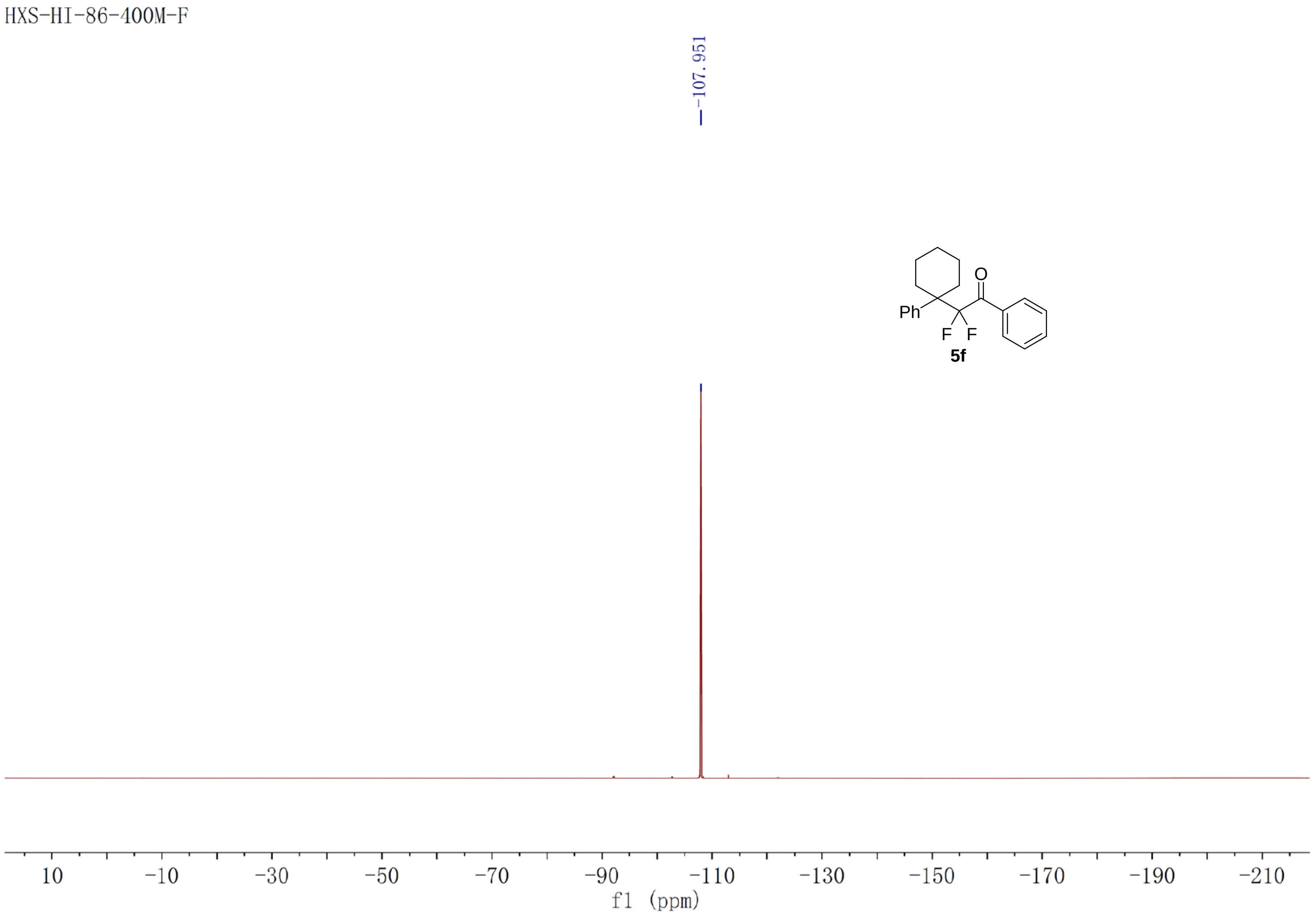

Supplementary Figure 148. ^{19}F NMR (376 MHz, $CDCl_3$) spectra for compound **5f**

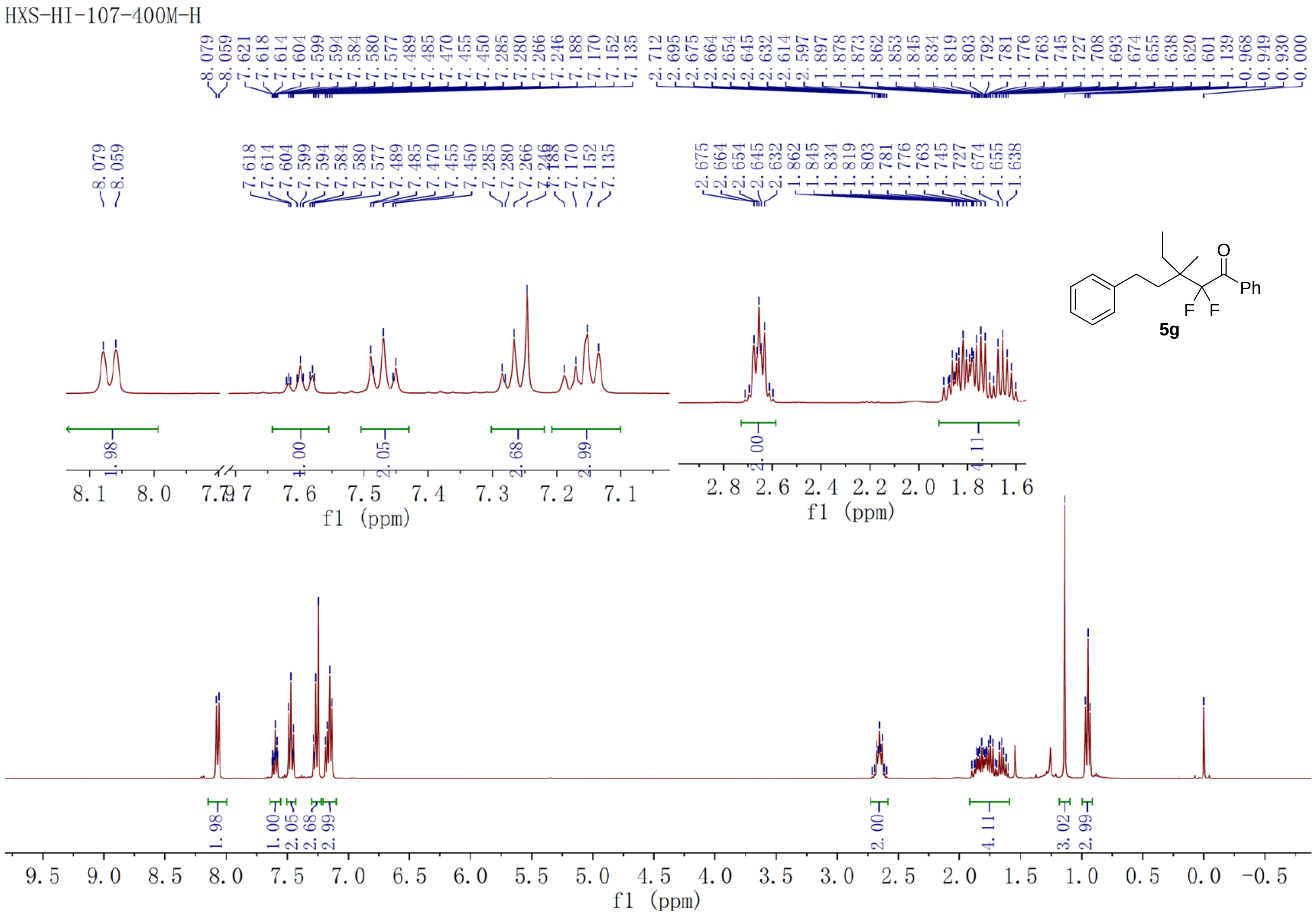

Supplementary Figure 149. ^{1}H NMR (400 MHz, $CDCl_3$) spectra for compound **5g**

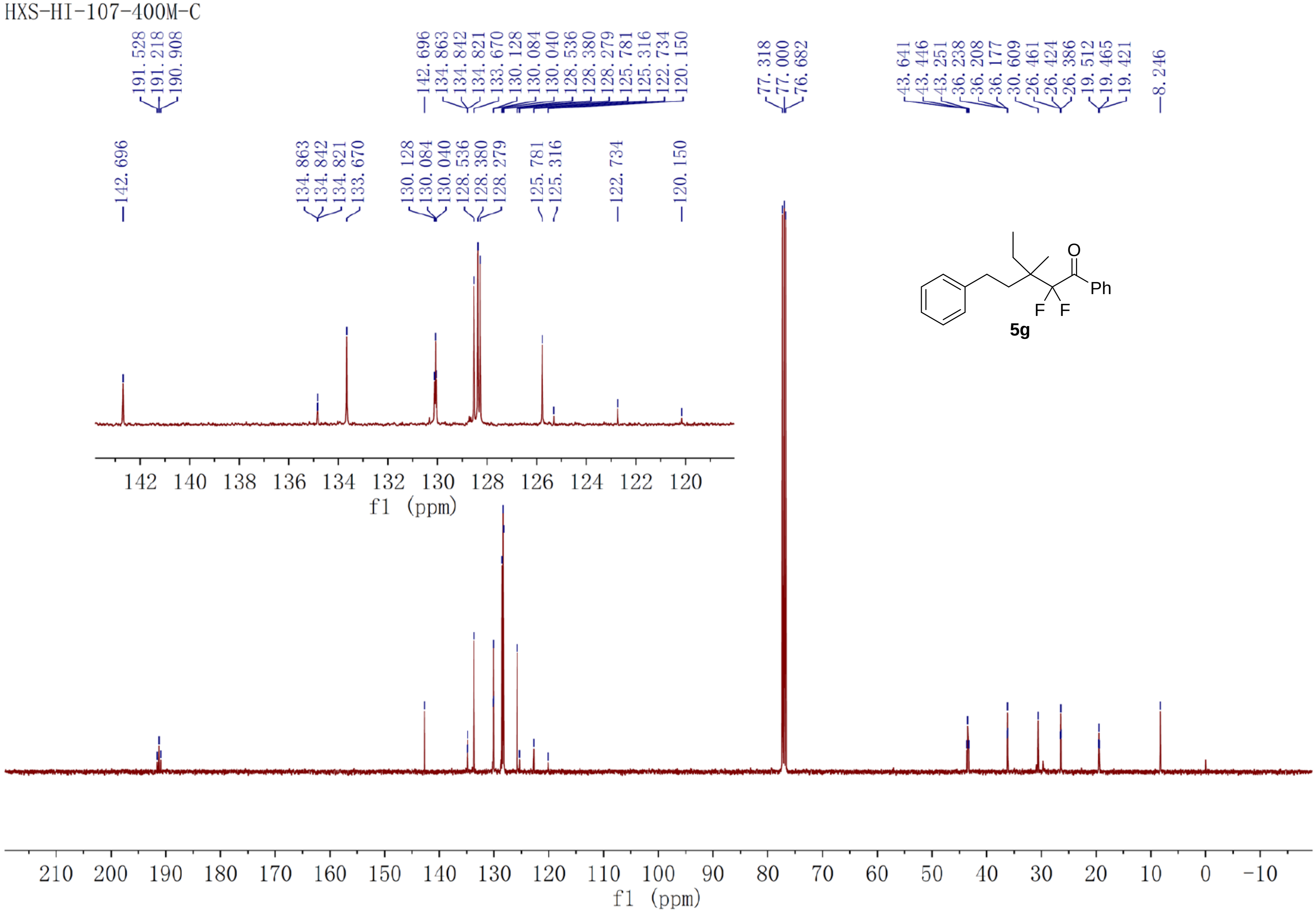

Supplementary Figure 150. ^{13}C NMR (100 MHz, $CDCl_3$) spectra for compound **5g**

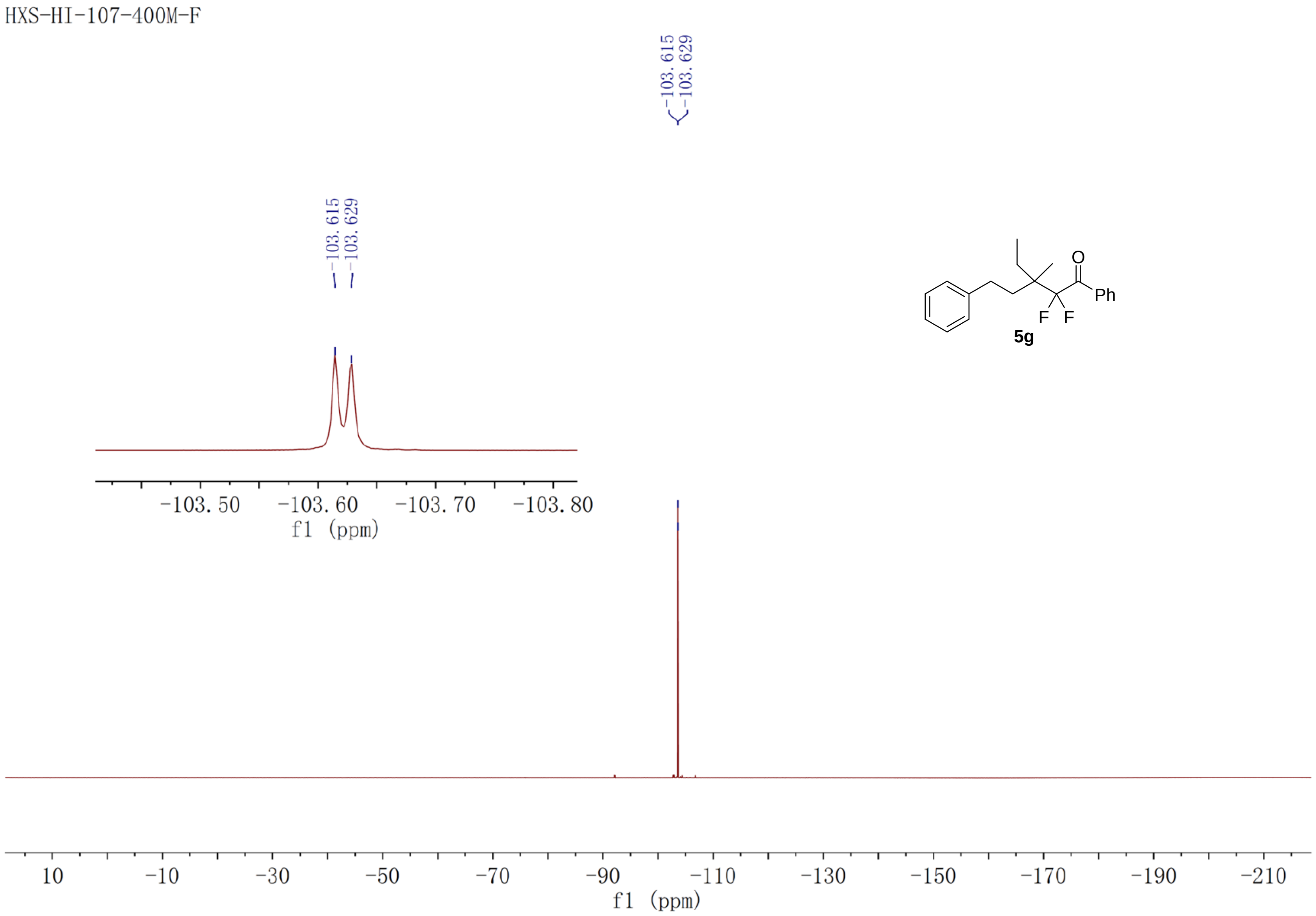

5g

Supplementary Figure 151. ^{19}F NMR (376 MHz, $CDCl_3$) spectra for compound **5g**

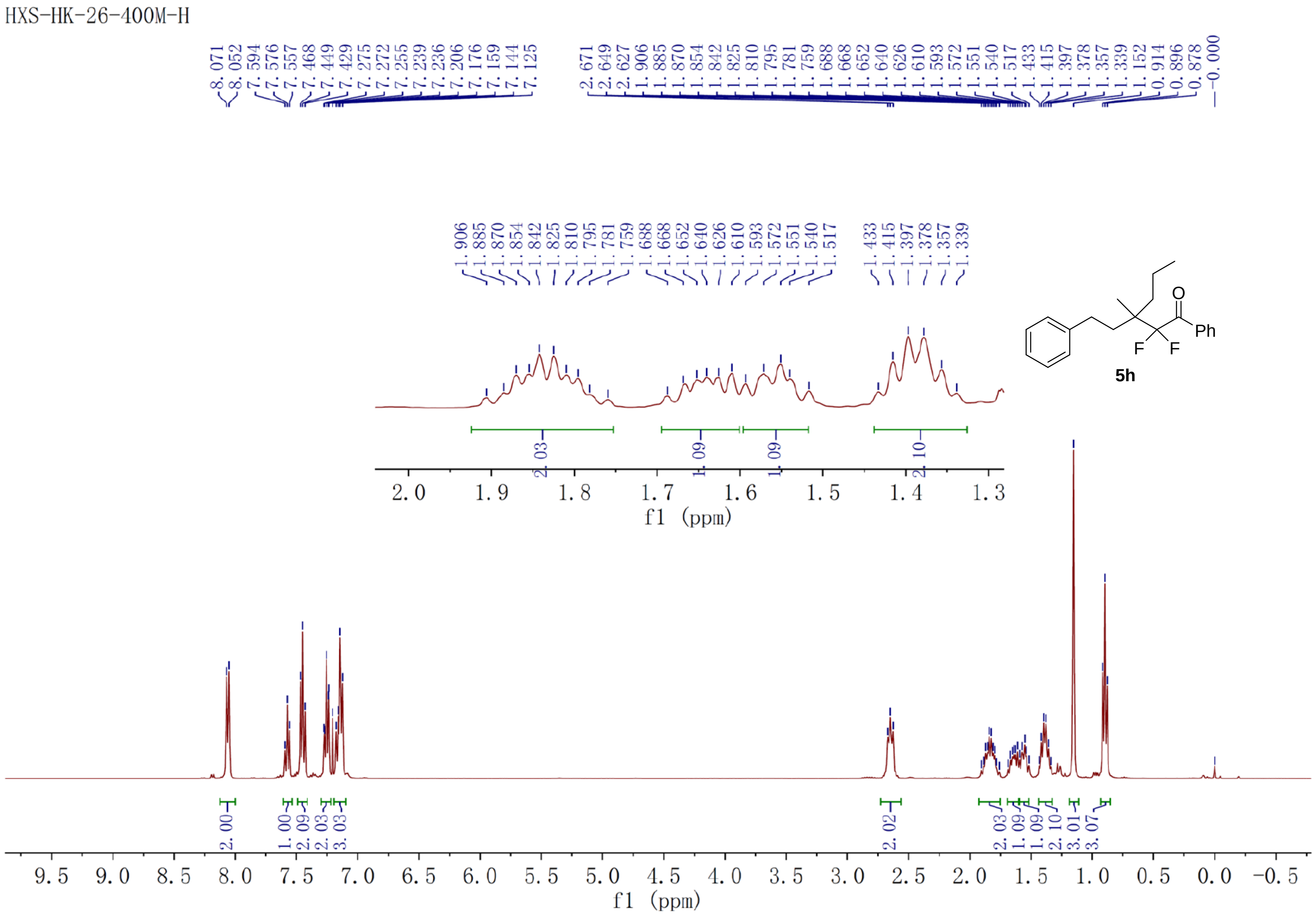

Supplementary Figure 152. ^{1}H NMR (400 MHz, $CDCl_3$) spectra for compound **5h**

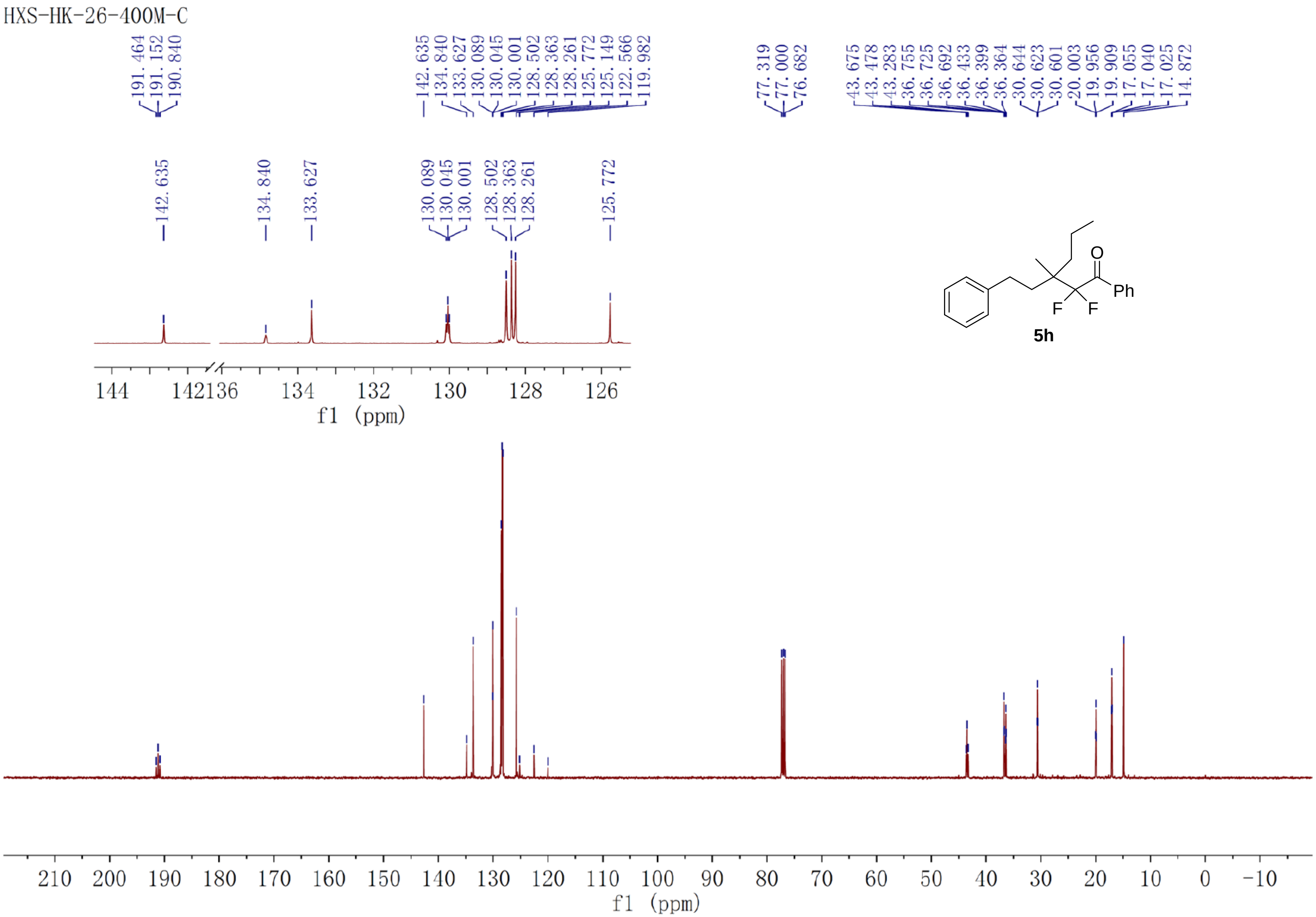

Supplementary Figure 153. ^{13}C NMR (100 MHz, $CDCl_3$) spectra for compound **5h**

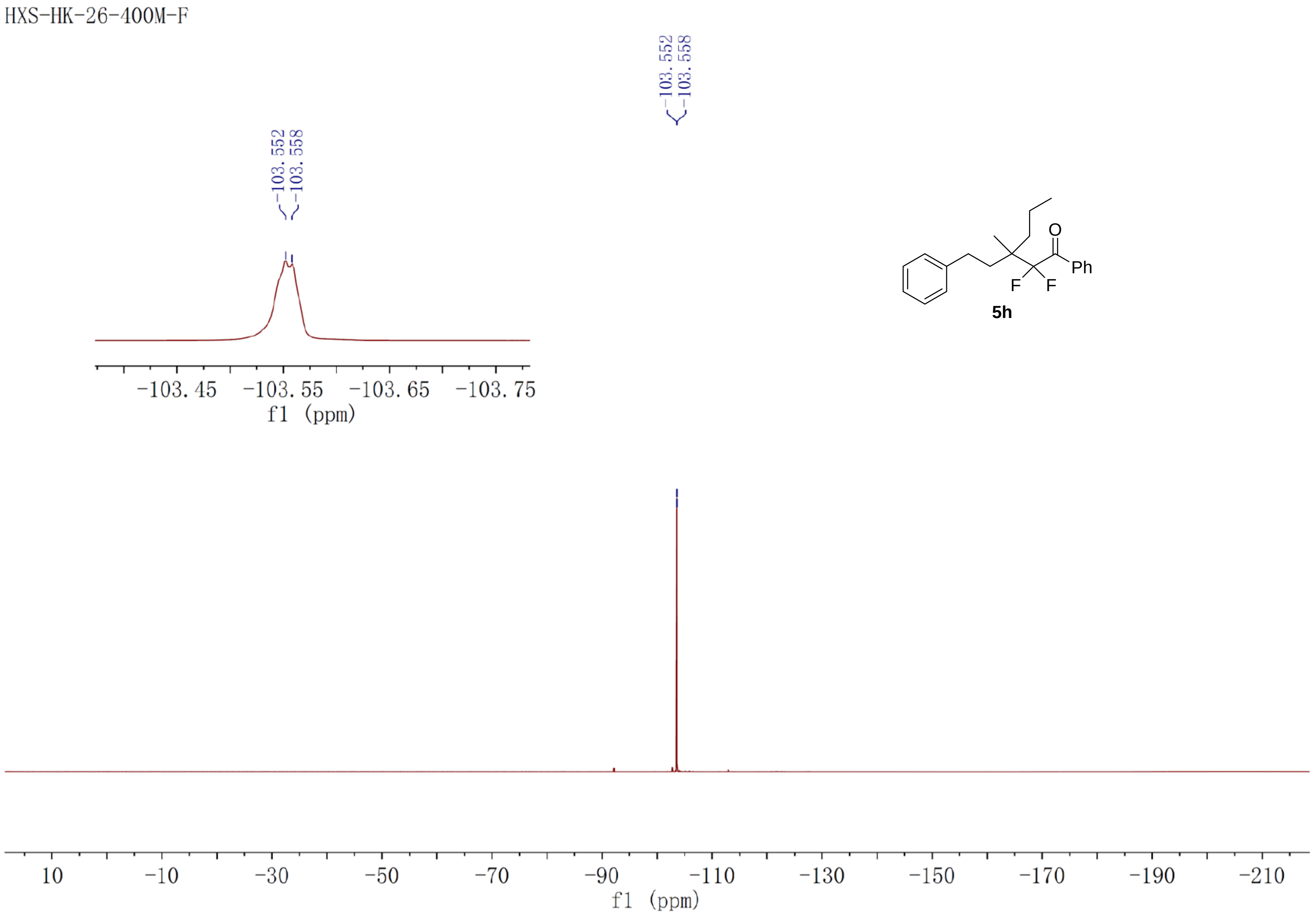

Supplementary Figure 154. ^{19}F NMR (376 MHz, $CDCl_3$) spectra for compound **5h**

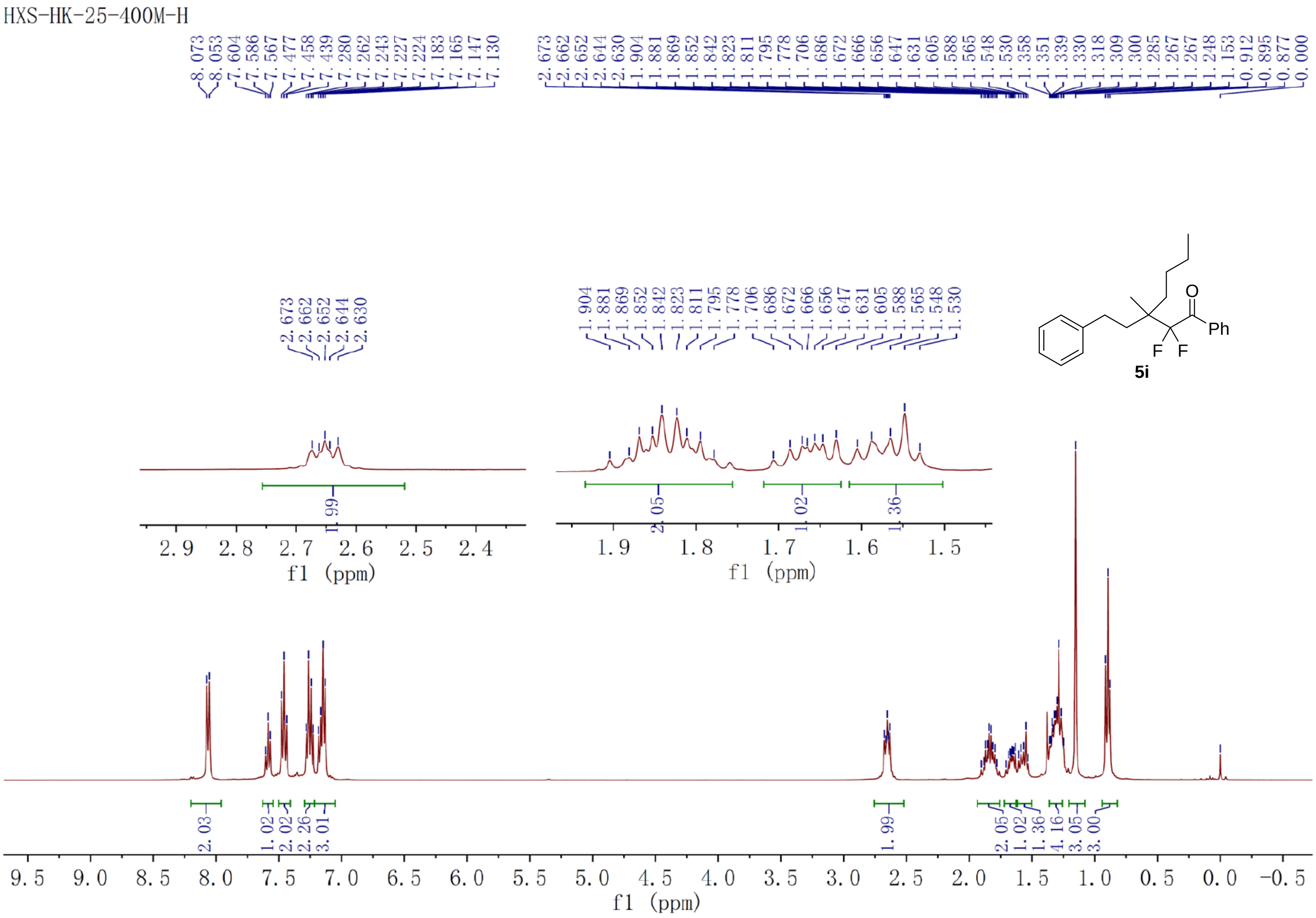

Supplementary Figure 155. ^{1}H NMR (400 MHz, $CDCl_3$) spectra for compound **5i**

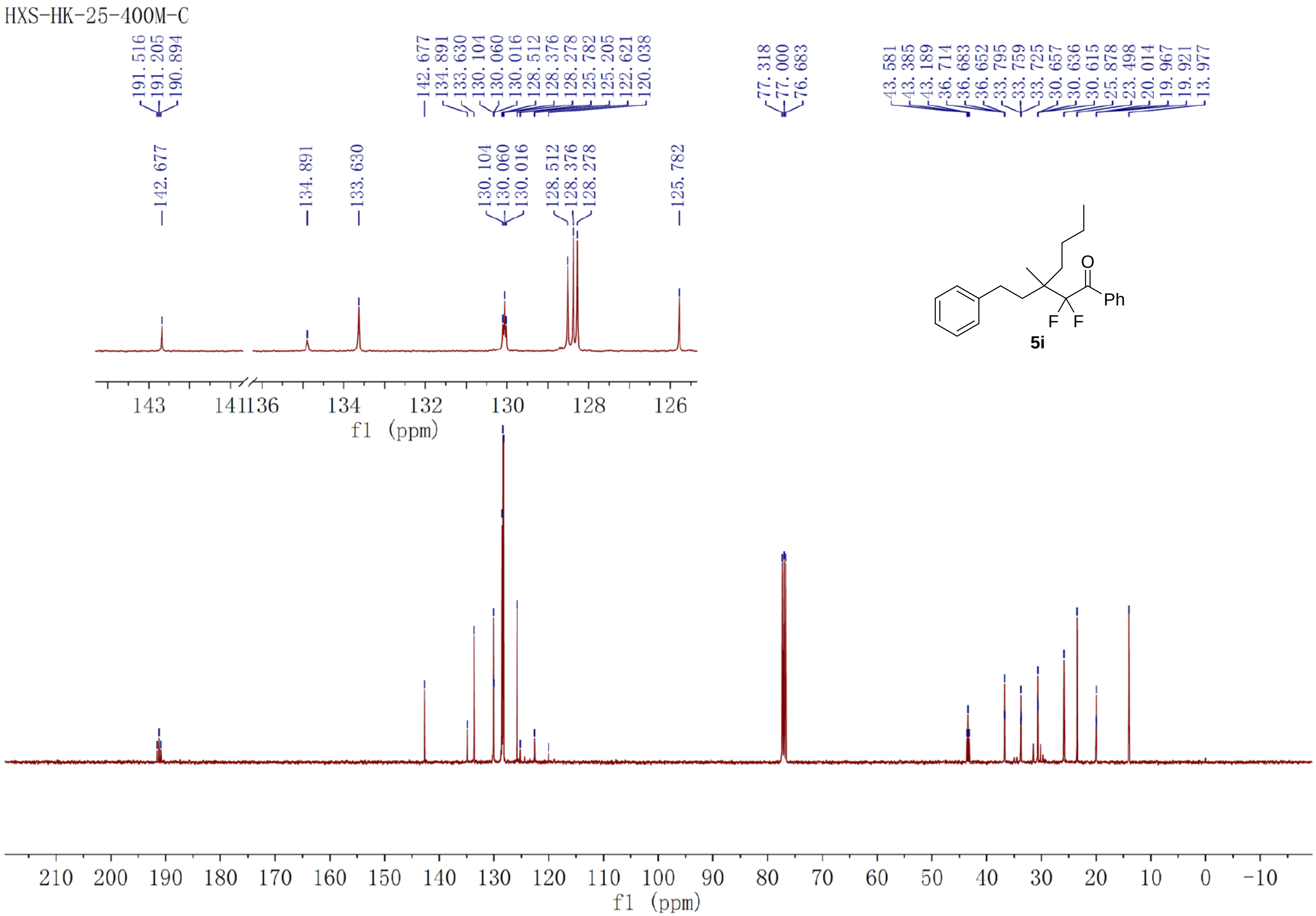

Supplementary Figure 156. ^{13}C NMR (100 MHz, $CDCl_3$) spectra for compound **5i**

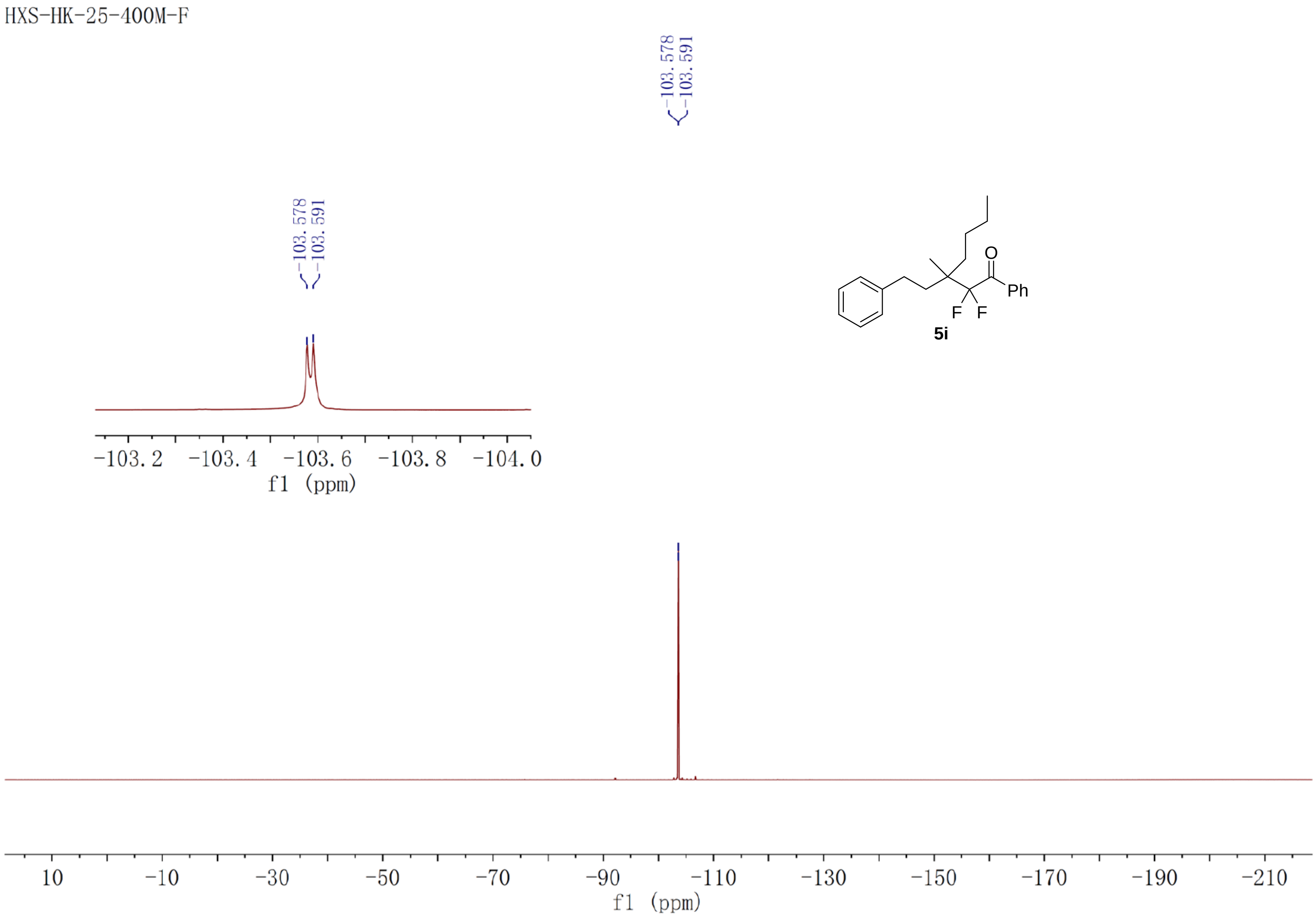

Supplementary Figure 157. ^{19}F NMR (376 MHz, $CDCl_3$) spectra for compound **5i**

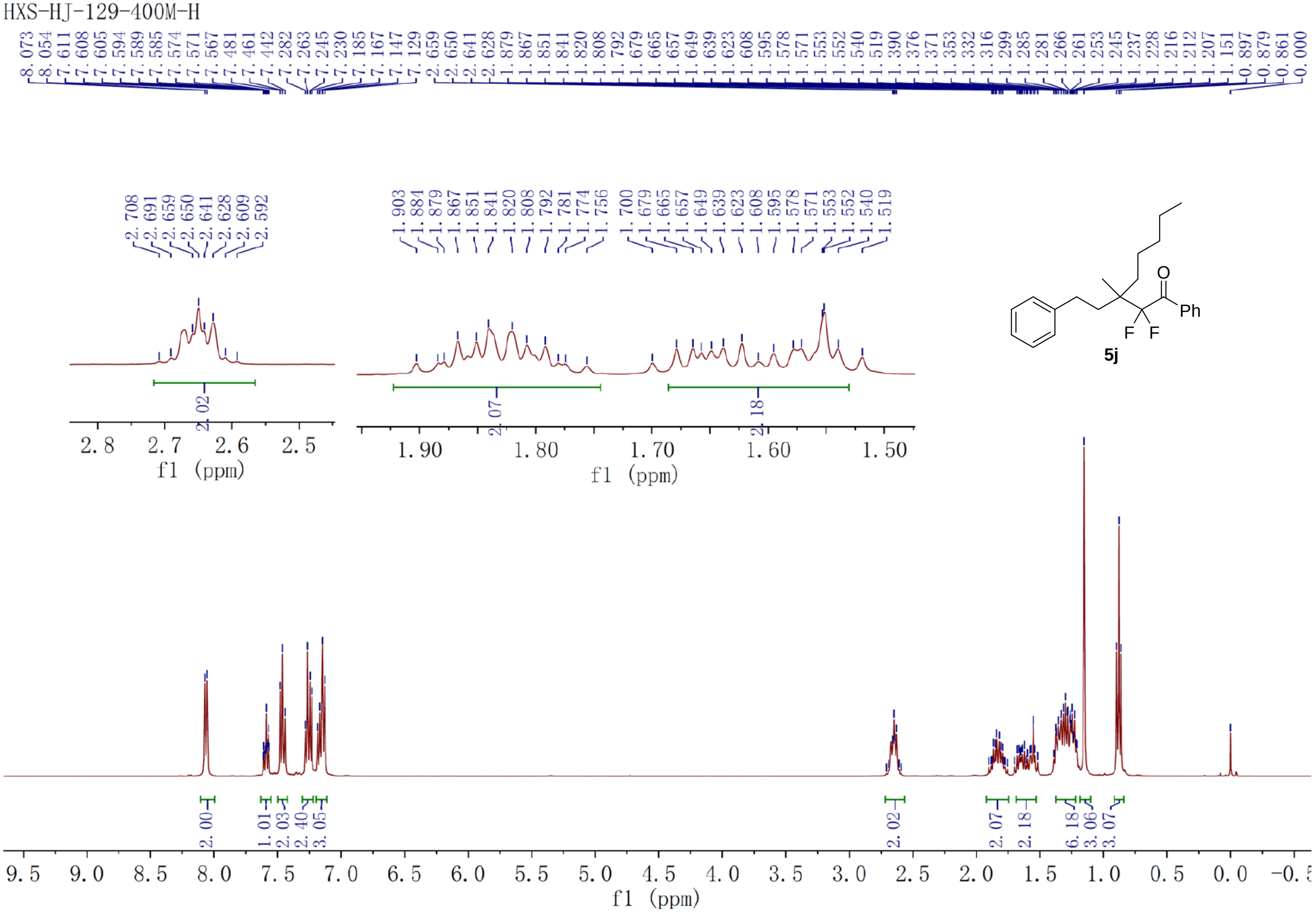

Supplementary Figure 158. ^{1}H NMR (400 MHz, $CDCl_3$) spectra for compound **5j**

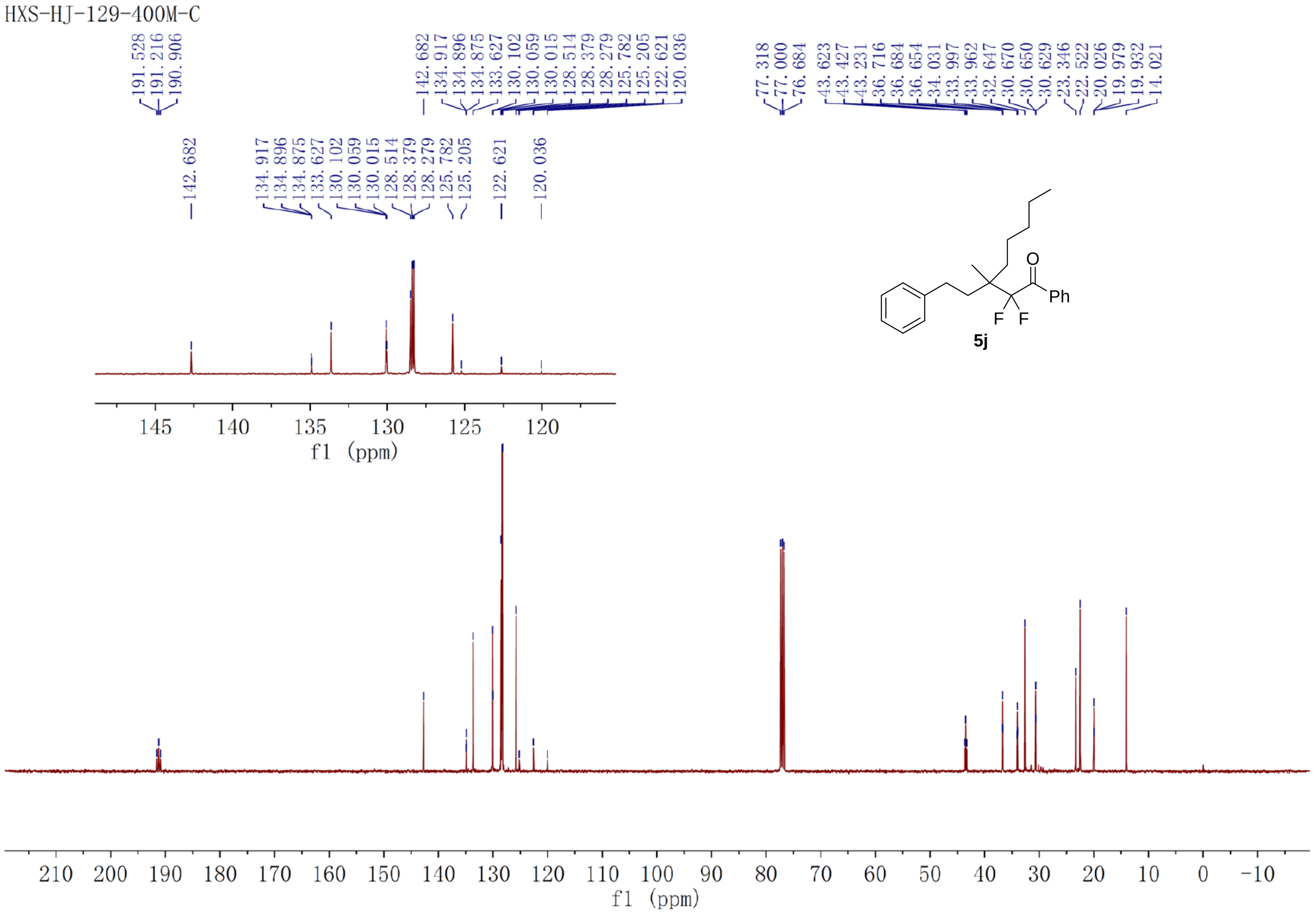

Supplementary Figure 159. ^{13}C NMR (100 MHz, $CDCl_3$) spectra for compound **5j**

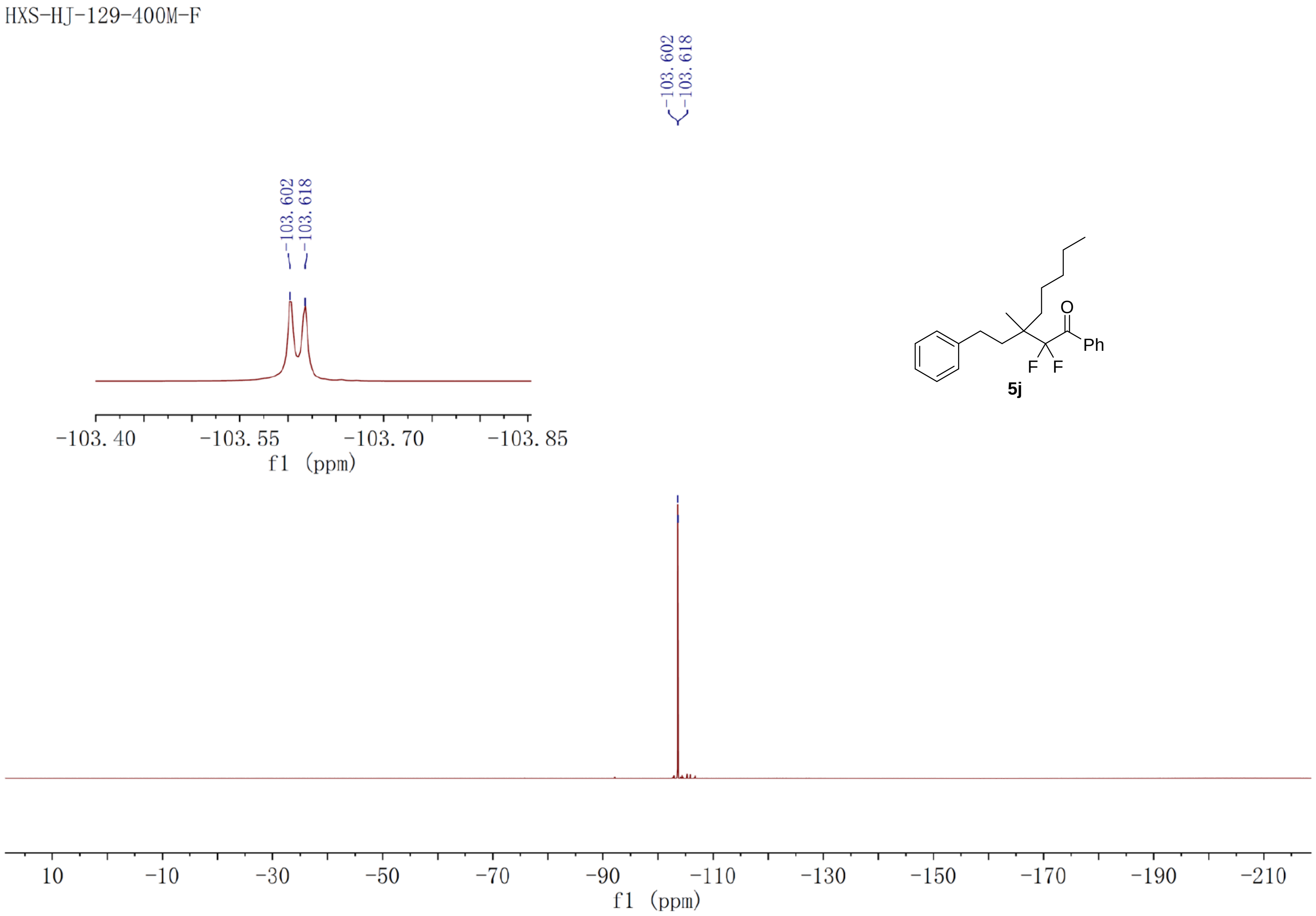

Supplementary Figure 160. ^{19}F NMR (376 MHz, $CDCl_3$) spectra for compound **5j**

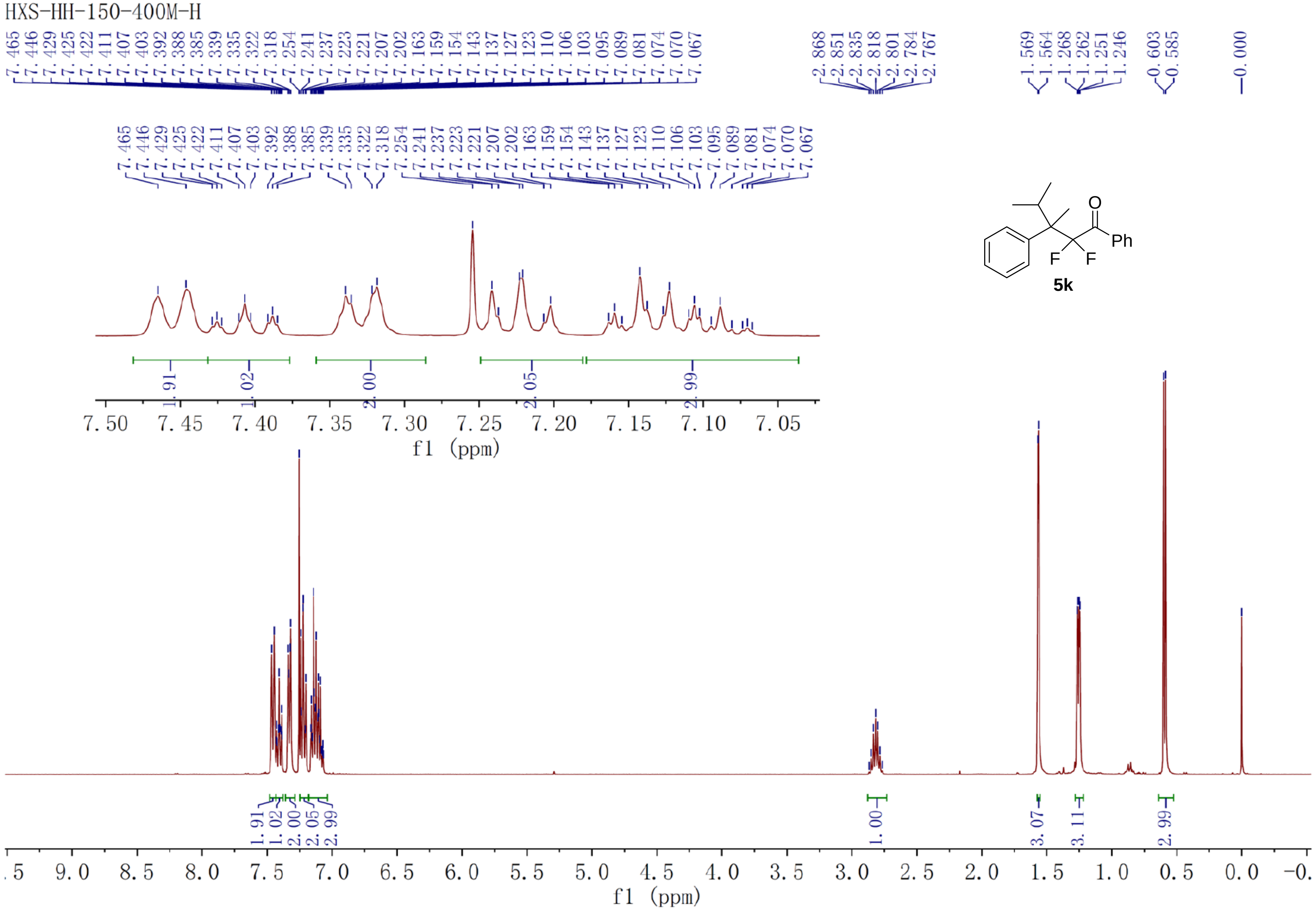

Supplementary Figure 161. ^{1}H NMR (400 MHz, $CDCl_3$) spectra for compound **5k**

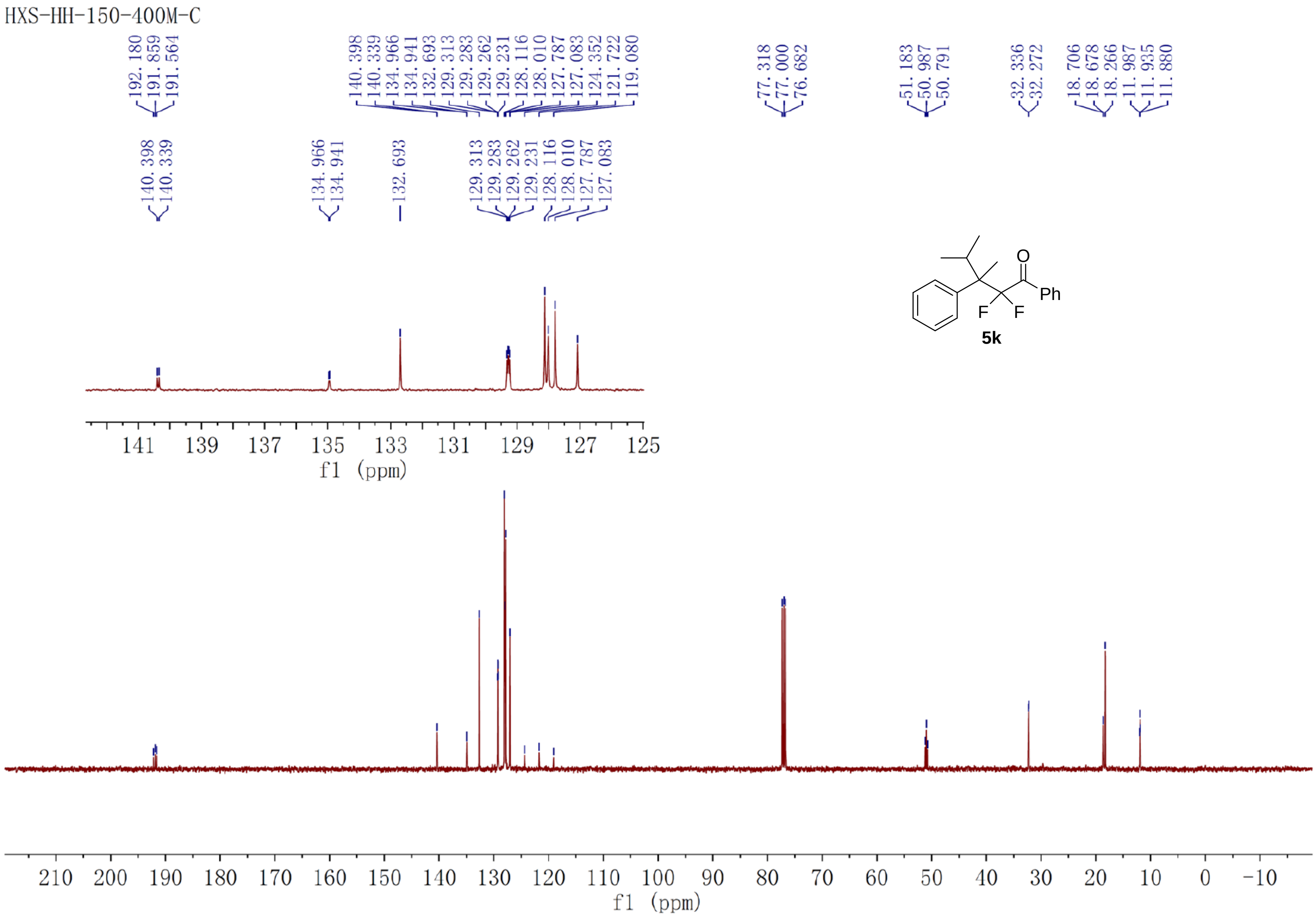

Supplementary Figure 162. ^{13}C NMR (100 MHz, $CDCl_3$) spectra for compound **5k**

HXS-HH-150-400M-F

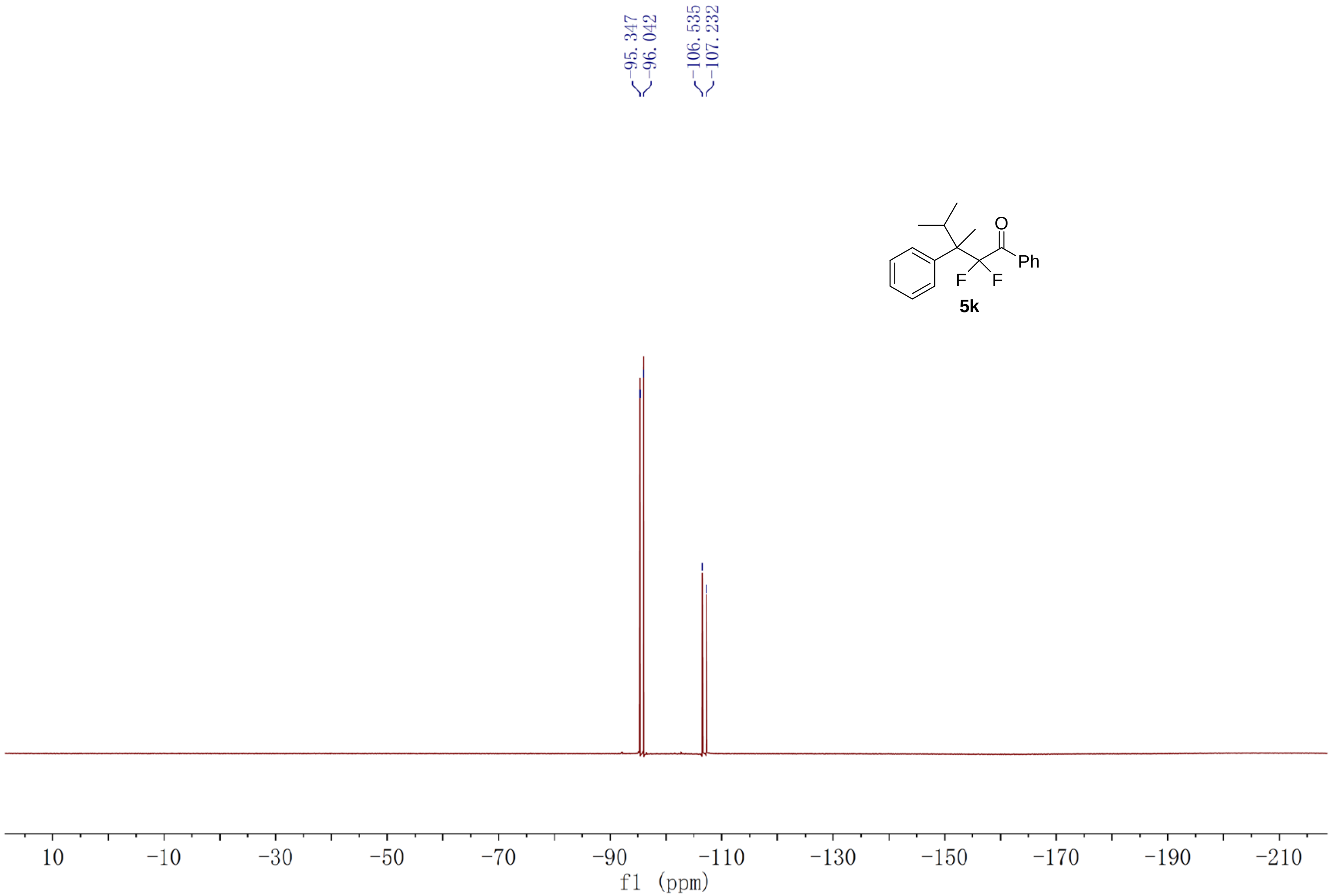

5k

Supplementary Figure 163. ^{19}F NMR (376 MHz, $CDCl_3$) spectra for compound **5k**

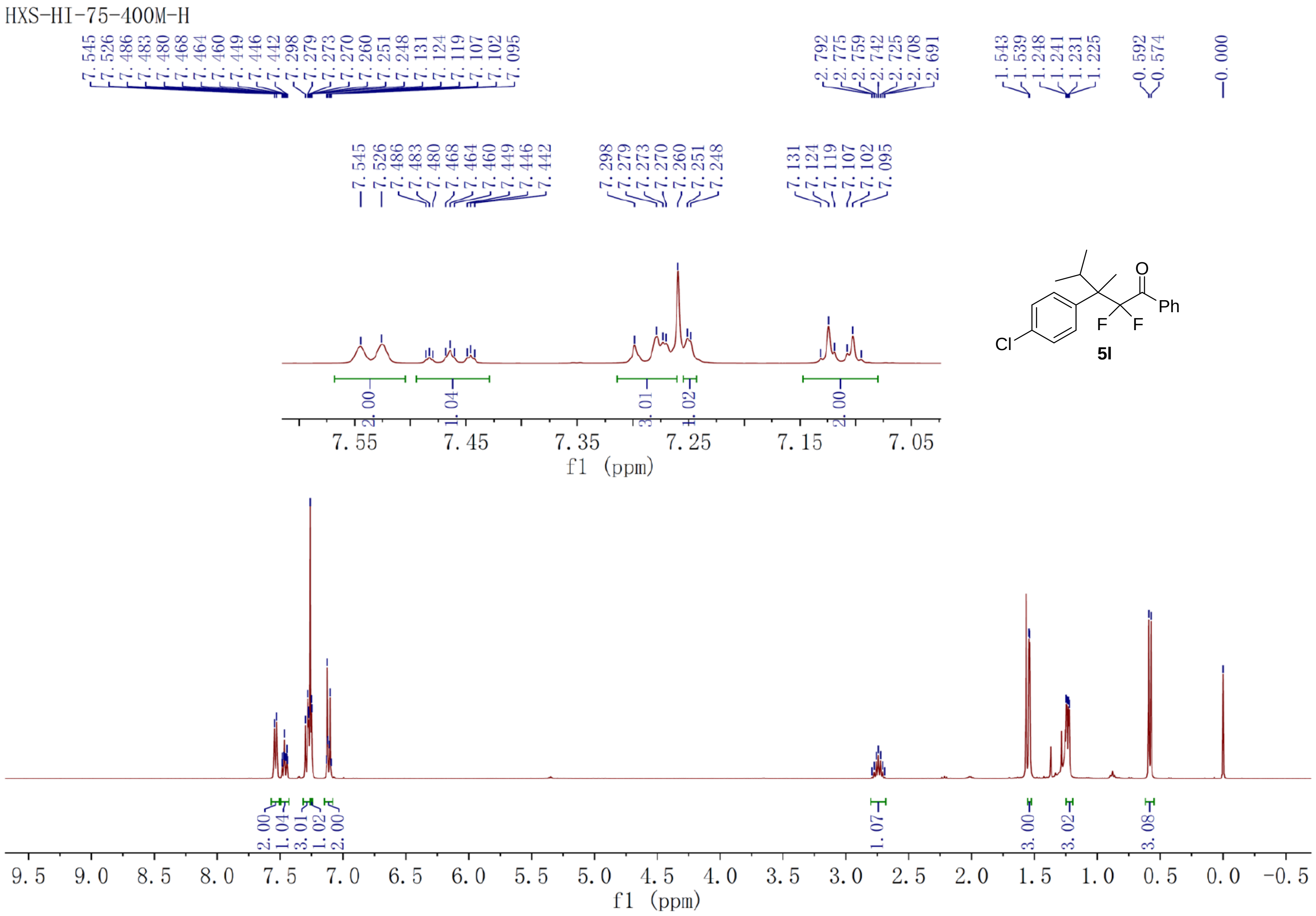

Supplementary Figure 164. ^{1}H NMR (400 MHz, $CDCl_3$) spectra for compound **5l**

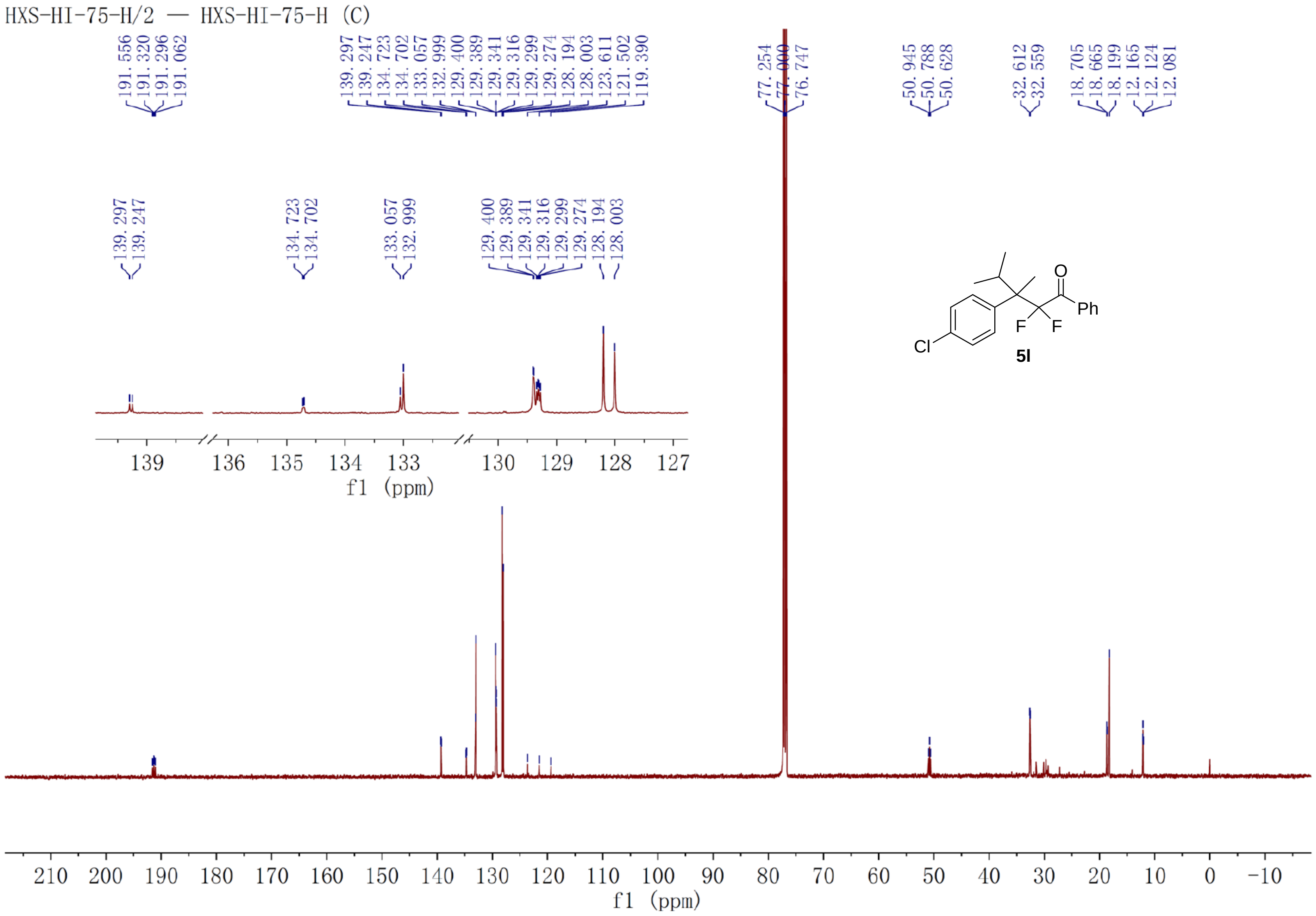

Supplementary Figure 165. ^{13}C NMR (125 MHz, $CDCl_3$) spectra for compound **5l**

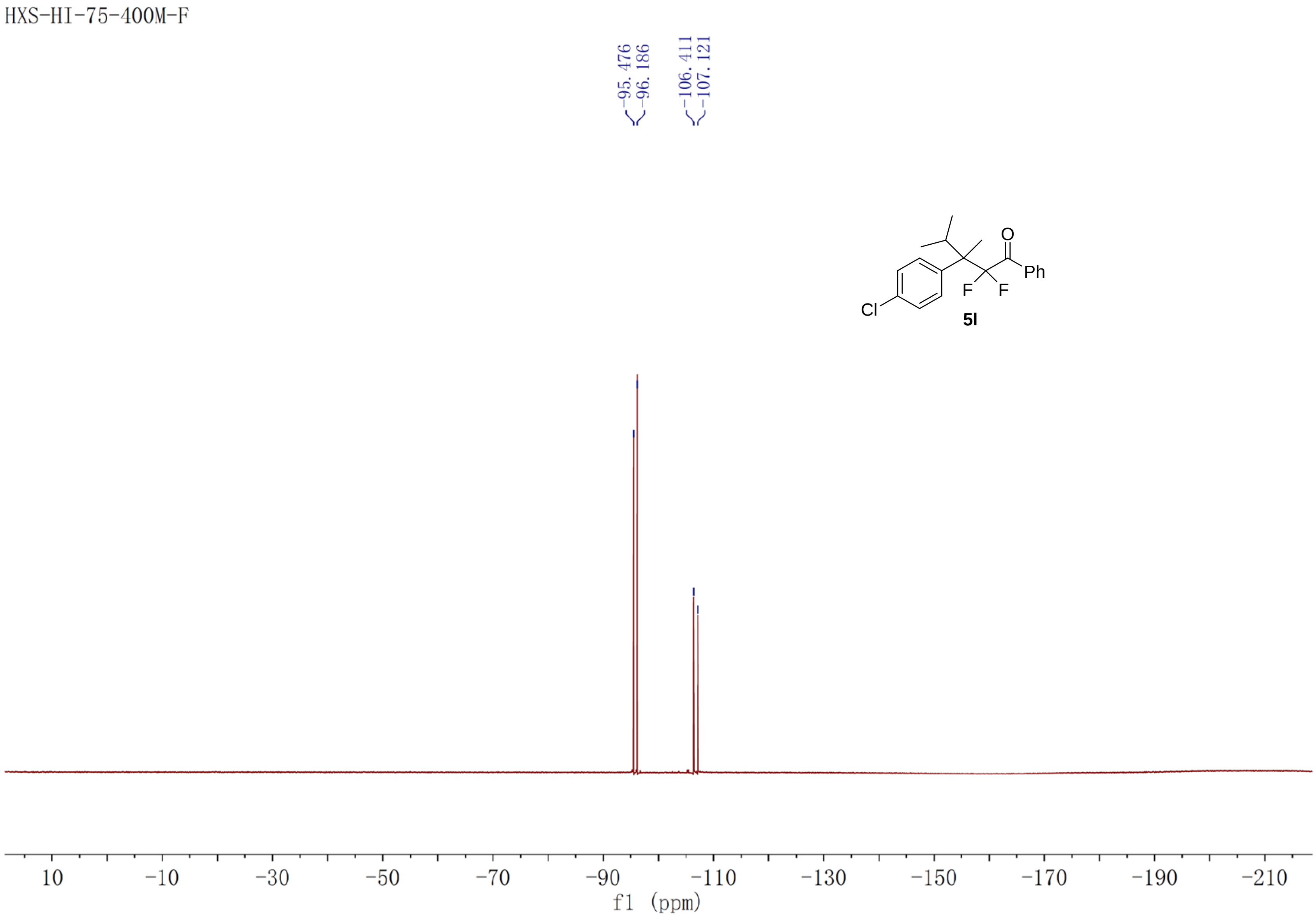

Supplementary Figure 166. ^{19}F NMR (376 MHz, $CDCl_3$) spectra for compound **5l**

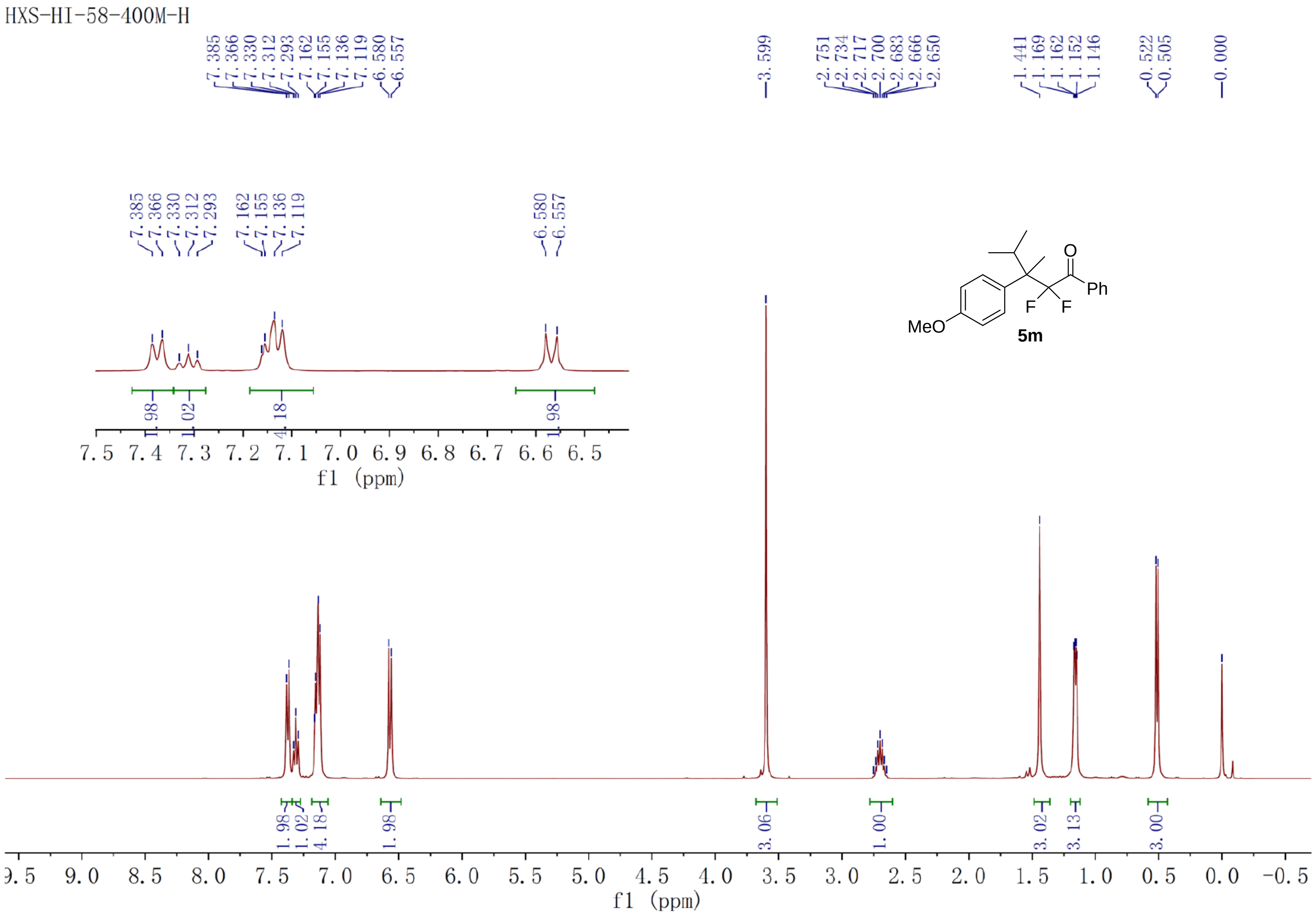

Supplementary Figure 167. ^{1}H NMR (400 MHz, $CDCl_3$) spectra for compound **5m**

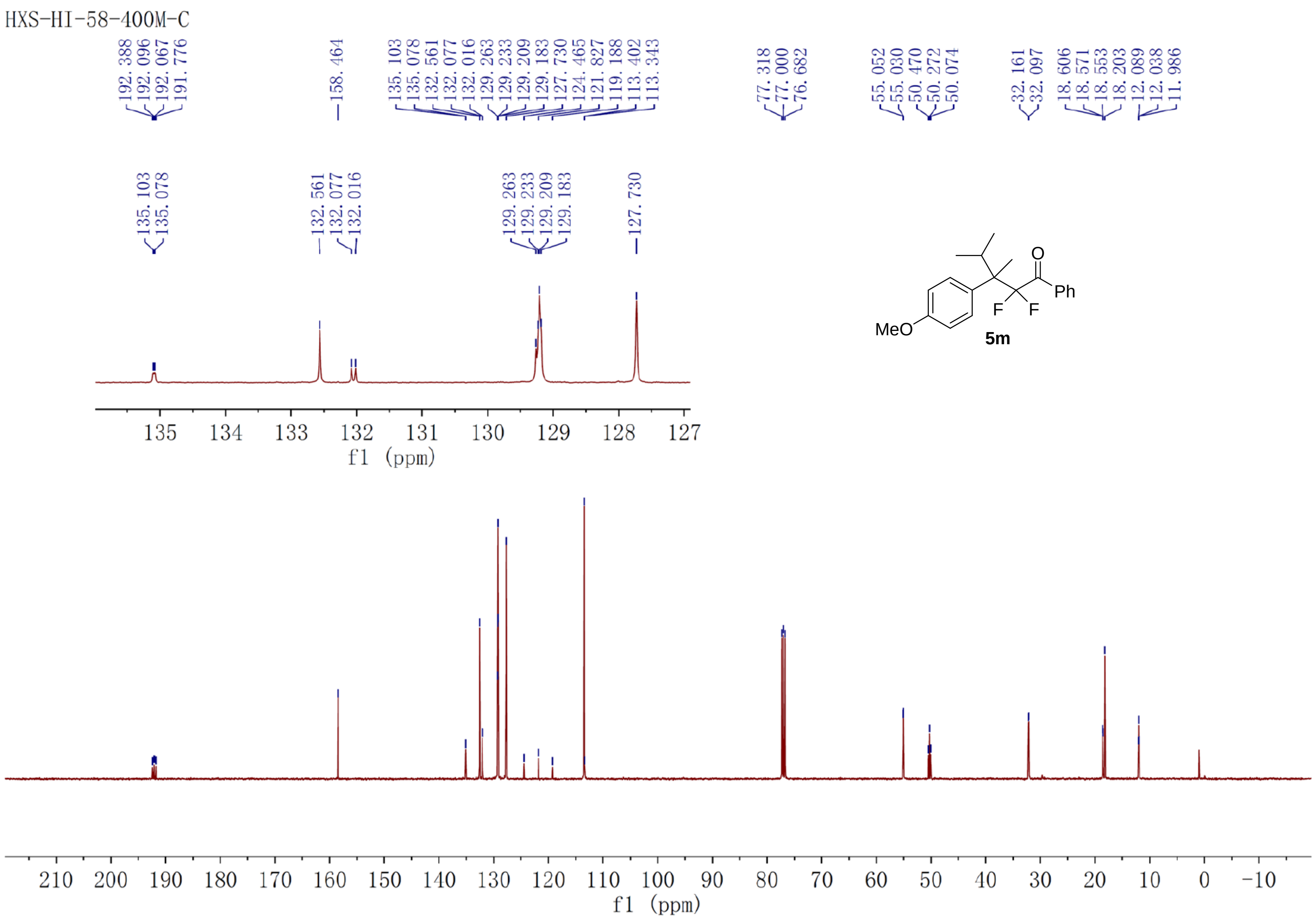

Supplementary Figure 168. ^{13}C NMR (100 MHz, $CDCl_3$) spectra for compound **5m**

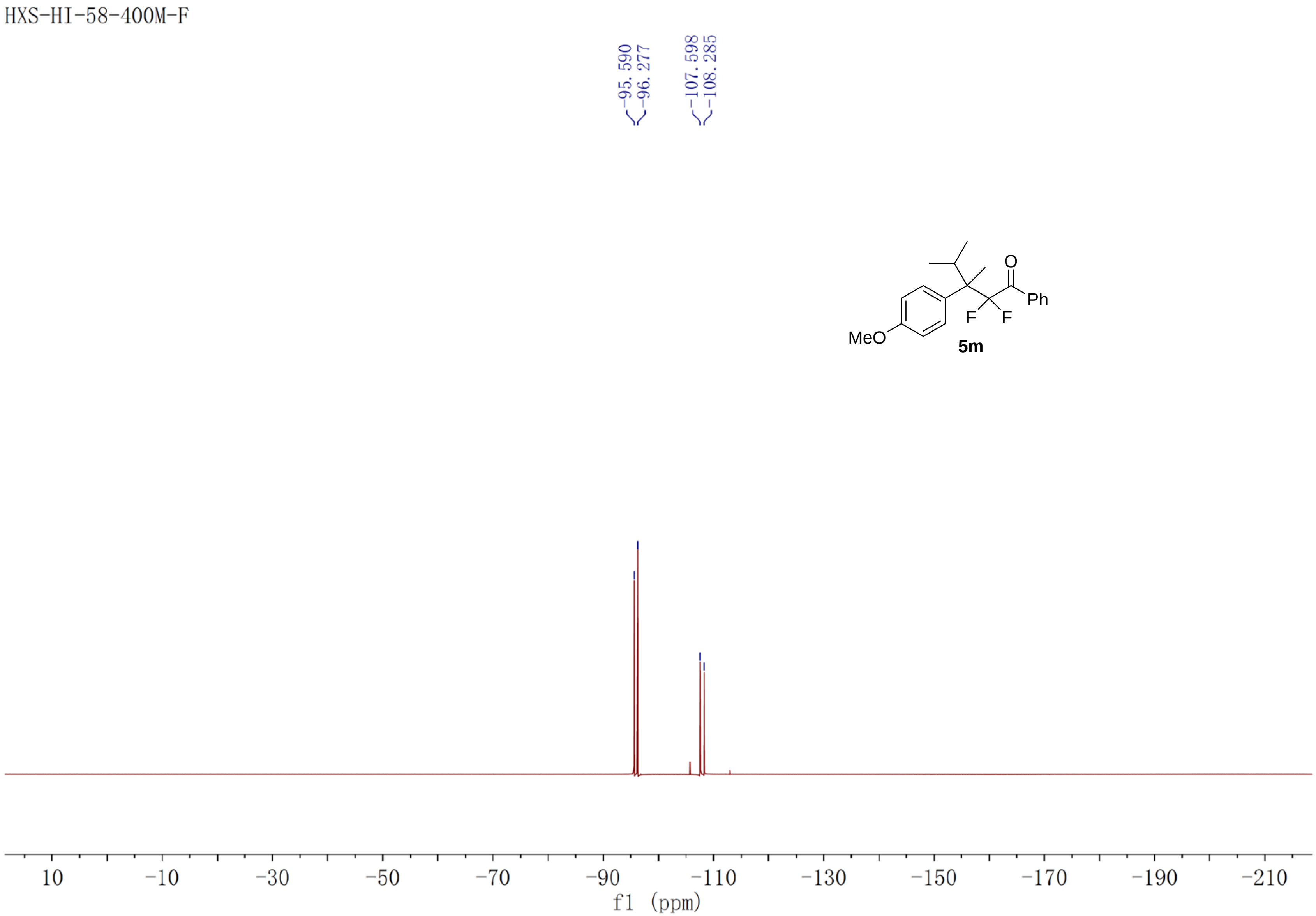

Supplementary Figure 169. ^{19}F NMR (376 MHz, $CDCl_3$) spectra for compound **5m**

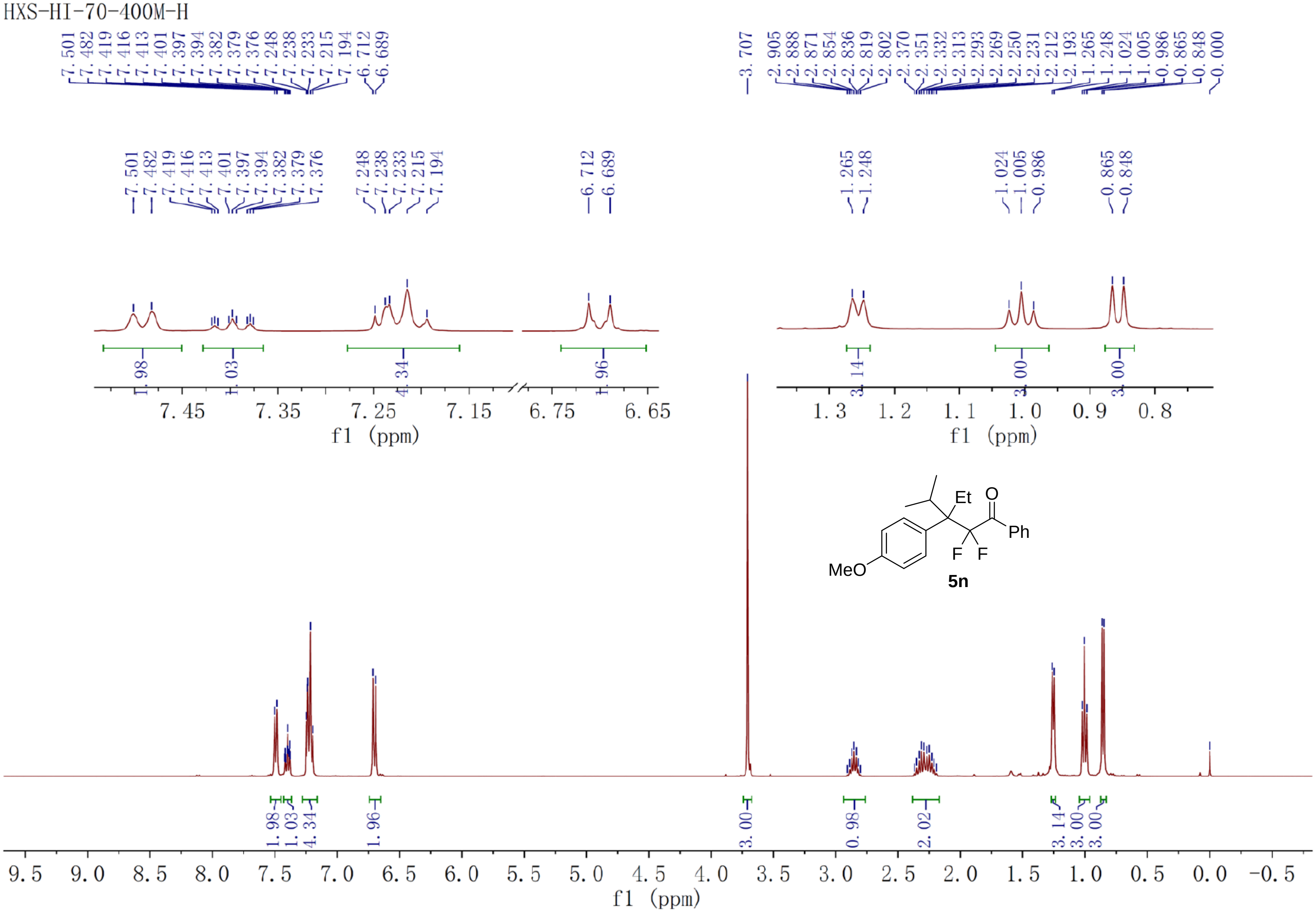

Supplementary Figure 170. ^{1}H NMR (400 MHz, $CDCl_3$) spectra for compound **5n**

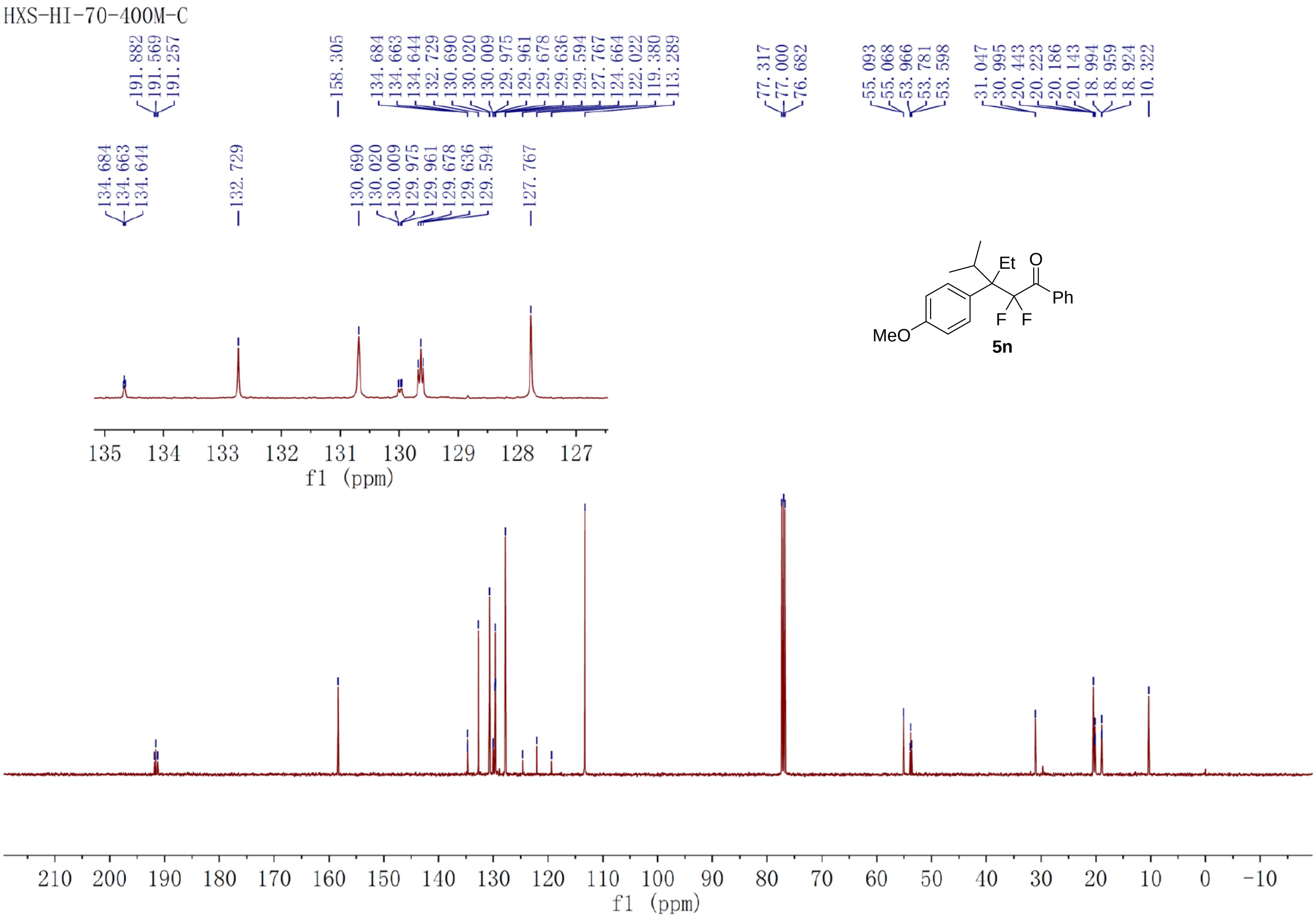

Supplementary Figure 171. ^{13}C NMR (100 MHz, $CDCl_3$) spectra for compound **5n**

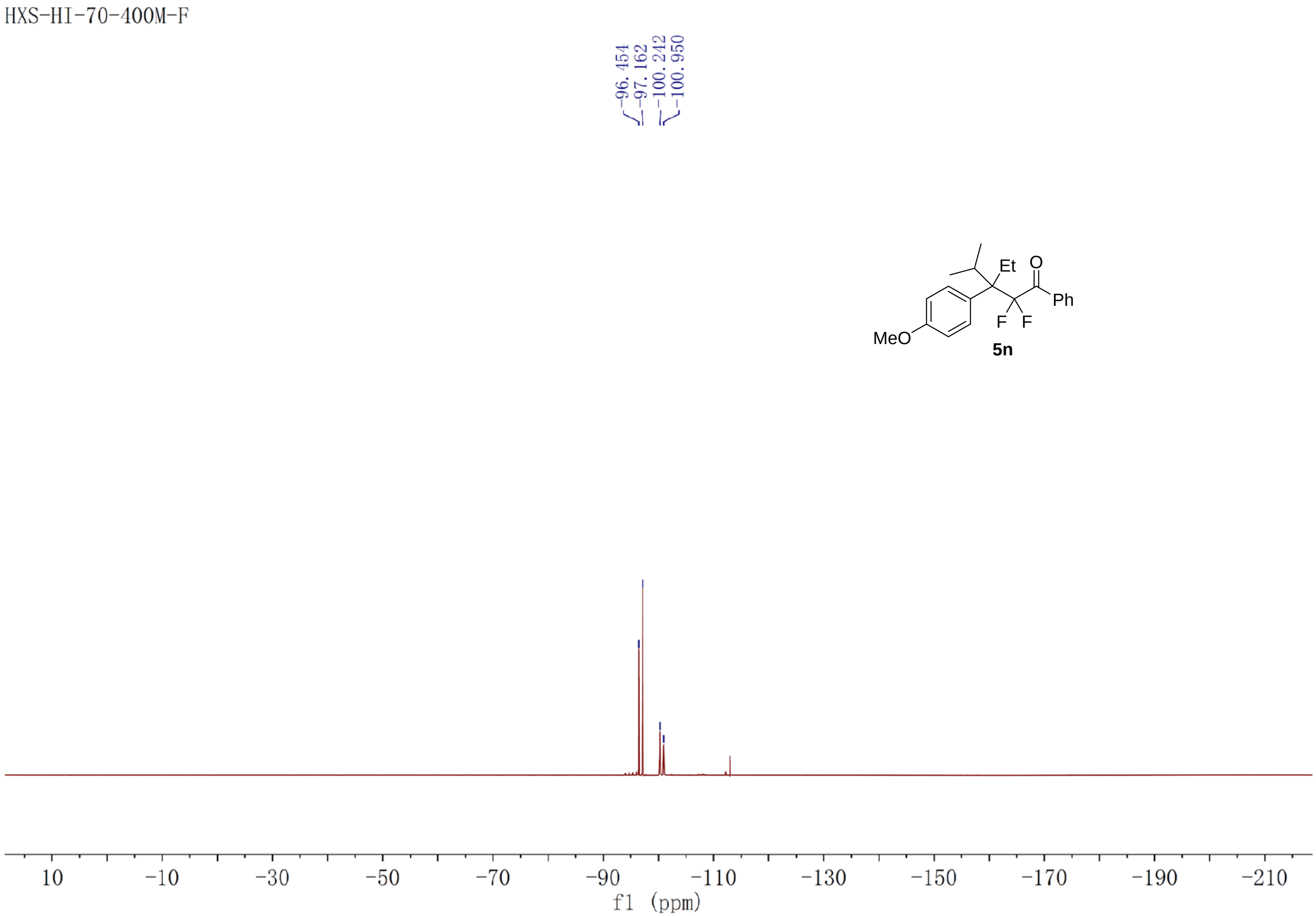

Supplementary Figure 172. ^{19}F NMR (376 MHz, $CDCl_3$) spectra for compound **5n**

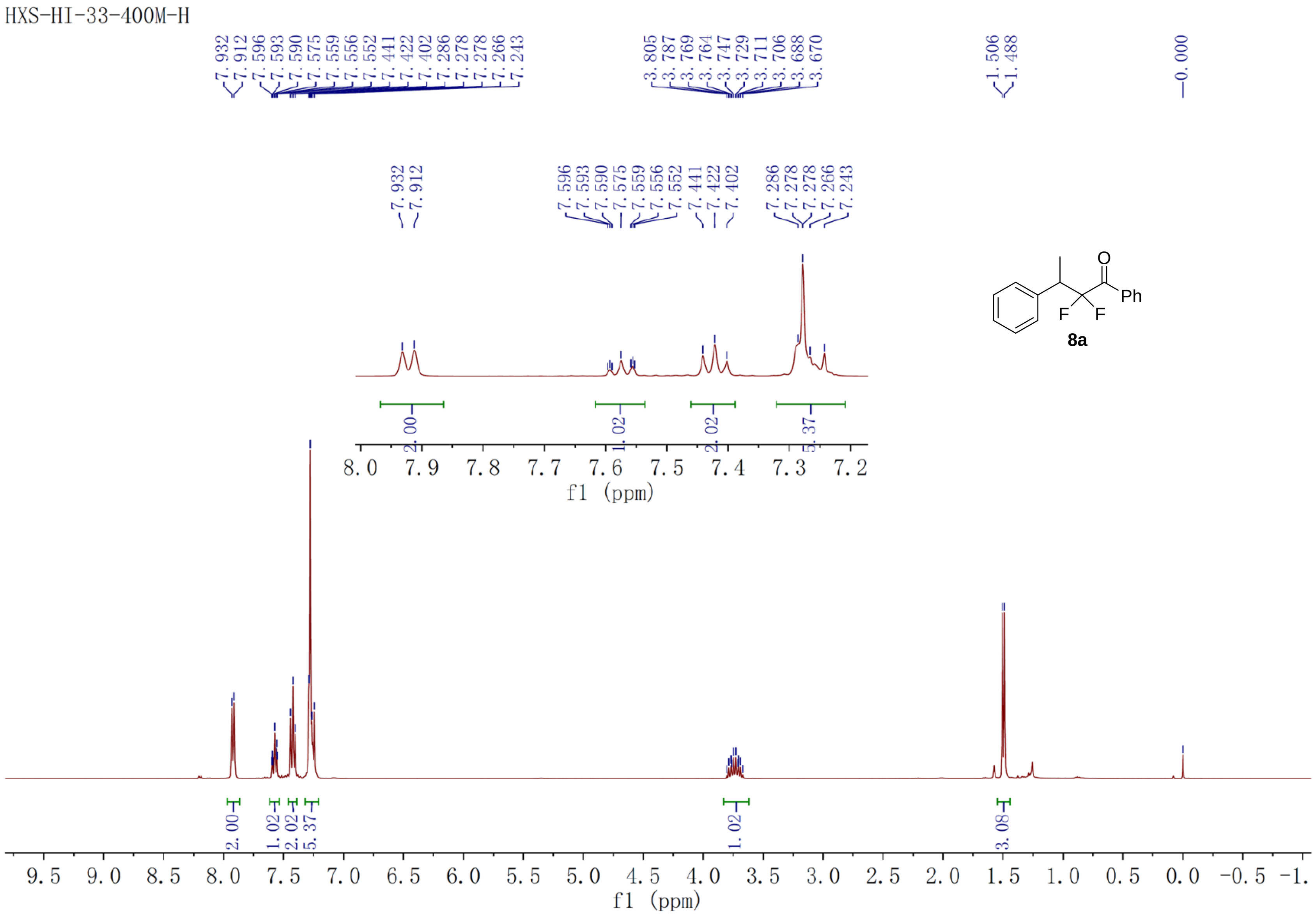

Supplementary Figure 173. ^{1}H NMR (400 MHz, $CDCl_3$) spectra for compound **8a**

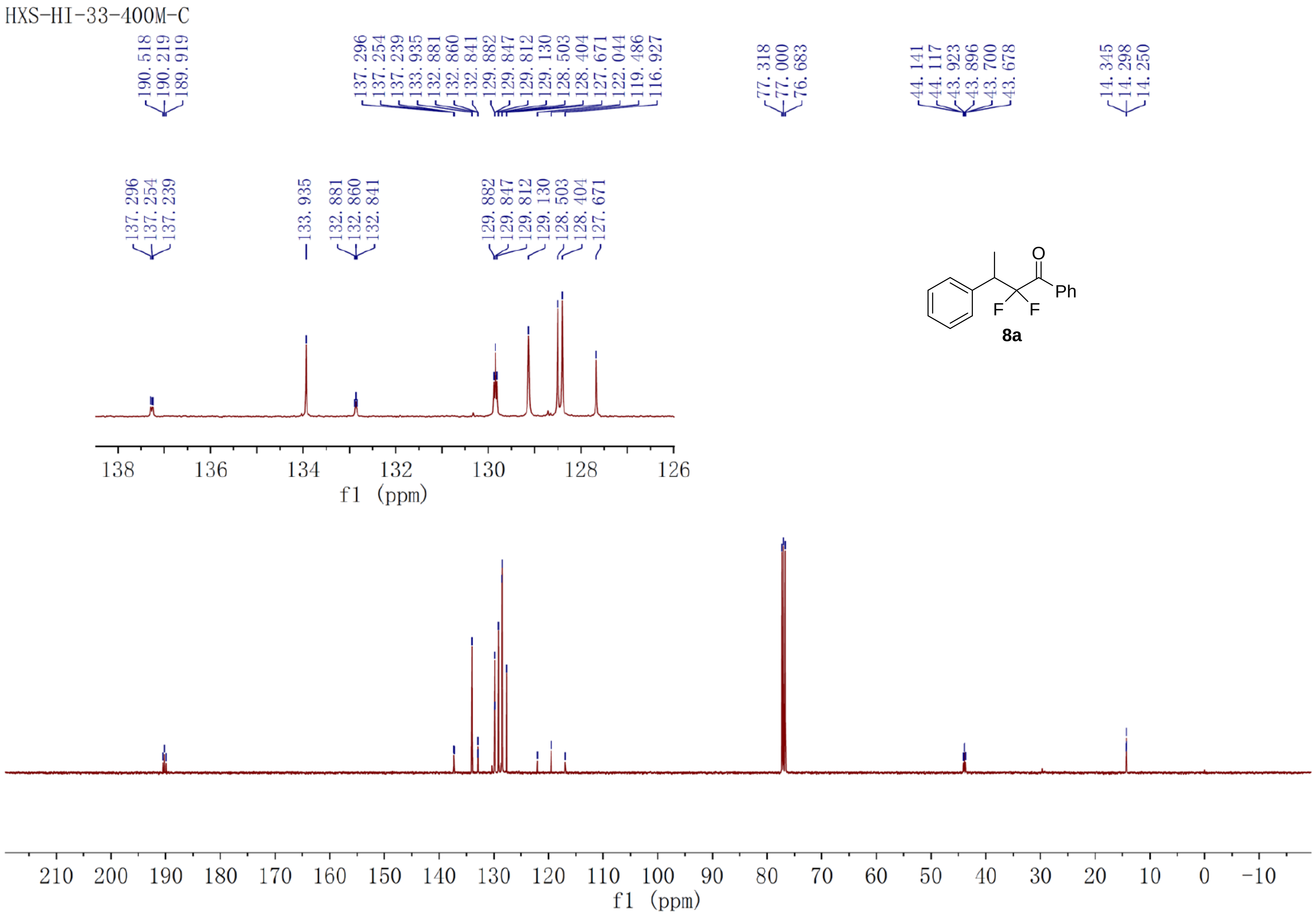

Supplementary Figure 174. ^{13}C NMR (100 MHz, $CDCl_3$) spectra for compound **8a**

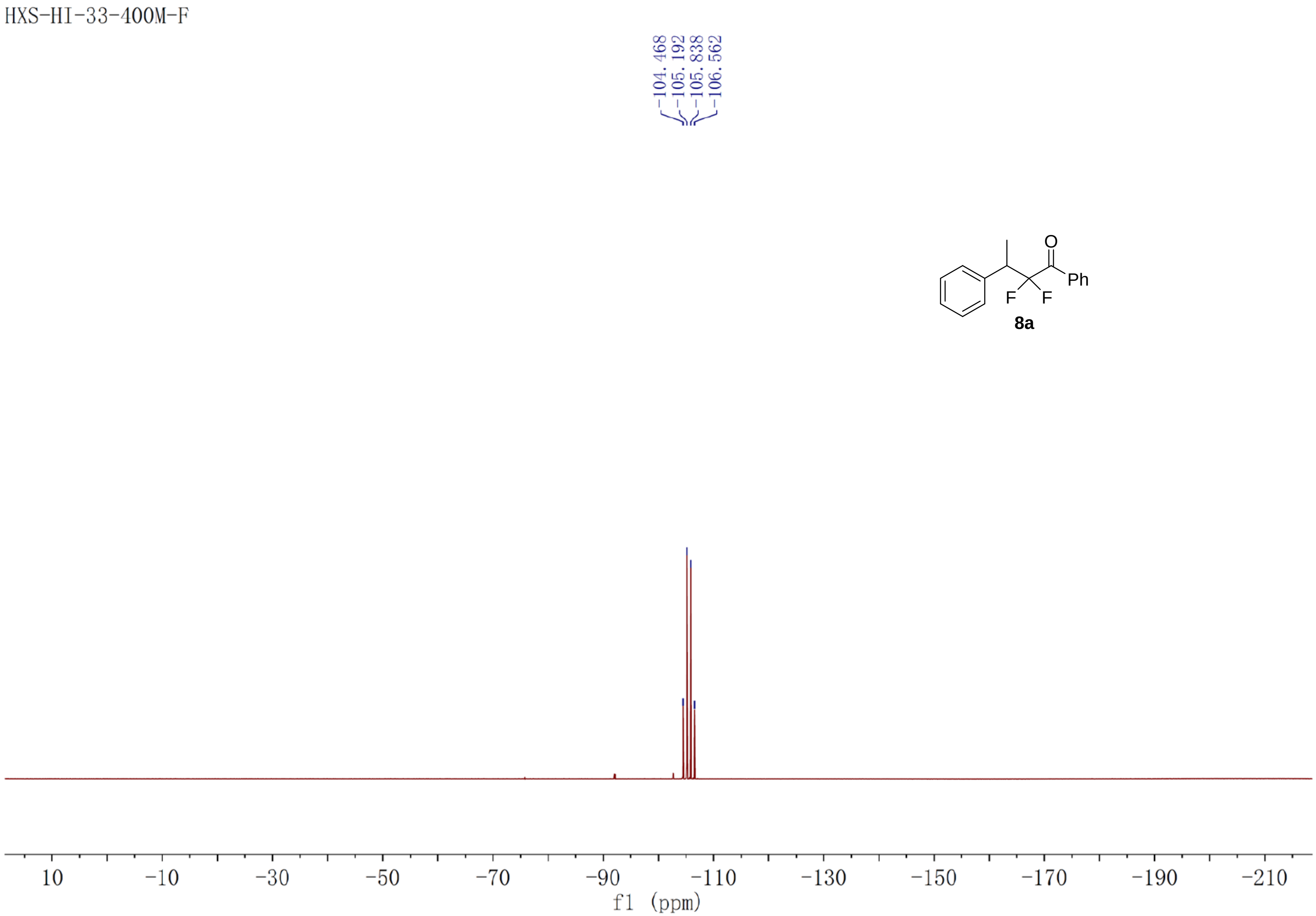

Supplementary Figure 175. ^{19}F NMR (376 MHz, $CDCl_3$) spectra for compound **8a**

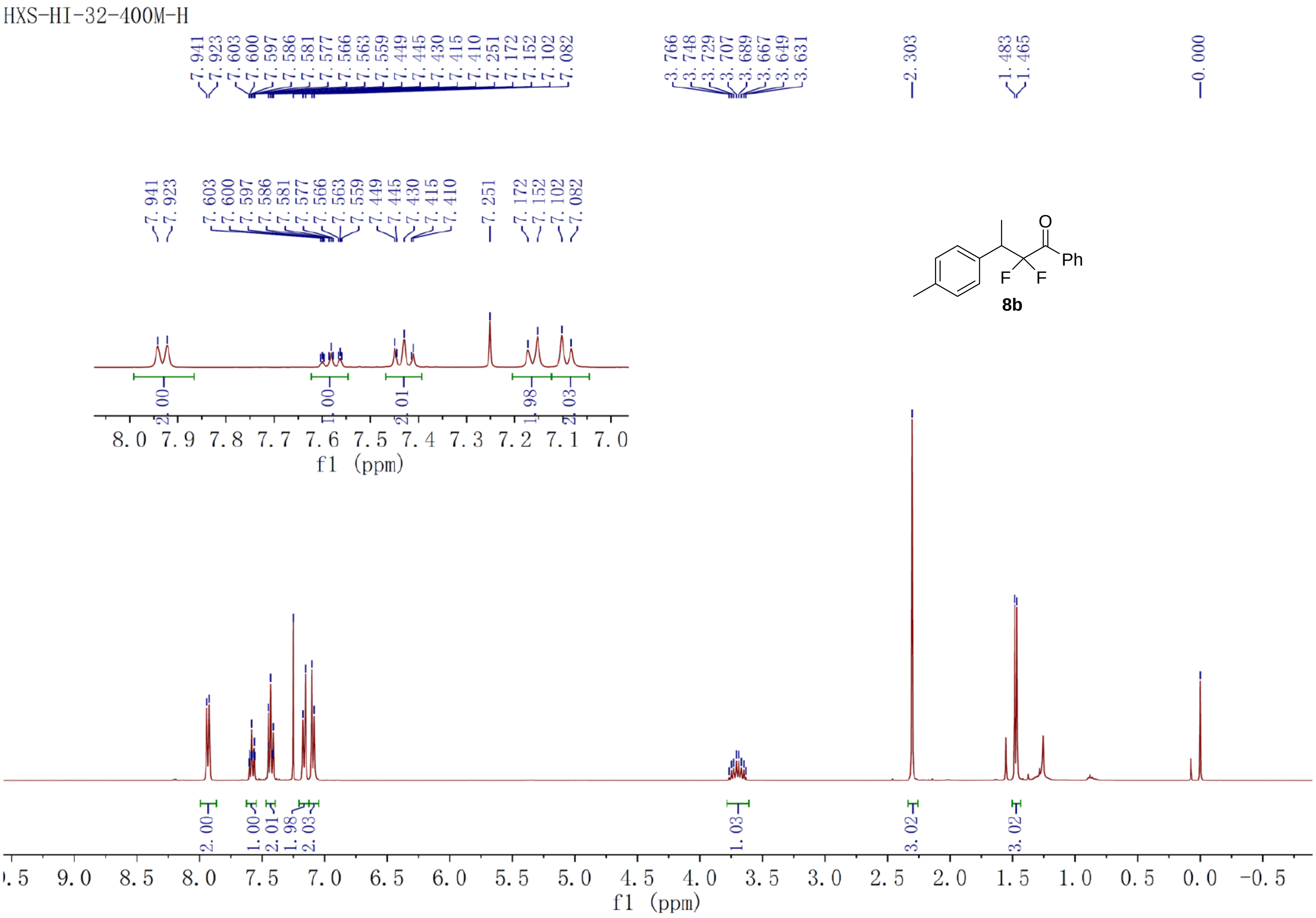

Supplementary Figure 176. ^{1}H NMR (400 MHz, $CDCl_3$) spectra for compound **8b**

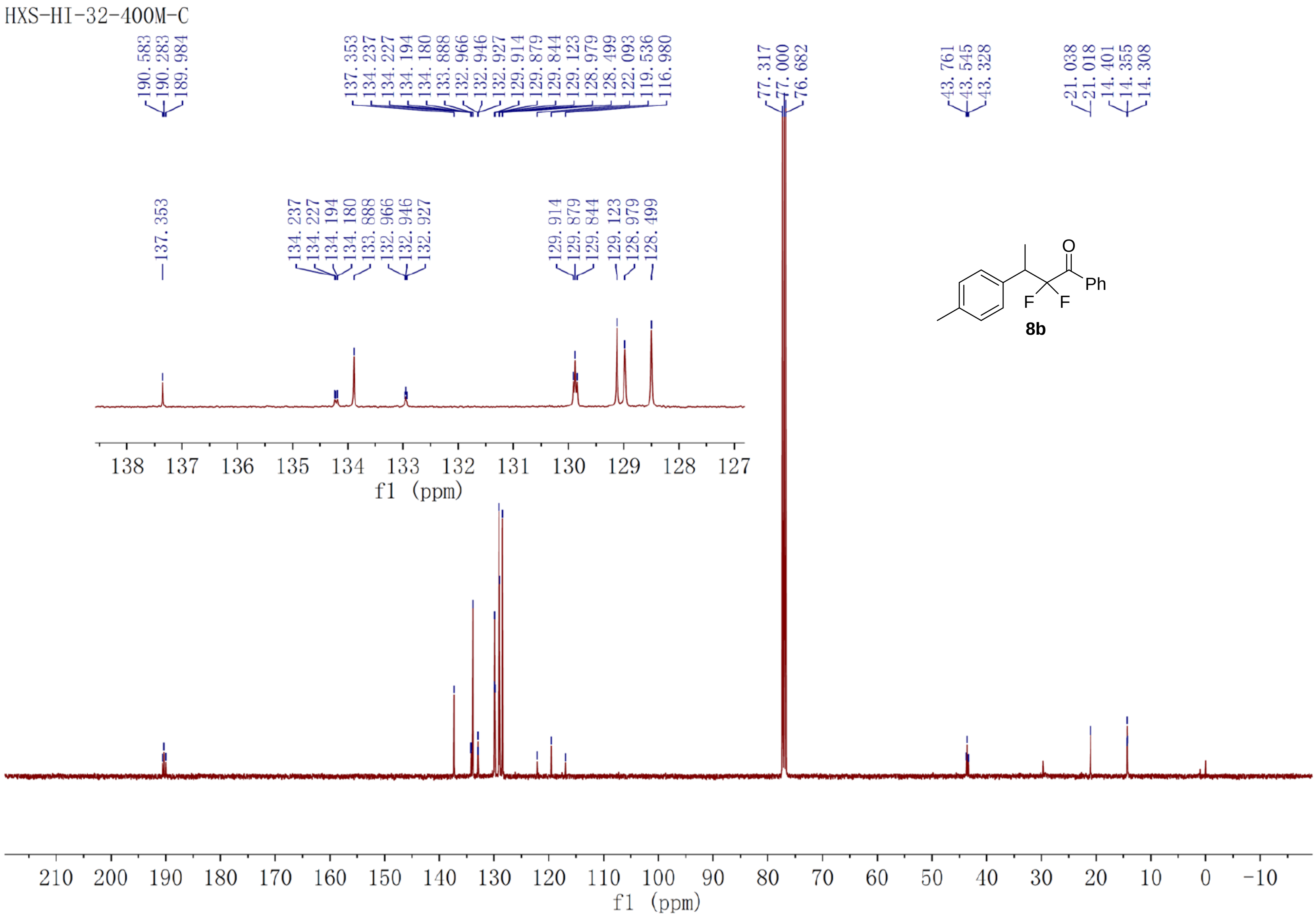

Supplementary Figure 177. ^{13}C NMR (100 MHz, $CDCl_3$) spectra for compound **8b**

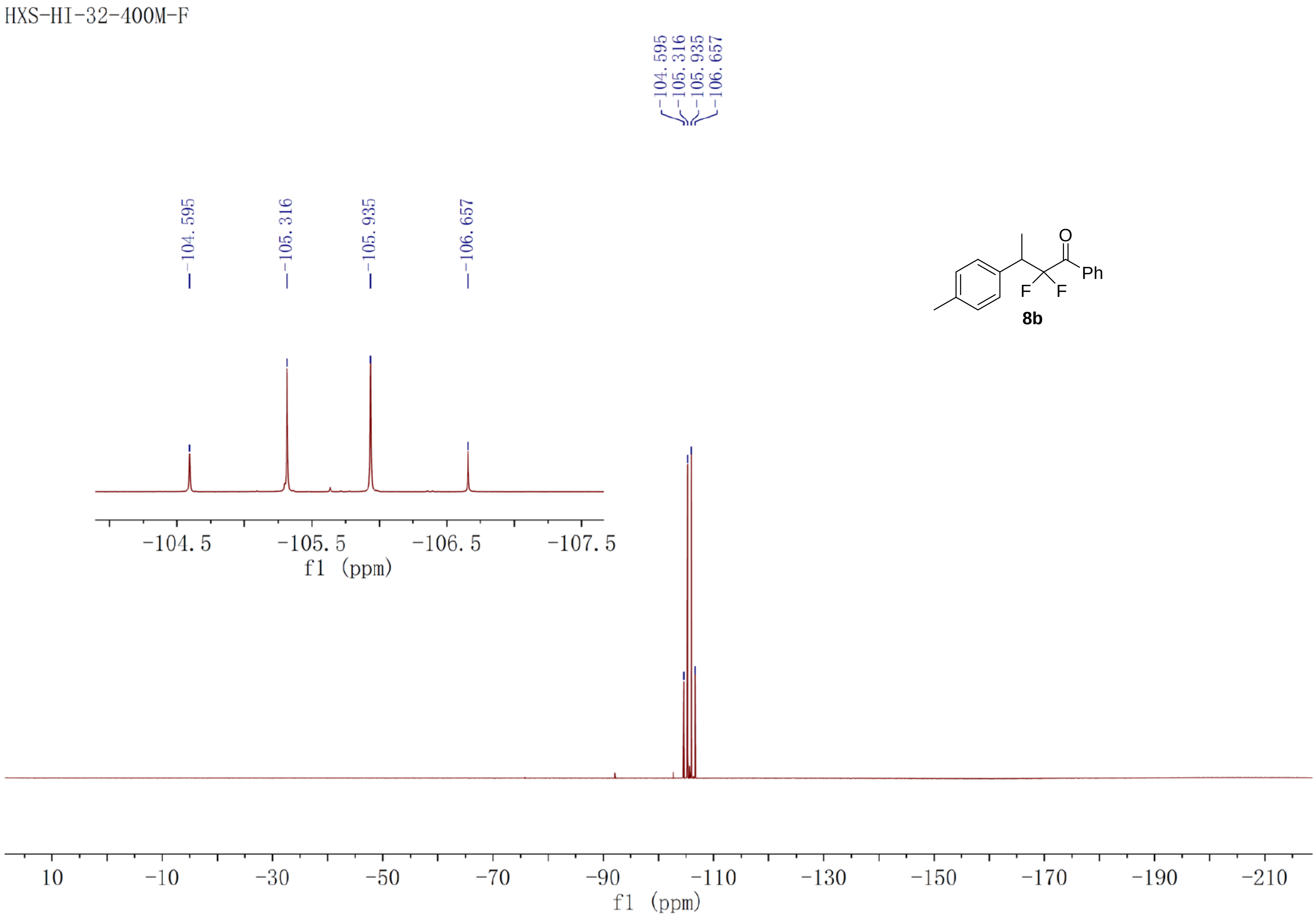

8b

Supplementary Figure 178. ^{19}F NMR (376 MHz, $CDCl_3$) spectra for compound **8b**

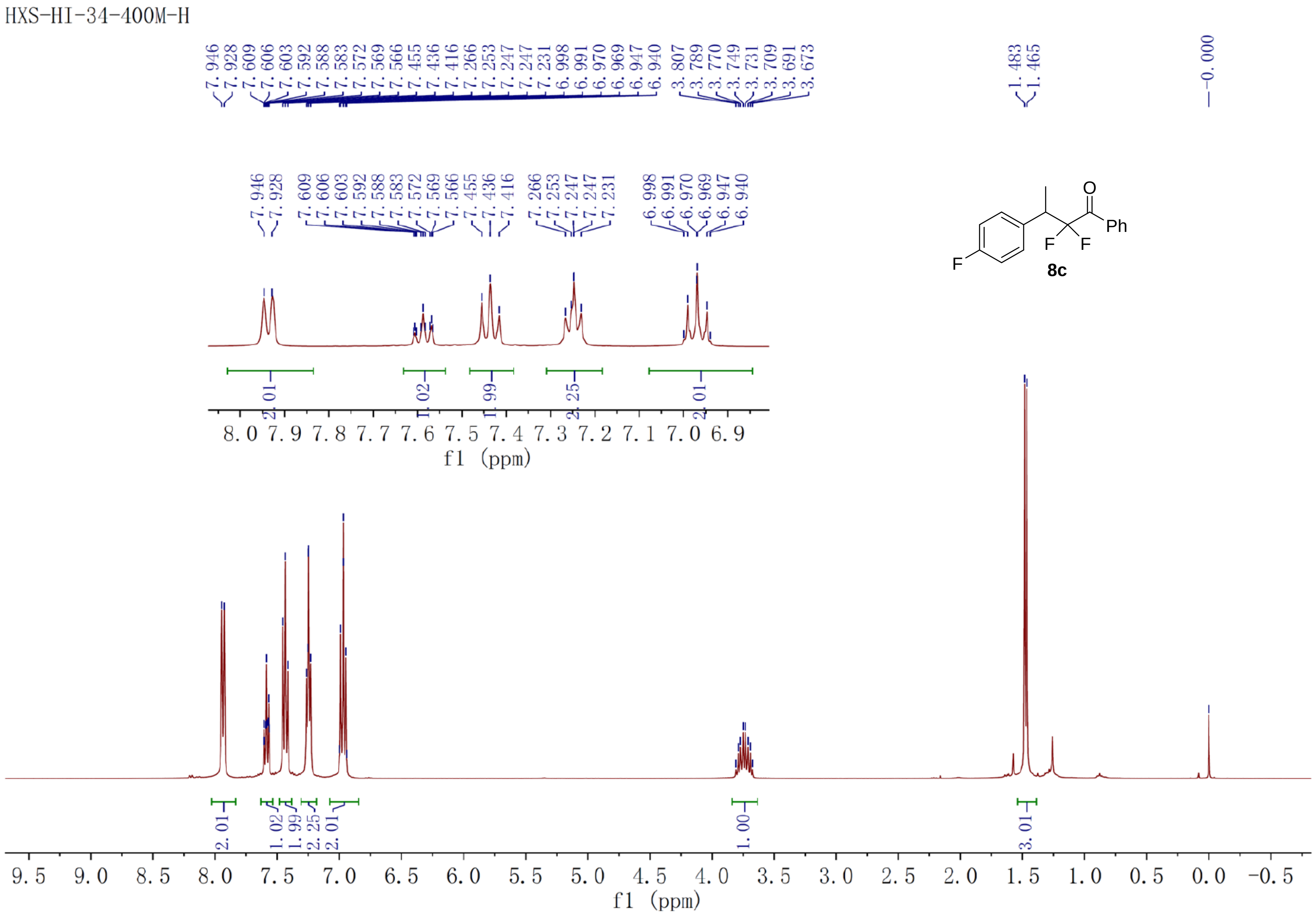

Supplementary Figure 179. ^{1}H NMR (400 MHz, $CDCl_3$) spectra for compound **8c**

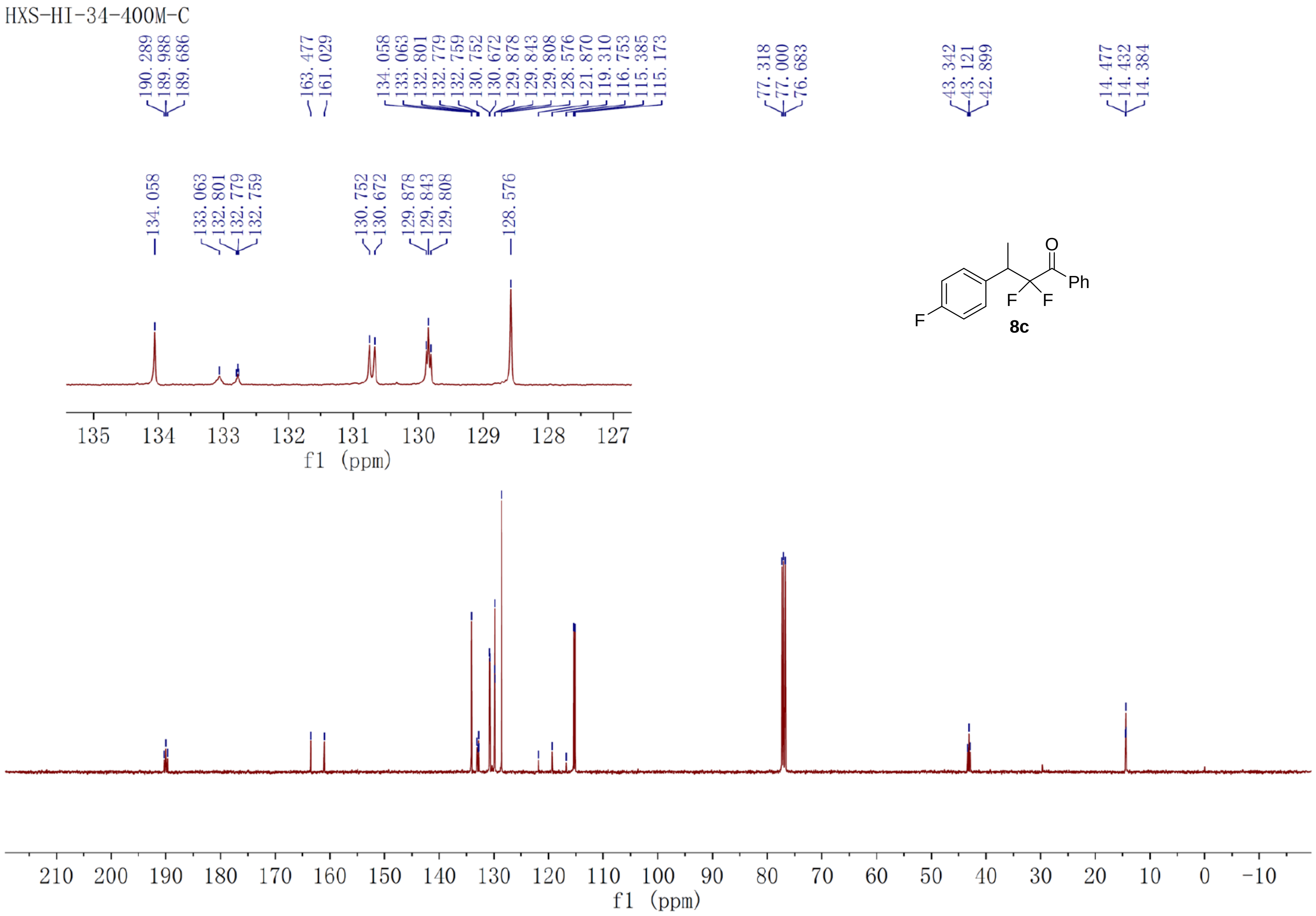
8c

Supplementary Figure 180. ^{13}C NMR (100 MHz, $CDCl_3$) spectra for compound **8c**

HXS-HI-34-400M-F

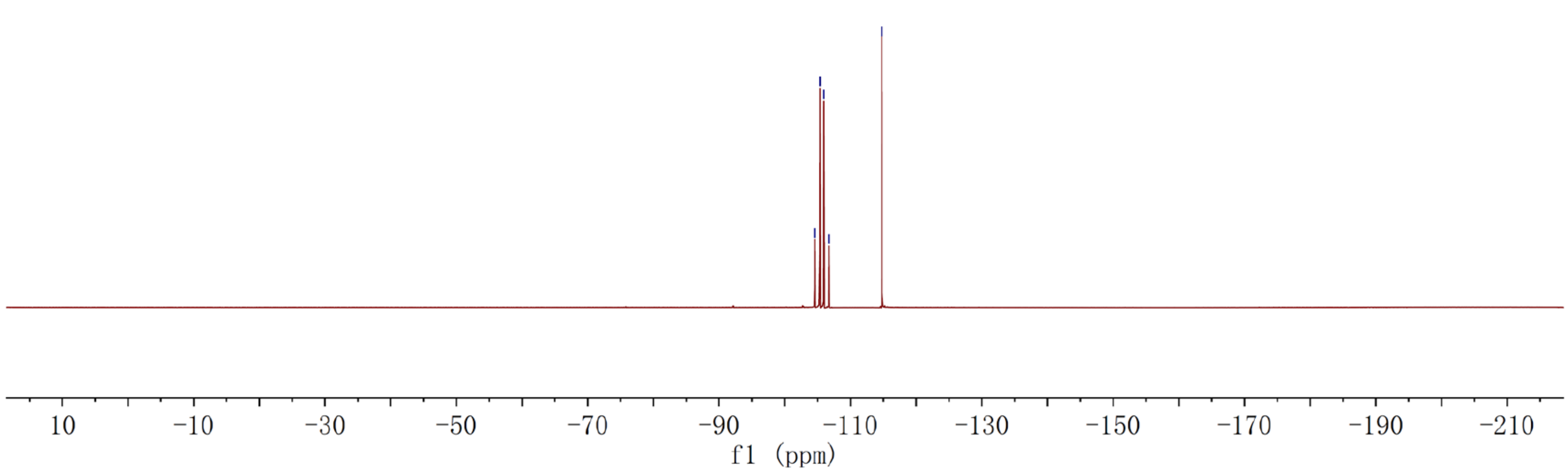

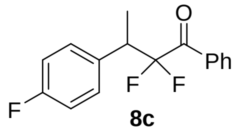

8c

Supplementary Figure 181. ^{19}F NMR (376 MHz, $CDCl_3$) spectra for compound **8c**

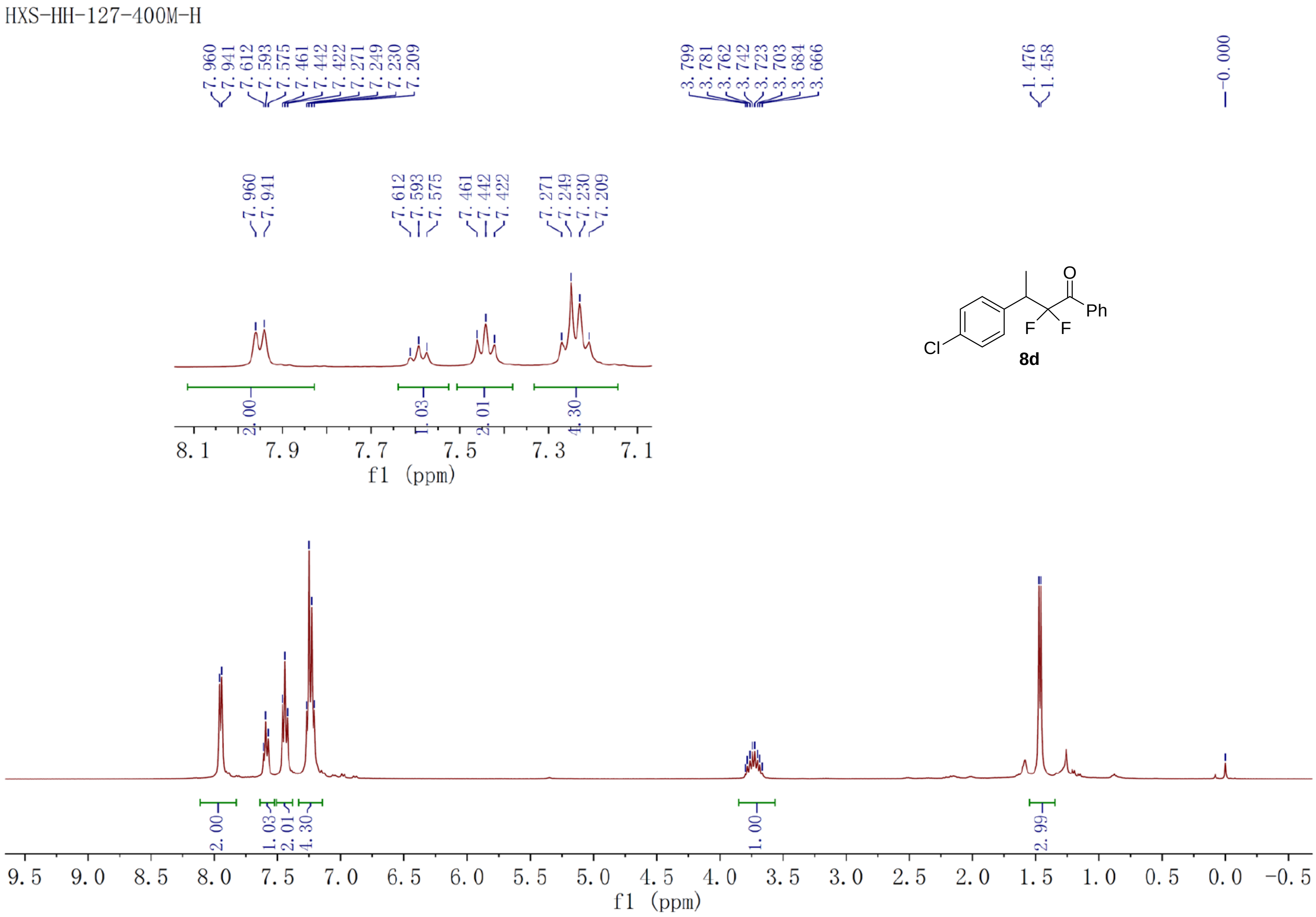

Supplementary Figure 182. ^{1}H NMR (400 MHz, $CDCl_3$) spectra for compound **8d**

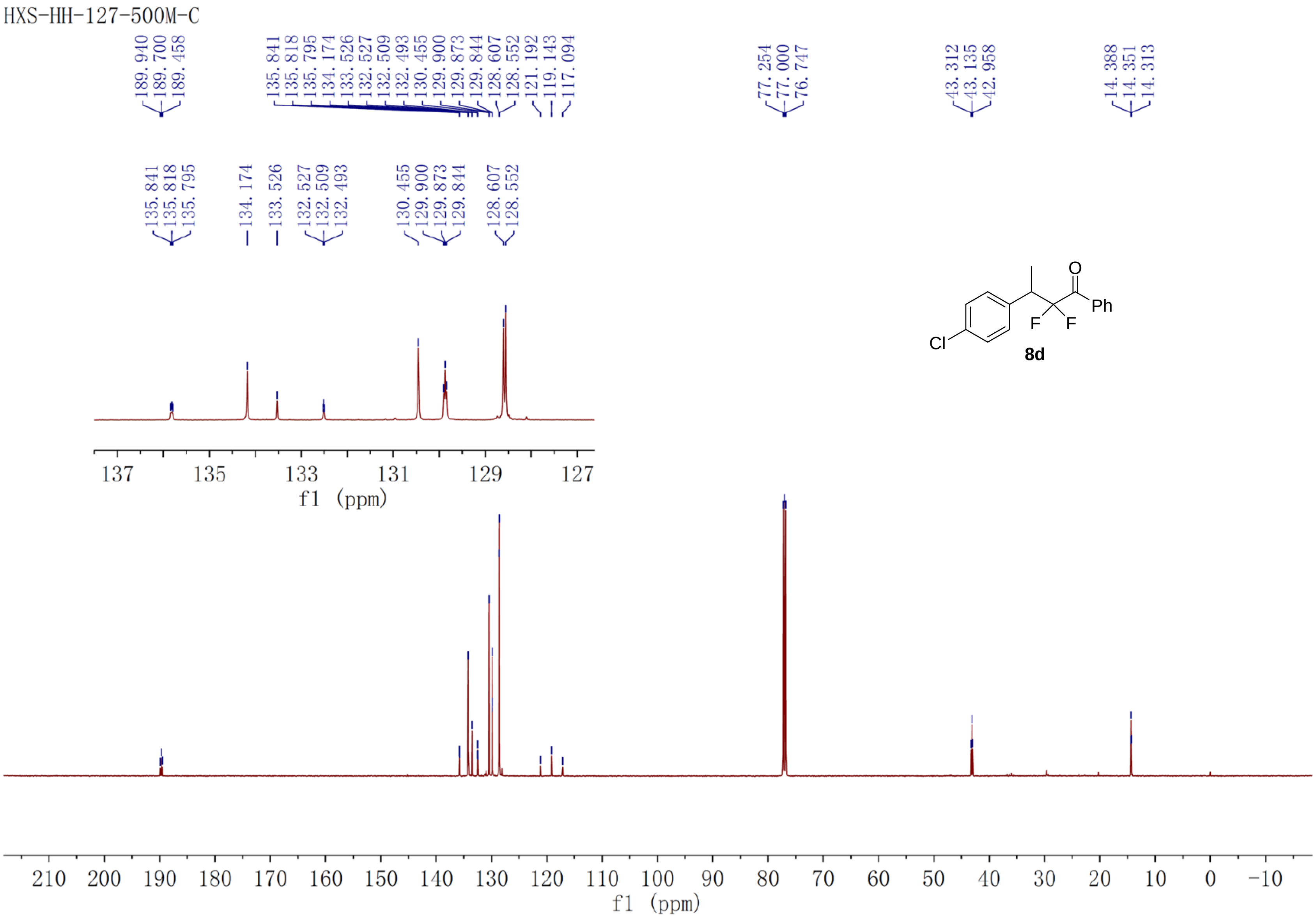

Supplementary Figure 183. ^{13}C NMR (125 MHz, $CDCl_3$) spectra for compound **8d**

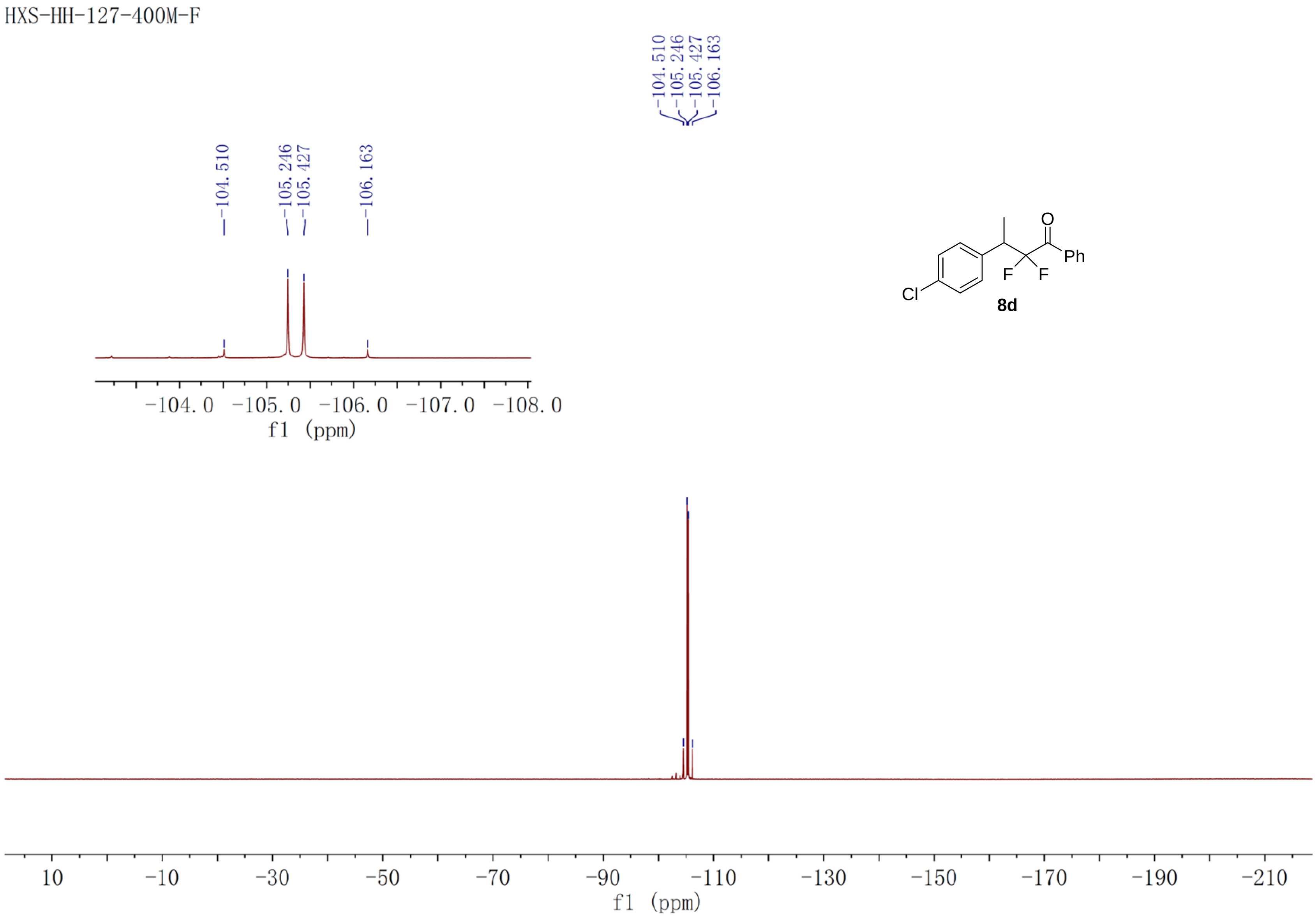

8d

Supplementary Figure 184. ^{19}F NMR (376 MHz, $CDCl_3$) spectra for compound **8d**

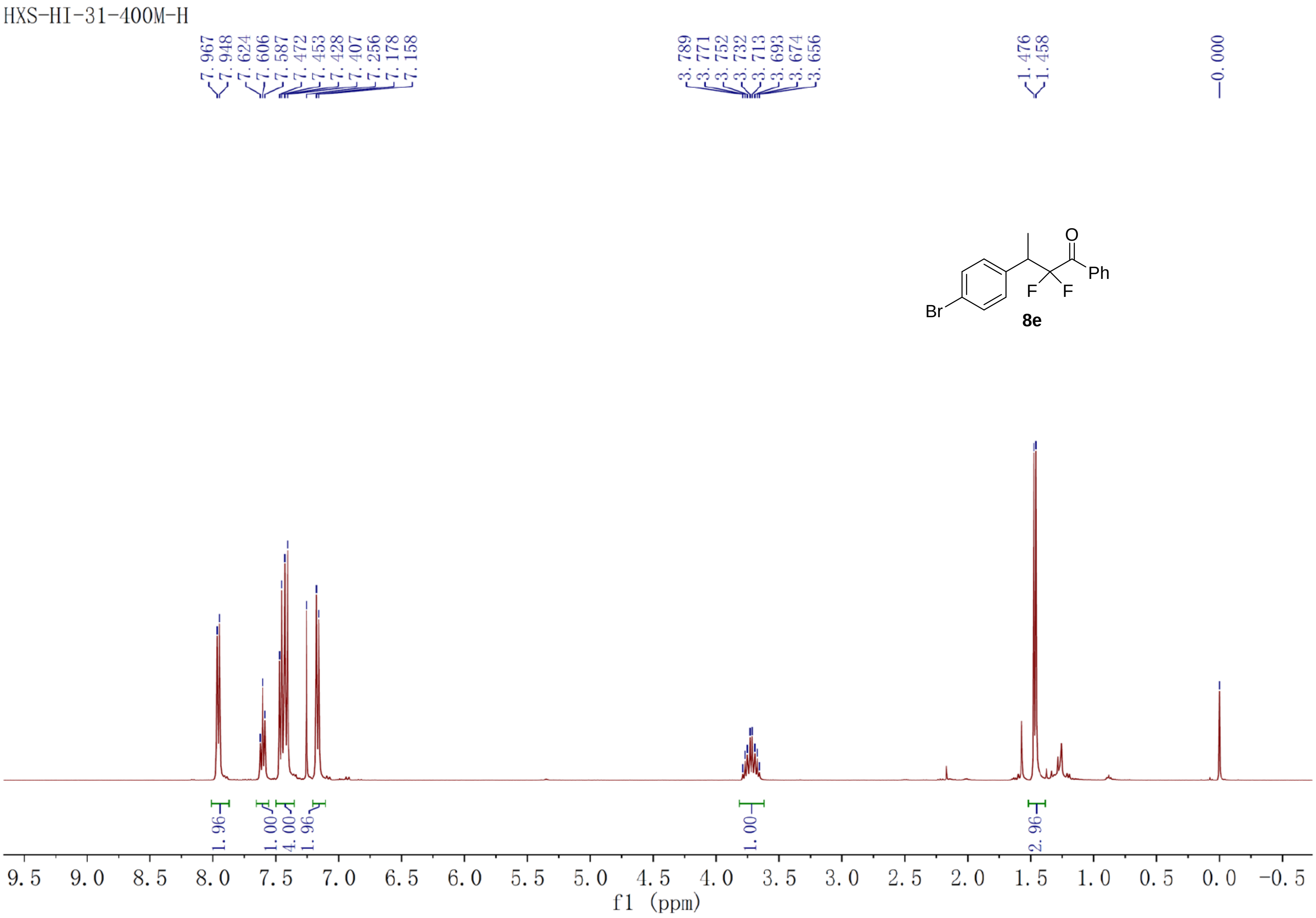

Supplementary Figure 185. ^{1}H NMR (400 MHz, $CDCl_3$) spectra for compound **8e**

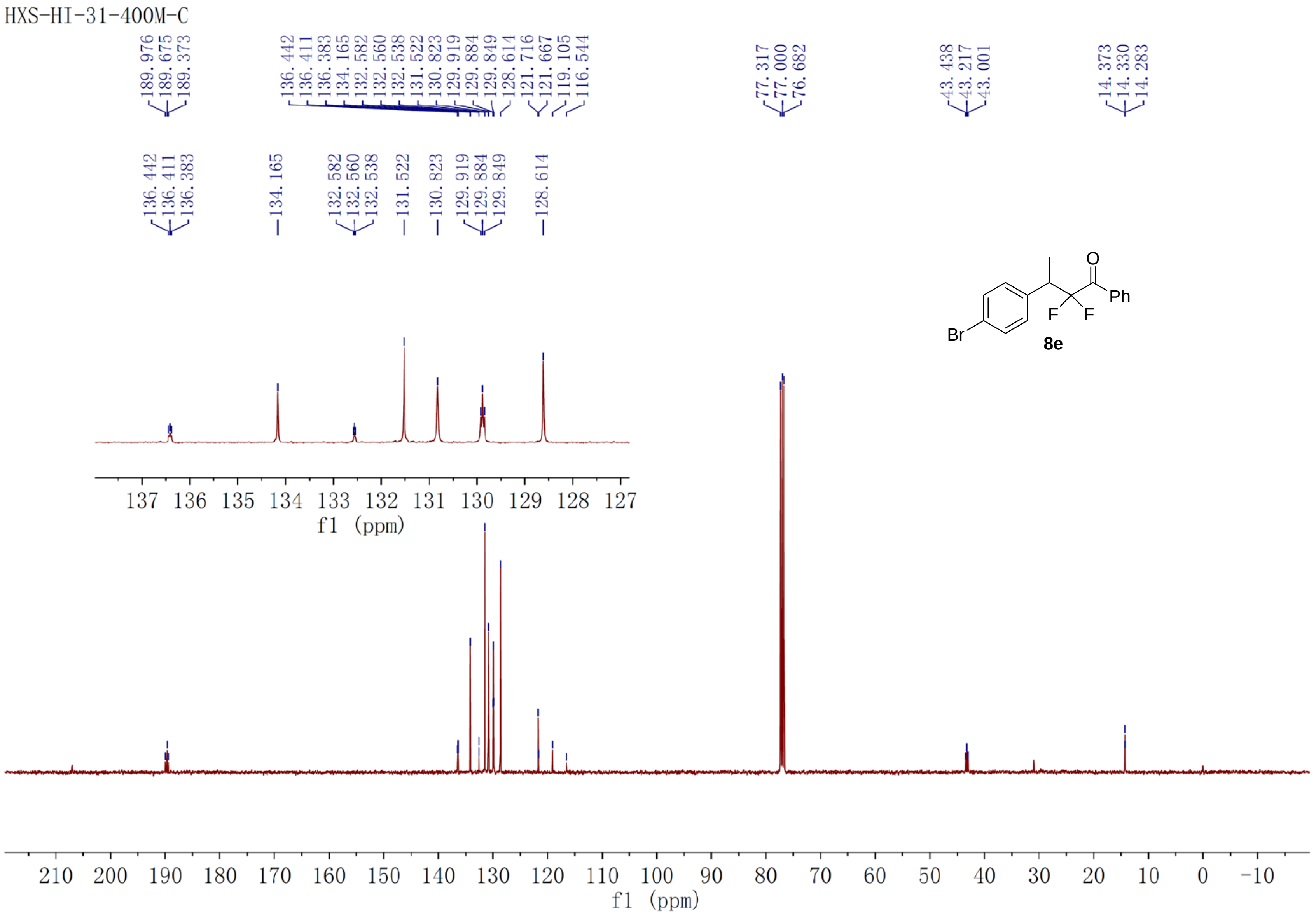

Supplementary Figure 186. ^{13}C NMR (100 MHz, $CDCl_3$) spectra for compound **8e**

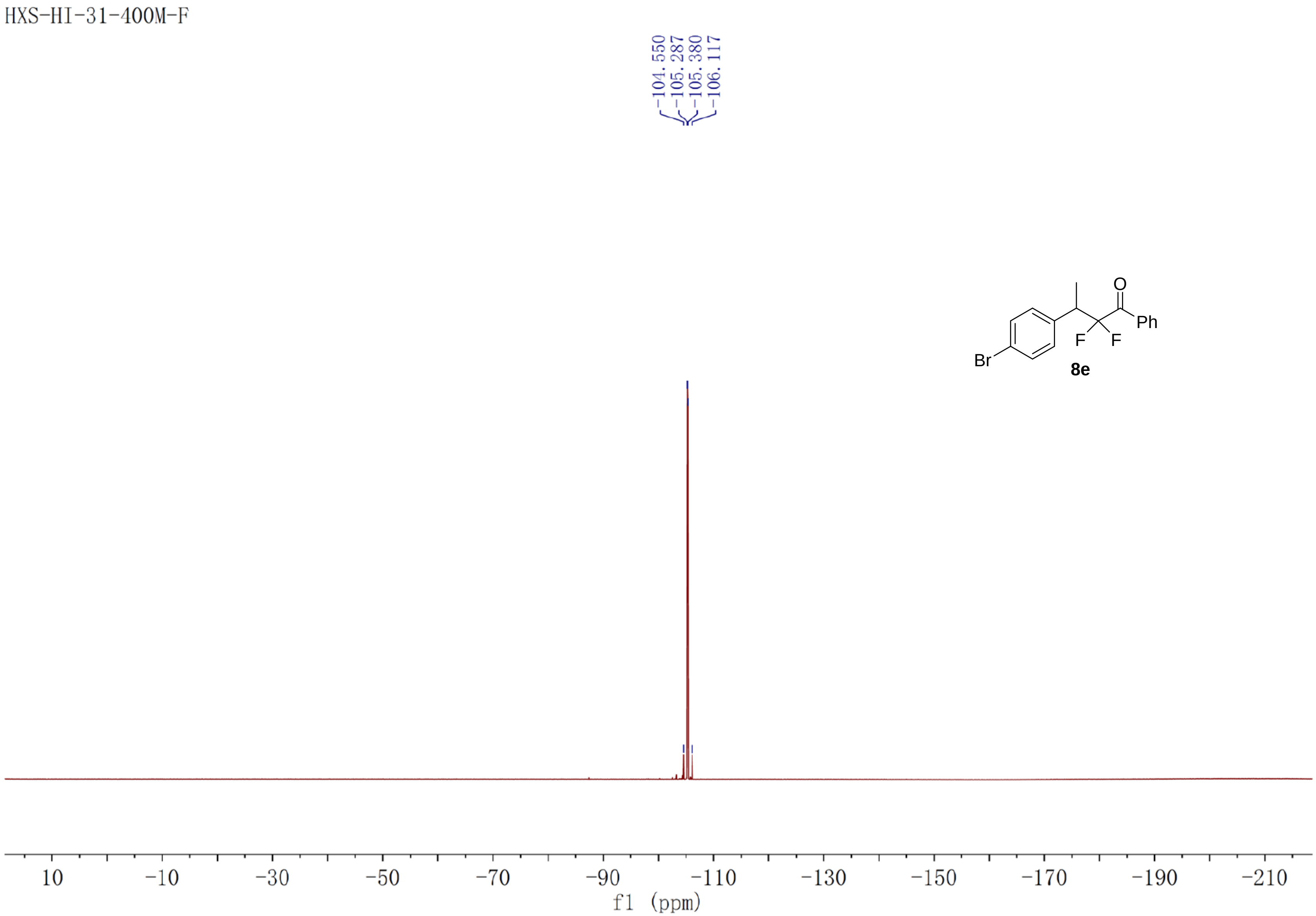

Supplementary Figure 187. ^{19}F NMR (376 MHz, $CDCl_3$) spectra for compound **8e**

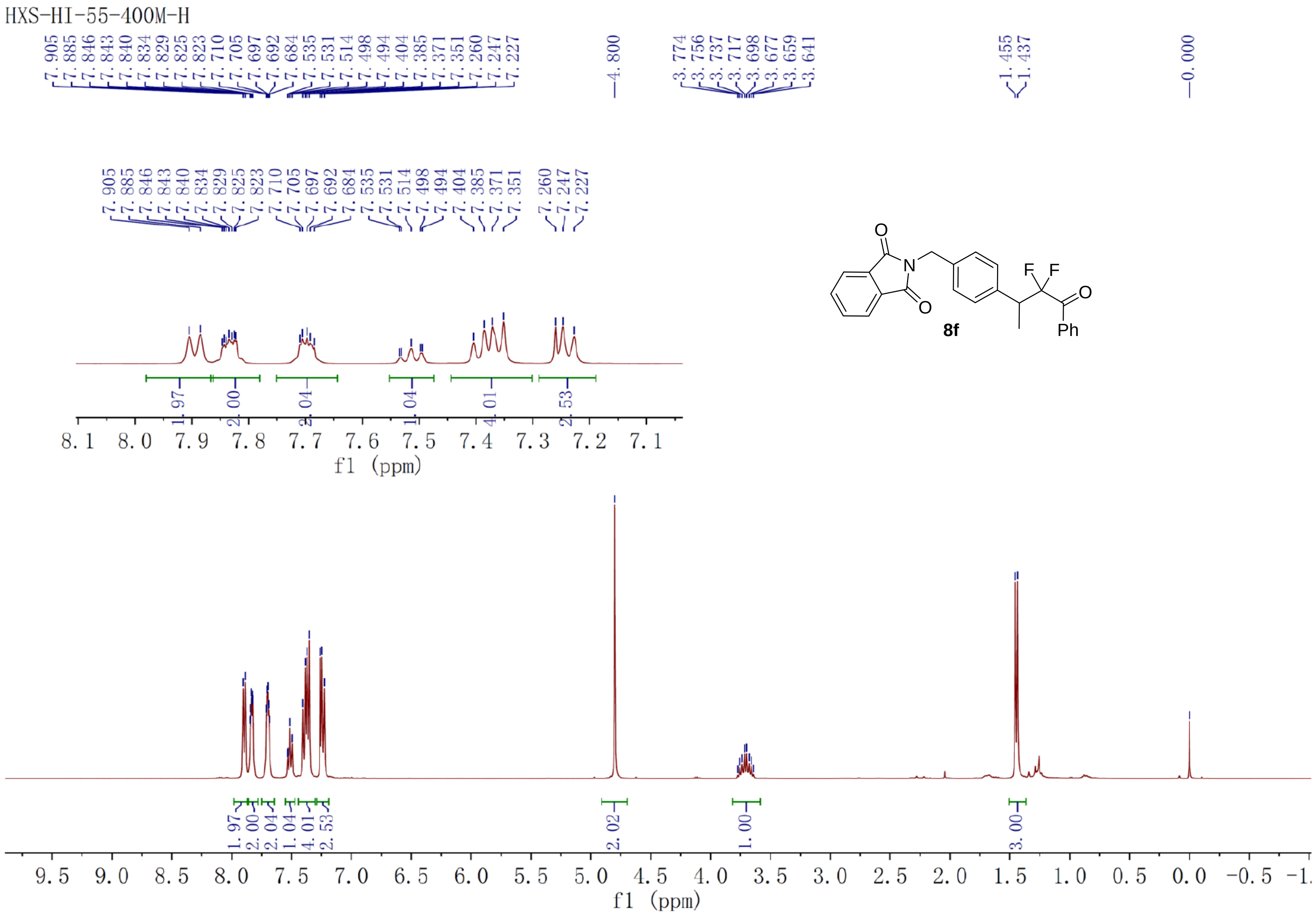

Supplementary Figure 188. ^{1}H NMR (400 MHz, $CDCl_3$) spectra for compound **8f**

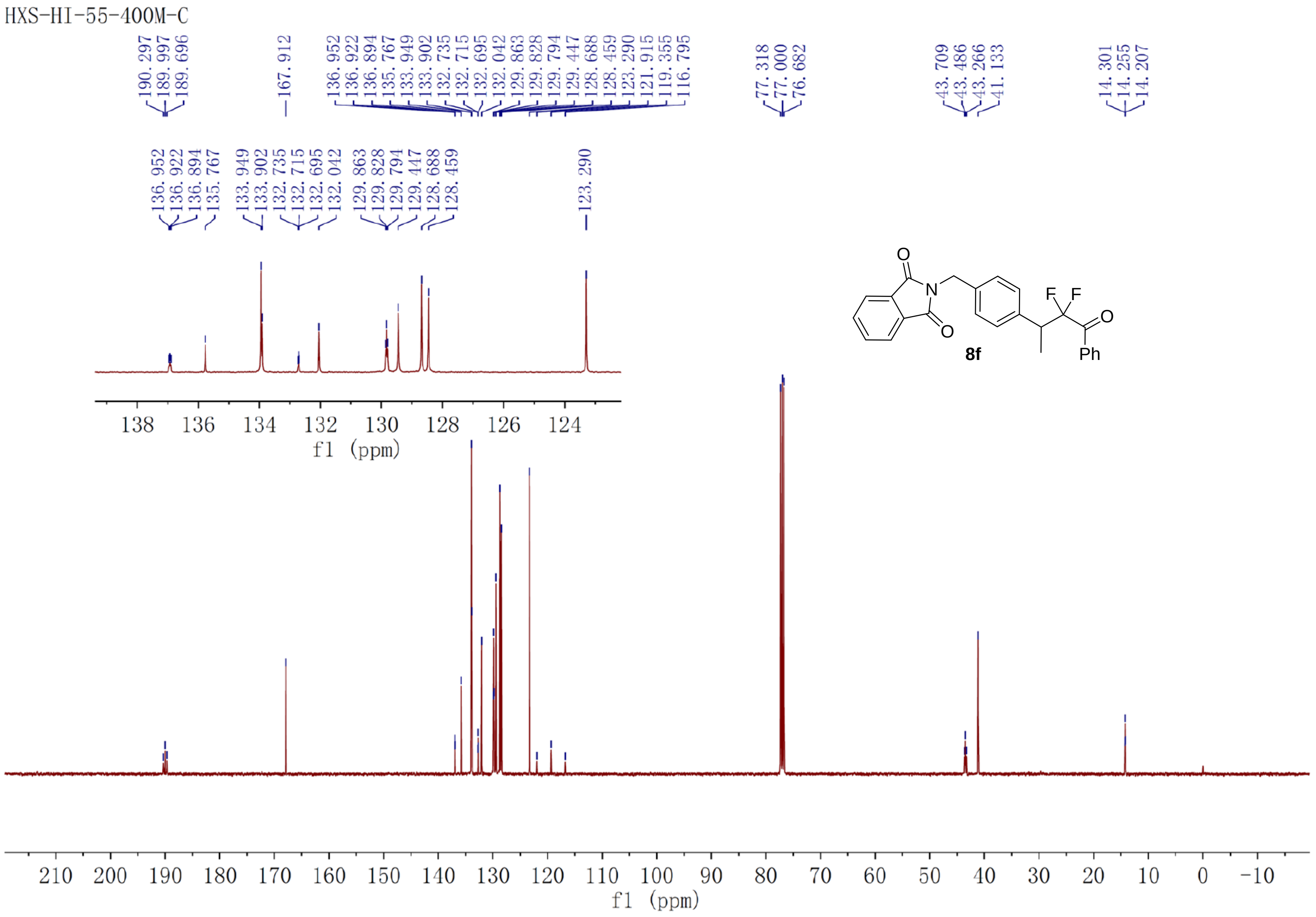

Supplementary Figure 189. ^{13}C NMR (100 MHz, $CDCl_3$) spectra for compound **8f**

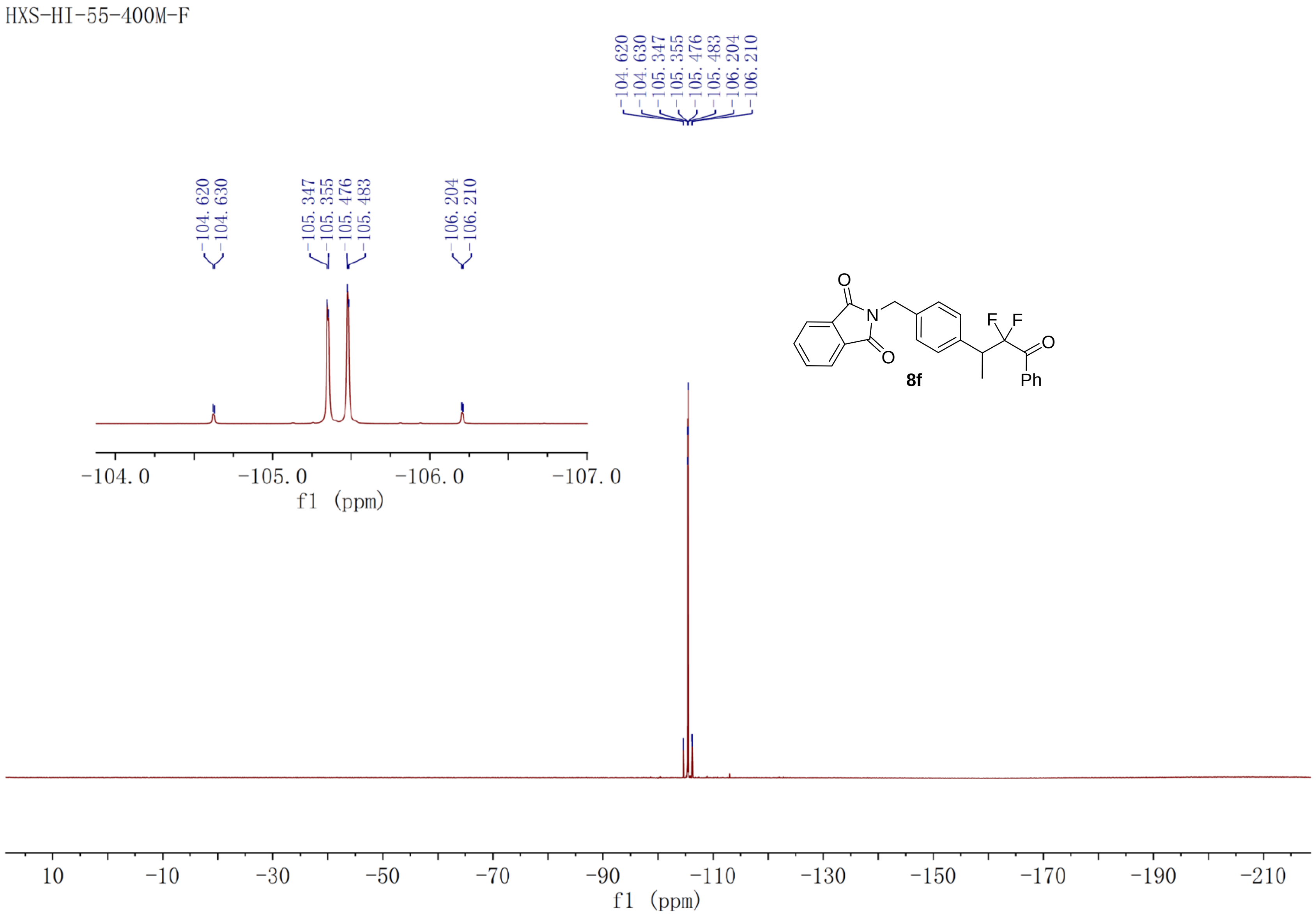

Supplementary Figure 190. ^{19}F NMR (376 MHz, $CDCl_3$) spectra for compound **8f**

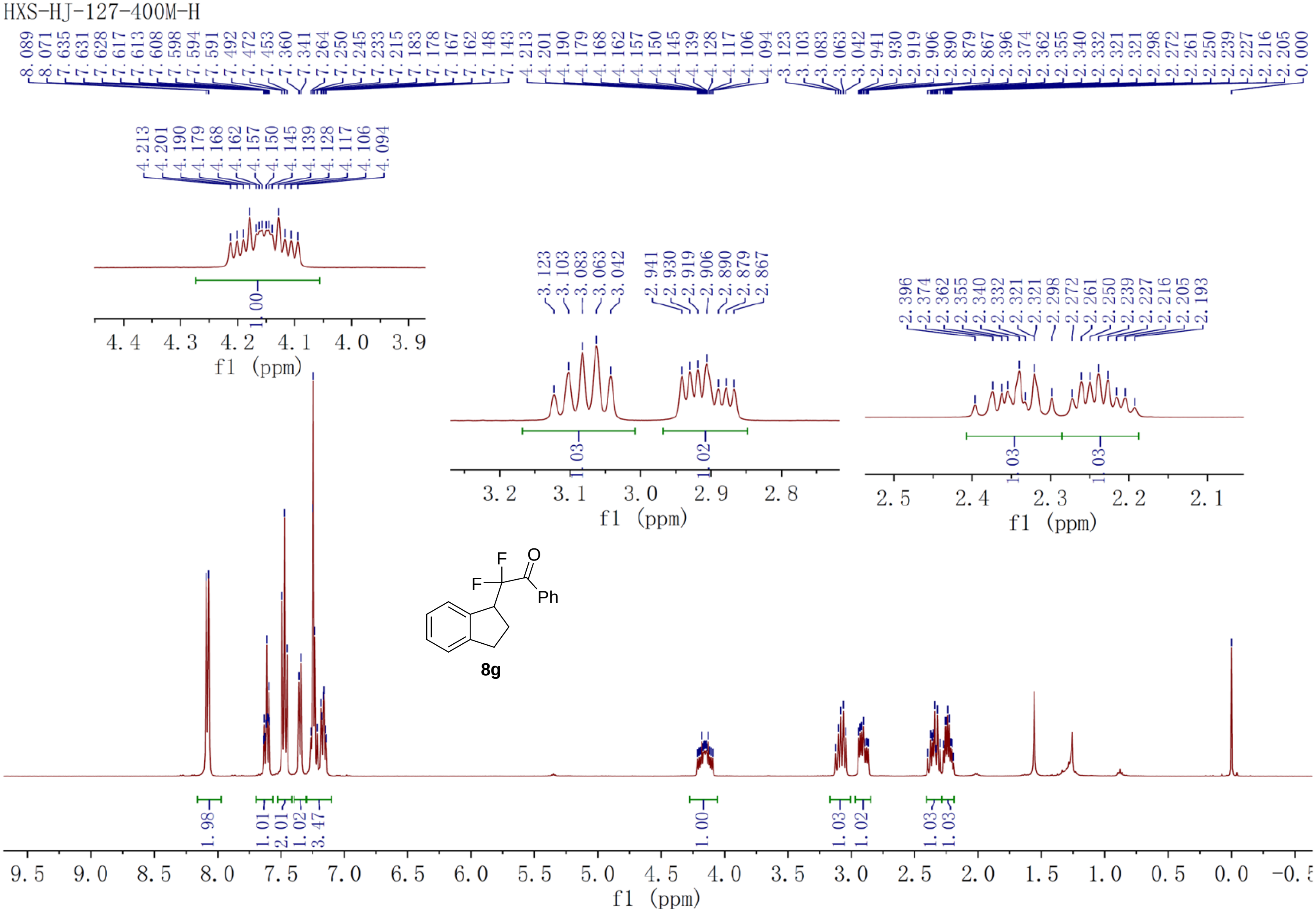

Supplementary Figure 191. ^{1}H NMR (400 MHz, $CDCl_3$) spectra for compound **8g**

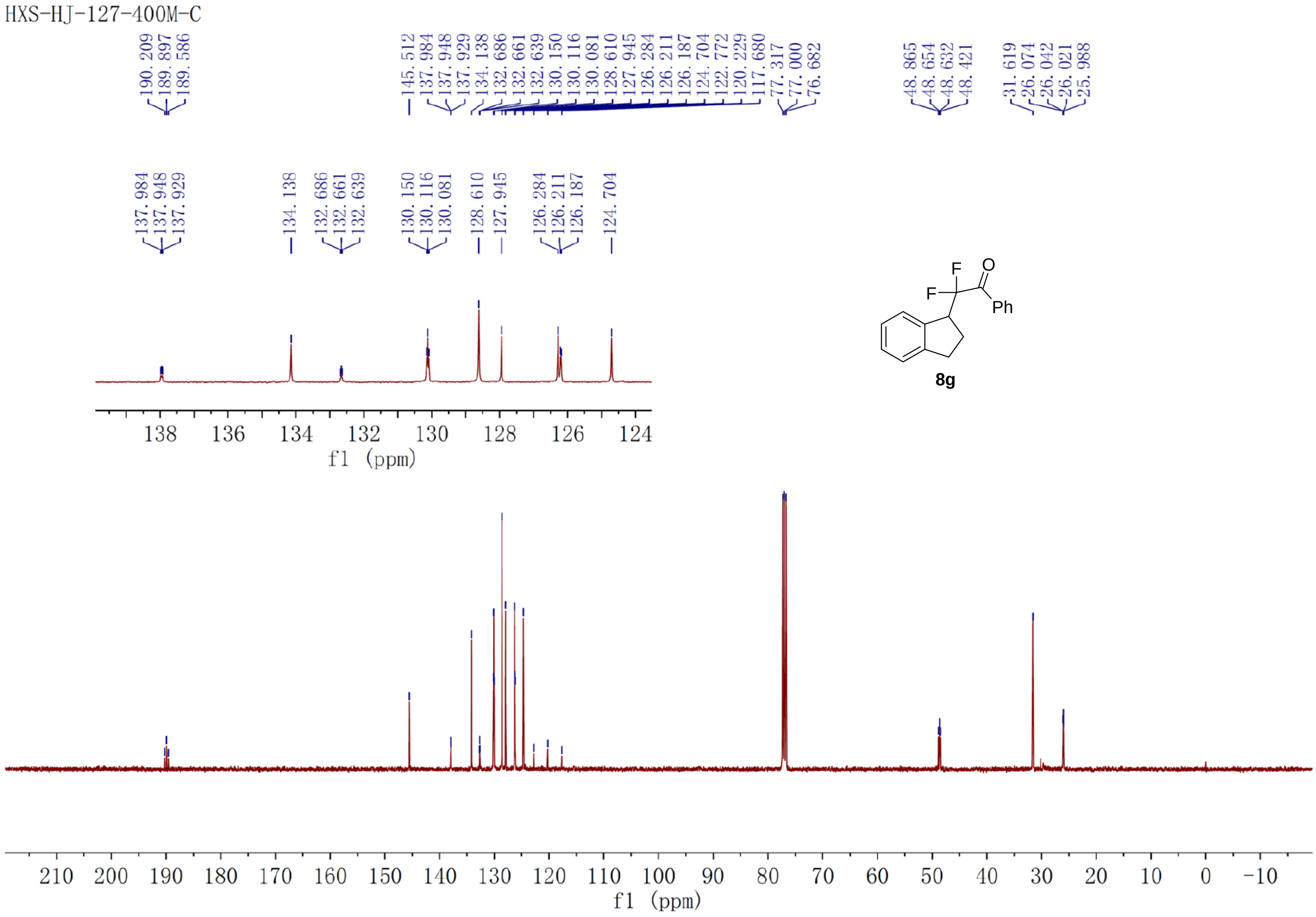

Supplementary Figure 192. ^{13}C NMR (100 MHz, $CDCl_3$) spectra for compound **8g**

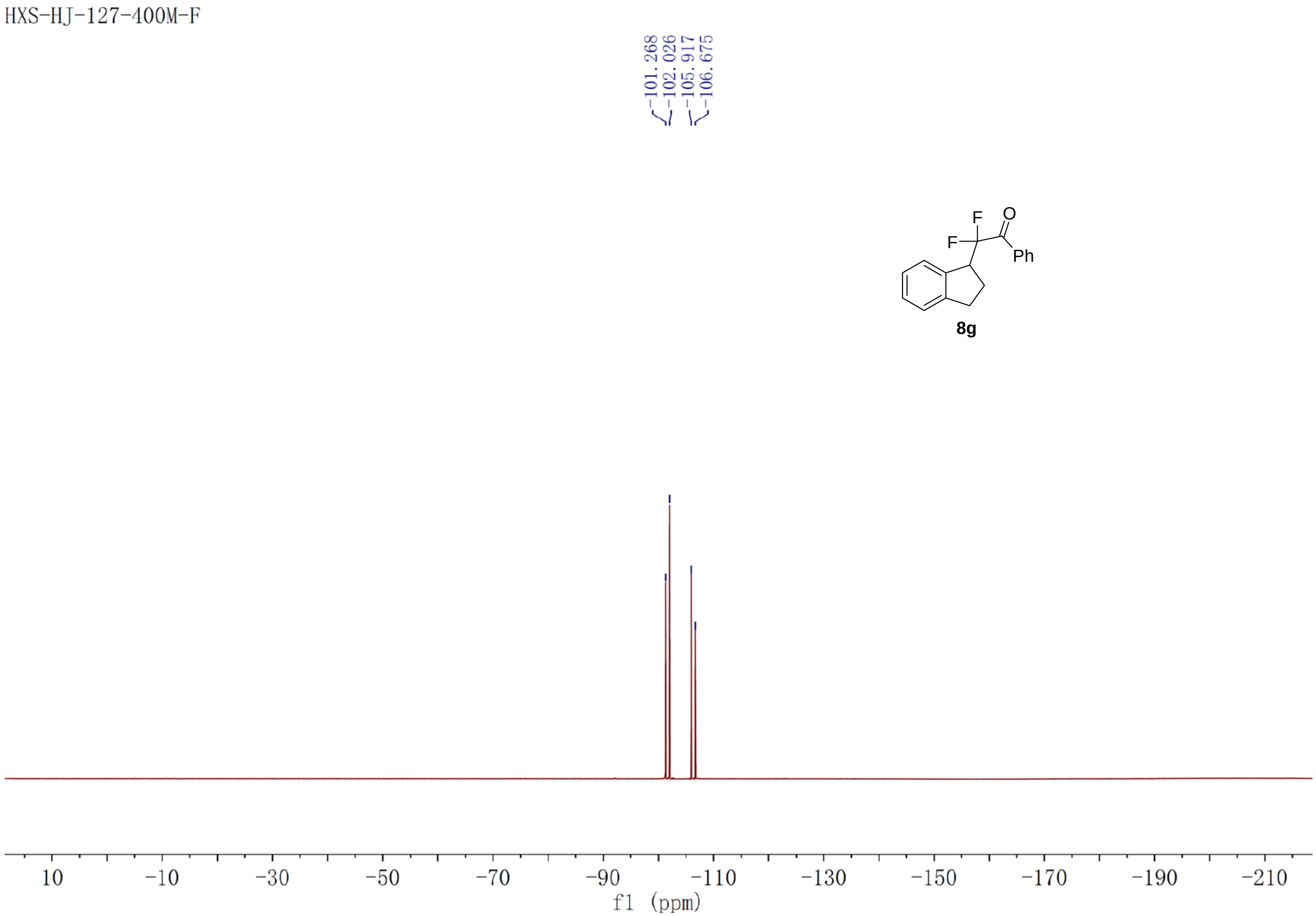

Supplementary Figure 193. ^{19}F NMR (376 MHz, $CDCl_3$) spectra for compound **8g**

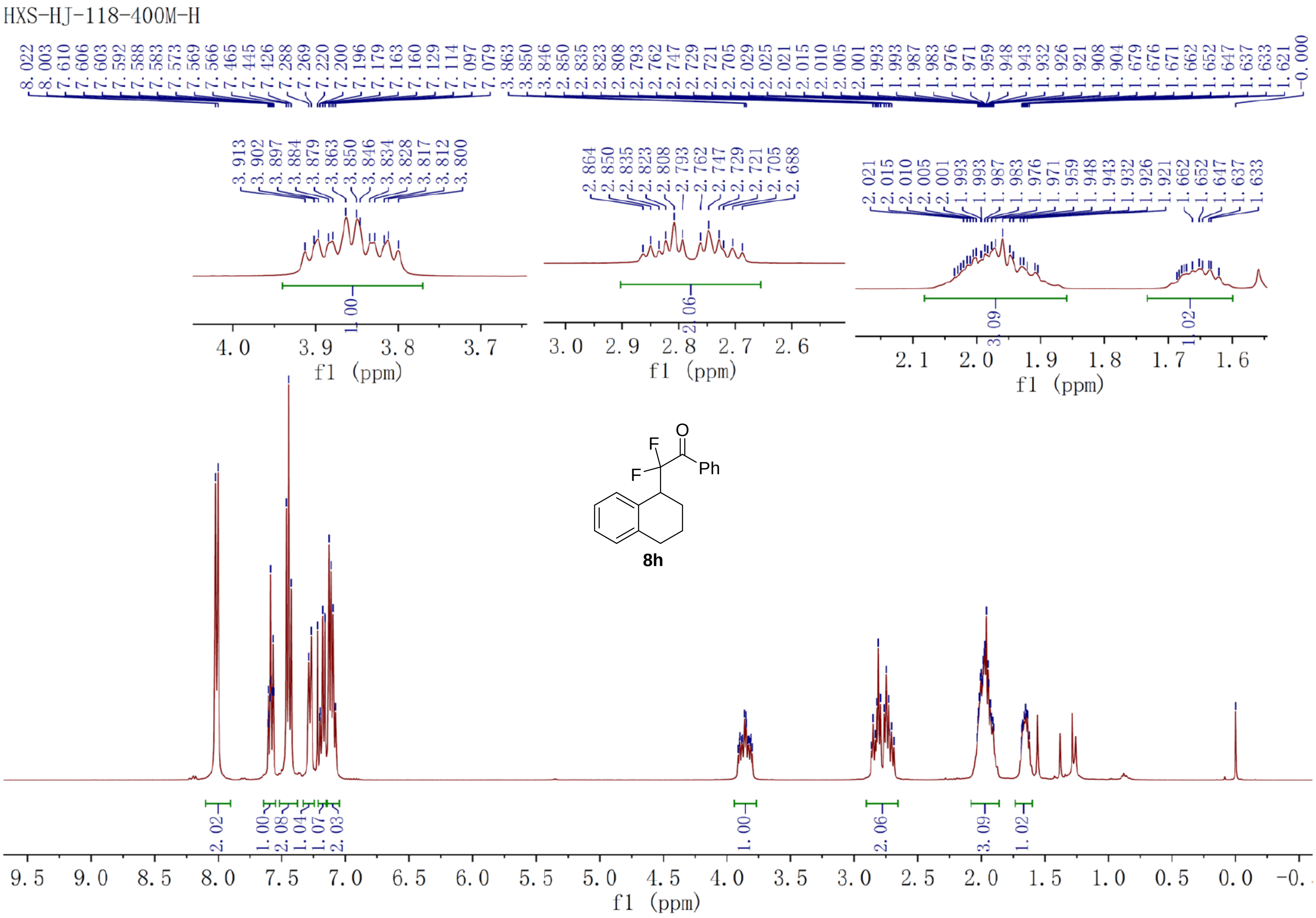

Supplementary Figure 194. ^{1}H NMR (400 MHz, $CDCl_3$) spectra for compound **8h**

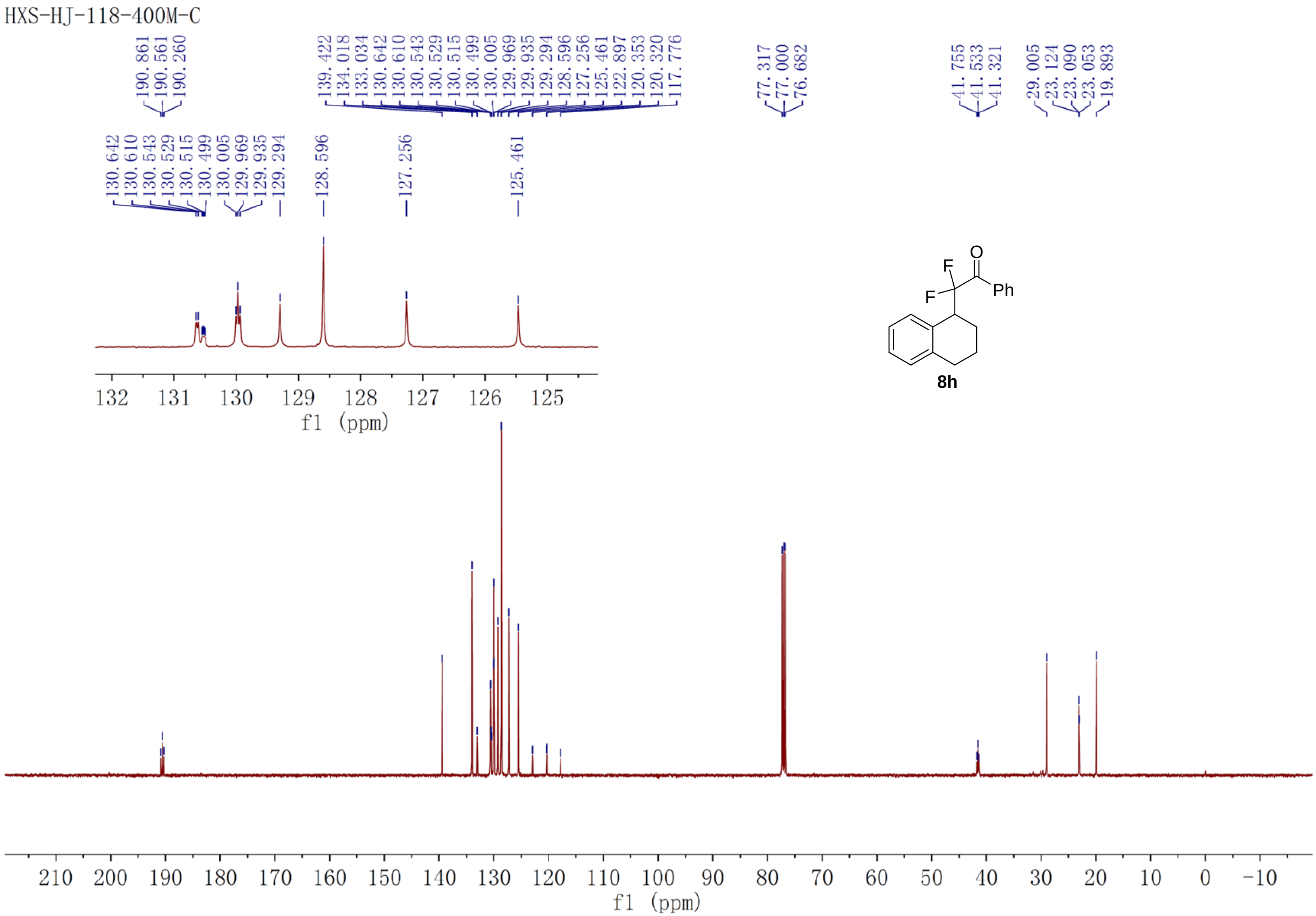

Supplementary Figure 195. ^{13}C NMR (100 MHz, $CDCl_3$) spectra for compound **8h**

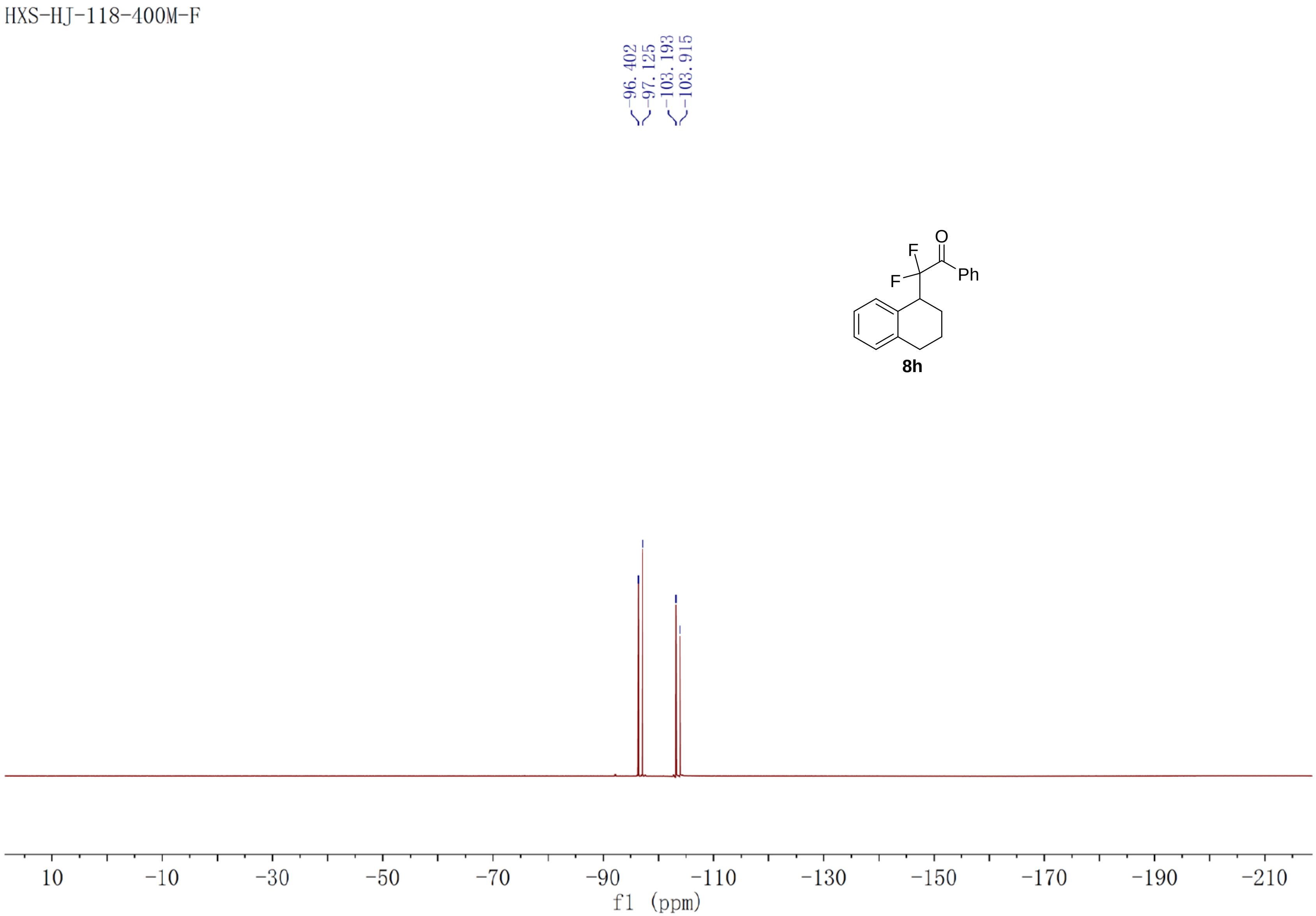

Supplementary Figure 196. ^{19}F NMR (376 MHz, $CDCl_3$) spectra for compound **8h**

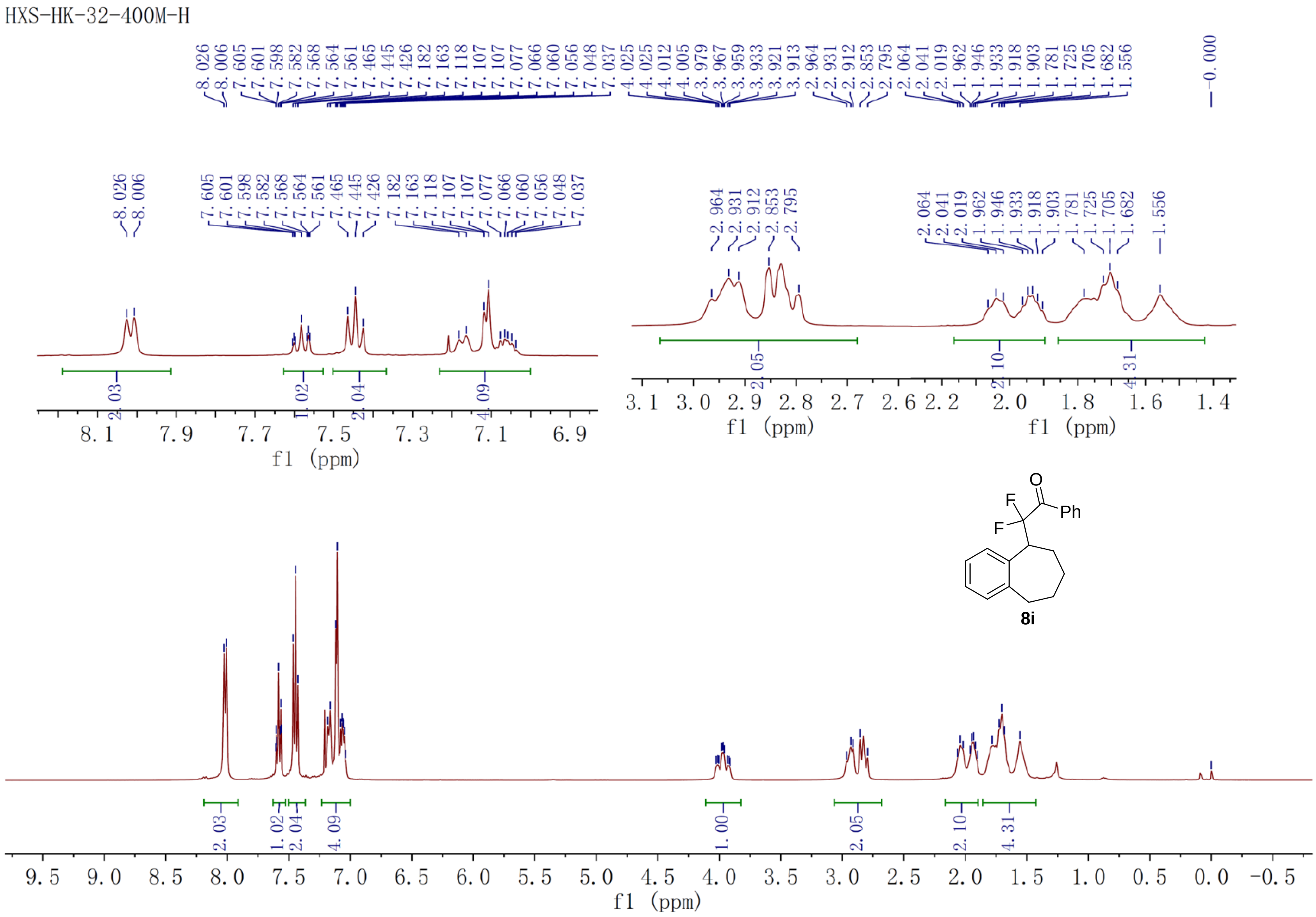

Supplementary Figure 197. ^{1}H NMR (400 MHz, $CDCl_3$) spectra for compound **8i**

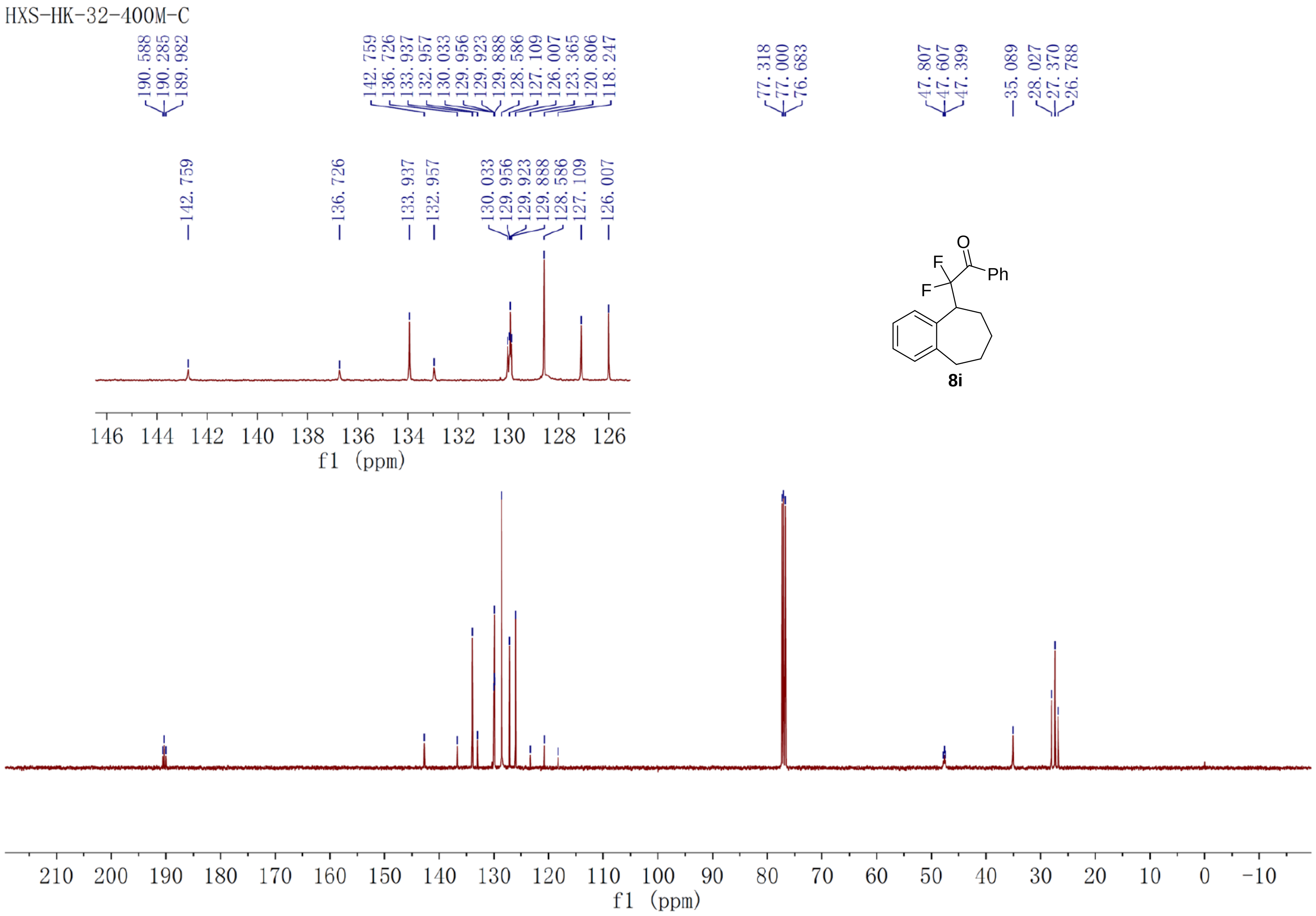

Supplementary Figure 198. ^{13}C NMR (100 MHz, $CDCl_3$) spectra for compound **8i**

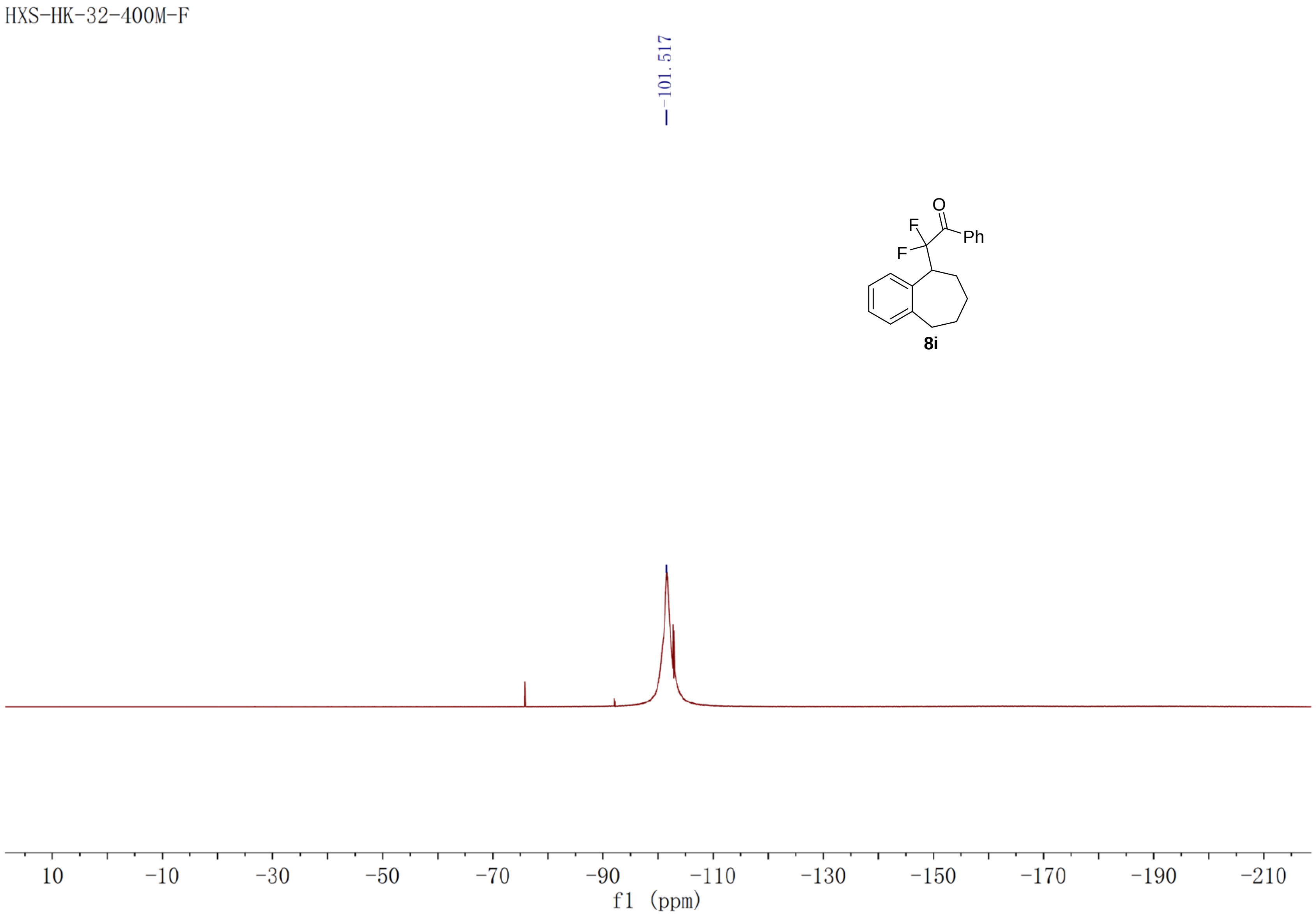

Supplementary Figure 199. ^{19}F NMR (376 MHz, $CDCl_3$) spectra for compound **8i**

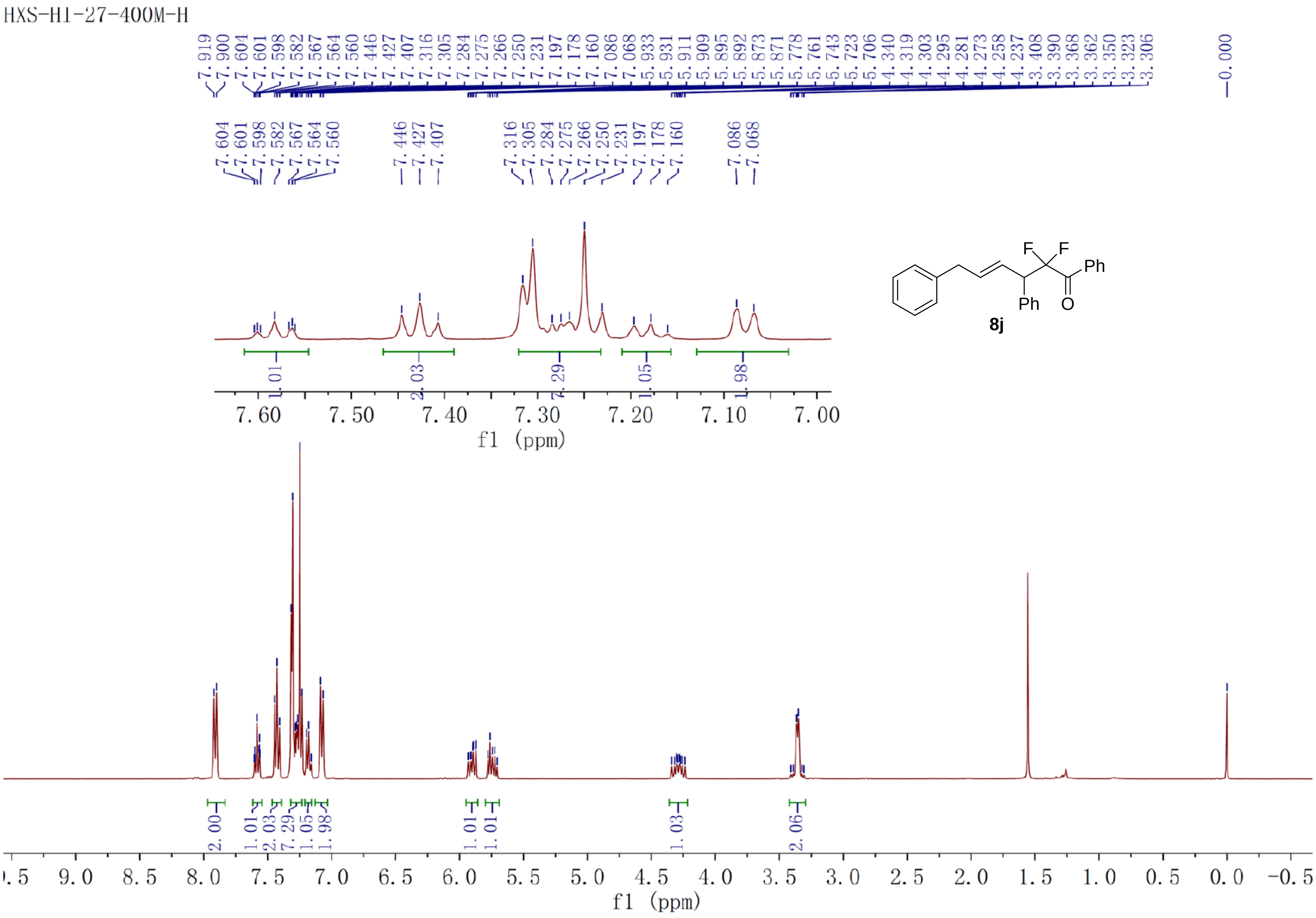

Supplementary Figure 200. ^{1}H NMR (400 MHz, $CDCl_3$) spectra for compound **8j**

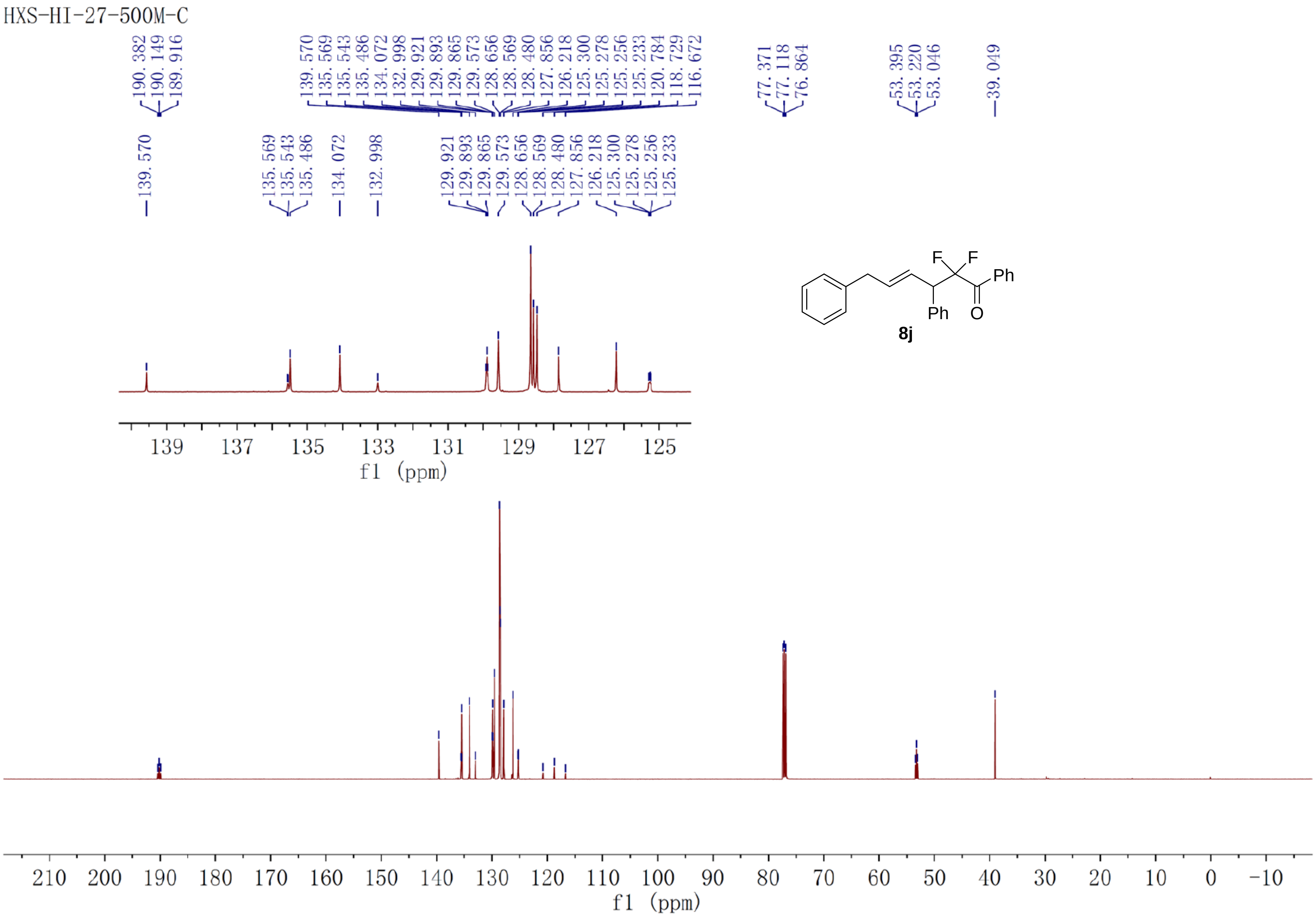

Supplementary Figure 201. ^{13}C NMR (125 MHz, $CDCl_3$) spectra for compound **8j**

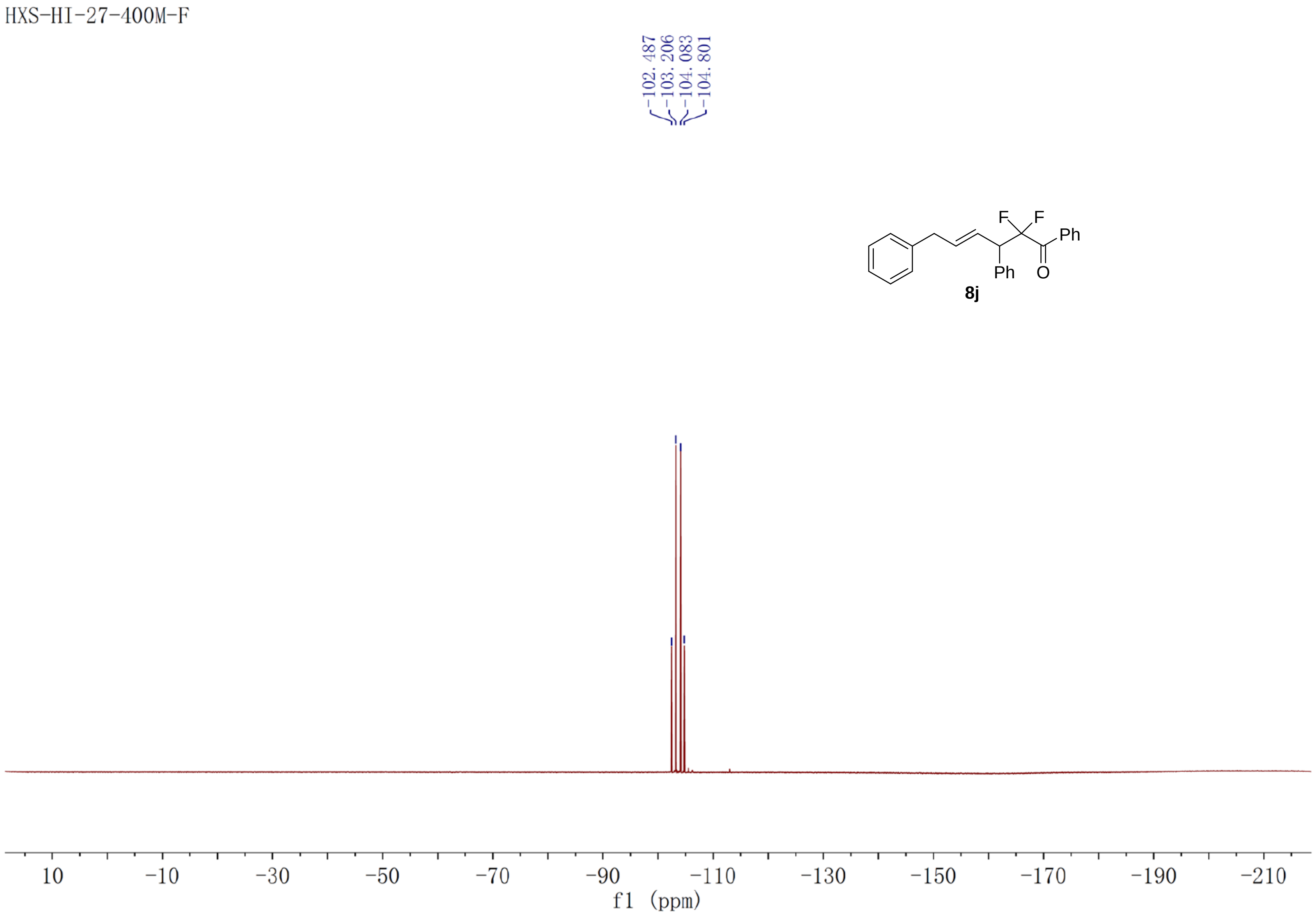

Supplementary Figure 202. ^{19}F NMR (376 MHz, $CDCl_3$) spectra for compound **8j**

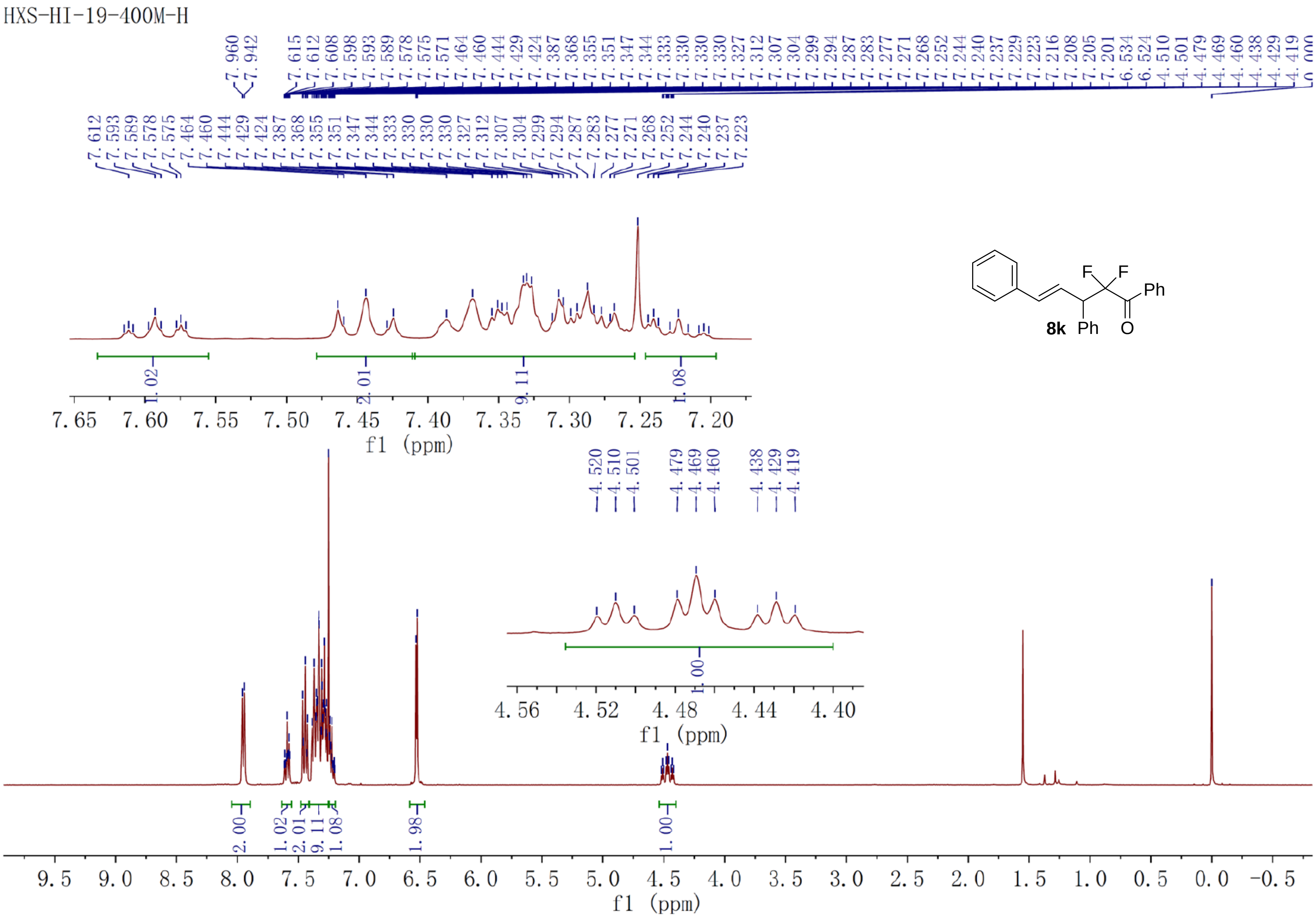

Supplementary Figure 203. ^{1}H NMR (400 MHz, $CDCl_3$) spectra for compound **8k**

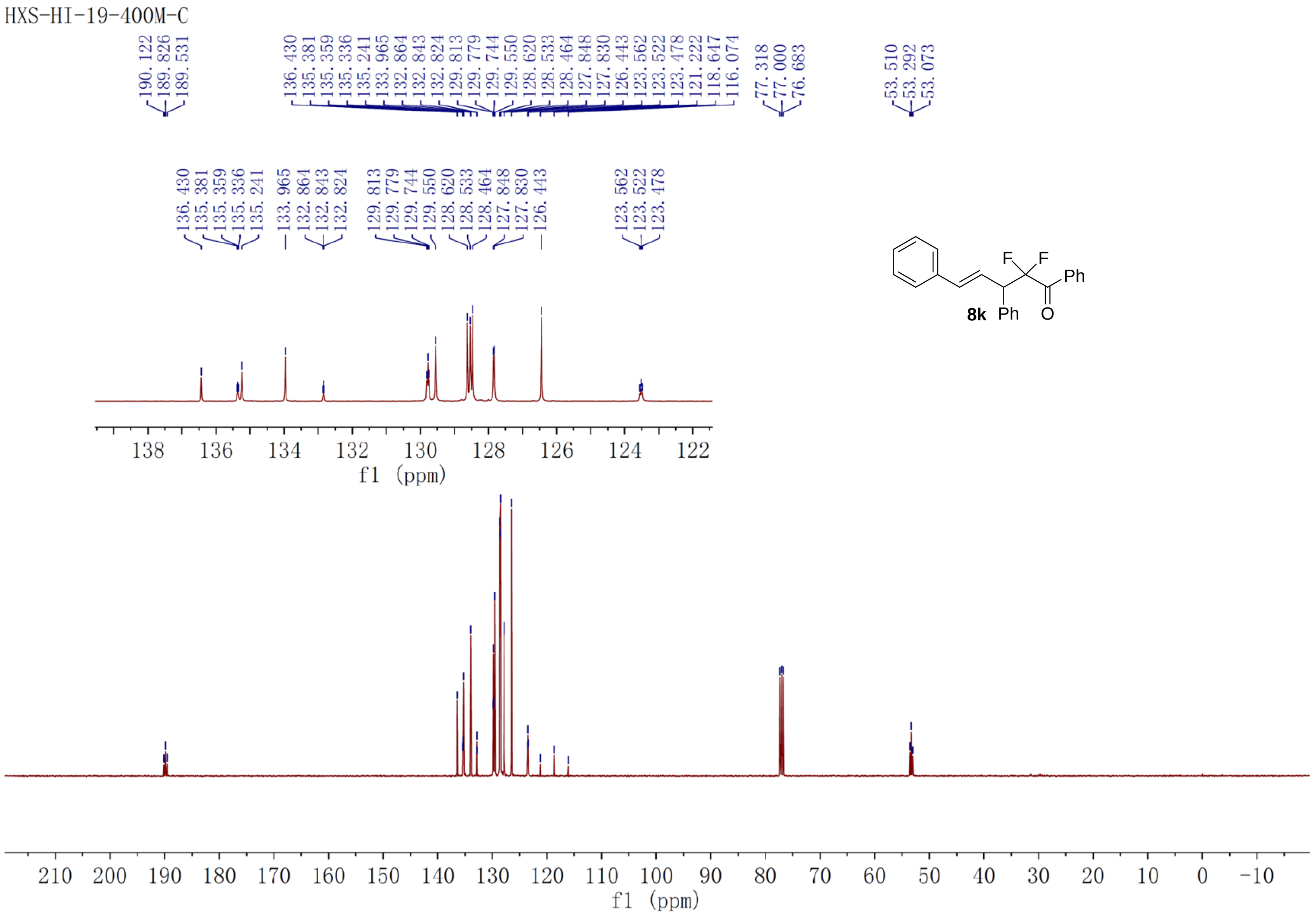

Supplementary Figure 204. ^{13}C NMR (100 MHz, $CDCl_3$) spectra for compound **8k**

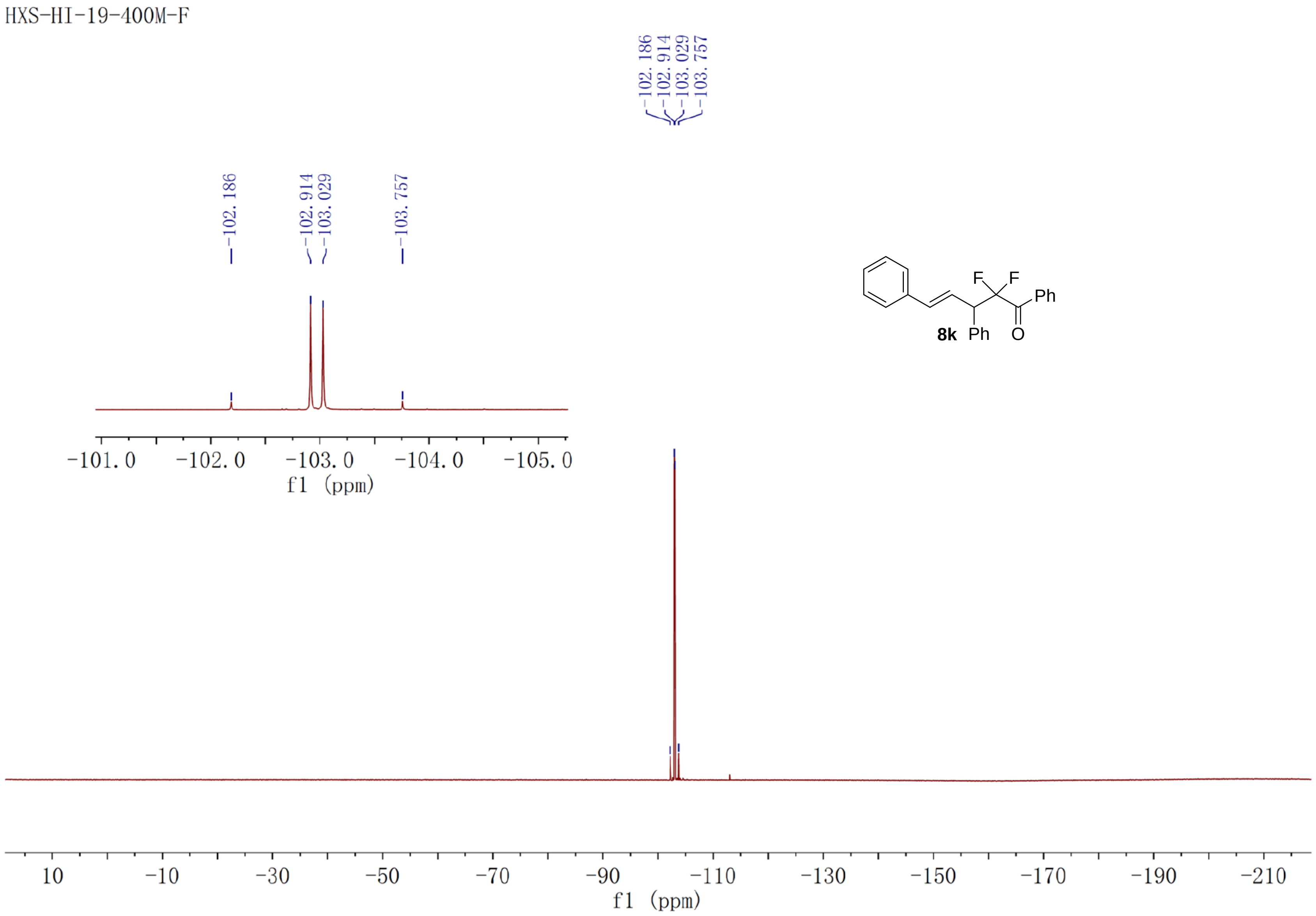

Supplementary Figure 205. ^{19}F NMR (376 MHz, $CDCl_3$) spectra for compound **8k**

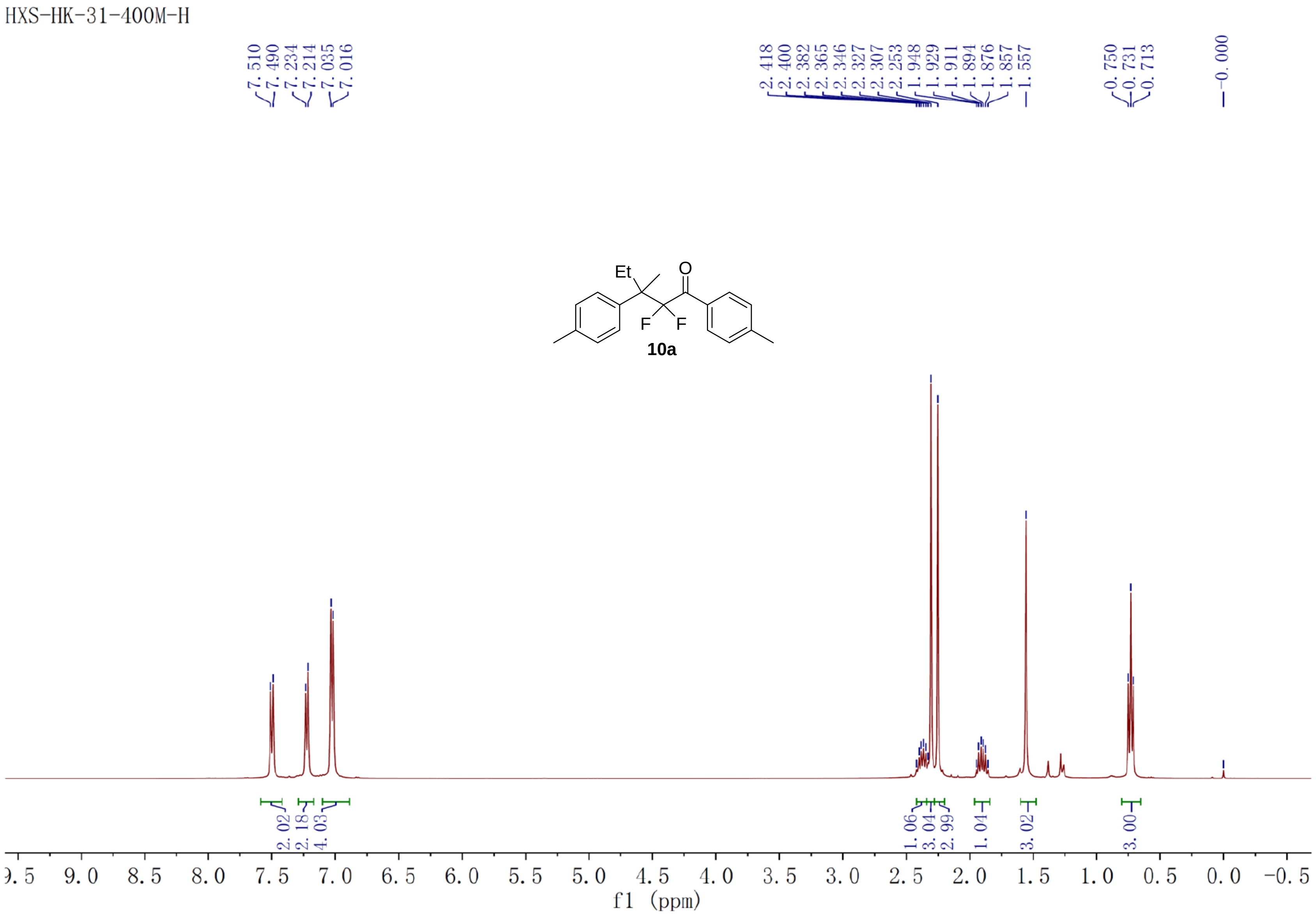

Supplementary Figure 206. ^{1}H NMR (400 MHz, $CDCl_3$) spectra for compound **10a**

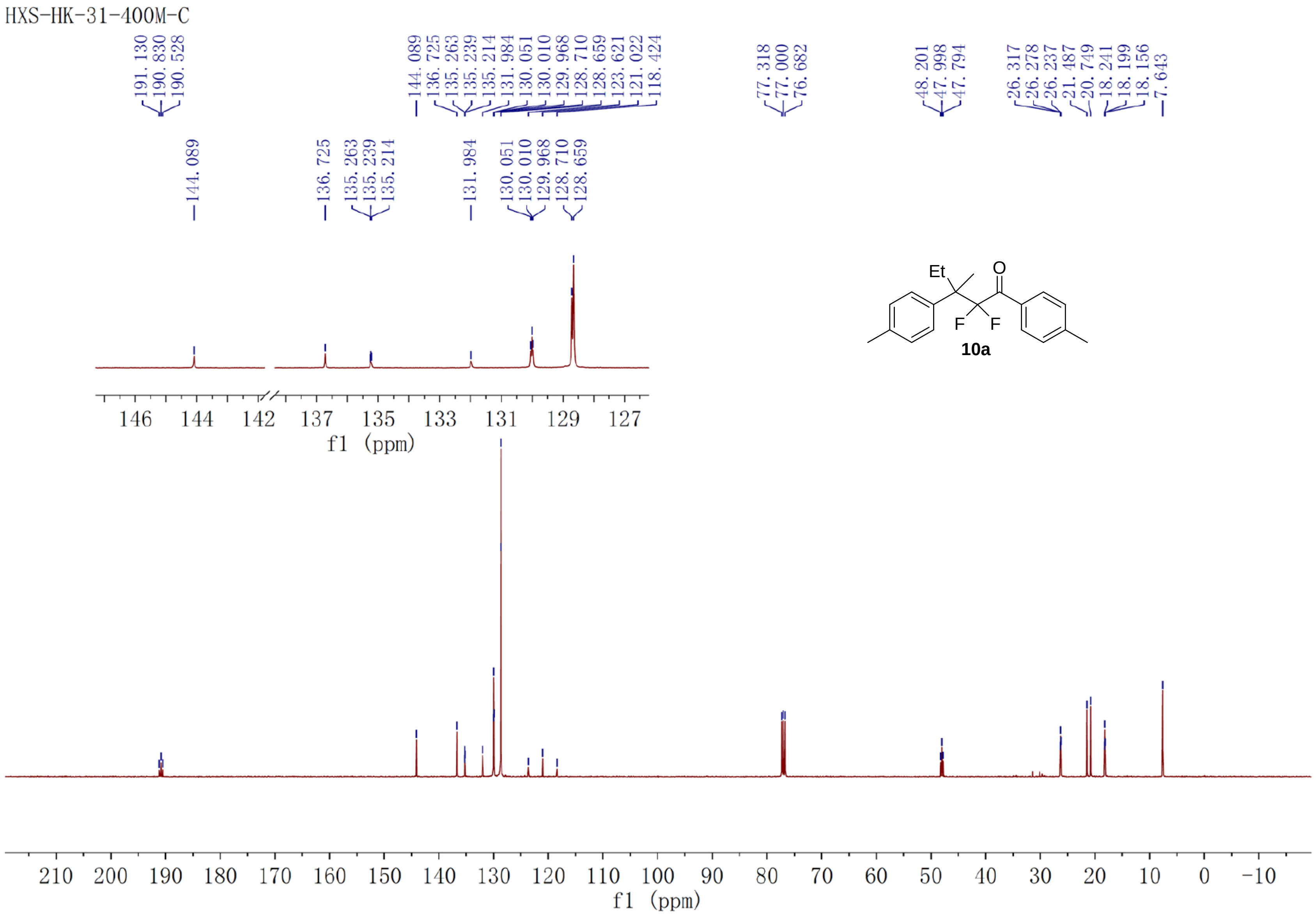

Supplementary Figure 207. ^{13}C NMR (100 MHz, $CDCl_3$) spectra for compound **10a**

HXS-HK-31-400M-F

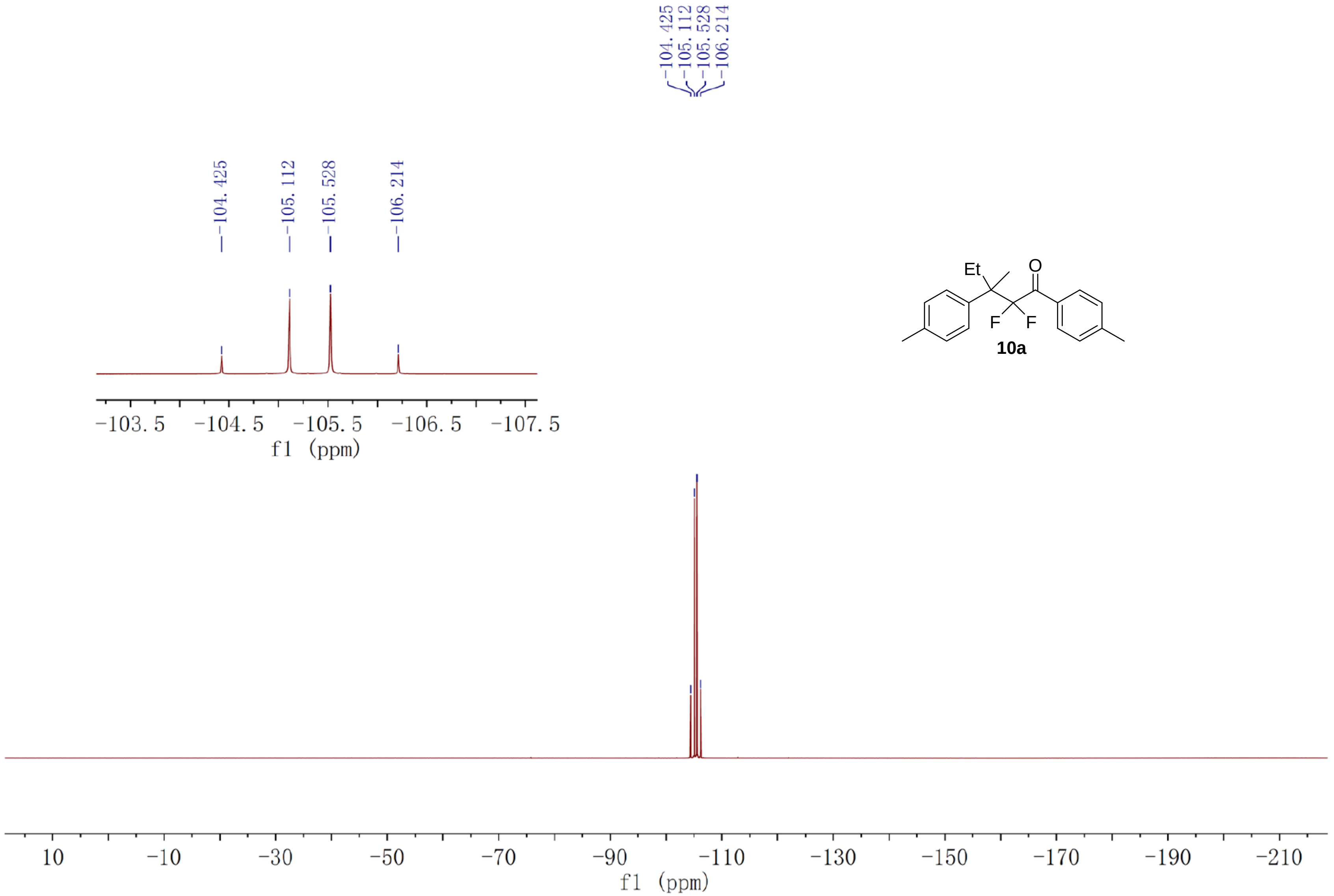

10a

Supplementary Figure 208. ^{19}F NMR (376 MHz, $CDCl_3$) spectra for compound **10a**

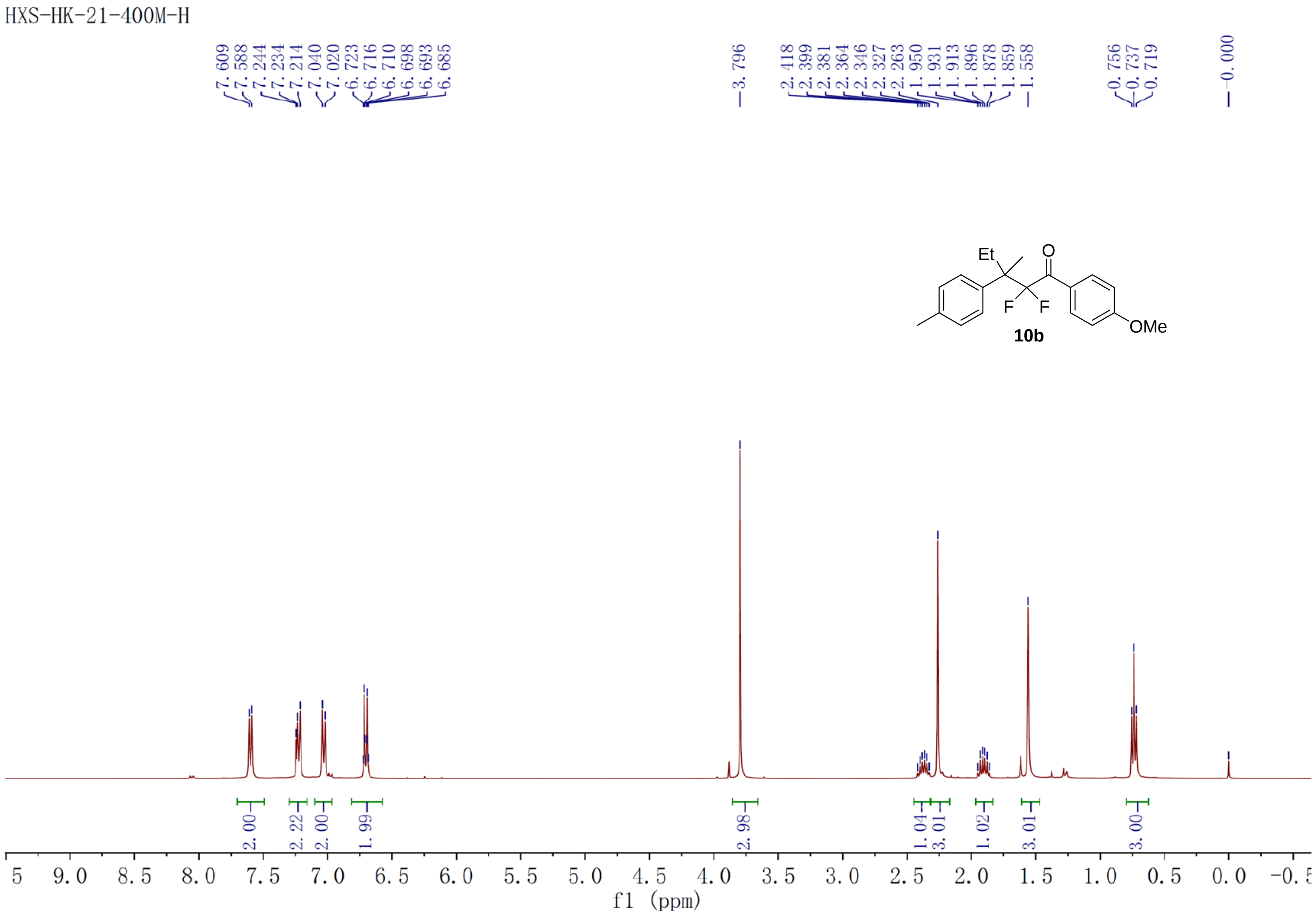

Supplementary Figure 209. ^{1}H NMR (400 MHz, $CDCl_3$) spectra for compound **10b**

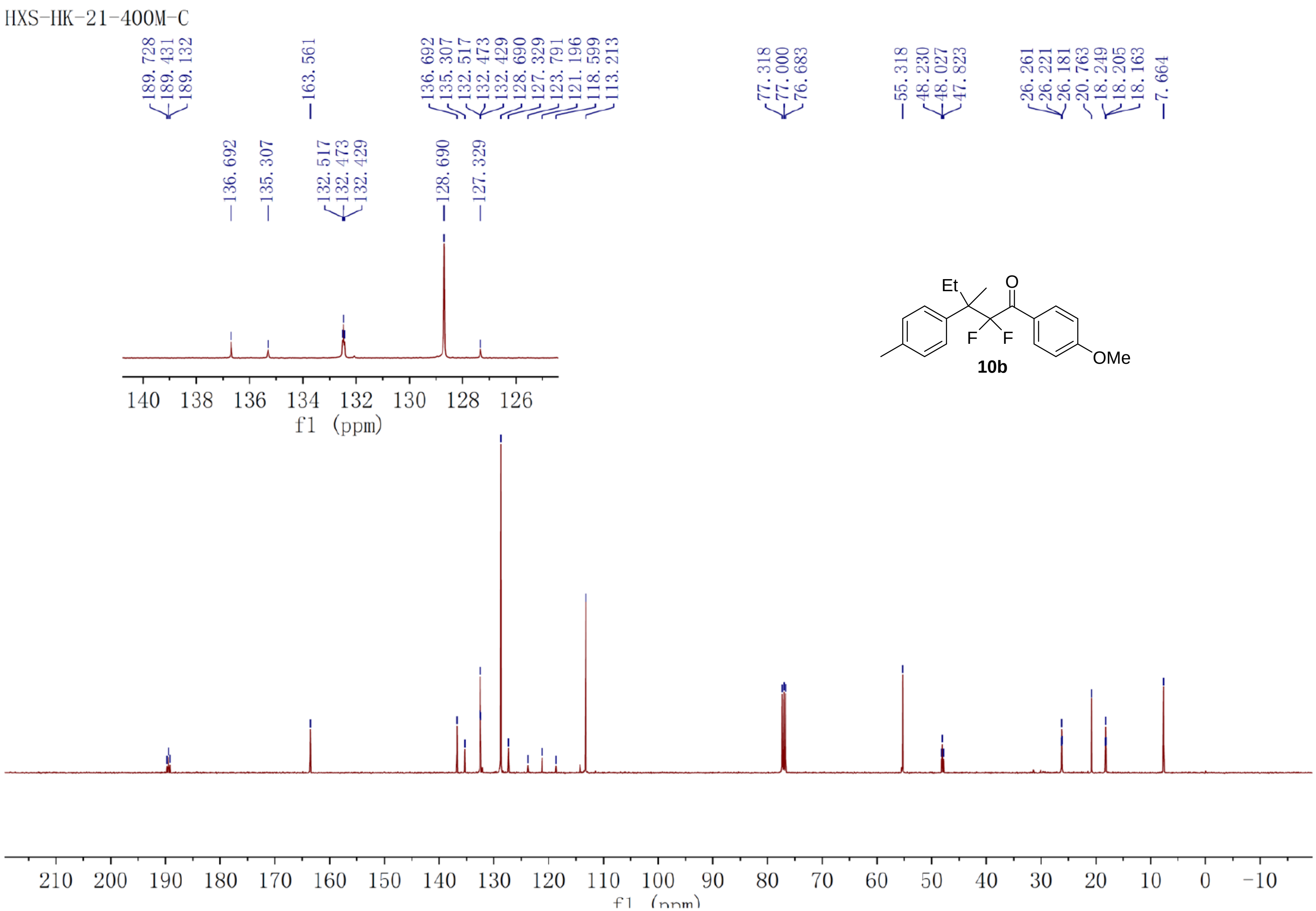

Supplementary Figure 210. ^{13}C NMR (100 MHz, $CDCl_3$) spectra for compound **10b**

HXS-HK-21-400M-F

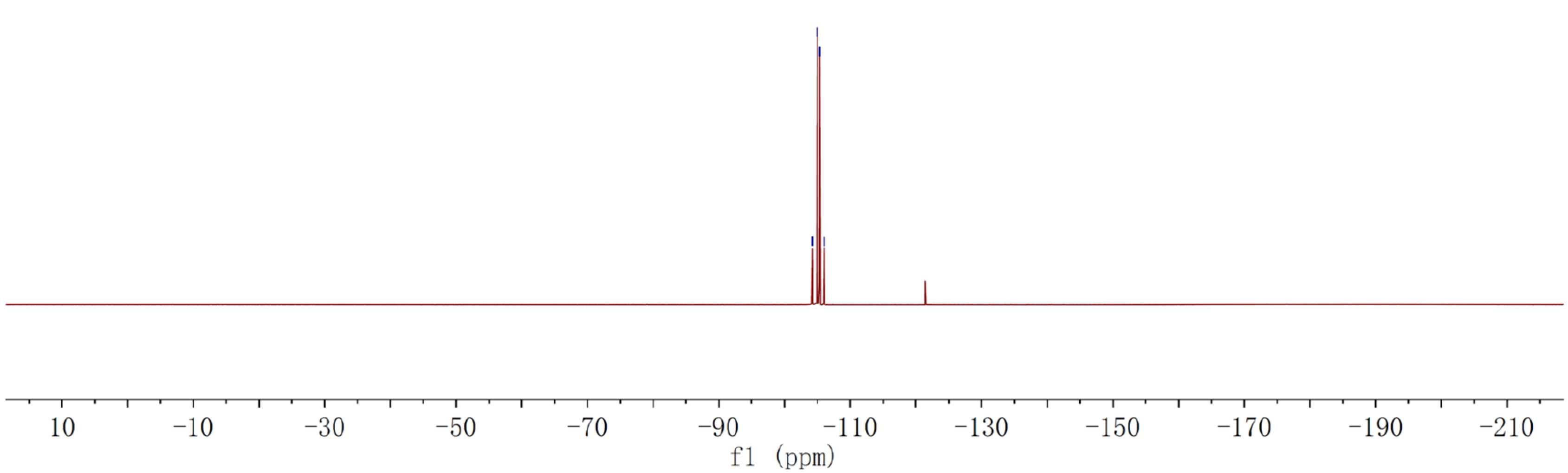

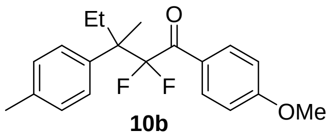

10b

Supplementary Figure 211. ^{19}F NMR (376 MHz, $CDCl_3$) spectra for compound **10b**

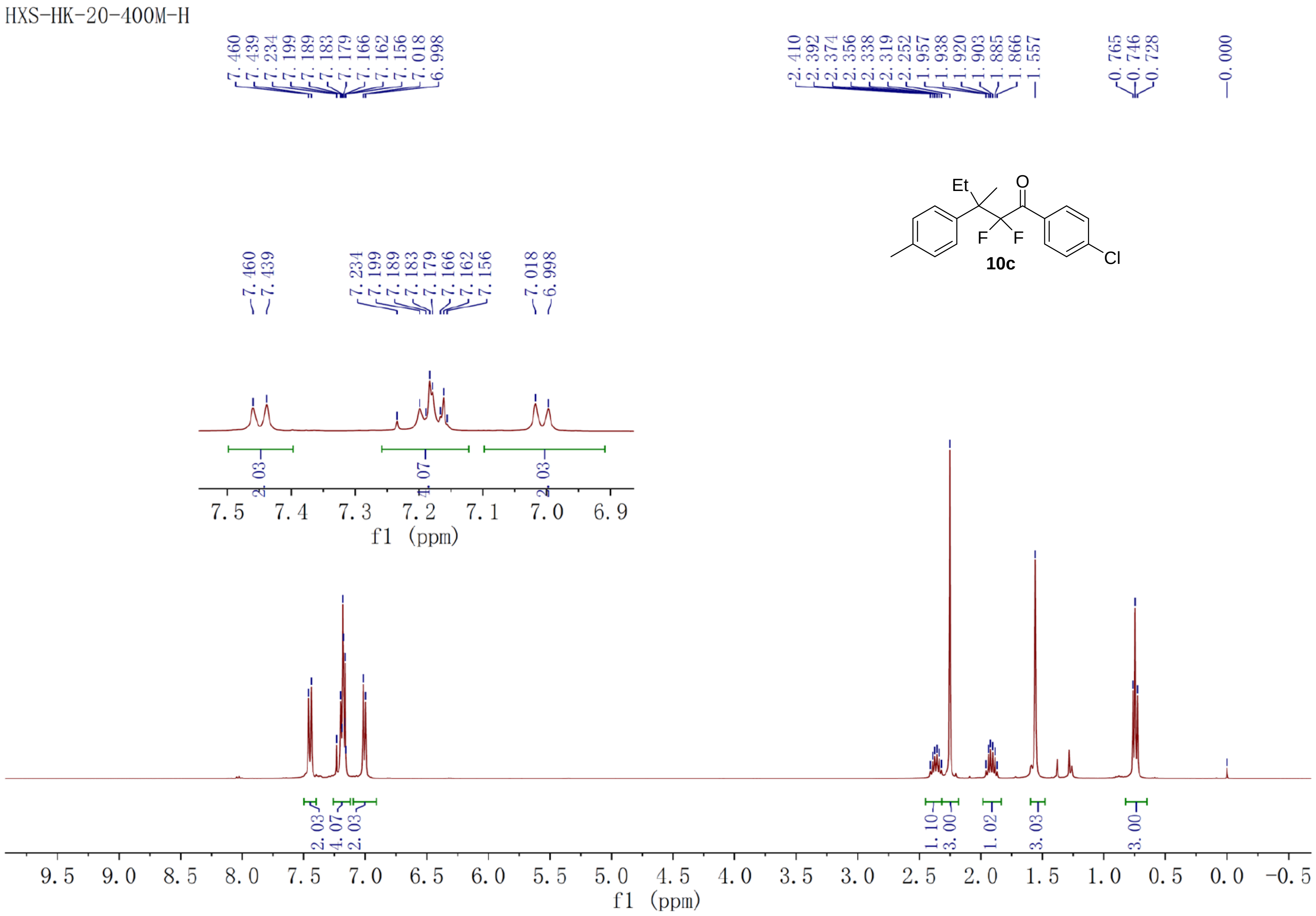

Supplementary Figure 212. ^{1}H NMR (400 MHz, $CDCl_3$) spectra for compound **10c**

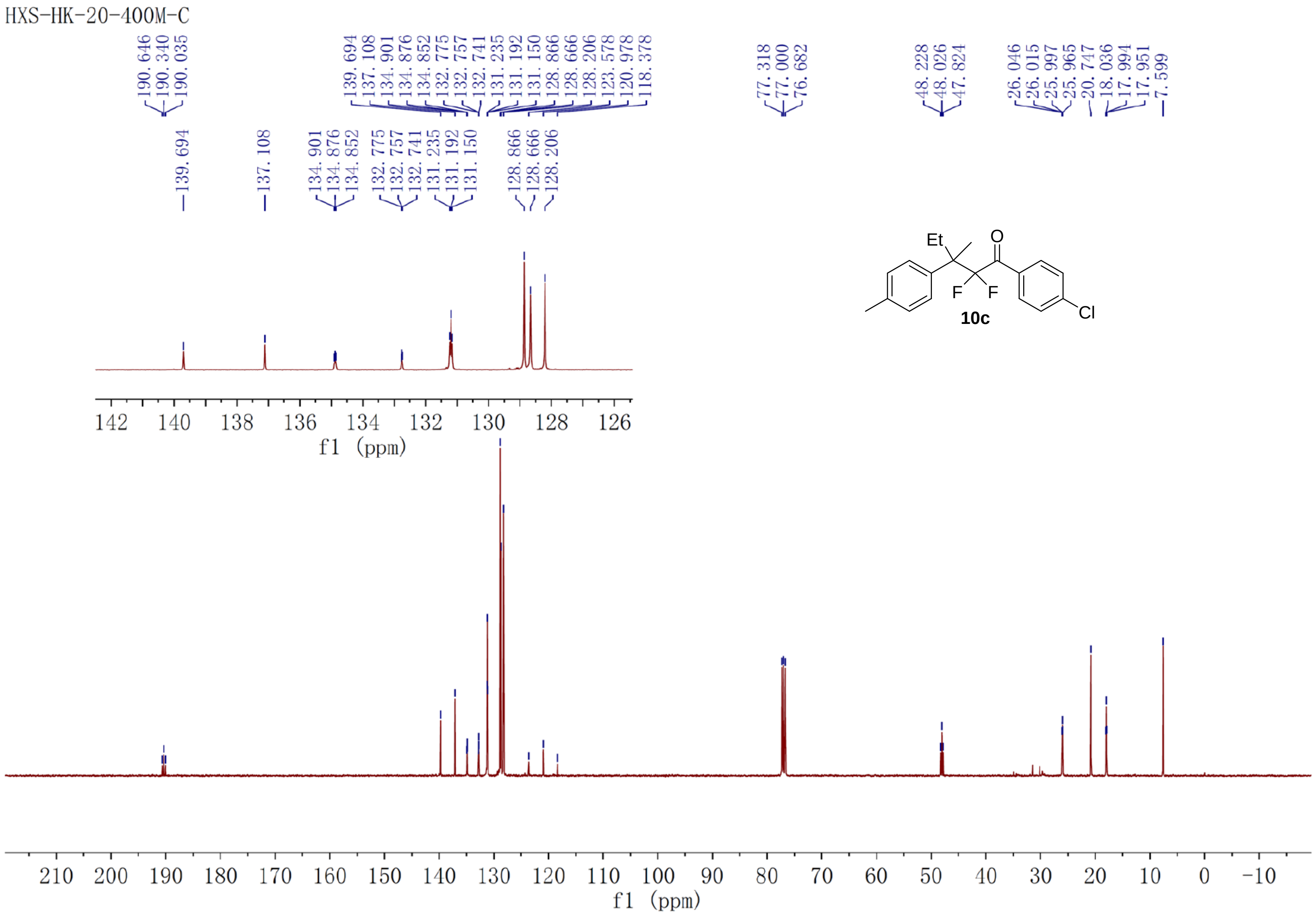

Supplementary Figure 213. ^{13}C NMR (100 MHz, $CDCl_3$) spectra for compound **10c**

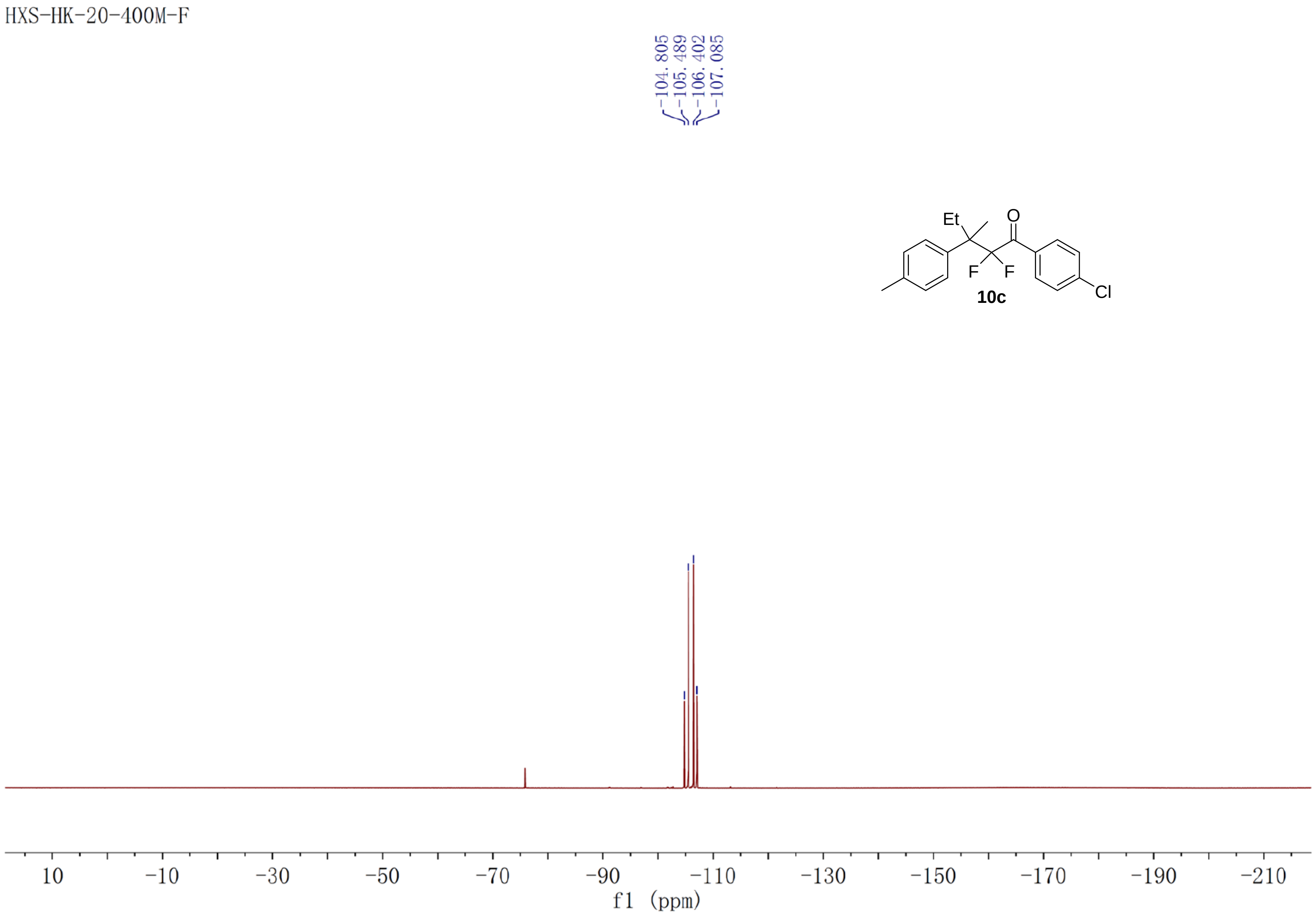

Supplementary Figure 214. ^{19}F NMR (376 MHz, $CDCl_3$) spectra for compound **10c**

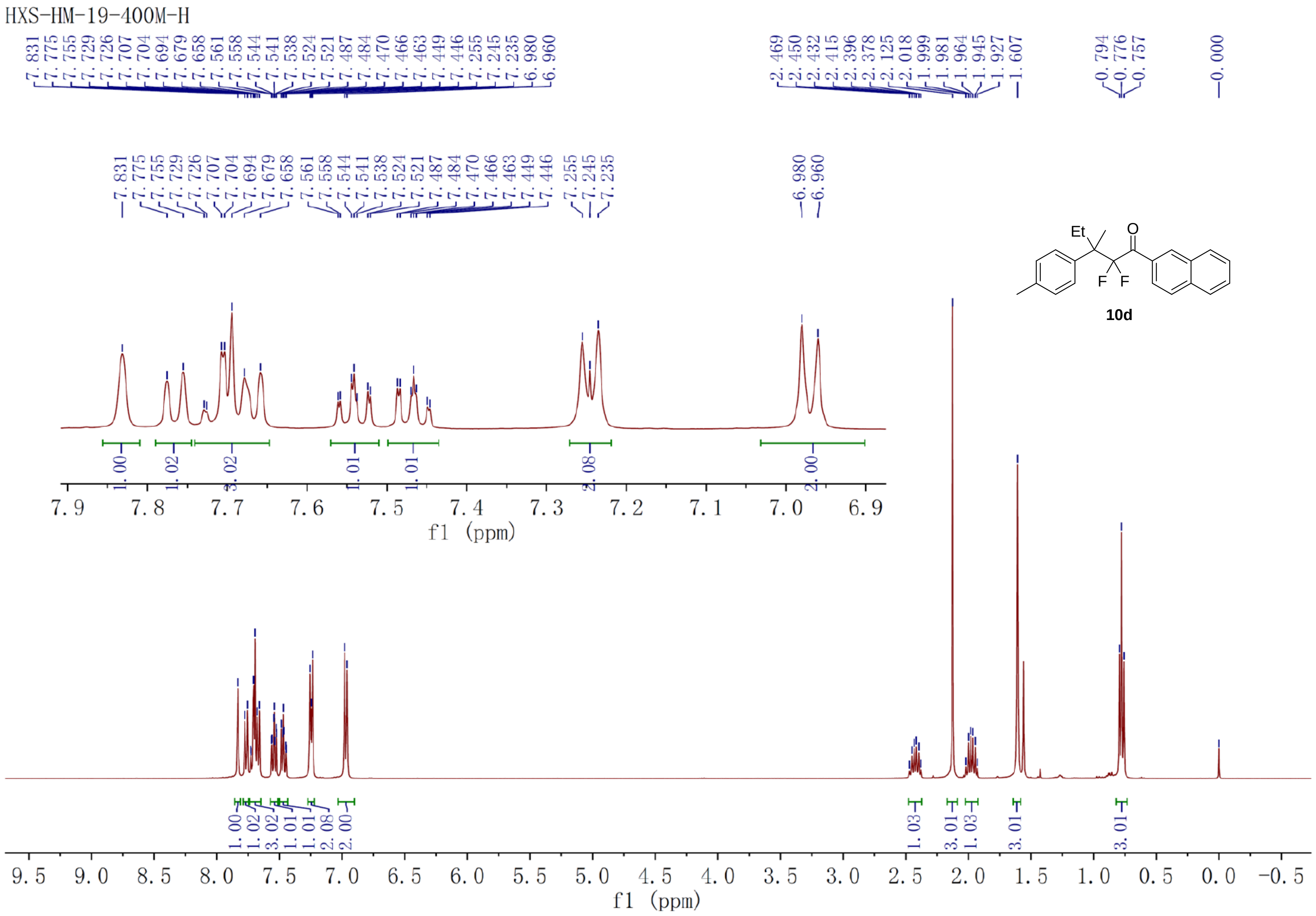

Supplementary Figure 215. ^{1}H NMR (400 MHz, $CDCl_3$) spectra for compound **10d**

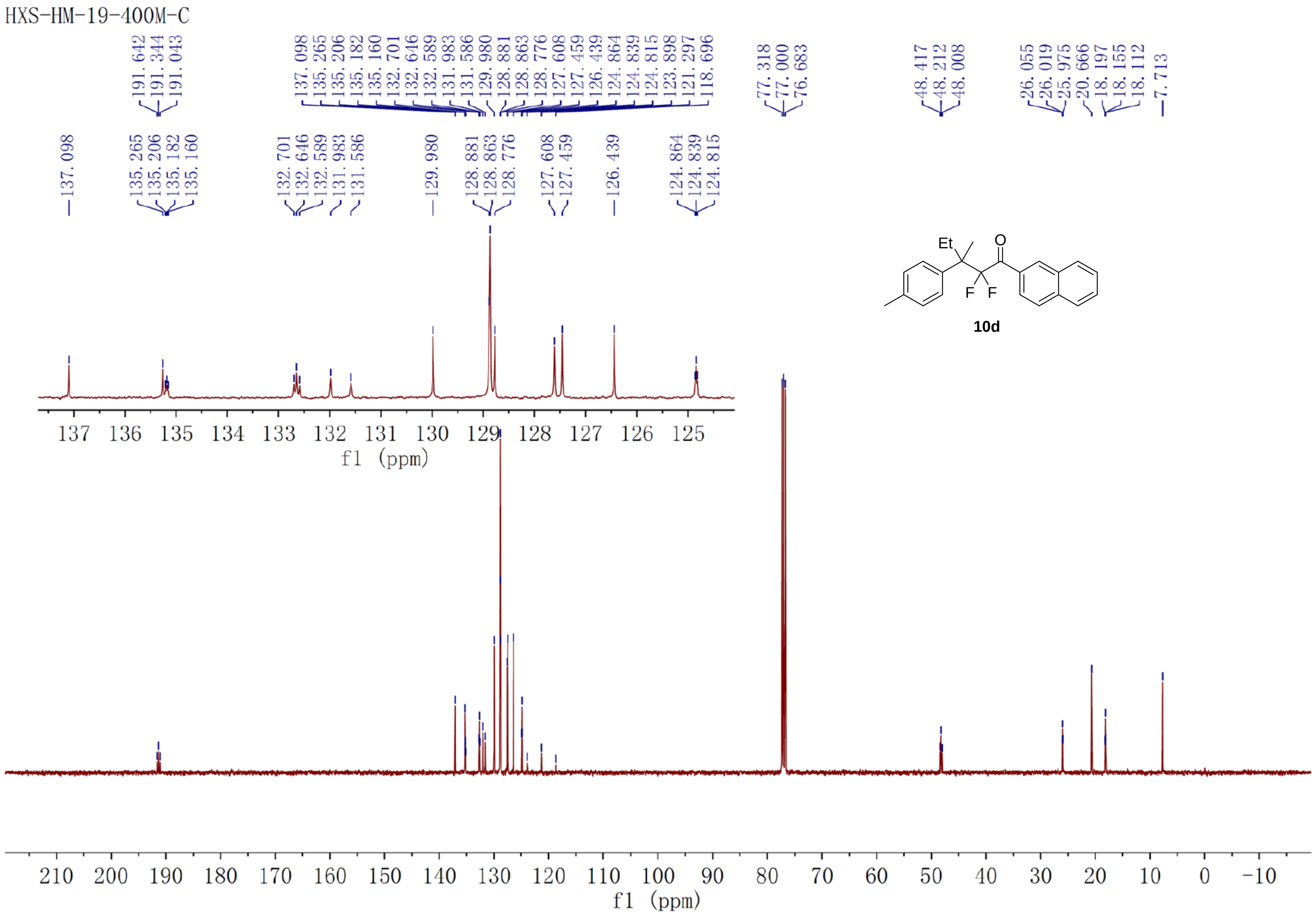

Supplementary Figure 216. ^{13}C NMR (100 MHz, $CDCl_3$) spectra for compound **10d**

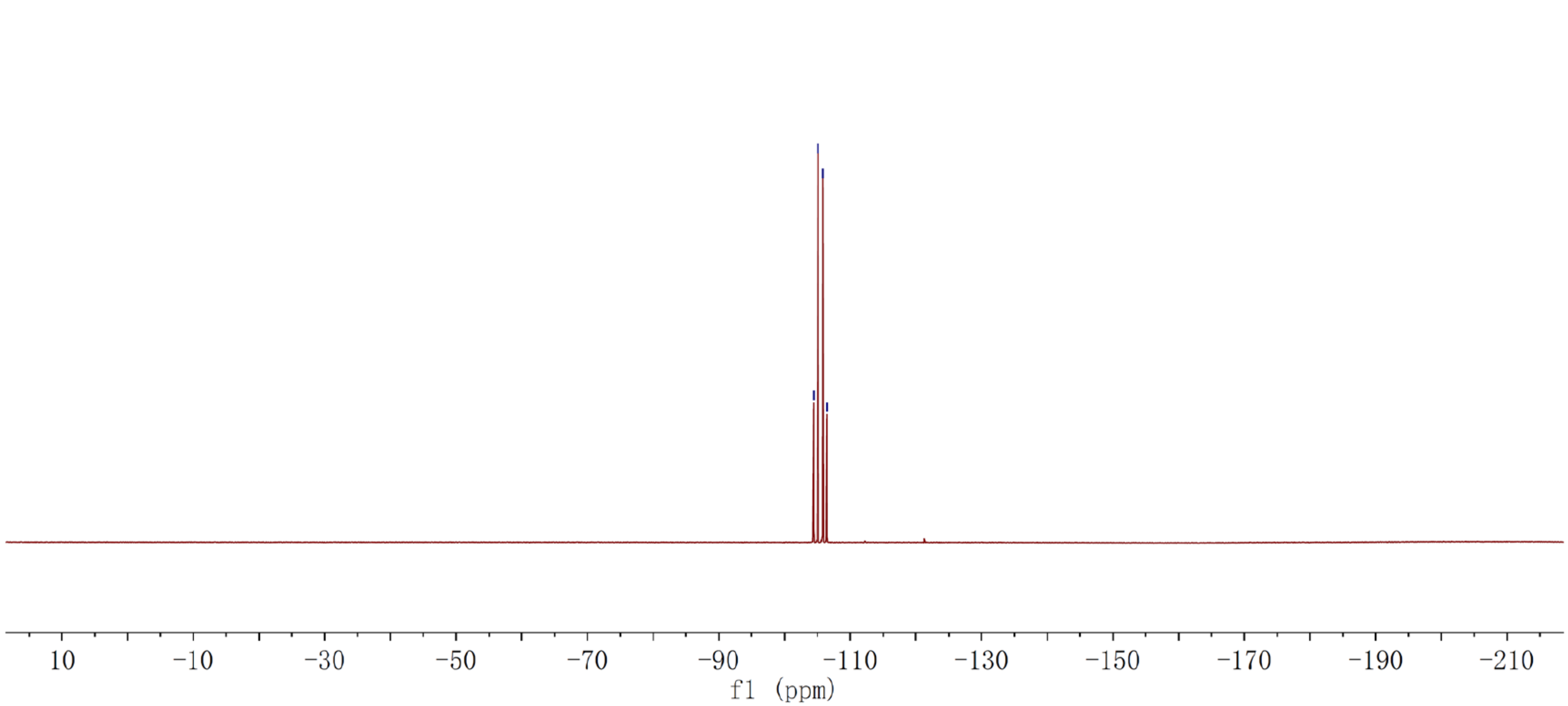

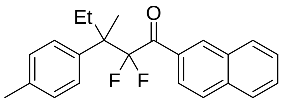

10d

Supplementary Figure 217. ^{19}F NMR (376 MHz, $CDCl_3$) spectra for compound **10d**

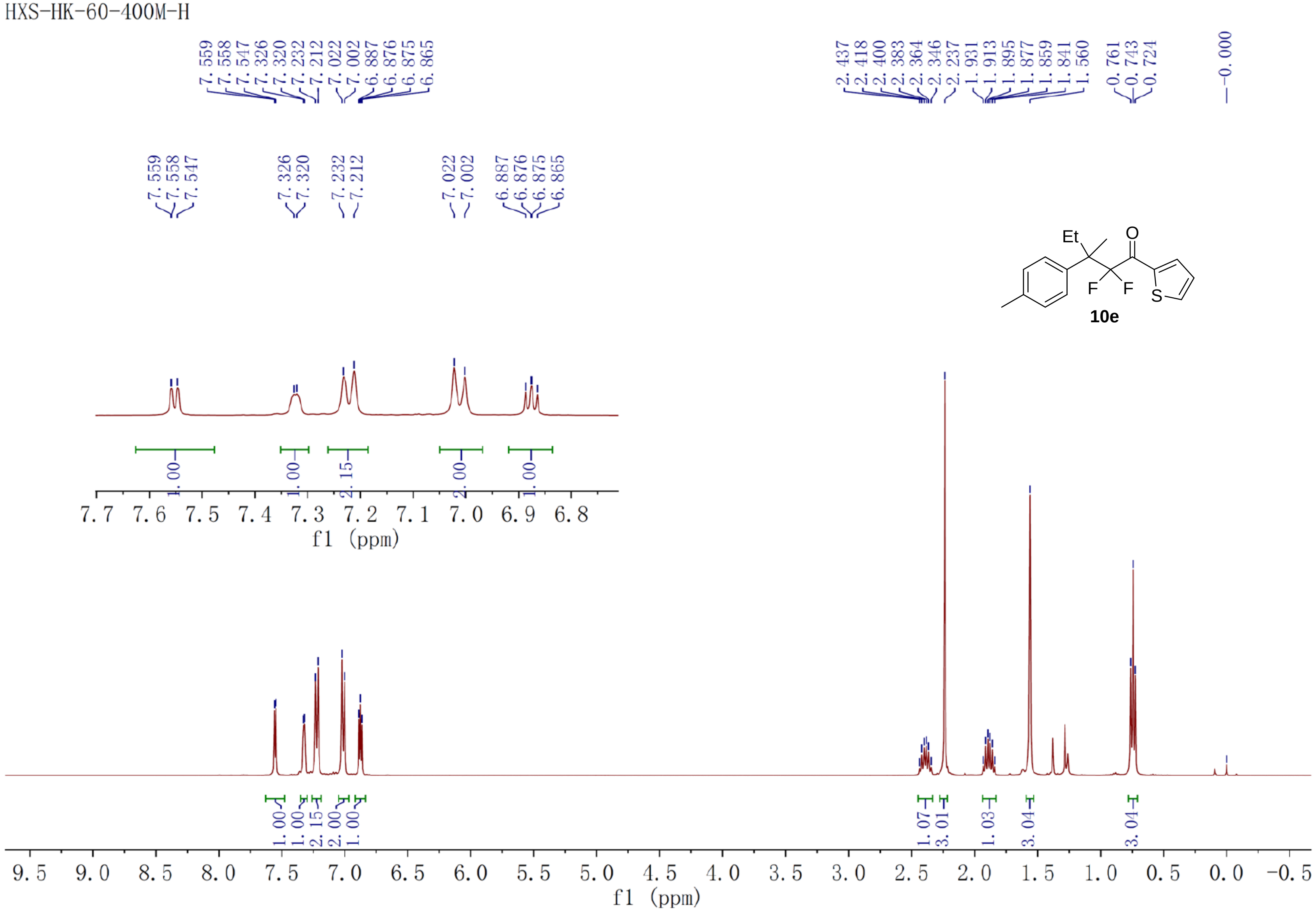

Supplementary Figure 218. ^{1}H NMR (400 MHz, $CDCl_3$) spectra for compound **10e**

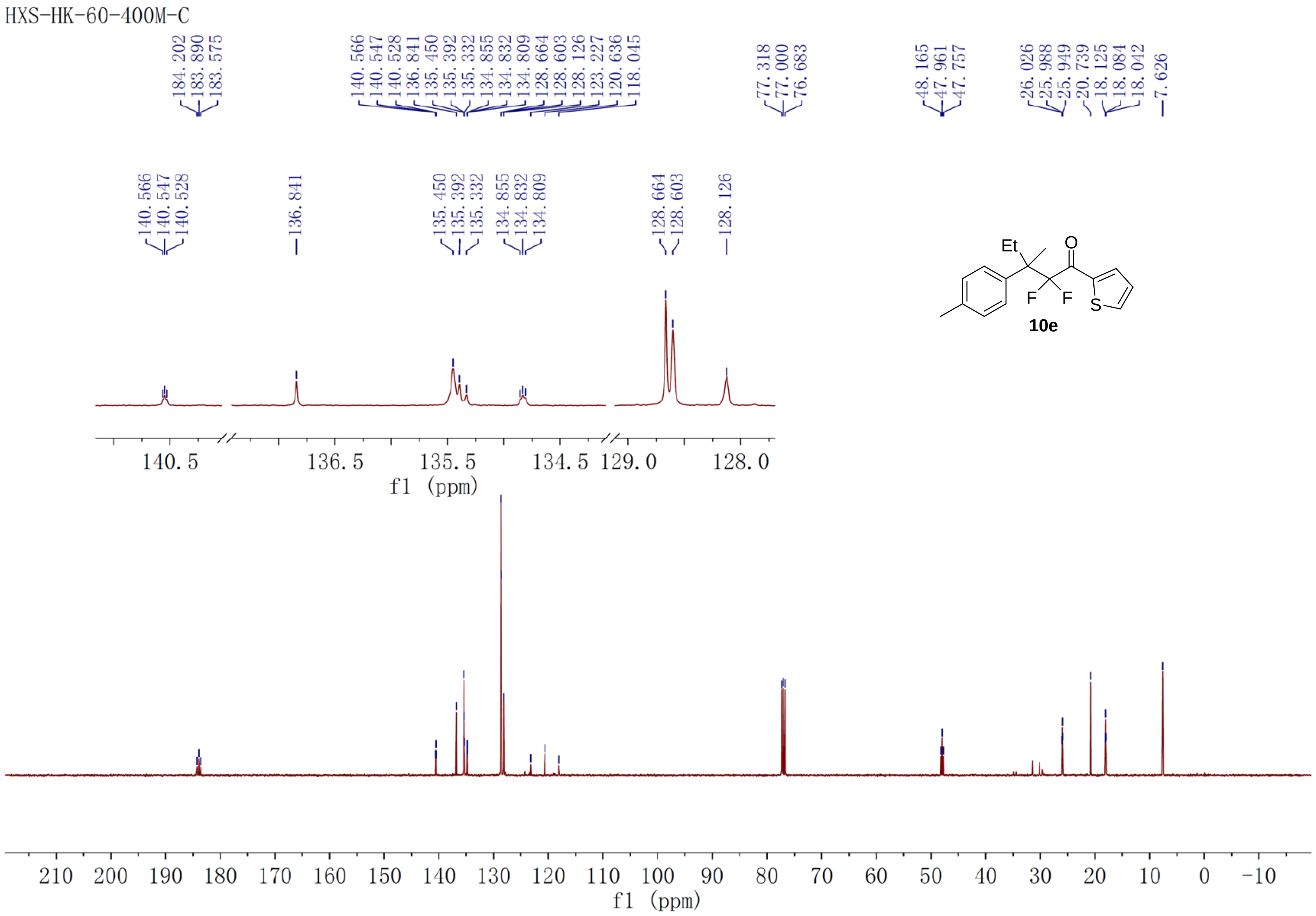

10e

Supplementary Figure 219. ^{13}C NMR (100 MHz, $CDCl_3$) spectra for compound **10e**

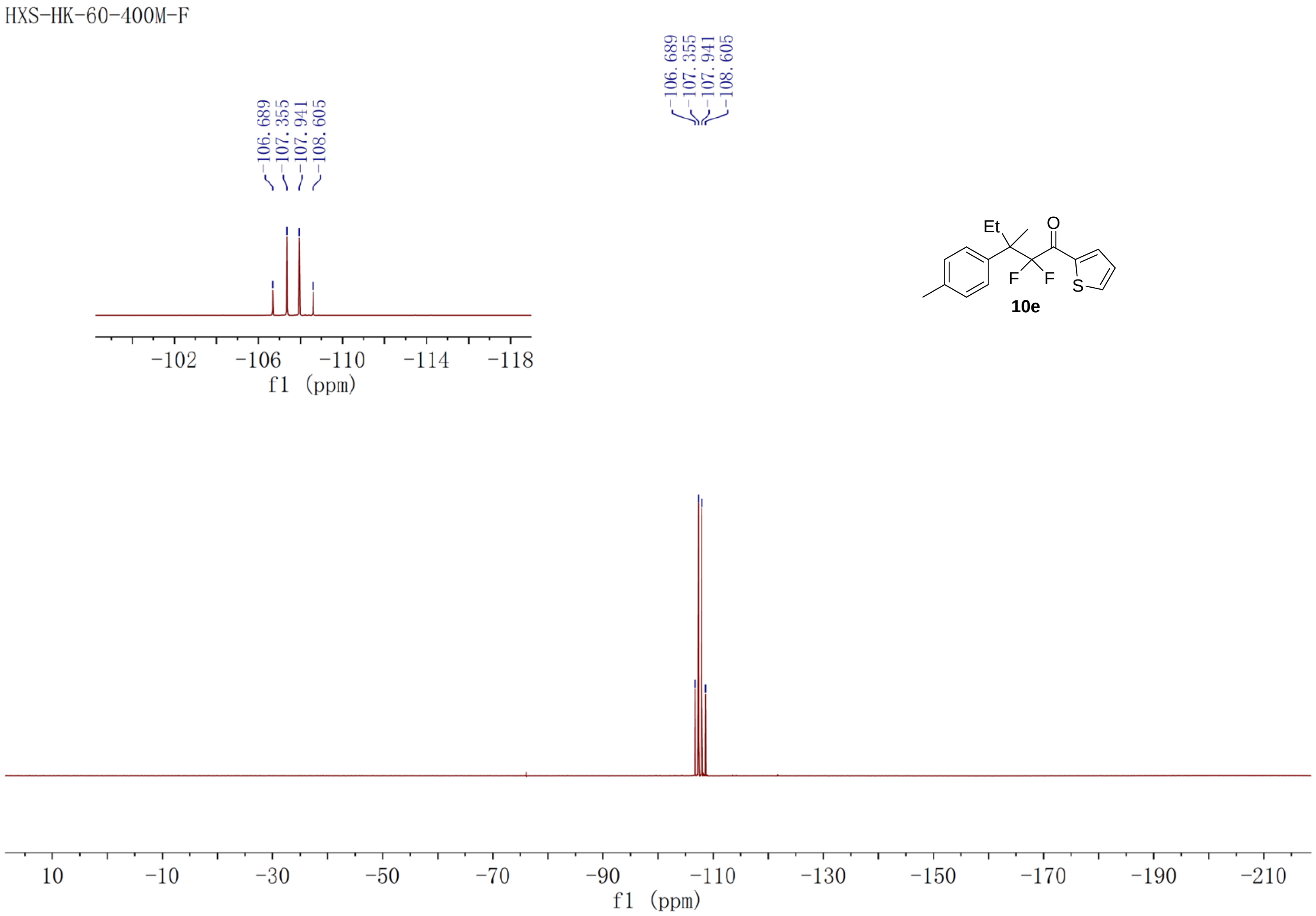

Supplementary Figure 220. ^{19}F NMR (376 MHz, $CDCl_3$) spectra for compound **10e**

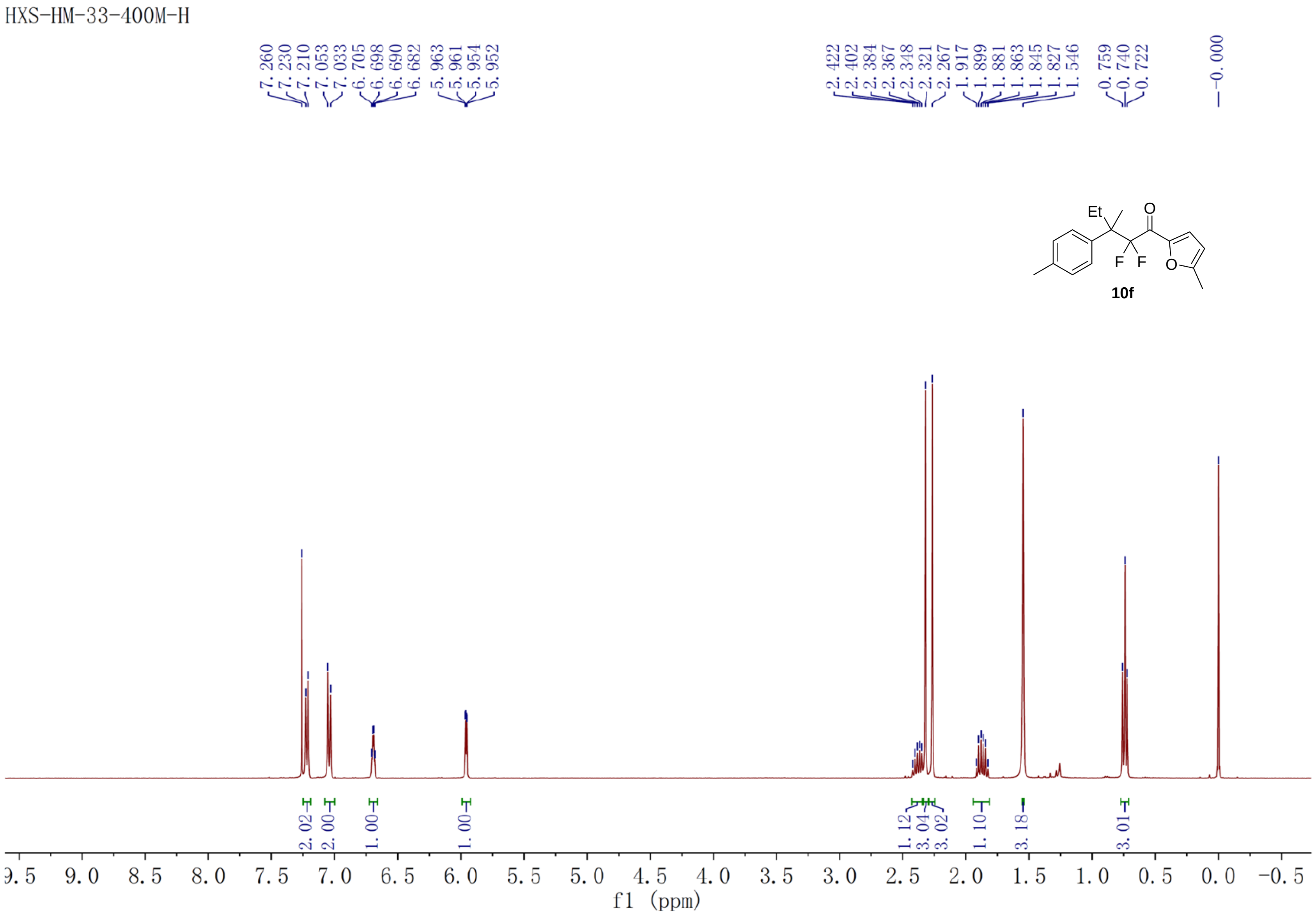

Supplementary Figure 221. ^{1}H NMR (400 MHz, $CDCl_3$) spectra for compound **10f**

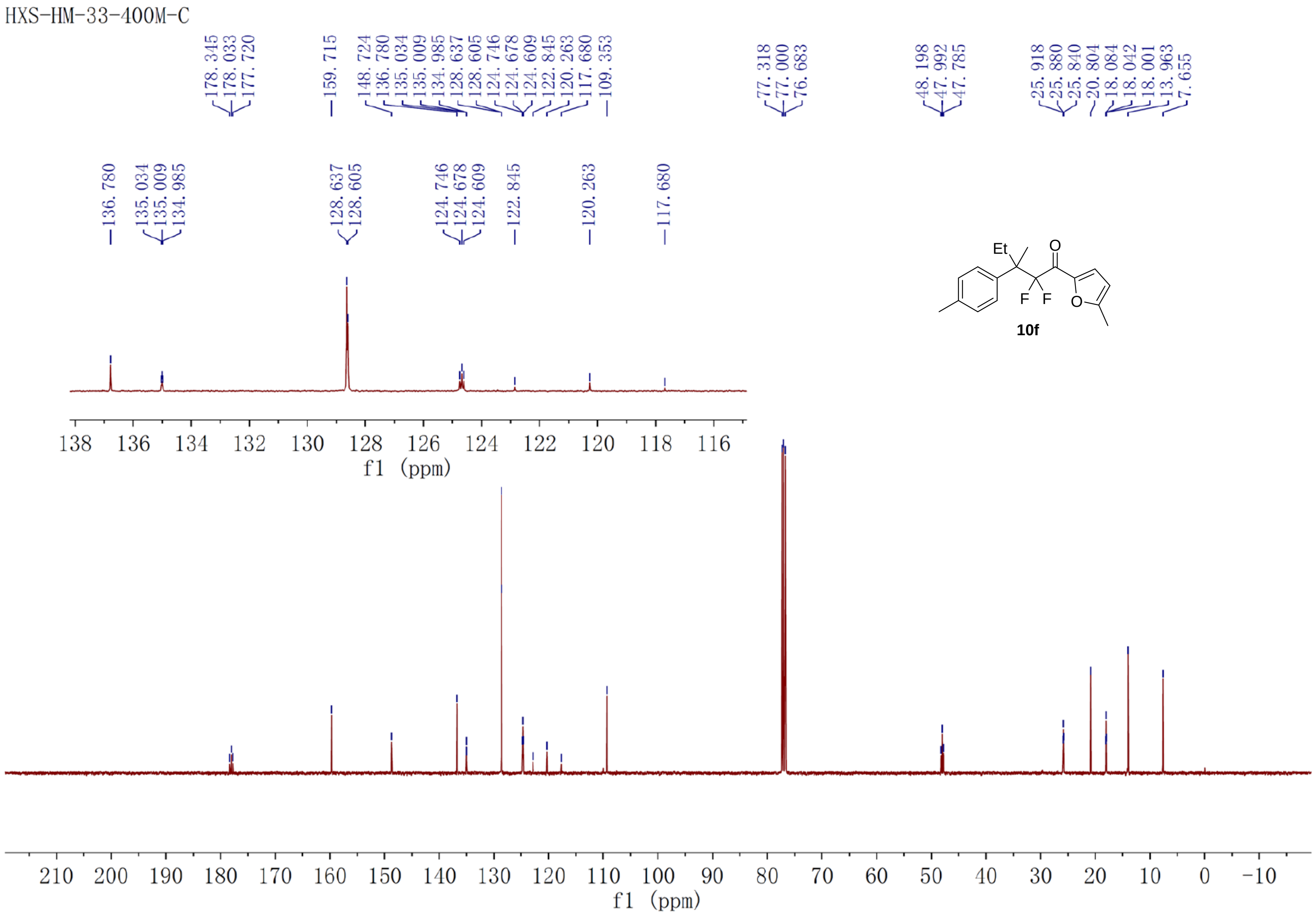

Supplementary Figure 222. ^{13}C NMR (100 MHz, $CDCl_3$) spectra for compound **10f**

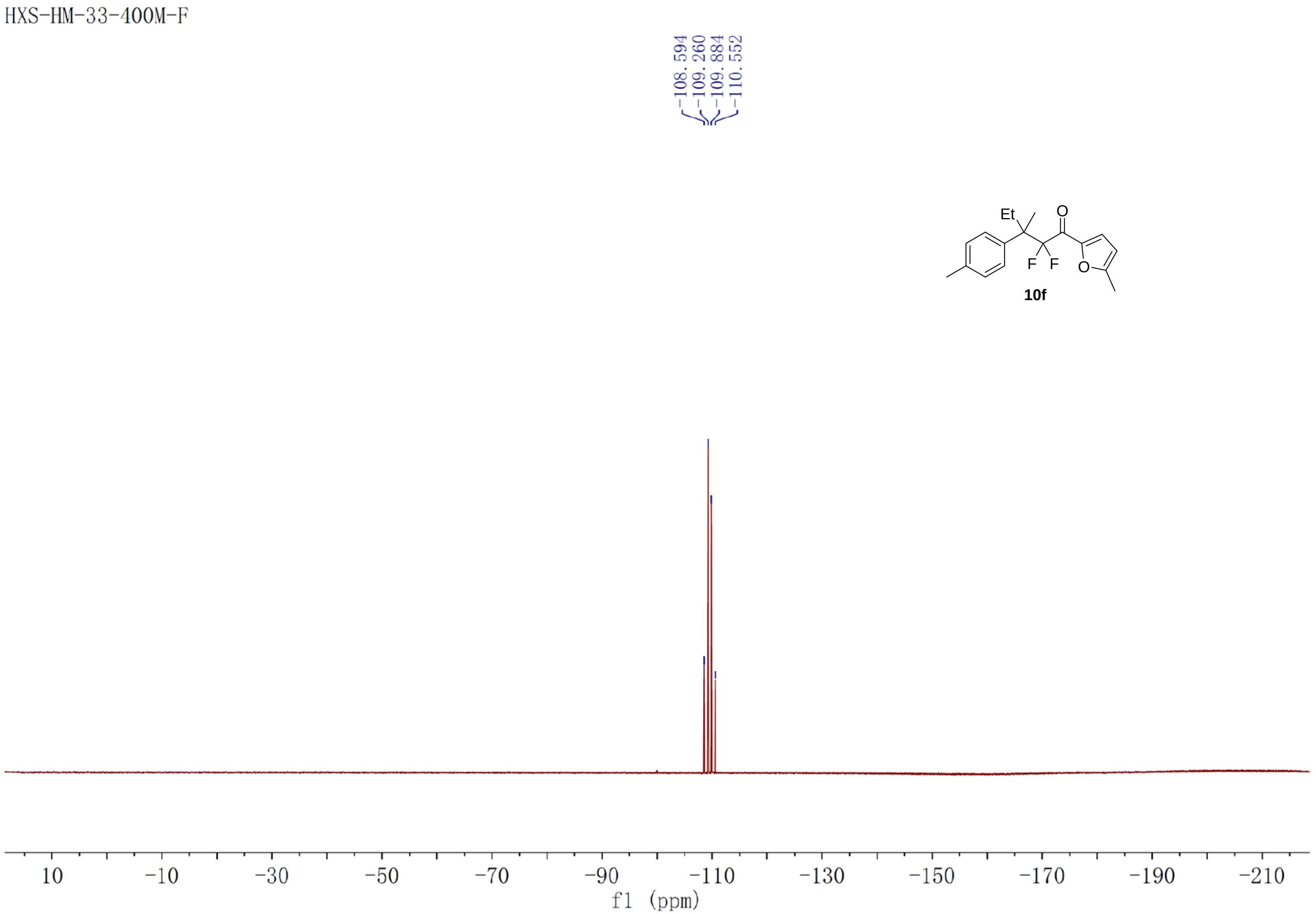

Supplementary Figure 223. ^{19}F NMR (376 MHz, $CDCl_3$) spectra for compound **10f**

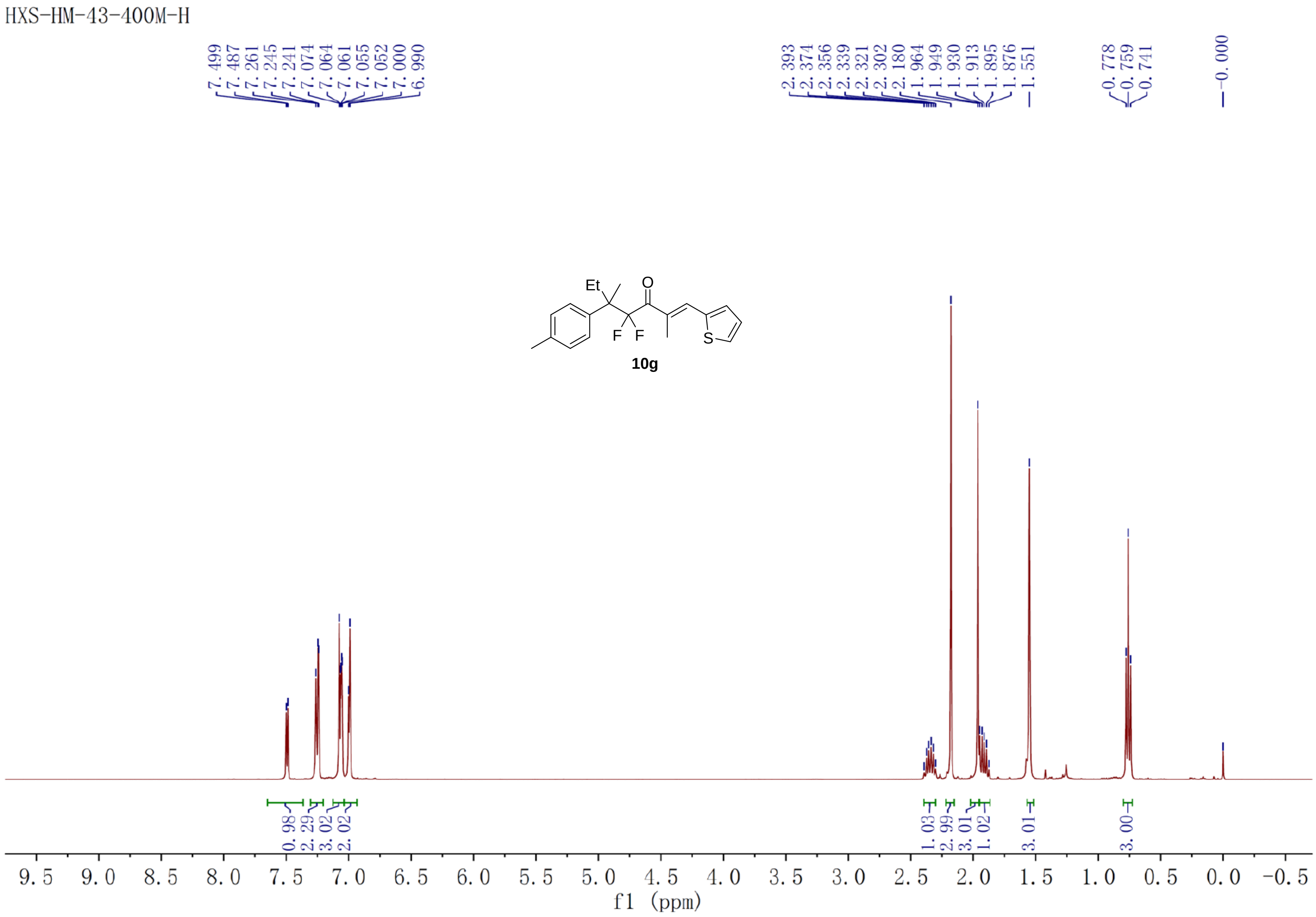

10g

Supplementary Figure 224. ^{1}H NMR (400 MHz, $CDCl_3$) spectra for compound **10g**

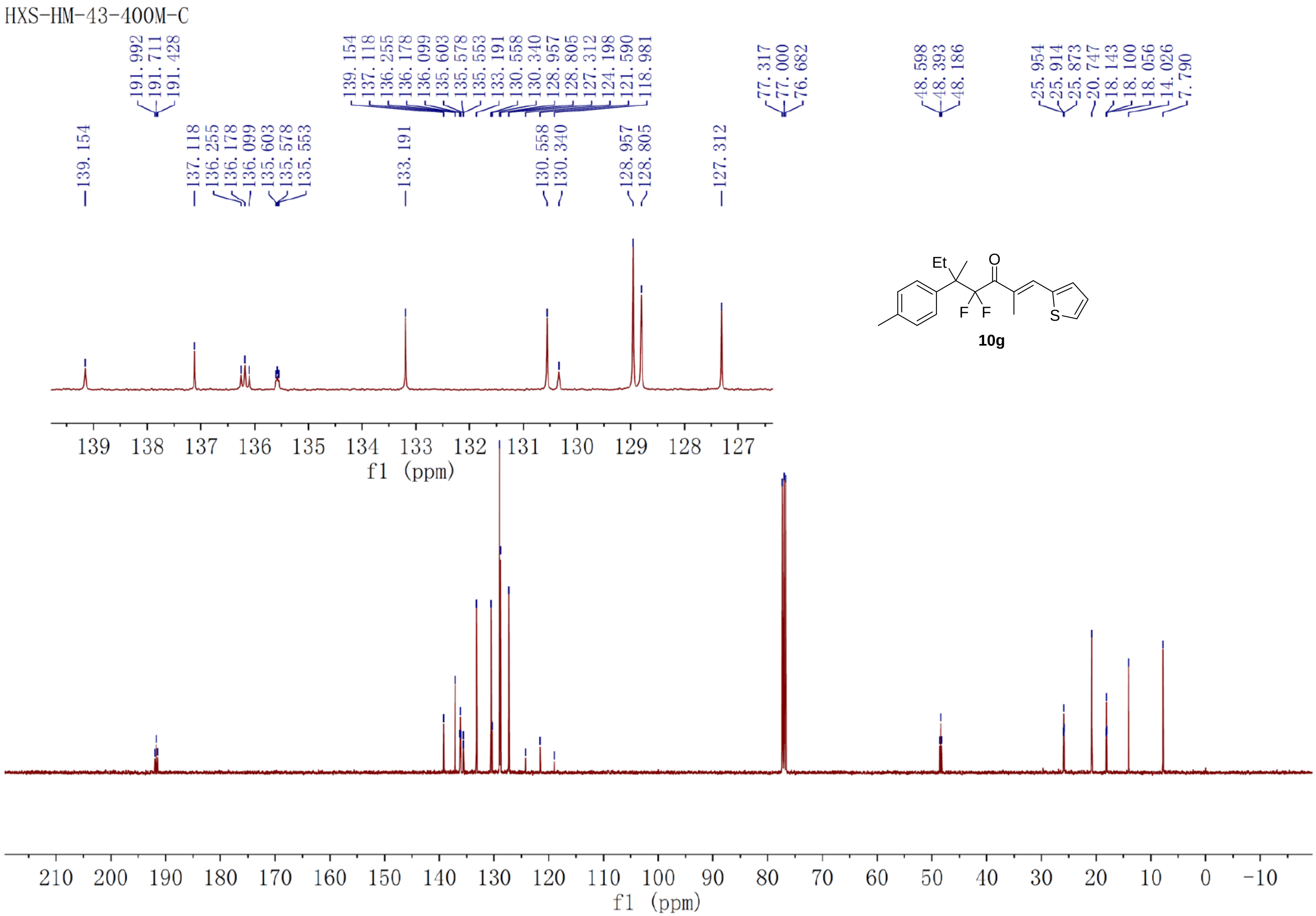

Supplementary Figure 225. ^{13}C NMR (100 MHz, $CDCl_3$) spectra for compound **10g**

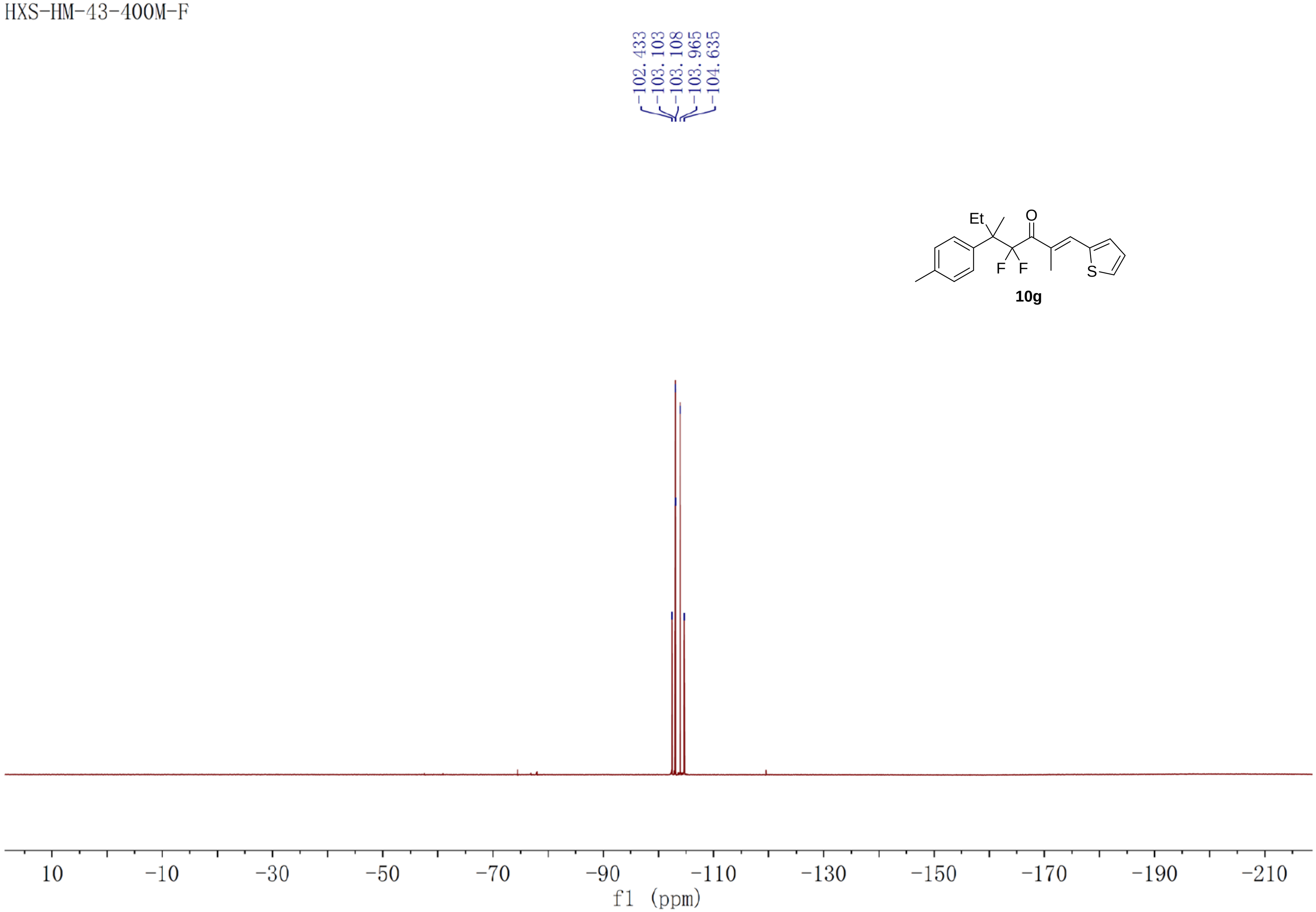

Supplementary Figure 226. ^{19}F NMR (376 MHz, $CDCl_3$) spectra for compound **10g**

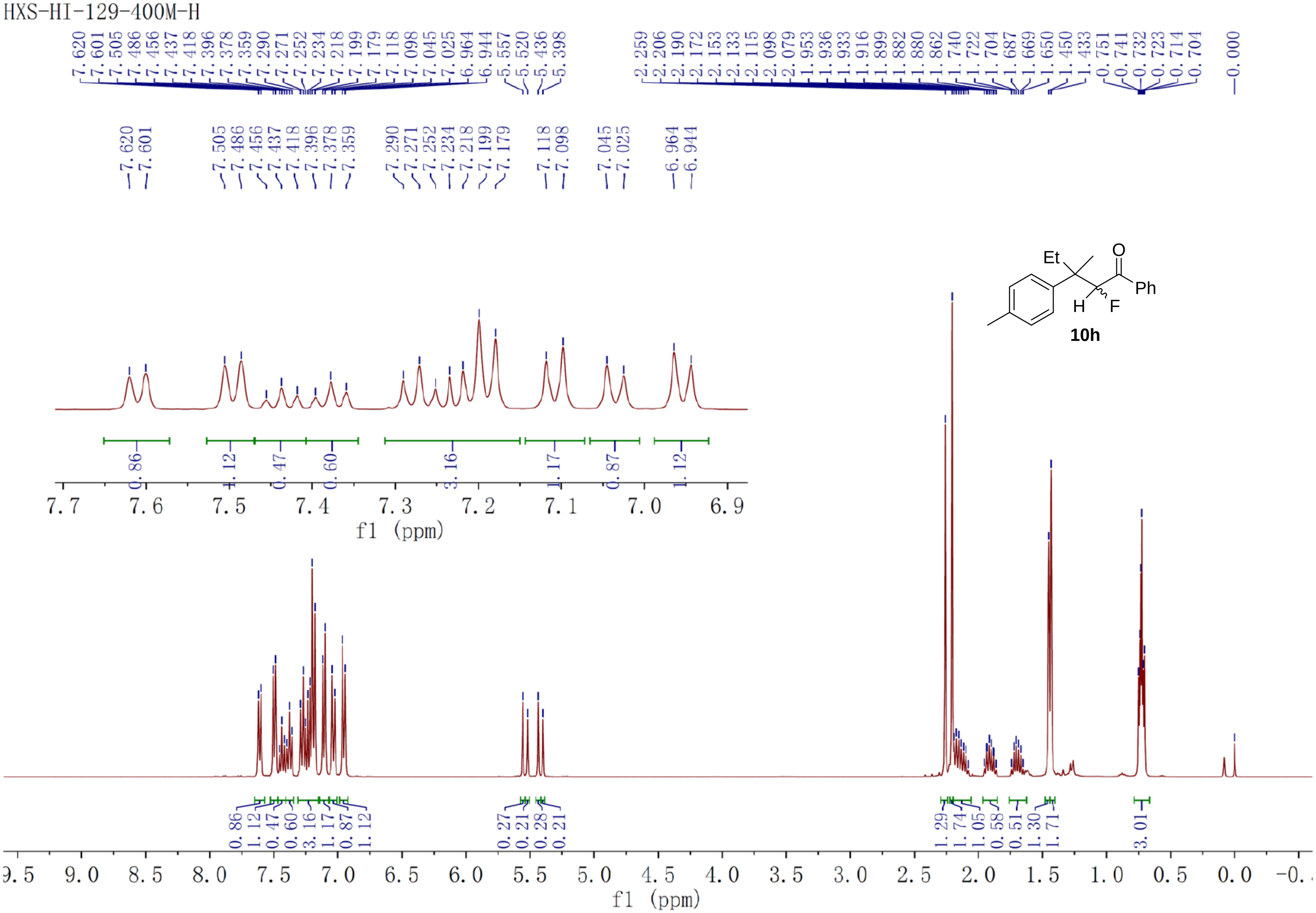

Supplementary Figure 227. ^{1}H NMR (400 MHz, $CDCl_3$) spectra for compound **10h**

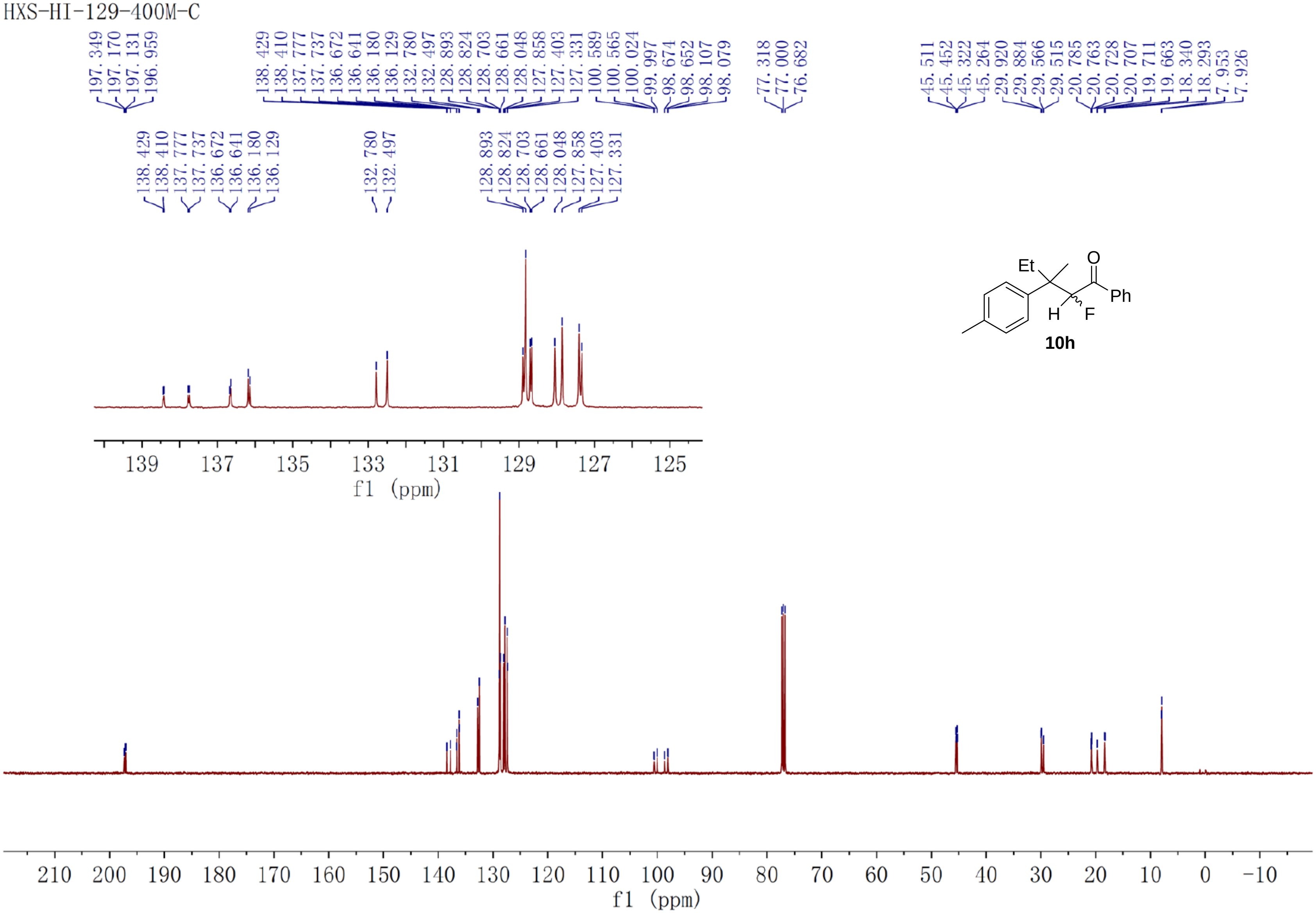

Supplementary Figure 228. ^{13}C NMR (100 MHz, $CDCl_3$) spectra for compound **10h**

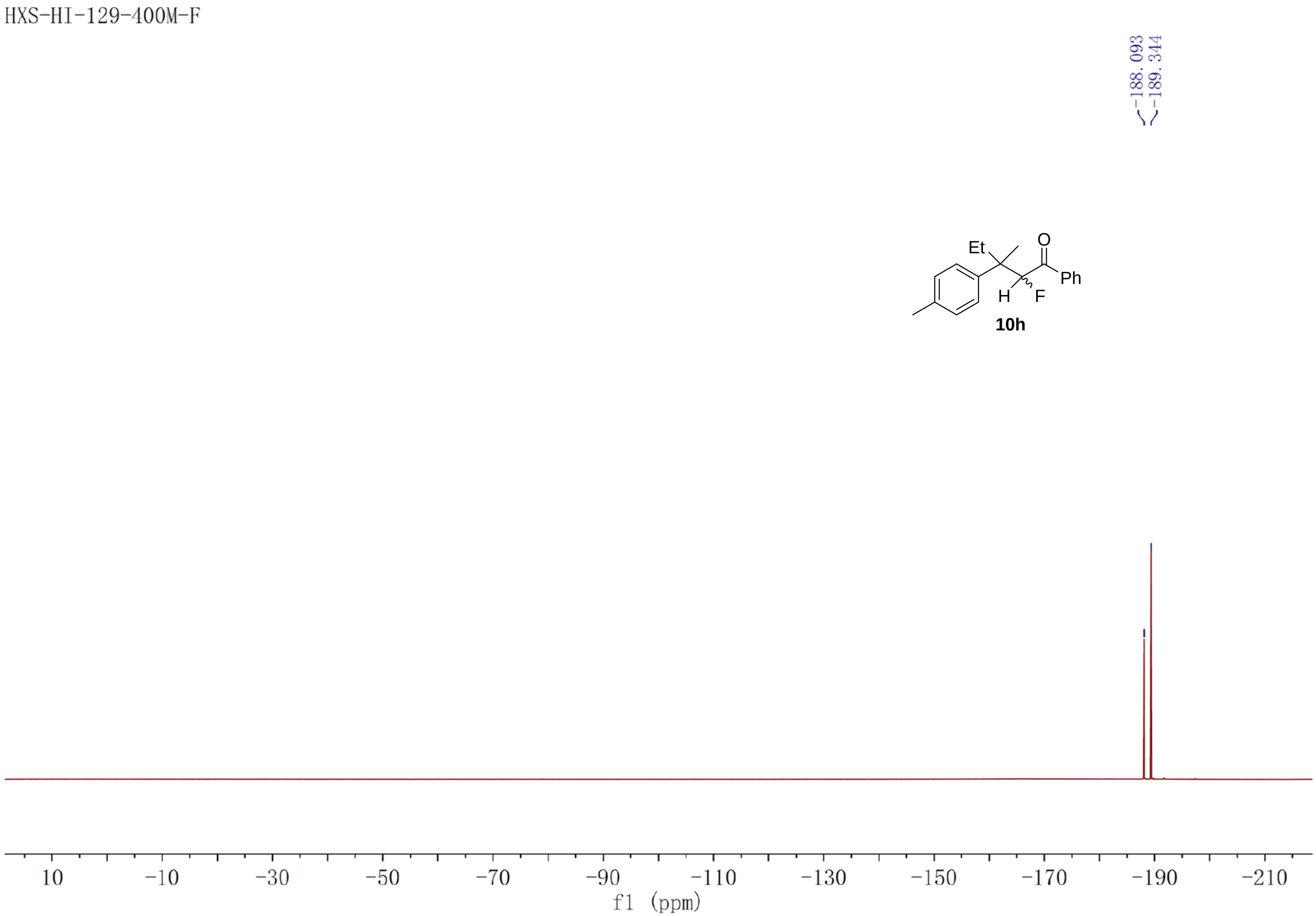

Supplementary Figure 229. ^{19}F NMR (376 MHz, $CDCl_3$) spectra for compound **10h**

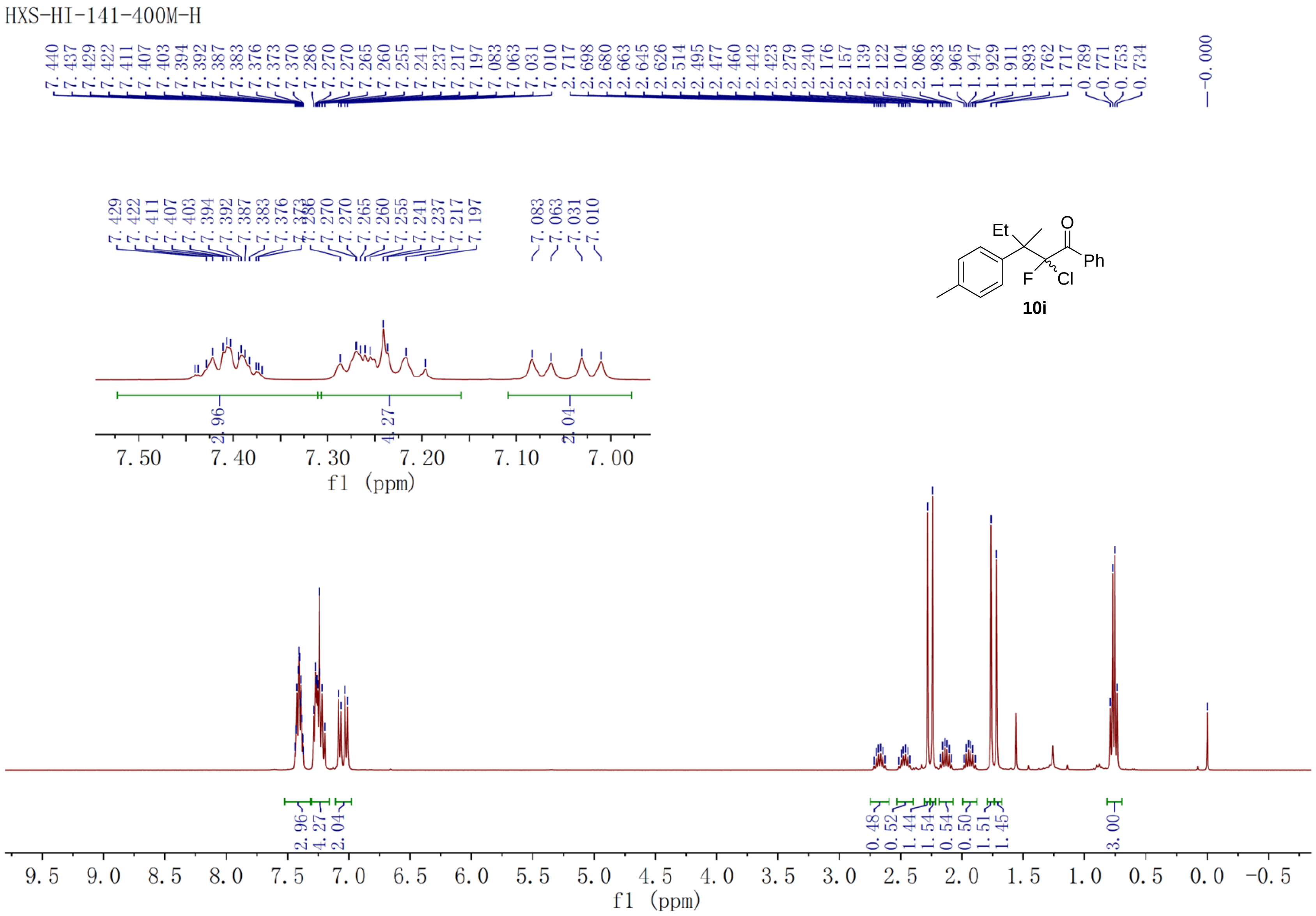

10i

Supplementary Figure 230. ^{1}H NMR (400 MHz, $CDCl_3$) spectra for compound **10i**

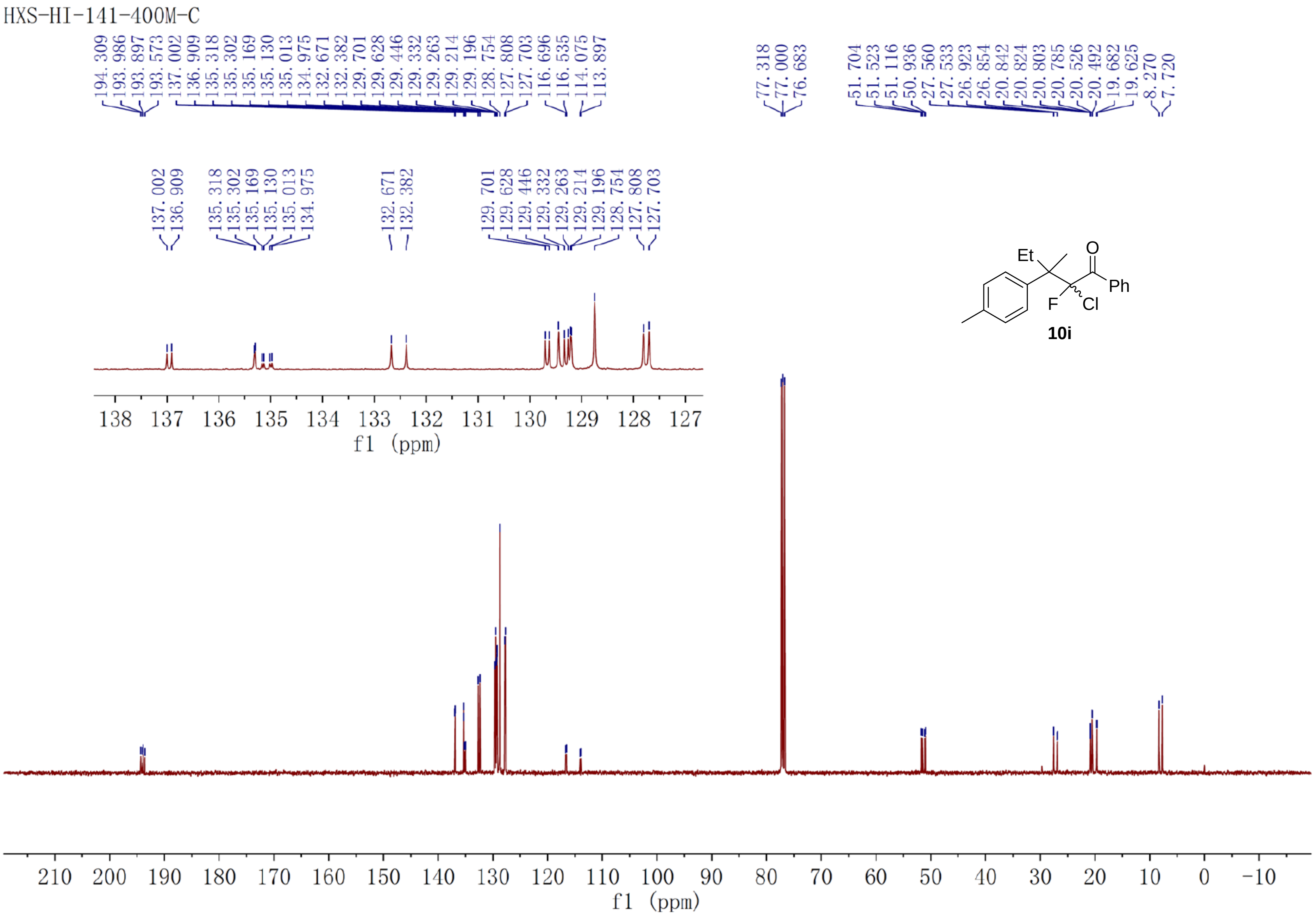

Supplementary Figure 231. ^{13}C NMR (100 MHz, $CDCl_3$) spectra for compound **10i**

HXS-HI-141-400M-F

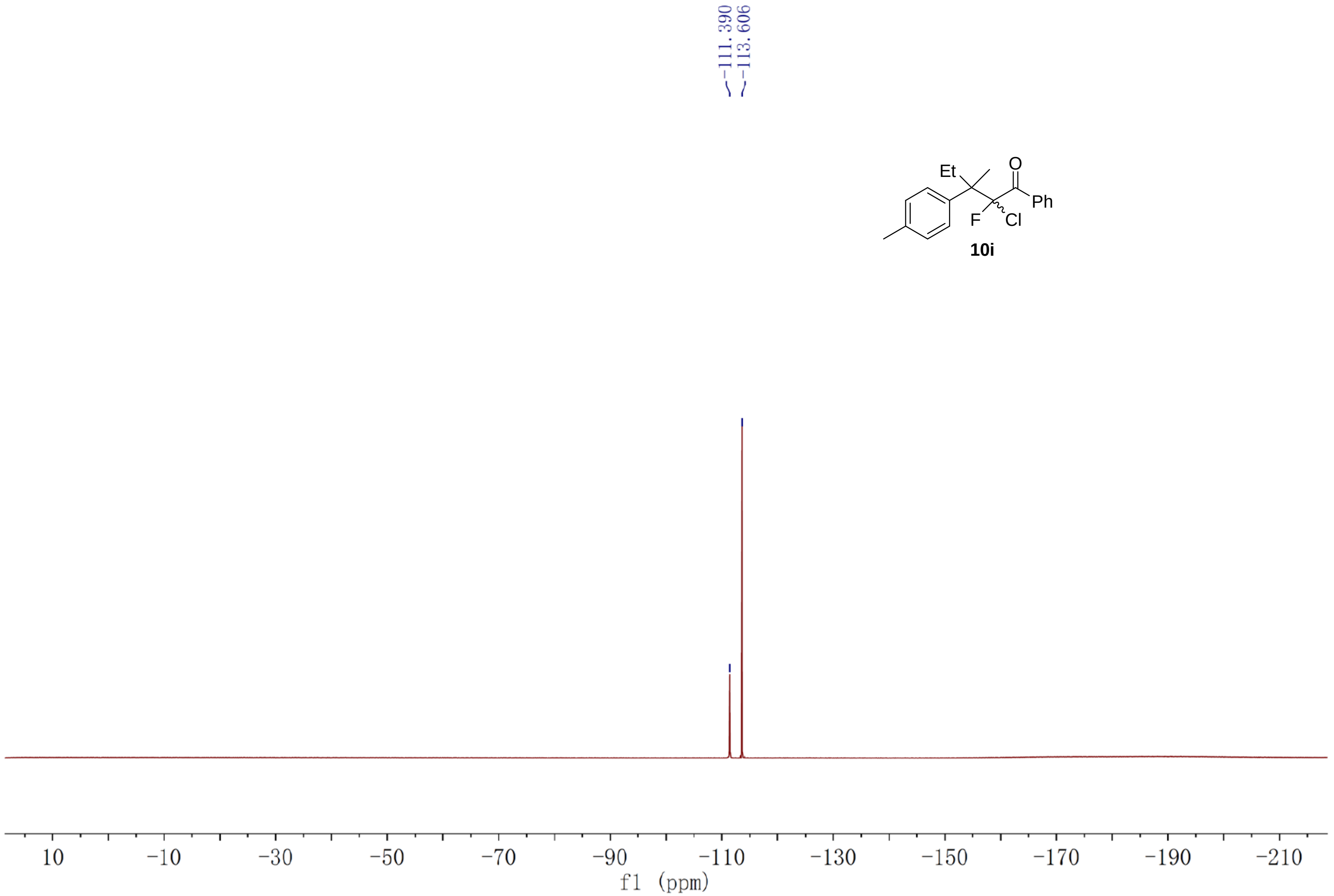

10i

Supplementary Figure 232. ^{19}F NMR (376 MHz, $CDCl_3$) spectra for compound **10i**

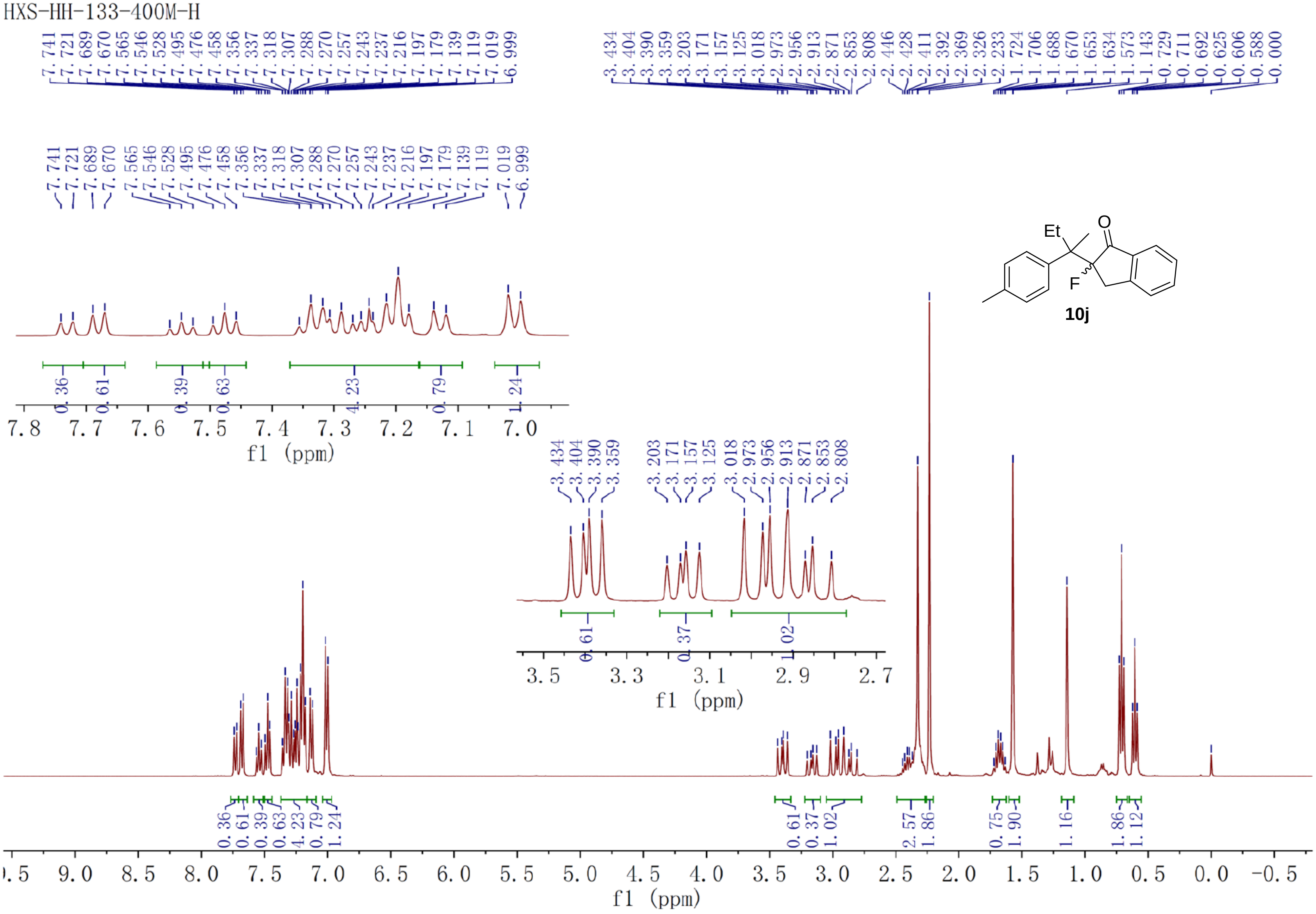

Supplementary Figure 233. ^{1}H NMR (400 MHz, $CDCl_3$) spectra for compound **10j**

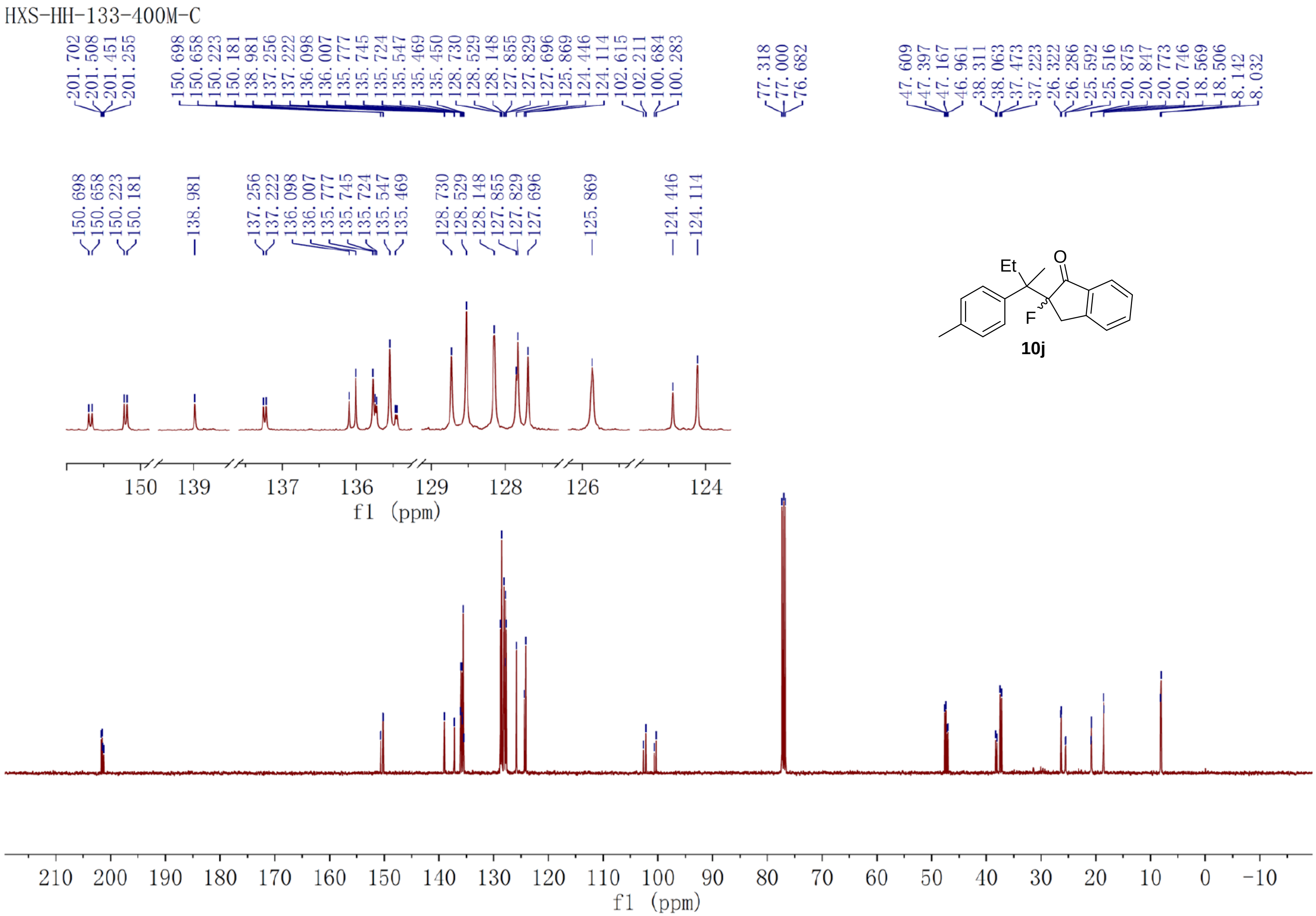

Supplementary Figure 234. ^{13}C NMR (100 MHz, $CDCl_3$) spectra for compound **10j**

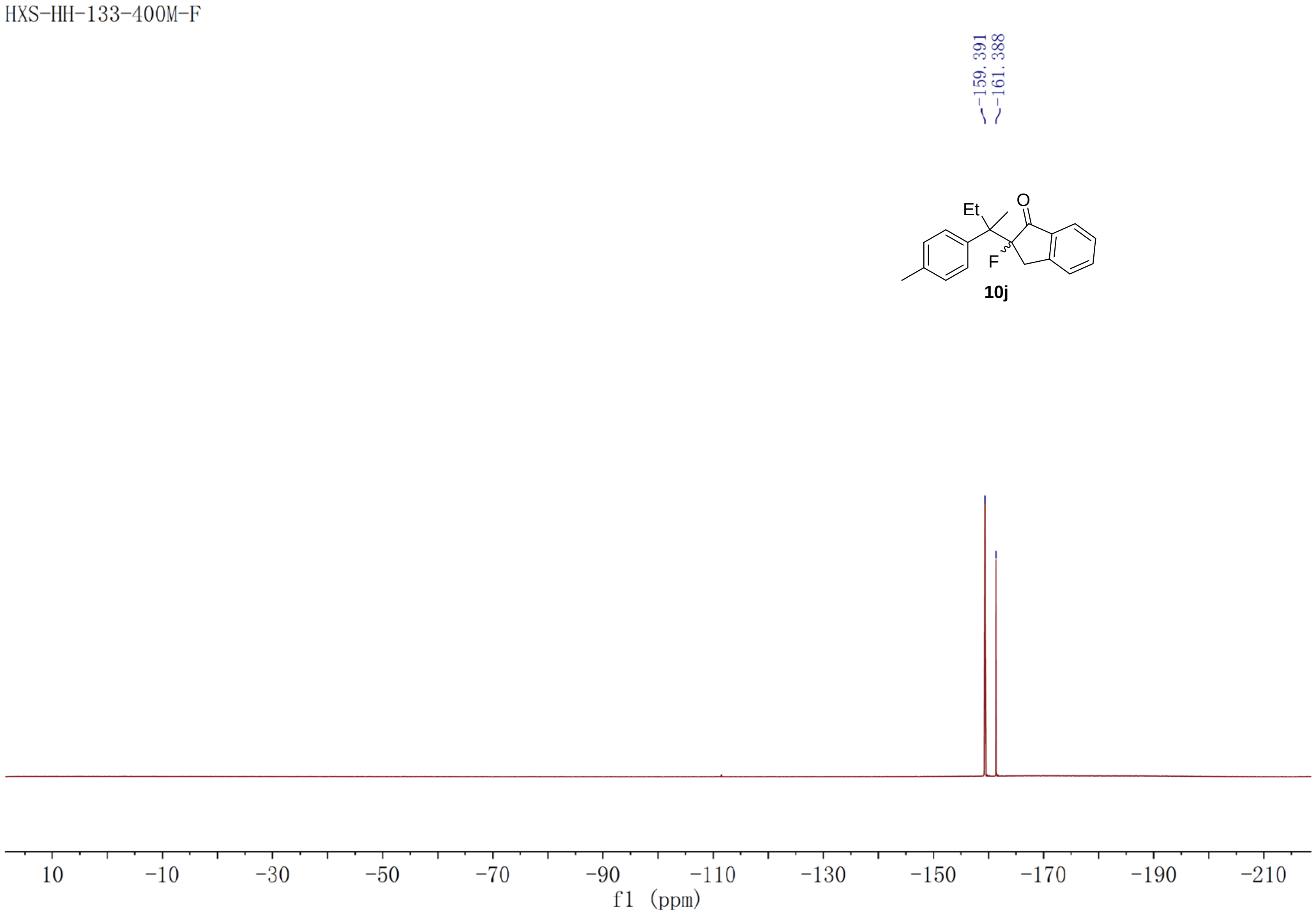

Supplementary Figure 235. ^{19}F NMR (376 MHz, $CDCl_3$) spectra for compound **10j**

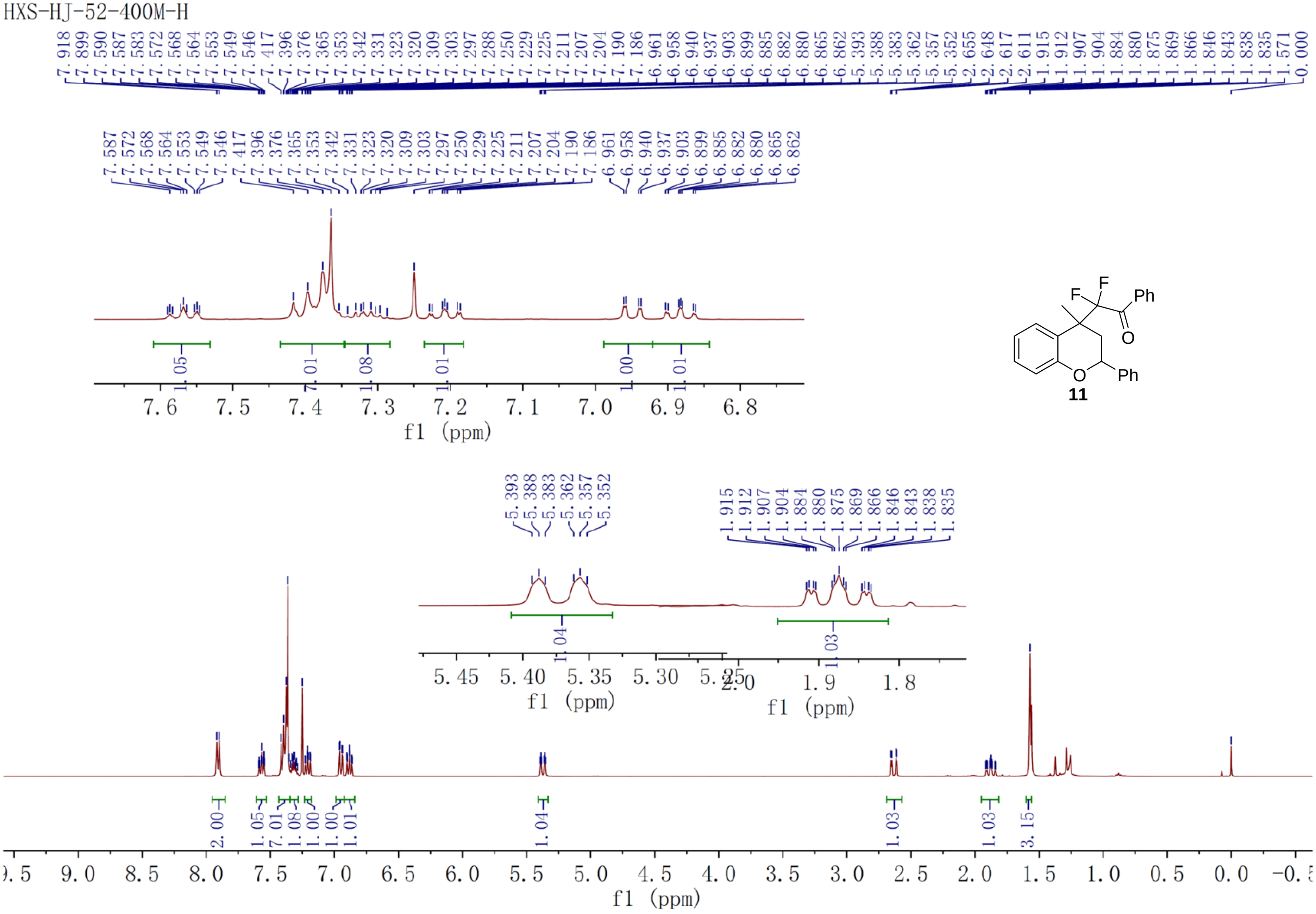

Supplementary Figure 236. ^{1}H NMR (400 MHz, $CDCl_3$) spectra for compound **11**

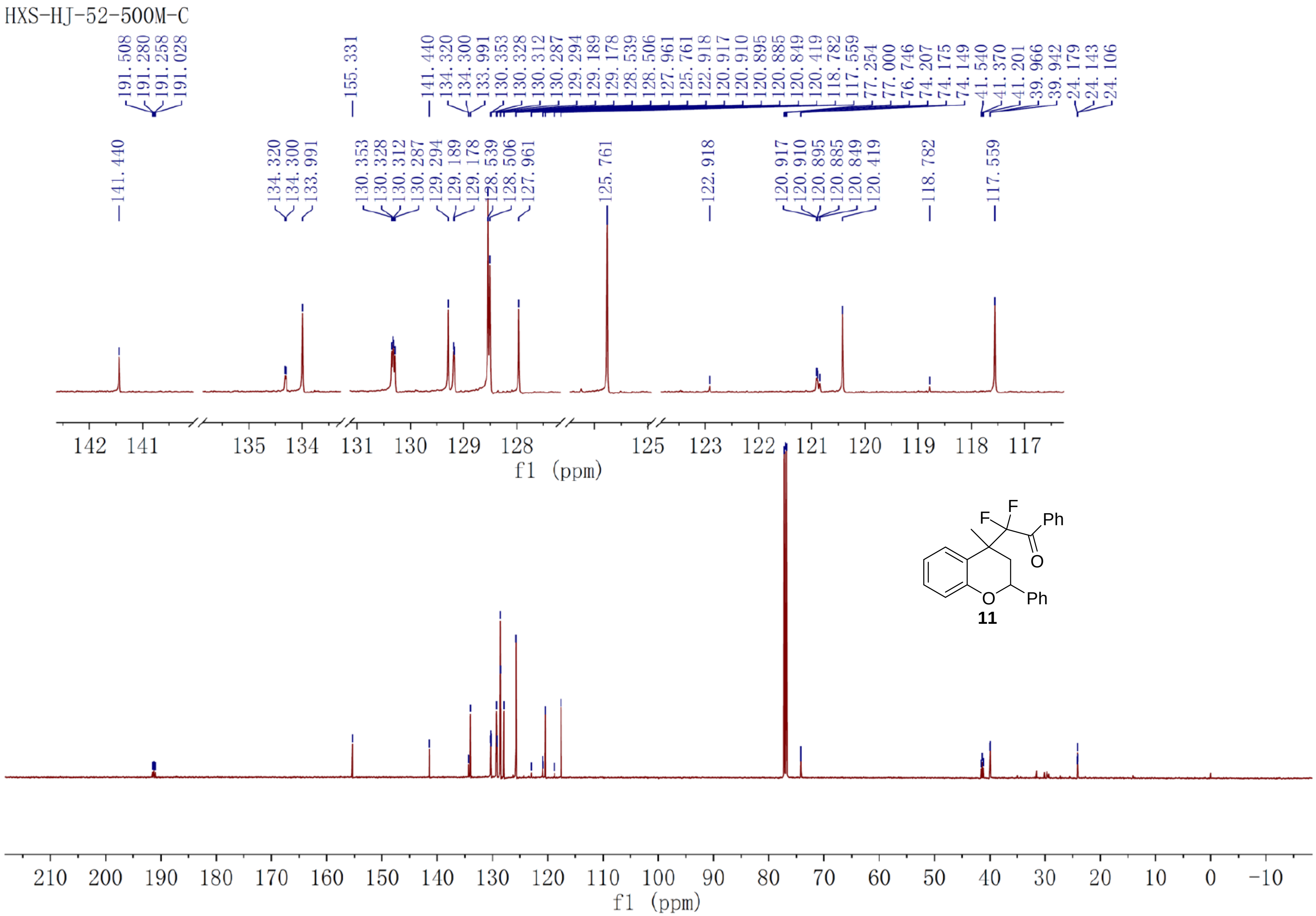

Supplementary Figure 237. ^{13}C NMR (125 MHz, $CDCl_3$) spectra for compound **11**

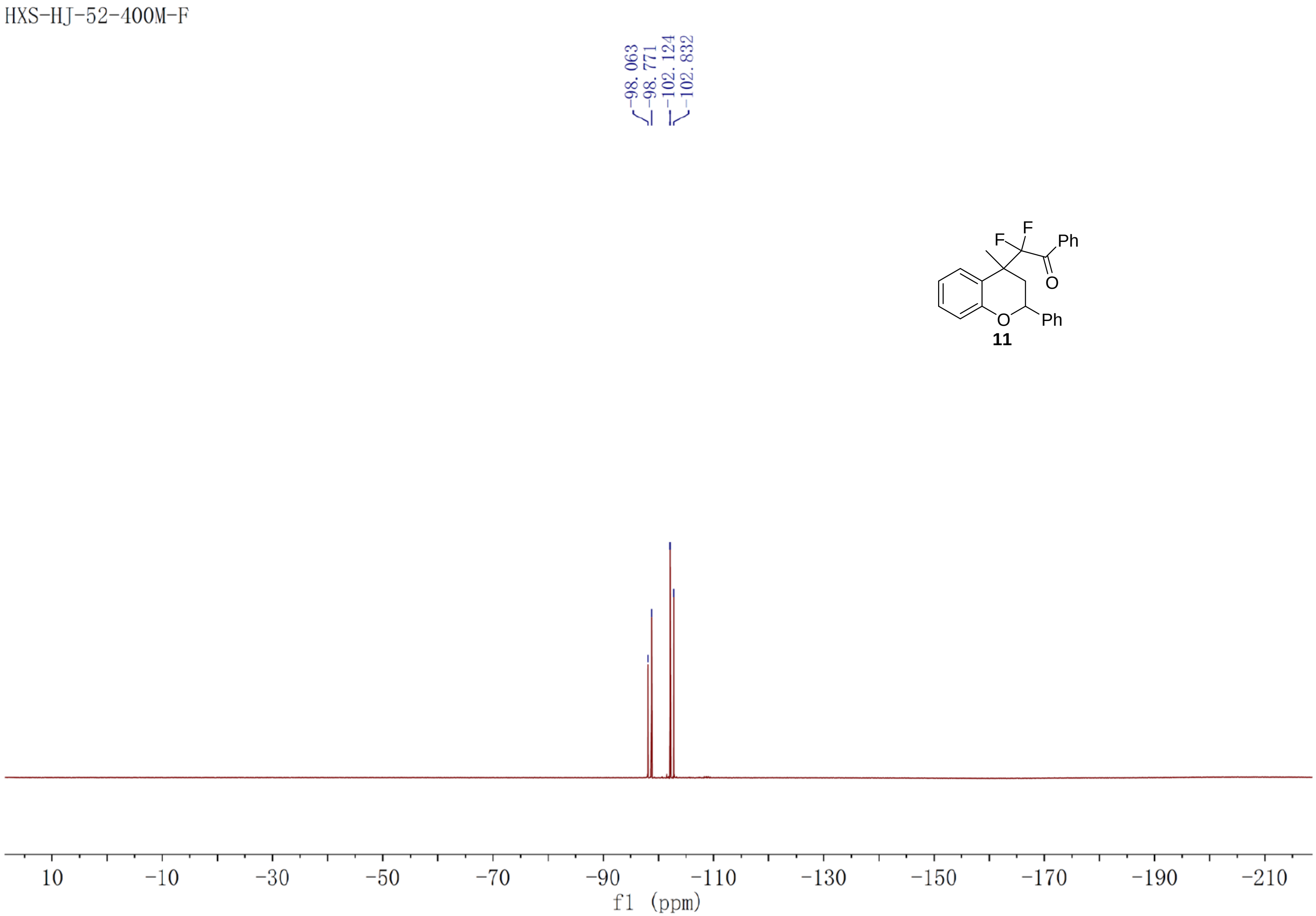

Supplementary Figure 238. ^{19}F NMR (376 MHz, $CDCl_3$) spectra for compound **11**

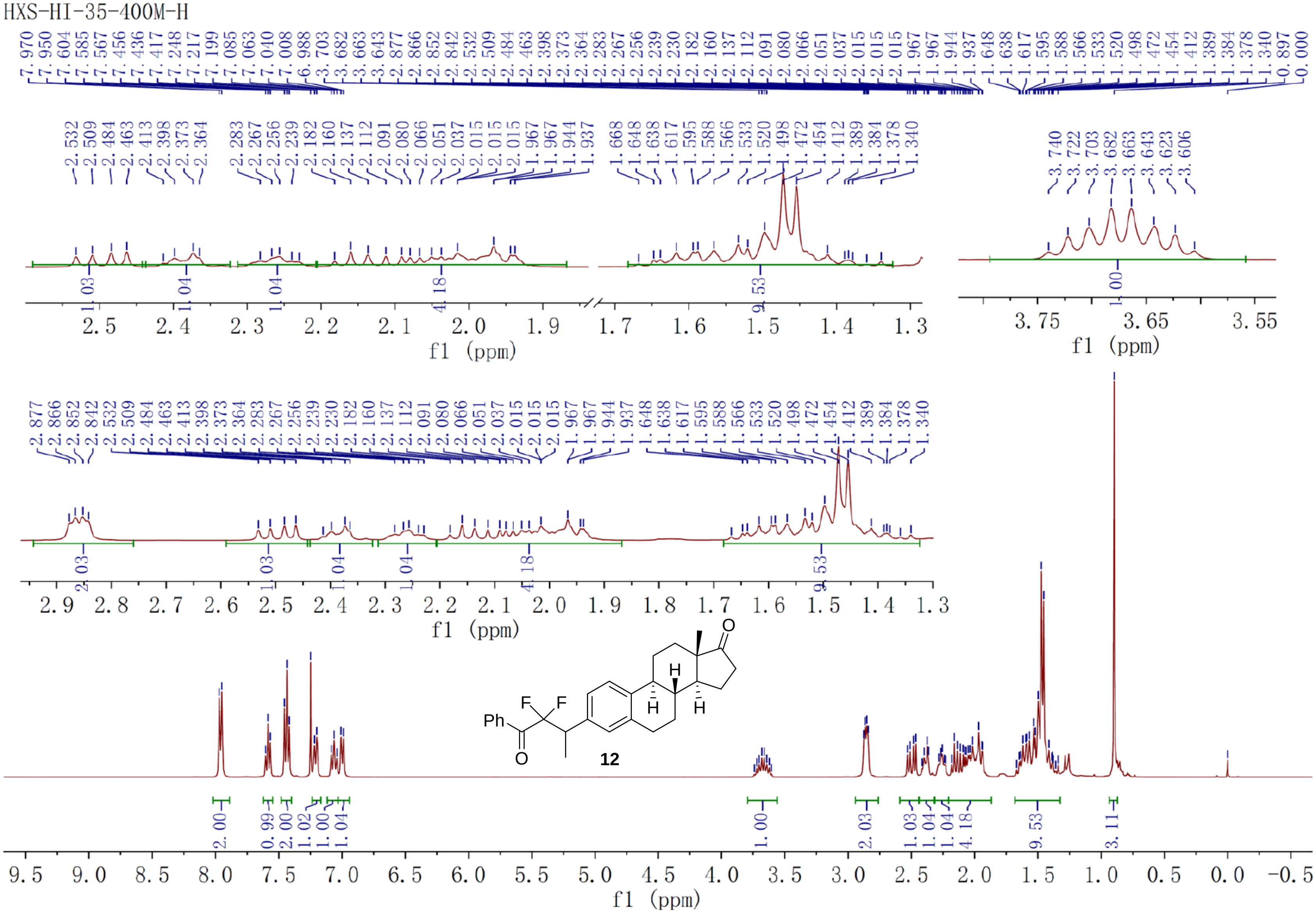

Supplementary Figure 239. ^{1}H NMR (400 MHz, $CDCl_3$) spectra for compound **12**

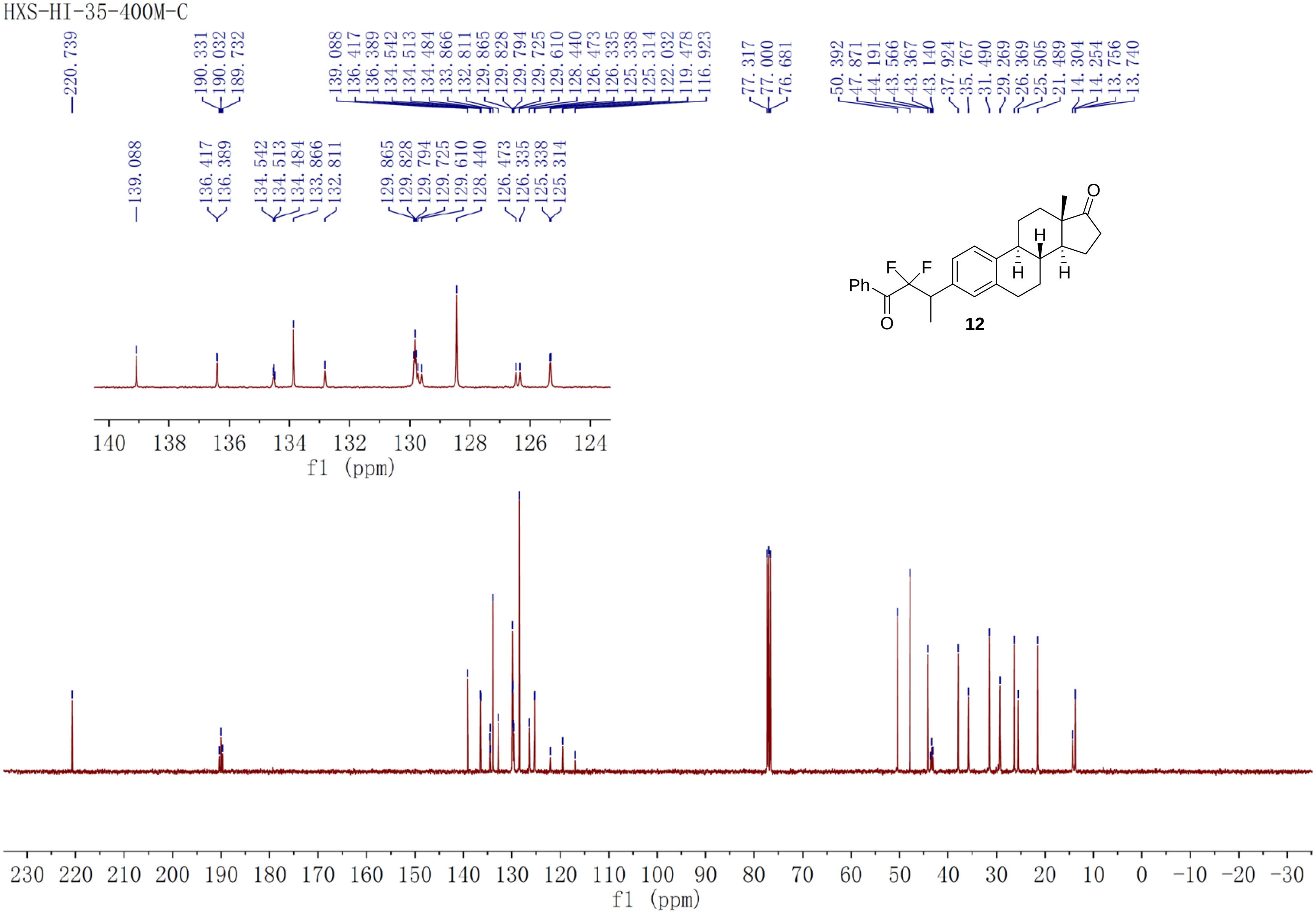

Supplementary Figure 240. ^{13}C NMR (100 MHz, $CDCl_3$) spectra for compound **12**

HXS-HI-35-400M-F

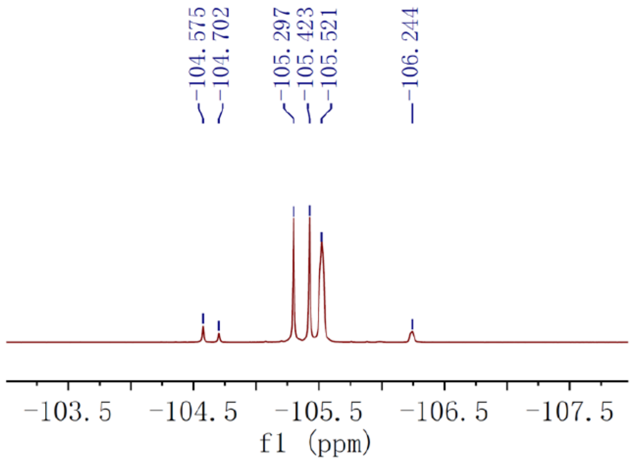

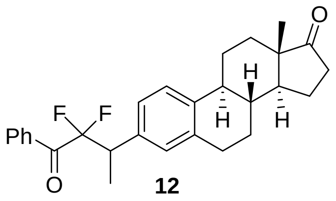

12

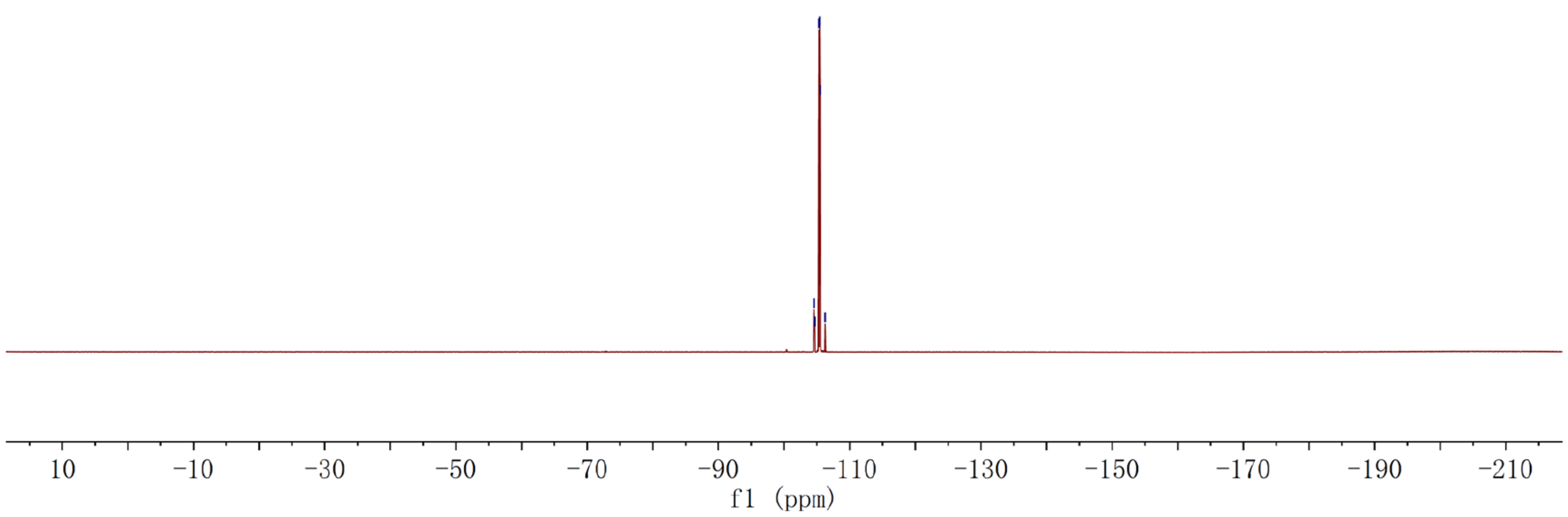

Supplementary Figure 241. ^{19}F NMR (376 MHz, $CDCl_3$) spectra for compound **12**

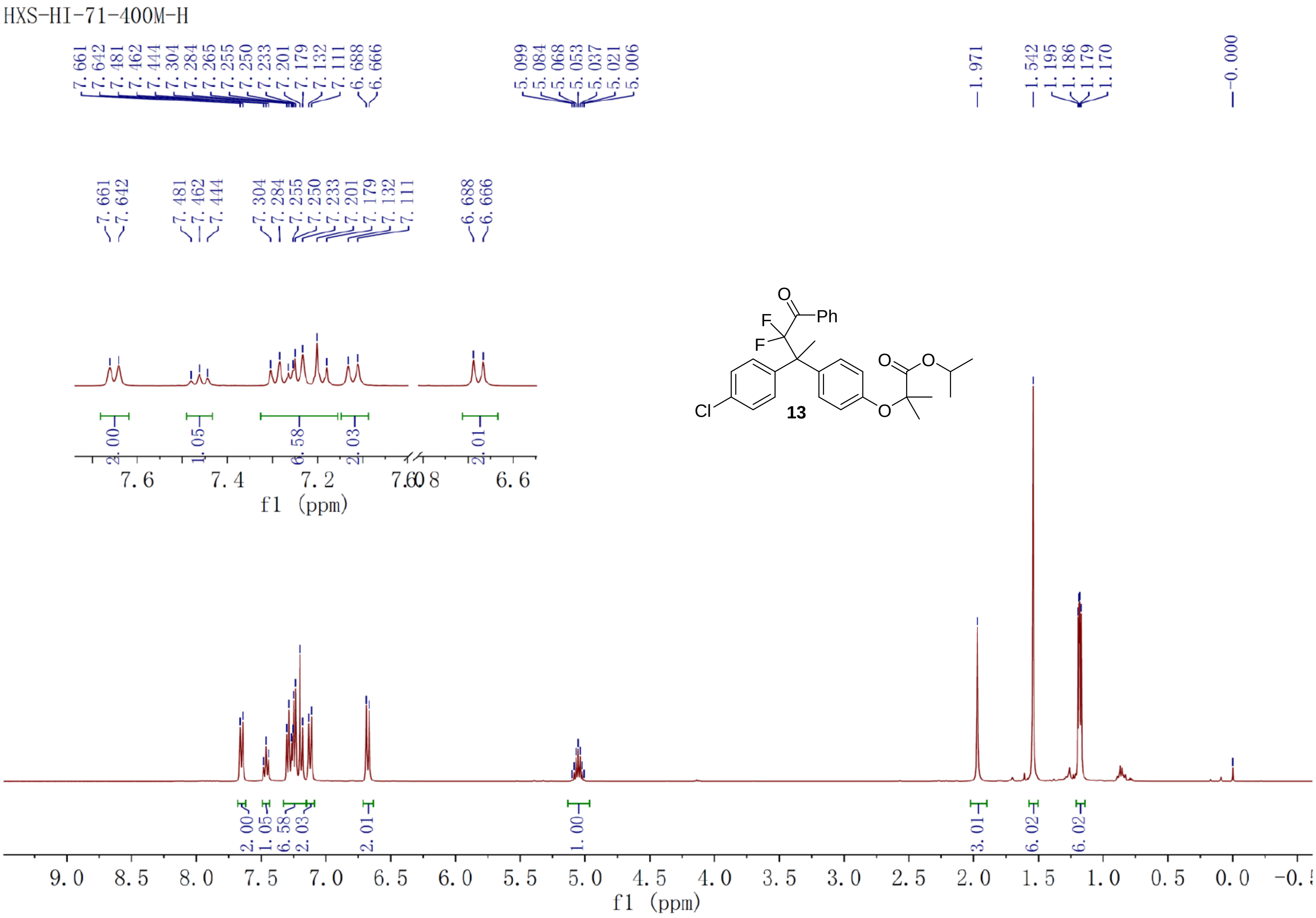

Supplementary Figure 242. ^{1}H NMR (400 MHz, $CDCl_3$) spectra for compound **13**

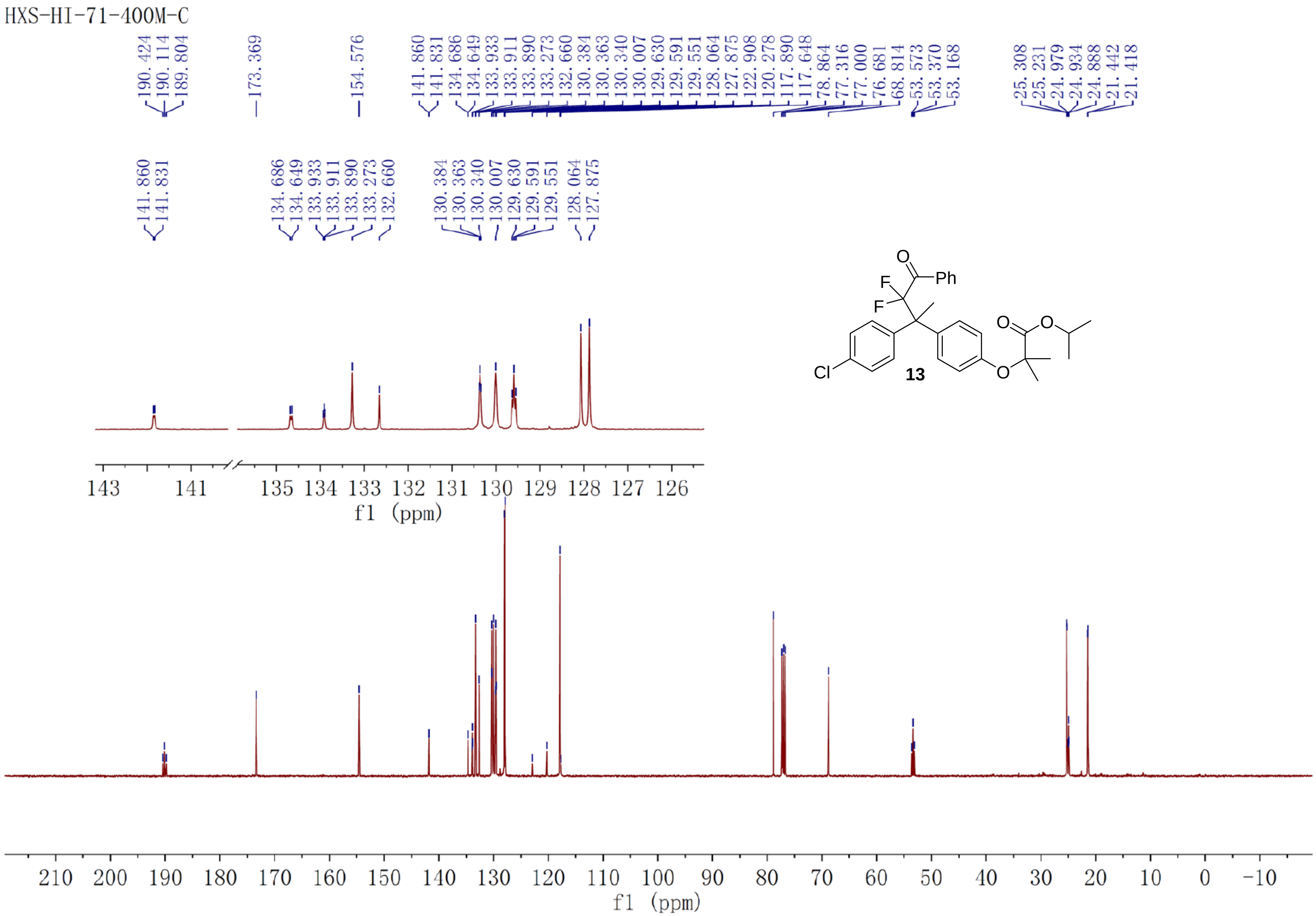

Supplementary Figure 243. ^{13}C NMR (100 MHz, $CDCl_3$) spectra for compound **13**

HXS-HI-71-400M-F

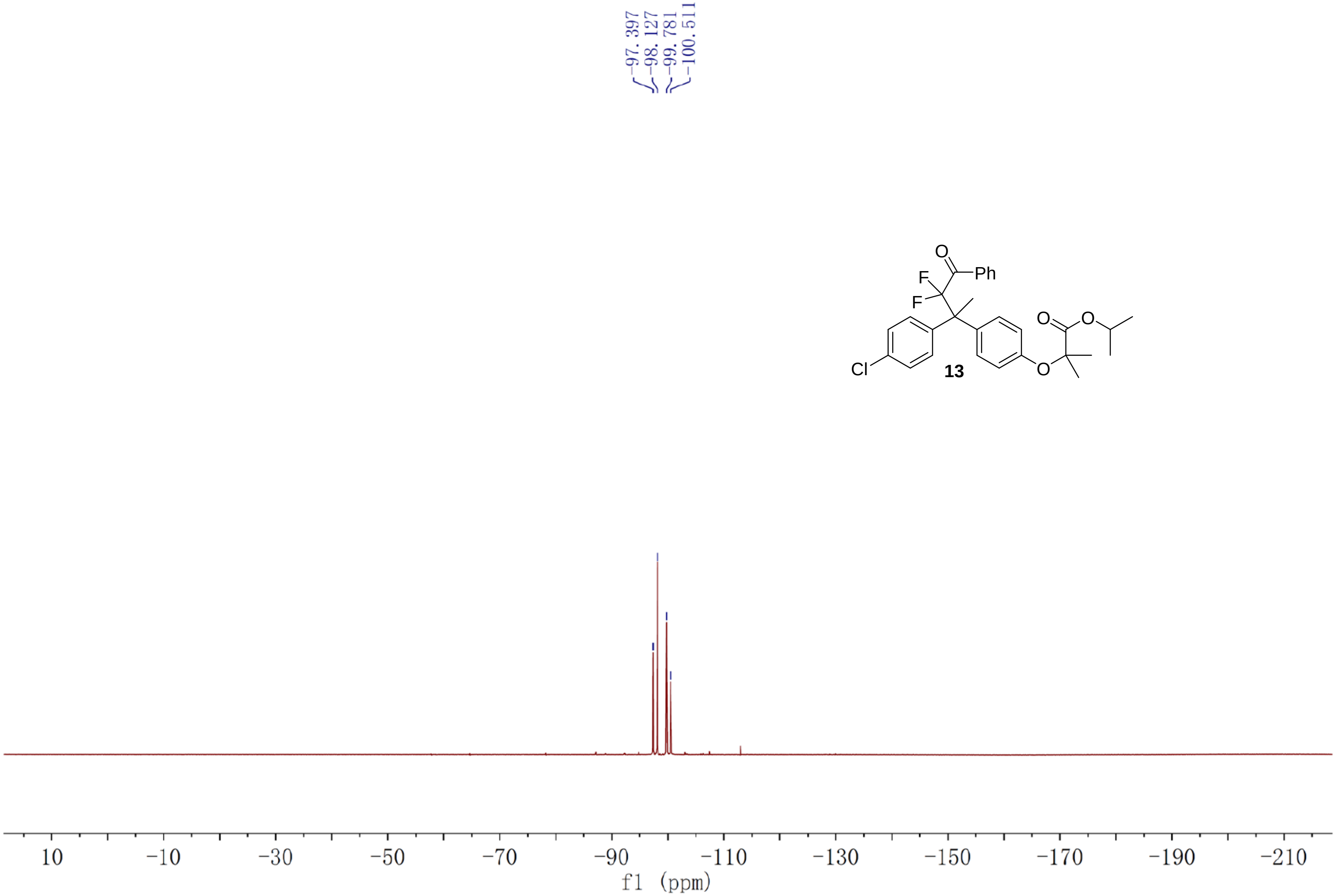

13

Supplementary Figure 244. ^{19}F NMR (376 MHz, $CDCl_3$) spectra for compound **13**

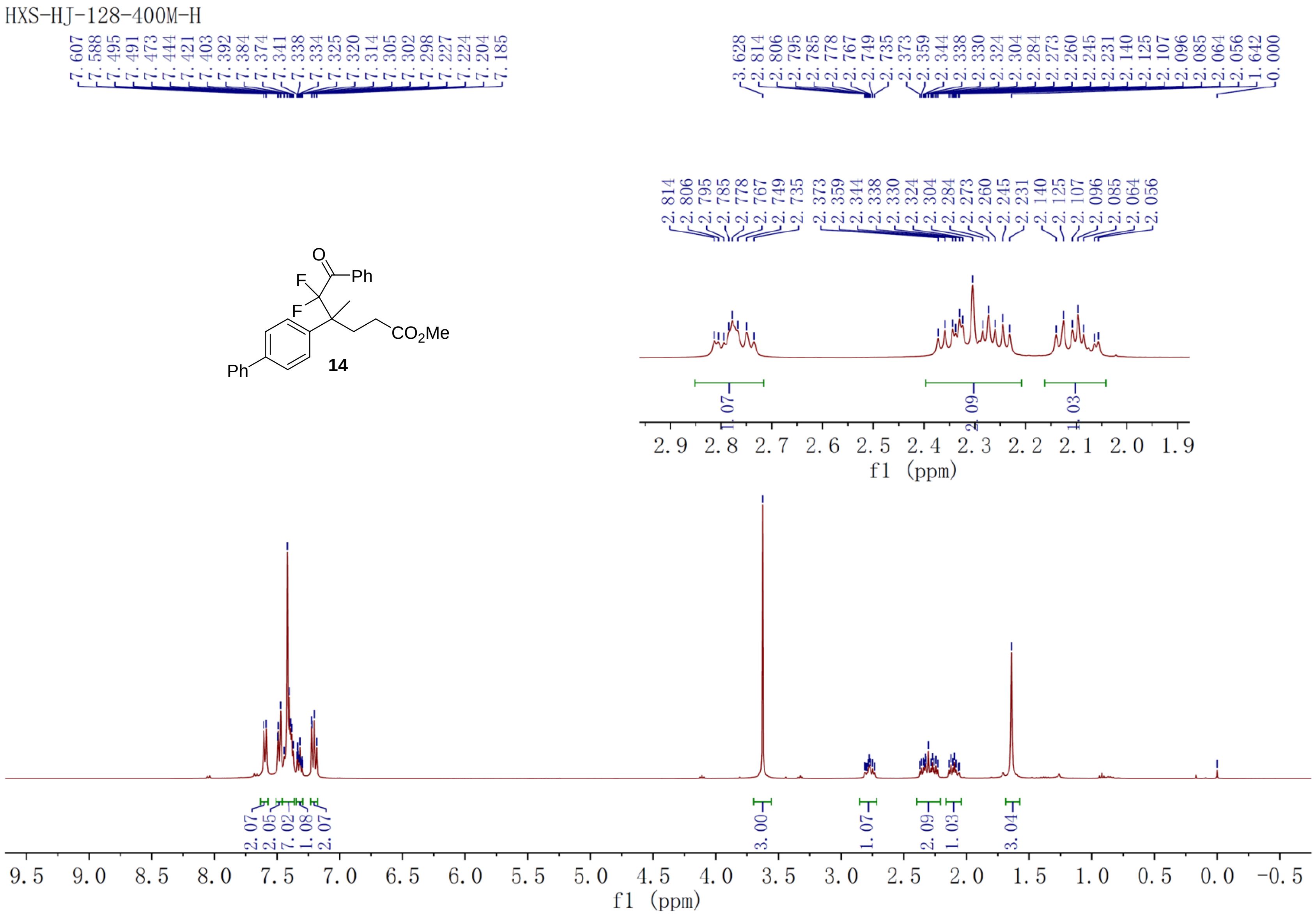

Supplementary Figure 245. ^{1}H NMR (400 MHz, $CDCl_3$) spectra for compound **14**

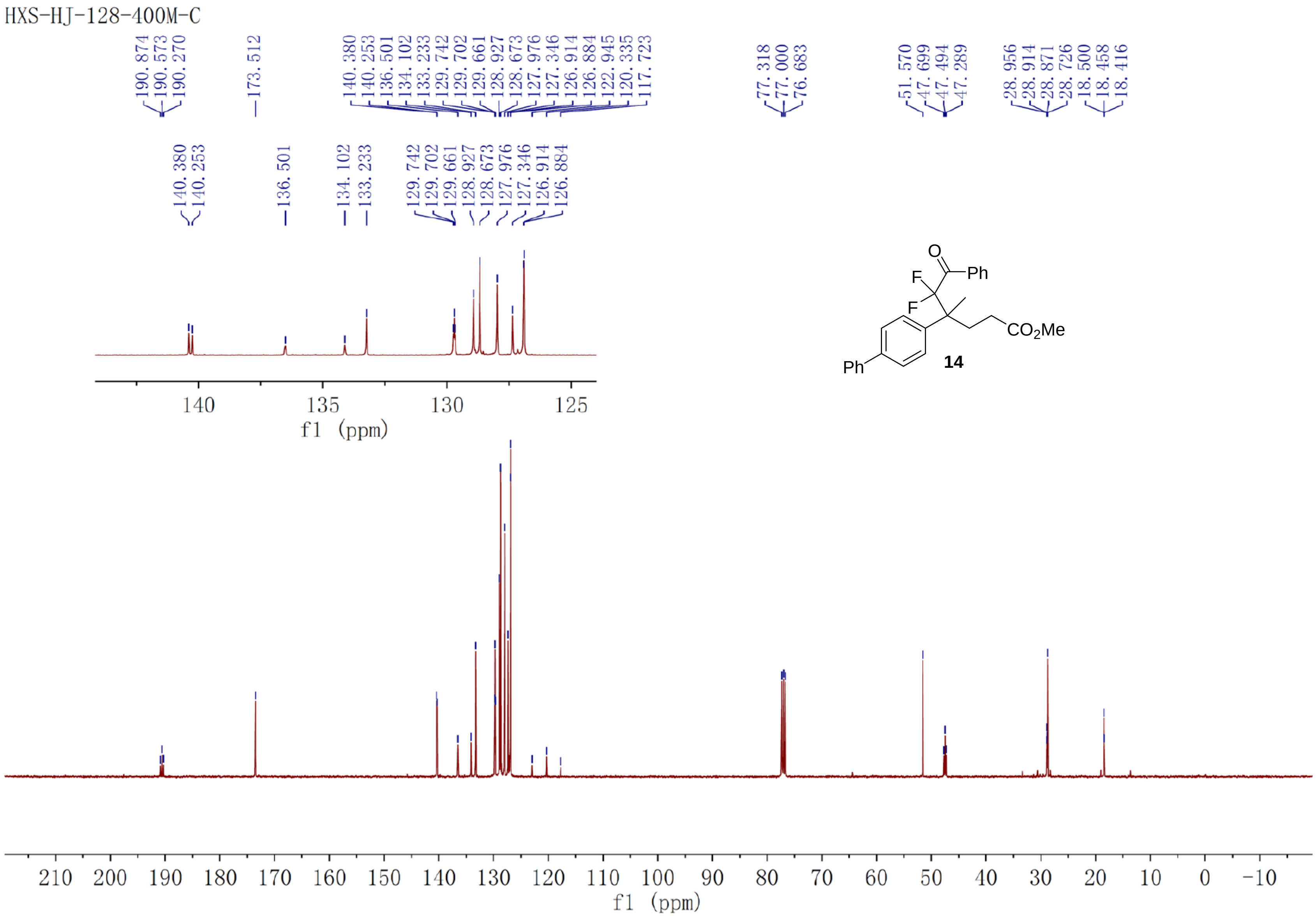

Supplementary Figure 246. ^{13}C NMR (100 MHz, $CDCl_3$) spectra for compound **14**

HXS-HJ-128-400M-F

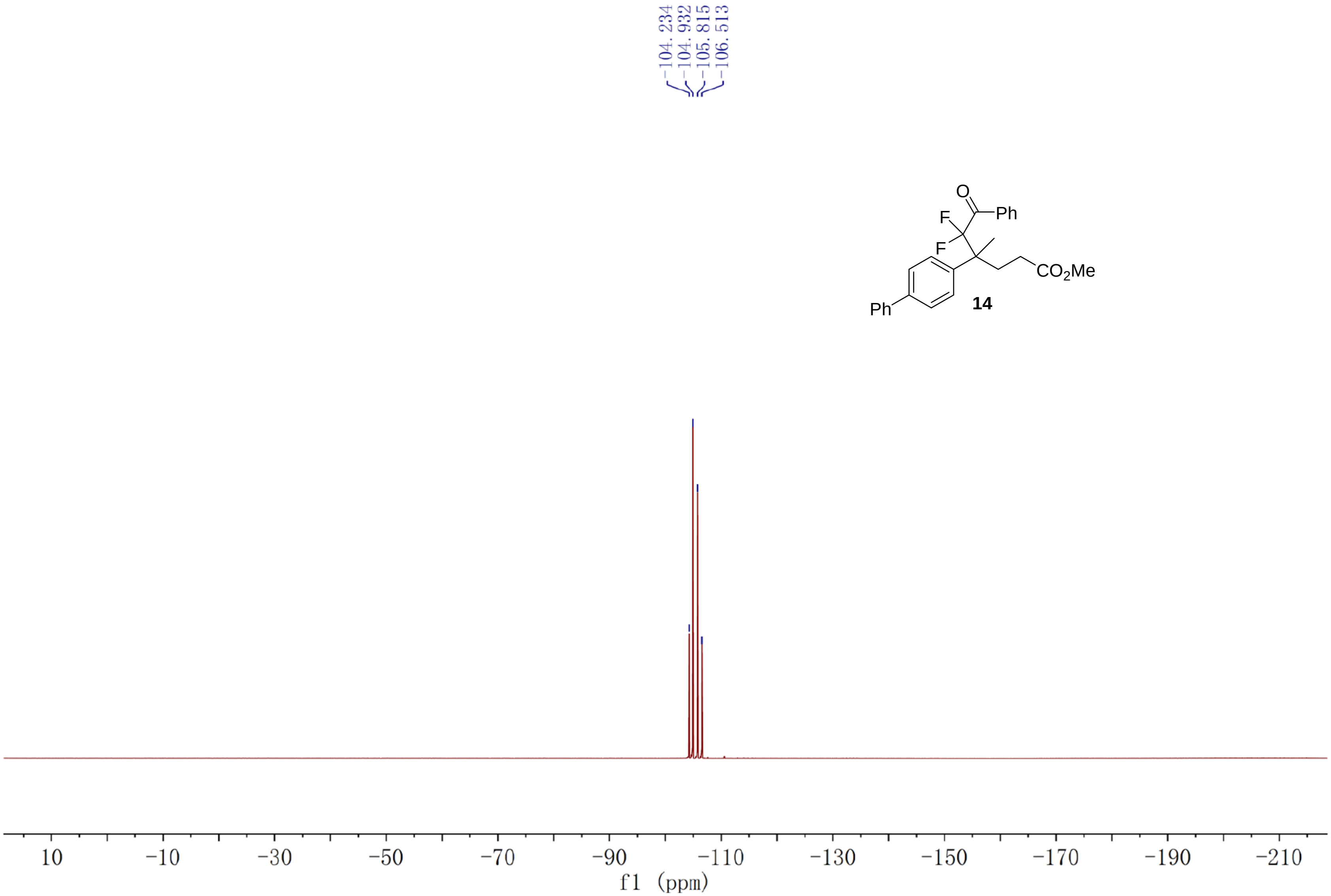

14

Supplementary Figure 247. ^{19}F NMR (376 MHz, $CDCl_3$) spectra for compound **14**

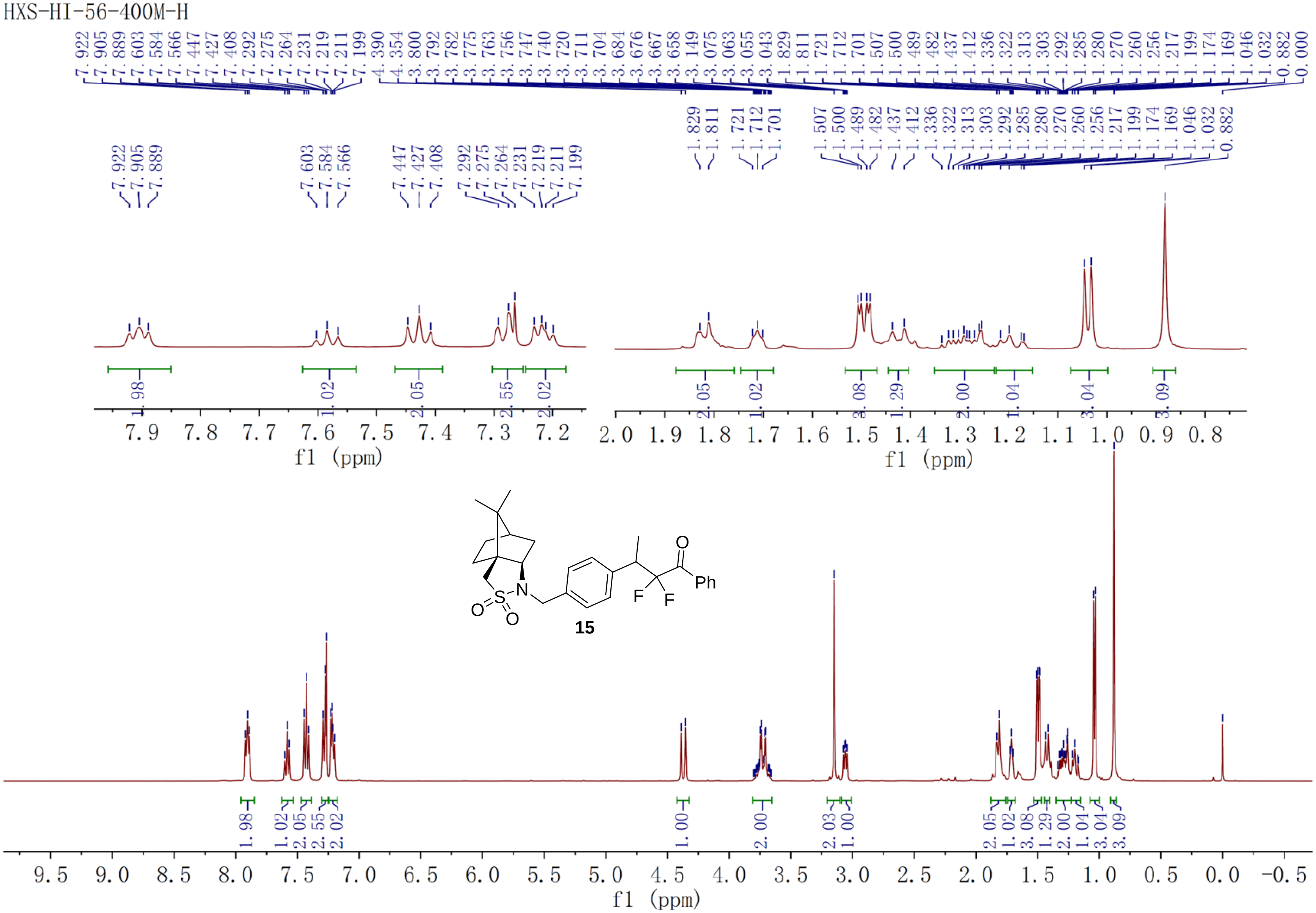

Supplementary Figure 248. ^{1}H NMR (400 MHz, $CDCl_3$) spectra for compound **15**

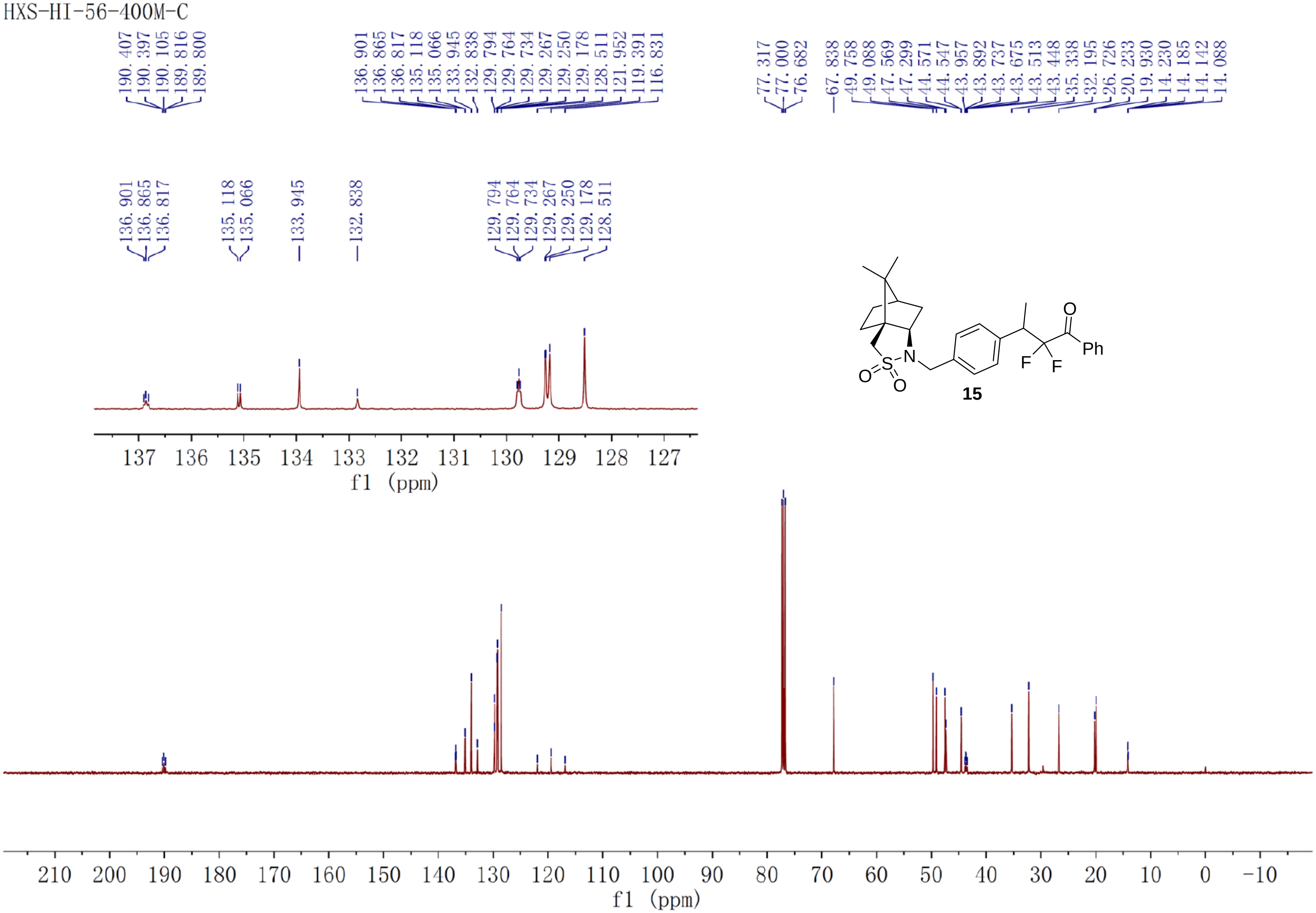

Supplementary Figure 249. ^{13}C NMR (100 MHz, $CDCl_3$) spectra for compound **15**

HXS-HI-56-400M-F

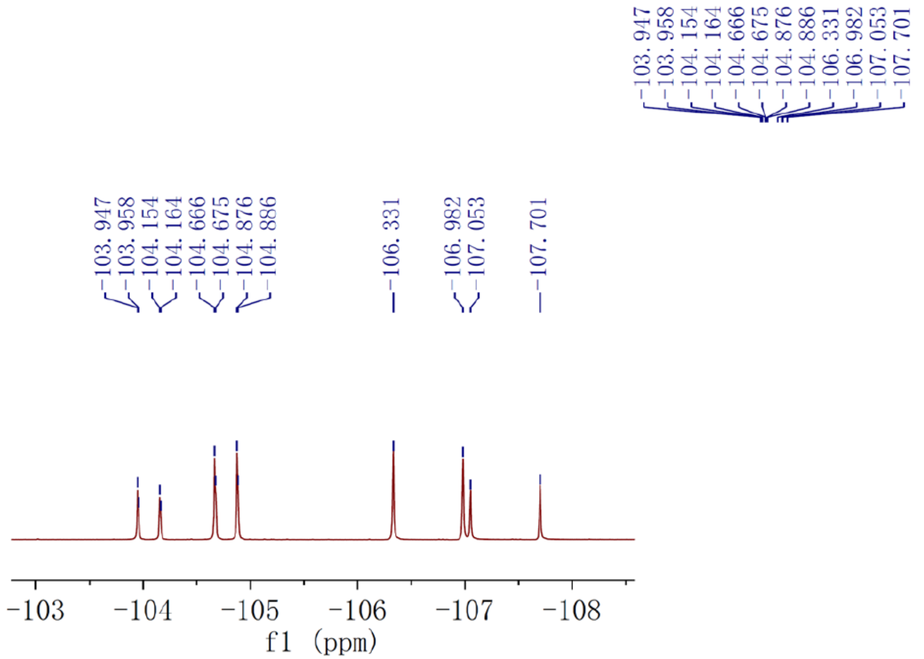

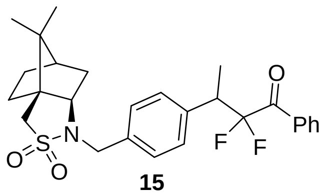

15

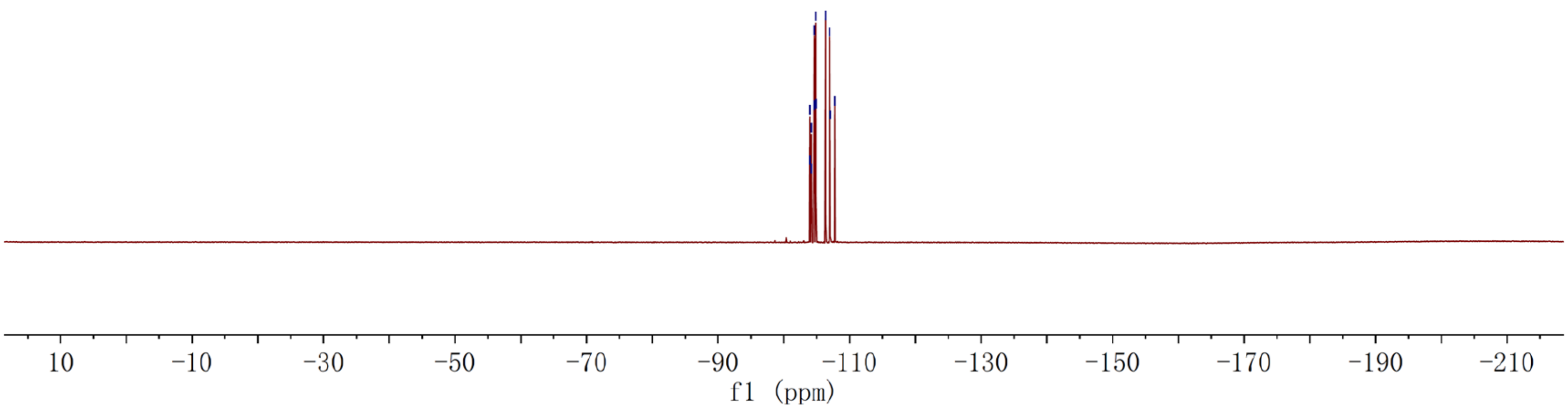

Supplementary Figure 250. ^{19}F NMR (376 MHz, $CDCl_3$) spectra for compound **15**

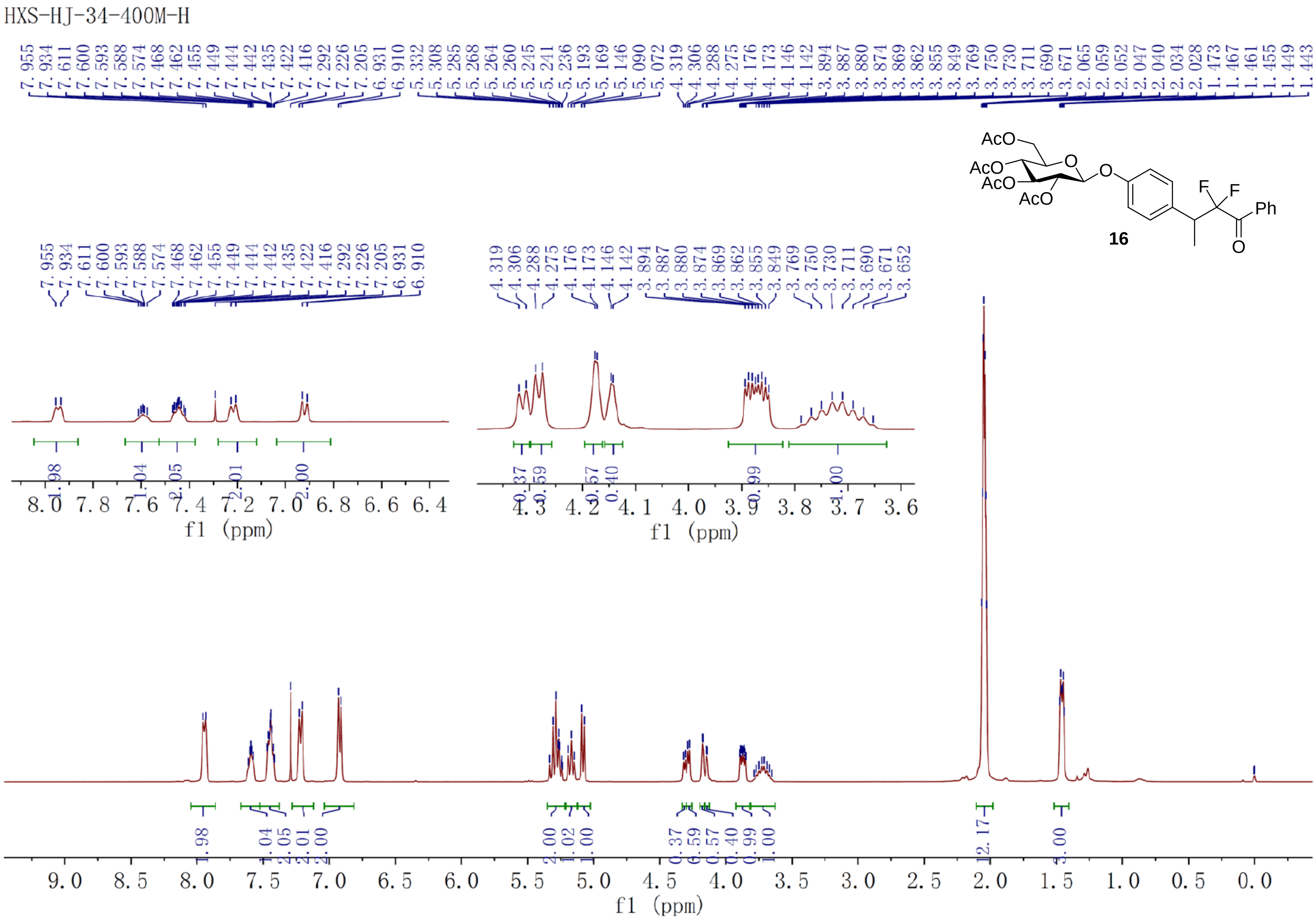

Supplementary Figure 251. ^{1}H NMR (400 MHz, $CDCl_3$) spectra for compound **16**

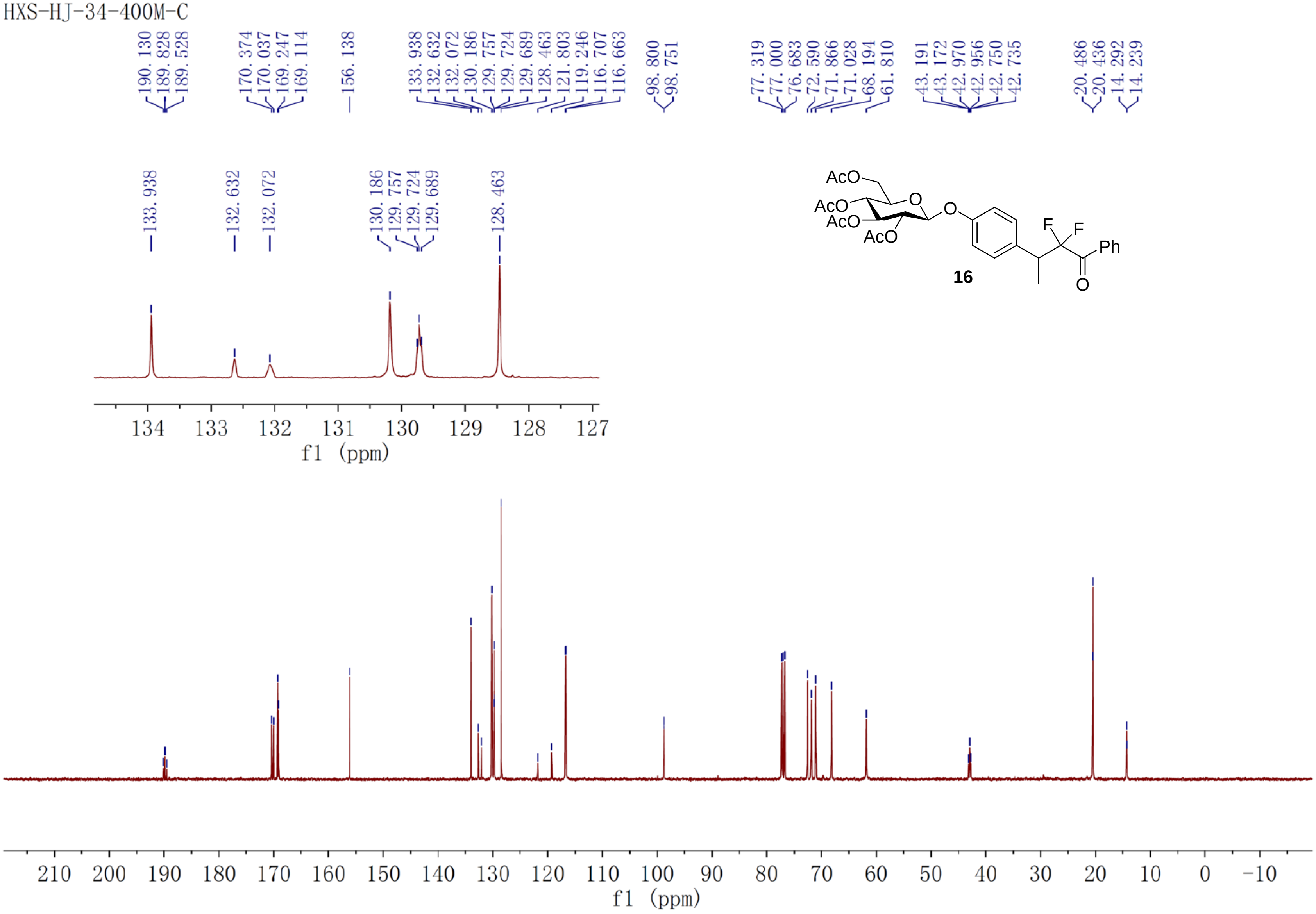

Supplementary Figure 252. ^{13}C NMR (100 MHz, $CDCl_3$) spectra for compound **16**

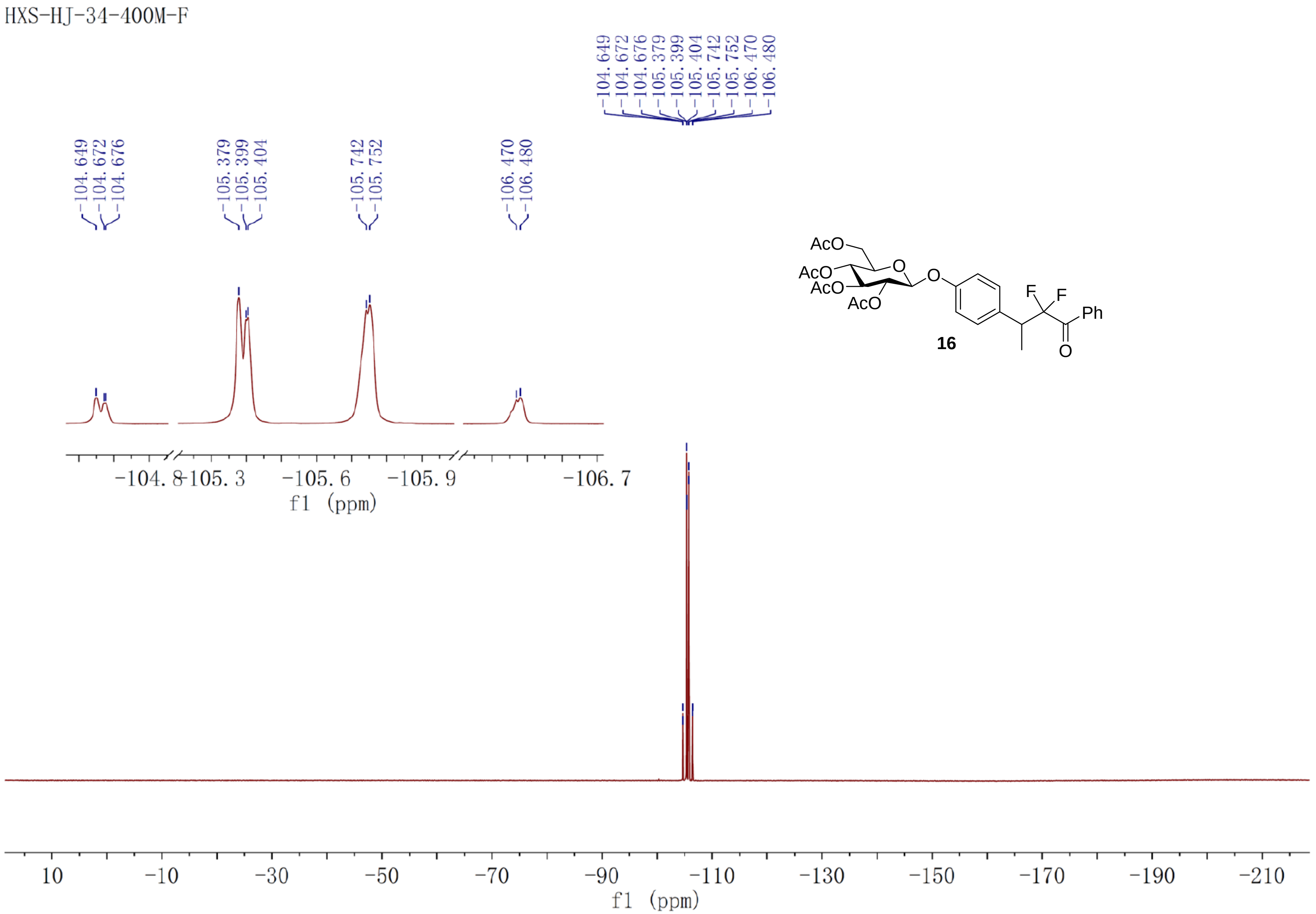

Supplementary Figure 253. ^{19}F NMR (376 MHz, $CDCl_3$) spectra for compound **16**

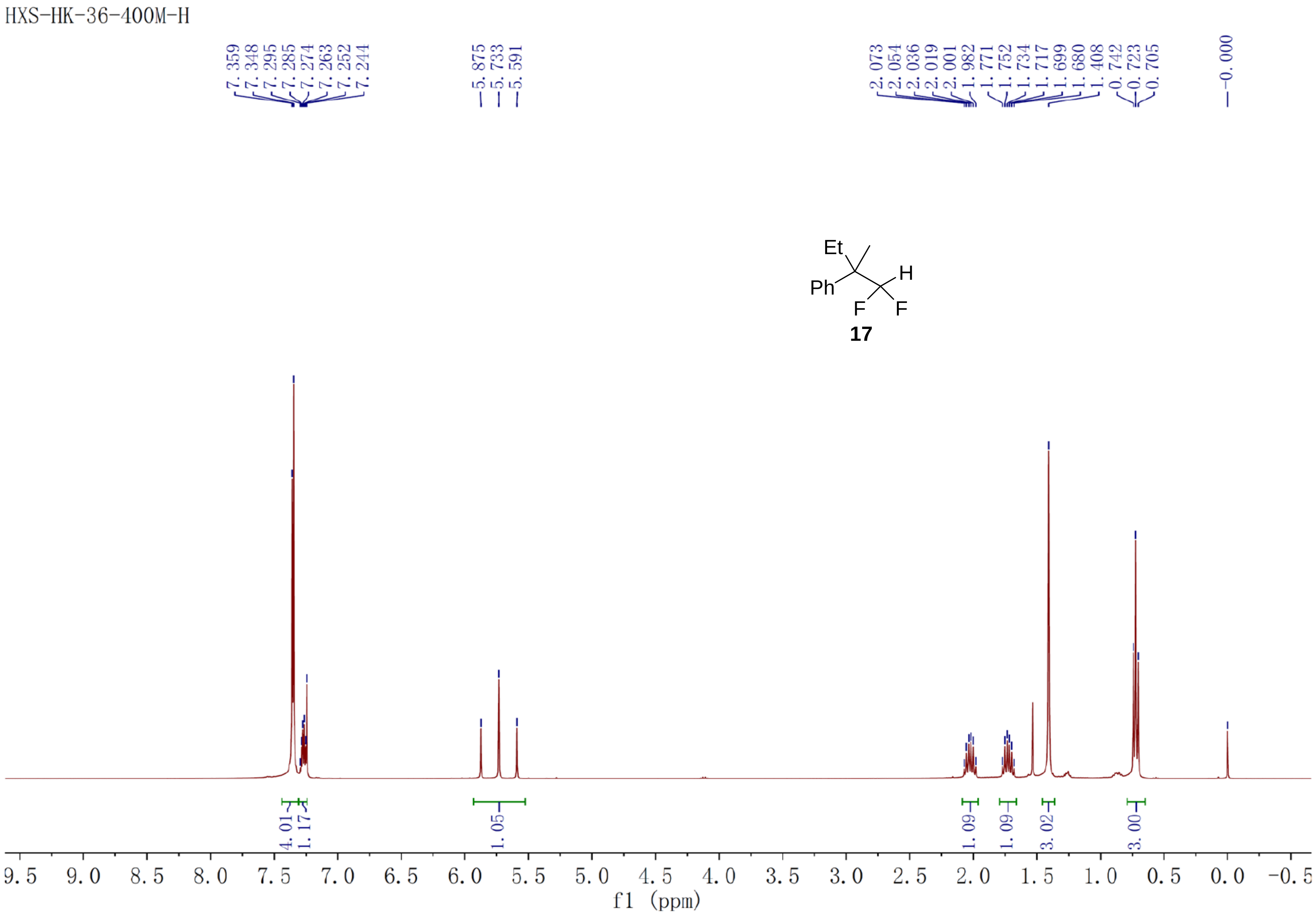

Supplementary Figure 254. ^{1}H NMR (400 MHz, $CDCl_3$) spectra for compound **17**

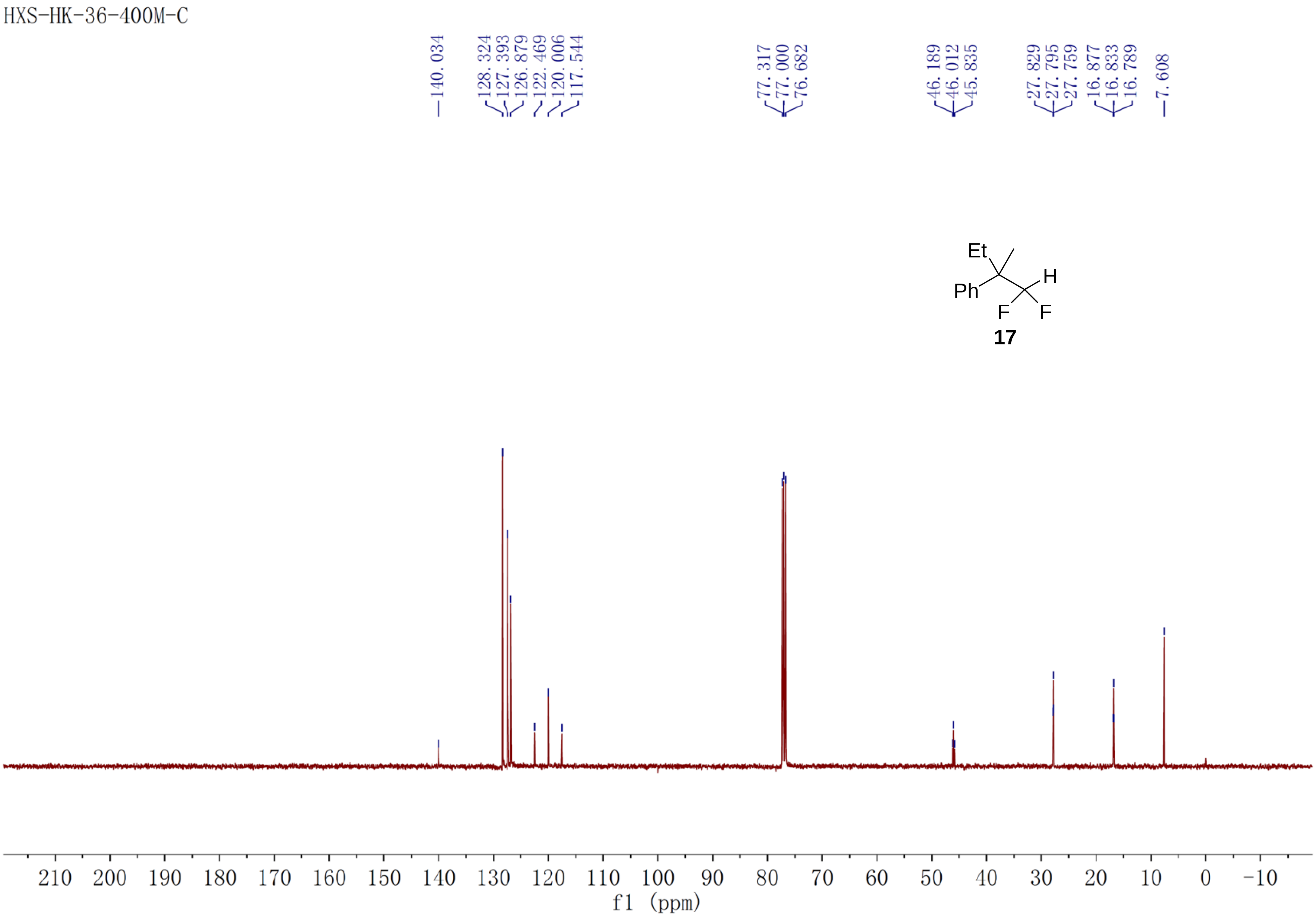

Supplementary Figure 255. ^{13}C NMR (100 MHz, $CDCl_3$) spectra for compound **17**

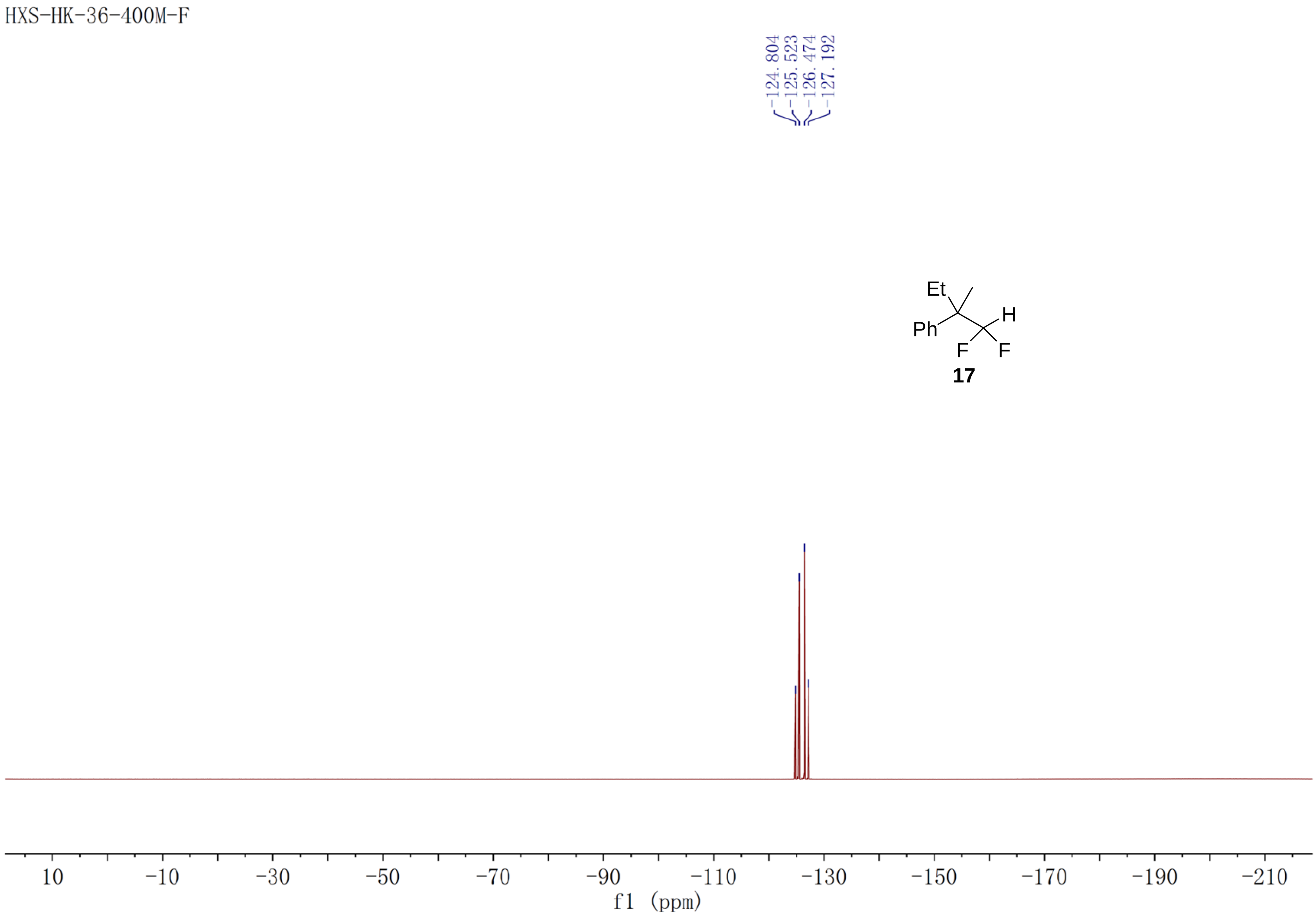

Supplementary Figure 256. ^{19}F NMR (376 MHz, $CDCl_3$) spectra for compound **17**

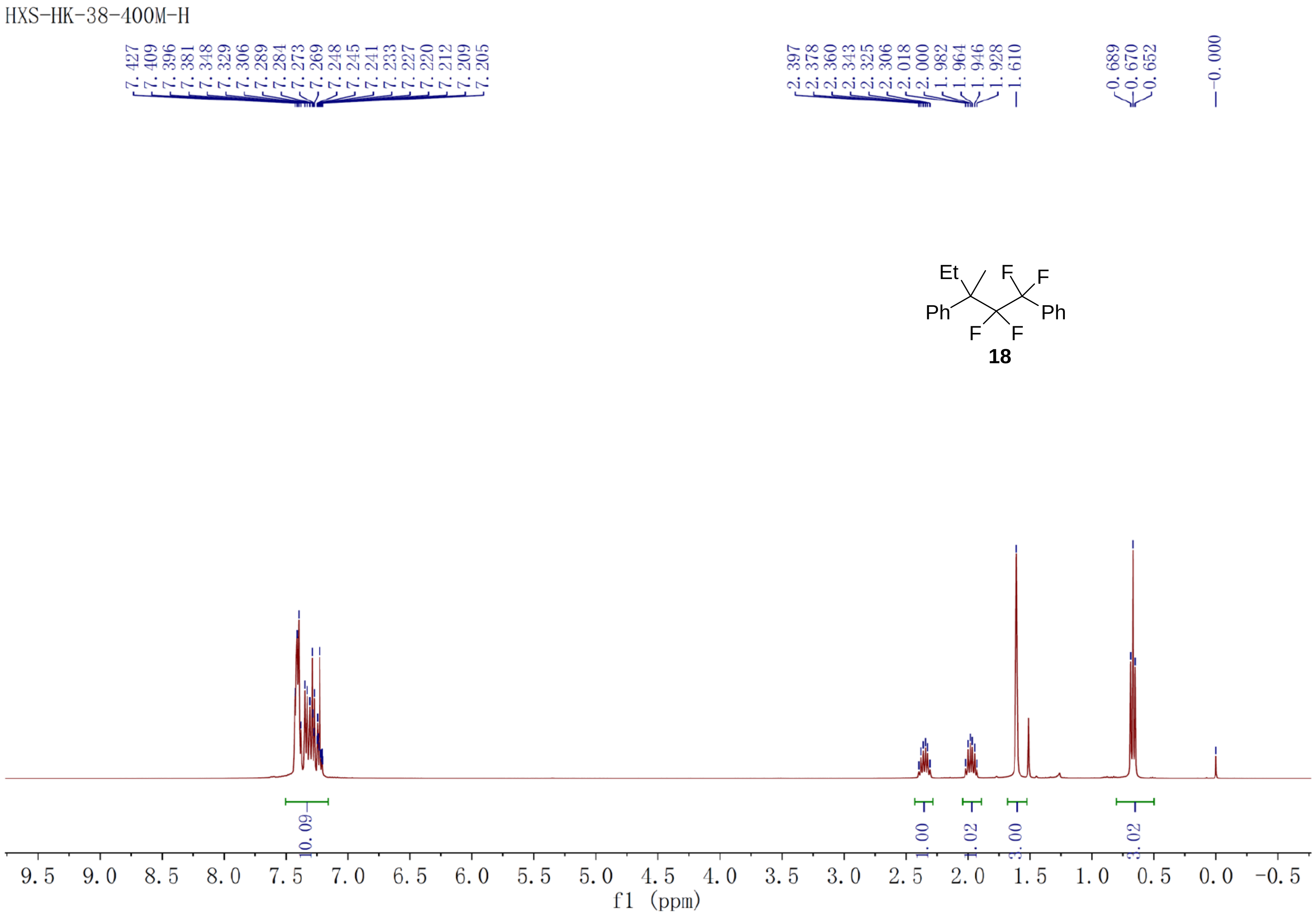

Supplementary Figure 257. ^{1}H NMR (400 MHz, $CDCl_3$) spectra for compound **18**

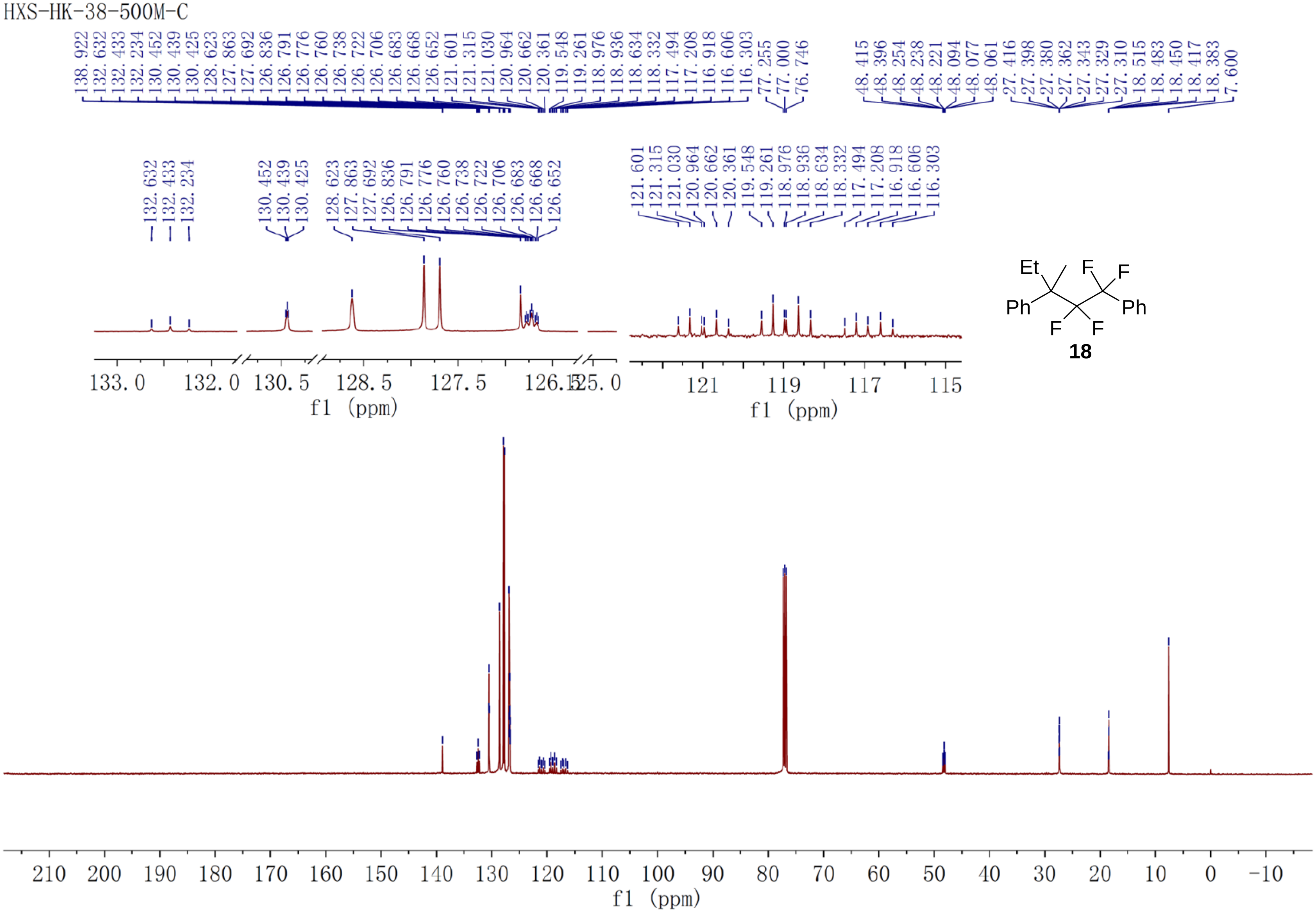

Supplementary Figure 258. ^{13}C NMR (125 MHz, $CDCl_3$) spectra for compound **18**

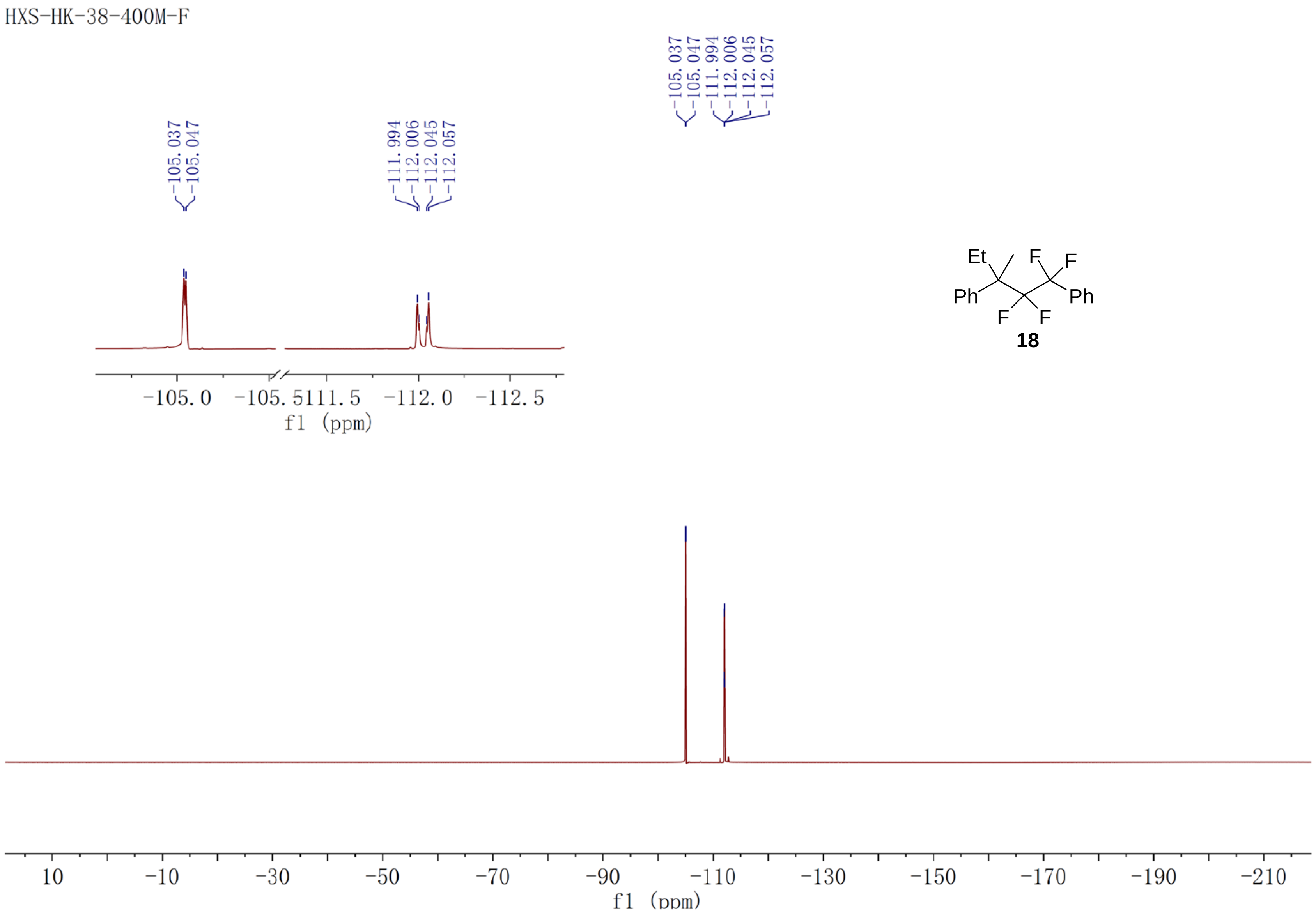

Supplementary Figure 259. ^{19}F NMR (376 MHz, $CDCl_3$) spectra for compound **18**

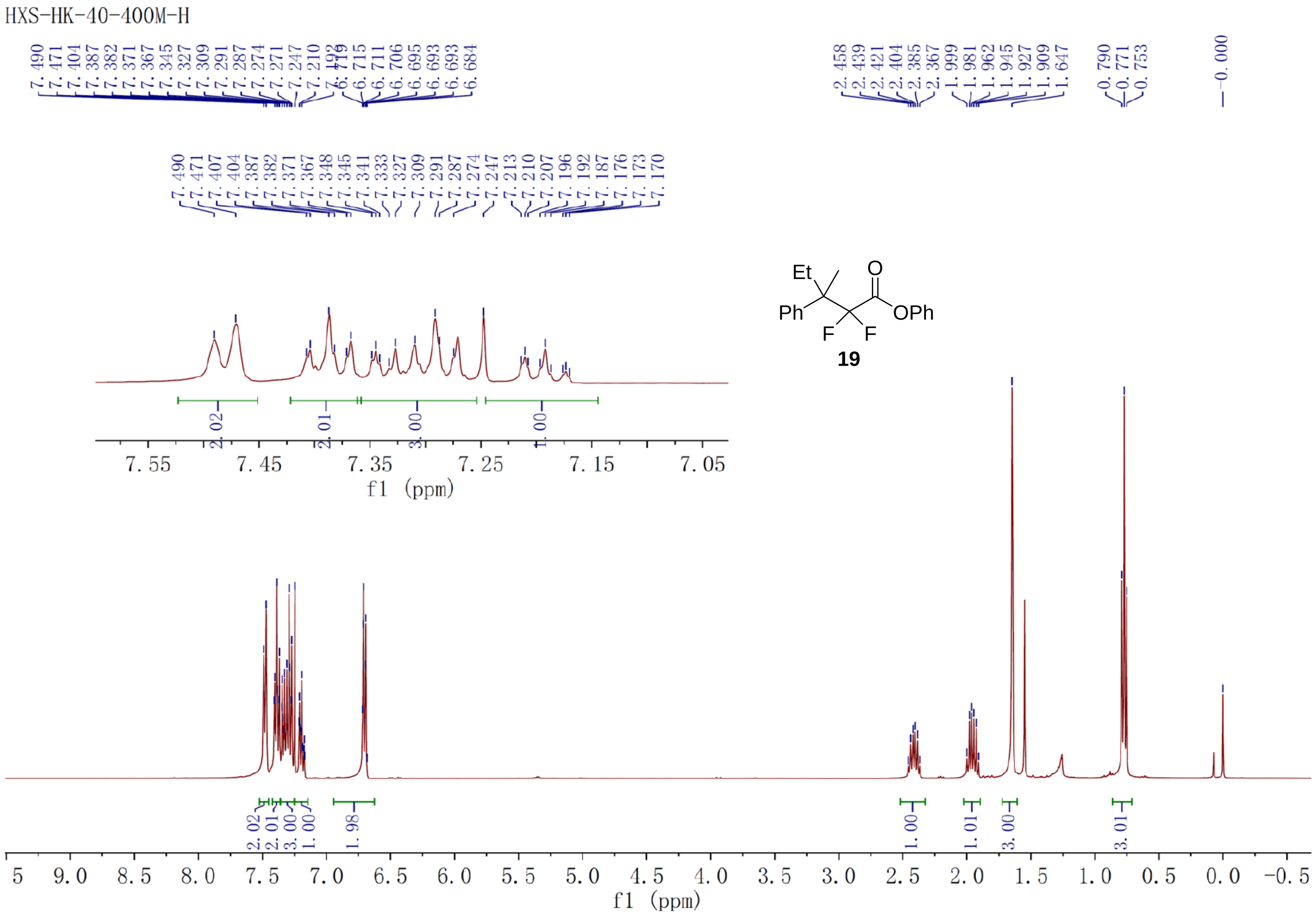

Supplementary Figure 260. ^{1}H NMR (400 MHz, $CDCl_3$) spectra for compound **19**

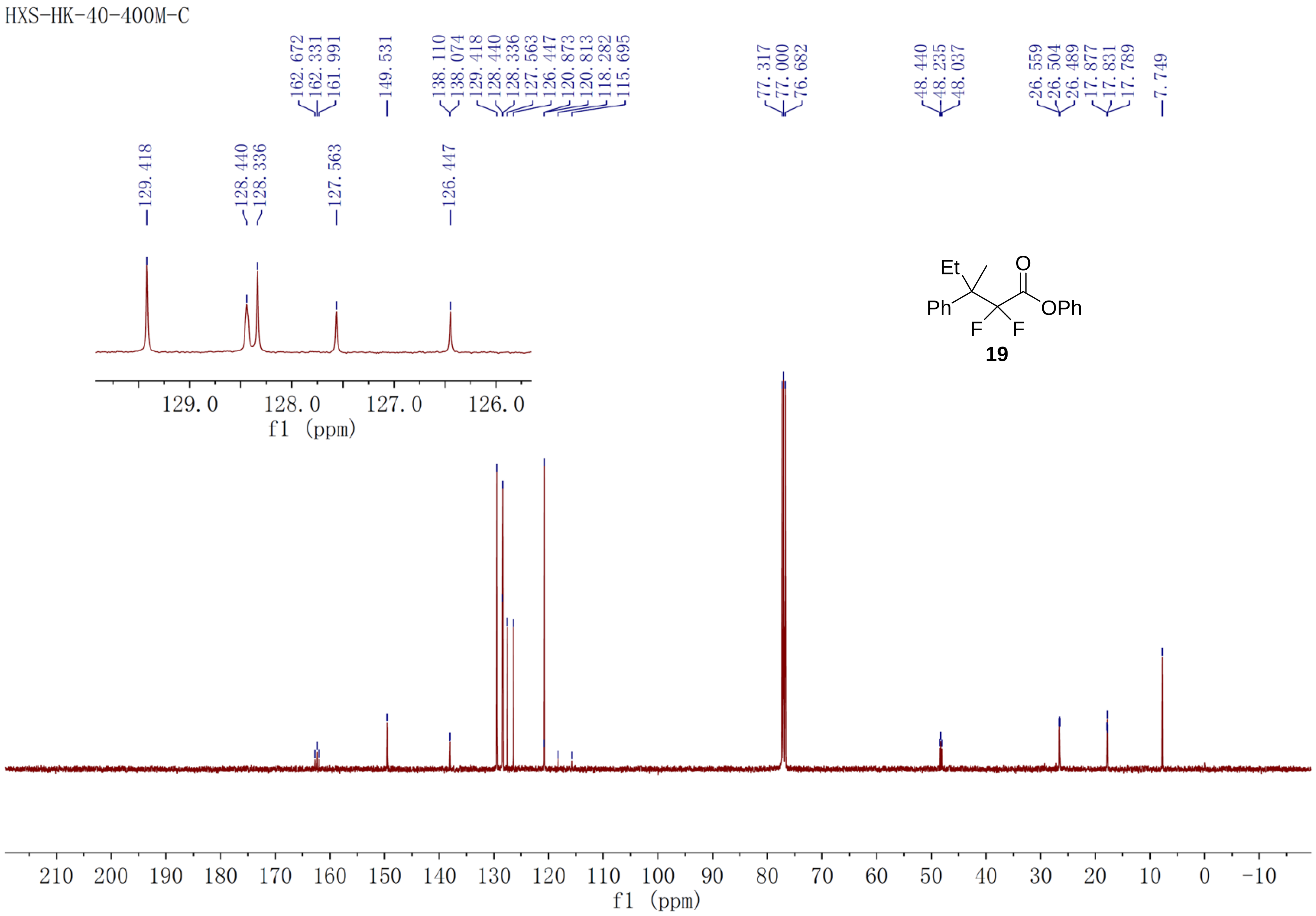

Supplementary Figure 261. ^{13}C NMR (100 MHz, $CDCl_3$) spectra for compound **19**

HXS-HK-40-400M-F

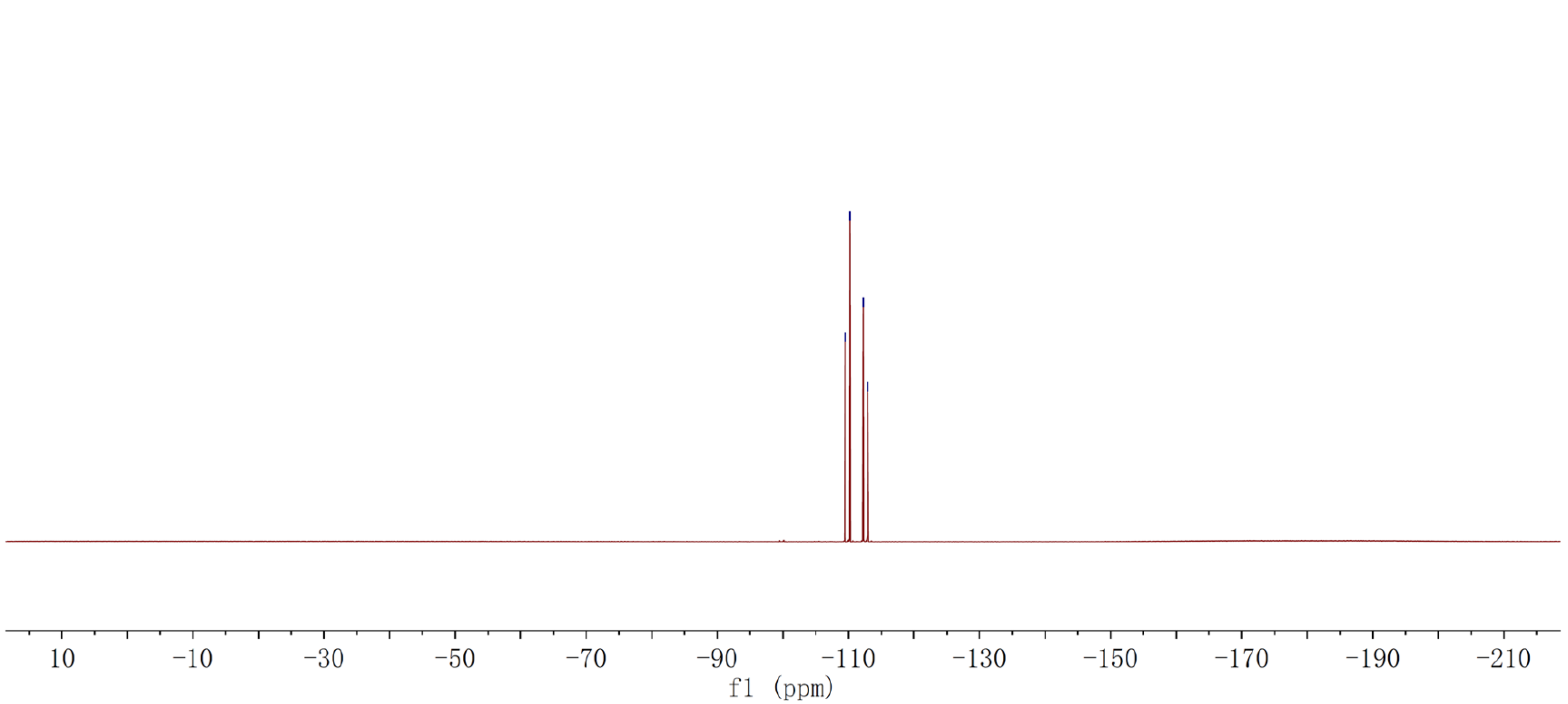

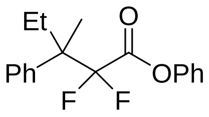

19

Supplementary Figure 262. ^{19}F NMR (376 MHz, $CDCl_3$) spectra for compound **19**

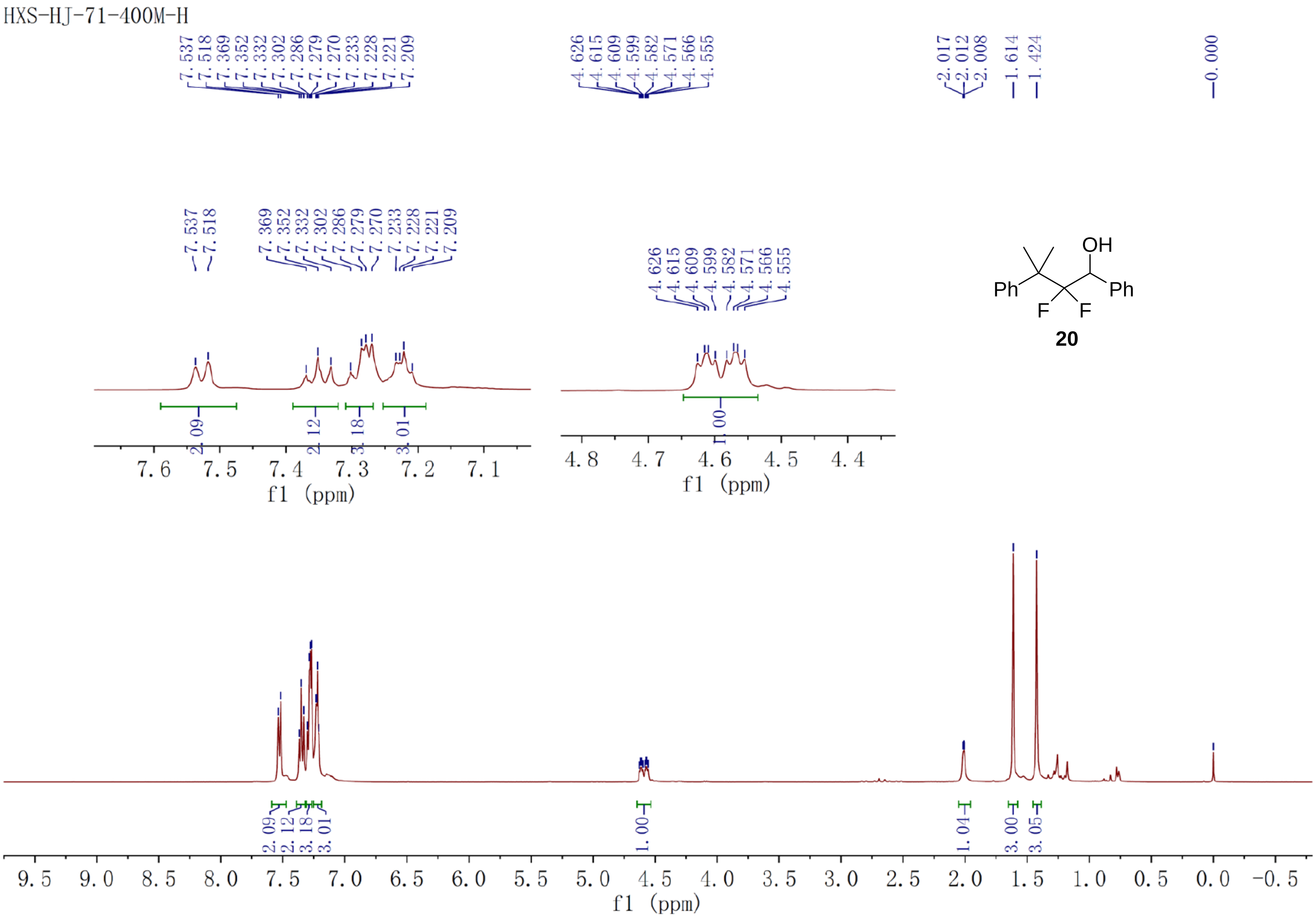

Supplementary Figure 263. ^{1}H NMR (400 MHz, $CDCl_3$) spectra for compound **20**

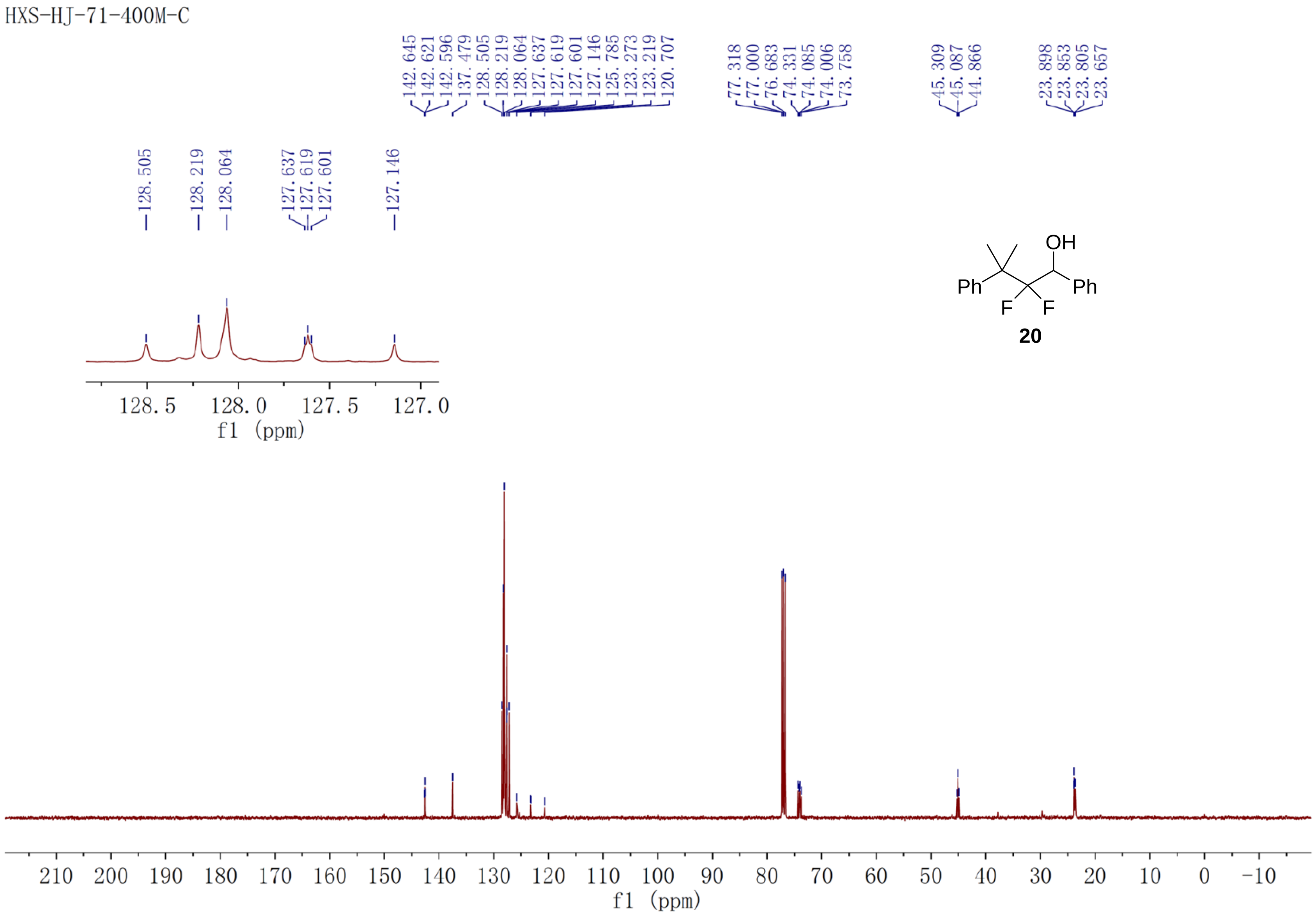

Supplementary Figure 264. ^{13}C NMR (100 MHz, $CDCl_3$) spectra for compound **20**

HXS-HJ-71-400M-F

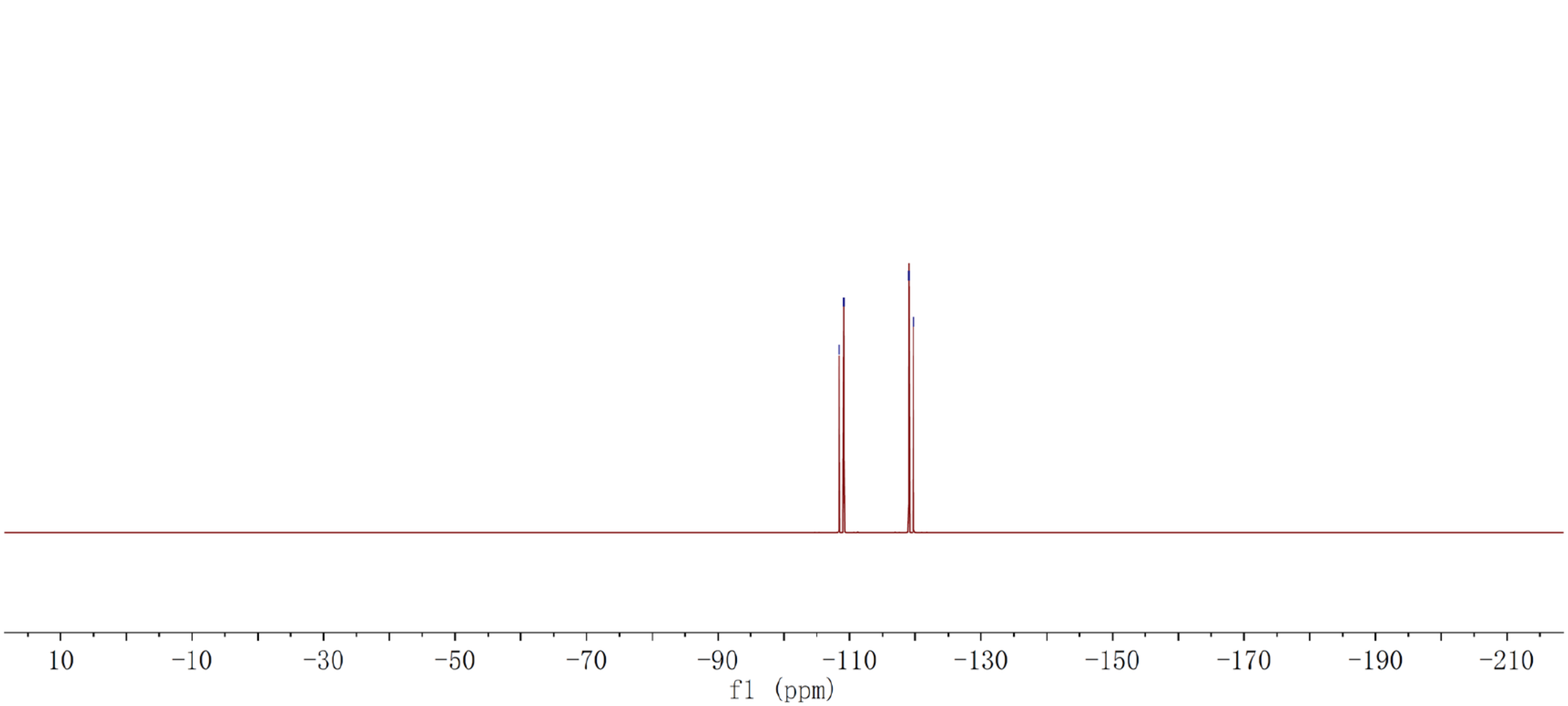

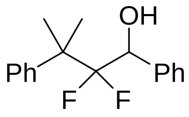

20

Supplementary Figure 265. ^{19}F NMR (376 MHz, $CDCl_3$) spectra for compound **20**

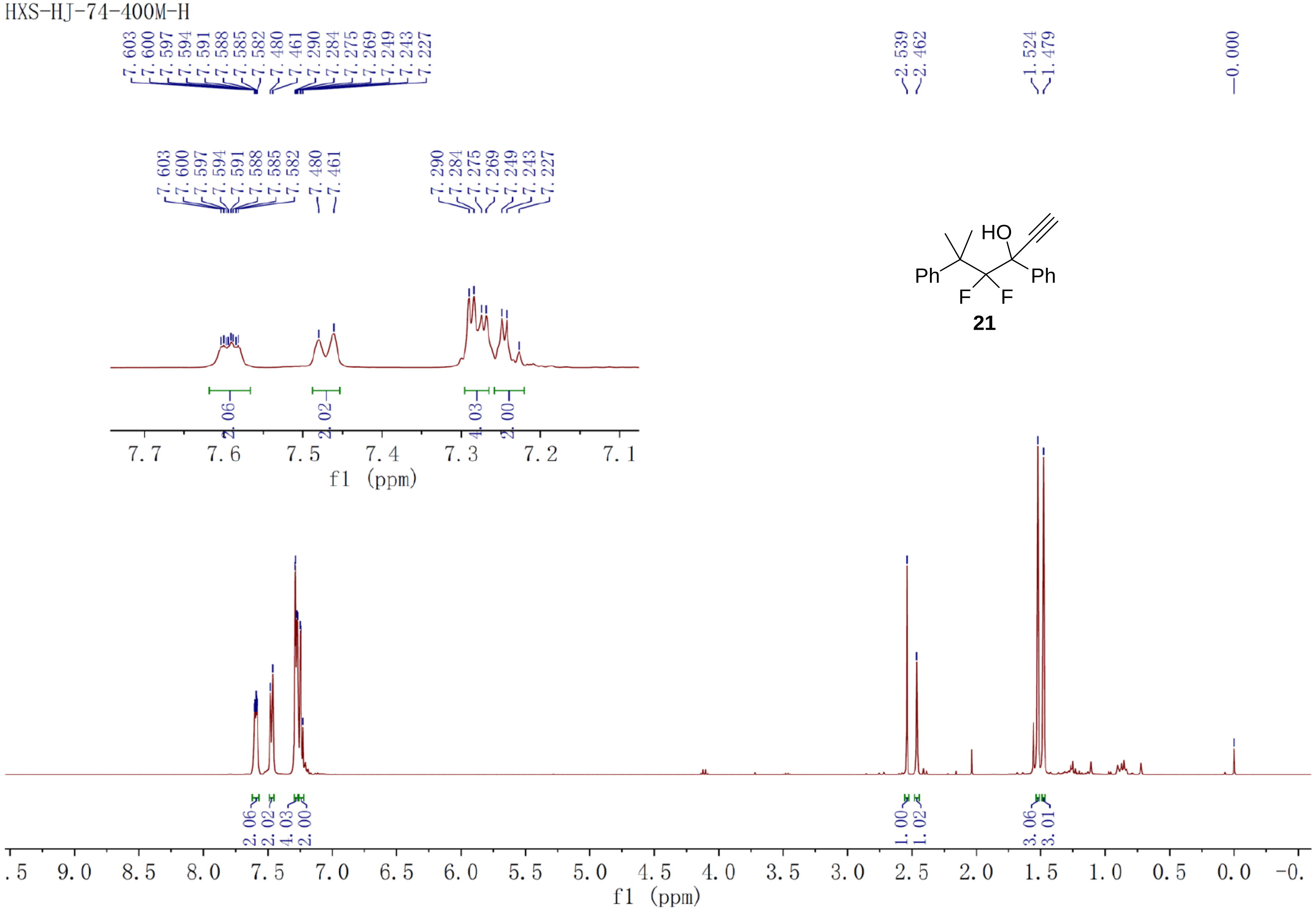

Supplementary Figure 266. ^{1}H NMR (400 MHz, $CDCl_3$) spectra for compound **21**

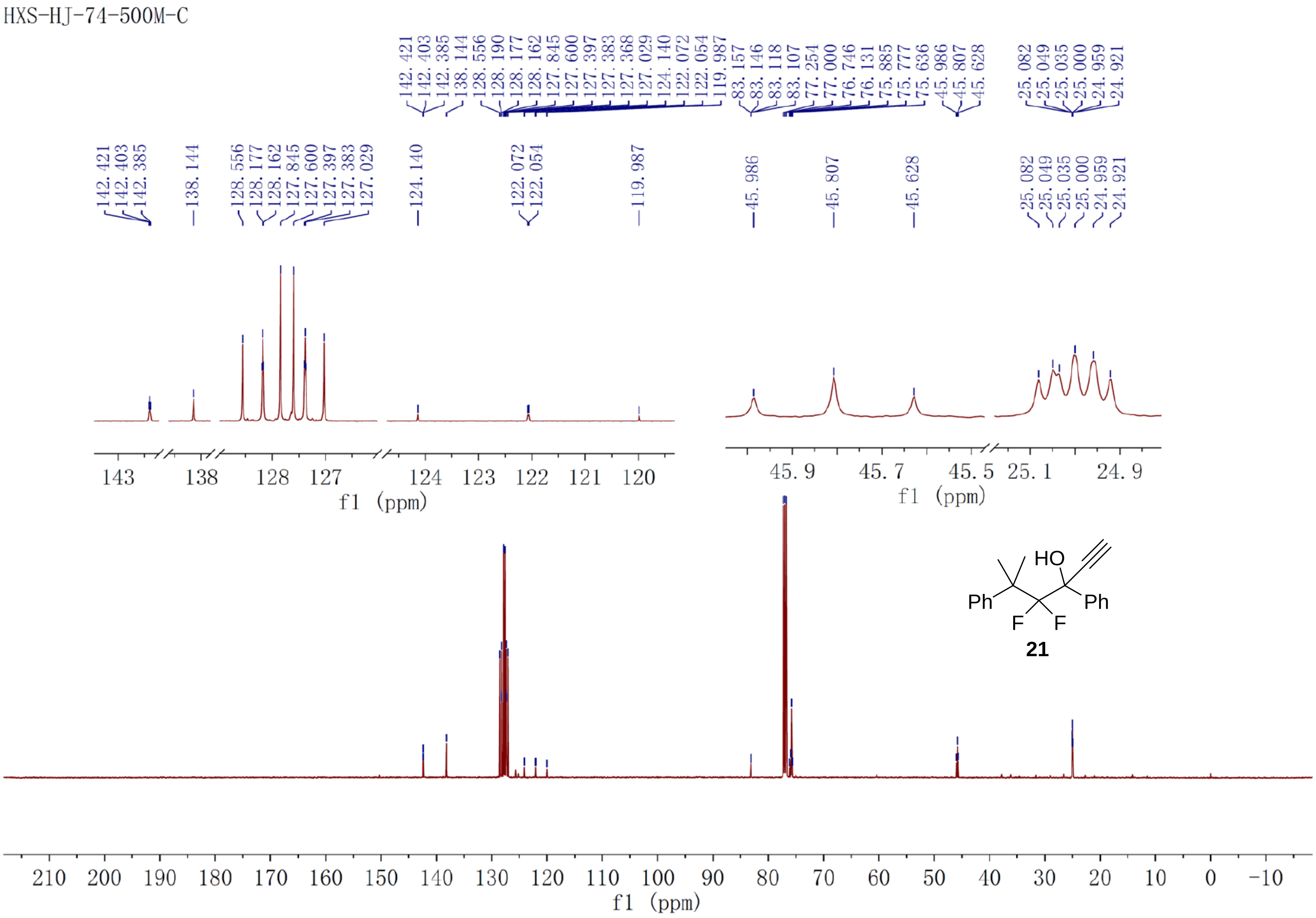

Supplementary Figure 267. ^{13}C NMR (125 MHz, $CDCl_3$) spectra for compound **21**

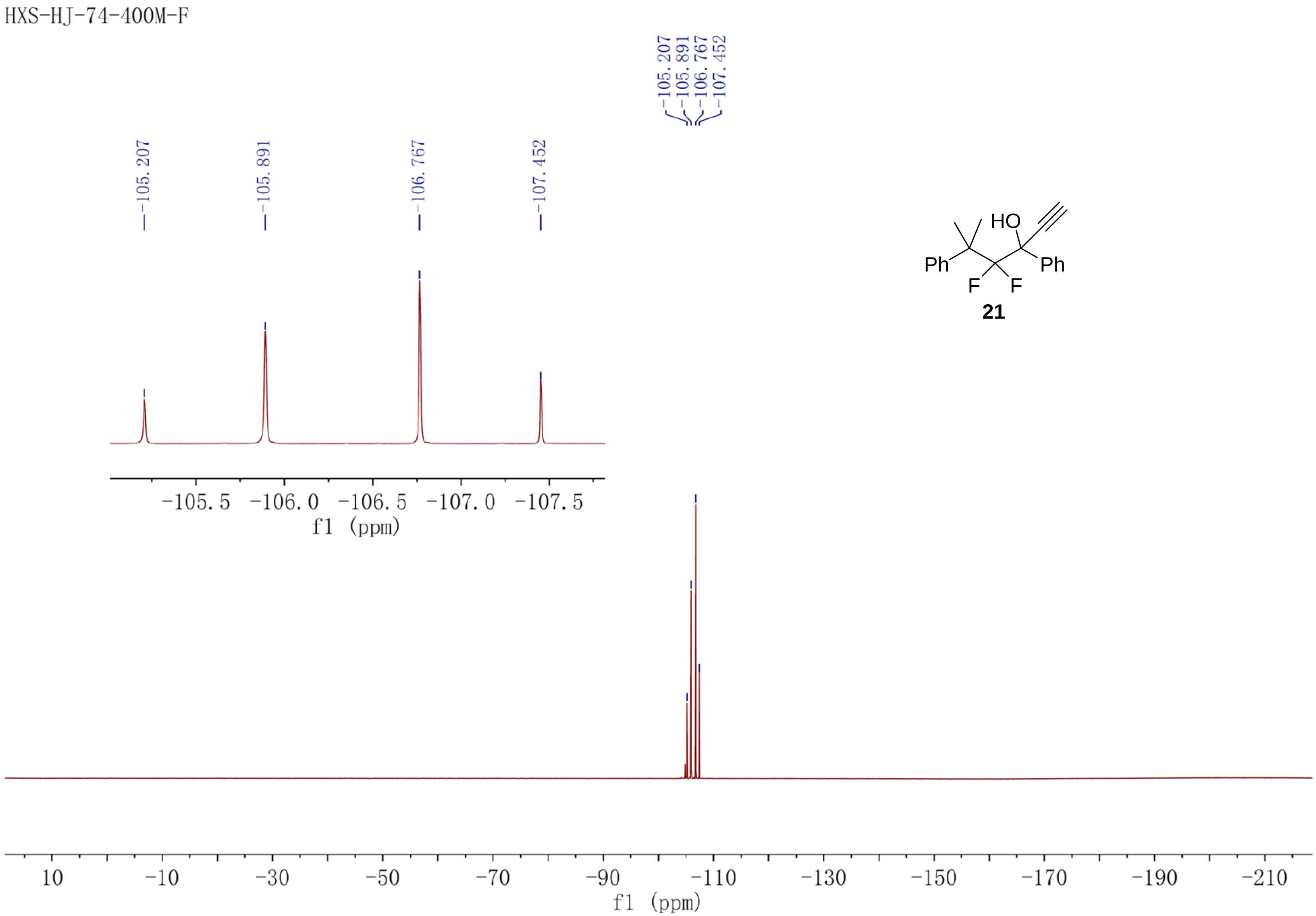

21

Supplementary Figure 268. ^{19}F NMR (376 MHz, $CDCl_3$) spectra for compound **21**

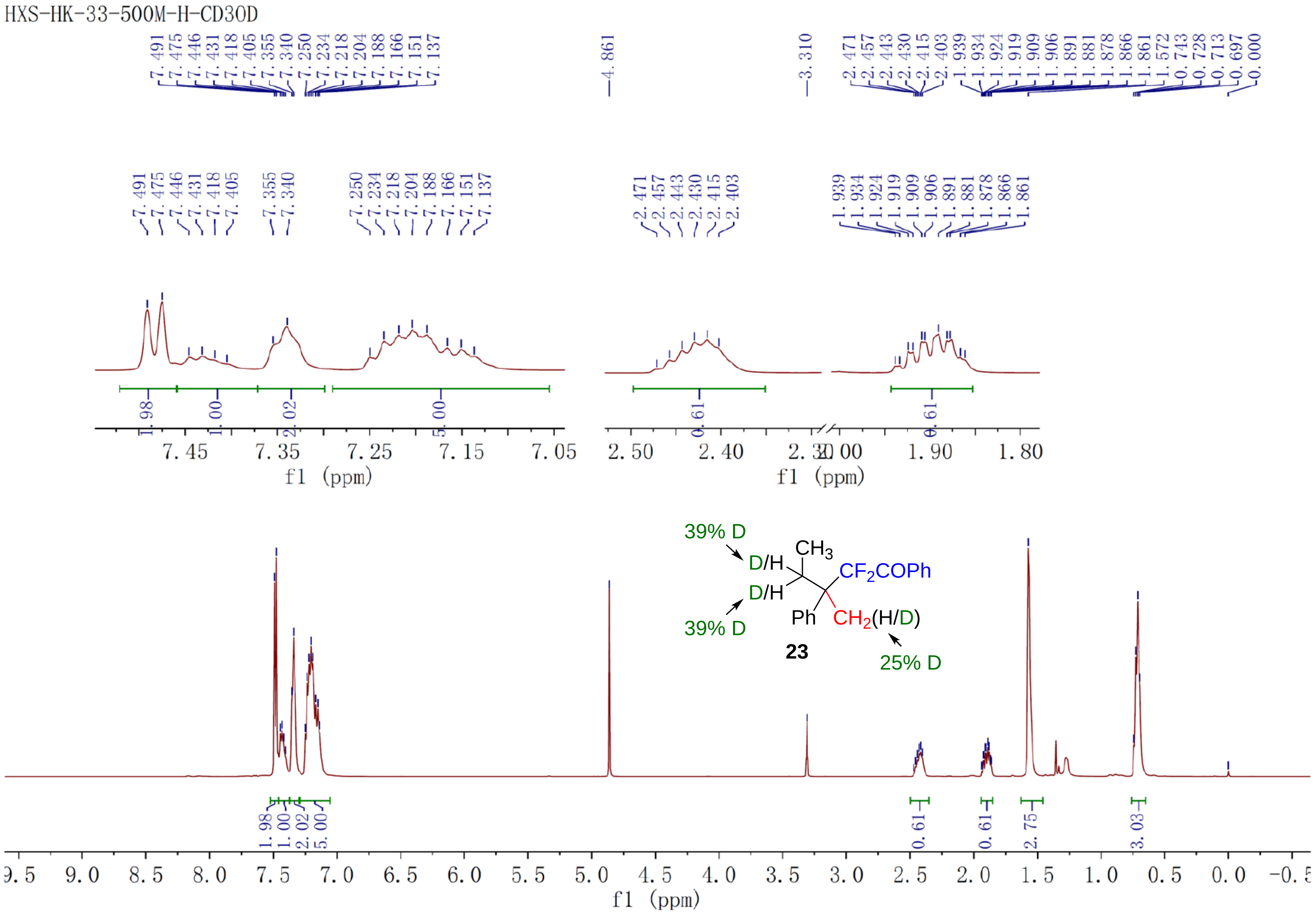

Supplementary Figure 269. ^{1}H NMR (500 MHz, CD_3OD) spectra for compound **23**

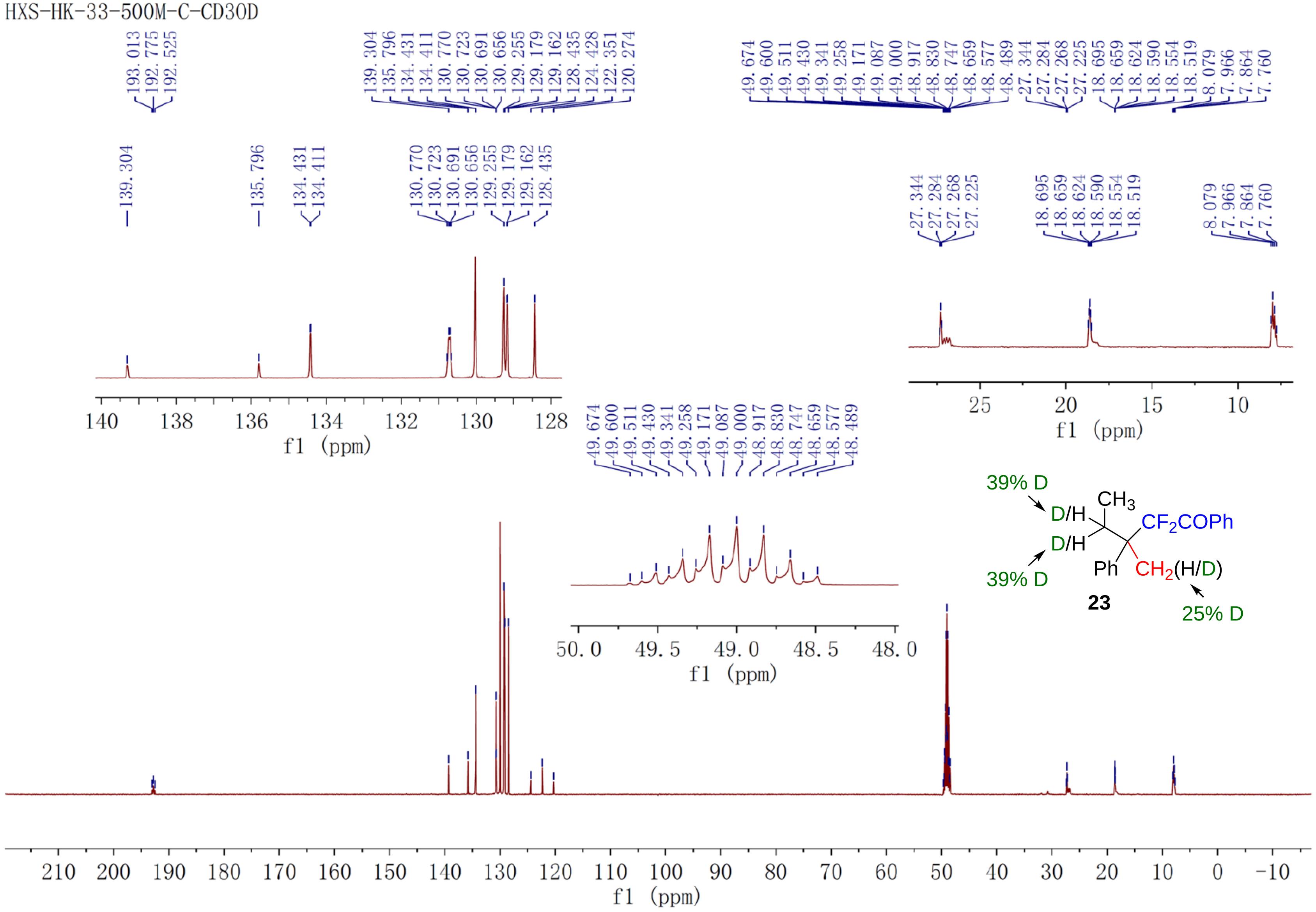

Supplementary Figure 270. ^{13}C NMR (125 MHz, CD_3OD) spectra for compound **23**

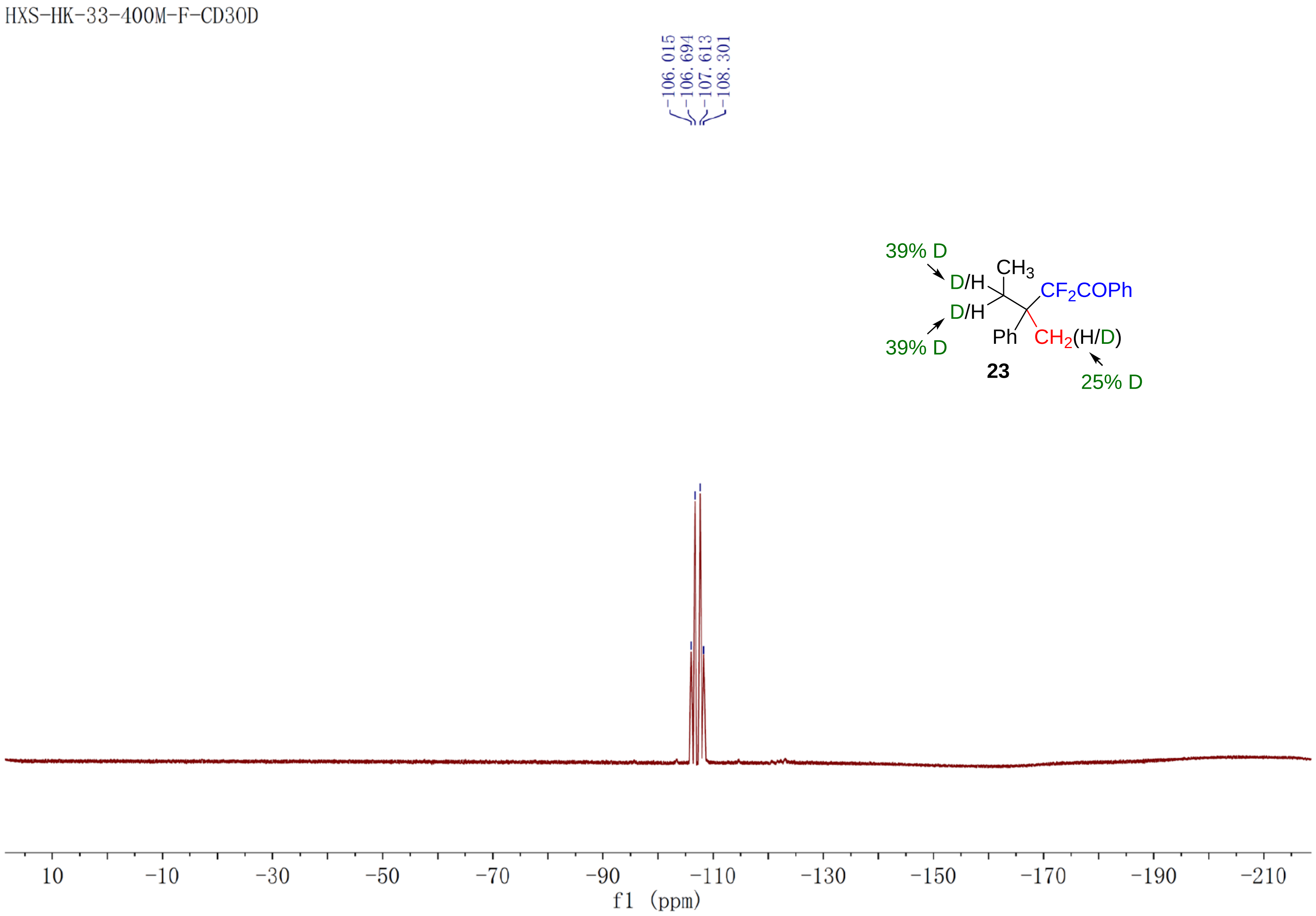

Supplementary Figure 271. ^{19}F NMR (376 MHz, CD_3OD) spectra for compound **23**

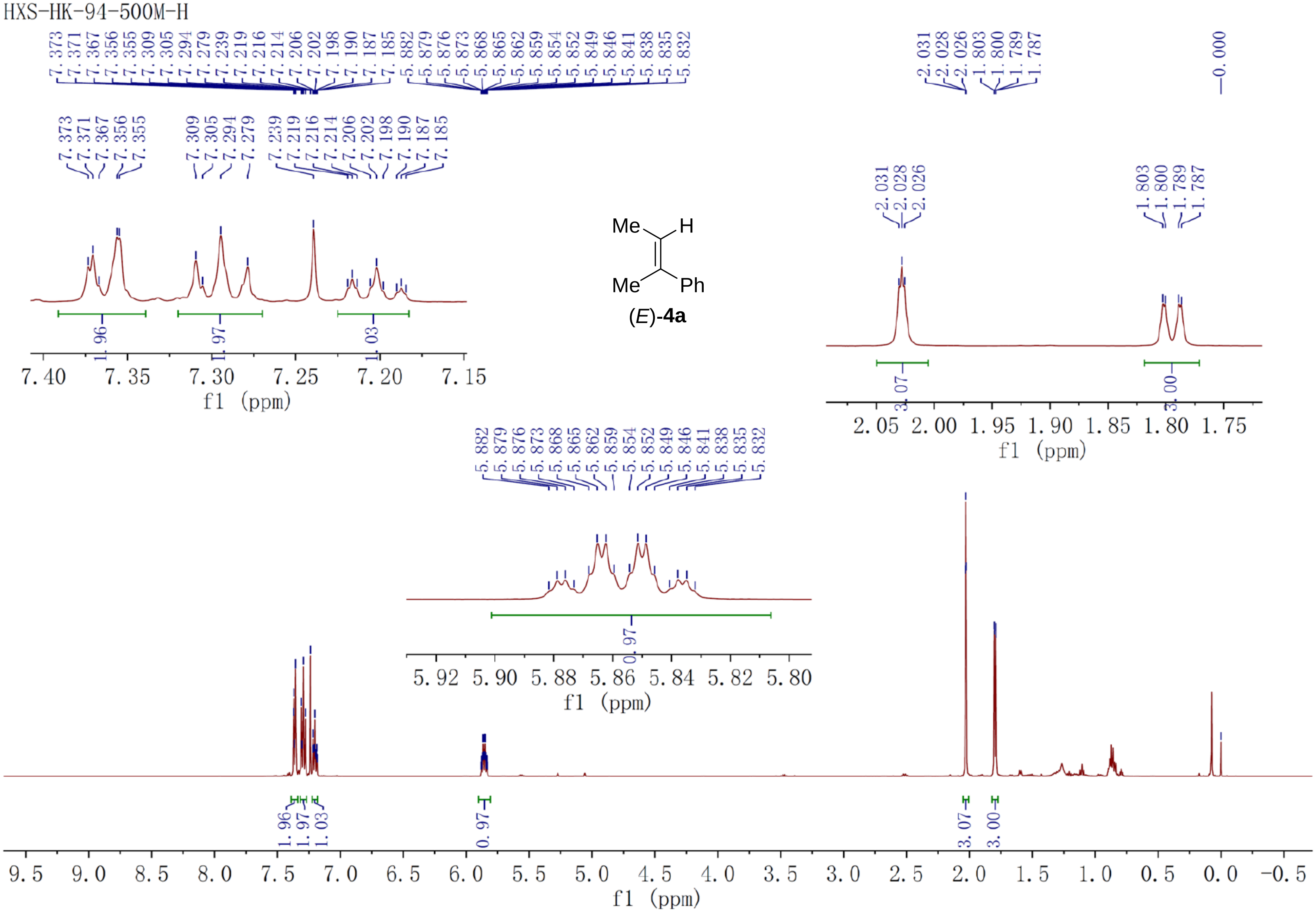

Supplementary Figure 272. ^{1}H NMR (500 MHz, $CDCl_3$) spectra for compound (*E*)-**4a**

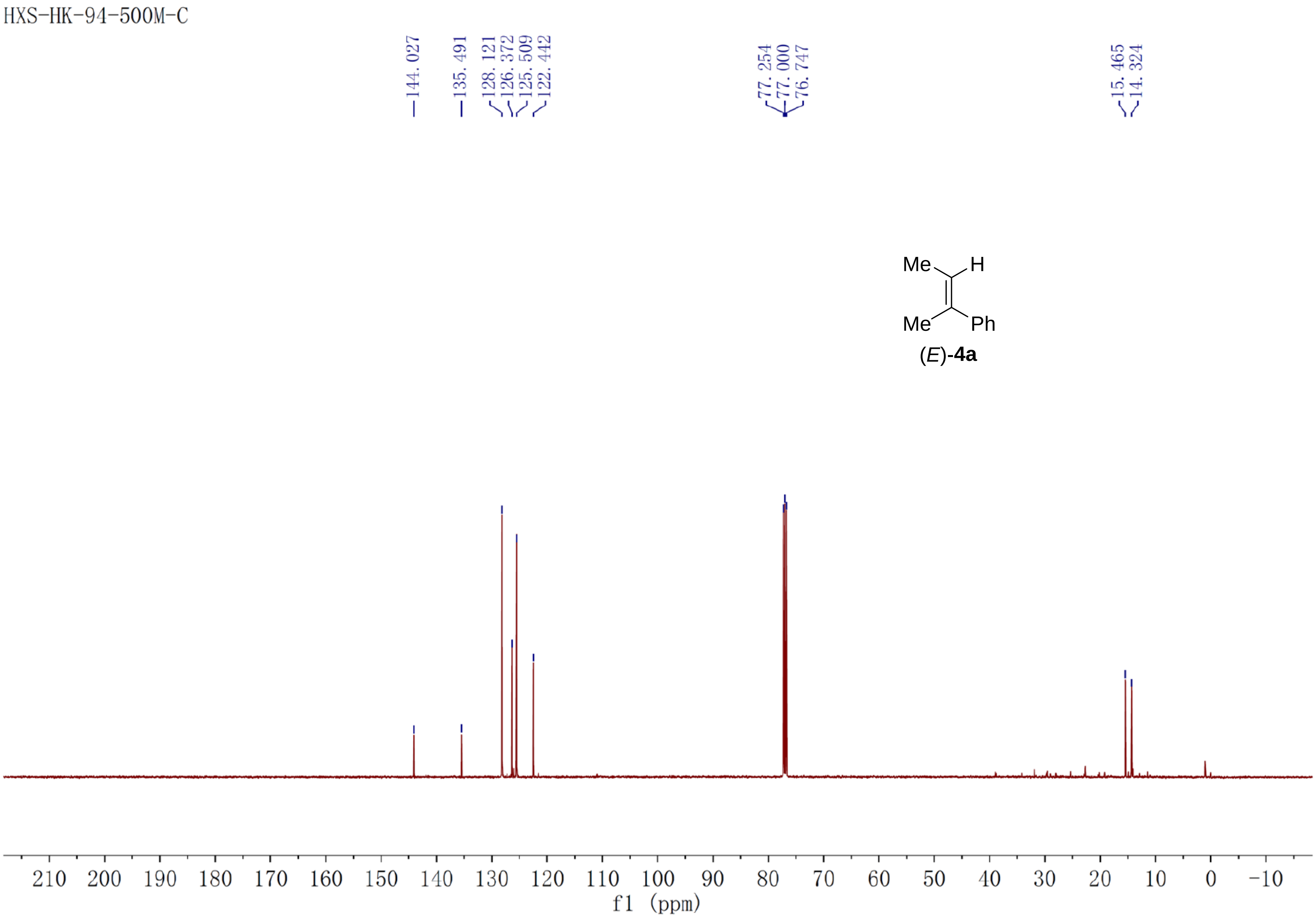

Supplementary Figure 273. ^{13}C NMR (125 MHz, $CDCl_3$) spectra for compound (*E*)-**4a**

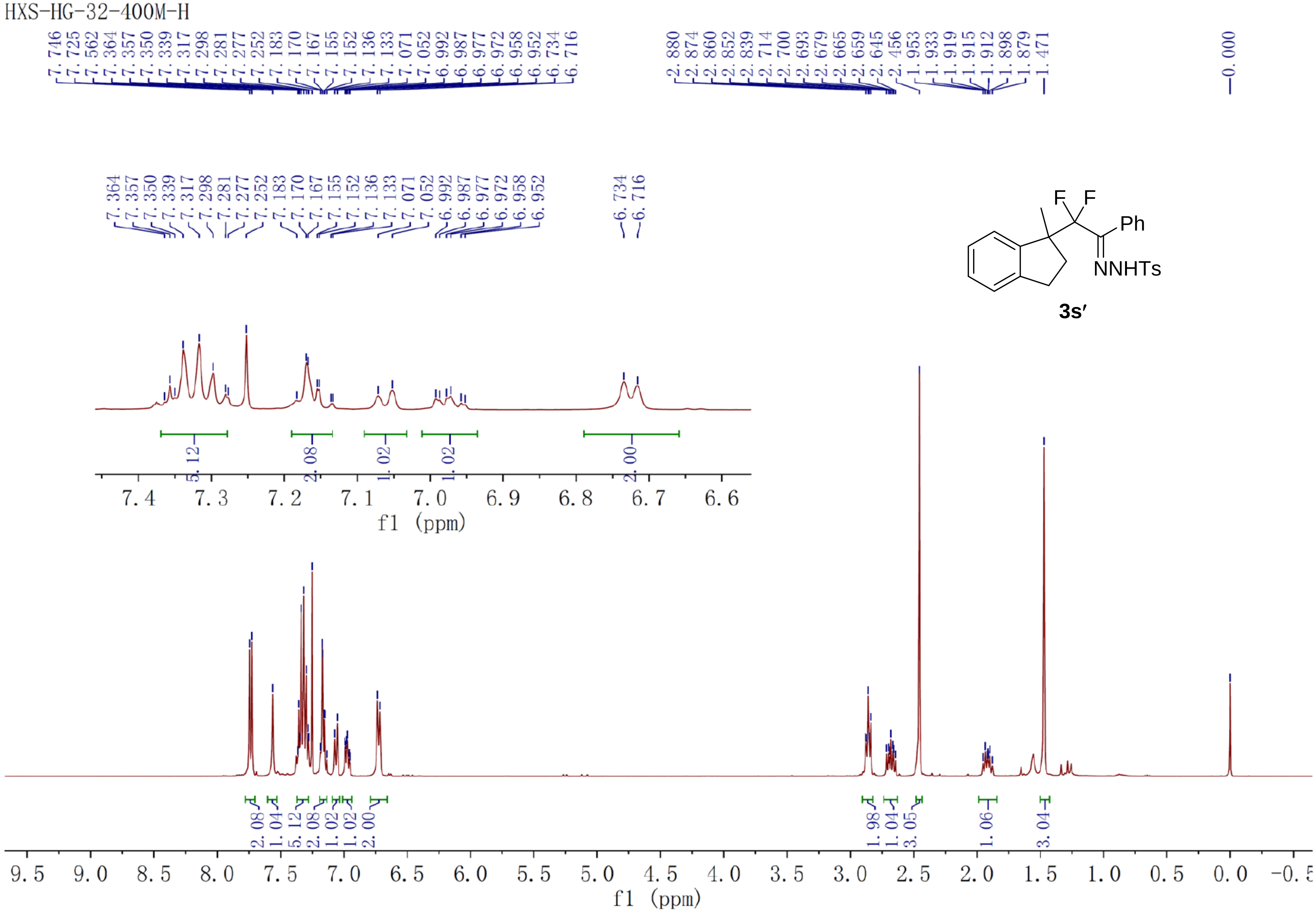

Supplementary Figure 274. ^{1}H NMR (400 MHz, $CDCl_3$) spectra for compound **3s'**

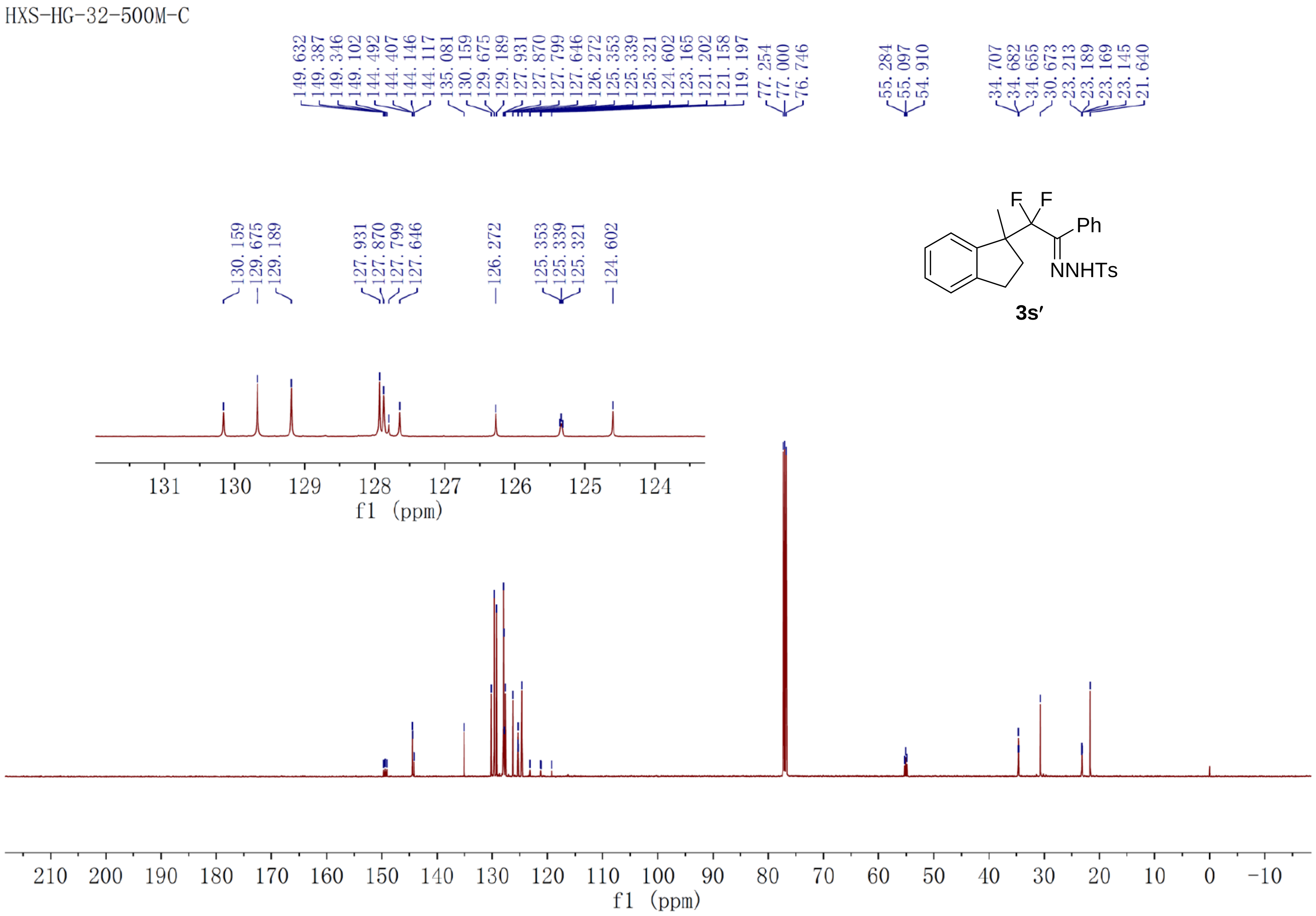

Supplementary Figure 275. ^{13}C NMR (125 MHz, $CDCl_3$) spectra for compound **3s'**

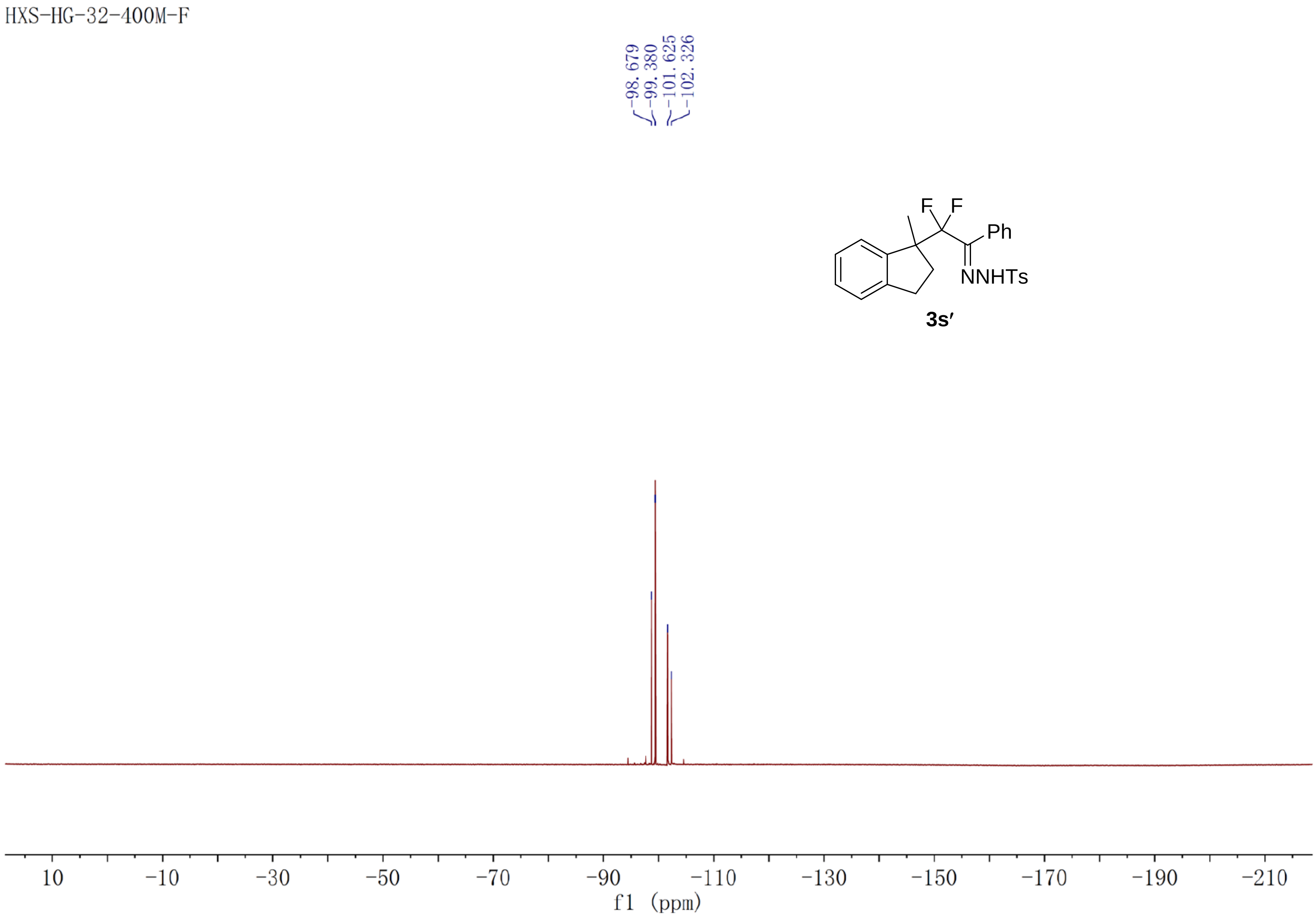

Supplementary Figure 276. ^{19}F NMR (376 MHz, $CDCl_3$) spectra for compound **3s'**

Supplementary References

1. Amii, H., Kobayashi, T., Hatamoto, Y. & Uneyama, K. Mg^0-promoted selective C–F bond cleavage of trifluoromethyl ketones: a convenient method for the synthesis of 2,2-difluoro enol silanes. *Chem. Commun.* 1323-1324 (1999).

2. Prakash, G. K. S., Hu, J. & Olah, G. A. Facile preparation of di- and monofluoromethyl ketones from trifluoromethyl ketones via fluorinated enol silyl ethers. *J. Fluorine Chem.* **112**, 355-360 (2001).

3. Liao, F.-M., Cao, Z.-Y., Yu, J.-S. & Zhou, J. Highly stereoselective gold-catalyzed coupling of diazo reagents and fluorinated enol silyl ethers to tetrasubstituted alkenes. *Angew. Chem. Int. Ed.* **56**, 2459-2463 (2017).

4. Bélanger, É., Cantin, K., Messe, O., Tremblay, M. & Paquin, J.-F. Enantioselective Pd-catalyzed allylation reaction of fluorinated silyl enol ethers. *J. Am. Chem. Soc.* **129**, 1034-1035 (2007).

5. Yu, J.-S., Liu, Y.-L., Tang, J., Wang, X. & Zhou, J. Highly efficient "on water" catalyst-free nucleophilic addition reactions using difluoroenoxysilanes: dramatic fluorine effects. *Angew. Chem. Int. Ed.* **53**, 9512-9516 (2014).

6. Eames, J., Coumbarides, G. S., Suggate, M. J. & Weerasooriya, N. Investigations into the regioselective *C*-deuteration of acyclic and exocyclic enolates. *Eur. J. Org. Chem.* 634-641 (2003).

7. Pratsch, G. & Overman, L. E. Synthesis of 2,5-diaryl-1,5-dienes from allylic bromides using visible-light photoredox catalysis. *J. Org. Chem.* **80**, 11388-11397 (2015).

8. Li, X.-T., Gu, Q.-S., Dong, X.-Y., Meng, X. & Liu, X.-Y. A copper catalyst with a cinchona-alkaloid-based sulfonamide ligand for asymmetric radical oxytrifluoromethylation of alkenyl oximes. *Angew. Chem. Int. Ed.* **57**, 7668-7672 (2018).

9. Xu, S., Wang, C., Komiyama, M., Tomonari, Y. & Negishi, E.-i. Asymmetric synthesis of chiral cyclopentanes bearing an all-carbon quaternary stereocenter by zirconium-catalyzed double carboalumination. *Angew. Chem. Int. Ed.* **56**, 11502-11505 (2017).

10. Gärtner, D., Stein, A. L., Grupe, S., Arp, J. & Wangelin, A. J. v. Iron-catalyzed cross-coupling of alkenyl acetates. *Angew. Chem. Int. Ed.* **54**, 10545-10549 (2015).

11. Friedfeld, M. R., Shevlin, M., Margulieux, G. W., Campeau, L.-C. & Chirik, P. J. Cobalt-catalyzed enantioselective hydrogenation of minimally functionalized alkenes: isotopic labeling provides insight into the origin of stereoselectivity and alkene insertion preferences. *J. Am. Chem. Soc.* **138**, 3314-3324 (2016).

12. Wang, G.-Z., Shang, R., Cheng, W.-M. & Fu, Y. Irradiation-induced Heck reaction of unactivated

alkyl halides at room temperature. *J. Am. Chem. Soc.* **139**, 18307-18312 (2017).

13. Bonvallet, P. A., Todd, E. M., Kim, Y. S. & McMahon, R. J. Access to the naphthylcarbene rearrangement manifold via isomeric benzodiazocycloheptatrienes. *J. Org. Chem.* **67**, 9031-9042 (2002).

14. Menichetti, S. & Stirling, C. J. M. Phenyl group acceleration of [1,4] carbon-to-oxygen silicon-mediated elimination-rearrangement in β-silyl sulfones. Synthesis of *O*-silylated cinnamyl alcohols. *J. Chem. Soc. Perkin Trans.* **1**, 28-30 (2002).

15. Noto, N., Koike, T. & Akita, M. Visible-light-triggered monofluoromethylation of alkenes by strongly reducing 1,4-bis(diphenylamino)naphthalene photoredox catalysis. *ACS Catal.* **9**, 4382-4387 (2019).

16. Fu, N., Sauer, G. S., Saha, A., Loo, A. & Lin, S. Metal-catalyzed electrochemical diazidation of alkenes. *Science* **357**, 575-579 (2017).

17. Wang, F. et al. Enantioselective copper-catalyzed intermolecular cyanotrifluoromethylation of alkenes via radical process. *J. Am. Chem. Soc.* **138**, 15547-15550 (2016).

18. Socolsky, C. et al. Effects of *p*-vinylphenyl glycosides and other related compounds on the oviposition behavior of ceratitis capitata. *J. Chem. Ecol.* **34**, 539-548 (2008).

19. Yang, B. & Lu, Z. Visible-light-promoted metal-free aerobic hydroxyazidation of alkenes. *ACS Catal.* **7**, 8362-8365 (2017).

20. Moria, Y. & Seki, M. A practical synthesis of multifunctional ketones through the Fukuyama coupling reaction. *Adv. Synth. Catal.* **349**, 2027-2038 (2007).

21. Zheng, Y.-Y., Guo, L., Zhen, X.-C. & Li, J.-Q. Synthesis and antidepressant activity of arylalkanol-piperidine derivatives as triple reuptake inhibitors. *Eur. J. Med. Chem.* **54**, 123-136 (2012).

22. Rao, X., Ishitani, H., Yoo, W.-J. & Kobayashi, S. Zirconium-β zeolite catalyzed continuous-flow Friedel-Crafts acylation reaction. *Asian J. Org. Chem.* **8**, 316-319 (2019).

23. Ren, R., Wu, Z., Xu, Y. & Zhu, C. C–C Bond-forming strategy by manganese-catalyzed oxidative ring-opening cyanation and ethynylation of cyclobutanol derivatives. *Angew. Chem. Int. Ed.* **55**, 2866-2869 (2016).

24. Norsikian, S. et al. Synthesis of enantioenriched 1,2-*trans*-diamines using the borono-Mannich reaction with *N*-protected α-amino aldehydes. *Chem. Commun.* **51**, 9991-9994 (2015).

25. Rudzinski, D. M., Kelly, C. B. & Leadbeater, N. E. A Weinreb amide approach to the synthesis of

trifluoromethylketones. *Chem. Commun.* **48**, 9610-9612 (2012).
26. Kobayashi, S., Tanaka, H., Amii, H. & Uneyama, K. A new finding in selective Baeyer-Villiger oxidation of α-fluorinated ketones; a new and practical route for the synthesis of α-fluorinated esters. *Tetrahedron* **59**, 1547-1552 (2003).
